# Transcriptome analysis and identification of genes related to environmental adaptation of *Grylloprimevala jilina* Zhou & Ren 2023

**DOI:** 10.1002/ece3.10717

**Published:** 2023-11-20

**Authors:** Yuxin Zhou, Lin Zhou, Qiuyao Li, Xiaoyan Zhu, Zhongbo Yu, Haoqin Ke, Qi Chen, Bingzhong Ren

**Affiliations:** ^1^ Jilin Provincial Key Laboratory of Animal Resource Conservation and Utilization Northeast Normal University Changchun China; ^2^ Key Laboratory of Vegetation Ecology, MOE Northeast Normal University Changchun China; ^3^ Istitude of Plant Protection Jilim Academy of Agricultural Science/Jilin Key Laboratory of Agricultural Microbiology/Key Laboratory of Integrated Pest Management on Crops in Northeast China Ministry of Agriculture and Rural Areas Gongzhling China

**Keywords:** cave insects, environmental adaptation, *Grylloprimevala jilina*, transcriptome

## Abstract

*Grylloprimevala jilina* is a true cave insect living in the dark areas of caves. It has the characteristics of sparse skin pigmentation, degeneration of the compound eyes and monocular eyes, and obvious preference for high‐humidity and low‐temperature environments. Given the highly specialized, rare, and limited distribution, *G. jilina* is considered an endangered species and also a first‐level national protected insect in China. Cave creatures often undergo dramatic morphological changes in their sensory systems to adapt to the cave environment. Most previous studies mainly focused on morphological adaptive changes in cave insects, and only a few studied the changes at the gene level. In this study, we performed transcriptome analysis of *G. jilina* and constructed phylogenetic trees of genes that are related to environmental adaptation, including chemosensory, visual‐related, reproduction‐related, temperature adaptation‐related, and winged morph differentiation‐related genes. Besides, the expression levels of environmental adaption‐related genes in different tissues, including antennae, heads, thoraxes, abdomens, legs, and tails, were analyzed. The results showed the loss of chemosensory genes and vision‐related genes, the conservation of reproduction‐related genes and temperature adaptation‐related genes, and the conservation of wing‐related genes despite the loss of wings, and the results were consistent with other cave insects. The identification and expression study of genes possibly related to the environmental adaptability in *G. jilina* provided basic data for the protection of this endangered species and increased knowledge about insect evolution in general.

## Graphical abstract

*Grylloprimevala jilina* is considered as an endangered species and also a first‐level national protected insect in China. we performed transcriptome analysis of *G. jilina* and constructed phylogenetic trees of genes that are related to environmental adaptation, and the expression levels of environmental adaption‐related genes in different tissues, including antennae, heads, thoraces, abdomens, legs, and tails, were analyzed. The identification and expression study of genes related to the environmental adaptability in *G. jilina* provided basic data for the protection of this endangered species and increase knowledge about insect evolution in general.

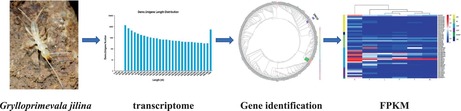

## 1 INTRODUCTION

Grylloblattodea, an extremely small monophyletic branch of the class Insecta, has low species diversity. It is the second order in the class Insecta after Mantophasmatidea. Grylloblattodea with ancient origin, dating back to the Carboniferous period (most abundant in the Permian and Triassic periods), is considered the “living fossil” of modern insects (Walker, 1937). A study believes that Grylloblattodea, which is the only existing ancient remnant group of Insects, is one of the most important living insects, due to Grylloblatta is an extremely primitive representative of the Orthopteroid group, and the study of this insect is of the greatest importance in the consideration of the evolution of the group as a whole (Crampton, 1926). During geological evolution, Grylloblattodea has experienced a series of morphological changes, including size change from large to small and winged to wingless, and distribution changes from wide to narrow, becoming extremely sensitive to temperature, humidity, and other environmental factors (Bai et al., 2010). Therefore, the order Periplaneta is an important material for the study of insect origin, evolution, environmental adaptation mechanism, and its relationship with geological history.

Caves are a special geological environment with limited food resources, low light and temperature, and high humidity (Fernandes et al., 2016). Adaptations to cave environments are often accompanied by dramatic morphological changes in the sensory systems of cave creatures (Balart‐García et al., 2021). For example, the evolution of taste buds and olefin nerve balls, loss of eyes, and hyperpigmentation were noticed in some cave fishes compared with those living above‐ground; elongation of antennae and body appendages were found in *Asellus aquaticus* (Turk et al., 1996); loss of thermal acclimation capacity was found in cave beetles (Rizzo et al., 2015). Such adaptive changes in cave creatures are essentially genetic changes (Howarth, 2019). In recent years, with the development of sequencing technology, some cave insects have been studied at the genome and transcriptome level (Balart‐García et al., 2022; Stern & Crandall, 2018). However, less research has been done at the transcriptome or genome level.

*Grylloprimevala jilina* is the national first‐class protected insect and there are only three Grylloblattodea species in China. *G. jilina* is a true cave creature living in the dark areas of caves. It has rare body pigment, degeneration of compound and monocular eyes, body surface sensors, and an obvious preference for high‐humidity and low‐temperature environments (Zhou et al., 2023). *G. jilina* is considered endangered because of its very limited and highly specific distribution (Zhou et al., 2023). However, so far, the limited studies on *G. jilina* mainly focused on its systematic classification. Therefore, studying the *G. jilina* genes related to environmental adaptability and identifying those involved in the regulation of related phenotypic characteristics will help understand its adaptation to the cave environment and provide basic knowledge for its protection and cave insect evolution in general.

## 2 MATERIALS AND METHODS

### 2.1 Insects

**FIGURE 1:**
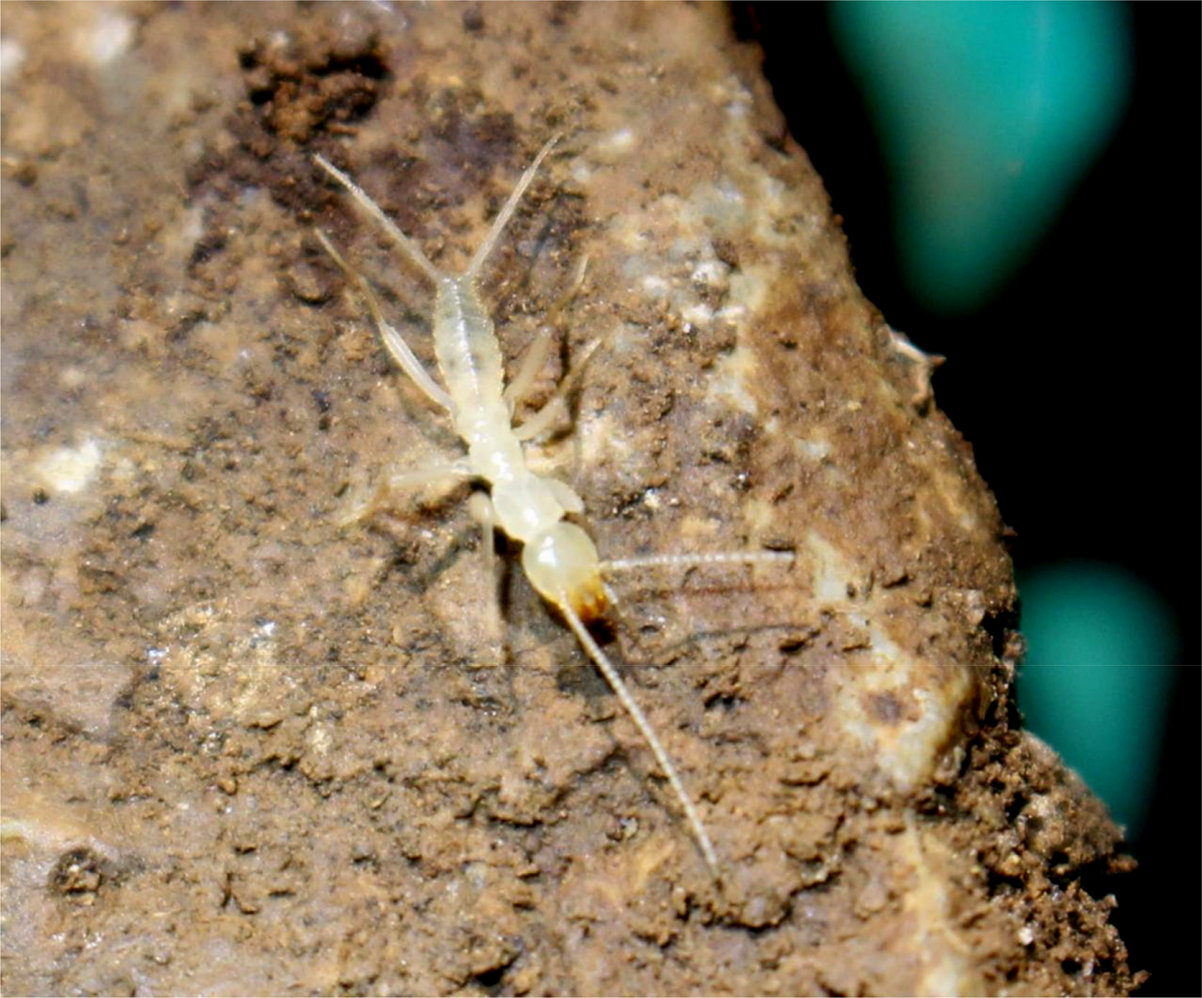
*Grylloprimevala jilina*'s photograph. *G. jilina* was photographed in the natural caves of the pristine forest in Ji'an City, Jilin Province, China.

*G. jilina* was collected from a rock pile in a dark area of a natural cave in a primitive forest of Ji'an City, Jilin Province, China (Figure 1).

### 2.2 RNA‐Seq library construction and sequencing

*G. jilina* has Class I national protected status, thus only one sample was used for the transcriptome analysis. The samples were prepared from different tissues (antennae, heads, thoraxes, abdomens, legs, and tails) of *G. jilina* and frozen in liquid nitrogen before storage at −80°C until RNA extraction. Total RNA was extracted from tissue samples using TRIzol reagent (Invitrogen, Carlsbad, CA, USA) following the manufacturer's protocol. The integrity of the RNA was determined by a NanoDrop 2000 (ThermoFisher Scientific, Waltham, MA) and 1% agarose gel electrophoresis (Wen et al., 2019).

Transcriptome sequencing of six different tissues of *G. jilina* was performed using the second generation of high‐throughput sequencing technology (Illumina NovaSeq platform) by Beijing Baimaike Biotechnology Co., Ltd., China. Sequencing libraries were prepared using the NEBNext® Ultra™ RNA Library Prep Kit for Illumina® (NEB, USA) according to the manufacturer's recommendations, and index codes were added to attribute sequences to each sample. The libraries were sequenced on an Illumina HiSeq 2000 PE150 platform, and then the 150 bp paired‐end reads were generated. The raw reads were finally obtained.

### 2.3 Transcriptome assembly and annotation of functional gene

The junction sequences and those of low quality were removed to obtain effective sequence clean reads. The left.fq and right.fq functions in Trinity (r20131110) were applied with all default parameters for transcriptome assembly. Total reads from all the samples were assembled into one transcriptome. The Trinity_cluster_with_id_com Pl was used for clustering the transcripts. Unigenes were defined as the longest isoform from each trinity assembly (Grabherr et al., 2011). Total reads the final unigene data set was generated. BUSCO v. 3.0.2 was used for the evaluation of the quality of the constructed transcriptome sequences (Simão et al., 2015). DIAMOND (Buchfink et al., 2015) software was used to compare the obtained Unigenes in the NCBI nonredundant (NR) database (Deng et al., 2006). Besides, eggNOG4.5 (Huerta‐Cepas et al., 2016), COG (Clusters of Orthologous Groups) (Tatusov et al., 2000), Pfam (Protein family) (Finn et al., 2014), GO (Gene Ontology) (Ashburner et al., 2000), Swiss‐Prot (a manually annotated and reviewed protein sequence database, http://www.expasy.ch/sprot), KOG (EuKaryotic Orthologous Groups) (Koonin et al., 2004), and KEGG (Encyclopedia of Genes and Genomes) (Kanehisa et al., 2004) databases were used for the functional annotation of Unigenes.

### 2.4 Identification of genes related to environmental adaptation

*G. jilina* chemosensory genes, visual‐related genes, reproduction‐related genes, and temperature adaptation‐related genes were preliminarily screened based on the annotation results of the transcriptome database. When selecting these genes, we considered two main factors. First, these genes play significant roles in insect environmental adaptation. Second, the functions of these genes have been well established in other insects rather than being speculative. In the category of chemosensory genes, we have identified odorant receptors (ORs), gustatory receptors (GRs), ionotropic receptors (IRs), odorant binding proteins (OBPs), chemosensory proteins (CSPs), and sensory neuron membrane protein (SNMP) (Leal, 2013; Sánchez‐Gracia et al., 2009). In the context of visual‐related genes, we identified visual system homeobox (VSX) (Sanes & Zipursky, 2010). For reproduction‐related genes, we identified spire, vitellogenins (Vgs), selenoprotein F (SPFs), Tudor, doublesex (Dmrt), sex peptide receptors (SPs), Soxes, and vasa (Gustafson & Wessel, 2010; Li et al., 2020; Manseau & Schüpbach, 1989; Poels et al., 2010; Wilson & Dearden, 2008). In terms of temperature adaptation‐related genes, we identified Trehalases (Trets), heat shock proteins (HSP), DnaJ, and transient receptor potential (TRPs) (Chen et al., 2021; Liu & Zhang, 2022; Tang et al., 2018). Last, for winged morph differentiation‐related genes, we identified six wingless (Wnt), ecdysone receptors (ECRs), ecdysone‐inducible protein E75, insulin receptor (InsR), insulin‐like growth factor‐binding proteins (IGFBPs), insulin‐like peptide (ILP), juvenile hormone esterase (JHE), juvenile hormone‐binding proteins (JHBPs), protein germ cell‐less (CGL), methoprene‐tolerant (Met), and epidermal growth factor receptor (EGFR) (Corona et al., 2016; Hayes et al., 2019; Llimargas & Lawrence, 2001; Truman, 2019). Combining annotation results from transcriptome database and manual annotation using the basic local comparison search tool (NCBI‐blast‐2.11.0), *G.jilina* chemosensory genes, vision‐related genes, reproduction‐related genes, and temperature adaption‐related candidate genes were initially screened. Based on the final annotation results, the ORF finder tool (https://www.ncbi.nlm.nih.gov/orffinder/) was used to predict the respective open reading frames (ORF) and amino acid sequences of gene transcripts. Finally, the obtained sequences were submitted to NCBI BLAST (https://blast.ncbi.nlm.nih.gov/Blast.cgi) and manually rechecked against the GenBank nonredundant (NR) protein database using the BLASTx program (*E*‐value <10‐5).

### 2.5 Phylogenetic analysis

Phylogenetic trees were constructed for the analyses of environmental adaptation‐related genes using corresponding amino acid sequences from other insects. All the used amino acid sequences in this study are presented in the Appendix S1. Using the “One Step Build a ML Tree” function of TBtools (Zhu et al., 2022), the Muscle program was used for multiple sequence alignment, TrimAl was used to prune the alignment results, and finally, IQ‐tree was used to automatically screen the amino acid replacement model to build the maximum likelihood (ML) trees (Chen et al., 2020), which were visualized with FigTree v1.4. (Drummond et al., 2012).

### 2.6 Transcriptome composition analysis

The sequencing reads were aligned with the Unigenes dataset using Bowtie (Langmead et al., 2009), and their expression levels were evaluated by RSEM (Li & Dewey, 2011). The fragments per kilobase of exon model per million (FPKM) mapped fragments were used to represent the expression abundance of the corresponding gene transcripts (Andersson et al., 2014; Leitch et al., 2015). The clustering heat map tool (https://hiplot.com.cn/cloud‐tool/drawing‐tool/detail/106) was used to construct heatmaps of differentially expressed chemosensory genes based on Log10[FPKM+1] values.

## 3 RESULTS

### 3.1 Tissue‐specific transcriptome of *G. jilina*

In this study, we performed RNA extraction from six different tissue samples of *G. jilina* for double‐end sequencing of corresponding cDNA libraries using the Illumina NovaSeq SBS technique. From the six samples, a total of 45.16 Gb effective sequencing data were obtained, a minimum of 5.79 Gb from each sample (PRJNA951510). There were 151,402,042 clean read pairs (Table 1) and 48,071 Unigenes, including 16,227 Unigenes larger than 1 kb in length (Table S1 and Figure S1). The result of BUSCO for the transcriptome data is 83.5% [S: 71.3%, D: 12.2%], F: 7.6%, M: 8.9%, n: 303 (C: complete BUSCOs, S: complete and single‐copy BUSCOs, D: complete and duplicated BUSCOs, F: fragmented BUSCOs, M: missing BUSCOs, n: total BUSCO groups searched). Sequence similarity searches against nine public databases (NR, Swiss‐Prot, TrEMBL, KEGG, COG, KOG, GO, eggNOG, and Pfam) were used for sequence annotation. The results showed that a total of 20,517 (42.68%) unigenes were successfully annotated in these databases, of which 17,958 (37.36%), 17,928 (37.29%), and 15,711 (32.68%) were significantly matched in the TrEMBL, NR, and GO databases, respectively (Table S2).

**TABLE 1:** Transcriptome assembly summary of *Grylloprimevala jilina.*

Sample tags	Clean reads	Clean bases	Error rate (%)	Q30	GC content
Antennae	23,183,235	6,918,562,838	0.01	94.06	37.76
Heads	22,565,753	6,714,056,742	0.01	94.29	40.63
Thoraxes	20,401,289	6,092,396,242	0.01	94.09	42.86
Legs	20,998,628	6,267,080,942	0.01	93.79	38.94
Abdomens	22,412,321	6,683,763,696	0.01	94.01	45.44
Tails	19,402,525	5,794,384,278	0.01	93.27	43.15

### 3.2 Identification and homology analysis of chemosensory genes

**FIGURE 2:**
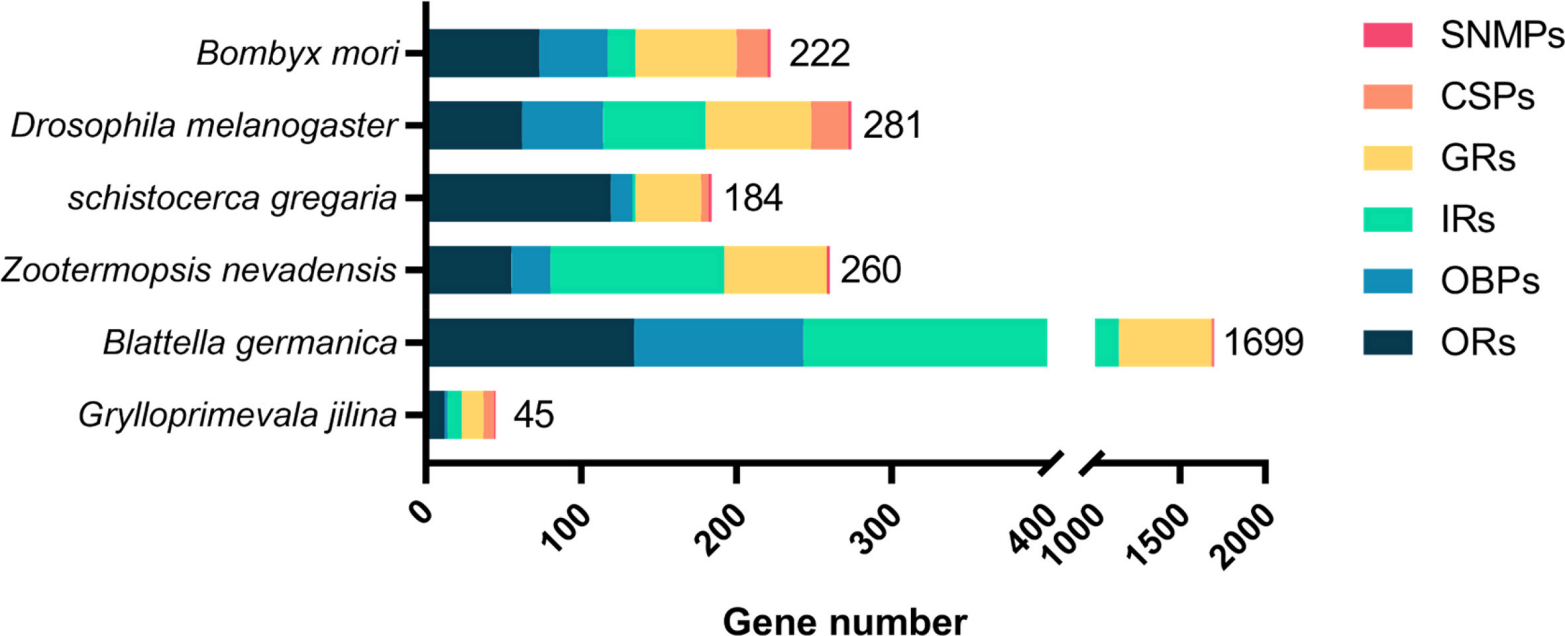
Comparison of chemosensory genes in different species.

From the transcriptome sequencing of *G. jilina*, a total of six chemosensory gene families were screened. Twelve ORs were identified, encoding 45–472 amino acids (aa), with a homologous similarity of 28.82%–68.5%. *GjilORCO* and other homologous genes clustered into a single branch, and their similarity with *ZnevORCO* was 68.5% (Table S3 and Figure S2). Two GRs were identified, and their homologous similarity with known insects was 40.51%–42.35% (Table S4 and Figure S3). Nine IRs were identified and they have a homologous similarity between 33.33 and 90.30%. IR8a is 90.30% similar to other species and clustered with *DmelIR8a*, *BmorIR8a*, *SgreIR8a*, and *LmigIR8a*. *GjilIR93a* clustered with *DmelIR93a*, *BmorIR93a*, and *ZnevIR93* in the 93a family (Table S5 and Figure S4). In total, 14 OBPs were identified with amino acid lengths of 141–286. *GjilOBP14a* and *GjilOBP14b* clustered as independent branches. Also, *GjilOBP22a* did not cluster with other species (Table S6 and Figure S5). Seven CSPs were identified with amino acid lengths 111–135; *GjilCSP19* did not cluster with other species genes (Table S7 and Figure S6). One SNMP of 513 aa was identified, which is 51.22% similar to other homologs (Table S8 and Figure S7). In total, 46 chemosensory genes (12 ORs, 2 GRs, 9 IRs, 14 OBPs, 7 CSPs, and 1 SNMP) were identified in *G. jilina*, which are less than those in model insect *Drosophila melanogaster* and most other insects of the related order Blattaria (Figure 2).

### 3.3 Identification and homology analysis of visual‐related genes

**FIGURE 3:**
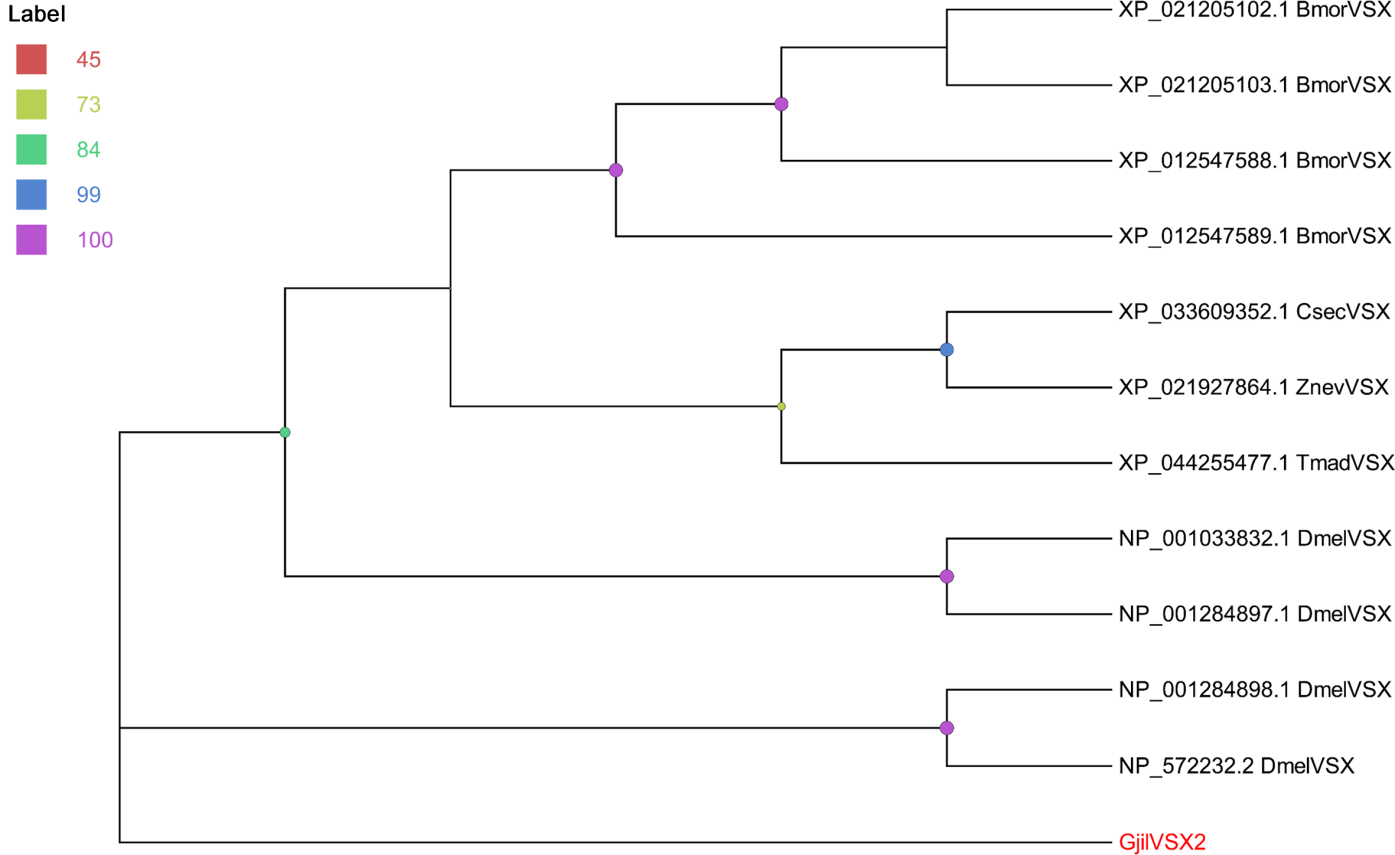
Homology analysis of VSXs from *Grylloprimevala jilina* and other representative insect species. Among them, the homologous genes of other species are screened from the NCBI genome. Bmor: *B. mori* (GCF_014905235.1), Csec: *Cryptotermes secundus* (GCF_002891405.2), Dmel: *D. melanogaster* (GCF_000001215.4), Tmad: *Tribolium madens* (GCF_015345945.1), Znev: *Zootermopsis nevadensis* (GCF_000696155.1).

We only identified VSX in the visual‐related genes, *GjilVSX2* with an amino acid length of 138 aa, was identified. The highest similarity with other homologs was 50.42%, and it did not cluster into a single branch with other genes; the genes from other insects included *Bombyx mori*, *Cryptotermes secundus*, *D. melanogaster*, *Tribolium madens*, and *Zootermopsis nevadensis* (Table S9 and Figure 3).

### 3.4 Identification and homology analysis of reproduction‐related genes

Seven reproduction‐related genes or gene families were screened, including four Vgs with 32.84%–97.81% homology similarity compared with other insects (Table S11 and Figure S9). Three SPF genes, encoding 86–162 aa, were identified (Table S12 and Figure S10). One Dmrt gene, encoding 265 aa, with 67.59% homologous similarity to known insects' genes, was identified (Table S14 and Figure S12). Three SPs, encoding 344–448 aa, have 65.97%–88.44% similarity with other species (Table S15 and Figure S13). Six Soxes were screened that encode 63–562 aa and have 40.29%–80.35% similarity with other species (Table S16 and Figure S14). The data of spires, Tudor, and vasa genes were in the supplement information.

### 3.5 Identification and homology analysis of temperature adaptation‐related genes

Four temperature adaptation‐related gene families were screened. The protein lengths of 33 identified Trets ranged from 86 to 871 aa, which were 34.83%–95.51% similar to each other and those from other species. *GjilTret1‐c153918*, *GjilTret1‐c154529*, *GjilTret1‐c152950*, and *GjilTret1‐c158653* clustered into one branch (Table S18 and Figure S16). TRP genes were identified, which encode 93–1286 aa. *GjilTRPV‐c157412*, *GjilTRPC‐c181968*, *GjilTRPV‐c152094*, and *GjilTRPV‐c80333* were part of an expanded cluster (Table S21 and Figure S19). The data of HSPs and DnaJs were in the supplement information.

### 3.6 Identification and homology analysis of winged morph differentiation‐related genes

Eleven genes or gene families related to winged morph differentiation were screened; six Wnt genes, encoding 93–391 aa, were identified (Table S22 and Figure S20). Two types of EcR pathways were identified, including two ECRs and one E75. The amino acid length of EcRs is 87–366, having 66.28%–77.92% similarity to other species (Table S23 and Figure S21). E75 is 90 aa long with 76.47% similarity to other species (Table S24 and Figure S22). Concerning InsR, four IGFBPs were identified. The aa length of InsR is 1688, which is 64.15% similar to other species (Table S25 and Figure S23). The length of IGFBPs is 524–761 aa, with 35.56%–87.02% similarity to other species (Table S26 and Figure S24). Four types of juvenile hormone pathway genes were identified, including one ILP, one JHE, three JHBPs, one CGL, and one Met. ILP gene encodes 135 aa and is 57.78% similar to other species (Table S27 and Figure S25). JHE gene encodes 60 aa and is 61.67% similar to other species (Table S28 and Figure S26). JHBPs gene encodes 245–275 aa and is 29.26%–88.36% similar to other species (Table S29 and Figure S27). CGL protein is 424 aa long and is 51.54% similar to other species (Table S30 and Figure S28). The met gene encodes 634 aa and is 47.28% similar to other species (Table S31 and Figure S29). One EGFR gene, encoding 1418 aa, is 76.50% similar to other species (Table S32 and Figure S30).

### 3.7 Analysis of gene expression profile related to environmental adaptation of *G. jilina*

**FIGURE 4:**
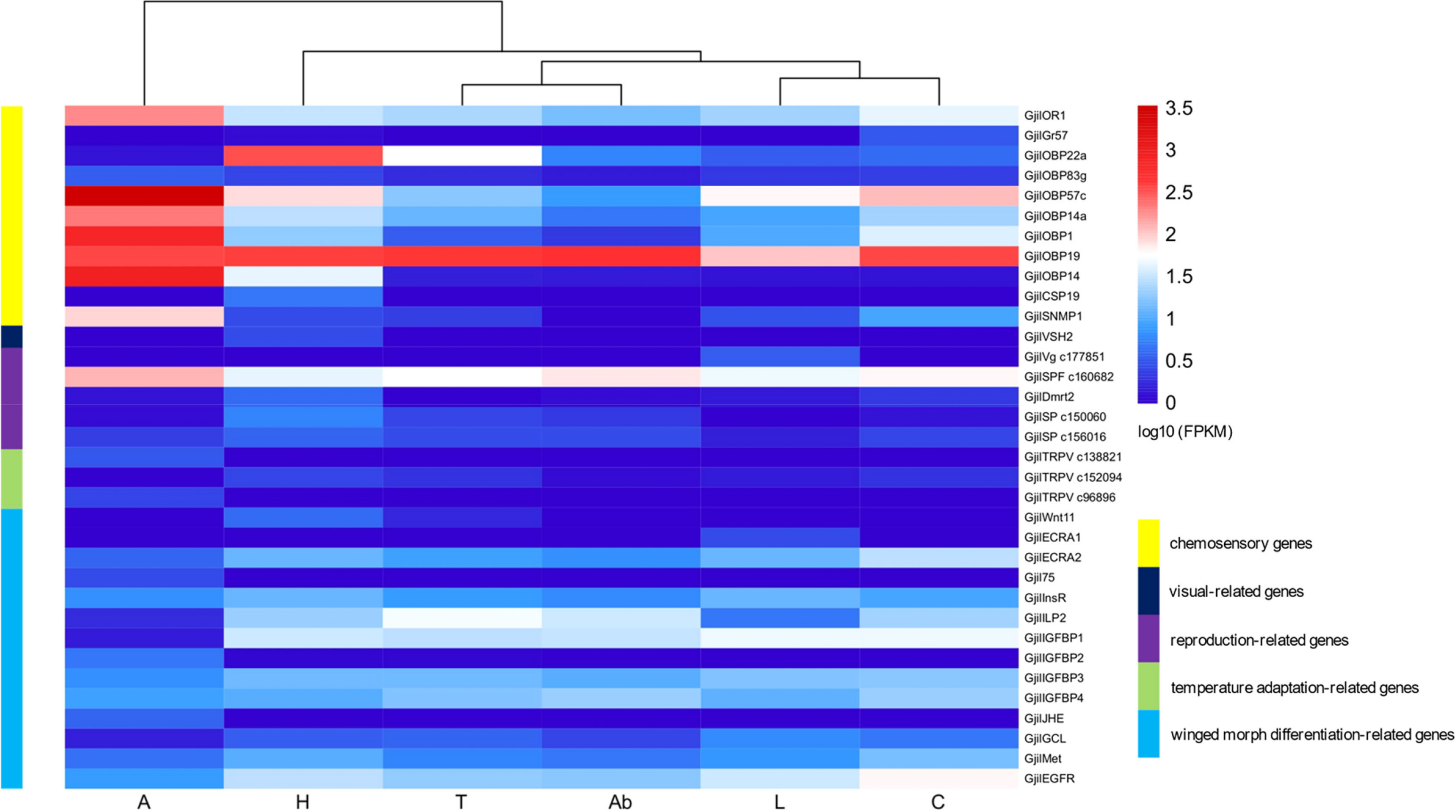
Expression patterns of genes related to environmental adaptation in *Grylloprimevala jilina*. Each row represents a gene, and each column represents a specimen. The specimens are (A) antenna, (H) heads, (T) thoraxes, (L) legs, (Ab) abdomens, and (C) tails. The color gradient from red to green represents log10 (FPKM) values from large to small.

Based on the FPKM transcriptome sequencing results of six tissue samples (antennae, heads, thoraxes, abdomens, legs, and tails), we found that ORs were mainly expressed in antennae, and *GjilORCO* exhibited the highest expression among the ORs. Among GRs, *GjilGR57* was expressed in the heads and tails. Most OBPs were expressed in all tissues, however, *GjilOBP56* and *GjilOBP84a* were expressed only in the heads. The genes *OBP1*, *OBP19*, *OBP14*, *OBP14a*, *OBP57c*, *OBP83g*, and *OBP22a* were expressed in the whole body of *G. jilina*. Among CSPs, *GjilCSP19* was expressed only in the heads. *GjilSNMP1* was mostly expressed in antennae but was not detected in the abdomens. Among the visual‐related genes, *GjilVSX2* was expressed only in the heads. Among the reproduction‐related genes, *GjilVg‐c177851* was expressed only in the legs. Among SPFs, *GjilSPF‐c160682* was expressed in the whole body. Among Dmrts, *GjilDmrt2* was mainly expressed in the heads and antennae. Among SPs, *GjilSP‐c150060* was mainly expressed in the heads, while the expression of *GjilSP‐c156016* was lowest in the head. Among TRPs, which are temperature adaptation‐related genes, *GjilTRPV‐c138821* and *GjilTRPV‐c96896* were only expressed in the antennae, while *GjilTRPV‐c152094* was expressed in all tissues except the antennae. Among the winged morph differentiation‐related genes, most Wnts were expressed in all tissues, while *GjilWnt11* was highly expressed only in the heads and thoraxes. Among EcRs, the expression of *GjilECRA2* was the lowest in the antennae and legs; *GjilECRA1* was highly expressed only in the legs, and *Gjil75* was highly expressed only in the antennae. *GjiInsR* was expressed in all tissues, *GjiILP* was expressed in all tissues except the antennae, most *GjiIGFBPs* were expressed in all tissues, and *GjilIGFBP1* was expressed in the antennae. *GjiJHE* was mainly expressed in the antennae, while *GjiGCL* and *GjiMet* were expressed in various tissues, including legs, tails, heads, and thoraxes. *GjiEGFR* was expressed in all tissues (Figure 4).

## 4 DISCUSSION

We found fewer chemosensory genes in *G. jilina*'s transcriptome than in other insects. It can be likely attributed to the lack of food but a stable cave environment (Croset et al., 2010; Niu et al., 2016; Robertson et al., 2003, 2018; Venthur & Zhou, 2018; Vieira & Rozas, 2011; Vogt et al., 2009). *G. jilina* does not need to retain the comprehensive chemical signal‐sensing ability, and less gene expression will reduce meaningless energy consumption (Wagner, 2005). Such a gene set suggests *G. jilina*'s adaptation to the barren and stable environment of the cave (Zhou et al., 2023). In addition, the BUSCO analysis reveals that many genes are missing from the assembly and this also might lead to our genes of interest not being detected. Moreover, in a study of a cave beetle, it was found that compared to polyphagous beetles inhabiting surface habitats, the detected diversity of the odourant and gustatory gene repertoires had decreased (Balart‐García et al., 2021). Our results are consistent with their findings.

ORs are important chemoreceptors involved in the recognition of chemical volatiles and pheromones (Wicher & Miazzi, 2021). The homologous evolutionary tree analysis showed that ORs from *G. jilina* formed intensive clustering regions and 6 of the ORs clustered into a single branch. This clade suggests ORs expansion, which might be related to *G. jilina*'s adaptation to the cave environment. Also, ORs of *G. jilina* were mainly expressed in the antennae, and *GjilORCO* exhibited the highest expression, which is consistent with other insects (Jones et al., 2005; Larsson et al., 2004). As key taste receptors (Katz et al., 2002), GRs can be divided into the bitterness family, sugar receptor family, CO_2_ family, and fructose taste receptor family of proteins (Miyamoto & Amrein, 2014; Xu et al., 2017). GjilGR57 clustered with *D. melanogaster* fructose receptor DmGr64a, indicating that it could be involved in fructose recognition. *GjilGR57* was expressed in the head and tail tissues, suggesting that these body parts participate in fructose recognition and other taste perception functions. GRs contracted in *G. jilina* and only two were founded, which would be interesting if it was linked to the scarce resources in the cave. IRs play an important role in olfactory recognition. In this study, two IRs, *IR8a* and *IR93a*, were identified in *G. jilina*. *IR8a* is related to the perception of lactic acid in *Aedes aegypti* (Raji et al., 2019), and *IR93a* is related to the preference for temperature and humidity in fruit flies (Enjin et al., 2016), suggesting similar roles of *IR8a* and *IR93a* in *G. jilina*. OBPs play an important role in binding and transporting liposoluble odors during chemical signal detection in insects (Ribeiro et al., 2014). Notably, CSPs have similar functions (Angeli, 1999; Li et al., 2021). PBPA1 and *B. mori* PBP clustered together and may sense pheromones (Lautenschlager et al., 2007). The genes *OBP1*, *OBP19*, *OBP14*, *OBP14a*, *OBP57c*, *OBP83g*, and *OBP22a* were expressed in the whole body of *G. jilina*, suggesting their role in other physiological functions of the body, such as the transport of nutrients, recognition of taste substances, dissolution of fatty acids extracted from food, etc. (Ishida et al., 2013). *GjilOBP19* was expressed in the whole body but the highest in the tails, while *GjilCSP19* was expressed only in the heads. Due to this particularity, these genes should be further studied. Benton et al. found that SNMP1 plays an important role in the recognition of sex pheromone in *D. melanogaster*, and the *GjilSNMP1* gene highly expressed in the antennae of *G. jilina* might also participate in the recognition of sex pheromone in males (Benton et al., 2007).

The visual‐related genes of *G. jilina* are notably fewer compared with other insects, including other cave‐dwelling species. Compared with some cave beetles, we did not find opsin genes in the transcriptome of *G. jilina* (Tierney et al., 2015). This could be due to two possibilities: first, the regressive evolution of visual capabilities in *G. jilina* might be more extensive; second, because some genes may not be expressed, the transcriptome might not have effectively detected all the genes. We found only the VSX gene in *G. jilina*. VSX gene participates in the regulation of proliferation and differentiation of visual cells and helps maintain the function of bipolar cells (Ohtoshi et al., 2004; Valleix et al., 2006). The compound eye of *G. jilina* completely degenerated, leaving only a dark red funnel‐shaped structure in place of the compound eye. The VSX gene from *G. jilina* did not cluster with the proximal species selected in this study and exhibited very low similarity with the ancestral genes of other species. Therefore, we speculate that *GjilVSX2*, which is highly expressed in the heads, may have other functions.

In total, 26 reproduction‐related genes (2 spires, 4 Vgs, 3 SPFs, 6 Tudors, 1 Dmrt, 3 SPs, 6 Soxes, and 1 vasa) were identified in *G. jilina*. The number of genes identified was similar to that of other species and the genes are relatively conserved. Vgs participate in egg maturation and embryo development of female insects (Santos et al., 2019). *GjilVg‐c177851* was only expressed in the legs and would be more interesting if it performed other physiological functions. Jh‐related genes and ECR‐related genes play a close role in Vg synthesis. The SPF gene mainly participates in the folding and quality control of glycoproteins in the endoplasmic reticulum (Ren et al., 2018). *GjilSPF‐c160682* was expressed in the whole body. We suspect that it assists in the endoplasmic processing of proteins in complete worm bodies. Among Dmrt genes, *GjilDmrt2* was mainly expressed in the heads and antenna. Dmrts not only participate in sex determination and differentiation of biological individuals but also regulate the transcription of other genes involved in organogenesis, embryonic development, and other biological processes (Kim, Kettlewell, et al., 2003). Based on its expression location, *GjilDmrt2* may be involved in the development of the heads and antennae. Among the SP genes, *GjilSP‐c150060* was mainly expressed in the heads, while *GjilSP‐c156016* was the least expressed in the heads. SP genes regulate individual sensitivity to high temperature, hunger, and stress (Hui et al., 2021). The differences in the expression of *GjilSP‐c150060* and *GjilSP‐c156016* suggested that these may have distinct functions in *G. jilina*. The Sox gene family participates in early embryonic development processes such as sex determination, bone tissue development, nervous system development, hematogenesis, lens development, etc. Sox genes are expressed in spatiotemporal‐specific manner (Wegner, 1999). It seems that Sox genes participate in the development of different tissues in *G. jilina*. In short, reproduction‐related genes were preserved in *G. jilina*.

Caves present unique abiotic challenges to animals and many of these stressors can be mediated by membrane composition something which Tret gene family is known to influence (Jain & Kumar, 2010). In this transcriptome study of *G. jilina*, Trets formed a unique cluster, indicating gene expansion. This may be a speciation event indicating the adaptation of *G. jilina* in cave environments under perennial low temperatures. Moreover, most Terts were expressed in all tissues of *G. jilina*, suggesting their role in stress resistance through all tissues. TRP plays an important role in temperature sensing, immunity, sensory conduction, and other aspects. These genes improve the body's maximum voluntary contractionary force and strength endurance and antistress ability, increasing the survival probability of the organism (Diver et al., 2022; Wei et al., 2015). TRPs formed a unique cluster, indicating expansion of these genes in *G. jilina* can be related to adaptation to the cave environment. TRPV participates in hearing perception in insects (Kim, Chung, et al., 2003; Kim, Kettlewell, et al., 2003; O ‘Neil & Heller, 2005). *GjilTRPV‐c138821* and *GjilTRPV‐c96896* were expressed only in the antennae, whereas *GjilTRPV‐c152094* was expressed in all tissues except the antennae. Consistent with the expression location of GjilTRPVs, we found that there were more mechanical sensors on the antennae of *G. jilina* than in other parts (data not published yet).

In total, we identified 22 winged morph differentiation‐related genes (6 Wnts, 2 EcRs, 1 E75, 1 InsR, 4 IGFBPs, 1 ILP, 1 JHE, 3 JHBPs, 1 GCL, 1 Met, and 1 EGFR) in *G. jilina*. The number of these genes is similar to those in other species. However, *G. jilina* has no wings, which seems to be contrary to the fact that there are still more wing‐related genes expressed in this cave insect. Therefore, we speculate that these genes in *G. jilina* may perform other functions, such as growth and development or these genes have not yet fully diminished during the cave evolution of *G. jilina*. The tissue expression analysis of Wnt genes showed that most Wnts were expressed in all tissues, meanwhile, *GjilWnt11* was highly expressed only in the heads and thoraxes. Notably, the Wnt signaling pathway is involved in the regulation of nerve induction, and the regulatory effects are different in different developmental stages (Wilson et al., 2001). Although *G. jilina* has no wings, its Wnt genes may still participate in neural induction. Among the EcRs, *GjilECRA2* expression was low in the antennae and legs, *GjilECRA1* was highly expressed only in the legs, and *Gjil75* was highly expressed only in the antennae. Hentze et al. (2013) showed that EcRs play important physiological regulatory roles in growth, metamorphosis, molting, reproduction, and innate immunity response in insects. Though *G. jilina* has no wings, its EcR genes may be involved in the development of antennae at different times. InsR was expressed in all tissues of *G. jilina*, and ILP was expressed in all tissues except for a low expression in the antennae. Most IGFBPs were expressed in all parts of the body, and *GjilIGFBP1* was expressed in the antennae. InsR genes play important regulatory roles in cell proliferation, differentiation, development, reproduction, and longevity in different developmental stages of insects (Colombani et al., 2005; Hafen, 2004). Notably, the overexpression of InR, Chico, and AKT led to the overgrowth of tissue cells in *D. melanogaster* (Verdu et al., 1999; Weinkove et al., 1999). InRs NlInR1 and NlInR2 regulate the activity of NIFOXO and control the development of short‐ and long‐wing types in *Nilaparvata lugens* (Xu et al., 2015). Therefore, the absence of wings on *G. jilina*'s body surface may imply that InsRs do not regulate winged morph differentiation in *G. jilina* but have other functions. JHE was mainly expressed in the antennae, whereas GCL, Met, and EGFR were expressed in various tissues. EcR initiates and controls insect molting and metamorphosis, whereas JHE inhibits EcR to regulate the molting process (Dubrovsky, 2005). GCL participates in the determination of individual sex, Met regulates growth, development, and reproduction, and EGFR regulates cell proliferation, differentiation, and migration, affecting organ development and wound repair. EGFRs were shown to regulate the development of the nervous system, epidermis, and organ formation in *D. melanogaster* (Weisman, 2005). EGFR is highly expressed in the nervous system and participates in the development of the central nervous system and the formation of neural precursors. In *D. melanogaster*, EGFR regulates the formation of native receptor organs by recruiting ectodermal cells, facilitating the sensing of the body's position and spatial motion via transmitting information to the central nervous system (Inbal et al., 2004). EGFR also regulates legs development (Grossmann & Prpic, 2012) and embryonic organ formation in insects (Olivares‐Castiñeira & Llimargas, 2017). Therefore, it would be interesting if JHE, GCL, Met, and EGFR genes do not regulate winged morph differentiation in *G. jilina* may have other functions.

Compared with the outside environment, the cave environment has a low temperature, high humidity, no light, poor food, high CO_2_ content, and different soil composition (Fernandes et al., 2016). Therefore, to adapt to the cave environment, true cave animals undergo visual degradation, nonvisual receptor evolution, pigment degradation, cold specialization, low metabolic rate, life cycle evolution toward K‐selection characteristics, appendage specialization, sensory organ specialization, and large and few eggs production (Lavoie et al., 2007; Strecker et al., 2004; Wilkens, 2001; Wilkens et al., 2000). This highly selective environment can easily induce genetic changes in species. Previous transcriptome studies have studied the specific evolution of chemosensory genes in the underground beetle pedigree (Balart‐García et al., 2022), and the loss of gene expression related to vision in cave animals (Stern & Crandall, 2018). Our findings in *G. jilina* are similar to their results. Transcriptomic studies can provide important information about the genomic composition and genetic diversity of a species, including aspects related to adaptive genes. However, there are fewer transcriptomic studies on cave biota, which need more attention from researchers. Concisely, ours is an in‐depth study of the genes related to environmental adaptability and their tissue‐specific expression in *G. jilina*. It is possible that not all gene families were identified, and there may be some new gene families that need to be investigated in the future to interpret the function of all genes. The identification of adaptive genes can provide insights for future studies on the correlation between environmental conditions and corresponding adaptive genes, allowing the scrutiny of environmental changes in endangered insects. This will also help us understand the evolution of cave insects.

## AUTHOR CONTRIBUTIONS

**Yuxin Zhou:** Conceptualization (supporting); formal analysis (lead); investigation (supporting); methodology (lead); writing – original draft (lead); writing – review and editing (supporting). **Lin Zhou:** Conceptualization (supporting); investigation (lead); methodology (supporting); writing – original draft (supporting); writing – review and editing (supporting). **Qiuyao Li:** Formal analysis (supporting); investigation (supporting); validation (supporting); writing – original draft (supporting). **Xiaoyan Zhu:** Formal analysis (supporting); investigation (supporting); validation (supporting); writing – review and editing (supporting). **Zhongbo Yu:** Formal analysis (supporting); investigation (supporting); supervision (supporting). **Haoqin Ke:** Investigation (supporting). **Qi Chen:** Conceptualization (equal); project administration (equal); validation (supporting); writing – review and editing (equal). **Bingzhong Ren:** Conceptualization (lead); project administration (equal); supervision (lead); validation (supporting); writing – review and editing (lead).

## FUNDING INFORMATION

This study was funded by the National Natural Science Foundation of China (31172133), the Young Science and Technology Talent Support Project of Jilin Province (QT202121), and Fundamental Research Funds for the Central Universities (135111010). English‐language editing was provided by the editing service.

## CONFLICT OF INTEREST STATEMENT

The authors have no relevant financial or nonfinancial interests to disclose.

## Supporting information


Appendix S1

Appendix S2

## Data Availability

Raw reads have been deposited in the National Center for Biotechnology Information (NCBI; BioProject accession number PRJNA951510, https://dataview.ncbi.nlm.nih.gov/object/PRJNA951510?reviewer=an4jiceir7rv7d39nbrbeikalm).
