# Supplementary material for: Transcriptome analysis and identification of genes related to environmental adaptation of Grylloprimevala jilina Zhou & Ren 2023
Source: Ecol Evol. 2023 Nov 20;13(11):e10717. doi: 10.1002/ece3.10717 (PMC10659822; doi:10.1002/ece3.10717)
Supplement: Supplementary file 1 — Appendix S1 [file ECE3-13-e10717-s002.docx]

# Supplementary Data S1. ORs

>GjilOR1

MMKFRNHGLVADLWPLIRSMQLTGHYLFEYHEENGFMWRTLRLGYSIVQT

VVILLQFLFMFGNLYIEADDVNDLAANTITVLFFTHCVTKLLYFAITSKK

FYRTLNVWNTVNSHPLFSESNARQHALSVAKMRKLMVMVATVTVFSVLAW

TTVTFLGDSVKEVPDPEVENGTMTIDVPRLMIKAWYPWDANQSGPYIMSF

VYQFYWLFITMAHSNLLDVMFCCWLIYTCEQLVHLKDIMKPLMELSAALD

TLVPHSTELFRAASGGSNHPSIGESMDVNIRGIYSNQRDFSGFRGQGALS

TLQTGNVGPNGLTKKQEMLVRSAIKYWVERHKHVVRFVGSIGDSYGAALL

LHMLTSTVTLTLLAYQATKIETVDVYASTVLGYLFYTLGQVFLFCFYGNR

LIEESSSVMEAAYSSHWYDGSEEAKTFIQIVCQQCQKAMSISGAKFFTVS

LDLFASVLGAVVTYFMVLVQLN

>GjilOR23d

METKVKPQPYFTWNIISLKILGMWVYDKASTQPIMYYLYFAYQAFCWFFW

STFIISLAIITVIEVNNLHKIADSVSISLCNIIIIAKVYAAMKQRKLMDK

LMNEMGSLFDSLDDAMCPGKHILVSKALKYSRIITLGCCMLFGFTAVGWV

SSTAVQNKIRSKAHAIYSPNETFIPMLMMPAWYPFEINTSPNYEIIIIHQ

FFGAFFDATSDAALDVFFCTFIIYAHCNFKILNVALKNLTLRFIQGKATQ

AAEGPKKEKQVTAGSESPPEFDAGDPEESWINADHKDETLLQANQEMERR

ITECIKYHQSLLKYSKELGKSLSWMFFIQVLMTSITLCFGGFLVITEPEL

NIKVFFVSSYIVCILVELGLFSWFGNELTLESRNLRESIYESDWYKSSSK

FKFCVGMIIMRTQKPIKLSAGKFGSLSLNMFTALLRTSYSYLAILRQMNE

EQ

>GjilOR23a

MMDIEICKDKELISKKVFRYCHRFTLGIFIWLFTGGLGCTFTVVIYNEIS

YSYYDGSHNETFQFKQVLNCWCPFDYQNRPYYMIMMTHQIVSIVIIVYVF

SILFTLYITFFVYSTAHFKILNSALTNLTADSIGCSIQGLERATDKVNLS

EKKFRGFMESQIGSHDLTTITDEDPVDIFHLKMERRIVECVKYHQAMLDC

SKHTAAVLRSIFFVLYLTTGISLCLAGFQVLVVPPNSSEFLIMVIYVVTI

VCEVGLFCWYGNQLTLESQNIGDSAYNSDWYDSSLRFKLCVLLIIMRSSK

PVAIDAGIFGDLSLEMFTGLMGTSYSYLNVLRQMKEGKTDADI

>GjilOR23c

MTHAQIFSYVVVLSYNFSVVMWIVVAVAQNEMNFFKANSSSNETYAIKQI

YKAWYPFEYSEKPLYDLMMFHQISAALIIGLQMSSLDAFYCSFMIYATCQ

FKILNTALRNLTVQAMLGAGIPDPDLPGPSRKAPSALQSGLDKLKLLESA

SPDPTAEEVSDASVDVRLEMDRRITECIKYHQAIIICSDGMGETFRDMLF

VMYLCTALAVCLAGFQALVYPAQNTAEFLMKLVYLACLLLQIATYCWYSN

ELTLASAEVGDAAYASDWYSGSTQFKFCVAMLIMRTTRPVYIRAGMFGNL

SLQMFADLMSSSYSYLAILRQMHEEEEEGE

>GjilOR39b

MWWPFDSIETPNYELLYLYQILIAFTLCAINMASDNVYISLIIHASARFK

VIYSMFINIIEEAHKNSNDPNLLTNQGSIDEGSGSLPGQTRFQSYDFALR

QPPILAVHIQQGSNIVPKGEEITSSRDISFEMNQILTDCIKQHQSVIRFV

QDLNTVMSVGMLLQFMVSTSIICLSGFQATMTTGETETFVKFIMYLATAS

LELLLFCWFSEDLAAQSELVHEAAYCCGWHDSSQSFKSCIGLAIMRAQKP

LRLTAGKFYSVSLETFVSLLNTSYSFFVVLRQMDTL

>GjilOR67d

MAFDVFFLALVGLGVAQYDHLRNIFERVFEPLKGEHPLVLDNCENDSSID

DGKSLSEEQTEEELTRVLKPRLDLWIQHHQDAIKFGNELLELYSPLLVIQ

FLTTTLELCFHIFSSAANDSGSVLGSGMFVVAYVLQIFLLCRYGDRLAKS

SSVLGEAGFASLWYLSRKEIRSSLKVIFQRCQVPLQISAMGVVPLNASSF

ITIVRAAVSYYMILTEMQERGK

>GjilOR23e

MDRLLRIYVTTHQDLVKHAEELNALSSPMLYAQHIVVSVNLCLIGLMVLM

ESTDSTIMISYLLCLLCRLGIFCYYGAQLTALSSDIRVSAYGTDWYRIQS

KSFKCGVAMMIMRAGKPATITAGKFGALSLEMFAGMIQASYSYFAILRQV

YE

>GjilOR39a

MWMCMPLLTSLVGGPDDPFASNETDKAMKHFPFELYLPFDSTVSPNYQLV

YCYQLVDLVIICNCFCSADSLFVVLIAHATGLLKVLVIHLSTIEQNAMKN

CSVSQRNEFGSQNLYHKDEPQDYLNVLTGTEISVRGETVRRENTENSWRN

TQGVPFSSLKSEVTPAEESNYELTAMSQVRFDEEMNRSLIKGLKLHQAII

RYVRELENIIGPVMLFQFFITTFTICFSLFETAQSADLTNPGIMKFLCLT

FNVTFELLLFCKYGEDIINESETVYQGFYNCGWESGNSSFRRSCGIAMMR

AQRAITLCTAGRFYTMSLETFRKIVNASYSYFTLLRTLPE

>GjilOR23b

MIIMRSQKPVALSAGIFGDYSLELFSGLMGTTYSYLTVLRQIHDKQ

>GjilOR25

MIFFGIICNSAHLASQQVGSIFKTDLQSIAKRLMYTNNISAKREVQAFLH

VIDLNPPNINLGGFGDVNRELELSLLSSMVTYLVVLLQVQLSLTTPTQDC

SSNSTS

>GjilOR43a

MLIRAQVPLIFRGGPFYACTVQEWVAIMNTVMSQFMVLRSIHSSE

>GjilOR23f

MYACGQFELLNESLNEITIDNVKSKIMKTSKKIAHDIVESQGANKLHALS

LLNLQKFQDENTEWQVSEPGKGNDELPGQISYNKGFSNVTQDMLFNIQED

IDRDIGTNGELSPATIDAEMERLLGRSVKTHQELLLHSAEVQTLCNRMLF

SQYVVVSLNLCLIGYQILMESESDSVGIDNITLFAYLMCLLVRLGIFCYY

GTQLTVLSSETRDSAYKTDWYQTESKVVQSGVEMIIMRAGKPATITAGYF

GALSLEMFTGLIQTSYSYFAILRQMNE

>BgerORCo

MYKFRLHGLVADMWPLIRMMQMTGFFLLDYYEDMSFGFTSMRAGFSGTVGVLMVAQFG

CLFLNLMHQADEVNDLTANTITVLFFVHSLTKFFYFAIRRAKFYRTLATWNNANSHPLFS

ENNSRHHQASVGSIRKLVLYVSIGVILSVVSWTSITFVGESVREITDPDNTNETMKVEIPRL

MVRAWYPWDAMGGAGYFISFTYQFIWLILALSHSMLTDVMFCCWLIYTCEQLLHLKEIM

KPLMELSASLDTVVPYSAELFRAVSATGNQPLPGTDGDGIRSIYSNQHDFSQFRLNTGTIA

NVKQGGVGPNGLTKKQELLVRSAIKYWVERHKHVVRFVSNIGDTYGAALLLHMLTSTVTL

TLLAYQATKIDQVNVYAASVIGYLFFTLAQVFLFCFFGNRLIEESSSVMEAAYSCQWYDGSE

EAKTFIQIVCQQCQKAMSISGAKFFTVSLDLFASVLGAVVTYFMVLVQLN

>BgerOR1

MTTQTGEVLDLLSKLHSFAPLLRLLNLSGMLPPLSIYKVPWKNFLYNIFSLLTFLSFIPLIIM

QLTGFYVYGNKLNLILVIAFQLAAYFDGMVTMAYFVWNRKRLLTMFLLMETKFIPLMSKV

GRPEKQKTILAKNWRFCKVITTVAIGAFLADMATWCVLPSVVRYMAYLHEEEEVEGEPE

HKMEYFVLVMWLPQNATAFPRYEMLHVFQFFSVWGVVANYAAGKLIIVTLFYHLATHFN

ILSSAIQDIDDICVNELSANEDMRLVVKQLAISHETNDSSVIEGALNLNLRSKIVQEKRLCPV

DLDPDNINVKESDIFGRLTYPDEVTYLTDCVIYHQELLKFCREINSFLTNILLFDLVIFQILM

CLPPLQLVLGQDASTFRFVSSIVDTGIWPLLICFWGEAVSQESLGVRKAAYDCQWYNRSQA

VKRLLQMVLMRTREPVRMTAGQFYNLSLETFADIENKVYAYFTLLKNMQG

>BgerOR2

MENRGESTLEKLNLLKKQYQFLHVSGILPYSSVCKSFWKTSLYNSFTILCMFYLPFTIVTFL

LAIEQFGDDLDIFVSILYEVFLFMYIVFELYYWILNRKYLLKLYNDLESKFIPHITKLNLSPD

CIQDSINFYNKLMIIIVIICVADIFAWAGVPFLLWCFETSEDYKNMEGNEFWKYFSNICWLP

ANATQTPMYQVVYSHQAFSSYLCGIHLTSCNVVMFSVIYHVATHFKLIIAALEKVDSRFLV

KESKQEERYLSKKDIETNEDQSKNDKRANEDLSVTPENFKNQRSSVIDEEKIYQYLIDCVK

YHQDLLEFCKEANTLMSPMLLATLVYIQISVCVPLLLVALGRYNGFAKYIVAVVEQFVMPC

MICYYGDYVIEQSLNVQKAAYGCIWYHRSPEVKKLLQIIIMRAQKPVQFTANSFYVVSLETL

GDIFNKVYAFFTVLKGMFE

>BgerOR3

MIGHKKQNLLNRNLIALYISGFLCPPNWQKNLCKKILYQIFTLETIAVFPAIITVQTIELFHR

FDDLFATTAILFQLACFISVYINFLYFLYYKPELIKLMDRIEIDFVPLMERVGSSKRRETILAE

RHEKSSSITILMISMHIFVTTAWGILPWVLSYIDYFIKTEEELAEVEIRNYFGCLMWLPENV

MKSPKFEVMQLFHFWGIYGIVSNITSCYMIMFMLTFHTATIFRLICAAFEDIDEFERSLRNE

ENFKKNSCVSDGKCVFVEIPSDEGFNENSNEEHDNISRNRKQENAQPFINDANGSNVSNP

QVNEEIRRRMNEYLINCIEFHQAAITYIEDLNDLVSPMVFIFFIFTEVMLCLSSFQLALAKW

DEKKIKFLSSVSCVFTWPLLLCIYGDDLKSSAVKESAFSIRWYCQSKTFNKLLQMVMIRAQ

QPVCIRAGKFYVATLETFSDLCHKVYAYLTLLRQMYDNS

>BgerOR4P

MTGKKRQHFLNRNLVALYISGLLCPPNWKNNWYKKILYKIFTLGTVVVIAAIIVVQAVELF

HRFDDLFATTAVLFQXTCSTSVYINFLYFLYHKSELMKLMDNIEIEFVHHMERVGSPGRIK

TILSERYEKSSSISILMISMHAFVTTAWAVLPWILSYIDYFIKTEKELRELEIERYFGCIMWF

PENVMKSPKFEFVQLFHFCGIYGIVSNITSCYMIMFMLTYHAATIFRLICASFQDIDEFERSL

RSEEHSKLNSFISDSVKMPLNKLRELESLDENSNDHDYVSRNRKQENVQPSISDVYVSTVS

NPHTNEEIRRRMNEYLINCVKFHQAAIKYIEDLNDLVSPMVFIFFIATEVMLCLSAFQFALA

KWDEKKIKFLSSVACVFTWPFLLCIYGDDLRSSAVRQSAFGIRWYCQSKTFKKLLQMVLIR

AQQPVCIRAGKFYVATLETFADLCHKVYAYFTLLRQMYDDK

>BgerOR5

MEPKKSTEEKWKGAKGVTANKLELMNFQLKLLSWFALLPPMNAKRDSWTYRFHLLFCC

TLLFWYIPMLIADTMAIPQNWGNLPLVIEVIFQISASISAMIGGYYITYHKYRVVEHFKMLE

TRFMGFINNSTVSEEHLSNTLAKVTKQAKLSSYLLILNVGTILLSWTGLPYIRMSKARASEN

ILDQSSPDFWGYFCFVMWLPENPIESPKYEFLYLFQLPCVAVVIFHITGLNMIFYFTILYISF

YFELLTKSLQDIDKRFPLESEHGPQIIENKLILNEEEDASNQQYIQTHDLSVVKNEDSYSRK

NLETSYLFNNVNQNETPEEYVANFGDKSPEFLSLEENAIEHLKQCIKYHQSLLEYHKLAND

LLSPMFLAFFMSNEISMCLSVFQILVNENSGILKTLSSGLTVCTWPFIISYSGEYLTNKGKEF

ESVIYDMQWYKRSEKFKKLLLMNLAGAQKPVRLSAGNFFDVSLESFAEIVNKVYAYFTIVK

KMYDG

>BgerOR6

MLSKEPNNGKCNDNKENNRKKLDLVSLQLKILTYFAILPPANAKRDSWISRFYLAFCYLLL

FWYIPMFAFDMMAIPQNPGNLPLITEVIFEMSASTTAGIASSYFIFNRHRVTEIFEMLETRF

EKFINNKTVSETNFKCIFMKVSKIAKIVTYTIAINVCLLVAAWICLPYTRRIMDETSGNVHD

ESKPEFWGYFCYIMWIPEKPLESPTYECLYLFQISCVLMVICHYTAMNTIFFFIIIYTLAYFQ

LLTTCITDIDKRFPIENGSKCDSEINRRLFRNLHGHVPKESHYKTKSDSCRDDALVLTKDT

NDGYLYDLHNDKLTAVNEELANGLEDDAVEHLKQCIEFHQSLLEYYKSVNSFLSPIYLAFF

LSNEISMCVSVFQLLVNENNGIMKIIIGAINACSWPLIISWCGDYLTEKSKELESAIYEMQW

YKRSERFKKLLFINLLGAQKPVRMTAGKFFDVSLQSFAEIVNKVYAYFTILKKMYDT

>BgerOR7a

MDFLKHQLFILYLSGLICPKLWVQNWCKKILYNMFTLAIVIVFPLILSVLMVTLMQQLDDL

STVIAIIYNMLCFISVYINFIYVLRNKSNLLNLIERIETEFIFYMERVGSPQRRETIILKRSKIA

KNITYLMLSMYLFVMFFWTIFPWFLGYKDYFMNTDEQINTDMQNYFGMPILLPENINKF

PIYEIVQIFHFVSAYSIVTNITACYMVIFILTYHAATLFKLIYAAFEYFDHFERNLRREYELED

DVISDCDSLKSDGSKTYDFHQSKTEERSANYGGNTNQDTANKSISINENELEFSINNSNTST

IQDPKNNEEYQRRLYKYLVDCVEFHQAAIRYTDDLNDLVSPMIFIFFIFTELMLCLSAFQLV

LAKMDERKLKFLTSSSTLFAWPLILCHYGDDLKSSAVKESVFSIRWYTQSKSINKLLQMVM

IRAQQPVTIRPGKFYVATLETFSDICHKVYAYLTLLRQMYDN

>BgerOR7b

MAEIKKNNFLEAPVFFLHLSGLLYPPNWKQNWYKKIFYDVFTLAIAVFIPLMEIVLLMHFI

ANLDDLFRATAVLFQILCLMSVHIIFFYFLRHKPKLLKLIDRIETEFDFYMERVGSPERREAI

LSERYENLKKIVNGMWLVYLFSMLFWTIFPWLLCYIHYFIKTEELSKEDTEKYFGLEMWL

PENINKFPIYEMFQMFNLMTVYCNVTNMSACYLIMFVLTYHTATLFRLICSAFEDFDYFES

TLRSEYEIYDTKNQSNSFKSDETERRSPARERSGHHDGYVDSFNQDSVGTNKSIDRNKFKV

IDTVTDVSNISNLEDNEEIKTRLNNYLIHCVKFHQAAIRYTDDLNDLVSPMIFIFFIFTELML

CLSAFQLVLAKMDERKLKFLTSSSTLFAWPLILCHYGDDLKSSAVKESVFSIRWYTQSKSIN

KLLQMVMIRAQQPVTIRPGKFYVATLETFSDICHKVYAYLTLLRQMYDN

>BgerOR7cP

MAELKRNNFLKVPLFFLHLSGHLYPPNWEQSWYXKDIVRYIYTLAMAIWSPSISVVLMVY

LIVNMDDLFRATAVLFQILIIISVHINFLIFLRHKSKLLKLIDRIETELFFYMERVGSRERIETI

LSERYEKLDKIVYCMWLVYLFAMLFWTIFPWFLRYIQXFIVTEELSKDTTQKYFGMLMWL

PENINTSPTYELFQIFNFMTLYGSVSNMSASYMIMFVLIYHTATLFRLICSAFEDFDYFESTL

RSEYELEDNTKSQFHSIESNQLKIYNILPLSAKKQSKHCDGLSGNTNQDHTVAFKTRHENK

FKLFINDTNVSINLNFENNEEFKERLNKYLVHCVEFHQATIRYTDDLNDLVSPMIFIFFIFT

ELMLCLSAFQLVLAKMDERKLKFLTSSSTLFAWPLILCHYGDDLKSSAVKESVFSIRWYTQ

SKSINKLLQMVMIRAQQPVTIRPGKFYVATLETFSDICHKVYAYLTLLRQMYDN

>BgerOR8P

MKRKNLFKRQLFVLHLSGILCPPNWEQNWFKKTLYYTYTLGVAICVPSMFTVLMMYLIN

HIDDLFKVTAVLFPNLCFITVYINFIYFMRNKSKLLNLIDRMETEFLVHMERVGSPERREVI

LSRYQNSKKITYLMLSIYVFAMLFWTIFPWSLGYLDYFIATEEISKADAEKYFGLPILLPENI

NKFPIFEIVEVCNLMSVSCNVTLMTACYMIMFILTYHAATLFRLIRAAFEDFDYFENKLRN

EYELGDDTKSQCHSIRSNHSKMHNFFGSPGKERSEHCDDGNTNQEHTDAFRSTKENKFK

SSINNTNVSIEFNPKNNEEFQNRLNKYLVHCVEFHQAVIRYTDDLNDLLSPMIFIFFIFTEL

MLCLSAFQLVLAKMDEKKLKFLTSSSTLFTWPLILCNYGDDLKSSAVKESVFSIRWYAQSK

SINKLLQMVMIRAQQPVTIRAGKFYVATLETFSDICHKVYAYLTLLRQMYD

>BgerOR9

MEGTKRLRLLHLQLIALYLSGILYPPNWEQNRCKKFIYDIFTFITIIWFPPTIFLQMIFLLDC

LGDLRTATGILFQVSCYISTYIIFLYFVWHRKALVKLIDRIETEFVFHLERVGTPSRRDAILA

EDYRKQKIITCLMLGLCFLVEAAWGVIPGMIGYIEYFIKTEEELANNEKGKYFGLAIWLPD

NVNKSPTYEMVHCFHFIAVYTVVSNITACYMTMFMFAYHTTTIFKLTCAAFEDADDFENT

LLVENESNHDIRLMVGSSSSKETMRSYLNSMAANRWNEHSDRMLDKKSKEDNNHGFTS

NELQTSNFNSTVSTGDDVDVSHQKMFNEDLKRSMQKYLENCISFHQAIIQYTSDLNELLSP

VMLVFFVFSEAMMCLSTFQLALGKMDEKKFKFLWSVLLASVWPLLVCLYGDDLMSIAVK

QSVFNTRWFCQTTAYNKLVLMVMMRAELPICLRAGKFYIATLQTFADIGHKVYAYFTLLR

QMYDD

>BgerOR10

MEPQTNKREVFGLNDTLIEYDEETQMKRLKLLGIQRRLLYTCTGLPTFSTLQSKFKMILFH

LVSNVLVFWFIPHTITQFMALYQHMNDIELLADLVFQIALYIQTGTMAFFFRFTRKELSNY

FELLETQFVPNLKELGLSIEHKQMVKNHIRYGNFMIRILFGHWCWILFSWAILPAFTGYYD

MYTQFDQFRNESLTLTNASITITDEYRKYFGILIWLPNNIDQFPVFELFYLFDFVGTYGVSS

WCASQVSVSLVLMHNLSLHFKLLIASIDNLDIVILKTLDMQKVSHTTISESHDKSAVDSQFQ

PDLAANQERSNRIQNLTKATSKVSTAKNNLIISSSRDQQLHLSREHEEIVFNYLVQCIKYHQ

ALLGFCEDINKYLGPVLLIFFLGYEAMVIFSAVRLAMGLSEGNFKFFSSVVYVIFWPLVICSV

GQDLTNQSENVQFAAYNSRWYNHSTRIKKQFQMMIMRAQKPVKISVGKFYSVSLETYSD

MGNKVYAIFTLLKRMMAE

>BgerOR11

MELQTTEREIFRSNDALLEYDEETQKTRLKLLGNQRRLLYACTGLPTISTLNSKFKSNLYH

FVSNVLVFWFIPHTITQFMALYQHMNDIELLADLVFQLALYIQTGTMAFFFRFTRKELSNY

FELLETQFVPNLKEIGLSIEHKQMVKNHIRQGNFIIRILFGHWCWILFSWAILPAFTRYYEL

YTQFDQFRNESLTMTNASTAITDEYRKYFGILIWLPNNIDQFPVFELFYLFDFVGTYGVSS

WCSGQVSIYLVLMHNISMHFKLLIASIDNLDIVILKTLDIHYDPHTTISEFKGESVEDSKFRS

DHSASQAGSNRIQNVTKDTTEVPTPKENLTISSSSKQQLDISREHEEMAFNYLVQCIKYHQ

ALLEFCEDVNQYLGPILLLFLLGFEGMVIFSAVRLAMGLSEGNFKFFASVIYVVFWPLVICIV

GQDLTNQSENVQFAAYNSRWYNHSTRIKKQFQMMIMRAQKPVKIPVGKFYSVSLETYSD

MGNKVYAIFTLLKQLMAE

>BgerOR12

MEDQPSSAILTISENLIRFDPKTHIKRMNLLGIQPQLLYVCGILTPNWFSKSTFRSVIFKFYS

NLLLLWFLPHITSQIMALYQHMNRFATLTDLIFQLALFIHTVIITFYFVTYKSQLAKILDLLE

SQFTPYFERICILNIHKPLIEDVTKQGTIVTKALLGVLSSCLLSWGVFPAMVRYYDLYFTTD

QEKDESLNITEEYIKYFGLVVWLPPNVNEFPVYELVYIFDFIGTVGVACNCSGVQSIFLVIMF

TISTHFKIIIASIQNIPIKFPEILKQKKYVFENNGPNTNLNILSMNVIALHPNRPDTMIDSDTE

YNNMKSNSDIIHSEDLLNPELEERVYKYLVNLTKYHQILLEFCKDVNDFLSPVLLVFVLGCE

AMVCFAAFRLALELNEGNFKFFTSAVSMVIWLLIICWYGEHLIEQSIKVQVNAYDYNWIN

QSTRIKKLLQMIIMRAQNPVKLTAGKFYPVSLQTFAEIGNKVYAFYTLLKQMLADSEITG

>BgerOR13P

MQLSSKKKNQEYKIVDFSTYERLKTFYKIQLIILHYTGIFPRPEINRNEMIRKFYNTCTALVI

ICSLTSPVAMCTFLYQNWGLITIATGVIFQTTFYTVNASVYAYMALNRQRLWKVISSSEVD

SGMWNSFPKGTSIILQSRTNRWKFXEAFNLNKVLTWSIVIANSAGFFSWIILPITLWFVQG

EETDNPEETGYNTGQWKYFIYMMWLPENAVEYPTYYYIYLYQTYICVMLITVYTAYNIMF

SSLVIDIISKFDSLMMLLKNIDNMNLLEEDNIDSKICKNTDMPNRIANDILNHKEEFKVTFR

RENVETQREISIKDIDSDTSINITEEEKKYYYLVNCIKYHQHILRQAELMNKMFSPVLFIVFT

LGLIMMCTTVFQITLGSGQGTSLKIASAMFATWFPISLICYFGDKLTQKSLKVQEIALSTRW

YDRSPRYKKLFHVLIMRTQKPIQLTGGKFFIASLETFSD

>BgerOR14

MKTFYDIQLKMLHFTGILPLTDIYDITWKRVCYNIYTIIVIMLEIPAPFAQFVTIYQNWGVL

EITTGVIFQVCFYFNNASIYMYLLFNRNRLRHATCSVARKFVEQFGLKELDIYDTIRKKAFA

FNKVLLWVILLINSVTYFFWLIMPFTLWCTEYQNLNDVEEMNGYNTGQWRYFIYMTWV

PQNALEYPIYNFIYLYQWFALSVILVLYYGYNLMLFSQIIDISSQFNALLVFLKEIDKMTLCM

DNVLLQDDESNSKSTIFNKVSNMATTTHLAAANMNLKNIEEFTNSHIKERFDIMDDLYM

DNIFTEPIIDNLNKKEKELYSYLINCIKFHQYILKRVEEINKVYSPILFVVFTIGMVMMCTTV

FQVTVGSGQGTNMKLLTATITVWFPISLICYFGDKITQKSLEVQKVILSGQWYEKSIRFQKL

MLILIMRAQKPVQLTGGKFFTASLETFSQVANKVYAYYTVLKQMKDSE

>BgerOR15

MFCWKRKSENNNKQDDDETSAFLTVQLTFLKFGGILPSKSIFNSPLKIKIYNFFATISMLW

YIPIVVAQGVAAYQHWGNVSFVTGLLFESAAVLNNLMISSYLMMNRHKISSIICKVNSAFK

NQTEQLPFEEKHQQSLSDASNKNLKFAWILIITNILSSTLWVLVPFLLWYTYDGDDIYEHD

ESQEKEIHWEFFSQKSWLPSNVYETPYYQIIWFYQTVPVYSILINFTGYNMFFYSVTTFAST

HFQILADLLRNANKYIESTTLIQSSLEHQALTRGNLNTENEHIQVSDVHELNISDENMHGK

MEIKSEFNYSINKNERKETYQDVAYLVKCLKYHQALLEFCADVQNLFGPILFIFFCMNGIM

MCITVFQATLPSEEEGFIKFSMASLSCWFPIFLLCWYGDHLTIQSLEIEERAYGCLWYNSST

TFKKILQFLIKRSQEPVQLNGGGVFPISLKGFADIANNVYAYYTILKKMQQA

>BgerOR16I

MLSRKVVSEDNQKHEKRNKSNFLTMQLTFLKIGGILPPETIFNSPWKLKLYNTYALTIML

WYLPIMSSQFFAVYQNWGNLNFVTALVLEIAASLNNVAISSYLMSKRKSIADLISKVNTSF

KNQTKLLPFGDKHKKILDHASSRLSKFTWVIIVVNVSTGIAWVVGPFISWYTYDGDDIYKP

NRNDEIDIHWEFFSQRSWLPPNVPVYETPYYQIFCLYQAMPVFSILVNYTGYFTLACSLVT

FTTAHFQVLADLLQNVYKHIETTKLNQEYPEQQSITDEALVMDEENNFQQLSSDIKENKDI

HNEEEIKIQYDYSIKGNESHEDVHYLVNCLKYHQIILEPDQDDGFMKFLTACLSCWLPPFV

LCWYGEQLTTQSLEVQQRAYDCPWYKSSTEFKKNLQMLIMRAQKPAQMNGRWKFSISL

KGFADIANNVYVYFTILKQIQ

>BgerOR17

MATKIVTTNKDPEFRSEMDLLDFMYKFFHLTFIAPMLKTTDNSWKKRLYNLVRQILLITY

FPSFLGIVLGLYKFWGDMKTVTNMFVTGVPLIMGFCAGLYFYWYWKEIRDFMESIERESN

FINPFVHSKTKLLQIVEDTKRKCKFITQFIAIFQFFAFVAFFIKPFILDFSEESELKIAEEEYR

NEQWKRLIYIMWLPIDITSPEMYYYTYAYQLITSLALYCHTATTITFIFVSSRYAAAQFTIVC

EALNDVSILNIFSVCRYEMNTGGRDLLNSQSMPEELHPNLRKSMDSCVNGLADKEIKSNE

VHIYIRECIQIHQSAIRYAMKLNNLLSPVFVMYLSLLTIGLIMGTIQLAVVDELDQKFPYFGS

VSVTLTDLFAFCWHGQILINESLSVEQAAYNMLWYNHCSSVKQLVRFIIFRAQKMTELKRS

GFLNLSIRTFSAVLNFSYKWFTILLEMHDD

>BgerOR18P

MDRPKQAGTTSHIPRECGRRMDLIGFMFRFLHFTFLGPSQEIKNNTWKXYCIIQFVMVFS

QILSILGIALALNKFQANVGDIIDILVTGSALSTSFLCALYFIKNWDSIZNFMNSMETESCFT

NPFVRSNGDLLQIIEETKRKCTLLTKVLVMTELIGICTFFFKPFILEYLEDPNKNLTEEENIM

NHWKKMIFVIWFPLDPTNPITFYCIYICQLFAALAFXCHTASIINFNFVLIRYSALQFTLSSK

ALSQTDIITSSSNLKSXENELEVMKNENISSEMAVESQSFLRDRSDTYIDEETRGQKEAESN

EVFYYVRGCIXFPKQMNKLLSPLFVLYLLLPSIILVVSTFQLTMGGGIEKNMHNLSVSTIVLS

DTFALCWFGQTLMDESLAVKQAVYNTNWYDQPSSVKKLLSLIMVRSQNETGFPNISIATF

SMILNASYQWFTVLLKMLPE

>BgerOR19

MDIQTRLSAIHLSTECDRRMDLLGFMFKYLHFTFLGPSQETMNNTWKRRLYSLVQVIILFS

YIPAIIGQAFGLCKYREDVEDISDILVSIVPLITFFPCSLYFITNWDNIKRFMLSMETESCFAS

PFVQSKNNLLQIIEGTKKKCIILTKVIVITEICGTCCFLFKPFVLDYLENQNRNLSEEEHVKN

QWKKLMFLMWLPVDPRDPLIFYSIYTYQLFVCFVLFCHSTSIINFNFVLIRYSFIQFTLTCR

AISETDVLTSCSKLESKNDSEVVKNENISSEIVAKSQSLSRDSNDIYINEETTEQADTESSEV

YNYVRECTILHQSAISFAKEMNKLLSPLFVMNLLILSTVVVVSTFQLAMGGGVEKSLPYLSA

SMVVLMDTFALCWFGQQLMDESLAVEQAVYNTNWYDQPNSVKKLIYFIMMRSQNVVEI

KETGFPNLSIETFSVILNASYQWFTILMNMVHE

>BgerOR20

MESQASRSSIHLSGESDRRMDLLGFMFSYLHFTFLGPSQETRDTKWKKVVYNLVQFVMLF

LYIPTFLGIALGFYKFRENVTDMTDILVTGLSYVIAFPCAVYFIKNWDEIKNFMVLMETRSC

FTNPFVHSNSNLLSIIEETKRKCTFIAKVIVIIEFIGVFTFYLKPFILNYFEDPNIKLTDEEQIM

KQWKKMIFVAWFPVDPRKPVIFYSIYAYQVLTAFTFFCHTSAVMNINLVLIRYSVVQFTLT

SKALSEIHIQTSSSTLESKTHSENEKDSSSETTIEPQSTLKDSTLLYTTEETMEQADIESNEV

FNNIRECIILHQSAISFAEQMNKLLSPLILISLLISSINLVVSTFQLAMGGGLQENMPYLSVSSI

VLLYAYAFCSFGQTLIDESLAVNDAVYNIHWYNRPSSVGDMISFMIMRSQNAVEIKETGFP

NLSIATFSMIVNSSYQWFTVLLNMIHD

>BgerOR21P

MESQASRSSIHLSKESERRMELLGFMFKYLHFTFIGPSQETRDTKWKKVLYNLIQLVMLFF

YIPNFLGIALGLYKFRENVADMTDIFVIGVSYVTSFPCALYLIKNWDEIKNLMVSMERRSCF

TNPFVHSNCNLLSIIEERKRKCKFLTKAFVMTVFIAYFTFVLKAFILDYLEDPNINSTDEEHI

MKQWKKMILVVWFPLDPRNPVIFYSIYAYQVLTVFTFFCHSSAVMNFNVVLIQYSSLQFTL

TSKAISEIHNQTICSTLESETYSETMEQADTKSNKVFNNVRECILLHQSAISFANQMNSLLS

PLFVMYLLTPSITLVVSSFLLAMGGGLEENLPYLSVSSIVLMYAFVVCSFGQTLIDE

>BgerOR22

MESQASRSSIHMSRESERMDLLGFMFKYLHFTFLGPSQETRDTTWKKLLYKLVQFVMLFL

YIPTFFGIALGLYKFRENVADMTDVLVTGVAYVTSFPCALYFIKYWDEIKNFMVSMETRSC

FTNPFVYSNSNLLSIIEETKRKSAFFTKVLVIIEFIGLWTFYFKPFILDYLEDPNINLTDEERI

MKQWKKMIFVAWFPVDPRKPVIFYSIYAYQVLTAFTFFCHTSAVMNFNLVMIRYSAVQFT

LTCKTLSEIHIQTNSSTLESKTFSENGNFSSSETAVEPQSILRDLTHLNITEETTEQADTESN

EVFNNVRECIILHQSAISFAKQMNSLLSPLFTIYLLMPSITLVVSTFQLAMGRALEENLPYLS

VSSIVFLYCFELCSYGQTLIDESLAVNEAVYNIQWYNDPSSVKDLISFIIMRSQNAVEIKETG

LPNLSIATFSMIVNSSYQWFTVLLNMIH

>BgerOR23P

LENQTGRTAINFPRESERRMDLLGFMFKYLHFTFLGPSQETKDTTWKKVLYNLIQFVMLF

LYIPTFLGIALGLYKFRENIADMTDILVTGVSFMTAFPSALYFVKNWDEIRKFMVLMETKS

CFTNPFVYSNSNLLSIIEETKRKCTLLTKAFVITEFIGICTFLCKPFILNYLEDPNINLTDEEYI

MKQWKXMIYVIWFPVDLRESVIFYSIYTFQVLATMTFFCHTSSIMSFNLVMIRYSAMQFTL

TIEALSEIDNVTNSPTLESKTYSENINISSSDMTVESFLRDLSPSHIPEHTLEQADSNEVFNY

FRECILLHQIAISFAKRMNKLISPLITIWLLMPSIPLVVATFQIAMGGGLQKNAPYLNVSLVA

LSYLFALCSFGQTLIDESLAVDHAVYNIQWYTHPRCVTDLISFMIMRSQNAVEIKETGFPNI

VNASYQWFTLLLNMLHE

>BgerOR24

MENQTGRTAINFPRESERRIDLLGFMVRYLHFTFLGPSQETKDTTWKKVLYYLVQFVMLF

LYIPIFLGIALGLYKFRENIADMTDILVTGVSFMTAFPSALYFVKNWDEIRKFMVLMETKSC

FTNPFVYSNSNLLSIIEETKRKCTLLTKAFVITEFIGICTFLCKPFILNYLEDPNINLTDEEYI

MKQWKKMIYVIWFPVDLRESVIFYSIYTFQALAAMTFFCHTSSVMSFNLVMIRYSAMQFT

LTIEALSEIDNVTNSPTLESKTYSENRNISSSDMTVESFLRDLSPSHIPEHTLEQADSNEVFN

YFRECILLHQIAISFAKQMNKLLSPLFVIYLLLPSISLVVSTFQLAMGGGLEKNAPYLSVSSIV

LLYTFALCSLGQTLIDESLAVDEAVYNVNWYTHPRCVKDLINFIIMRSQNAVEIKESGFPNV

SIATFSMIVNASYQWFTVLLNMLHE

>BgerOR25I

MENQTGRTAINFPRESERRIDLLGFMFRYLHFTFLGPSQETEDTTWIKVLYNLIQFVMLFL

YIPTFLGIPLGLYKFRENIADMTDVLVTGVSFMTAFPCALYFVKNWDEIRKFMVLMETRSC

FTNPFVNSKSNLLSIIEETKRKCTLFTKVFVITELIGVCTFLCKPFILNYLEDPNINLTDVEYI

MKQWKKMIFVIWFPVDLRESVIFYSIYTFQILAAITVFCHTSSVMSFNLVMIRYSAMQFTL

TIEALCEIDNLNNSPTLESKTYSENSNISLSDMTIESQSFLRELSPLHIPEDLTEQADTESNE

VFNYVRECILLHQSAISFANQMNKLLSPLFLIYLLVPSISLVVSTFQLAMIVNASYQWFTLLL

NMLRE

>BgerOR26P

MDVQMKRTVVRQSKKCERRMDLLGFMFKVLHFSFLGPSQEIQDNTWKKTLYRLVQFIMI

FTYIPAFLGIALGLYKYQEDFGDITNILVPTLPLITYFHCALYFVKNWEAIKHLMQSMETQS

CFTSPLVHSNGNLLQIIEETKSKCKFLTKIIVIMEIFGIRXPFIIDYLEDQNISLTEEEGMKNQ

WKKLMFLIWLPVDPRNPIIFYSIYTYQSYVAIVFFCHTISIININFVLIRYAALQFTLTTKAIS

ETDILASSFILESKSDSENSTESEIDTESQSFVNDLTDIYIDEEAESNEVFNYLRECIILHQTAI

SFAKQLNTLLSPLFLMNLLIISTTAVVSTFQIAMGGGVENNIHYVLVTLIVLMDTFVLCWFG

QKLIDESIAVEEAVYSIHWYNYPSGAKKLINFVIMRSQYAVEIKEPGFPNLSIATFSMIVNAL

YQCFTVLLNMFHE

>BgerOR27

MDVKTGSTVHQSTEYSRRIDLLGFMFKYLHYTFLGPSQETMDSTWKRRLYSLVQFIMLFS

YIPALLCTALGLYEFRHDIRDITDMLVTIVPFLAAFPCGFYFIRNWDQIKQFMVSMETDSYF

TSPFVHSKDNLLKIIEDTKKKCVFLTKFLVIAEFIGLTSFFFKPFILSYLEDSQINLTEEESIRK

QWKNMIYVIWLPVDPRKPLVFYSVYIYQLFTALVFFCHSSSVININFVLIRYSAVQFTLTCK

ALSQTDILTSSSTLKSKNEMLINEYHAQSEIDSESQSFSGDLTDSSFSQDTSAQKEKESDESF

NYVRQCIILHQSAISFATEMNALLSPLIVMYLLNPSFALVVSSFQFAMGGGIEKNMPYFWV

SVVSLAETFALCWFGQKLIDESLAVEQAVYNMQWYYQSSSVKEIVSFIIMRAQNAVELKET

GFPNVSIATFSMIINASYQWFTVLLNMLNE

>BgerOR28P

MDVQMRTTLVRQSKKCERRMDLLGFMFKVLHFSFLGPSQEIHDNTWKKTLYRLVQFIMI

FTYIPALLGIALGLYKYQEDFGDITNILVPTLPLITYFHCALYFVKNWEAIKHLMQSMETQS

CFTSPLVHSNGNLLQIIEETKSKCKFLTKIIVIMEIFGIRXPFIIDYLEDQNISLTEEEGMKNQ

WKKLMFLIWLPVDPRNPIIFYSIYTYQSYVAIVFFCHTISIININFVLIRYAALQFTLTTKAIS

ETDILASSSILESKSESENSTESEIDTESQSFFNDLTDIYIDEEAESNEVFNYLRECIILHQTAI

SFAKQLNKLLSPLFLMNLLIISTTAVVSTFQLAMGGGVEQNIHYVLVTLIVLVDTFVLCLFG

QKLIDESLAVNEAVYNIQWYNHPSSVKMLINFIIMRSQNAVEIKEPGFPNLSIDTFSMIVNA

LYQCFTVLLNMLHE

>BgerOR29

MDVKTRSTVHQSTEYSRRIDLLGFMFKYLHYTFLGPSQETMDSTWKRRLYSLVQFITLFSY

IPALLCTALGLYEFRHDIRDITDILVTTVPFLTSFPTGFYFIRNWNQIKKFMVSMETDSYFT

SPFVHSKGNLLRIIEDTKKKCVFLTKFLVITEFIGLTSFFFKPFILSYLEESQINLTEEESIRKQ

WKNMIYVIWLPVDPRKPLVFYSVYIYQLFTALVFFCHSSSVININFVLIRYSAVQFTLTCKA

LSQTDILTSSFTLKSKNDSEMLINEYPTQSEIDSESQSFLGDLTDSSFHQDTSAQQGRETEE

SFNYVRQCIILHQSAISFATEMNALLSPLIVMYLLNPSFALVVSSFQFAMGGGIEKNMPYFC

VSVVALAETFALCWFGQKLIDESLAVEQTVYNMQWYYQSSSVKEIVSFIIMRAQNAVELKE

TGFPNVSIATFSMIINASYQWFTVLLNMLNE

>BgerOR30

MEESTTQLSPRFEKKMDLLTFIVKFCHFAFVGPADYIAETPWKRRCYRLVQLTLIGSYIPAF

IGIVLGLYTFWGDIEVITNILVTGCPLFTGAFIGVYCFIYWDEFKLFIDSIESKSCFSHYFVHS

KKNLLEIIEDTKLKGKLLTKFVATVEFIGLASFFFKPFLLEYLEGSNINLTEEEQIMEQWKK

MIFIIWLPTDPRQPQIFYSVYVCQFISCLAFYSHTATIITFIFVSTRYVASQFTIVTEALKEVD

VLTSIPNYRTETDTKLLGNKFETEQKNQMESRTYLEDFTESYVNENILEDEENNSKEVHD

YIRECIKIHQSSIRFAMQLNQLLSPIISFLLVDLTIVIVTAMFQLAVGDGIQNDFPYLGVAIVA

LTDLFGLCWHGQELINESQAVEFTAYNIHWYNHSSNVKYLVRFIILRAQKAVEIKGSGFINL

SINTYSAVLNFCYQWFTLLLKLHDD

>BgerOR31

MENQHNEDLEENYVVNQRLGLFEMNLKLLSVTGIVPNRNITCSKWKLKTYRLLQFLSLLI

YIPVLILQVLGLCFYWGNITLTTDNICITCSLLIGYIPALYQAVHAENLHRVIDMIEQQSLFS

MKAVKENSAYTTIVNDAKKTASYLTWLTSISLSVTGILWTSYPLVMHYFQNNSSDDNSLD

HQFKYLVFVMWLPFEISQPDCYRLTYLLQVIVFLTAMAYIIGILTLYLNIMVYLKAQLKIVT

AAIRELDKAYFFEKEEDMNQGHTFEYDKQFRQEIVQEVINETYKNKENRNTLQAVEESDI

VRKSEDIQLYSIASLELLTTKIESNNVLNGEENSDKYIVQCIKFHQSAISFSKEANNILGFGLI

VAIVCNVVLIITETFQLSLVVTTKYCSGFFTSLTQQFIFSYFGQGIINQSLAVGAAVYKVKWY

CLPVRFQRLLLLILARAQNPVKITFGKIFPLSLILFTQVLNVSYKFYTVLLQVNDP

>BgerOR32

MVNLLLCNFQSHLQTMKEDDGLKFKIVELNLKLLNASGMIPSGGIKSTVWKSRLYSIYQIF

QYLLYFPILLSQLLALYYNKNDIIVLIDNICALTIPSSSYIAAILLKFKGNAVFKLIQNVENSYI

YDLPEVKNNEKCIEALKSSKKMCRIMFWFTIGSGLSGMFFWVLSPILLDFAEQVIYDESNS

NDRNTTENKFVFAMWLPFELESSSSYVTVYIVQTVIFFVSGNALFGFFAFSLTLLIHAANQF

EIVTLMVKDIDDELSKYATDEFHSNTLPLKIRFKNQNEITKLRLDVYKTSPYYINTKLNSRG

REYEKEHAMELYHRNTSNVYYFKTDLKIEMDAEEHHATNYLRQVAKAHQIAIQNMTNTE

EIIGLPLFCITFLALTCIVGSTFILSMNPGFQQMMKSFATLTFALAQFFFFCIFGEAVITQSLA

VGEAAYSCQWFERGPAFKRLLLIVLTKAHKPVYLTLGKLLIQSMEMFAWVVQSSYKFFNL

LLQMNEQI

>BgerOR33

MTAERVDLIPLNLKLLFSAGVVPTSDIRSSIWKLRVYRIYPMFMSFLYSMVLTAQCLAIYKY

WGHLDVITDIGFTMVGIFMCYVMAGYAIKNTERILQLIKMLETELPTIEEPVKEAVRKSRIF

TYIMFFLVHGMLSTWIAAPILLRYAQDEKEEPETDEPYPYFCFVIWLPFDATQSPGYELVY

TVQTLCFLMASLYYTSINTLFITFIIHVAAQFQILVQSLKCLDDHPHDEYFINCIKHHQTIIKF

SKELDLVLSPLLFFFFCCSQMIMCVVTFQVVLTWADGTIQVKLILGLMAALCGPLMFCWF

GTVMIQEGLAVEQAVYDCKWYERPTNFRRLLGMVLMRSQKPVRLTAGQFYDVSLTSFTQ

MLNAVYTYFAVLKQLYDE

>BgerOR34

MEALSLNLRLLQISGIVEPPSVSKSGWKHVIFSMYMTTAVIIFIPILVGELLAFYHFWGDLV

VITNNMFTIVGNITFFWEALYIIVRRGAFNRLVATLQRMLKDMPRWNLKQQTIAKNSIKR

GRRLTWFMVIIVYMVPFSWSIAPLIGMLLPENEEEIQSVPEDEVEDFWKSLISIMWLPLDA

TKSPVKEIIYTCQFMIFILTASYYSSVNTVFVTFIVNLTGQLETLTATVEDMDQIIDQYDDKE

LHNVFVDIIRHHQSIIDFSQELNAVMSPLLFFYFFSTQVMMCVMAFQMVLTWGEQSNFVK

FFFGLLCVLAGPFFFCWQGNILTEQSVQAEKAVYNCQWYERSQRFKKMVLTVIMRGQKPI

ALTAGSLYVLSLDTFAKMLNSVYYYFAVLKQLHEE

>BgerOR35

MTENAIPEQFHEYEQNIFDLNKKWLYRAGIVPLYSIKRNPFKMLLYKLFILMSYILYFPTLI

GQLLALYHFWGQLNILINGLYNMVACLMCYIIGTYGLFKKNEITQLFVAFEHEILPKMANV

ILNERKIEIFKTASKRARKITWIVIIVLDIFAILWIPVPLTNHYLKERNGTASELDDGKTWIH

FCFLIWFPYDIRVTPYYEIMYLSQVILFFTACSYLKAVSMSIASLMVHIAGQFEILSETLADID

ELLTNTAIQKAAQIQNNQFLHNNIQEMADELPTSTPKTSNANLSTVVKPLSQFESKEENLE

LIADSLEQKKCFINIVKYHQSILWFLEDLNRVSGPVILASLFSCQFLGCLMIFLMTLEWAQE

KNSSNFARYIFAFICAMSFPISFCWYGNTVTESANNVRDAIYKIQWNRHTKAFNKDLLLLG

EGARKQVYISGMQFYKLSLETLREMMSTMYSLYTLLHKIYHT

>BgerOR36F

MPQSKTTETFEDMKEKALLLNLISLQKLGIALPSNVKSNPLQKMKYYLMIFFSCLVYIPTFI

GQMYALYHFWGNLDILVHGLYNMIAIFMCHLLGMYGLIMKDEIANLFVTYKDEVLTKVEK

VGFKNSTNEIFSKTSKKAHQISSSAMISLQIFFILWAPVPLATHYLENNDETMSAEDDEKR

WLHFCFLIWLPYDIRVAPYYEIMYLTQIIMFYTASCYLCYVSMTMASFIIHVAGQFEILYKAI

EDMNLFLKTAEGQKETQMQNKVTMNENIKHFPSASDTMMEYFENIDSSVHYLSTKGSAD

HLEIILDSQEHREYFLNIIKLHQAILQFADEVKKIAGPVIFLILFTCQFLGCLMIFLMTLEWK

LGSRKGENFARFGIAGICALFYSYIFCSNGDIATQNCRKVASALYENRWYHYTTKFNRDLL

MALRCAQKDIYFSGANIYKLSLETYGQMLNTMYTLYTLLQKAYEG

>BgerOR37

MPKSNTIETFEDIKKKSLHSNMESLQRIGIIQSPSTKKNPLKKMICNLLTAFTFLIGIPTLIGQ

ILALFHFWDNLFILFSGLFNFVALVLCYILGVHGVIMKDDIMQLFVTFKDELLIKTENVGIK

EKTREIFAETSKKAHHMTMYILTILVTLMVLWAPVPFYTHYTEDRNATRSAEEDEARWL

HFCFLIWFPVDIRVTPFYEIMYLSQCLIFYVNLSHIHSVGITMASFIVHIGGQFEILSKAIEDM

DTFLETTDIQDKMQRNQTMMRKTMKEHAKHSPPHFHIKTGVPKDLNENIHALSIYGSDG

DKLEFILDYPEQREYILNIVNLHQAVIEFSREVNRVAGPVILALLFICQFLTCLMIFLLTLEW

KQGTRKAENFARYAIAGLCAISFSFELCWHGENAKQSSRRLADAIYESQWYRHTVTFNRD

LFMIKELAQKEVTFSGGNFYVLSLETFGEMMNTMYTLYTLLQNAYEG

>BgerOR38

MLEALFDNSPPQVADIDEMESHELGLMNVVRLELCIVGLIPTNGIVNDRWKLKLFRYYQN

VMICVYIPVMLGQLMAIQHFWGDLDTVTDCAGMFSVFVACFFDYLYLIEHEKTILHVCEVL

EETPIPKVKNPRLIEKYLAIVEICRTEIRIVMEIFWGLAAIGAIKWLLYNPIENLIIDRHFLNH

TLKEDKPNADFVFIIWFPFDATWSPLFEIIYMFQSILLVMATCHNICANSTFLTFMVHAWG

KLEFVECTVSSIDDELNPKRVGDEAVEDKISEIFGEFDDEDTIPAADDTNDDDTTVTLEEE

AEQETGLNGDLQVFSPEGANTNSDVVPVENGVEVAMDAHVEYLKRCVKQHQDAIDFVHE

LDDLVSTWLAVRMFAFQMVAAGEVFQLIVNMDDWEKLVGHGTILFFIFAQILIYCWFGEQ

ILQGGWDVDRAVYETPWFTYSQTYRKNLILIIMRAQRPVEVTVGHYYSLSLQSCELILQNIY

FFSMFLNQINNKASKQAALAELEA

>BgerOR39

MFKQNQNLPDHKGNSDEPETGREVMSFCINLLRIAGLTPIYERNPIIAHAHNFFIFALLMT

STILQIVGLIECWGDFKPMCFISGTLSGCSAITVNYILLIKNRDAIFKVIHVLKIEYISRVNPKY

MGYVRNSEKEVVKSAILKILFGTALTGLGTIIPLINKSIDDGGSVTGNLTKYQYMTKYQIFVT

YTPLDIQQSPQFELNYVVLVILTAFVVGTDAVIDSLYYALLSHLTAQFKILVAVLDDMDENL

NFIDNEESINYSKTTSKKCEVKQRYSTDISKEEMDSIQLNEVSNVYPDGKDIHKAYLKDCIR

RHQALLSFSDDLNSMMSFAAFLQVISTPVMICMGGFLMTTSLTVFTDVIKYSSLVALAFYK

LLIYCMYAEDLTQASLKVREALYNLNWYGLSMQCQRMLPIMIIRSADAVVPKASVFYELS

MDSFGTVLNTAYTYFTLLLQMSDS

>BgerOR40

MPDINDNDIKKLNLIRPNIRLLAIFGLLIRDLTPWKQTLRRCGVYLFIVAAFFNIVSQTIEAC

RVTDARLMSEGGLWVIFGVKGVTKVITIFYYEDEIWKLINIFEENIFKNAHLLTSTEKKSAK

ESLNFSKRMLSYTLAASLLSNGTLALDILPPSEEGLQALEAASAPDWNSPTKYAAPFKGMR

NRYWYNRVGYIFSMSYLTYYPMMFIYSFNCQLIMYLATHFAILSDSLENAARNVQEMMKR

STSTSSLQVFSTNENMKEEIMTSLDREMSSKDNLEMTEDCAVRDQRFDEEMYKYLLERT

KQHQEYLLLFHRLNKITRVVVFTDILGAAVYIAACLMGMAEIHETYEVLEFGFLLMFAVVE

LGVFCWVGNYLTTQTERVGAAAYASLWYVQSTKYQRHLQLVIMRSQRPLKLSVGPFGVVS

LELFAKIMNTAYTYYTLMREFVSPKKEEAGERHHERSE

>BgerOR41

MNRNELPDDIQKLNALRANIKFLKFLGLLRTTDAMSPHWKQIAHRCLKYSIGIPISIFALGA

IVEAYYFRDNSREIIQAITAFLSVLKSVIKYLSFILHEEEFKEISEHCDGNFIIEGNDLTQRER

KMIENDMAITKKLTYSTWFMCLLLLSGLTFNVFPPSEEEIENSDRDYVPAWRSLNRYAIPF

QSARSPYFFFRFLYSVFVEICAMVPFILINTMNALVITYLTTQFSVLSDALEHIEENVQAVLE

NEGSQIVTQENGRFREESHENRRVITQDALFHWEMEMYLRRCIIHHQKLLQFFDILNNAM

RTTLFADTLVASILISMMSFSLLIATDMGDVIQNLGILTLTTTELWFFCWLATRLSVQSERI

GEAVWSSLWYKQRIKFQQHSKFILTRAQRPAQFSIYLFGTVSLELFSKIMNTAYSYFTIIKE

M

>BgerOR42P

MPGIELPDDIQKLNVFRVNVKVLQFLGFFRTSYVNTPHWKKIVHRCLKYSIGITAFIFCFGA

LVEGXRCRDYPLEVIEAITAFLTGLKALIKYLSFIVHEKEFKEIAEHCDDNFIIEGNDLSERQ

RNIISKDMATTZKATYLFWFLAVITLVTATFNVFPLSEQELENYDRVFLPAWGSLNRYAIP

LQNARSPYFLIRVIYCTFVIILACVSFIIINTMSVLVITYLTIQFAVLSDSLKNIEENVQAVLER

GGSQFVTQGQCRLEDDWHGNREVVTQNEAFHLEIERYLRCCIKHHQKLLQFFEVLNNVL

RTTLFVDILVASVLISMLSFSFLISSDFSNVIQNIGILYFITIELWFFCWIGTRLSTQSEMIGGA

VWSSAWYKQRVKFQQRSAFILMRAQRPVGFSIDIFGTVSLELFSKIMNAAYSYFTIIKQMI

>BgerOR43

MEMHNIELPADIQKLNVLRVNIKLMKLLGLLKTYDEKTSPWKKLAHRCLKYSIGLPVFIFS

LGAIVEAYRFRNYPLEIVQAITGFLSALKALIKYLVFIIHEEKFKEITERCDGNFIIEGNDLTE

RERKIIEKDMAISRKLTHFVWLMSFLMLSGMAFNVFPPSEEEIQNSERDYVPAWNSLIRFA

IPFQSAKSPYFFPRVIYSVFVEMCACVPFILLNTMNVLVITYLTTQFAVLSDSLKNIEENVLV

ILEREGSQIVPQGKARLEEGWQENRELLTRDDLYHKKMEMYLKRCIKHHQKLLQFFEILK

NAMRTTLFVDTLVASGLIAMMSSSFLIATDFVAVMENLGALTFITMELWFFCWIATRLST

QSEKIGEAIWSSPWYKQRVKFQQHSKFILLRAQKPVGFSIDIFGTVSLELFSKIMNTAYSYF

TIIKEMI

>BgerOR44P

MSGIELPDDIQKLNVLRVNIKVLQFLGFFRTSYIDIPHWKQIVHRCLKYSIGITAFIFCFGAL

VEGYRCRDYPLELIXGLKALIKYLSFTVHEKEFKEIVEHCDDLTGRQRNIISKDMATTKKAT

YLFWFLAVITLVTATFNVFPLSEQELENYDRVFLPAWGSLNRYAIPLQSARSPYFFIRVLYC

TFFTILACVPFSIINTMNVLVITYLTIQFAVLSDSLKNIEENVQAVLERGGSQFVTQGQCRLE

DDWHGNREVVIQNEAFHLEIERYLRCCIKHHQKLLQFFEVLNNVLRTTLFVDILVASVLIS

MLSFSFLISSDFSNVIQNIGILYFITIELWFFCWIGTRLSTQSEMIGEAVWSCAWYKQIIKFQ

QRSTFILMRAQRPVGFSIDM

>BgerOR45

MPRIELPDDIQKLNVLRVNIKVLQFLGFFRTSCIDIPHWKQIVQRCLKYSIGITAFIFCFGAL

VEGYRCRYYPLEVIEAITAFLTCLKALIKYLSFTVHEKEFKEIVEHCDDNFIIEGNDLSERQK

KIISKDMATTKKATYLFWFMAIVTLVTATFNIFPLSEQEIENSDRDFLPAWGSLNRYAIPL

QSARSPYFFIRVLYCTFVDILACVPFIILNTMSALVITYLTIQFAVLSDSLKNIEENVEAVLER

GGSQFVTQGQCRLEDDWHENRVVVPQNEAFHLEIERYLRCCIKHHQKLLQFFEVLNNVM

RTTLFVDILVASVLISMLSFSFLISSDFSNVIQNIGILYFITIELWFFCWLGTRLSTQSEMIGEA

IWSCAWYKQRIKFQQRSTFILMRAQRPVGIYIHIFGTVSLELFSKIMNAAYSYFTIIKQMI

>BgerOR46P

MEMHNIALRADIQKLNVLRLNIKLXLGLLKTYDERSSPWKQIAHRCLKYSIAIPVFIFSLGAI

VEAYRYRNYPLEIVQAITGFFSGMKALVKYLVFIIHEEDFKELTERCDGNFIIEGNDLTDRE

RKIIEKDMDISRKQTHFVWFMSFLMLSGMAFNVFPPSEEEIQNSDRDYVPAWSSLIRFAIP

FQSAKSPYFFPRVIYSVFVEMCACVPFILLNTMNVLVITYLTTQFAVLSDSLKNIEENVLVIL

EREGSHIVPQGKGRVEEGWQENREILTRDDLYHKEMEMYLKRCIKHHQKLLQFFEILKNA

MRTTLFVDTLVASGLIAMMSSSFLXWFFCWIATRLSTQIMNTAYSYFTIIKEMI

>BgerOR47I

MEEYNLPEDIEQFNIMRFMIRFAGFIGLNFSSRKHSERWEIVYNVMSKTMKCFIYFCMLFS

TISSLINCFYIIKRDMKEFIEAIISFVGCLKTLMKFSIIIFHEDKLKELLNIYIQNFFIHGKYLTE

SEKKIIKDSLRTAKKVTKFIWVASIVGLAGILTNVSPPTPEELEENEYLPAWKSQSRFVIPF

NEGRSPFYLLRVICTELIAVGVMSAMIWHIPFIFLLITYLTTQFSTLTDSISNIADNVREQLF

YSKLDYVLNYNFFTDAMSTSAMISILGFAVLMSSNTAQVLQYLGMLVHFSMEFWCYCFIA

NRLSTQSSMIGETAYAISWYQQTTVFQRQIMMIIMRAQRPVEMSAGLFGKMSLELFSKIM

NTAYSYFTLMKETIS

>BgerOR48

MEPVNVKRCLDLNLKLLTFSSVRPTDVGSSPLANKLYSAFTVFTLLVFSLISSGILPWLIYT

DYTLEDLIEVISILITQIRCLTRFLTFIVFRKGLRNLISTLYDNFNIHGRDFDSEEKLIINNTM

ENCRKITKYYVGLFCCTGISMVIQPLTAPEPDELEDHSNSSLPHKPLPFKAYYPINTMKSPQ

YEIVYIAQSYLALIESWGIGSLDSFCVAILMYVTCQYELLCGSLMNMKRNVAIRMQMMAHS

KMVAFQERVRTEPISDEEIQVHEDRNLPREETPVLYRNVVCNTNLCLIEEEFNTYISKCIIH

HQAMLKYVDDFNACFRPMFFSILLTASILMCMMGFQVIVIPPKGIMFVRVVLHLLCTIFEL

GFFCWFGSDVMRKSASVCDAAYFSEWEDLSSDAKKNIGMIIMRSQQPVAIQAGLFGALCL

PTFGTMMKNAYSYLALLKQLHEGDMEE

>BgerOR49

MQSHIVINFQVRKVLTHEMDSESECLKLNVIFLKIGAAWPGDVSPVTWGNTLYSAYKILYR

FLFMLCTLGIVLYCFFAYETLEDLTENISVFMTQTSLCGRFFVLAMYRKELLVLIRTVANNF

YAPGPEEKAMVQRTIAICQKLGKSIFILYVFTTLAMISHPLTADERVLPFKSWYPMMNSSV

SPFYELQYMAQASLTIMEGWFAGAMDSFVISMLLYGALQFELVAHGIRKSKSVEELRTCIQ

YHQAALQYGEDMCTALNPVFTVQVFSDNLLICVLGFQVMVMETDGIKIIRVLLHLVCAIFQ

IWFLCAFCSHFTTQSSSVYEAAYESTWYDQKPAFKQMTWMVVERARKPVALSAGLFGEM

SLPLFSAIMRSSYSYMALLSQMNEENFN

>BgerOR50aJ

MDFQTCDFEFKDCTKFVVKLLQFSGLWCTSDENVLIYRVYKVYSITVAITMFYLVFSEVLV

FFSQNEAHDIIESIIIFIANLTTLSKMLIYFLKEKEVKHILDSVNRNFSINGGTLSIENRSIIKST

LELSKRLCIAYAIMTYSGVTFYVDVVPLTTIQFSEGKNFTHQTVRKLQYSIWSPIDIQSSFNN

YIFMYIVITILSHIMANILVSTQAAFLTLIICLTGQFKLLCESLRNMSKNVRCRLQRSSGSTK

WKSKELEEDASYSDEPTDLFLAEAEIYLKECIKHHQSLIEFAEKFDNVWKTTFFVQFLTAS

FLVCFLGFQAMLMPFGLNLIKMLIFIFVELFELGVACKFGSDLMTQSEDVYRAVYDSDWY

NQSTRFKQSARMLIMRAQKPVRLTAGRLGILSQPLFAAILRSSYSYLALLRQMHDD

>BgerOR50bP

MTFDKNYCEHNNCANLAVKFZKIAGLWSESDNSSNGPMYYVYLFYSKTVLLVQLYFWFA

FIAQLFIGKTELEETIELIILIITQCHIYFKLRIFYFKKEEVVPLVGALKSNFLIHKENISTENRD

TIKSTLILTRKMCILYAIMINGGITFYTDFVPIASSSFESQGSNETNSTQRVMPFSIWLPIDM

NTSQYYLEVYLLLFAGTHCIALVLMATQAFFLTLIICLTGQFQLLCDALRNLSMNVQIRLQS

TEGSTKWKSKELEEDASYSDEPTDLFLAEAEIYLKECIKHHQSLIEFAEKFDNVWKTTFFV

QFLTASFLVCFLGFQAMLMPFGLNLIKMLIFIFVELFELGVACKFGSDLMTQSEDVYRAVY

DSDWYNQSTRFKQSARMLIMRAQKPVRLTAGRLGILSQPLFAAILRSSYSYLALLRQMHD

D

>BgerOR50cP

LVDSDQAQTVWLHRLHQHRCLYRMFSIIVLLYQLYFFFGFVVELFISNTLEDTIEVLILSISC

FHILFKIHICHFKKEQLNILIERVKNNFFIHKEYLTTENREIIHSTIHTSKTICIIYAFLVNISTV

FYIDFVPIANSIFYSQSKNSMNATHRELPFKIWVPIDLPTTPYXYIHVYAFLAIGSHAMTNV

VVATETMFCSLIICLTGQFKLLSDSLRNVFKRVEMRLHPRTGSTKWKSKELEEDASYSDEP

TDLFLAEAEIYLKECIKHHQSLIEFAEKFDNVWKTTFFVQFLTASFLVCFLGFQAMLMPFG

LNLIKMLIFIFVELFELGVACKFGSDLMTQSEDVYRAVYDSDWYNQSTRFKQSARMLIMR

AQKPVRLTAGRLGILSQPLFAAILRSSYSYLALLRQMHDD

>BgerOR50d

MNTCEKHSRYYSEHEECTKIAVMFLRIGGLWSLNSGFIFVNRLYYIYSSFTKFMTFYFLIAFI

LELFANNTLEETMEILILWITSIHLLAKLLIFHFNKQQIEILFETVKNNFSVHKHYLTTENR

KIILSTMQMSRKINIFYAVLLNLGMTFYVDFVPLVTSVTYTNGTTIMERKLPFKVWVPLDI

TTSAYYVHAYVFLTVASHILATLLVATQSVFLTLIICLTGQFELLCDALRNMLTNVNYRLQH

NSDKGSTKWKSKELEEDASYSDEPTDLFLAEAEIYLKECIKHHQSLIEFAEKFDNVWKTTF

FVQFLTASFLVCFLGFQAMLMPFGLNLIKMLIFIFVELFELGVACKFGSDLMTQSEDVYRA

VYDSDWYNQSTRFKQSARMLIMRAQKPVRLTAGRLGILSQPLFAAILRSSYSYLALLRQM

HDD

>BgerOR50e

MMSFDCNDESDHYSCANLAVKFIKIAGLWPTSEHFSVRNCVYKLYYVMVFLIQLYYFFGLT

IQLFFVDTVLEETFEIIIFLITEVHILYKTRIFYFMKKEILSLLGSLRINFVIHKEFLTTENKQII

HSTAVLTRNMCLLYAIMINGGITFYCEFVPIATSVYDYCVHNESNILPRKPPYSIWLPIDMA

NSDYYIEIFLMLTLASHCIALVLMSTQAIFLTLIICLAGQFELLSDALRNLSKNVQCRLQSTM

GSTKWKSKELEEDASYSDEPTDLFLAEAEIYLKECIKHHQSLIEFAEKFDNVWKTTFFVQF

LTASFLVCFLGFQAMLMPFGLNLIKMLIFIFVELFELGVACKFGSDLMTQSEDVYRAVYDS

DWYNQSTRFKQSARMLIMRAQKPVRLTAGRLGILSQPLFAAILRSSYSYLALLRQMHDD

>BgerOR51a

MKTGFDDELNKRVDLCMEFPILMLKNSGVWTSVFTTRPQLHKIHQRVLLVLITVMTITGV

TGIYVYRKRIEVVFELLGLLISHSVLICKLYIFVYCKEDVEDILNRVRTNFTIHEQRLTVENK

DIIKEVINKTRMIVLVFVSLSGFTTIFYFIVTPLVNIYMQNKLIFLQNDTEIYEPPLKILPVQL

YIPFEIDKSPVYELVYLLVSILAVNECFTFTAIETIIMSLLIYIPSQYSLLCDSLRNATGNVKM

RLQQNTIISDVCEFDSSPKHSEEKKEDNIDCDKDINEVSYNNINDNDGKNITEQIFQQEME

KYLRECVAHHQKLLEFTEKLDQLWEFAFFFQFMTVSLLICFIGFQAMSGPLDANLFKMLG

YLVSVLFQIFIYCAFGSNLTAKSAEVFNAVYDTDWYNQNNSYKLITKMMIMRSQKPVFLT

AGRFGALCLPLFTSMIRSSYSYLALLRQMQDS

>BgerOR51b

MYGILHTSLKISGVWCGAKNTMTLSNRLHQRFLLVVMAYAAIGIVMGIYTHSKRFQVVIEL

VCLSISNLVINLKLYVFIFRSEDFHYVLQGVRTHFFVHDRQLTLENKTIITDILNKVRNLLM

VYVSIMTYSSGTFIFITPFVNIYLEKNKSFMNNTETDETPLRILPIQLWLPFDINESPAYELG

FLIILITAVVLCIAFTAAEATIISLMVYIIGHYDVFCDSLRNATRNVKTRIQQKNIMADVCEF

DSSPKHSEEKKEDNIDCDKDINEVSYNNINDNDGKNITEQIFQQEMEKYLRECVAHHQKL

LEFTEKLDQLWEFAFFFQFMTVSLLICFIGFQAMSGPLDANLFKMLGYLVSVLFQIFIYCAF

GSNLTAKSAEVFNAVYDTDWYNQNNSYKLITKMMIMRSQKPVFLTAGRFGALCLPLFTS

MIRSSYSYLALLRQMQDS

>BgerOR52

MTSMKVHNSFNLVEKMMHISGLWEKQYDCILSARLYRCYTYIVKFFMIYFAIGIPMKIIYPE

STEKAMEMVIMGIAHVILLFKLYLFYFRKKELEQVMSDVKKNFHIHGNRLTTENQKIIYET

MLKARYICVTFAIMLYFSFFVYCDILPLVTIKNHEYTFNFESTGCLIHNITITYSEMRLPIDV

WLPYSLKKTFVFQLTYTLLLIGCQVEAFNYISTDALFITLILYISGQFELLCDSLRSMPKNLEI

RLAKIYTKVPECNDLRFDVLQREAEMYIRECAVHHQHLIRESRRVESLWRRVFFAQFLIES

LWICLMRFRAMTMENRTDILMMILMLVCILMQMSLYCSFGSQLLTQSENVSNAVYSTDW

YNQSENFKLTARMIIMRAQKPVRISAGLFGTISMPLLTKILRSSYSYLTLLLNLTEA

>BgerOR53

MKRAMSRFSTKNNFYSKPRIKTQDVENCFRLIHKLLVLSGTWELKNTSTFWIITYQCYSLF

TKFSFVFLAFALPLKIINSEQPTDEVESFITSIAHSILLFKVSIIYFHKEDLKCIMTLVKRNFYI

HDKKLTTENEDIIVVTLQKAKFVTIVFVTMITSTFFIYAGILPFLTATENEYGIMSEFDDPLT

HNTTFRELHMRLPVNVWIPLDVNKSLNYELVFVPLLVGCVVEGLNLTVIDSLYFTLMIYM

TGQFELLCDSIHKISQDTTQRFLETKENTLDNQGCEPEWKSLQKEAEIYIREYAVYHQSLIL

ASEKIDKLWGRMFFVQFLVESIWLCLMGFEVMRMEMDSNRMIMIMLLICCLVQMGLYCI

FGSNLMQQSENVYKAAYGSDWYNQSKYYKQTTRMMIMRAQKPVKITAGLFGPVSMPLFA

YILQSSYSYLALLRQLNEQ

>BgerOR54

MTSKGEEGNYDDCLKVALFLMNLVGLGYNTNFSKVTGKLYRIYQVFTQMGVIIFTVGVTV

EMYVLRDDTENALEMIGWIITHIILCYKLYVFVIRKPEINYLISTLPKNFVIDGKNRRIDNKD

LADIVMKGARSVVMTYAIVMGSSIVLYVDISPLINYMTSAAEVQNITLFNQTEGAQRELPV

RLWLPFDTTETPMFEITYVYLAICAHTEGMLSCSIDVFCMSVIIILTGQFELLCDALKNSTN

YSLARSESTTQKGVNYDNQQKIMDNDIPNISNYDLEEAEDHLEECIKHHQKLIDFSERLNS

LVSSIFFLQFLTASIMICMLGFRLTTMDFDINLMKLLSYLLTCICQLCLYSIFGSNLMTQSEA

VHNAVYDCEWYDQSNHFKKSITMIIMRAQKPVTIMAGQFGSLCLPLFASMMQSSYSYLAL

LMQLNEEVEDE

>BgerOR55a

MDKSCEWSDIETEKQARNRINFYVKLLKIGGLWHMGKSELSEFLSSFYKKVLIIWFFIHGL

SIYISLCESRNNFDDLVEVLPICISTTIFYIKIYAFFFRRIEMEHIVQNVRDNFFIHRNGLTLE

NKTIIMSTLNQGKKFTIIYLSWNCFVNCLFSVVTPLLATAPETANGTDAVSPLIIKIWTPVD

HTVSPNYEIISLYISITCIMLGFDIFVTDLFIMILIIYCTGQFELLSDSLSKASVNVKKKLLQEE

PIKLLKIEGEQEVKMEENMSSTLSTKLMHSETEKYLMDCIRHHQSLIEFAARVEDLWKTY

FFAQFLTASFLICFLGYKSMTMDMDVNLLKNMGYLGSVIFQLALQCLFGSNLMTESSAVY

DAVYSSDWYNQSNKYKFCSRMMIMRAQKPVQIRAGRFGIMSLPLFASMMRSSYSYLALLK

QMQEE

>BgerOR55b

MSKRSEWSDAEIEKNARNRMNTFVKLLKFGGIWDMRKSRLFGVFSSLYNKILIFWCGLHG

FTVYASLCESRNNFDDFVEVMPLCISMTIFYLKLYAFIFRRNEIEDIIQKVRENFFIHGNGLT

SENKTIIMSTIKQGKKFTIIYLSWNGFVNVFYGVVSPLLAPVPEMANGTDAIYELPMKIWT

PLDLSVSPNYEIVSVYVSLTCIVIGFNFFLTELFIMVLIIYCTGQFELLCDSLSKASTNVKRKL

LQEESKSDTLLKIGEIEGEQEVKMEENMSSTLSTKLMHSETEKYLMDCIRHHQSLIEFAAR

VEDLWKTYFFAQFLTASFLICFLGYKSMTMDMDVNLLKNMGYLGSVIFQLALQCLFGSNL

MTESSAVYDAVYSSDWYNQSNKYKFCSRMMIMRAQKPVQIRAGRFGIMSLPLFASMMRS

SYSYLALLKQMQEE

>BgerOR55c

MDKTSELSDAQIEISVKNRMNLYVKLLKFGGLWQLKKSKLTGFLIFFYNKILLMWFIYHGF

SIYVSLYESRNNFDDLVEVLPLCISMTLFYLKIYAFIFRRKEIENIVQKVRENFFIHRNRLTLE

NKTIIMSTIKQGKKFTIIYLSWNGFVNFIFSVVTPLLATVPETANGTETVSPLLVKIWTPVD

PSVSPNYEIISIYISVTCIVIGFDFFVTDLFIMVLIIYCTGQFKLLCDSLSKAQANVNRKLLKEE

LKTHTLLKCAEVGGEQEVKMEENMSSTLSTKLMHSETEKYLMDCIRHHQSLIEFAARVED

LWKTYFFAQFLTASFLICFLGYKSMTMDMDVNLLKNMGYLGSVIFQLALQCLFGSNLMTE

SSAVYDAVYSSDWYNQSNKYKFCSRMMIMRAQKPVQIRAGRFGIMSLPLFASMMRSSYSY

LALLKQMQEE

>BgerOR55d

MNSDSELSDFEIENNVRNCMNLLVKLLKIGGLWCTEKSGYTLNFFYNKIVIILIYIHGVAVF

MKLYESRNNFDELLEVMSVSISVSMYFLKVNAFIFKKTEIEHILQNVKENLFIHKNKLMIE

NKKIIMSIIKHGRKITVIYMSWNLTINILYTLVSPLLAPIPETVNATDFVRPLAMKLWTPFD

QTVSPNHEIMTAYVSIAGILHGFNFFVTDLFIMIMMVYCSGQFVLLCDSLFETTANVKKMI

LDDQNKSFNQPTLNVSLKKQKRKGEQEVKMEENMSSTLSTKLMHSETEKYLMDCIRHHQ

SLIEFAARVEDLWKTYFFAQFLTASFLICFLGYKSMTMDMDVNLLKNMGYLGSVIFQLAL

QCLFGSNLMTESSAVYDAVYSSDWYNQSNKYKFCSRMMIMRAQKPVQIRAGRFGIMSLPL

FASMMRSSYSYLALLKQMQEE

>BgerOR56

MACGNQKRPSSYKEELIINTFKLNRTFLYLSGLVPSDAIVAVPWKLGLYRVYTFFTLFIISTG

IIATFVSIFEHWQDVERAGESAFVCISFVLVFLGSIYILLYWDELQKLMRTAEHEILSNIQKT

YVKHLHEAERFSRFLTRFLFISCLSVAVTRSYVPLIIHYVRKYVYGAGGTNRHTLTLLFDM

WFPFHIDNTPLFLLAYIIQMLIQCISVAHNVSVITYFFTLFIYSCTRFKILQAALKSIDKFIPEY

EEYDHKLSSELIQTDNCEFSDSSKTVNASSISEYNLIEEKTLSDIRFANVENEELNDSPGYDV

TFPGYYYERDFTHGRSSKMSEEIRNTNSKSSCSKSSEVPRPNLKDAGYNIPGMSAQYPNQV

IQSENSIYTVSIDVYCKNKNAENITEHIELEVVETKRTSVTVIDKRIVHEEDMDSSTFVEEE

MSVPNDKEPQNRKAERYFIECIREHQDVISFVDNLNNIISTWTFLYSWLEHCTVYYSILYLV

ESMENMKFFLRALFGLIGELIRVYILCFFGENIIHKSLEIHEALYEcRWYNCCPKLKRLFHIS

MMRAQKPSVLKTGKWGFLSNETFSELITNAYICFILILELRISNHHIS

>BgerOR57a

MYFIKLFRSPEKVNVLQMNLGFLRIFGVLAQQSVNTSRLRVLIDYLLKWAMAVLILFFLFG

TTVRLSFVLDDFSKTADTLLFVITHVKSTVKISSLIAFREKFLNLITSIEENTYIKGINPLKREI

SCVDGYKKLARKICQFTWVTFFLSVSTWFTKLPQKPDLDLLANSTLGEYRRESFFNLWLP

VKGAESPYFELFNIFEYLAIGAVYLFVTLMNSSIIVLIIHKTAQFALLAETIENTNIHADEQN

QMKKATSNNEITKKESTKASEEMSTPARKVKFSSLEVEEVLLFDSCNPPPEVDELYINQVD

THMRLCVQYHQNLLKQAKILDKALALIMFTQILSSSFLLAIIGVLMATAKDSSTMTMGATY

LSYVLMETGLLCWIATQLRIQSEMVGQAAYNCMWYGYPKRIKLSLRFIIKRSQKPVILSLGP

FGSLSMELFGKILNSAYSYFTLMKDVSRN

>BgerOR57bP

MYFIKLFRSPEKVNVLQMNLAFLQFFGVLAQQSVNTSRLRVLIDYLLKWTMALLILFFLFG

TTAQLSFVLDDFSKTADNLVYVITYVKTTIKISSLIAFRDKFLXNLITSIEENTYIKGINPLKR

EISCVDGYKNLARKICQFTWVTFSLSISTWFTKLPQKPDLDLLANSTIKYRRESFFNLWLP

VKGAESPYFELVNIFEYLSTSVVYLFVTLMNASITVLIIHKTAQFALLAETIENTNIHADEQT

QMKATSNNEIAKKESTKASEEMSTPARKVKFSSLEVEEVLLFDSCNPPPEVDELYINQVDT

HMRLCVQYHQNLLKQAKILDKALALIMFTQILSSSFLLAIIGVLMATAKDSSTMTMGATYL

SYVLMETGLLCWIATQLRIQSEMVGQAAYNCMWYGYPKRIKLSLRFIIKRSQKPVILSLGPF

GSLSMELFGKILNSAYSYFTLMKDVSRN

>BgerOR58

MEVVIKPLPNDLRKLSPFGMILSILRCGGLLVDTSSIKNWSSAIGYSLRKTLTITCLFIVGIGF

LVETYLSRHNVEEFSECLTLVLKQTRNTTRLVSLFFHRKKILDLIKSIENGFFIHDRELNKE

EYSMIRHYLRNSRRFSYLYWLQWFLVLLFEVSAQRPPEESLETQNISMPISNQMPIKLWVP

FDTAESPFYEIGYFYNTFFCVIGSLLAAVTDTLIFGFLFSLTSQFNFLGLSLRNMSRDVAMT

MHPETLETINALVIQGQLNDDGTNNDDLTSDGTNFNEEIIKHMARCIRYHQQLLDNVEIF

NSFLSPIEGIEVLSASVLIALSGFQIIAGQFNAVHLPRQVSFLSFIVLALGLHCWFANRLTNQ

SEEVYQDMYASEWFLLPVNVQHSVPFIIMRAQRPVLLSAGPFFKLSLETFGKIMQTSYSYL

TLLTQFYAED

>BgerOR59

MEEDTAPKWLLEEDALIVIKNLLRFCALLPGKTKREILQRKITVNVILILMIFTVIGAFIEAY

NTRSNFMGFIQCGTVCITLVKCLFKIWYTRFKEKDLRYLLDTLPRNFYVKDNVHQEKILST

IRAKKKTAWILSVPYTSIFIFTIFLIALDKMSSLTYRPPEGTIIVNGTSNASLFIRTLPLRIWLP

LDEQKSPYYEIGYLYQLTIFTYQIYSTCVIDACIAVLVMYASVQFELLASTIEHAKQNVKELL

EENAADGRQVLSGEVTDDEEPNIKDSKWRSEMDNYLKLCVKRHQALLEYIRRLNIISSPIE

LLQALTSSILICTLGFTAITSGNTAILPKVILYTACAFLQIGLPCYCATQLQTQSMAVAEAAY

NCAWYEEPVSFQKSISLILMRAQKPELIFVGPFGTMSLELFAGVAQSAYSYLTLLRQVYE

>BgerOR60F

MGENTVPTWLLEEDALSLHKQIFPYFGLLPGNTTSEKRRTRIIGIISCTLMTGMVIGSLIEA

YQNLSSFKGRIASLSASITQIKCLLKTLVLIYYEDDVRYLLDKIMENFQVCSDIEKEKIISTIRA

KKRTAWWITVLYIGCFLGSILLVPIESIPDLLYRNPLATNGTNNTLEIKEFKRRLPLRVWLP

IDEQKTPYYEIGFFYQMIFFTYEILTACSIDTFIAILIMYASVQFELLGSAIEDKEEKVKTLLE

RKLSNEMIGSQNGPRMRSSEAETTKHIGKINLEKYISQSLNDDIDNIEEHASDEWNKEMF

NYLGLCVKRHQSLLEYVERLNIISSPIEFCQAFSSSLLICTTVFVVITLTKVSALPQYIMYASL

ALLQIALQCIYATELQIQSEKVADAAYNCAWYSDTLSFQKSLIFIIVRAQKQVVLEVGPFGTL

SLEFLGWVVQTAYSYLTILLQAYD

>BgerOR61

MKMEHTLHSDKQESGTMDLLGFMIKLSHYCFIVPKKTMVDSSWKYKLFRLTQMILVLLF

PPTLFTKMIAMNHFWGQMEVITYILVTGLPMYIGFHGGAYFFKNCKEIRNIVDQMETNSV

FTHPLVHSKDHLKQILIDTKRKCINITLLVTVSECITLFGFIMKPFIVSHLLDKEGKTDEEIV

DEIWKNIIFTMWLPIDPRDPLTYYILYTFQVVIALAYFCFTGPIVALIFVLARYASAQFAVVS

AALKDVDVLFNFPKPHLTIDTKLKNQFEMKQELQRASLIMEVSESILVNEDVNTNEMYSY

VRECVRLHQSAIRFAKEVNDVFSPIFFLYLSVHTAILVVVVFQIAVDSSTGSRVLFGCLASVI

LMHCFGFCWHGQELIDQSLAVERAAYNILWYNHPRSVKELFLLIILRSQKEVELKGLGYIN

LSINTFSLILNSVYQWLTVLLNMHDE

>BgerOR62

MDQLPSHVYMSTQLKVSRIFGIWPYPEGTPLWKKVASKFVFYVLLGLKVASSYLVLYHIYY

EWGHLPDPIDTVINITAHFNTTFGMMYIPCKIKKFQALLKLMDRTFMVSEDKSEDDFEKD

RQRLFERAMKSAAFVTKLFISVGLMTGVFYSIQPLGQQDGERKLPYRVTFPFGIDVSSGTN

YWLAYSLLMANFFGMCINGTINCDMFVSLIIKTTCQFSFLKHMLLNIRESVRRQKNTFNIR

LESADSKVEEKSLKVSTHKEEEDKTSKNEIEFLDDQENELIVKRLNECLKFHQDMLLFVKE

MDEHVSLFMLAQIGVYVGFLCMNIFALSIVPMLSPLFLQVAMFITSISCLLLVFSWYGNELQ

LKSLEVADAAYNCDWVNGPEPFKKSLIMLIWRAQKPVKLTAWKFFTVDLNLFITVMKTS

YSYYQVMHTMYSEE

>BgerOR63

MEQLPSEFYLKTQLKFSRNWGVWPWTEETPLWKKIFCKIIFYITLFLKLAAFACLLYHVYV

EWGHLPNPIDNIILASALLNCSFGMIYIPYKIEGFLDLLKSMDRIFVIPKDESDDELERFKKQ

TFERYMKSAALVTRLFLGSSLATGIMSGIEPLAYEEGKRPFPLLVSFPFDCSSGIHYWYAYF

FLMASWMSCCINGTINCDLFVSLIIKTTCQFHFLELGLLKIRDTAIKQKRTRTESVKQLKIK

FLDPSEEELDAEEDDLMVQNLKVCIKYHQDLLGFAAALEAHVSPFVFMQMFVYFTFLCM

NIFAISLVPVASMEFMEQLLFIICITCLLLILSWYGNELKLKSMDVAKAAYDCDWLNGPLEF

KKSLVTLILRAQKPVILTALKFFTVDLNLFIVVMKTSYSYYQVLHTMYSKES

>BgerOR64P

MGEQPTPASTNETFEMALPDMAVRSSEVYLEKQLKVQRFFGIWPHSEDAAPWKKVSSKV

LFYVMLVMKLVSLPLILYHIYDEWGALPNPIDTMVVIASHFNTIFGMIYIPYNYGDFQSLVG

LIDKTFVIPRSGSGDESQHRVFKKTMRTASFLTNLYVGLAVTLSVLYGIQPXAFPPEERPLP

YRLKVPFGIDVSSGRNYWIAYCVTLANWMGITINAAIVCDFLISMTIKTTCQFKVLELRLLQ

IRDFVRKQKQEKEQPAATVEENQLLLRRLRECLKYHQDILTFVQELDYHVSPFILGQISVYL

VFICMNIFALSFLPVLSVMFMQCAMFIVCILFLLLMFSWFGNELRLQSETVAEAAYNCDW

VDAPQDFRRALVLLIWRAQKPVKLTGWKFFTVDLNLFISVLQTSYSYFQVMRTVYSEEQE

>BgerOR65

MAVRSSEVYLEKQLKVLRFFGIWPHSEDAATWKKVSSKVLFYVMLVMKLVSLPLILYHIY

DEWGALPNPIDTMVVIASHFNTIFGMIYIPYNYGDFQSLVGLIDKTFVIPRSGSGDESQHRV

FKKTMRTTSLLTNLYIGMAISLSVLYGIQPFAFPPEERPLPYRLKVPFGIDVSSGRNYWIAY

CVTMVNWMGMTINGGIVCDFLISMTIKTTCQFKVLELMLLQIRDFVRKQKQEKEQPAATV

EELEELESELLLQRLRECLKYHQDILTFVQELDYHVSPYILGQISVYLVFICMNIFAMSFLPV

LSVMFMQCAMFVVSTLFLMLMFCWFDNELRLQSETVAEAAYNCDWVDAPQDFRRALVL

LIWRAQKPVKLTGWKFFTVDLNLFISVLQTSYSYFQVMRTVYIEEQE

>BgerOR66

MESFLLIMKNKLQAIDLNQSKNDLSINVNTNIQYLKMLAIWPLKEDIQIWKRAIMYVVFVI

GVFVQFIIICSQILDIVVGVDEHGELTDNIFMTGIAFNGLFKQVYVAYRKKSFQSLVKSIDKV

FYKAQEPFKNEKKIILENSLFYGKLVTWSIICVCIFSGFCYPLIPLSAGFHSLVDSNSTAPRPL

PHSGWFPFDKNESPYYEIVYAIMSFNAFYIALYASSTDTLIISLMIHTFKQFEILQFSIRNVK

QYAINRINAPNNQFGQNERHLSITSDTTVYSYNSNNISLIKKTNKNGFQKLNNENYMTDK

ESREKFNEELNICLGLCIKKHQELLSFSEHLNDLASPFLLVQLVVDVSFLCILTLHMTVVPV

MSFKFISAFGIVMAVLNIVGLISWCSGELSVKSEGIRDAAFECEWETTSSHFKNSLKFLMLR

AMKPCTIKAGKFMDFNLANYAMVLKASYSYFTVLQRVYHDK

>BgerOR67

MEDSSKKEEMSAHKYLGAHLALLEAGAMWPLRKLKTPTHNQIGMVGLIISVICQILLMIM

EQGYLYSSTSDPLEIIAYIGTACLRIEALGKLLYMLFIRKKVKNLLQSLDTCFKLSIHGENEG

EELDRKEEVFSIMKSWSYGAKIMAVSWTGLCTFGGTQWALVPFGINGYVTVTLDGDEHL

HQGNLTNFTEIGPRTETFYLRVLPLRGWYPFNETLTPAYETIFLIQGIGNICTAFAVGIFDQ

FYCAVALLLCGQFECLKNSLRNINIKDKSAILYDISANLTQLKFERNEKDLIANKNDEFSIQY

SLHDGNIENIKELIFRLTHKFDDDLENDYEETTREKTLNSEQMAMEDELKACIKHHQKIVS

CASELNMVYSPMMFMQCQKSLLALCLVAFQSTMVNGDLLQLFSLVGYVMLLVIQLFLFC

WCASELTERGKSVQDAAYDSGWPDANFGYKSSLIIMIHNCQVPILLTGGAFYILTMELFIEL

LRLSFSTYTVLKEMHEGQQD

>BgerOR68

MDLVSPKMIKFLGIEPERGAKEFLNFNLQRLRYLGIWRWDVPHFKLNQAFACVQIIAIFLF

SVTEILSAFDNLNDLDHLTKLCITTVFVYLLIFKNIYLAMRMDYACELIDMLENKFFTSSRP

PTQEQLMIVNRYGARAKFFTILRSNMALSIITFWLCAPLKDMLTEFTSDISFNEDYHSEEV

ESVVNKTSERQLPFVATFPFDIQNSLFNYIIGYVFQVFCGFTVFIGMPAWDMMFVSIFIHTS

GHFKALQHVLFNLQRNLSQTKMRQDYYSQAVQSSLLAEEQDQCNTNTEALDTSSMQVIIA

NQDLDDNMLETLNNCIQHHQLILNFVEKLEGLLGPVMLMQMMASVIAFCVIGFQMSVIPI

STESVKFVKLSVSLVSALVEQGMFYWFGGELLEESAQTLNAAYHCEWYSTDMKFRQNLR

LMMERAKRPVKLTAGGFSLLTLESFASVVQSSYSYFTMLKAVHDEDQE

>BgerOR69

MEYCLVFNINLLHYFGIWPSEDTRKPWKKAAYRMVSIFLLLQLILFYITELAGFYMNWGDI

RKMTEAMCQITANTHLICKISYLLFNHKRFHKLLQSLNNISSHYPGIKNNNNLVFVKNCES

VIKGFTFAYIGAGWLVGIFWVISPLIDNESGHSELPFHSWTPFNVSDPVLYSVMYTLHVMH

ASLFSTYIPSCSMFIFGLICHASARFKILHSLLLQSEISMESDKRMDKKEHEHKGLELYNYAF

PEVEVKGNRKDTNNYKNLLNEELTTLQECIIYHQSILRFCKELEEILSPIMFMEIITTGIALC

VVAFQVTEVPVYSLRFFTMATFLISVLFECGIFFWAGEQLITESLKTADIIYGCMQWYHNCS

LFSRGIPIIIACSQSEVKLTCGKFYILSLSNYANVIKTSYSFYAVLKSLHDS

>BgerOR70F

MAKVLDEEFNRKQLMNLNIKTLQLVGLWRGSMFAMKYKWRKMAYNVYGWLLWLVLF

MNLISQSMDVFLTRRNLEEMANNGCTTLTYAAAVAKQFMFLLNHKKILNLVEALHSGTL

SSSLKWSADQDNVLRIAHFQCRFVSWCYYCFGIVTLLLIDLTAITKSFPEAFPFLEVTPSNQ

TVKFLPFNAWYPFDVQAPLNYEMVCTFQIVMGMFGPTVNIGIDTFIVSLIICCSGHFRVLKY

SLRAIDSVSLSHLEIEDKDYICLIECIHHHQPLLKLVGDIEEVLSSCLFVQFLSSSCTICLLLFII

AVRIDSDGIVAQLSSIQFLSISFLQLLLFCWYGQQLIYQSDSLTTAMYESPWYDSSKGYRRSL

CIMMSQTMKLCSLTGGKLYILSLETFRAILTASFSYYTVLRNLNFGQ

>BgerOR71

MTLFNKNEKIYRNNNKTFMNINVTALQIVGLWPNINIPPKYEWIKMIYVYYGWTFFVVN

VLNVATQIADIAYTWGDLENLAANGGVTLMYIACILKQLNFFIYRRKIAEMVENIQNGFFS

DSLTWDDERNRIANSSNKFALTVSWTYFGVTACTVGTFILGGFLSSYPEIFGVEPLMVGNQ

TIKVLPLKEVFPFDIQKRGLFEIAFIFQFILLTLGPLLNAGMDNCMTSLIVHCCAQFKILKYA

LRNIDKRAYVLLGYEKTDETEDSNFTAKANDGEISNLTTNKLEYGERFPERVNEKAYKCL

RECIQHHQNVLEFFDDLRNVLSMYLFGQFLCSTSVLCLILFYIGMGVESENGFAEKVGFYQL

FVTATMQILMYCWFGNDLTYESDSLTKAMYDSPWYEASVEYRKNLCIMMARTVKLCYLT

GGQLYIASLETFRSIMGASFSYYTVLSSMKVEEN

>BgerOR72

MFSNKNEDESRFPDDFLRVNIKTLWMCGLWNCFGIQRKENFTYTLFSIMASFVLLHQSVT

QTAYLMFNMKYFAILISSAGLILTYLTLLFKRFVYLTVEQRIYRLVNGLRDGDLSSSSNWTE

EQVEMAKDYDRRARNMSWSYYWLGVVCLFFLSMSGIVNGPLKEDKSVNSTSWRNLPYN

EAIFEFDIQNVVYYGIALVLQYLAVFFGPTTNIGIDTMFVALVIHASGQIQILKLSLTKMKER

AIRMEPDDLNIRNTDRPIWDKIDPDSITEELKLIESFNDQFDEDNLESTQYENDEGRNYLS

NLLQVKLSMCLNDCIRHHQEIAKFTTELEYIFSPMSLIQFLSSSVTLCLIIFSIAVSDVSRLPT

NIVFLSISITQLLLYCWFGSELTYQFESLVAAIYDAPWYGSSLNFQKNILTMLIRATKSFILT

GGTIYLMNLNTFLTMMKASFSYYTMLKEMDIS

>BgerOR73

MSGKNEDKRKFPDDFLRVNIKTLKLCGLWNCFGIERRGKHLYTVFSGIGSFIILHHNLTQIA

YAIYNRHDFVKVISVAGLIVTYGTLFLKRILFLLLNKRIYRLVYKLRDGELSSSDNWTEEEA

DMAKNYDQRARMMSWSYYWLGIVCLFFFCLTGMLTGVIEEEEFMNKTTTSIRNLPYNEA

VFEFDIQKGQYYAIALLLQGLVVFFGPTSNIGQDTMFVALVIHASGQIKILKLALRKMKERA

IRMKDTFKSEKSSQFNIHNTDIFNKDETDSINEDLNLIEAFKNEFNEDIHLKIYEDQSYLTR

LLQVKLAMCLNDCIRHHQEIAKFTKELQDIFSPISLIQFLSSSVALCVITFTIAVSDASQIVTYI

VFLAISIMQLLFFCWFGSELTYQFETLGEAIYDSPWYESSLDFQKNIHTMLIRSTKSFILTGG

NIYLMNLNTFLTMMKASFSYYTMLKEMDVS

>BgerOR74

MDLHSYLKEKALTPYEPQEYDFMAINIKAIHVVGLWNYHWKKKNQWRYYAYCLFSSIQIS

ILAVHTVTQFLDLCINSHDMATYATTAWLTINYFAATTKQVFFVYHRDELQDTTLKLKGG

ILSKGLRWSKEQDDIALKTHKQVRTLSLIYDWMGVICVIGIVSIAIQSSYYKLYAEFTGLQNY

TGQETEFQLPLQAWLPFDIQKPGNYLIAFTFQVTTLLIGPIVNIGTDGFMAGLMIHACGEF

RILKHSLKMLKRRARQLQTEETEIRKLSSECPSELQPGMDIQEIDESTAKNDDSMLNRTSG

DLRPYLYKALVECIQHHQEIHKFISELEEIFCSLMFIQFLSISVRLCLNVISMTLSGSKLLVQM

TNVPLISVTFMQGLLYCWFGSELTYQSESVARVIYETPFLEASYRFKRNIIIMMMRAQKRT

QMTGGKIYVLSLDTFQALVYASFSFFRLLQEFA

>BgerOR75

MAINIKAIHVVGLWNYHWKNKNQWRYYAYCLFSSIQISILAVHTFTQFLDLCINRHDMAT

LAFTAWSTINYFAATAKQVFFVYHRDELQNVTMKLKGGILSKGLRWSKEQDDIALKTHK

QVRTLSLIYDWLGVFCVIGTVSIAIQSSYYKLHAEFAGLQNYTGQDTAFELPLQAWLPFDIQ

KPGNYLIAFTFQVTTLLIGPIVNIGTDGFMAGLMIHACGEFRILKHSLKMLKRRARQLQTE

ETENRKVPLDAENPSELQSRMDIQEIDESTRKNVDSIVHRTPQSEDLRPYLYKALVECIQH

HQEIHKFISELEEIFCSLMFIQFLSISVRLCLNVISLTLSGTKLLVQMTNVPLISVTFMQGLLY

CWFGSELTYQSESVARVIYETPFLEASYRFKRNIIIMMMRAQKRTRMTGGKIYVLSLDTFQ

ALVYASFSFFRLLQEFS

>BgerOR76

MEELNSASHLRFNFWVLSVVGMWPASNYIILGLSYILTVIIFAFWLSFLALGISVRNDMNR

LPVCVGFVCGYFVSLYKWSRIVVYRKDIQDLIRSFTRCFKLGFVAAKDDSDSWRQILEATR

KKVRNLSVGWVTYLMYISHHGVLFALSLNGMKVPIPKTETEKPLFNDNVNITELHTDESA

NQTKSEEMFMTLKFLPFGDWPFVDTRDSPAYELVYFLQATGAIVHAWAHAATDVLFLAI

STLVCGQLEILILTLSKVGKSNNYKEFSSCIRHHQQILSIAEVINDIYSPIMLVQFFNTMMAL

CTFAFEASKMEGGGNQIWVQADFFIATALQVYLFCSVGSRLTTLSLEVADSCYESDWVNK

PKFWKKSVEIVIMRAQKPLKLVGGPFYVISKETFLALIQLSFSYYTVLRNVQEEKE

>BgerOR77F

MTCSRQIHDRFGLYLVFLRMVGIPVFMKKSKLYFAYEIVANICIYTTSLACWLQILEEKHDL

KEMMATLRIAMSMVLVNILHFYMRFYLKAFEELLQITDTFTWEELPARSPTTGKLTAVG

WIDALKKFMKYTLIYVLIFHCIQTAYRMWYVENSMVFRAWYPYNYTVSPAYELTNFSQAI

SSVCGATTVLAFPGLYSTMVAIGRCQFDKIRIMLQAIYSQESSASDAQNQLKECIVLHQQVL

EYLRKIEDVLSNCLGAVLFLQMTDLCVLAFSLIISWGDYADMSQSFFIYLVWMTNTFIICW

SANELSDAADSVKGSAYEVDWIGAPIPFQRSILLMITRTNQSFVLTAGKFVPVNNETMMNI

FKESLSLFMFLLEMKDKRGE

>BgerOR78P

MQTDNLLIQKRFGLYLLYFQVSGIRICMKEKSKLYFAYEIIAIICFYGTFLACCIHILDAMDDL

KKMMASLRIAMVMGLIIVQHLYIMIHVYTLYIDWMGNIFIICWCANELSDAAERVKVSAYE

VDWIGALIPFQRSILLMITRTNQSFVLTAGKFVPVNNETMMNIFKESLSLFMFLLZMRNKR

HEQIVILCYGMF

>BgerOR79I

MTTGSPLLESRFGLYFVYLRLAGIPIYMKEKSRYYLIYQSFVSIVFYGTCLGFCIQILEDKHDL

KKMIASVQVSMASVFVAVLSFQIRIDLKSLEELLQLTDNFSWEEMPLRNPVSGDFTAKGWI

CVIRMVIKKTFVGADIIYITLSLLRMLYIQDSMVLPVWYPYNHTLSPIYELTNLSQNWGDY

ADMAQSAVMYVLITATSFLVCWPGTELTDKSESVKDAAYSSEWVGAPITFQKSILLIMTRA

NSEFTFTAGKFVPVNNSTLMNVLNESMSLFMFLMNVQEKQIAV

>BgerOR80

MKFNIGSPLVHNRFGLYLLCLRIAGIPVFMKEKSKLYILYEIFVNVCYHGMALACCIHVIEVK

DDLKEMLATLRIAMSVAMVSVLHIFIRIRVKEFEELLQLTDMFTWEEVPARNPETGKLTS

VGWMDMIRKIVKHALISTLIFHSTQSLYRLLSAQDSLVFHAWYPYNYTTSPGKELTNISQA

LASVFGMATIFAFPGLYATVVAIGCCQFHKLKMMMQVIFQKENSVDTENQLKKCIVLHQN

ILEYMSKMEEVLNNILGALLFTLMTNICVVAFSVIISWGDYVDMAQSIFIYVNWMTQVFIIC

WSANELSGAAEDAKRTACETDWIGAPVYIQRSILLMMTRANQPFVLTAGKFVPVNNETM

MNIFNESLSLFMFLLEMKDKHDVEINA

>BgerOR81P

MGNESQNGNPLIKNRFGLYLIYLQIAGIPIFMKEKSKIYFAYEIFVNICIYGTLLACWIQLLEV

KHDLKAMMATLRIAMAMGLAVLIHFYIRIHRLKFEELIQLTNDFTWEELPTRSPTTGNLT

AAGWMDIIRKVIKFSVICVLIFHAIQSMYRICTQGSLVFYAWYPFNYTVSSSYELTNLSQVL

VSVVLAPTFFAFPGIYLTTVAIGCSQFDKLKKMLKSINHKQSSLTDSKDELKKCVVLHQQIL

HPKZLLGCCSLLIMIIICLNWGDYEDMAQSFVIYINMMTTISTVCWPANELSAENVKLAAY

EVCWIGAPISFQKSILLMITRTNQPFFLTAGIFVPVNNVTMMNILNESLSLFMFLLQMKVT

HDEQD

>BgerOR82F

MEKELHNGSPLIKNRFSLYLVYLRIAGIPIFMKEKSKLYFAYELIVNVCVYGTLMACCIQLLE

FKHDLKVMMTTLRVAMSIVLGTLVHFYIRIHTLKFEELIQLTNDFTWEELPTRSPTTGNLT

AAGWIDIIRKVIKFTVNCILIFHTIQSLYRICTQGSLVFYAWYPFDYTVSPAYELTNLSQALV

SVVVTYGFCAFQGIYLTIVAIGCSQFDKLKMKLKSINLKQNSVTDSKEELKKCVVLHQKILR

YLIKMEEVLSNCLGAVLFLMMINICLDSFSVIIWGDYADMAQSFVIYTVLMATIFTICWPAN

ELSVAAENVKLAAYEGCWIGAPISFQKSILLMITRTNQPFFLTAGKFVPVNNATMMNILNE

SLSLFMFLLQMKVKHDEQN

>BgerOR83P

TGNNANVDSTLTQNRFGLYLVCLRIAAIPVFMKEKSKLYFVYEIFVTICFYGTLLSCCIHILE

TIDNIKEMMATLRVAMALGIVAVMRILIRXHTQGFEELLQLTDSFMWEELPHRNPKTGNL

SAVGWIATIKKISKYGIIFSCFFHFTQSFYRILXQDLVMFPAWYPYNYTVSPAQELTNFSQS

VASVLAIATIFGFFSLYSTIVAMGCSQFDKFKIMMQAICQKENSINTQNQLRTCIVLHQHVL

RYLSKMEEVLNISLGCILFLEMAILCIVAFAVIINWGDYADMAQSLFIYINSMIHIFIICWSAN

ELSDAAESAKLAAYEVDWIGTPISFQRSILLIISRTSKPFILTAGKFLPVNNATMMNIFNESL

SLFMFLLHMKDKHDEGIKT

>BgerOR84

MERDLTRTRFNIIIFCMRISGIPIFIERKTKMYLAYEILVYVCGCSSFIACWLDVLLNQENLK

EFLASVRVSIQILISAWILFYMKFHVKSVENLLRFTEQFTWEELPQKDPENGKITSAGVALII

QKMVKFSLIAILTFHFIQSTYRMLTKHEMIYLSWYPYDFKASPAYELTNLSQVLASIVAATS

LHFYFGFYGVTVSIACSQFYKLKLALLNIRDKQEPSTEELTNCIRHHQIILMFLDEIELAFNR

SIFGILFVEMTVACLCAFSVTTNWDVYADLLQALLILAISMGGISMLCWGGNILTESAESVK

EAAYSVDWVEAPETFKRSLLLMITRTNKEFTLTAGKFIPVNNKTMMNIFNEICSLILFLLE

MKNRYIQVD

>BgerOR85

MEGDLTHSRFKIIIFLMRLSGLPVFMERKTKIFLAYEILIELCGYSTFIGSWLNLFLNTENFK

ERLVSVHLPLQLLIAAWIHLDMRLHVKTIESLLRFTEQFTWEELPQRDPENGDITSSGVAL

ILPKMVKYSSLGLLSFHLLQSTYRMLTKHEMIYIAWYPYDFKASPGYELTNLSQVFASIQSA

AIFCSYLGYYSLSVSVACSQFYKLNLDLQKMNAKQDFSTEELNNCIKHHQTILVFLDEIELA

FNKSICGVLLVEMTVSCLCAFSITSGWGEYADLVQALAVFVFNMAGISMLCWAGNKLTES

AENVKEAVYSIDWVGTTETFKRSVLLMITRTNKQFTLTAGKFIPVNNETMLSIYKETWTLI

LFLLEMKNKYVQVD

>BgerOR86

MEGDLMHSRFNIIIFCMRVSGLPVFMERKAKIYLAYEILIELCGFSTFIAFWLDVFLNTENL

KEFLASVHLPIQSLIVAWIHLDMKLNVKTIESLLRFTEQFTWEELPQKDPENGDITPTGVT

LKLPKILKHSLIGVLSFHFIQSIYRMLTKHEMSFVSWYPYDFMASPAYELTNLSQVVSSIQY

AAIFWSYLGLYSLSVSVSCSQFYKLNLGLRKMNAKQGFSTEELNKCIKHHQTILAFLDEIEL

AFNKSICGVLLVEMTVACVCAFSITSNVEYADLLQAIVLFSLNMATISILCWAGNKLTESAE

SVKEAVYSTDWVEATESFKRSVLLMITRTNKQFTLTAGKSIPVNNETMMNIYKQTWTLIL

FLLEMKNKYVEAD

>BgerOR87

MEGDLTYSRFKIIIFYMRLSGLPVFMERKTKIYLAYEILIELCGFSTFIASWLEVLLNTEDFN

KFLTSVHQPIQFLIIAWIHLDMKFHVKTIESLLRFTEQFTWEQLPQRDPESGDITPTGVTLK

LPKILNHSLIGALSFHFIQSIYRMLTKHEMSFVSWYPYDFMASPAYELTNLFQVFASIQYVA

IFWLYVGLYSLSVSISCSQFYKVNLDLLKINAKQDFSTEELNNCIKHHQTILVYLDKIELAFN

TSICGILLFQMTISCLCAFSITSGWGEYADLLQAIVLLAINMTGISILCWAGNKLTESAESVK

EAVYSTDWVEATGSFKRSVLLMITRTNKQFTLTAAKFIPVNNETMMNIFKEIWTLILFLLE

MKNKYVEAD

>BgerOR88

MEVSSRTKSRFDLYIFLLRAAGIPFFMKQKSFLFLAYELFMYASTIITLVSTWICVLEGKQDI

KVNMATLRVSMGALVIIPFNFCFRFQMKDFEQLTKMTELFTWEELPTKDPDSGEITSAGL

VAPLRKLVKYSFIGGITGHTFQCLYRIIWNHELIFVIWSPYDWQVSPAYEITNVMQMAGSL

MVFCSLCGYLGLYCTLVAVACSQFDKLKMNMLQVYHGEEETRDKRLNACIKHHQLVLQF

LDDMENALTAGMCIALLVLMTALCVIAFSAVTSVGDPIDMIQIIMLYIIWMSAICIICWFGN

ELTDKADSVREAAYDGEWIGKPISFQRSLLIIMTRCNKSFNLTAGKFVLLNNETMMNILKE

TLSLFMFLLEMKDKSIEEGM

>BgerOR89

MEENFPRTKKRFSLYVFFLRAAGVPIFMKRKWKAYIAYEIILYSCSVALLITSWLRVVNER

DNIREMMATMRIAAGMLVIVPFNFCVRFQLKKFENVIEMTEGFTWEEMPFKDPIMGTLT

SAGMLPLIWKIIKYGFMTMGYGHLIQSFYRIIVNHELMFRTWCPIDCHASIVFEVTNISHIM

TSILAACSFFGYMGIYCSLVAIGCSQFDKLKMNMLSIYHTEQYNSNMDYHLQACVRHHQQI

FQFIREMENVFTIGMCLTLLVLMTSTCIAAFSAVISRGEVEDVVQIIMLYVAWMTSICVICW

TANELSDKAESVKNAAYEGEWIGTSISFQRSVLLIITRSNKIFNLTAGKFVLVNNETMMNIL

NETLSLFMFLLNMKDKTVE

>BgerOR90

MGTKAQSESLLTITRFRFNILFMRVGGIPIFMQMSIGYFMFVMVWYFCTFVTLAGVWLDA

FLGHHDLKDILASLRMAIAFTVTVWVHFCLRLNVKSIEQLFRCTEVFSWEEMPSKDPSTG

QLTSLGYWQWFQKGLKYMVIVNWFMHLVPCCHRIFIEHRMMLPVWLPIDLKASPAFELT

NLCQFLGTVQAGASFYSLLGLYSTTMFIVYCQVDKLKMSFKNVKSEPSELNQNEIELNKCII

HHQQILEYLPKMESTLNICIGGVLFLEMACCCTCAFSAVISWGDISDMAQAMLIYFIYMTSI

CCVCWAGEQLSNRVESVRDVVYEGHWIEAPISFQKSIMLIITRTNARFNLTAGKFVPVNNK

TMMNILQQTLSLFMFLLEIKDKNADEKNTINRRG

>BgerOR91

MKDDIDDNEDFDIYMKICKVLGYAPSNSFLYRIYSFCVMFLGNACTLSILIDMWLYLGDLE

HVMMSARIGLPLSISTCIDIFIKLRNYDMREVIKHSTNFTTIDSGTGALIPKFRYLVTRGFPL

AYSIHCLFTIITIITKDGRPLAMNSWFPYDTSYTPVYQFTVIAQIVATGIHTYRFVVFLGIYFT

LVMIACSQLEQLRALILGNMKNSQIELNQCIRYHQRILEFINHMEDVFNITLLSQLLFIMGS

VCFSAFSIVNCNGNTELLGEAIVTIVVMLEILFIYCWSGEQLMQKSKELEEAIWESNWVGA

PVVFQQCVLFVGAASSKELKFTAGKFVPMSNNTMLNILQSCYSYLMCLMSMSSE

>BgerOR92F

MADDVATSALELYLKILNYIGYSSSNSLVWSVYNFGVIFLAESSVIGVLVDMWLYLGDMEH

LMVTARIAFPLSISTCINVFLMFRGSALKEVINHSSLFITSDYGLGALIPKFRNMATKGMTII

FLLHSVFTIITITTREGRPLALNSWFPFNATRRPIYELVNIVQIISTGIHTARIVAYLGIYFTLV

ITACSQLEQLCELLMKMENTQSELNYCVRRHQMILKLVRHIEDVFNLHLLSHFLLLMGSM

CFSSIAIVMGKDNTELLGEAVLTQGVMLSILFCYCWSGEQLRQKADAVALAAWETEWVGA

PIAFQRSMLFVISAASKEIIFTAGKFVSVSNVTLVKILQQTLSLITFLLSLSE

>BgerOR93P

MTLRRGLLKSKFLKRDRFAMATEKFRSRFKFLILLIQIGAVPLFMEKKSLYYKVYEVIALFM

GYSVLLVTLLDATFNNEDIQRTMGSLRIFFPCLMIIWLQTILRVRMHAVEDLFRCTDYFTW

EDLPTKTSDGRLTNAGLVPVTQKLVKGFIGFIWGIHSLQSLARIIFAHDMVFDSWYPFDVS

ETPIYEIANLIQLLGSVILQSIFAAFTGLFATLIQVACSQIEKLKIFLNNIDKEDMYRELLQCIR

HHQQVLKYMRNMEEAFSWCVAGIFLINIMSLCAIAFSAVQSWHDAVALIQIVAIYFSMISE

NWVMCIVGEELTQQASSVGEAVYAVDWIGQPVHIQRLIYFMIANANSGLKQLSAARFVPV

SKGTFVKFMNETMSLFMFLLQVQERGDG

>BgerOR94P

MEAEDINENKFHKRFKFLIFITRLGAVPLFMEKKPLYYKVYEVIALLMGYSCMVVTFLDAA

CNNEDIQRTMGSLRICIPSCMIIWLQTILVQKHAVEDLLHATDSFTWEDLPTQTLDGHLT

NAGLIPVTQKLLKCFIPFIWGMHTLQSLVRVIFAHDMVFDSWYPFDVTESPIYEIVNLIQLL

GSMVLQSIFTAFLGLFATVIEISCSQIEKLKISLKNIGEQDIHKELIQCIRHHQQVLKYVREIE

EAFSWCVAGIFLINIISLCVIAFSAVQSWHDAVALVQIVVIYITMLLQNWVMCSLGEELMQQ

MCIRDSHISFMIANTNSGLKQLSAARFVPVSKGTFMKFMNETMSLFMFLLQVQERDDGKL

IEKY

>BgerOR95

MKGRELSEKRFRKLFLMMRGAGIPLYMKNTSIFFKVYSYLVTVCMLCTIITIFIGTVTNSDC

LKRIMQNVRVSFAMLDMSVAYIFIRFFHDRIEYLISLTEEFTWDELPKSNEFRMTSWIERV

QTLGKYICIIVLSYHYIQSTVRIITLQEIVFDAWFPFDTSVSPTFEIIIFFQLLASVAIICIFAGYL

PLNCIFIAVACSQLENLAFELKSLKQNCEIEEQLRNWILYHQKTLRFVKAIEDMLSTYIGVQ

MIPLYLGPCLSAFSAITSWNDFTDQTQAFVAVSVFFCQASVLCWFGTQLTESAENVKRAA

WECDWIGAPIPMQKTILFIISRGNKDTEITAGKFVPLSNVTLLHMMNDCVSIFMFLLNVKE

RKERQHD

>BgerOR96

MTGSALSEKRFGVQFRLLRACGIPLFMKNPSIFFKLYSYLVVVCMTSLSITVLIGTVTNSDTL

KRFMQNARIFFGIFGVTVAFMFNRFFLDKVEYLINLTEQFTWEDLPKTESFRMTSWIPRV

QTAGKYTFIVIFIFHYVQSAVRVLTLEELVFDIWFPFDTSKSPTFEIIVFFQFIASLTVVAIFA

GYLPLNCALMAVACSQLEKLAVDLIELKQDHDFMLQLQNWIRCHQQALGYVKAMEDSLS

MYMGVQFIPLFLGPCIFAFSAITSWGDFTDQTQAFVAFTAFIGQACVLCWFGAQLTEAAEN

VKRAAWGCDWIGTPIPVQKTILFIIARGNTDVTITAGKFVPLSNVTLMHMINNCLSMFMF

LLNVKDRKEIA

>BgerOR97P

MKLIYTLVLFYFKLRMVRGEEGKSVTETRFKFLIQFMRVGGIPIFMETQTKLYKAYQLVLF

TCTITTIISSWLHLIYENTMNDALKIILLPIAFVFVAAYSIFIRFDIKSIEKLLKSTENFTWEE

MPSRDSKTGNLTAAGALGIGTIYFKRVILGLAIYQSLICVIQFFMRVMFFPSWYPYDACASL

AFELTFLSQLLGFVAQFVSYLSFFASYCLFVAIGCSQCDKINTSLLSLNHNHNSTSENHQEL

CKCIILHQQVIEYINKMEXHLYLCGALLFQVTTLGVSALAILINLDDRFAAVQALATYVTMSI

SAFLVCWPGTKLSGKVESVKTAVYDVDWIGTPISFQKSLLFITKMADKPFKLTGGKIKPIN

NETLMDILNNTYVMFTFLLNMQNKQSEKEV

>BgerOR98

MESQSTDLIMKRFKFIIYLWRFVGVPMFLRKPSMVYKIYQAVTWLLMVSFPIFNCLDAYE

NRDNVARAAISLRTAFPMCIMLMPHTAVSLNLELFETLFHKTDTLLWEDMPEKDPVTGQ

LTIAGWIPRTMTLVKCGIIGSIFAHAVQVTDRIRSHELTTYSWYPFDCYSSPAYEITNFVQVI

QALMAICSMYAFIGVYALHLEIACTQLDKLTSSLLAIRQTSDSQEDFKAIEQELNRSIVHHQ

QILEYMQALENAMNFSICTIFLFLMATLCIISFSAVKVLNDPVELEQVIHLNIVFTIYLFILCG

FGTLLTNKAQYIAEAAYGVDWVGTPLSIQSSILIIITQSNKEFTLTAGKFVPVSNKTMLSILS

EAWSLFMFLLQVQDPEANN

>BgerOR99NP

FYRTDVVNVLILTNSFTWDEKEAKDPDTGGLSIATWIPRIKKITRNLFMTMWALHASQTI

LRIIFSDKPPLTLRAWYPFDTTSNPGYGVAMFLQLYSSLVLSAYMYGFPPFYATLVCIACSQ

LEKLRINLSRVNQDQELLNTSIRHHZKIIEYMKAMEVAFAPCLLGWFLSITAGLCISAFTAV

VSLENFSLLMQSVLIYTVLIANVFAFCWFGSELTDQAQKVSDAAFGMDWLGSQIPFQRCIV

FMISQANKEFKLTAGKLMPVNKITMKNMIEQSVSFFMFLVQVKDPKD

>BgerOR100N

FYRTDVVNVLKLTNSFTWDDKEAKDPDTGGSSIAAWIPRIKKFAFNLFIIIWIVHVSQTAL

RIIFSDERHLTVRAWYPFDTTSNPGYSVAFCHQLYASLVLSIYMHCFPPFYATLVCIACSQL

EKLRVNLSRVNQDQELLNACIRHHQKIIEYMNAIEVVFAPCLLGWFLLITTGLCISAFTAVV

SLGNFTWFLQALMIYTLLLANVLEFCWFGSELTDQAQKVSDAAFGMDWLGSSIPFQRCIV

FMIAQANKEFKLTAGKLMPVNKITMKNMIEQSVSFFMFLVQVKDPKD

>BgerOR101N

MMVDACMHLGDLGHIIENVATLLPHLCGIWIDVFLRFRRPAIERLIYHTDEFVWEDWPAR

DEITGSLTMAGLFPRIQKVIRVLVLVIWGPHGVYMVYRGIMDRELLGFNAWFPFDTFSSPT

HEIVILIQVYCSFALTTNFLANLGLYASLLTVACTQLTKVQNGLIQLGSCTFMDLIKVQFFCR

SENILCLGKQYFRDCSLGNVGALYEIIVVFNGQMMLLAAYCLFGTELSTQAEEVKFAAYSC

DWTGCSLSEQKSLMFIMAVASKEFIITAGGVIPVNRETMLAVMNQAFSYTMFLLNFKDT

>BgerOR102

MSTKDVMGDARKRFPIFRRLYKLSGNAIFGDGKTILYKTYSYISRVVVYLNWLAIVVDTIFN

LNNLERLIENVSVILPHLCSVWLDIFLQFRKSSMEKILMETNMFAWEDYPLRDEVTGWPT

LRFVIVNVPKICFCNVWFTVISHSSYMVYRGVTSRDILGFNAWFPFDSTLSPMHELVIAMQ

IYYSFMMTAHFYNNVAVYSTIVSIGCIQLNNLLKDIHSLQLDDLDDRKKKTKFKECVRYHQ

KILNYLGIMQDSLNVLMFGHFLIIIIGMAFAAFSAVMSWQNLGAMFQLIVVFFGQMILLAV

YCILGTHISTLTEDVRFAIYESDWIGSPVPTQKSMVFMMAAANNEFKLSAGGFIPVTRETM

LAMLNQMFSYTMFLLNFKDEEE

>BgerOR103

MALYRTRHVSKYVEDYYGFFISWFWLSATPIIKYNRLSLYGIASILLMINAYVCVATIIIDIFA

VPHDLQYMVENTRVAVPASNALWFHFIISFHKSEITELMAFQWEGFETTVLGPMTLLLPR

IQPMTVKTNIVFFGIHSIYSFLRVILTKTRQLAVNSYWGFDVTPSPIYQLIFVAQYCMEWICF

MLFFGFTGFYAYTVATACSHLETLREDLIRTKESSDIKKHLQKCIRHHQKILAYVGLVEKVS

SPVLLGQFLLILGGMCLSAFSAAMSWRSPLHIAQAFLVYSSFVAQLFVYCWFGSELSDELQL

VSEAAYNSDWVGIPLSEQRSIQFIIVAANKEVLLTAGKFVPATRKTMQNVINQTISFLMFLI

NVSNDDVQV

>BgerOR104NF

KEIFQFLCFSFFYVIISTTSERNNYGGKFGTDPRFQILKRLFKIAGTPLFPEKESNLYILYRNV

VVVSGYLTLLTTFIGIVQNITDLEYVLGAARVSFVMINLIWMHFFISFNIQKVRNLLNMIGH

FTWSDLPLHDHEGSISMAGWIPKIQNLLWKFNFCDWGFHCLYLVLRGVSSGSYHPLFFDA

WSPFNTENIMGYAVVLLIQGFGSVMIGTSLFAVMGLYISSVAVACTQLQKIQAALVNIKQK

DETLLNDELILCIRHHQQVLRYIKLLEETFNPVLLGPFMSVVAALCFTAYAAITIAGRFVEII

QIFLISCAMVFQIQAFCWFSTELTIETVKIRDAAWHTDWVGAPSSFQRSVLFMIAVSKEFT

LTAGKILPVSRRTFMMVVNETYTYLMLLLHFVDTSTQNITYKEL

>BgerOR105I

MDLSETDKRFKTLQNQFVRVGLPLWHIEKRFYNFYKICIIFCGNTTFIGAVINLIINIDDKSY

ILENIQMNFSMFNCIWIHLILSYKINELRNILKIVEEFTWSEESSDFMSRWIARAQNFSDKG

SVLAIGCFNLYFVLRNVLSEGRKVALETWAPVDLRENLPAYIITVIMQGIACIMYGNIVFAG

VILYTVLVAVACTQFHKLHSALLNIRTHEISESEMSHQLAKCVKHHQRIYEFVKGLEDTFNI

AILGPVFIVAAILCFSAFTAMKTGRVGDVAWESDWIGAPIPYQKSISIIIAASKEFVLTAGKII

PITRHTFMTIMNQTYSLLMFLLKF

>BgerOR106

MVELLKLQRSILGGVCFLGSKGKWYIYLGVSCWLLVLMAQLYGVHKFWGNIDAMAEGIGV

ILMLGLLIVQAAHCLKHSDQLQHIIDFLENLFNQFENEEcREIVLRTQALTNKVIKSLVPIFV

TDVSFWIFPPFIRYYFDEDKHGRPRADNEPFPYFCFIMDFPFDATHSPIFEVVFLVQLTCTT

VLALFDIALLTIVFSTILFTAAYLKALATMLRKLVDFEPGDKEIEDPEAYLNNCIRLHQQLLS

FSVDLQQYLSPILIFFLKPFELFLCIAVYEAVQMTDKVSTFVVSAIALLSGISVLCAFGEHLKE

QSELLEKEVTACRWYLHSTRFKRHHLLLLAQCQRTIRLKVGFFYPLTLETFIKILNTTYAYF

NLLQQLR

>BgerOR107

MAELLRIQKTILTYSSFLGSRSKWHVYLVVCCWLFLTTGQVYGIRSFWGNIDAIAEIVGIGV

LCIFLIIQAFHCLRNKDQLQHIIDSLENLFKQFENDEcRQIVEKTDKLNNKITKMLVPIYVT

DTVLWFIIPFIRYYFDVDKHERPRADNEPFPYFCFIVDFPFDATHSPIYEFVILVQSTITAILT

LYNTAFLTIVFSTILFTASYFKALAAMVRKLVDFEPGDKEIEDPEAYLNNCIRLHQQVLSLV

SDLQEYISPILVFFIKPYEVFLCIAVYETVLMTDKASAFIEGTFGLLFCIAVFCSFGEHLKKQS

ELLEAEVTACRWYVQSTRFKRSHMILLAQCQRTVCLQVGLFYPLTLETFIKILNSTYAYFN

LLQQLR

>BgerOR108P

MAELLRIQKIIFTYTCCIGSIGKWHFYLAVCCWLFVFMGQIYGIHSFWGNIDVVAEIIGITVL

YIFLIIQAFYCLMHKDQFQHIINTLENLYIQFENEEcREIVMKTNQFNNKMTKILVPIFVTD

VTLWIITPFIRYYFDENKHERERADNEPFPZFSYIVDFPFDATHSPIYEFVFLVQFTMTSIM

SLYNIAFLTIVISTILFTASYFKALAAMVRKLVDFEPEDKEIEDPEAYLNNCIRLHQQLLSLL

RDLQEYISPILVFFIKPYELFLCISVYEAALMTDKVTTYIEGAVGMLFCICIFCSFGEHLKKQA

EVAACRWYVQSSRFKRSHMILLAQCQRTVCLQVGLFYPLTLETFIKILNSTYAYFNLLQQIK

>BgerOR109

MAEILRLQKTILTYTSFLGSRSKWHIYLAVGCWLFVIMGQVYGIRSFWGNIDAIAEIIGVAV

LCIFLIMQTFHCLRHKDQLQHIIDSLENLFKQFENEECHQIVMKTTQFNNKMTKMLVPIFA

TDMTLWIIIPFIRYYFDEDKHERERADNEPFPYFCFIMDFPFDATHSPIFEFVFLVQFTITA

VLTLYNIAFLTIVFSTILFTASYFKALAAMLRKLVDFEPGDKEIEDPEAYLNNCIRLHQQLLS

LVSDLQEYISPILVFFIKPYEVFLCISVYEAALMTEKVSAFIEGAVGALFCIAIFCNIGEHLKKQ

SELLEAEVTACRWYVQSTRFKRSHMILLAQCQRTVCLQVGLFYPLTLETFIKILNSTYAYFN

LLQQLQ

>BgerOR110

MAELLRLQKTIFTYTCCIGSNSKWHFYLAIGCWLILFMGQVYGIRSFWGNVDAIAEIVGVA

VLCTFLFIQAFHCLRHKDQLQHIIDSLENLFKQFENEEcRQIVMNTNQFNNKMIKILVPIFV

TDVTLWVIIPFIRYYFDEDKHERKRDDNEPFPYFSYIVDFPFDATHSPIYEFVFLVQFTMTS

IMSLYNIAFLTIVISTILFTASYFKALAAMLRKLSDFEPGDKEIEDPEAYLNNCIRFHQQLLS

LMSDLQDYIGPILVFFVKPYEVFLCISVYEAALMTEKVSAFIEGALGMLFCIAIFCSFGEHLK

KQSELLEAEVTACRWYVQSTRFKRSHMILLAQCQRTVCLQVGLFYPLTLETFIKILNSTYA

YFNLLQQLQ

>BgerOR111C

MAQLLRVQRKILSYLCFFDFKYVPYFLLGFCSWIFVFSGQLYGLYQFYGNMDVIVEGLGVTL

YFIMLIMQALYCMTHQSQLARIVNFFEYKYDELENEEARAIMMKTSNLCNKLCLALIPTFI

VDIIVWSAIPLAQYYDKSSKCHQERADDEPYTCFIYVLVFPFDFTYSPAYECAFLIQMICTFF

VMFINLGVMVLVFTTMLYCAAYFKVLATMVRELGDSDQFDDPNEEVVKTCIDNPEAYLD

KCIHLHQQLLHFLKELQYFIGPVLAMFLKPYELLLCIAVYEAALMNDKSTIFILTATVMLIF

VYIVCAFGEHLQQQGRLVEKEVLACAWYDQSRRFKRHRLIFMAQAQRDVLVHVGVFYPL

TLESFIM

>BgerOR112

MKRSLGVDIAIHLKIMFWVCLWVPSNTYIAKLLYGSFVLFTVVFVMSLLLAQLLYLVIEHF

DLILFVNSLTTLTSNIKILLIMTRFRFMKEELQDLIKNFNALRSNRKSVSESENKILETAEKE

SRVLTNGLLSGFITLFILMVAKPLFVQYTSTHHSNSTDLCPERSFIMLSWYPWGTCSTTIYA

AIYVSQIYALFMIIMQIAVYQTLNTSILIHIAAEVDIIYDKFISVLNQNNRKISEERNNKFPNL

DIDTNRSKNNEKLKQCYALNKDIVCAETNSTALKNNHIEGIVGILDNVSVAYECVKDPISN

MFSNDYDKSEEEGEQEEEEEVYNRLIEIVKHHQSVFRFSEEVNNMFSPFYLFQFSTVIIGLC

FASFLASMPSLDSETRMQFIIFTFLLMAQLLLPCWYGQRVTDQSERIRDAVYGCRWYDKS

PRFKKAVQIIIMRCQRPICFSVGGFAVISRETWLSLINFTYSLLAVLRQMDEDTLQEN

>BgerOR113P

MKRSLGVDLAIHLKVMFWLALWVPSESNIAKFLYGIFTLFTGVMACVFLLFQLLNVAYGN

LNLILLVHSLAIIAPSIRVVLIFIKLTFTRKDLKNLVQNFNTLRSNWKSSSETENKILESAEKE

SRIITNGLLSVFIILMILMSVKPFVFQYTSMNNTNLTDVHPERSFAVPSWYPWDFSSTTSM

FAVTYISHIYGIYLLTXAIFQAFTSAILIHIAAEFKIIYEKFISVLNQNKHAEESKIDFSRASNST

FDIKRNQNKNTVRKRRNEIREDISSEINSRNSEYANASVSNISSINYEKPENEDENEAYERL

IEVVKHHQNVLNFCEEFDSIFSSLYLLQFSSIIVCLCISCFIASLSYVDSGTRMQFINITGTAM

AQLLLTCWYGQRVTDQSELIADAVYGCGWYNASPRFKKAVHIIIIRCQRPVCFSVGGFAVIS

RETWLSILNFTYSLLAVLRQMDEDKLPEN

>BgerOR114

MKRSLGVDIAFHLKVMFWVALWVPSDSNIAKFLYGIFTLFTGAMVCGFLLSQLLNIALGHF

NLILFVHSLATIAPCVKVLLILIKFTFTKEDVENLIKNFNTLRSNWRSSSETENKILDSAEKE

SRLMTNGLLSGYVILMIFMSVKPLVFQDTYMNNTNLTDIHPERSFVVLSWYPWDTRSTT

MYAVTYISQIYALYVVIIQVAVFQTFNTAILIHIAAEFKLIYQKFTSILNQNTYNHAEESKNIF

SHASNSTCDVKRSQNKNTIRKRRCEIRKGIRSEIKSKNHVKEGGEKPYSEYANDSGSNIFSI

NYDKPENEAYERLIEIVKHHQNVLRFCEEFGTIFRSLYLFQFSTVIFGLCFSCFLASLPSVDS

ETRMQFIIFTGLLMAQLLLPCWYGQRVTDQAELIRDAVYSCRWYDESARFKKAVHIIIMVC

QRPISFSIGGFAAISRETWLSLINFTYSILAVLRHMDVDKFAEN

>BgerOR115P

MKRSLGVDIAIHLKIMFWAGLWVPSNSSIAKILYGFFILVTGVMAAALLLSQLLNLGLGDF

NLILFANSLATITSNIKILIILIRFTYMKKELQNLINNFITLRTNWKSYSETENKILENAGKES

QKLTKVILSGYAVLMTIMSVQPLVSQDNYINQTNSTDIFPERLFVVPSWYPWDTTSTTMY

AVTYISQIYAFFMVCMQIILYLTLTSSIFIQIAAEVDIIYEKFIAVLNPNIYKNSEEZKYKFSQA

SNSTVQNKRCHNNNEKLKRRYALNEGDVSAETNSSVLNENHLEETHEKLDFTSVSFEYA

NDPISNISLNSYDRSEEEEEEEEEEEEEEEEEEEEEEEEEEEDETYDDYDDEAYRRLIEIVK

HHQYIFSFSKEVDTMFRPIYLFQFASITFGLCFSCFLAASPSSDSETKMQFIVFTGLLMAQLL

LPCWYGQRVTDQSERIQDAVYGCRWYEESTKFKKAVQIIIMRCQRPICFSVGGFAYISRET

WLSIIHFTYSILLVLRNMDEEKLSAN

>BgerOR116P

MNTFLAVESVSTFNFNMNTSLGVDVGIHLKIMYWGALWVPSKNTSXKFIYGLFILITGVTV

TFLLFCQISYLIFKHFDLILFARSLTTLTSFIKILLIMTRFTFMKQELQNLIDNFVTLRSSWKT

YSESEIKILEIAGKEXTKLTIVILSGYLILMTIMVTKPIIFQDISTNLANTTFMHIERSYXLSWY

PWDTTTMMYALTYISQVYGYFMLTLQIVVYLTLITSILIQITAEVDIVHNTFTSVLNPDNCK

NLEESENNFTEVTRFTVDIRGDQRKKRNSNKNIVKIKILMLQSQIRQLLKGILGXEYGNGS

MSHSSSMSYNKSEVEKKEEIEDETYKRLIEIIKHHQDVFSFSEEVDTMFNPLYLFQFSSVIF

GLCFSCFLASSPSSDSETKTQFIVIAVLMVVLLLLPCWCGQRSERIGDAVYGSGWYDESPRF

KKAVRIIITRCQRPISFSIGGFSDISRETWLSITNFTYSLLLVLQNFDKEKLASN

>BgerOR117F

MLISGNISMTSEELPADSKCNKFRTSGIQLKINRKILWAMGLWYPRKARSSQYRDYWLFS

WFMTFGLFAHISTEIVAMYVYSNDIRIMLNIFCTMISGLGAFYKALYTVYRKYQMQDLVNK

LEGSTFAFDHQIGEEQQRIIRSACRLSNFITALFEILCVFIVTLWMIAPMVFEDPTSPLEQPL

PLPASFPWDLRNTMGYSAAYIFQLESLAVTLEKLVESLNSEHKRISIRDISTMNRSSERKIQ

EKAERRETSQERNRHSGELKSHQDTYAISENLTNEDLIQEFTERELESYQKLKECIKHHNE

ILSFAEEVQTHFGPVMLFQCGSSTLILCFVAFLLTESSGDIGTLIKCIMYLVVGTFELWLYCV

FGDRVIELSGNISNAAYGSEWIVMSQRFKKALTMMIMRAQKPIIFKGGFLYTVSLETFMSLI

NASYTYFTVLQQAKE

>BgerOR118

MVTGSPTASSLEVNQESNKKWLRAKQFHLSFLNIIGILPSQSISSNKYKLFIYNIFCVINMSI

KFPIAILVFCNISHAWGDFSDVSDTVHITNAVSLDGVMFTYLFFKRKELENIFNQMQSKFM

KITEQIVSPQIYQTVVDRTTRRCWILSTIFITWLSFMIAVWTVLPFSLLFVPHLMEVGSTLK

NETSYVFMLPMWWPEELKMTPIYEIVFVIQLFETFYCLTIIAAFGTVYLYIVNGIVMRFEIL

GYCIEHTEANIRYWLENASEQSGNIENKLTNSDNQIKFEERERLQQLEKGIHEVDNNLQQ

DFEIEVSLINQLMDTNKTREEEEEEEEEMFRIYLLQWIKIHQDLLDYCEEIKIFFNVLLMAF

FTTISLNMIFLAFGLAQDSRNSIVAVTLSAIYAFGCPYLLCLYGDKLTEASLNIKDAAYSCEW

YTKSPVIKRMLQMIIMRAQSPVVLKAGWYSISLEQFSEIINTVYAYFNIMRENANKN

>BgerOR119

MKKGSDSLNTSIREGSCELDLMNLQQKCLILAAIEPSNEIKINFWKSILFRIYQIIIVSTYIPLL

VIQIFGCYHYRYDFLTLLDGIVPIGLASIGFFVPLSANWKIATELVRKFERDSVFIKAINQNN

TKNINLLNEAQRFTKFLTCTLLVCITLSAGIWFTQPHIFAVLESFTNKKENSNSTIIMDPLK

MYPLVIWIPYVDITATIPYMFISTLIGTCIFIVSTRGATFISYSVSIMIYTTTQFKMVATCMNE

IDNVDDNELEFTDNNIISNNNGLSKQVLNCPNNKSPDELKRTHEEHLDDVKKQIKRKKFN

VYKNIKLQDFEYEKCLLCPEEDEVKAITQLINSVKDHQQILKIINEVNDAFSSMFFMTIMLG

ALSLSLALFVVAVNPDFSNKLKNASAVMVLLIYGWIIFSNGEDVKEEGIKIHHTAYNLQWF

KHSTKFKKILQIIIMRSQKPCGIMLGPLMDMTIENYSNILNTAYSYFTLLVQFESKDSEK

>BgerOR120P

MKKENDSLNTIVREGACELDLMNLQQKCLILAAIEPSNEIKINFWKNILFRIYQIIIVSTYIPL

LFLNICGSYHYRNDLLTLLDGIVPIGLASIGYFVPLSAKWKIATELVRKFZRDSVFVKAINQN

NTKNINLLNEAQRFTTFLTRTLLVCTYISAVMWSIQPHILAVLESFTNSKKNLNSTIVDPLK

IYSHVIWIPHVDITANKPYMFITALIVTCAFVISTRAAAFISYSVSIMIYTSTQFKIVSTCLKEI

DNMDDNELEFMENNIISNNNDLSKQVLNWPNDEPPDEFKHFHEEHHDGVKKRIKRKKS

NVYKNVKLKDSEYEKCVLCPEEGEVKAITQVINCVKDHQQILKTIKEINDAFSSMFFVTIMI

GALSLSLALFQIAANPDFSNKLKNASSGMAILIAGWIIFSNGEDVKEEGINIHHTAYNLHWF

KHSTTFKKILQIIIMRSQKPCGIMLGPLMDMTIENFSHILNTAYTYFTLMVQFESKASEKW

DHTST

>BgerOR121P

MKKGSDSLNTNIREGSCELDLMNLQQKCLIFAAIEPSNEIKINFWKKILFRFYQIIIVATYIP

VFFFNICGCYHYRHDLLTLLDGIVPIGIASIGYFVPLSAKWKIATELVRKFERDSVFVKAINQ

NNTKNINLLNEAQRLTTFITRTLLFCTLFSSGIWCIQPQIFAVLESFTNSKENSNSTIVDPLK

MYPLVIWIPYVDITAIKPYMFITTLIVSXCIFIVSARAAAFISYSVSIMIYTNTQFRIVATCMN

EIDNVDDNEPEFTENNIISNNNDLSNQVLNWRNNKPPDEFIHSDEEHLDGGKKRIKRKKS

NAYKNVKLKDSEYEECVLCPEEYEVKAIIQLINSVKDHQQILISIKEMNDAFSSMFFITILFA

VLSLSLALFVVAVNPDFSNKLKNASSVMAILIAGWIIFSNGEDVKEEGIKIHHTAYNLHWF

KHSTKFKKILQIIIMRSQKPCGIMLGPLMDMTIENYSNILNTAYSYFTLLLQFDS

>BgerOR122F

MKKGSDSLNTSIREGSCELDLMNLQQKCLILAAIEPSNEIKINFWKSILFRIYQIIIVSTYIPLL

VIQIFGCFHYRYDFLTLLDGIVPIGLASIGFFVPLTAKWKIATELVRKFERDSVFVKAINQNN

TKNINLLNKAQRFTKFLTRTLLVCTTFSAGIWFTQPHIFAVLESFTNSKENLNSTIVDPLKI

YPLVIWVPYVDITATIPYMFITTLIGTCIFIVSTRIAAFISYSVSILIYTSTQFKIVATCMNEIDN

VDDNEPEFMENNIISNNNDLSKQVLNSLNDKPPDEFKHSDEEHLDGGKKRIKRKKSNAYK

NVKLKDSEYEECVLCPEEDEVKAITQLINSVKDHQQILTIINEVNDAFSSMFFTTIMIGALSL

SLALFVVAVNPDFSGKLKNASAVMVTLIAGWIIFSNGEDVKEEGIKIHHTAYNLHWFKHST

KFKKILQIIIMRSQKPCGIMLGPLMDMTIENYSNILNTAYSYFTLLVQFESKDSEKWDHGST

>BgerOR123

MNTIKDSKIMREDNIELPFCKRLETTYEIELMGFQNRCLLYLGILPLKDTNEIWKIKLYRFY

QVLGYIMYIPVILMEIYGCFHYRNDLNVVIDAIVPIGLGCIGFFVPLTVNWDNVLNHFEKME

TKSAFIRIINDNNRKKIAILEEHYHWSRFFTKTFVLIANITGITWGLQVYLLEFLETLTGEN

HSNSTINEEFPALKLYPIVVHLPYVDMTDTVICIILNVIIMILIIMTCNRAGMTIVYFSTLLLY

HSTLFTLVAVSLEEVDTEDSINIESNFNTSHDIGQPVIRKENNRFQDSFENYVLQKTQCNN

LDIVNKRPRPIKSSMFNYSDNENQTEIANEEQEIRAIDLLKDAIKDHQEALQYVDRVNDLIS

SYLFSEISVGAVVLALALFESVVNPNFAAKAKHVASALAVVIGGWIVFRSGATVTDQGQKV

CEAACNTLWYKHSMKFRKLLYILIWKSQKPVIVVAGPLIEMTEKNFADVVNGSYSYFTLLL

QFESTKS

>DmelOR1a

MSKLIEVFLGNLWTQRFTFARMGLDLQPDKKGNVLRSPLLYCIMCLTTSFELCTVCAFMVQNRNQIVLCSEALMHGLQM

VSSLLKMAIFLAKSHDLVDLIQQIQSPFTEEDLVGTEWRSQNQRGQLMAAIYFMMCAGTSVSFLLMPVALTMLKYHSTG

EFAPVSSFRVLLPYDVTQPHVYAMDCCLMVFVLSFFCCSTTGVDTLYGWCALGVSLQYRRLGQQLKRIPSCFNPSRSDF

GLSGIFVEHARLLKIVQHFNYSFMEIAFVEVVIICGLYCSVICQYIMPHTNQNFAFLGFFSLVVTTQLCIYLFGAEQVR

LEAERFSRLLYEVIPWQNLPPKHRKLFLFPIERAQRETVLGAYFFELGRPLLVWIFRTAGSFTTLMNALYAKYETH

>DmelOR2a

MEKQEDFKLNTHSAVYYHWRVWELTGLMRPPGVSSLLYVVYSITVNLVVTVLFPLSLLARLLFTTNMAGLCENLTITIT

DIVANLKFANVYMVRKQLHEIRSLLRLMDARARLVGDPEEISALRKEVNIAQGTFRTFASIFVFGTTLSCVRVVVRPDR

ELLYPAWFGVDWMHSTRNYVLINIYQLFGLIVQAIQNCASDSYPPAFLCLLTGHMRALELRVRRIGCRTEKSNKGQTYE

AWREEVYQELIECIRDLARVHRLREIIQRVLSVPCMAQFVCSAAVQCTVAMHFLYVADDHDHTAMIISIVFFSAVTLEV

FVICYFGDRMRTQSEALCDAFYDCNWIEQLPKFKRELLFTLARTQRPSLIYAGNYIALSLETFEQVMRFTYSVFTLLLR

AK

>DmelOR7a

MAVSTRVATKQEVPESRRAFRNLFNCFYALGMQAPDGSRPTTSSTWQRIYACFSVVMYVWQLLLVPTFFVISYRYMGGM

EITQVLTSAQVAIDAVILPAKIVALAWNLPLLRRAEHHLAALDARCREQEEFQLILDAVRFCNYLVWFYQICYAIYSSS

TFVCAFLLGQPPYALYLPGLDWQRSQMQFCIQAWIEFLIMNWTCLHQASDDVYAVIYLYVVRIQVQLLARRVEKLGTDD

SGQVEIYPDERRQEEHCAELQRCIVDHQTMLQLLDCISPVISRTIFVQFLITAAIMGTTMINIFIFANTNTKIASIIYL

LAVTLQTAPCCYQATSLMLDNERLALAIFQCQWLGQSARFRKMLLYYLHRAQQPITLTAMKLFPINLATYFSIAKFSFS

LYTLIKGMNLGERFNRTN

>DmelOR9a

MSDKVKGKKQEEKDQSLRVQILVYRCMGIDLWSPTMANDRPWLTFVTMGPLFLFMVPMFLAAHEYITQVSLLSDTLGST

FASMLTLVKFLLFCYHRKEFVGLIYHIRAILAKEIEVWPDAREIIEVENQSDQMLSLTYTRCFGLAGIFAALKPFVGII

LSSIRGDEIHLELPHNGVYPYDLQVVMFYVPTYLWNVMASYSAVTMALCVDSLLFFFTYNVCAIFKIAKHRMIHLPAVG

GKEELEGLVQVLLLHQKGLQIADHIADKYRPLIFLQFFLSALQICFIGFQVADLFPNPQSLYFIAFVGSLLIALFIYSK

CGENIKSASLDFGNGLYETNWTDFSPPTKRALLIAAMRAQRPCQMKGYFFEASMATFSTIVRSAVSYIMMLRSFNA

>DmelOR10a

MSEWLRFLKRDQQLDVYFFAVPRLSLDIMGYWPGKTGDTWPWRSLIHFAILAIGVATELHAGMCFLDRQQITLALETLC

PAGTSAVTLLKMFLMLRFRQDLSIMWNRLRGLLFDPNWERPEQRDIRLKHSAMAARINFWPLSAGFFTCTTYNLKPILI

AMILYLQNRYEDFVWFTPFNMTMPKVLLNYPFFPLTYIFIAYTGYVTIFMFGGCDGFYFEFCAHLSALFEVLQAEIESM

FRPYTDHLELSPVQLYILEQKMRSVIIRHNAIIDLTRFFRDRYTIITLAHFVSAAMVIGFSMVNLLTLGNNGLGAMLYV

AYTVAALSQLLVYCYGGTLVAESSTGLCRAMFSCPWQLFKPKQRRLVQLLILRSQRPVSMAVPFFSPSLATFAAILQTS

GSIIALVKSFQ

>DmelOR13a

MFYSYPYKALSFPIQCVWLKLNGSWPLTESSRPWRSQSLLATAYIVWAWYVIASVGITISYQTAFLLNNLSDIIITTEN

CCTTFMGVLNFVRLIHLRLNQRKFRQLIENFSYEIWIPNSSKNNVAAEcRRRMVTFSIMTSLLACLIIMYCVLPLVEIF

FGPAFDAQNKPFPYKMIFPYDAQSSWIRYVMTYIFTSYAGICVVTTLFAEDTILGFFITYTCGQFHLLHQRIAGLFAGS

NAELAESIQLERLKRIVEKHNNIISFAKRLEDFFNPILLANLMISSVLICMVGFQIVTGKNMFIGDYVKFIIYISSALS

QLYVLCENGDALIKQSTLTAQILYECQWEGSDRIEIQSFTPTTKRIRNQIWFMILCSQQPVRITAFKFSTLSLQSFTAI

LSTSISYFTLLRSVYFDDEKKLD

>DmelOR19a

MDISKVDSTRALVNHWRIFRIMGIHPPGKRTFWGRHYTAYSMVWNVTFHICIWVSFSVNLLQSNSLETFCESLCVTMPH

TLYMLKLINVRRMRGQMISSHWLLRLLDKRLGCDDERQIIMAGIERAEFIFRTIFRGLACTVVLGIIYISASSEPTLMY

PTWIPWNWRDSTSAYLATAMLHTTALMANATLVLNLSSYPGTYLILVSVHTKALALRVSKLGYGAPLPAVRMQAILVGY

IHDHQIILRLFKSLERSLSMTCFLQFFSTACAQCTICYFLLFGNVGIMRFMNMLFLLVILTTETLLLCYTAELPCKEGE

SLLTAVYSCNWLSQSVNFRRLLLLMLARCQIPMILVSGVIVPISMKTFTVMIKGAYTMLTLLNEIRKTSLE

>DmelOR19b

MDISKVDSTRALVNHWRIFRIMGIHPPGKRTFWGRHYTAYSMVWNVTFHICIWVSFSVNLLQSNSLETFCESLCVTMPH

TLYMLKLINVRRMRGEMISSHWLLRLLDKRLGCADERQIIMAGIERAEFIFRTIFRGLACTVVLGIIYISASSEPTLMY

PTWIPWNWKDSTSAYLATAMLHTTALMANATLVLNLSSYPGTYLILVSVHTKALALRVSKLGYGAPLPAVRMQAILVGY

IHDHQIILRLFKSLERSLSMTCFLQFFSTACAQCTICYFLLFGNVGIMRFMNMLFLLVILTTETLLLCYTAELPCKEGE

SLLTAVYSCNWLSQSVNFRRLLLLMLARCQIPMILVSGVIVPISMKTFTVMIKGAYTMLTLLNEIRKTSLE

>DmelOR22a

MLSKFFPHIKEKPLSERVKSRDAFIYLDRVMWSFGWTEPENKRWILPYKLWLAFVNIVMLILLPISISIEYLHRFKTFS

AGEFLSSLEIGVNMYGSSFKCAFTLIGFKKRQEAKVLLDQLDKRCLSDKERSTVHRYVAMGNFFDILYHIFYSTFVVMN

FPYFLLERRHAWRMYFPYIDSDEQFYISSIAECFLMTEAIYMDLCTDVCPLISMLMARCHISLLKQRLRNLRSKPGRTE

DEYLEELTECIRDHRLLLDYVDALRPVFSGTIFVQFLLIGTVLGLSMINLMFFSTFWTGVATCLFMFDVSMETFPFCYL

CNMIIDDCQEMSNCLFQSDWTSADRRYKSTLVYFLHNLQQPITLTAGGVFPISMQTNLAMVKLAFSVVTVIKQFNLAER

FQ

>DmelOR22b

MLSQFFPHIKEKPLSERVKSRDAFVYLDRVMWSFGWTVPENKRWDLHYKLWSTFVTLLIFILLPISVSVEYIQRFKTFS

AGEFLSSIQIGVNMYGSSFKSYLTMMGYKKRQEAKMSLDELDKRCVCDEERTIVHRHVALGNFCYIFYHIAYTSFLISN

FLSFIMKRIHAWRMYFPYVDPEKQFYISSIAEVILRGWAVFMDLCTDVCPLISMVIARCHITLLKQRLRNLRSEPGRTE

DEYLKELADCVRDHRLILDYVDALRSVFSGTIFVQFLLIGIVLGLSMINIMFFSTLSTGVAVVLFMSCVSMQTFPFCYL

CNMIMDDCQEMADSLFQSDWTSADRRYKSTLVYFLHNLQQPIILTAGGVFPISMQTNLNMVKLAFTVVTIVKQFNLAEK

FQ

>DmelOR22c

MTDSGQPAIADHFYRIPRISGLIVGLWPQRIRGGGGRPWHAHLLFVFAFAMVVVGAVGEVSYGCVHLDNLVVALEAFCP

GTTKAVCVLKLWVFFRSNRRWAELVQRLRAILWESRRQEAQRMLVGLATTANRLSLLLLSSGTATNAAFTLQPLIMGLY

RWIVQLPGQTELPFNIILPSFAVQPGVFPLTYVLLTASGACTVFAFSFVDGFFICSCLYICGAFRLVQQDIRRIFADLH

GDSVDVFTEEMNAEVRHRLAQVVERHNAIIDFCTDLTRQFTVIVLMHFLSAAFVLCSTILDIMLNTSSLSGLTYICYII

AALTQLFLYCFGGNHVSESSAAVADVLYDMEWYKCDARTRKVILMILRRSQRAKTIAVPFFTPSLPALRSILSTAGSYI

TLLKTFL

>DmelOR23a

MKLSETLKIDYFRVQLNAWRICGALDLSEGRYWSWSMLLCILVYLPTPMLLRGVYSFEDPVENNFSLSLTVTSLSNLMK

FCMYVAQLTKMVEVQSLIGQLDARVSGESQSERHRNMTEHLLRMSKLFQITYAVVFIIAAVPFVFETELSLPMPMWFPF

DWKNSMVAYIGALVFQEIGYVFQIMQCFAADSFPPLVLYLISEQCQLLILRISEIGYGYKTLEENEQDLVNCIRDQNAL

YRLLDVTKSLVSYPMMVQFMVIGINIAITLFVLIFYVETLYDRIYYLCFLLGITVQTYPLCYYGTMVQESFAELHYAVF

CSNWVDQSASYRGHMLILAERTKRMQLLLAGNLVPIHLSTYVACWKGAYSFFTLMADRDGLGS

>DmelOR24a

MERHYFMVPKFALSLIGFYPEQKRTVLVKLWSFFNFFILTYGCYAEAYYGIHYIPINIATALDALCPVASSILSLVKMV

AIWWYQDELRSLIERVRFLTEQQKSKRKLGYKKRFYTLATQLTFLLLCCGFCTSTSYSVRHLIDNILRRTHGKDWIYET

PFKMMFPDLLLRLPLYPITYILVHWHGYITVVCFVGADGFFLGFCLYFTVLLLCLQDDVCDLLEVENIEKSPSEAEEAR

IVREMEKLVDRHNEVAELTERLSGVMVEITLAHFVTSSLIIGTSVVDILLFSGLGIIVYVVYTCAVGVEIFLYCLGGSH

IMEACSNLARSTFSSHWYGHSVRVQKMTLLMVARAQRVLTIKIPFFSPSLETLTSILRFTGSLIALAKSVI

>DmelOR30a

MELKSMDPVEMPIFGSTLKLMKFWSYLFVHNWRRYVAMTPYIIINCTQYVDIYLSTESLDFIIRNVYLAVLFTNTVVRG

VLLCVQRFSYERFINILKSFYIELLQSDDPIINILVKETTRLSVLISRINLLMGCCTCIGFVTYPIFGSERVLPYGMYL

PTIDEYKYASPYYEIFFVIQAIMAPMGCCMYIPYTNMVVTFTLFAILMCRVLQHKLRSLEKLKNEQVRGEIIWCIKYQL

KLSGFVDSMNALNTHLHLVEFLCFGAMLCVLLFSLIIAQTIAQTVIVIAYMVMIFANSVVLYYVANELYFQSFDIAIAA

YESNWMDFDVDTQKTLKFLIMRSQKPLAILVGGTYPMNLKMLQSLLNAIYSFFTLLRRVYG

>DmelOR33a

MDSRRKVRSENLYKTYWLYWRLLGVEGDYPFRRLVDFTITSFITILFPVHLILGMYKKPQIQVFRSLHFTSECLFCSYK

FFCFRWKLKEIKTIEGLLQDLDSRVESEEERNYFNQNPSRVARMLSKSYLVAAISAIITATVAGLFSTGRNLMYLGWFP

YDFQATAAIYWISFSYQAIGSSLLILENLANDSYPPITFCVVSGHVRLLIMRLSRIGHDVKLSSSENTRKLIEGIQDHR

KLMKIIRLLRSTLHLSQLGQFLSSGINISITLINILFFAENNFAMLYYAVFFAAMLIELFPSCYYGILMTMEFDKLPYA

IFSSNWLKMDKRYNRSLIILMQLTLVPVNIKAGGIVGIDMSAFFATVRMAYSFYTLALSFRV

>DmelOR33b

MDLKPRVIRSEDIYRTYWLYWHLLGLESNFFLNRLLDLVITIFVTIWYPIHLILGLFMERSLGDVCKGLPITAACFFAS

FKFICFRFKLSEIKEIEILFKELDQRALSREECEFFNQNTRREANFIWKSFIVAYGLSNISAIASVLFGGGHKLLYPAW

FPYDVQATELIFWLSVTYQIAGVSLAILQNLANDSYPPMTFCVVAGHVRLLAMRLSRIGQGPEETIYLTGKQLIESIED

HRKLMKIVELLRSTMNISQLGQFISSGVNISITLVNILFFADNNFAITYYGVYFLSMVLELFPCCYYGTLISVEMNQLT

YAIYSSNWMSMNRSYSRILLIFMQLTLAEVQIKAGGMIGIGMNAFFATVRLAYSFFTLAMSLR

>DmelOR33c

MVIIDSLSFYRPFWICMRLLVPTFFKDSSRPVQLYVVLLHILVTLWFPLHLLLHLLLLPSTAEFFKNLTMSLTCVACSL

KHVAHLYHLPQIVEIESLIEQLDTFIASEQEHRYYRDHVHCHARRFTRCLYISFGMIYALFLFGVFVQVISGNWELLYP

AYFPFDLESNRFLGAVALGYQVFSMLVEGFQGLGNDTYTPLTLCLLAGHVHLWSIRMGQLGYFDDETVVNHQRLLDYIE

QHKLLVRFHNLVSRTISEVQLVQLGGCGATLCIIVSYMLFFVGDTISLVYYLVFFGVVCVQLFPSCYFASEVAEELERL

PYAIFSSRWYDQSRDHRFDLLIFTQLTLGNRGWIIKAGGLIELNLNAFFATLKMAYSLFAVVVRAKGI

>DmelOR35a

MVRYVPRFADGQKVKLAWPLAVFRLNHIFWPLDPSTGKWGRYLDKVLAVAMSLVFMQHNDAELRYLRFEASNRNLDAFL

TGMPTYLILVEAQFRSLHILLHFEKLQKFLEIFYANIYIDPRKEPEMFRKVDGKMIINRLVSAMYGAVISLYLIAPVFS

IINQSKDFLYSMIFPFDSDPLYIFVPLLLTNVWVGIVIDTMMFGETNLLCELIVHLNGSYMLLKRDLQLAIEKILVARD

RPHMAKQLKVLITKTLRKNVALNQFGQQLEAQYTVRVFIMFAFAAGLLCALSFKAYTNPMANYIYAIWFGAKTVELLSL

GQIGSDLAFTTDSLSTMYYLTHWEQILQYSTNPSENLRLLKLINLAIEMNSKPFYVTGLKYFRVSLQAGLKILQASFSY

FTFLTSMQRRQMSN

>DmelOR42a

MDLRRWFPTLYTQSKDSPVRSRDATLYLLRCVFLMGVRKPPAKFFVAYVLWSFALNFCSTFYQPIGFLTGYISHLSEFS

PGEFLTSLQVAFNAWSCSTKVLIVWALVKRFDEANNLLDEMDRRITDPGERLQIHRAVSLSNRIFFFFMAVYMVYATNT

FLSAIFIGRPPYQNYYPFLDWRSSTLHLALQAGLEYFAMAGACFQDVCVDCYPVNFVLVLRAHMSIFAERLRRLGTYPY

ESQEQKYERLVQCIQDHKVILRFVDCLRPVISGTIFVQFLVVGLVLGFTLINIVLFANLGSAIAALSFMAAVLLETTPF

CILCNYLTEDCYKLADALFQSNWIDEEKRYQKTLMYFLQKLQQPITFMAMNVFPISVGTNISVTKFSFSVFTLVKQMNI

SEKLAKSEMEE

>DmelOR42b

MVFELIRPAPLTEQKRSRDGCIYLYRAMKFIGWLPPKQGVLRYVYLTWTLMTFVWCTTYLPLGFLGSYMTQIKSFSPGE

FLTSLQVCINAYGSSVKVAITYSMLWRLIKAKNILDQLDLRCTAMEEREKIHLVVARSNHAFLIFTFVYCGYAGSTYLS

SVLSGRPPWQLYNPFIDWHDGTLKLWVASTLEYMVMSGAVLQDQLSDSYPLIYTLILRAHLDMLRERIRRLRSDENLSE

AESYEELVKCVMDHKLILRYCAIIKPVIQGTIFTQFLLIGLVLGFTLINVFFFSDIWTGIASFMFVITILLQTFPFCYT

CNLIMEDCESLTHAIFQSNWVDASRRYKTTLLYFLQNVQQPIVFIAGGIFQISMSSNISVAKFAFSVITITKQMNIADK

FKTD

>DmelOR43a

MTIEDIGLVGINVRMWRHLAVLYPTPGSSWRKFAFVLPVTAMNLMQFVYLLRMWGDLPAFILNMFFFSAIFNALMRTWL

VIIKRRQFEEFLGQLATLFHSILDSTDEWGRGILRRAEREARNLAILNLSASFLDIVGALVSPLFREERAHPFGLALPG

VSMTSSPVYEVIYLAQLPTPLLLSMMYMPFVSLFAGLAIFGKAMLQILVHRLGQIGGEEQSEEERFQRLASCIAYHTQV

MRYVWQLNKLVANIVAVEAIIFGSIICSLLFCLNIITSPTQVISIVMYILTMLYVLFTYYNRANEICLENNRVAEAVYN

VPWYEAGTRFRKTLLIFLMQTQHPMEIRVGNVYPMTLAMFQSLLNASYSYFTMLRGVTGK

>DmelOR43b

MFGHFKLVYPAPISEPIQSRDSNAYMMETLRNSGLNLKNDFGIGRKIWRVFSFTYNMVILPVSFPINYVIHLAEFPPEL

LLQSLQLCLNTWCFALKFFTLIVYTHRLELANKHFDELDKYCVKPAEKRKVRDMVATITRLYLTFVVVYVLYATSTLLD

GLLHHRVPYNTYYPFINWRVDRTQMYIQSFLEYFTVGYAIYVATATDSYPVIYVAALRTHILLLKDRIIYLGDPSNEGS

SDPSYMFKSLVDCIKAHRTMLNFCDAIQPIISGTIFAQFIICGSILGIIMINMVLFADQSTRFGIVIYVMAVLLQTFPL

CFYCNAIVDDCKELAHALFHSAWWVQDKRYQRTVIQFLQKLQQPMTFTAMNIFNINLATNINVAKFAFTVYAIASGMNL

DQKLSIKE

>DmelOR45a

MDASYFAVQRRALEIVGFDPSTPQLSLKHPIWAGILILSLISHNWPMVVYALQDLSDLTRLTDNFAVFMQGSQSTFKFL

VMMAKRRRIGSLIHRLHKLNQAASATPNHLEKIERENQLDRYVARSFRNAAYGVICASAIAPMLLGLWGYVETGVFTPT

TPMEFNFWLDERKPHFYWPIYVWGVLGVAAAAWLAIATDTLFSWLTHNVVIQFQLLELVLEEKDLNGGDSRLTGFVSRH

RIALDLAKELSSIFGEIVFVKYMLSYLQLCMLAFRFSRSGWSAQVPFRATFLVAIIIQLSSYCYGGEYIKQQSLAIAQA

VYGQINWPEMTPKKRRLWQMVIMRAQRPAKIFGFMFVVDLPLLLWVIRTAGSFLAMLRTFER

>DmelOR45b

MYPRFLSRNYPLAKHLFFVTRYSFGLLGLRFGKEQSWLHLLWLVFNFVNLAHCCQAEFVFGWSHLRTSPVDAMDAFCPL

ACSFTTLFKLGWMWWRRQEVADLMDRIRLLIGEQEKREDSRRKVAQRSYYLMVTRCGMLVFTLGSITTGAFVLRSLWEM

WVRRHQEFKFDMPFRMLFHDFAHRMPWFPVFYLYSTWSGQVTVYAFAGTDGFFFGFTLYMAFLLQALRYDIQDALKPIR

DPSLRESKICCQRLADIVDRHNEIEKIVKEFSGIMAAPTFVHFVSASLVIATSVIDILLYSGYNIIRYVVYTFTVSSAI

FLYCYGGTEMSTESLSLGEAAYSSAWYTWDRETRRRVFLIILRAQRPITVRVPFFAPSLPVFTSVIKFTGSIVALAKTI

L

>DmelOR46aA

MSKGVEIFYKGQKAFLNILSLWPQIERRWRIIHQVNYVHVIVFWVLLFDLLLVLHVMANLSYMSEVVKAIFILATSAGH

TTKLLSIKANNVQMEELFRRLDNEEFRPRGANEELIFAAACERSRKLRDFYGALSFAALSMILIPQFALDWSHLPLKTY

NPLGENTGSPAYWLLYCYQCLALSVSCITNIGFDSLCSSLFIFLKCQLDILAVRLDKIGRLITTSGGTVEQQLKENIRY

HMTIVELSKTVERLLCKPISVQIFCSVLVLTANFYAIAVLSDERLELFKYVTYQACMLIQIFILCYYAGEVTQRSLDLP

HELYKTSWVDWDYRSRRIALLFMQRLHSTLRIRTLNPSLGFDLMLFSSIVNCSYSYFALLKRVNS

>DmelOR46aB

MVTEDFYKYQVWYFQILGVWQLPTWAADHQRRFQSMRFGFILVILFIMLLLFSFEMLNNISQVREILKVFFMFATEISC

MAKLLHLKLKSRKLAGLVDAMLSPEFGVKSEQEMQMLELDRVAVVRMRNSYGIMSLGAASLILIVPCFDNFGELPLAML

EVCSIEGWICYWSQYLFHSICLLPTCVLNITYDSVAYSLLCFLKVQLQMLVLRLEKLGPVIEPQDNEKIAMELRECAAY

YNRIVRFKDLVELFIKGPGSVQLMCSVLVLVSNLYDMSTMSIANGDAIFMLKTCIYQLVMLWQIFIICYASNEVTVQSS

RLCHSIYSSQWTGWNRANRRIVLLMMQRFNSPMLLSTFNPTFAFSLEAFGSIVNCSYSYFALLKRVNS

>DmelOR47a

MDSFLQVQKSTIALLGFDLFSENREMWKRPYRAMNVFSIAAIFPFILAAVLHNWKNVLLLADAMVALLITILGLFKFSM

ILYLRRDFKRLIDKFRLLMSNEAEQGEEYAEILNAANKQDQRMCTLFRTCFLLAWALNSVLPLVRMGLSYWLAGHAEPE

LPFPCLFPWNIHIIRNYVLSFIWSAFASTGVVLPAVSLDTIFCSFTSNLCAFFKIAQYKVVRFKGGSLKESQATLNKVF

ALYQTSLDMCNDLNQCYQPIICAQFFISSLQLCMLGYLFSITFAQTEGVYYASFIATIIIQAYIYCYCGENLKTESASF

EWAIYDSPWHESLGAGGASTSICRSLLISMMRAHRGFRITGYFFEANMEAFSSIVRTAMSYITMLRSFS

>DmelOR47b

MNDSGYQSNLSLLRVFLDEFRSVLRQESPGLIPRLAFYYVRAFLSLLCQYPNKKLASLPLYRWINLFIMCNVMTIFWTM

FVALPESKNVIEMGDDLVWISGMALVFTKIFYMHLRCDEIDELISDFEYYNRELRPHNIDEEVLGWQRLCYVIESGLYI

NCFCLVNFFSAAIFLQPLLGEGKLPFHSVYPFQWHRLDLHPYTFWFLYIWQSLTSQHNLMSILMVDMVGISTFLQTALN

LKLLCIEIRKLGDMEVSDKRFHEEFCRVVRFHQHIIKLVGKANRAFNGAFNAQLMASFSLISISTFETMAAAAVDPKMA

AKFVLLMLVAFIQLSLWCVSGTLVYTQSVEVAQAAFDINDWHTKSPGIQRDISFVILRAQKPLMYVAEPFLPFTLGTYM

LVLKNCYRLLALMQESM

>DmelOR49a

MEKLRSYEDFIFMANMMFKTLGYDLFHTPKPWWRYLLVRGYFVLCTISNFYEASMVTTRIIEWESLAGSPSKIMRQGLH

FFYMLSSQLKFITFMINRKRLLQLSHRLKELYPHKEQNQRKYEVNKYYLSCSTRNVLYVYYFVMVVMALEPLVQSCIMY

LIGFGKADFTYKRIFPTRLTFDSEKPLGYVLAYVIDFTYSQFIVNVSLGTDLWMMCVSSQISMHLGYLANMLASIRPSP

ETEQQDCDFLASIIKRHQLMIRLQKDVNYVFGLLLASNLFTTSCLLCCMAYYTVVEGFNWEGISYMMLFASVAAQFYVV

SSHGQMLIDLSTNLAKAAFESKWYEGSLRYKKEILILMAQAQRPLEISARGVIIISLDTFKILMTITYRFFAVIRQTVE

K

>DmelOR49b

MFEDIQLIYMNIKILRFWALLYDKNLRRYVCIGLASFHIFTQIVYMMSTNEGLTGIIRNSYMLVLWINTVLRAYLLLAD

HDRYLALIQKLTEAYYDLLNLNDSYISEILDQVNKVGKLMARGNLFFGMLTSMGFGLYPLSSSERVLPFGSKIPGLNEY

ESPYYEMWYIFQMLITPMGCCMYIPYTSLIVGLIMFGIVRCKALQHRLRQVALKHPYGDRDPRELREEIIACIRYQQSI

IEYMDHINELTTMMFLFELMAFSALLCALLFMLIIVSGTSQLIIVCMYINMILAQILALYWYANELREQNLAVATAAYE

TEWFTFDVPLRKNILFMMMRAQRPAAILLGNIRPITLELFQNLLNTTYTFFTVLKRVYG

>DmelOR56a

MFKVKDLLLSPTTFEDPIFGTHLRYFQWYGYVASKDQNRPLLSLIRCTILTASIWLSCALMLARVFRGYENLNDGATSY

ATAVQYFAVSIAMFNAYVQRDKVISLLRVAHSDIQNLMHEADNREMELLVATQAYTRTITLLIWIPSVIAGLMAYSDCI

YRSLFLPKSVFNVPAVRRGEEHPILLFQLFPFGELCDNFVVGYLGPWYALGLGITAIPLWHTFITCLMKYVNLKLQILN

KRVEEMDITRLNSKLVIGRLTASELTFWQMQLFKEFVKEQLRIRKFVQELQYLICVPVMADFIIFSVLICFLFFALTVG

VPSKMDYFFMFIYLFVMAGILWIYHWHATLIVECHDELSLAYFSCGWYNFEMPLQKMLVFMMMHAQRPMKMRALLVDLN

LRTFIDIGRGAYSYFNLLRSSHLY

>DmelOR59a

MAEVRVDSLEFFKSHWTAWRYLGVAHFRVENWKNLYVFYSIVSNLLVTLCYPVHLGISLFRNRTITEDILNLTTFATCT

ACSVKCLLYAYNIKDVLEMERLLRLLDERVVGPEQRSIYGQVRVQLRNVLYVFIGIYMPCALFAELSFLFKEERGLMYP

AWFPFDWLHSTRNYYIANAYQIVGISFQLLQNYVSDCFPAVVLCLISSHIKMLYNRFEEVGLDPARDAEKDLEACITDH

KHILELFRRIEAFISLPMLIQFTVTALNVCIGLAALVFFVSEPMARMYFIFYSLAMPLQIFPSCFFGTDNEYWFGRLHY

AAFSCNWHTQNRSFKRKMMLFVEQSLKKSTAVAGGMMRIHLDTFFSTLKGAYSLFTIIIRMRK

>DmelOR59b

MAVFKLIKPAPLTEKVQSRQGNIYLYRAMWLIGWIPPKEGVLRYVYLFWTCVPFAFGVFYLPVGFIISYVQEFKNFTPG

EFLTSLQVCINVYGASVKSTITYLFLWRLRKTEILLDSLDKRLANDSDRERIHNMVARCNYAFLIYSFIYCGYAGSTFL

SYALSGRPPWSVYNPFIDWRDGMGSLWIQAIFEYITMSFAVLQDQLSDTYPLMFTIMFRAHMEVLKDHVRSLRMDPERS

EADNYQDLVNCVLDHKTILKCCDMIRPMISRTIFVQFALIGSVLGLTLVNVFFFSNFWKGVASLLFVITILLQTFPFCY

TCNMLIDDAQDLSNEIFQSNWVDAEPRYKATLVLFMHHVQQPIIFIAGGIFPISMNSNITVAKFAFSIITIVRQMNLAE

QFQ

>DmelOR59c

MTKFFFKRLQTAPLDQEVSSLDASDYYYRIAFFLGWTPPKGALLRWIYSLWTLTTMWLGIVYLPLGLSLTYVKHFDRFT

PTEFLTSLQVDINCIGNVIKSCVTYSQMWRFRRMNELISSLDKRCVTTTQRRIFHKMVARVNLIVILFLSTYLGFCFLT

LFTSVFAGKAPWQLYNPLVDWRKGHWQLWIASILEYCVVSIGTMQELMSDTYAIVFISLFRCHLAILRDRIANLRQDPK

LSEMEHYEQMVACIQDHRTIIQCSQIIRPILSITIFAQFMLVGIDLGLAAISILFFPNTIWTIMANVSFIVAICTESFP

CCMLCEHLIEDSVHVSNALFHSNWITADRSYKSAVLYFLHRAQQPIQFTAGSIFPISVQSNIAVAKFAFTIITIVNQMN

LGEKFFSDRSNGDINP

>DmelOR63a

MYSPEEAAELKRRNYRSIREMIRLSYTVGFNLLDPSRCGQVLRIWTIVLSVSSLASLYGHWQMLARYIHDIPRIGETAG

TALQFLTSIAKMWYFLFAHRQIYELLRKARCHELLQKCELFERMSDLPVIKEIRQQVESTMNRYWASTRRQILIYLYSC

ICITTNYFINSFVINLYRYFTKPKGSYDIMLPLPSLYPAWEHKGLEFPYYHIQMYLETCSLYICGMCAVSFDGVFIVLC

LHSVGLMRSLNQMVEQATSELVPPDRRVEYLRCCIYQYQRVANFATEVNNCFRHITFTQFLLSLFNWGLALFQMSVGLG

NNSSITMIRMTMYLVAAGYQIVVYCYNGQRFATASEEIANAFYQVRWYGESREFRHLIRMMLMRTNRGFRLDVSWFMQM

SLPTLMAMVRTSGQYFLLLQNVNQK

>DmelOR65a

MTELRSERKNGNWDRLFGPFFESWAVFKAPQAKSRHIIAYWTRDQLKALGFYMNSEQRRLPRIVAWQYFVSIQLATALA

SLFYGISESIGDIVNLGRDLVFIITIIFICFRLVFFAQYAGELDVIIDALEDIYHWSIKGPATKEVQETKRLHFLLFMA

LIITWFSFLILFMLIKISTPFWIESQTLPFHVSWPFQLHDPSKHPIAYIIIFVSQSTTMLYFLIWLGVVENMGVSLFFE

LTSALRVLCIELRNLQELCLGDEDMLYRELCRMTKFHQQIILLTDRCNHIFNGAFIMQMLINFLLVSLSLFEVLAAKKN

PQVAVEYMIIMLMTLGHLSFWSKFGDMFSKESEQVALAVYEAYDPNVGSKSIHRQFCFFIQRAQKPLIMKASPFPPFNL

ENYMFILKQCYSILTILANTLE

>DmelOR65b

MEASHSSIYYWREQMKAMALFTTTEERLLPYRSKWHTLVYIQMVIFFASMSFGLTESMGDHVQMGRDLAFILGAFFIIF

KTYYFCWYGDELDQVISDLDALHPWAQKGPNPVEYQTGKRWYFVMAFFLATSWSFFLCILLLLLITSPMWVHQQNLPFH

AAFPFQWHEKSLHPISHAIIYLFQSYFAVYCLTWLLCIEGLSICIYAEITFGIEVLCLELRQIHRHNYGLQELRMETNR

LVKLHQKIVEILDRTNDVFHGTLIMQMGVNFSLVSLSVLEAVEARKDPKVVAQFAVLMLLALGHLSMWSYCGDQLSQKS

LQISEAAYEAYDPTKGSKDVYRDLCVIIRRGQDPLIMRASPFPSFNLINYSAILNQCYGILTFLLKTLD

>DmelOR65c

MESSYSAVYYWREQMKAMFLYTTSKERQMPYRSSWHTLVIIQATVCFLTMCYGVTESLGDKVQMGRDIAFIIGFFYIAF

KIYYFQWYGDELDEVVEALETFHPWAQKGPGAVDYRTAKRWYFTLAFFLASSWLVFLCIFILLLITSPLWVHQQILPLH

AAFPFQWHEKSIHPISHAFIYLFQTWNVMYFLTWLVCIEGLSVSIYVEITFAIEVLCLELRHLHQRCHGYEQLRLETNR

LVQFHQKIVHILDHTNKVFHGTLIMQMGVNFFLVSLSVLEAMEARKDPKVVAQFAVLMLLALGHLSMWSYFGDLLSQKS

LTISEAAYEAYDPIKGSKDVYRDLCLIIRRGQEPLIMRASPFPSFNFINYSAILNQCYGILTFLLKTLD

>DmelOR67a

MDNVAEMPEEKYVEVDDFLRLAVKFYNTLGIDPYETGRKRTIWFQIYFALNMFNMVFSFYAEVATLVDRLRDNENFLES

CILLSYVSFVVMGLSKIGAVMKKKPKMTALVRQLETCFPSPSAKVQEEYAVKSWLKRCHIYTKGFGGLFMIMYFAHALI

PLFIYFIQRVLLHYPDAKQIMPFYQLEPWEFRDSWLFYPSYFHQSSAGYTATCGSIAGDLMIFAVVLQVIMHYERLAKV

LREFKIQAHNAPNGAKEDIRKLQSLVANHIDILRLTDLMNEVFGIPLLLNFIASALLVCLVGVQLTIALSPEYFCKQML

FLISVLLEVYLLCSFSQRLIDASENVGHAAYDMDWLGSDKRFKKILIFISMRSQKPVCLKATVVLDLSMPTMSIFLGMS

YKFFCAVRTMYQ

>DmelOR67b

MQDQLDHELERIDKLPKLGLLWVEYSAYALGVNIAPRKRSSKYCRLTRILVLIVNLSIIYSLVAFIMENYMISFETYVE

AVLLTFQLSVGVVKMFHFQNKVESCSQLVFSTETGEVLKSLGLFQLDLPRKKELLSSVSLILLNNWMIIDRQVMFFFKI

VCMPVLYYCVRPYFQYIFDCYIKDKDTCEMTLTYPAIVPYLQLGNYEFPSYVIRFFLLQSGPLWCFFAVFGFNSLFVVL

TRYESGLIKVLRFLVQNSTSDILVPKDQRVKYLQCCVRLFARISSHHNQIENLFKYIILVQCSVSSILICMLLYKISTV

LEVGWVWMGMIMVYFVTIALEITLYNVSAQKVESQSELLFHDWYNCSWYNESREFKFMIKMMLLFSRRTFVLSVGGFTS

LSHKFLVQVFRLSANFFLLLRNMNNK

>DmelOR67c

METAKDNTARTFMELMRVPVQFYRTIGEDIYAHRSTNPLKSLLFKIYLYAGFINFNLLVIGELVFFYNSIQDFETIRLA

IAVAPCIGFSLVADFKQAAMIRGKKTLIMLLDDLENMHPKTLAKQMEYKLPDFEKTMKRVINIFTFLCLAYTTTFSFYP

AIKASVKFNFLGYDTFDRNFGFLIWFPFDATRNNLIYWIMYWDIAHGAYLAGIAFLCADLLLVVVITQICMHFNYISMR

LEDHPCNSNEDKENIEFLIGIIRYHDKCLKLCEHVNDLYSFSLLLNFLMASMQICFIAFQVTESTVEVIIIYCIFLMTS

MVQVFMVCYYGDTLIAASLKVGDAAYNQKWFQCSKSYCTMLKLLIMRSQKPASIRPPTFPPISLVTYMKVISMSYQFFA

LLRTTYSNN

>DmelOR67d

MLKMAKVEPVERYCKVIRMIRFCVGFCGNDVADPNFRMWWLTYAVMAAIAFFFACTGYTIYVGVVINGDLTIILQALAM

VGSAVQGLTKLLVTANNASHMREVQNTYEDIYREYGSKGDEYAKCLEKRIRITWTLLIGFMLVYIILLGLVITFPIFYL

LILHQKVLVMQFLIPFLDHTTDGGHLILTAAHVILITFGGFGNYGGDMYLFLFVTHVPLIKDIFCVKLTEFNELVMKRN

DFPKVRAMLCDLLVWHQLYTRMLQTTKKIYSIVLFVQLSTTCVGLLCTISCIFMKAWPAAPLYLLYAAITLYTFCGLGT

LVENSNEDFLSVIYTNCLWYELPVKEEKLIIMMLAKAQNEVVLTAADMAPLSMNTALQLTKGIYSFSMMLMNYLG

>DmelOR69aA

MQLHDHMKYIDLGCKMACIPRYQWKGRPTERQFYASEQRIVFLLGTICQIFQITGVLIYWYCNGRLATETGTFVAQLSE

MCSSFCLTFVGFCNVYAISTNRNQIETLLEELHQIYPRYRKNHYRCQHYFDMAMTIMRIEFLFYMILYVYYNSAPLWVL

LWEHLHEEYDLSFKTQTNTWFPWKVHGSALGFGMAVLSITVGSFVGVGFSIVTQNLICLLTFQLKLHYDGISSQLVSLD

CRRPGAHKELSILIAHHSRILQLGDQVNDIMNFVFGSSLVGATIAICMSSVSIMLLDLASAFKYASGLVAFVLYNFVIC

YMGTEVTLASGKVLPAAFYNNWYEGDLVYRRMLLILMMRATKPYMWKTYKLAPVSITTYMATLKFSYQMFTCVRSLK

>DmelOR69aB

MQLEDFMRYPDLVCQAAQLPRYTWNGRRSLEVKRNLAKRIIFWLGAVNLVYHNIGCVMYGYFGDGRTKDPIAYLAELAS

VASMLGFTIVGTLNLWKMLSLKTHFENLLNEFEELFQLIKHRAYRIHHYQEKYTRHIRNTFIFHTSAVVYYNSLPILLM

IREHFSNSQQLGYRIQSNTWYPWQVQGSIPGFFAAVACQIFSCQTNMCVNMFIQFLINFFGIQLEIHFDGLARQLETID

ARNPHAKDQLKYLIVYHTKLLNLADRVNRSFNFTFLISLSVSMISNCFLAFSMTMFDFGTSLKHLLGLLLFITYNFSMC

RSGTHLILTSGKVLPAAFYNNWYEGDLVYRRMLLILMMRATKPYMWKTYKLAPVSITTYMATLKFSYQMFTCVRSLK

>DmelOR71a

MDYDRIRPVRFLTGVLKWWRLWPRKESVSTPDWTNWQAYALHVPFTFLFVLLLWLEAIKSRDIQHTADVLLICLTTTAL

GGKVINIWKYAHVAQGILSEWSTWDLFELRSKQEVDMWRFEHRRFNRVFMFYCLCSAGVIPFIVIQPLFDIPNRLPFWM

WTPFDWQQPVLLWYAFIYQATTIPIACACNVTMDAVNWYLMLHLSLCLRMLGQRLSKLQHDDKDLREKFLELIHLHQRL

KQQALSIEIFISKSTFTQILVSSLIICFTIYSMQMSPVLQDLPGFAAMMQYLVAMIMQVMLPTIYGNAVIDSANMLTDS

MYNSDWPDMNCRMRRLVLMFMVYLNRPVTLKAGGFFHIGLPLFTKTMNQAYSLLALLLNMNQ

>DmelOR74a

MSFHRYRPRLPGGELAPMPWPVSLYRVLNHVAWPLEAESGRWTVFLDRLMIFLGFLVFCEHNEVDFHYLIANRQDMDNM

LTGLPTYLILVEMQIRCFQLAWHKDRFRALLQRFYAEIYVSEEMEPHLFASIQRQMLATRVNSTVYLLALLNFFLVPVT

NVIYHRREMLYKQVYPFDNTQLHFFIPLLVLNFWVGFIITSMLFGELNVMGELMMHLNARYIQLGQDLRRSAQMLLKKS

SSLNVAIAYRLNLTHILRRNAALRDFGQRVEKEFTLRIFVMFAFSAGLLCALFFKAFTNPWGNVAYIVWFLAKFMELLA

LGMLGSILLKTTDELGMMYYTADWEQVIHQSDNVGENVKLMKLVTLAIQLNSRPFFITGLNYFRVSLTAVLKIIQGAFS

YFTFLNSMR

>DmelOR82a

MGRLFQLQEYCLRAMGHKDDMDSTDSTALSLKHISSLIFVISAQYPLISYVAYNRNDMEKVTACLSVVFTNMLTVIKIS

TFLANRKDFWEMIHRFRKMHEQSSHIPRYREGLDYVAEANKLASFLGRAYCVSCGLTGLYFMLGPIVKIGVCRWHGTTC

DKELPMPMKFPFNDLESPGYEVCFLYTVLVTVVVVAYASAVDGLFISFAINLRAHFQTLQRQIENWEFPSSEPDTQIRL

KSIVEYHVLLLSLSRKLRSIYTPTVMGQFVITSLQVGVIIYQLVTNMDSVMDLLLYASFFGSIMLQLFIYCYGGEIIKA

ESLQVDTAVRLSNWHLASPKTRTSLSLIILQSQKEVLIRAGFFVASLANFVGICRTALSLITLIKSIE

>DmelOR83a

MKSTFKEERIKDDSKRRDLFVFVRQTMCIAAMYPFGYYVNGSGVLAVLVRFCDLTYELFNYFVSVHIAGLYICTIYINY

GQGDLDFFVNCLIQTIIYLWTIAMKLYFRRFRPGLLNTILSNINDEYETRSAVGFSFVTMAGSYRMSKLWIKTYVYCCY

IGTIFWLALPIAYRDRSLPLACWYPFDYTQPGVYEVVFLLQAMGQIQVAASFASSSGLHMVLCVLISGQYDVLFCSLKN

VLASSYVLMGANMTELNQLQAEQSAADVEPGQYAYSVEEETPLQELLKVGSSMDFSSAFRLSFVRCIQHHRYIVAALKK

IESFYSPIWFVKIGEVTFLMCLVAFVSTKSTAANSFMRMVSLGQYLLLVLYELFIICYFADIVFQNSQRCGEALWRSPW

QRHLKDVRSDYMFFMLNSRRQFQLTAGKISNLNVDRFRGTITTAFSFLTLLQKMDARE

>DmelOR83b

MTTSMQPSKYTGLVADLMPNIRAMKYSGLFMHNFTGGSAFMKKVYSSVHLVFLLMQFTFILVNMALNAEEVNELSGNTI

TTLFFTHCITKFIYLAVNQKNFYRTLNIWNQVNTHPLFAESDARYHSIALAKMRKLFFLVMLTTVASATAWTTITFFGD

SVKMVVDHETNSSIPVEIPRLPIKSFYPWNASHGMFYMISFAFQIYYVLFSMIHSNLCDVMFCSWLIFACEQLQHLKGI

MKPLMELSASLDTYRPNSAALFRSLSANSKSELIHNEEKDPGTDMDMSGIYSSKADWGAQFRAPSTLQSFGGNGGGGNG

LVNGANPNGLTKKQEMMVRSAIKYWVERHKHVVRLVAAIGDTYGAALLLHMLTSTIKLTLLAYQATKINGVNVYAFTVV

GYLGYALAQVFHFCIFGNRLIEESSSVMEAAYSCHWYDGSEEAKTFVQIVCQQCQKAMSISGAKFFTVSLDLFASVLGA

VVTYFMVLVQLK

>DmelOR83c

MSTSESPSSRFRELSKYINSLTNLLGVDFLSPKLKFNYRTWTTIFAIANYTGFTVFTILNNGGDWRVGLKASLMTGGLF

HGLGKFLTCLLKHQDMRRLVLYSQSIYDEYETRGDSYHRTLNSNIDRLLGIMKIIRNGYVFAFCLMELLPLAMLMYDGT

RVTAMQYLIPGLPLENNYCYVVTYMIQTVTMLVQGVGFYSGDLFVFLGLTQILTFADMLQVKVKELNDALEQKAEYRAL

VRVGASIDGAENRQRLLLDVIRWHQLFTDYCRAINALYYELIATQVLSMALAMMLSFCINLSSFHMPSAIFFVVSAYSM

SIYCILGTILEFAYDQVYESICNVTWYELSGEQRKLFGFLLRESQYPHNIQILGVMSLSVRTALQIVKLIYSVSMMMMN

RA

>DmelOR85a

MIFKYIQEPVLGSLFRSRDSLIYLNRSIDQMGWRLPPRTKPYWWLYYIWTLVVIVLVFIFIPYGLIMTGIKEFKNFTTT

DLFTYVQVPVNTNASIMKGIIVLFMRRRFSRAQKMMDAMDIRCTKMEEKVQVHRAAALCNRVVVIYHCIYFGYLSMALT

GALVIGKTPFCLYNPLVNPDDHFYLATAIESVTMAGIILANLILDVYPIIYVVVLRIHMELLSERIKTLRTDVEKGDDQ

HYAELVECVKDHKLIVEYGNTLRPMISATMFIQLLSVGLLLGLAAVSMQFYNTVMERVVSGVYTIAILSQTFPFCYVCE

QLSSDCESLTNTLFHSKWIGAERRYRTTMLYFIHNVQQSILFTAGGIFPICLNTNIKMAKFAFSVVTIVNEMDLAEKLR

RE

>DmelOR85b

MEKLMKYASFFYTAVGIRPYTNGEESKMNKLIFHIVFWSNVINLSFVGLFESIYVYSAFMDNKFLEAVTALSYIGFVTV

GMSKMFFIRWKKTAITELINELKEIYPNGLIREERYNLPMYLGTCSRISLIYSLLYSVLIWTFNLFCVMEYWVYDKWLN

IRVVGKQLPYLMYIPWKWQDNWSYYPLLFSQNFAGYTSAAGQISTDVLLCAVATQLVMHFDFLSNSMERHELSGDWKKD

SRFLVDIVRYHERILRLSDAVNDIFGIPLLLNFMVSSFVICFVGFQMTVGVPPDIVVKLFLFLVSSMSQVYLICHYGQL

VADASYGFSVATYNQKWYKADVRYKRALVIIIARSQKVTFLKATIFLDITRSTMTDLLQISYKFFALLRTMYTQ

>DmelOR85c

MKFMKYAVFFYTSVGIEPYTIDSRSKKASLWSHLLFWANVINLSVIVFGEILYLGVAYSDGKFIDAVTVLSYIGFVIVG

MSKMFFIWWKKTDLSDLVKELEHIYPNGKAEEEMYRLDRYLRSCSRISITYALLYSVLIWTFNLFSIMQFLVYEKLLKI

RVVGQTLPYLMYFPWNWHENWTYYVLLFCQNFAGHTSASGQISTDLLLCAVATQVVMHFDYLARVVEKQVLDRDWSENS

RFLAKTVQYHQRILRLMDVLNDIFGIPLLLNFMVSTFVICFVGFQMTVGVPPDIMIKLFLFLFSSLSQVYLICHYGQLI

ADASSSLSISAYKQNWQNADIRYRRALVFFIARPQRTTYLKATIFMNITRATMTDLLQVSYKFFALLRTMYIK

>DmelOR85d

MLTKKDTQSAKEQEKLKAIPLHSFLKYANVFYLSIGMMAYDHKYSQKWKEVLLHWTFIAQMVNLNTVLISELIYVFLAI

GKGSNFLEATMNLSFIGFVIVGDFKIWNISRQRKRLTQVVSRLEELHPQGLAQQEPYNIGHHLSGYSRYSKFYFGMHMV

LIWTYNLYWAVYYLVCDFWLGMRQFERMLPYYCWVPWDWSTGYSYYFMYISQNIGGQACLSGQLAADMLMCALVTLVVM

HFIRLSAHIESHVAGIGSFQHDLEFLQATVAYHQSLIHLCQDINEIFGVSLLSNFVSSSFIICFVGFQMTIGSKIDNLV

MLVLFLFCAMVQVFMIATHAQRLVDASEQIGQAVYNHDWFRADLRYRKMLILIIKRAQQPSRLKATMFLNISLVTVSDL

LQLSYKFFALLRTMYVN

>DmelOR85e

MASLQFHGNVDADIRYDISLDPARESNLFRLLMGLQLANGTKPSPRLPKWWPKRLEMIGKVLPKAYCSMVIFTSLHLGV

LFTKTTLDVLPTGELQAITDALTMTIIYFFTGYGTIYWCLRSRRLLAYMEHMNREYRHHSLAGVTFVSSHAAFRMSRNF

TVVWIMSCLLGVISWGVSPLMLGIRMLPLQCWYPFDALGPGTYTAVYATQLFGQIMVGMTFGFGGSLFVTLSLLLLGQF

DVLYCSLKNLDAHTKLLGGESVNGLSSLQEELLLGDSKRELNQYVLLQEHPTDLLRLSAGRKCPDQGNAFHNALVECIR

LHRFILHCSQELENLFSPYCLVKSLQITFQLCLLVFVGVSGTREVLRIVNQLQYLGLTIFELLMFTYCGELLSRHSIRS

GDAFWRGAWWKHAHFIRQDILIFLVNSRRAVHVTAGKFYVMDVNRLRSVITQAFSFLTLLQKLAAKKTESEL

>DmelOR85f

MEPVQYSYEDFARLPTTVFWIMGYDMLGVPKTRSRRILYWIYRFLCLASHGVCVGVMVFRMVEAKTIDNVSLIMRYATL

VTYIINSDTKFATVLQRSAIQSLNSKLAELYPKTTLDRIYHRVNDHYWTKSFVYLVIIYIGSSIMVVIGPIITSIIAYF

THNVFTYMHCYPYFLYDPEKDPVWIYISIYALEWLHSTQMVISNIGADIWLLYFQVQINLHFRGIIRSLADHKPSVKHD

QEDRKFIAKIVDKQVHLVSLQNDLNGIFGKSLLLSLLTTAAVICTVAVYTLIQGPTLEGFTYVIFIGTSVMQVYLVCYY

GQQVLDLSGEVAHAVYNHDFHDASIAYKRYLLIIIIRAQQPVELNAMGYLSISLDTFKQLMSVSYRVITMLMQMIQ

>DmelOR88a

MKPTEIKKPYRMEEFLRPQMFQEVAQMVHFQWRRNPVDNSMVNASMVPFCLSAFLNVLFFGCNGWDIIGHFWLGHPANQ

NPPVLSITIYFSIRGLMLYLKRKEIVEFVNDLDRECPRDLVSQLDMQMDETYRNFWQRYRFIRIYSHLGGPMFCVVPLA

LFLLTHEGKDTPVAQHEQLLGGWLPCGVRKDPNFYLLVWSFDLMCTTCGVSFFVTFDNLFNVMQGHLVMHLGHLARQFS

AIDPRQSLTDEKRFFVDLRLLVQRQQLLNGLCRKYNDIFKVAFLVSNFVGAGSLCFYLFMLSETSDVLIIAQYILPTLV

LVGFTFEICLRGTQLEKASEGLESSLRSQEWYLGSRRYRKFYLLWTQYCQRTQQLGAFGLIQVNMVHFTEIMQLAYRLF

TFLKSH

>DmelOR92a

MLFRKRKPKSDDEVITFDELTRFPMTFYKTIGEDLYSDRDPNVIRRYLLRFYLVLGFLNFNAYVVGEIAYFIVHIMSTT

TLLEATAVAPCIGFSFMADFKQFGLTVNRKRLVRLLDDLKEIFPLDLEAQRKYNVSFYRKHMNRVMTLFTILCMTYTSS

FSFYPAIKSTIKYYLMGSEIFERNYGFHILFPYDAETDLTVYWFSYWGLAHCAYVAGVSYVCVDLLLIATITQLTMHFN

FIANDLEAYEGGDHTDEENIKYLHNLVVYHARALDLSEEVNNIFSFLILWNFIAASLVICFAGFQITASNVEDIGVYFI

FFSASLVQVFVVCYYGDEMISSSSRIGHSAFNQNWLPCSTKYKRILQFIIARSQKPASIRPPTFPPISFNTFMKVISMS

YQFFALLRTTYYG

>DmelOR94a

MDKHKDRIESMRLILQVMQLFGLWPWSLKSEEEWTFTGFVKRNYRFLLHLPITFTFIGLMWLEAFISSNLEQAGQVLYM

SITEMALVVKILSIWHYRTEAWRLMYELQHAPDYQLHNQEEVDFWRREQRFFKWFFYIYILISLGVVYSGCTGVLFLEG

YELPFAYYVPFEWQNERRYWFAYGYDMAGMTLTCISNITLDTLGCYFLFHISLLYRLLGLRLRETKNMKNDTIFGQQLR

AIFIMHQRIRSLTLTCQRIVSPYILSQIILSALIICFSGYRLQHVGIRDNPGQFISMLQFVSVMILQIYLPCYYGNEIT

VYANQLTNEVYHTNWLEcRPPIRKLLNAYMEHLKKPVTIRAGNFFAVGLPIFVKTINNAYSFLALLLNVSN

>DmelOR94b

MESTNRLSAIQTLLVIQRWIGLLKWENEGEDGVLTWLKRIYPFVLHLPLTFTYIALMWYEAITSSDFEEAGQVLYMSIT

ELALVTKLLNIWYRRHEAASLIHELQHDPAFNLRNSEEIKFWQQNQRNFKRIFYWYIWGSLFVAVMGYISVFFQEDYEL

PFGYYVPFEWRTRERYFYAWGYNVVAMTLCCLSNILLDTLGCYFMFHIASLFRLLGMRLEALKNAAEEKARPELRRIFQ

LHTKVRRLTRECEVLVSPYVLSQVVFSAFIICFSAYRLVHMGFKQRPGLFVTTVQFVAVMIVQIFLPCYYGNELTFHAN

ALTNSVFGTNWLEYSVGTRKLLNCYMEFLKRPVKVRAGVFFEIGLPIFVKTINNAYSFFALLLKISK

>DmelOR98a

MLFNYLRKPNPTNLLTSPDSFRYFEYGMFCMGWHTPATHKIIYYITSCLIFAWCAVYLPIGIIISFKTDINTFTPNELL

TVMQLFFNSVGMPFKVLFFNLYISGFYKAKKLLSEMDKRCTTLKERVEVHQGVVRCNKAYLIYQFIYTAYTISTFLSAA

LSGKLPWRIYNPFVDFRESRSSFWKAALNETALMLFAVTQTLMSDIYPLLYGLILRVHLKLLRLRVESLCTDSGKSDAE

NEQDLIKCIKDHNLIIDYAAAIRPAVTRTIFVQFLLIGICLGLSMINLLFFADIWTGLATVAYINGLMVQTFPFCFVCD

LLKKDCELLVSAIFHSNWINSSRSYKSSLRYFLKNAQKSIAFTAGSIFPISTGSNIKVAKLAFSVVTFVNQLNIADRLT

KN

>DmelOR98b

MLTDKFLRLQSALFRLLGLELLHEQDVGHRYPWRSICCILSVASFMPLTIAFGLQNVQNVEQLTDSLCSVLVDLLALCK

IGLFLWLYKDFKFLIGQFYCVLQTETHTAVAEMIVTRESRRDQFISAMYAYCFITAGLSACLMSPLSMLISYHEQVNCS

RNFHFPVYPWDNMKLSNYIISYFWNVCAALGVALPTVCVDTLFCSLSHNLCALFQIARHKMMHFEGRNTKETHENLKHV

FQLYALCLNLGHFLNEYFRPLICQFVAASLHLCVLCYQLSANILQPALLFYAAFTAAVVGQVSIYCFCGSSIHSECQLF

GQAIYESSWPHLLQENLQLVSSLKIAMMRSSLGCPIDGYFFEANRETLITIVRTAISYVTLLRSLA

>PameOR1

MYKARLHGLVADLWPLIRIMQMTGFFLLDYHEDMSFGWTSIRAGYSGTVSGLMVIQFLLLFLNLMHQAED

VNDLTANTITVLFFVHSITKFFYFAIRRNKFYRTLATWNNANSHPLFAENHARHHATAVGSMRRLVMYVV

AVTVLSGFAWTGITFVGDSVHEIADPENANETIIEELPRLMVRSWYPWNAMSGGGYFVSFIIQLIWLFLA

LLHAMLMDTMFCCWLIYTCEQLIHLKEIMKPLMELSASLDTVVPHSAELFRAVSANTNNPAATGDDGIRA

IYSNQHDFSNFRLNTGTLANVNTGNVGPNGLTKKQELLVRSAIKYWVERHKHVVRFVSNIGDTYGAALLL

HMLTSTVTLTLLAYQATKIEGVDVYACTVIGYLVYTLGQVFLFCFYGNRLIEESSSVMEAAYSCQWYDGS

EEAKTFIQIVCQQCQKAMSISGAKFFTVSLDLFASVNQIIGKLYHVCTMQST

>SgreOR113

YVFSGYLVAPEVDADSQRDLFFLNRDHKGWKLYTSLVNNIRHHQLIIEYVKDLEVVTSTSLYLLLLANALNVCLHSFGFV

ALFQEGATGSTVIKEVLSFPSFLGQTALYCFFGQLVIDQADRLQFSGFSCDWPQADETFRRSLRIFMLQAARPLSVRVGK

LVTLSRNSFLQALNA

>SgreOR110

HLAILVGGLVEIVIDPPPLDVILEGIFTIACSFTWGLRMVSVLVRQSRVQQLVVDVLNMRKQFTENSAVLRKSYHRRALI

VCVAWVVFPLLAVPMWFVEPALTKTLVTTSENTTIVIRKTPFVMWMPLDTQTHPNYEITYVIQIVLLVSVVQATVLVDLF

FASLMINITADIAILNNNIANMRLHKENGVTKESAEGVKVSGAWEVTYKRKKSLAEKGEQHDENIATTTPYTSDPSAQLY

RTLARNIQHHQVIMSIINDLESLMSESSVLMLGVNSVNICLQGLGFVDAFRPGAKRSTVLKKVLTFPAYINQTAHFCWYG

QEIIDQSERLLESAFSCGWAEADQRFCSSLRIFMLQASRPLKLQIGKIFTLSRNLFLQILNTSYTIFNMMINF

>SgreOR112

VMLWMGDPLMQTLMSPPAENGTRPLIFWIPVEVKNSPAYEITYAIEAFFIGSATETSILIDIILIILLVYAAGEIAVLNE

NIAGMRLSNTRQREKVEPNKGLKALERSIDEVSISQTAYVFSEDSSDSKRNLFVLDRNDTYWKLHSSLVRNIRHHEVILT

YINDLEIVLSTSIYVLLLANALNICLHSFGIVALFQEGATSSTVFKEVISFASFLAQTGLFCFIGQLIIDQADRLQFSAF

SCDWPQADETFCRSLRIFMVQAARPLSVRVGKLVELSRNSFLQAMNASYTIFNMLFNLQTAD

>SgreOR108

FQCTNCSSWTIRIVLFMQQRVRLGRLVMTLLDTRKRYAEQVPGIRSSYDQGAVIIFFAWQVLPFTSISLWALGPVTDTPK

LVTAGNYSVVERREPMVLWLPLDTQQSPTYEIVFVLQVVGVVAMSEVSILLDIFFVCLMIHVTAEIAVLNKNLSSIHLLS

LNGDRSQMLELVDVTALGRQRYNGGEQHTDSEETIEWSVTGSGPLRSSTFLSSQDMNDAKRRLYATLKTNIQHHQTIILC

VSELEQGMSESTYVVLLVNALTICLQAFGFVELFQGGWKGPAVMKRLLACPIYMAQTALFCLFGQSLTDHSERLLDSGFS

CGWPTADSRFCSALLIFMQQASQPLSIRVGKIFTLSRNSFLQIMNASYTIFNMLLNTQ

>SgreOR105

FSFSLDGHRLTELLRLLAASRQRFPDTQGRRSRHHVTATRLHRFLQVTYRMNSAYWSVAPIIRIVAAGSASRASRDLVIP

LWLPLDTRASPAYETLYCLQLAFGWTVSETTVLVDGSLIALMLQVAAELAVLNDRLAATAADIGEGVPGTTRVQVSPASP

SSLVVGERCGSSITTMSRSRYPPDEMYQQLVDNIKHHQMIIRCVSLLQKLLSRATSVLLFCNTVSICFQIMATAVLLQED

VEVIQTVKMLMSSTLYAYQVALFCLLGQKIINESDRLPRSAFSCGWPEADTHCWRLLVALCRRSSRALSLKVCGLYTLSR

ETLLQILNVSYSLFNFMYQTDKSAASRH

>SgreOR106

DTQSSPTYEILLGVQVPCCWICSETSVLLDCAMLALMLQAAAELAVLNDRLCGVGADRAPSAAPTACHKDTPPAKASYHS

EGKSAGAAEAEVRSPLYSHDEMFRSLVENINHHQIIINYMHLLQMLLSRGISVLLICNTISICFHIVATVALLQEDIEPV

GMTKMVLGSTLYAYQTAILCLLGQRITTQSERLPASAFSCDWPAADGRFRRLLIVFCVRSSQALVIRVCGLYSLSRETLL

QVLKAAYTLFNFVYQTVGEEEPLN

>SgreOR119

GGATSRGRRTVVQSGLLSTRSQSSLGCVSFTRIVLISLLVKALWGPGEMSDDLGELLGPVAAALRLLGLQAPSTSWSLTW

RALLLLVVNASVPAMAASKLWVAPAAKLEELAVEVFVCVTGVAMTVKVAAFLWQQEQTQRLAVLLADCRRRFVCSGGGPG

DTRARYRRHIRRIVVYMQVMVVVPTTLWLLDPLVGAGSSAGRLPMPMWVPPGLRSSPGHQILYAFQALAMITVVEASLYL

DVCFVVLMLSVSAELHVLNDAVASIKAPAVLRPALYSASYRVTNVINGSWLRCGLSSPRGAIAYNGHQSTEQRTESSAII

TDDANGIGTEMYRVLVKSIKHHQAIIRCVEELESVMSQSVFVLLFLNMMNICVHIFVTSVLLQKDVERTTMSKMLCTLPI

YMYETGLYCIFGQTIIDQGEQLTASAFSGDWPEGDARMRKALLVLMLRSSKPLKLTVGKLYVLSRHTFLQILNGSYTLFN

MLYQVQTKK

>SgreOR117

MEEEGDLLGATALALQLMGLWRGGGGGGSAAARLAVAAPTLFVMGSAVVLSGAKLCADTPAVYEELIAVIFILVASVSWT

FKVITAVPIAMWALEPLLSGGQNTPLPAWLPLDIHATPAYELLCTFQAVAVTLSVEASVCLDMFFIVLMVAVAGELHILN

DNLEAMRLHPVDTFPRKAAENSATRWNGKASPLMEKGIPNTQQYGYHPENHKSTSALYATADAHELMYRSLVKNIQHHQL

ILKCVKELETAMSYSIFVLLFLNMVTICTLIISTTVLLQSDSDPTSLYKMVSSLPIVMFQTGLFCIFGQMIIDQSERLPA

TAFGSGWLEGNVRLRRALLLLMRRAATPLRITVGRMYPLSRHTYLQLLNGSYTIFNMMYQVRGRSD

>SgreOR103

YEILYAAQCVALTIASVCSFCTDIFFIHLMQVLGTEFEVLSDNFSAVQKNYEQPARASDRGELSEYQTTKETLVSSCAKI

YVDCVSGEQMHRQLGKNIQHHQALLRSLSLLQTTMSVPIFFVLAINIVNLCGNLFISIVLLQRDG

>SgreOR111

ILVENIKHHQLILVYIKKLEELMRTSIFIILLSNALSICLHVLGLAVMLQEGVDLPSVLKMLITFPAYSCETGLYCMFGQ

AIIDQSERLVDSAFNCDWINMGPRFRRSLTIFMLQASQPLNVKVGKRVILSRHTFLRLLNGSYSLFNMLHGVQK

>SgreOR116

CVGDLERVMGQSIFVILLTNVINICSQAFASAVLFRSGIDWGLVTKMLFTFVAYMFETGMFCMFGQAITDQSERLVESVG

GCGWLEAEAGVRRALLVMLTQASRPLTIHVGKVAALSRSSFLQLLNMSYTVFNLLFELQSAE

>SgreOR114

MRDEVDELLAPCAAALRLLGMWGVATRRGVFVLLLNLAGTACAAVTLAFQPPRGLERLAMNAYVCAQGTLLSVKVAAFLW

HRKRLRQLALQLVSCWRQFEDVGGGVRAIYRSQAARVVRYMQVMTGIPTMMWMLEPLFSGGEDSPQGRSLPLPTWLPDTL

QQSPTYELLYVLQVFTIVVAIAVSVYLNIFFAVLMLSTAAELHVLNNNMMAMGKFGDNETAESYRGDEGVQRDGVTSSLL

SSHRRPRGKLVSAGHSLPVAVYTHNGSHDQMYHQLIKNIRHHQVILRSVAELQKAMTHSIFVLLFLNMLNICVVIFAGTT

LLQKQADQVAMYKMLFSVPIYMYETGFFCVVGQTIIDQGERLSMSAFASAWLDSPRKLHRLLLVFMLRCTRPPTITVGKT

YTLSKRTFLRTQNSTKSQFK

>SgreOR104

CVSELEKAVSMGIFAVLSINILNLCSHVFSLVVMLETDTSMSTKIKMFLAAPVFMCQSGLYCLTGQAIIDQSDRLADSAY

SCGWPDADQRFKRSLRVLMTRAEQPLCIKVGKLIELSRATFQELLKGTYQLFNLVYQVHTN

>SgreOR118

VSDVPVDSTTQRISDDMYDQLVGNIRHHQLIIECVQVLQMAMSYSIFALLFFNMTSICLNIFVTASLLQSDADLVTALKA

VFTTPVFLYESAMYCIFGQMIIDQSEHLPLSAFSCGWTDADTRLQKALLVFMLRSSQPLRIQVGKTYELSKDTFVRVLNG

SYALFNMLYTFQGKK

>SgreOR109

DDAKRRLYAILKMNIQHHQAIMLCVNELEQVMSMSTYIILLVNALTICLHAFGFVELFQGGWKGPAVVKRLLACPIYMAQ

TALFCLFGQSLTDHSERLLDSAFSCGWPGADGRFCSALLIFMQQASQPLKIRVGKIFTLSRNSFLQIMNVSYTIFNMLLN

TQ

>SgreOR88

MSRRINRCCTLPAITQFEVSASEEGLRLSDSAVSLLGPGATLRRLMGLWQPEDGAVLSVSSVLASITLGSIAFPVVCSIL

KLCVDPPQPLEEVTLCGFVACMDFGFLIKAALFIKQGATMHQLVRLLWETRGRYSNGLNNEKSRARYEKLVGRLYVYMQV

VVVPALLCWVSSPLLSQVFLTSREDAPDGSRQLPVHVWLPADVNRSPTYEILYVAQSFSLMVLSQATVCMDVFFVHLMLM

VAAELEVLNDNISAMEQGQLQRGRSRYDGTTITDDRNIDEHEFIMSDHLQDVALTSSLGAEDKHMYAELVKNVHHHQQVL

RSVSLLQKAMDASIFILLFINMANLCGTVFVAAVLLQRDGNITKALKELMLIPCVLYETGMYCLCGHMIINQSEKLVSSA

FSCGWPDCDRRFKHSLLIFMVAAMRPLEITV

>SgreOR115

LCWLGKGMKQPEERLLQPCAGPLRFLGLWGPPVRPLRACAVLLLNAAGTLCAALALWAAPGRSLERLAADAYVGVTGIAM

TVKVSAFLWHRSRLQRLARILGAAGERSGGARGRYRRNAARVVRYMQMMVSVPAVMWTLDPVIVDDGGASAGRSLPLPIW

LPGLDTSASPLYELVYALQVCTLVVAVEASLYLDVFFVVLMLAVAGELHALNDAVAAIRSPGGRPRPAPTARAATGTVDA

APGAAPSAYQAMVGVVGRHQLILASIRELELVMNHSIFLLLFLNMLNICVHTFVTAVLLQKEVQPTTMYKMVSTIPIYMY

ETGLFCIFGQSIIDQGER

>SgreOR102

PAPLLGLQLGMVGVLASTAAHIHQGGGGDEDEEARQTSVAIFIVGTVIGMLVKIWSFMGQRGRMQLLLGLLLEMRRRYQR

DSRGARPRAQANGVTLFYILQVNAVAAALAWSVQPLISGADRRLPLPAWLPFDATASPYYEAVYVGQALSLLLVPQISLC

LNICYFALMLHLAAELAILRDNVAVVGWRRPSKEAAFQAERQAVDREFAAAPENRLLEDNVRHHQLIIRAVSELEQIMST

SVYIHLFVNMINVCSHIFVISVVLLETDEMAVVVSTASSLAVFLSGIALYCIIGHTIIDQSEQLPEAVYSSGWTGADASF

RSTVSILMVRASQPLSITVGKMRVLSKPTFVQVLNGSYTLFNFLYRTQSDKERKS

>SgreOR81

MALHTSLSLCVRLLNMAGLWPPASARHWLTPAGLLYRLFTAVMVAPSWHILLPEAAGLVHFAGDMKAATEDMCLLCAFVT

TSYKLLVILLRRTQIVRFVEALDARVTAMAAESAEARAVVRRRDLWSRRLALFMVIQSTSAAVIWSVNGLRIGLTMGSSL

RTLPIISWYPYNMTVWSNYEVTFFTQMLALVTAAFCNRACDILIITLMTQISSLLEMLNLKFRDVSKIDQSP

>SgreOR95

MERADSDLQALLGPGASIRRLMGVWWSQGRYGRICSAASASVTVSSFIWLSTFTGLKLVLDPPQQLEDIALCCFVIVLCS

GFLAKVAFFIYKSDALRELLQLLSDARRNYGDGKSSRSIRRRYQKLSRRLYLCMQVAVLQGLAAWVSTPVLVRVFLTSEQ

NSLESARQFPVPLWFPGNMYASPTYEILYILQAFSQLAATQSSLCVDAFFIHMILMVAAELEVLNENISTMQKIHLKFQG

TEHQHHRTITKYEEPTIENGYLHTTTYVSSEDFSGEMYLQLVKNVQHHHVILRSVSLLQRVMTISIFILLFVNMADLCSC

IFVTAVVLQRDGNITKALKPLLTIPPLLYETGMYCIFGQILTDQSEKLAYSAVNCGWADCDARFKRSLLVFMTVAGRPLE

ITVG

>SgreOR32

LSKIVLLQFTGATVIICVTLFQSSKNTENIAALLLLQAYLGVVIYEIFMYCWYADDILYQNCRLAKSAYYCGWPGAPPDL

QRALVFIICRTHRPLGLTAGKFYYVSRETFVRLMSASYSYYALLNQVNGN

>SgreOR18

DFGEFMAPRDVPLLRRHSRNQRRFVLAYLSLGMCDVSTWLTSPMRDTGLPLDVILPFDTAHPIGWAAGWLYCAFITVHAV

VMNMMADAFNVSLMAQLRMQLIILRKNINDLASNGESAVSSKSRTTFKGAERPWKLYTRMYKPYPLRKFNNHNQKMNSIM

LVAEAKKHQQSSEFSNSNDIHYRLKRIIIQHQTIIRNTEFLQKCLGGMLLGQSLALGVAICILLFQVGLSAQSARETGKF

GAYLCAMFTELSVYCWFGDKLMSESEKVAFAAYDVVTSMQECPVSIKRTLLLIMLRAQRPLCITAAGFFPFSRESFVSII

NVSYSFFAILRNFKEE

>SgreOR25

AESMCSGQPAHSRRDVAAPADTSEECVRRRLAQCVRYHTDVDRCVQQLSTLLGPILLGQVLADVVTISATAFVTTTGKTD

SGWVFKYGSYLAAIAEQLLLYCWFGNDVLTESERLQLSAYSSQWVAASPRFRKELLLFLCRAHRPLRLTASKFYTISRET

FLMLMNASFSYYAVLRQLNSD

>SgreOR107

AAMEESSLLGPSAVGVRHLGLWAAPGDTGPPGGSLRLALVVAAHLGLFSTSTANLVMDTPDDLEVLTINAFACMTTLGMI

VKVVSYVVDRGSFNALLVRLAAARRAHADPGGRNAATRARRLAFGTALYRFTQVSTVFCTSLWSVAPLLGGSSGSRDFPV

PIWLPLDMRATPTYQVVYLLQVFCIWAVVQGTVFLDTTFQILMLQLAAELEVLNDNVAAIYNREVGPSAVKRAEDIKIDA

SPQREKDEAIDRHGYIELHTDTAEDEMYSHLVSNIKHHQTVIGCLSQLETVMSHSTLILLSSNMVCICLHIFVTAVLLQD

EIQFDKTFKMMCAFAIYTYQTGLFCLIGQTIIDQSERLVNSAFSCAWPDADARFKLSLSVFMVRSARLLEIRVGKMYTLS

RETFLQILNGSYRLFNLLYQTNLEN

>SgreOR10

YLLRPLHWMAVLRHPSGAGRSPLCFRLYTFAMASFTLSFFCSEVIVLYREGTEDLDVFTLTLSVADTDIIWLFRMAHAIV

CERAFHKLALQVGHDFAEFLTWDDIPVLRVQCRKVRRFTLTYISFGIGACSCYLVSPVSAEGLPFILALPFDATQPLGFG

VTWVYCMITTLHVVVMTMILDSFNVSLIAQLRMQITLLSSKIVSLAKEVSERPENCSETYAQYELRYRLLKCIRHHQTII

KNADLLERSLGAMLLAQSLSIGASACFQMFQIATSANGLQQTGKFICYLFAMLAELFVYCWFGDDLITESENLALAAYDA

VTSLQECPLSLKRSLLLLMHRAQRPLRVTAGGFFPLSRE

>SgreOR84

LMGLWQPRGRVARVLNRLLAGMTLSCITFLVLCASLKLYMDPPEDLEQIALCTLVATVGAGFIIRAVLFKAQGGTLRQTL

RLLEDTRLQFCNGDDNELMRRRYQTLSNNIYYYCQMVAVPAVTAWAVCPLLSRIVTKTDQEQMEAQCQLPVPVWLPADIY

SSPTFEFLYVVQSFSVLVATESCLSIDIFFVHMMLMVAAELEVLNDNLSAMEHINLEMLTTEGEGFISGYKRKDRRLSVI

TSGQSLGEQTLTEKAAHEWLHQQLVKNVLHHQAILRSVSLLQSAMNVSIFSLLFVNMANLCSSLFVASILLQKEGNVGKA

LNALFTIPAQVYETTIYCIYGHIMTDQSERLMYSAFSCGWVNSDARFKRSLIIFMKVTARPIVITVGKTCTLSKQMLLQV

LNGTYGLLNMLYSMH

>SgreOR83

WVKAVFFMLDRTRLEELMSILVDSTKRFPEDGSGIRSKYHRRAAMLSLAWQAAPVLSLPTWMMIPMLERHYVTHGNITEV

YRRPMFYMLTPGNGLASPNYEIIYVVQCVAVLVAVEGSVLMDIYFVNIMIKVTSELELLNVNLVRMRLCTPAQRQIQSVG

NIAVPEHSHTRKGHSKATYFLNKTNTQAGEELHDSAPIYESTNGASEEFSKLYKSLVKNVRHHQQIIVCVDELEAAMTKS

ITTVLAVSVFSICLQAFGFVEMFQGVVSQATVVKRMMSFLAYMTHNALFCFLGQSI

>SgreOR78

TLLAFVASQVSAARHFWGDLLNVTMSTCLMFTYSMAIVKVIVFLRMRSSAEELIRELNSSMERYGRQVGGAAGVYAWCTR

RSRLVSLSHVGMGAVAYCLWVAVPAAMGRRCRSADCRATTALPAVVWCPFPFTESPAYEVVYAAVSVAMFYGYILTTTLD

AFFFTLIIYTAGQLRVLNLMARDLCARGSLRQMNARLADCVSYHIHIERCVQRLGALVGPIVLGQFLADIVTISAATFVA

TVS

>SgreOR82

CATTAILLSTAAANFLITVCSALKFYLDPPEETEKASQVSFLLTVSIPNTVKGISLTRQRTRLQQVVVWLLVMRRAVQDG

AGRRRLYARSVNLVGKMWMVMALMGGMIWAVDPLLSQPLPLNGTSPDPALPLPVWLPFDGSAPDTYSVTFALEAIVFGWV

AFVAICVDVLYITLIINFAAELHVLNDNVEITGNGADVAGHSNRKIGEVRHVGASRAGATHDGPAVIPNFPSAQSAGAKP

LIPAYIPGHCDVERSDGNDTYRLLVKNIQHHQLIIKCIDEFQKATGTPVLFIVAINVLNLCSSIISLAALLEKDPRSSTV

VKSLIGCLGFVIETAIYCLPGQ

>SgreOR61

LNARLAQLHLYSGGGKLRITATRVLRKVTTEDRDDMYDELCLCIDTHQEIMRLISFLDSVMNPIVLTQFMLSVMAACLTL

YQQTYSPDGNSVMKSASYLPTPGIEVFVYCWGAHSIREQGEAVSEAAYSCSWFDGSPRFKRALRMVMCRAHKPLVVTAGR

LYAINRATFLSLVNASYSYFALLSRIHNR

>SgreOR28

MGLFYGCIIFTSQDGFFWSLIIYVGAHLRFLNFMVTNMSSGESDTKSPGGSPQLEEKMRRRLKECICYHNDIDRCVQRLS

SLLGPVMLGQFLTDIVTISASAFVATMLKADSGWLLKYGSYLSGTIEHMFLFCWFGNDILTESERLQLSAYSSAWADASP

RFRKELRIFLCRSQRPLILTASKFCAISRQTFLRLMNASYSYFALLNQLSSE

>SgreOR68

HLEETTLVFIFTSTCSCGIIKMVFFVRNRQSYSLMAREVAGLIALQNGASSKDPALAAILHDSRKHAFRITMGMLLFMFS

QCFIWYPIPIVAHAGERRLPFSQHGWDNNSNFYELSYTLQCVSGLYMSQISFGLDCLFASIMILVTAQLKILRRRILKLH

KEVIPADGNDSFRRWDQMPADKCYDNMYERLCLCIDSHQHILRFVKHLQVTMSPIAMTQFASSVVIACMALFQSTYGEDI

SAALKCASYLPIPGGQVYLYCWAANSVTENGESVSTAAYSCSWVECSARFKHALRILIVRAQKPLVLTAGHLYPINRGAF

LSLLNASYSYYALLGQMNKR

>SgreOR42

MAVLETFARQRLGATREPWDNVLRRNVKVLRFGGVWRPASKSGWRHCAFPLYFASVCGSLLNIITLDIVRSWLLWGDMTE

VTFALVSAMTNVNGVFKMVHCFRHCETYSRLVSELNGLVELQRPYCEGNGDLMAAFRKACRRAARLTIGCLTYMNVLGQM

WCVVPLVSHVPPGSRESPLPLVSLPGLHRENRGWYLFAYLVECHAVFYWNFASLGMDMFFASIMIQVTGQLNILNIRLTQ

LRQEGSTEDQAQLPRSTGFNLSRNDQHQQRSANDFTRMYSELCECVKHHQAILKYLEFLERVMSPVVLTQFLCSVVAVCV

TLYQITFNPEGSGVIKCAMFLPIPALQIFVYCWCGHDIMEAGLSVSLAAYSCAWVGVGQRVTSALRIVMCRAQRPLQLTA

GKVYPVNRDTFLSLINASYTFYTLLRQMRNR

>SgreOR86

MEQPPADSDSMVVPGSTLRMVLGLWQPRGAAPSSLNRLLACVTLASITFLALCVALKLCADPPEELEQITLCGLVGSACV

GYFFKAGLFVAQGGTLRHTVRLLADTRLRFCSEDSSETIRRRYQNHSNIIYYLCQMVAVPAAIGWVLCPVLSRAVTKTDK

EHQEAHRQLPLPVWLPVDVYASPTYELLYVVQSFCSLVTSQSCLSVDSFFVHMMLMVAAELEVLNCNLSSMEHTKSTRTE

GERFFSGHERNGKRLAVLDSGQVVDQQTLPKNAAHGWLHRQLLKNVLHHQAILRSVSLLQSAMSVSIFSLLFINMATLCS

SLFVAAVLLQRDGNIGKAMNALFSIPGLLYETTIFCIYAHIMTDQSEKLMYSAFSCGWVNSDARFK

>SgreOR57

AETASGRSLVSAMEKQKGRSRQLKDTGSAVTEDLTWDEVNCSVLGTNIRLLYYMGLWPLGSSRAYHAYTAFSLSFSAAAI

LMKVVGACYNLSDIDQVTGALSTILPMSGGLANGLFLMRRRPAICRMVRSIDRLVDQQR

>SgreOR60

VGALLQLLGQRVSSLRVSSDGDSEDCASDEGRHILYAELCSCIQSHQKITKFLQDLEEAMSGMVLVQLSSTMVNLCTALY

QQTKIKDVSSALQYAMLLPFHTAQSTLLLAAHTVTEQAQMISAAAYGCSWVEESERFKHAMMILINRAQKPLVLTAGHLY

PIDKPAFLSLVNASYSYYALLGQMNKR

>SgreOR4

ACNMAVSGKDLQTNVLASGIPCLDLIVYLEPILRPLATTGMWSSSVFSNNVLKVVAMTAGLSLFGMFLVQLLAEVVGTPL

LLAANGDIDRLIHDMSVLGIHLDTFWKWLFMLTQRRRFIQLLRQMESCMCLGVLACPTDRLALQDGYLQGQGIQRWAVPT

KLYEVLERTRFRYWMVTVVWSVLCVLGASHWYFVPLLKGDDSLRYDAWYPFDSQQPTIKKMVYWMQYACSMYSVMILCFF

DCLLVWLQQLLCVQLRYLAGNLRELSRSGGAFSVDRRQTLAVCISHHEHILRVMRELNAFVAPLLFLQCFKNMMILCLVA

FLASM

>SgreOR36

LNVRITQIREDIYRNGHVSESPAGTRDEGTEEDSAWRELCECVEHHKDVIKLVGDLEGLANIVILSQFMGATIIICVTLF

VITTSKQHFAALVKLDGYLVVVVYEIFMYCWFGDDIMYQNSRLVNSVYTCGWPGAPQKLQKALIIILLRAQRPLGVTAGK

FYHVSRRTFVSLLKASYSYYALLNQMNK

>SgreOR71

MNDNLSWTASGKSVLKLNIRHLWVLGIWELGETSLFTLQSIIAFGMGAFIIVDRAVAVYFIWGNFELTTLVLLITFTGGS

GVLKMAVFVYNRRQYHTLANQLDELLSLQRGSCSEDPDLAAILVSSHRKAARLTKGLLLLMLSQTLLWISVPLVAYPEES

RLPFVQHQWDNNNHFYGISYIVQCLSAVWVSQISLGVDCLFAAVMILVAAQLEILVNRLLNVRNEVDLMDKETAVFEKKE

LDSKSDQNMYDDLCICIKTHQNILRFVACLQDTMSPIAMTQFVMSVVIACMALFQATYSEEFSAVLKCASFLTIPVGQLY

LYCWAATNVTAQAEAVSAAIYSCSWVDASERFKRALRIIISRSQKPLVLTAGHLYPIDKEAFLTLVNASYSYYALLSQMY

NR

>SgreOR87

QELEQLTLCCLVVSICVGFLCKVALFVAQGSTMRESVRLLEDARTQFCNGDNNQLTRRRYQKLSDNIYYYYQVVAVPAVI

GWVLCPLLSSSVTMTDQDQQEARRQLPMPVWLPFDINKSPTYEFLYVAQSICSLVTSQSCLSVDIFFVYMMLMAAAEFEV

LNDNVSAMEYTNLKTARTEPEEFISKYKRSGGNLALPQNGQDLGEETYIENAKHTWLHQQLLKNVLHHQKILRSVSLLQS

GMNVSIFCLLFINMANLCSSLFVAAVLLQRDGSVGKALNALSTIPVQLYETTIYCIYAHILTDQSDRLMYSAF

>SgreOR45

MAQTNRKMPPANVLATNISILRWSGLWPPERQTGWARLFTAYTAVAFLSQVVAIDMTLYHIYNSEGDIYEITLTMMVTMT

LVGGVLKMLHFFANADTYSRLVRDLQEVIDLQRGNCERDDAVSAIFCSYHRQAILFTCGSLGYLNVLAPTWFLMPVITGV

ATDPNDRKLPFSQLKGLRSDDLIGYSAAYFVQCHAIFYWNFISVGLDVFFATAMLHAAAQLKILSHRLSRLGKQTTRYEE

QPPYWNALQGDAAPVRQIITYEEENDLYLQLRNCIKNHQEILRLVLFLETVMGPVAFIQFLCSVVAACVALFQATFNAEG

NGVLKCTMYLPTPAFQIFIYCWCGHEIMEEGLYVSRAAYSSGWVGASQRFTRALRIVMCRAQRPLLLTAGKLYPVNRLTF

VSLINASYTFYALLRQMRDR

>SgreOR26

RRRASSQLYGTYSGCVVVILLLFVASLLFAMVHYWGHMLGVTMNACLMFTYVMNSIKIVAFLRMRPAIDQFIDELDSCMQ

EYGQELRSQKAAVFRWTALKSRIVSVARLSVTAMGCVYWAVMPAVRASACGHTVEcRASVGLPAHVWYPFSYTVSPVYEV

IYAAVAAGLMYGALLSSIMDGFLVSLFIYMAGHLQLLNLMLRSISGDHEEDDEDGDHHGKQRRDVAAAGPFLQEQLMRWR

LAQCVTYHCRIDMGVQRLSTLFGPILLGQFMMDIIAISATAFVAIAKNADSTWLVKYTSYLSAVIQQLLFYCWFGTDVLT

ESERLQMSAYSCNWMDASPRFRMELRIFLCRTHRPMRLTASKFYTISKETFLMLMNASVSYFAVLREINAK

>SgreOR30

AFSPLRAAGRAAILREARRRALLMFCVFFGIGAVALSLFYAGPALQNLQDAEVLASAAGNGTLERHLGRNLPMLIGWYGG

QPVRTPYYQLSYALICYWFMLIYLSTSTLDAFYVTLIIYLSSQLKMLNAALADTVQLPGLSEAATVGDQRPAASAARQGD

YRRLVQSVLFHQEIIKSVEEMESLLSPSVLAQFATSTLVICFTAFAVMTSTKRQEMPAYATYLATMFYELFMYCWYGNEL

LEQSDALQLSAYSCAWPGAGGRFQRSLCILMARVQRPLCLTAAKLYKISRETFLVLLKGSYTYFALLHQMNDPQHEA

>SgreOR11

MPLTRGSATSLLCYRLYTVGVIVLNLSFFASTVIVLYSEGTADLDVFTLTLSVADTNGTWLFRMAHTFVCERAFHKLSHQ

VGDDFAEFLTWDDIPMLRAQCRVMRRFTLTYIWFGIIACAYYLVSPVSAEGLPFILALPIDAMQPVGFAVTWLYCTVVTL

HAVVMTMVLDSFNVSLISQLRVQLTLLNKKIVSLAREMSERTDNSLETTDHAELHYRLQKCILHHQAIIKNADLLESSLA

GMLLAQSMSIGASTCFQMFQLATRTNGMQQTGKSGSYLIAMLAELFVYCYFGDDLINESENVALAAYDAVTSLQECPLSI

KRSLLLLMHRAQRPLRITAGGFFPLSRES

>SgreOR3

PGQPLWATIQPLVWSCLLMHLLCELADIGLNITDVQQLGKNLPVSSLVGGSWYRLSYFTLRRDAYCRLVSKVGESFRRGA

PGRMRRWLRRSRAYTLVYFVYGTIVCLFWLGHPLLIQQTTHTMFSTNSTNHSRMSETAEFPSGAWYPFDVRERRVYGAVY

AFQCLAIYFAGMLIMVTDIMFITLMLLTCGQFEELGDKLRNCWEIAAARTLRRAGTTQERELHKVVAHCVQYHDMLLRIV

GDIEDLHWTSMLVNFVLQLIILSFSAFEATASADLASPLKGTNLVTYLVLAIFQLFLLCRCGDKLMEAEEAVSSAVYESQ

WFEAPQSAKRSLGVIVMRSRLPQRVTVGKLVGLNLVTFSETLSRAFSYFTVLRQIRTSN

>SgreOR62

LAVGNAIENVLGVWKNWGDLTEVTYSLLNAFTIGAGVAKACHLMLHEERYCLLVRRVDRLARSQGVYCDADAGMLAVAVG

CRGTARRVTRAAFAYLTVLCLIWMFMPLVAHPGERQLPFNHIPWEPQSMPLFYELSYAVQSASSVIYVFISFAFDCFFAV

VMILVTEQLMVLNLRIKQLYAPTAGDGPIKKEKQFPDKTVTDRHEVMYKELCLCIDTHEDIIRLVSFLDSVMHPIALTQF

MLSVMAACVTLFLESYSPDSSSVLNSVSYLPTPGIQVYLYCWSAHNVLEEGAAVSEAAYGCSWYDGSPRFKRALRIVMCR

AQKPLVVTAGKLYPISRATFVSLVNASYTYYALLSRVHNRG

>SgreOR33

GMWRPPWIQPKWYLLYRAWVLFTLFSFLVAQIQALWHFWGNIDKITHDTCLMISIILSLIKFFSFVLRQEEFFRMVRRID

GISAEQKKTGDSETISILEASYRSARAVTLYMTFLGGSMPGVWATIPSIMRKLGVFPPERELPATAWYSSRDTQTPYYEI

LSTLQYFSMQYSFFTAVGPDLLFVSMIIHAAGQLEVLNARLRRVGKMNGKRCTSSSKQQKTEEVLSLEVSSGEVMWKDLC

SCIEHHRDVIELIKGVERLVSKTVLLQFMGATVIICVTLYQSSTNTENIAALLMLQGYLGLIMYEVFMYCWYAEDILYQN

SRLADSAYSCGWPGAPLKLQRALVIVICRTQKPLGLTAGKFYYVSRETFVRLMSASYSYYALLRQVNDK

>SgreOR75

VRYLNGVMSPIAMTQFVFSVLVGCLVLFQATYSTDITTVIKCISFLPIPGGQVYLYCWAAHLIIDQADAVSTAAYCCSWV

DADPRFKRAMRILISRGQKPLLLTAGYIYPINRGAFLSLVNASYSYYALLGQMNKRSAS

>SgreOR6

LTLGCVACCSGVGCVWAAGPLLADGDALPYRLWSPLDVTAAPGYHITYLLSVTILAPLVEAFVNLNALYLGLMVLTSARF

SSLNSRFAAISKEKDGSGRLATAELARCVALHVDLLRFCNELSDVLSPVLLSQFGFTIVIICQTALQTTMELELSPRTMK

FMGLLWLGISELFLYSYMGHKIMSQASDVQLSAYSCGWPDLDSSFKKSVSFIIQRAQKPLHFSIGKMYVLSLRTFVEMMR

VSYAFFTLLQQVNGD

>SgreOR92

FSALKLIMDTPSELEEITLCCFVMSIAPGFLTKAVLFIYQGDTLKHLLKLMSGIRRLYRNNKRSDDIRHSYQILSGRVYL

YMQVAVVPAYAGWVSIPLLARFFFNSDDDSIESNHQFPVPLWFPGNIYVAPTYEILYGIQSFCILVTAQSAICIDVFFFH

VMLMVAAELQVLNENISVMQKLNVKIQGYEDYRFKFYGNNYELTFPRNALPISPIYSRSAGASDNKMCHHLVKNIRHHQE

IIRCFHVLQSVMNVSIFTLLFVNMADLCSCMFVTAVLLQRDGNVTKALKPMLTIPPLLYETGIYCIFGQIITDQSEKLMD

SAISSGWVDCDPRFKHDLLFFLMAAKKPLEITV

>SgreOR21

VALLLFICSQAVLLWRDGAADLERFTLSLSVLDTTSVFMIRLRHIAALEADFHRLAMQERRDFGEFLSADDLRLVHLRSR

SIRRVVSAYLLSGVFTSIVYAATPISAEGLPFILALPYDATRPLAFAATWLYSAYIVFSVDIGTMAADSFNITLMVQLRN

QLDLLSRNLRSLNDIVSPTKPTSFQTHSIKNIRSSEDLSHDIHYRLRKSVLHHQAIIRNVELLEQCLGRMLLGQSLSIGV

CFCFQLFQTAKRSNGVQEVGKTSSYLVIVLSLLFVYCWYGDDLISESEKVALSAYDAVTSLQECPTSTKRSLLLLMLRAQ

RPLRITAGGFFSLSRESFVSVLNVSYSFFTILRNFNDE

>SgreOR49

MGCDLEETEPLTWQYTAHSVLKYDLRILHLLCLWPLPGSLLFRTLTAFFTALCLGHIAEAGVNLCTLSGDMEAYTLALSA

VSVVIVGVLKVTFFLRHERKYCRLVRWLDALVAAEREAVHGRPLLEAIFPAVQKRAVRIARGLLLYNCFLLAIWLTVPLA

APPEARRLPLQQLPFTDENAYPLYELSYALQALSIIFIGLINVHMDSFFTVAMIYTAALLRSLALRLADLQAHDTLSRAK

GNGRGQKTATADEMYGELCFCIRTHQEITRFVQHLESVMNPIAMMQLALGVFDACMLIFPAAYSPERGALLKCLVSGPTV

AMQILLYCLGAHSVREQGESVSLAAYNCGWPDASARFTRAVQLIINRAQQPLALTAGGVYPIQRATFLSLLNAGYSYYAV

LQNFNGR

>SgreOR29

AERMAKAKDLPDPVVDLRWMSRALAPVGMWGPSGGSRLYDAYSLWTIVQLVLTAAGQVAGLQGHWDDLQTVFTTLCFAFT

VTCTIIKGTIFVLQRESMDALKRHIERNAVEFCSHLPEERRSLLVRARNLSRLIVCSFQSVGGVTLVSFITGPIVQNGRD

RELLASGLNNATLDRRLGHNYPMLMWWFGGLPVTSPGYEAAYIIMCYWLVLMYICTNVPDAYYVGLINYITAQLRLLHIA

LPKMATLEPDAVLQEKLAHSYGLTIKADVPADAGAQDVIQDRVYARLVECIRFHQEIMKCVDEMESVLSLTVLIQFFTST

LVICLTAITVISTETAYLPTYAAYLATMFYQLFIYCWYGGEVYLESESLQFSAYSCNWPETDARFRKTLRICLARMQRPI

SLTACKFYKLSRETFLLLLNGSYSYFTLLLQMNQKND

>SgreOR2

NADEPSRYMEFNILMVRSMGVLVNRGMLGVLLPWSLLALLLSQGLAGVLDLYENHSDIADITANLPVTTIVFSSAYRLFF

FTLHRDRYQAIVDTVAERFVASSGSINMAPWLRRSRIISILYFTYGSFVASTWQLHPLISAQLASAEMRKESNATFEGLP

RELWEFPFRAQYPFDARQPYVYTVVFLLQGACIFVCGCIIVVQDMMFITLTLLICGQLEILKDKLRNMRSIATDQRQNED

IGLVGKALEKQVQERRKMILNSSTDITKKDENFNEDLVTKKINLLLGECVEHHNMLLSMISEIEFMHWSAFMINFCVLLI

ILSFAAFEVTSGIPTSPAKVVNLAEYLLVSILQMFLLCDCGDKLVDQELSVSQAAYESEWYHCSESVKRTLQIIVLRTRQ

PEQLTVGKIAGLNLETFSDMLSRSFSYFTVLRQIRDG

>SgreOR70

MKDNLSWAASGNSVLKLNIRHLSLLGVWELGESRLLKFQSTFAFGLGLWGTVECVLAVYCIWGDLEQTTLVLLITFTCGS

GVAKMAVFLYDRRQYNSLAHQLDKLLSLQSDSCSEDPNLAAISEWSRRKAARLTLGLLLFMLSQSFLWNFVPLLVYPEER

RLPYVQHQWNNNSLYELSYAVQGLSAIWVSQISFGVDCLFAVVMILVAAQLEILGQRLVNLKNDVGIVGEDKSVSEKKER

DSKTGETMYDDLCLCIETHQEILRFVAYLQDIMSPIAMTQFALSVVIACMALFQATFSEEFSAVLKCASFLPIPGGQVYL

YCWAANNVTAQAEAVSVAAYNSSWVDASERFKRALRIIVSRSQKPLVLTAGHLYPINREAFLTLVNASYSYYALLSQMNS

R

>SgreOR101

WGLAPLLSCAFYSANSSVPCGKTPFPVWLPPALQVSPGYEVMYITQVSSHLVTTSTTTGVNLFFINLMLMIAAELDVLNE

NISDVRKATGKPAGGAVEEGAVASYVFTADRRTSTQNGFGRRDISDGVQSSGSVQRHTSGDIIIDPDDEMYQQLVVNIRH

HQYMIESVTLLQTAMDYPVFILLFMNMSTICANILIIFVNGMNPASALKSVVASLFFISETAIYCCFGHIIVQQSERLTN

SAFSCNWPDGSARFKRSVLIFMLRASQPMEITVGKTYSLSRQTLLQVLNGAYTLFNMLYRLNSTK

>SgreOR56

NMYNQLCQCIQTHQSLLRFVSFLDSVMNPIVMTQFTFGVMVAGVTLFQATFSPSNSTIFKCVTWLPMPSTQIFLYCWGAH

DIMDQGEAVSEALYSCGWMEAPARFRRAMRIVMCCAQRPLSLTAGRVYAINRA

>SgreOR93

MELVKREEMQTLLGPGATVRRLMGLWWPRRGRGRARSAVCAAVSLVSLAWLPTFSGLKLIIDPPPELEEIAMCYLLIFAC

TGFLTKAAFFIYKGNTMKELLQLLSDTRRFYCDGEISEVIRSSYLQQSRRVYRYMQGAICLAMVFWVSTPILVRAFLSSD

EDSPESYRLFPVPLWFPGNIYRSPTYEILYGVQSFSVLVAAQSTKTNAKPKTYGDQESRIIIDGKTKELAFRHNGHFTRK

ALSEEDSDEKMRLQLVKNIHHHQVILRSVLLLQNIMTVSIFILLFVNMVDLCTCIFVGAVLLQRDGNVTKALKPLSTVPP

LLYETGLFCIFGQILTDQSEKLADSAFSCGWVDCDDRFKRDLLFLLMSARKPLEITVGKMSKLSKQMFVQVLNGSYGLLN

LLYHFQSIQ

>SgreOR80

DHLLRPLHWAAVLRHPRRAAGSPLCFRLCSIAMASFAFSSTCSEVTVLFRDGTADLDVFTLTLSVADTNTIWLFRMAHIV

MCERAYHRLTGQVGHDFSEFMTWDDIPVLRSQCRLVRRFTLTYIWFGIGACACYLVSPASAEGLPFIMVLPYDVTQPLSF

AATWLFCTITCLHVVVMTMVLDSLNVSLISQLRVQLILLNKKIVSLAKEMSAKSVNSSETSVSPDLHYRLDKCVRHHQAI

IKNADLLERSLGAMLLAQSLAIGAAICFQMFQIATSANGLQQTGKFGCYLFAMLAELFVYCYFGDDLITESENVALAAYD

AVTSLQECPLSIKRSLLLLMHRAQRPLRITAGGFFPLSRE

>SgreOR96

GVWWCQGRRGRFCSTAAASVTLSTFAWLSIFAALKLVKETQQQLEDISLCCFVIVICFGYFSKVAFFIYKGGALRELLQL

LSDARGNYGNGKTSQSIRLRNQKLAHRLYIFMQVALLQGLAAWFSTPLLARAFLTSDEDSLESARQLPVPFWFPGNMYLS

PTYEILYISQAFCQLVVTQSATCVDAFFIHIMLVVAAELEVLNENITAMQTVRIKLQGTEYQEHNLRIVAKHGEPTAYVA

SQCTPDEMYLQMVKNIQHHYVILRSVSLLQRIMNVSIFILLFVNMADLCSCIFVSAVVLQRDGNITKALKPLMTIPPILY

ETFLYCIVGQILTDQSEKLAYSAVNCGWADSDARFKRSLLVFMTVAGRPLEITVG

>SgreOR31

NSGTALRSGEMADYVHRKPVVEIGLSKAALVAIGLWAPSRPSRLYWVYTRWSLLQLVVNIVGQVAGLQGHWDNLPVVSTS

LCFVLTITCTFIKAVIFVIQRPRVDALMAHLERNLAMFCSFMPRVKDAILRSCRLRVLFMFTSFMGIGGITLLSFYTGPI

VQNFKDAKLLADEASNVTMRPHMGRNLPMLMWWYNGQPEQTPYYQLTYVLMCYWLMLIYLACDVQDAFYVTLIIYLSAQL

TMLNAALTNALHPGDGEDAHTSSERRQLPSRGWRGDAESVVPTKRSGRDRLVECIRFHQEIIKSAAEMESILSASVLMQF

MTSTLVICFTAFTVITAERAYLHTYITYLATMFYQLFLYCWYGGELLLESEKLQLSAYSCAWPDADPSLKRSLRIMMARL

QIPVKLTACKLYGLTRETFLLLMNGSYSYFTLLHRMNQAPA

>SgreOR40

KGDRNYTRNSTASVLSSSFGRNGVLEPASTMNSKIHSEAEMYNSFVRCIQQHQEIISFAEELEKLMNDVLLVDFLCCMVV

TCSTLYVSTSANENFGDLLSHFGYLVAMTYPLLLYCLSAHEIKDQSQQVSMSAYCSPWHQATKGYRRALCVVMCRAQRPL

TLTAGKFYVVSRATFLTLMNASYSYYNILREINDSKRSD

>SgreOR63

LACAYIGAVLLASLANMAEAALGIAFGEASMDEITLVLPNTLTTACGVVKLGFFLRDHRRYYALVRRTERLVMSHEELAG

GAAEACARDVSRRVRAFALFVVALICVQGAVWCPMPLIAYPGERKLPFGQLPSTNYTQVPVYELSYVLQCMSSFTIANVT

LGFDCLFMGIMEFLGAQLDLLTLRLRSLRLDAALCTTGQKDGRGCAVQATKGHDSAHRELCLCIERHQEIIRFTRYLEET

MSPVAMTQFMFSVLSVCVLLFQATYSDDFSAVFRCVAFLPLPGGQMYIYCWAAHRITEKAEALSMAAYSCQWVESSQCFK

RSLAIVILRAQQQLVITAGHLYPVNRTTFVSLVNASYSYYALLGQINKR

>SgreOR53

VAHIAVAAVGIWYRPGDLAEVTIGLANVFVIFTALSKSLLFVSRRPLFYALARRVDLMTVEQQAFCTSDPTLQHAVSIAR

RRAGRLSVFFHWYVLVADILWSLIPLVQASKEKRWPFQQMPLGTWERSPFYELSYLLQCASTVFFSLISVDVDCFFVAVM

IHITVQLRILTSRFATVGTGMNVTVNELHCQASRLKDAIHEQLRACVETHQNLLRLVSFLNVVMSPVAMLQLAVGAVSSC

MVLFPATYSTDNAAAMKCWAALPVLGIQLYLYCSTAHHLKDQGEAVSSAVYCCAWPESGCRLQRSLLLVMCRAQRPLVLT

AGLMFPINRATFLSLVNATYSYYTVLKHVNNR

>SgreOR9

YIKELEDVYYIIMLWVFLPMMAVMCLIMFALLTITSVDIEFLEMLAFFLIYFITNGVISICGSMLTSKAESVVLAAYSSA

WPERGRRFSGAVRVVMVRFLQPAELTVAKFVPLSINTFSKLLQESFSYLMVMLSLVNEKDNETQPAAILEPASNHSAYH

>SgreOR67

MAKGAEEEIDRPLTWAESGQSILKLNIRHLWLCGVWPLPGCWLYDAYAIMGLIVGAVNAVESAVSLYFYWGDMEETTLLL

TSSVSNGCGTVKMAVFMRNQRQYFAMARRVEMMMSMQDEYTSEDPALDEIQQVAHKRGYRLTLFWTLLMFSQYFVWYPMP

FYAHPGERRLPFAQHAWDNNTNQYALSYFLQCAASARGTQLSWSLDLLFVSVMLLVAAQLEILTRRITSLKEESHEGKAA

AVGEKSVKGMAANIYDNMYDNLRLCIETHQKLLGFIRYLDDTMSSVVMIQFCVSVLLVCVALFQATYSTDSTAVLRCALY

LPMPGSQVFLYCWAAHSVTEQAEAVSLAAYSCSWVGTARRIKQSLRIIISRAQKPLVLTAGHIYPIDREAFVSLVNASYS

YYALLGQMSKR

>SgreOR72

IAMSEFFFSVLVICLGLFQATFNEDFGAVFECASFLPIPCAHVFLYCWAANSVTVQAEALSVAAYSCSWMEASERFKRAM

RILISRAQKPLILTAGHLYPIDKKAFVSLVNASYSYYALLSQTNKR

>SgreOR55

ERRRTATWRRHHQLVTEDTDSNSIWPQAMETSRNDHREMESGRCGEERVVTWRGAAGSVLKHNVRFLCLLGVWPACRGSA

YHAFSALVVALCGTHMAFVVLGVGKGSEDLQETTLALTNAFAVSGGATKVALLLRGRRRFYALVRRLDRLVAAQAEHRRA

DARRQAAFDAFHRKAVRLTFLLHSYLFLLSFVWFPMPLIAQRGERRLPFVQLSWLDTSQLAVYAATYTLQCAGTFLLAFA

TVSVDCFFVAIMVHVSVQLRILSSRINALGLRTNLAGSARTFESDVSGEASDIQRDMYRELRLCIQYHQDILRFVRYLDS

VMSPLAMTQFISGVLLACVALFQATL

>SgreOR59

RREATAAMAGALDKTKPWSEVDGQHGNRCTLAGHILYLRVAGLWPHAQGARYLLHTVMVQLCAVAYIAVGVASIYTARGD

VDGISHTLMHLLEVVSGMVKAGLFFSKRQSFYRLVQDLDLMVSEDWDRPELVSARRWARRMTVSLTAYIYTLILLWLPAP

LLAGGDQKLLPVVQIEGVDWSLWPGAYAALYALQCSVLLTQVPVVIGLDCFFVAAMLHVAALLQLLGQRISGLQVISGSV

ADLAGDVSLKRRQVLYAELCTCIVNHQKITKYLRNLEAAMSTMVLVQLSTNMICLCMGLYQQIQQGEALSEAAYSSHWVG

AGAGFQRALCIVMARAHKPLLITAGHLYPVNTAAFVALMKASYSYYTL

>SgreOR23

QLVVVGVVAVVVPCSDLFTVACVQRLGTLLDILERRVERLGVAGSPQSPGGSRHSPADPLHRALRDCVSLHQRIISQAEC

LNQAVGGALLPQVVASAAGICFLLFQVAKKTSFHVVETGKLLGYLTFMLSQLLFYCWFGDDMLSKSERVALAAYRCAWPG

APTHFQRSLLLVSMRARKPLTLRAGKFFVFSRQAFVQVMNVSYSYFTVLRSLSEA

>SgreOR8

MLDKRDTMALQEKRSLVQFGLNRRLLRCVGLWPEERGSPWRPSRWPNALLQQGSLAAMVTSELAALRQYWGGELSHTTIN

ACIILLVAVALCKASSLVSLRRPIIQLMHTLQDTSVPQNEWEERIYSGAARHARLLTLVLCVDYAIVAFIWDSLPLLNFM

QQSPESRYNNSDAIFSQYPIIALYPWEVQTGPAYAFTYTLQVMCGAIFTMTHLACDTFLMSLVIYICSQIDVLRASLQQL

GRRADRVGAAAAVAEAQADAELSVGRLGGGSDPVKALCGQGSEQELYQELVACIKHHKNIIGYVGVLQQVLSPVALAQFM

CSMVIICLSGFGIAISNDFGALCRYCVYFTGAAIQLLIFCWYGEVLITKSERVSEAAMGCGWTEVRGRHFKSSALILMIR

AQRPLALTGSKFYVISLKTFVQLLNASYSFFAVLRQLNESGHREEEGALASL

>SgreOR14

AVAAPATSSALTARSREIDPCIPDTMAAAGPAKASVAAVEAASDLGYLTRPLHWAAIMRHPRSVAGSVLLFRLRTLFFAT

VCFTFLCSQLTVMCRQGTTDLDVFTLTLSVADTTAIWLVRLLHIAMCERDFHRLALQVGQDFAEFLSPEDVPVLLSQGRS

VRRFTLTYISLGVLVTTYWLFTPVSPEGLPFFLALPYDASTPLGFAVSWVYCTVGTVHVVVMTMVLDSFNVSLINQLRMQ

LALLHNKIVSLPRENSSTPLDSLQTTGYWDLRYRLEKCVLHHQAIIRNSDLLEKSLKGMLLAQSLSIGAAACFQMFEVAM

SADGLQQIGKFGLYLTGMLAELFIYCWFGDDLITESENLAVAAYDAVTSLQGCPLSIKKSLLLLMHRAQRPLRITAGGFF

PFSRESFVAVVNVSYSFFAILRNF

>SgreOR54

MPAIKRQRRLPYQQLRWLDTSSAAVYGASYALQCTATFFCSFINTHLDVFFMAVMIHVADQFRILAARFAALRLDADCKP

ESRVLQDGSSRLGEELYRELSLCIQNHQDLVRLVHLLDDVMSPIAMTQFVVGAVNACMVLFPATYAETVSSAAYECCWLG

ADRKRRRALLLVMCRAQRPLQLTAGKLYPINRPTFLSLLQVTYSYYTLLQNLNSR

>SgreOR98

WAAFPLVQQLFSSNGEVARLLPLPFWLPLDIYTSPTYEVIYVVQVLLIPLSTTSLFSDFVFVDLMMRIAAELEILNYSIS

GLQNIKNSVSTKSKNEYKSVDRGSCDSIQLQLAKNVKHHQQILRCVVLLEEAMNTGVFILFLATTIALSSNIFAATA

>SgreOR19

MEPLASAASAAEDAANLDYLLWFLHWSATLRHPRTRAWSARIFYLRYIVCVMILVYSCSVHVVLMWQMGFDDFDYLTLAL

CLLDTYITWIARLGHIAKHERDFHRLARQVRDDFGEFMAPRDVPVLLSRSRTLRRFVLTYLSLGVCDVTMWLTFPMRGAG

LPLDVVLPFDTAHPVGWAAGWLYCAFITVHAIVMNMMVDAFNVSLMAQLSMQLVILGRKINDLANDAESMASSPPQTAFE

GDEQPRTFCSRMYNRYSSKKINDHNQRMNSLALDVETKEIRQSEELSSGSDVHCRLKKIILHHQTIIRNVEFLQQCLGDM

LLGQSLGLGIAICILLLQVGLSAQNVRETGKFGAYLCAMFTELSVYCWFGDQLMSESENVAFCAYDAVTSMQELPTSIKR

SLLLVMLRAQRPLCITAAGLFPFSRESFVSILNVSYSFFAILRNF

>SgreOR66

MARGVPEDIELPLSWAASGQSTLKLNIRHLWLFGAWPLSGSWFYDVYNAFCLAMGVWNAVESLLSLYFCWGDMDERTMVF

ISTFTNACGTAKMAFFMRNRRQYYALARSVEVLMSLQSEFCSADPALADIRRGSQKRGYRLTLGMLLLMFSQYPVWFPMP

IIAQPEDRRLPFAQHGWDNNTSYYELSYVVQCVLSTFATQLSNSVDLLFVTVMILTAAEMRILTLRIVSLKSENNEVGLV

KRDNVVIGQTENTCDDEMYEKLCQCIESHQKVIRFVKQLENSMSSIVMLQFYFSVLIICVTLFQATYSTDYSSVLKCACY

LPVPTGQIFLYCWGAHNITEQAEAVTLAAYSCSWLGASPRFKRALRILMCRAQKPLVITAAHLYPMSKDTFVSLVNASYS

FYALLGQMNRR

>SgreOR65

MKSREKGDTELPLSWSESERSVLKLNMRHLCLFGAWPLSRYRIFHVYSVINLALGIWYVNEASTYVSYIWGDMEEATLVL

ISTFNIGSIVVKTTLFLWYRGQYFALVRRVDDLMLVQSEACSQDPVLGHLLWRSKRTAARLTTAMLLLMVSQYVTWYPMP

LIMNWGERRLPLAQHPWDNNSNYYVLSYAAQCISVSWMTQISFGIDCLFVSIMILVAAQMKILASRVASLKMQDDELSRK

KHAIKCPRYKLYRDLCLCIETHQQLLRFIRELEDAMSPIVLTQFACSVLVTCVTLFPAVFSTDFTAVIRSAGFLPVPLGQ

PYLYCWSAHTVTEQAEAVSAAAYSCAWMEASESFKRALRILISRAQKPLVITAGHLYPINRPAFVSLVNASYTYYALLCQ

FNKRAKERAL

>SgreOR47

MDWDPQRSQPLTWQYTASSVLKYNVRFLNVSGLWPLQHLQLFRIFIATTVTLCLGHIAEAGINLCTLRGELEDYTLALSN

VSVIIVGMLKVAIFLRNEGSFCFLVRWLDALVERQTEYVRDQPSREAIFRGARQRASRISKGLDAYNLSLLTLWTVAPLI

ASTGDKRLPFQQLPMANTTTFPLYELSYALQGTSLIIICLINVHLDCFFTAVMILIGAQLKLVGSRIADLHPRNVAVKND

SMIDDTYKDLCLCIQTHQDITRFIKHLERVMNPIAMLQLALGVFNGCMLIFPAAYNAESGSLIKVLVTAPAVSMQLLVYC

LGAHSVREQGKLVSVAAYSCGWPDTDIKFQRALLLVMARAQKPLSLTAGGVYPIQRATFLSLLNAGYSYYAVLQNFTGR

>SgreOR13

LTLLSSKIVNLAKLLSERPERSSETTACREVHNRLEKCVRHHQAIIKNADLLEKTLGAMLLGQSISIGASTCFQMFQLAT

TANGLRQVGKSGCYLYAMLTELFVYCWFGDDLITESENVALAAYDAATLLQECPLSIKRSLL

>SgreOR37

PVVCWYGSWDTVTPYYEVLYVVQFVTIQGGYLVVMGSDLFFISLMIHAAAQLRILNMKLEKIVKNEDEWNGKGIEALTFI

SGYRISGLKGIEKCEGESKLTHYVTDQTSSYSELRVWVEQHKDVIKLVQRLEQLLNMIILFQFLGGTIIICVTLYQSSAK

TGEVTTMCKLQLYLSTMLSEIFMYCWYADGIVQQSARLARSAYSCGWPGAPQSLRRAVLIVMCRTQRPLSLTAGKFYTIS

RTTFVRLVNASYSYYALLRQMNDH

>SgreOR100

RQQMLGGRRQTAATLEEVQDLVSPNAAMLRLLGLWRSPRSDGEGTGGALAVVRGWLCCAMVGFASVTTGAKLCVDTPEDL

TALTDCGYSLLRLSAIAVKVACFILQRTTLQELVKQLADIRKIHGRRQANGRVRSFYQRRATIVYRSLQVLVMTVICGWV

TAPLLQHKKADEIPESQKVERQTPLPIWLPLDLQRSPTYEIIYVVQALCGTAAVQLSMLLDTSFYKLTLLVTAELQVLND

NLAVLGRADVSAERRGSFPTAVPGQERDGVDVPRIKQTAAPVTENNDRSRKLLYFQLVENVRHHQAIIKCFQLLQSALNY

SISILLLTNILTVCFAIFVASVMLQSDGGLRKAMKTITSIPTLLCETGMFCIFGQMVVDQSERLARTAYSCNWVDADARF

KRALLLLVTRASQPLEFSVGKLIKLSRETFLKILNSSYTLISLLYQFQVPKD

>SgreOR43

MVAIGSRTKPILVQGSKYSNKGQEDGASPSENVLWRNIRVLRLAGVWRPEGRCLARLYPLYFGTVCTSMLHIGALAILRS

YTIWGNMTEVTFALVSGLTCFNGAVKMIHHYSHSESYYRLVDELNILIDRQRPLCEGDAELAAALHAAYEKAKRLTFGVL

GYMFVLGQMWATVPLFMQFPPDDPSSPLPLVTITRVHKVHNHTLYSLAYLSECHTVLYWNFSSLGMDVFFGSVMIHVTGQ

LNILNIRLSRLSDGGAAEDLAHSSNFAKGSQQQKWNTHDSVSMYDEVRNCVKDHQEILRYLDFLENLMNPVPLAQFLLCV

GGICLTLYQITFNPDDGGVIECILFLPIPALQIFIYCWAGHGIMEESEYVSFAVYSCRWPGADRRVTNLLRIVMSRAQKA

SFLTAGKVHPINRDTFLSLLNASYSFYTLLRQMKNLEEGNEASP

>SgreOR58

LLGQRISGLRLSGDGAVDLAGDRGHSTRQMMYVELCSCIRSHQKITKFLRDLEAAMSTMVLVQLSTTMVGLCMTLYQQIQ

IEDALTALQYALILPFYSAEIYFYCWTADVITQQGEALSLAAYSSHWVGAGTSLQRALSIVMARAQKPLLITAGHLYPVN

TAAFVALMKASYSYYTLLRQLDGD

>SgreOR69

WLSQISYGIDCLFASVMLLVAAQLEILAGRIVALGKVANAAGCRPDESLAANTRNQTYRDLCVCVEAHQKVLRFVTHLET

TMSPIAMTQFFFSVVVACMALFQATYSKDFTAVFRCVAFMPIPCGQVLLYCWAAHNVTEQLVNASYSYYAMLGQMNRRSA

QA

>SgreOR52

RGVRFARNFLTDCWNNGCNCAFPAILHFLTPMMSWHSQQDEPLTWQYTAASILKFDVRILHAIGVWALPVTRVYRAYTGA

ILVLVVAYSVEAVIHIRLARFNLDDVTLAVSTYAVIVSSACKLLSFLHHEPGYWRLVRWLDALVAEQKQFCEERPRLQAI

FDRAQKRATRFTHALRIYNTSLILAWVFIPLMAPPGQRPLPFQQLPLSETDDFPLYIASYLLQSVCMLYMSLVSGCLDGF

FTAVMIHTAAQFRILAWRIAELRQNNEERKQQARSDVYEKSRKAAATDNVYEELRLCIRTHQEITSFAAHLESVMKSIAA

LQLVTGVTNGCLMIFPTAASSESGALLKCVACVPTISAQVLIYCLGAHSVQEQLLNAGYSYYALLKNFNSR

>SgreOR79

LQGLGKWAFMVARRRRYTRLVDRLQHCFRLSGLADTNDSASVLATSQTAQKVAVTRLQQQLSEcRRLSMWATAFWLWVCV

YGVTHWCLVPLIIGDNSLPFDALYLFDASQPPLRQVVHCLQYVAGLQNILLSVFFDLFVLWLHLLLCAQLRYLADNLR

>SgreOR15

VFSELLVLYHQGTEDLDLFILILGIADTIAMWVFRMAHITLHESDFHKLALQVSQDFADFVTWEDVPVMRRRCRTVRRIS

LTYMCFGLSNCAYFLSLPSSPDGLPYVLALPFDATKPASHTVAWLYCMIVTVHTVIMTIAINSFNISVITQLCIQLTQLN

KKIVSLNKDMPEEPGQSGKKPARRDLYCRLKQCILHHQAIIKNVDLLQSCLGVLLLGQSLSVGTAACFEMFQIATSADGL

QQIGKFGTHLFTILAELFIYCWFGDDLITESENL

>SgreOR38

RSLPTIAWYPYDETETPFFELTYVLQGISTFYCCITNVGLNVFLVSLIIYVSDELKNLNDSISSINYSTSHNCNCSYGKQ

HGLLYRSSKIVADDGERDIDTYKSHSKNLRQRTQFCCVVKAQQYLWMCLQYHQELIKTVKKLETTMTGVVFIEFVAGIIV

TCLTLFHAAVNAGNMALFLKFVMYLLYMTVGMFIYCWYGQDLMEKSEDLKWAAYSCNWQGAPRSFTDLLKIVMLWAQQPL

ILSAGKYYRISLKTFVTLLNASYSYYAVLRQINDTEK

>SgreOR46

RKCRPIHISGARTGTVNGGGKEGAQKCNSLRFWPFFVMDSDTQGSKSLTWQYTADSILKYDVRFLSVCGLWPLQGSRLFR

IFTVTIITLCLGHICEASINLCTLRGELEDYTLALSNVSVIIVGVLKVAIFLRDEGSLCRLVRWLDALVKRQTEYVRGQP

CREAIFREARQRASRISKGLDAYNVSLLFVWILAPLLASPGDKRLPLQQLPMANTTTFPLYELSYALQGISLTFIALINV

HMDCFFTVVMIHIAAQLKIMASRIADLHLTRYGGENSLTESKLGKNSLVMEDLYKKLCLCIHTHQDITRFIVHLEKVMNP

IAMMQLALGVFNGCMLIFPAAYSAESESLLKILVSAPVVSTQLLLYCLGAHSVREQGELVSLAAYSCGWPD

>SgreOR44

HLACLSCCSHISQEQSTPMAELKSGRGPTTGVCADDVLRHNVKLLTLGGAWPPSERSGLRRLFPLYTATIYFCQSATIAM

GIWLTYDLWGDVDEIMLTYVNTFTLLGGYIKLIYFSRDVRGYRELVSILRDVAREQWPYCENDAKLMAIFTGAYRKGLWL

TFGPLVYLNILGPTWFFMPLILRAFGSEERLLPFVNLRESVTNIFPLYVAIYVVQVYCMFFWNIISVGLDMFFVTSMVHV

AAQLKILNERLSNLGEDPPHDDYGAVLDRQIMRGFVNPQKWPFSRDGRRRDMYEELRNCIKTHQHILSSLKTLQRVMSPV

AMTQFLCSASGACITLFQATFNPEGNSTLKCLIFLPLPAFQIFIYCWAGHEIVYQEELLSVSGYGSAWVGASRRISVVLH

ILMCNAQKPLRLTAGKFYPVNRDTFVTLINASYTFYTLMRQTRDQGSTVQT

>SgreOR77

VNFVMSKLPGAGRPRTELRLQQKALRVAGVWAPTGGKASLAYGAYSGFVVLSVVVFLASQLSAMLHFWGDILSVTTNACV

TFTYAMSTFKFIVLLKMRPLTNKLIEELDRCMECYGRTYAHQKASIFDTCARRSRRVSQMQLFVGVSIYVVWEVVPILRV

QSCSSTEcRIQSGFPALVWYPFSFTEAPLYQVVFAVVSTGLFYGCFISVSLDGAFCSLMIYVAGHLRFLNFMARNMCRAE

KNGSRNRTPHGRHMTSATDPDEEMRGRLRECVRYHNDIERLIQRLSALVGPIMLGQFLADMITVSVSAFVTTTLSERLQV

SV

>SgreOR5

TDEHKKLLVACVAHHSDLLSTIQELNSCAGPSFFLQCFENTIRMCMIAFMATTTVADQMQVWSSAQFFLAAVGQLFLYCW

CGQQISHLTESISDAAYDSGWEEHDVSTQKNVAFIMQRAQKTIVFKGGWFYTLTTETFVELLRLSFSYYTVLRNINDR

>SgreOR27

TLVAGGLLCGALISCIVDAFFVSLIIYQAAHLQLLNLMLVNVCGAQGGAEPGLADSRGQKRREAAQSADVEGQMRRRLAE

CVCYHCDIDSCVQHLSSLLGPIILGQFLMDMVTISATAFVAIANNADSNWLVKYMSYLSSVIQQLLLYCWFGTDIITQSE

RLQMSAYSSDWVGATPRFGRELRIFLCRAHRPLRLTASKFYTISRDTFLLLMNASLSYFAVLREISNK

>SgreOR97

QVSTMVASVGWLGAPLVSRALLAAAGESGEVPRKLPLPVWLPVDVQASPTYEILYALEAYTTTLTGLVTLCIDVLFIRLM

LMVTAELEVLNYNVSTMANTHEKISDRLHNESHKQNTETQDFYKSDRALQKTETVYKHGSDFQLYQELVTNVRHHMIILR

TIDLLEAAMSKSIIILLFINMGALCSNLLVVGVLLQAGGGVTRPLTLTAMIPFLLYQTGMFCVFGQLVTDQSEKLMISAF

SCGWNESDARFRRSLLIFMAMVNRPLEITVGKTCKLSREMF

>SgreOR16

HWDGRNTELLENCTGGILLAQCLSIGAAVSFQLFQVAVSTQSLVQAGKFSCYLTVVLVELFMYCWFGDDLITESENVTLA

AYSAVTSLQGFPASVRKSLLIVMTRAQLPLRITAGGLFPFCRESFVSIVNMSYSYFAILRNFKND

>SgreOR1

ASTDVSNHIQKPYEINMSNRNEGEYFGAIINLMHFFKIWIPDSSKRNISFSVFILVPAYLFFFALSCIEIYHNWGDMLST

TDAVNTFVIYLATSHKYFRLIYHEKDLKKLMNMVENNFSVPIRQNDAMRNSIIKSYVQEVKKLTILWTTLCFTTLCGFMI

LPLADGLFHYYRTNSTKEIEWKLPYRTWTPFNDYGTVVTVPLYIYHMFMGFVLIAEIPAFDTIYFSLVNHSCAQLKILQN

SLRNIVTISAQNVHSNEKNDVTFDTMLEERYIIEENIGHSSLSPAHKMNSVLNSFTPSGNCDRPLNHIHASELDNKIRQN

VGELVNHHEKILEYIDGVEAIVNAVFLTQFLCSATLFCLTGFQLTVILKEQQLARFLNMMELLGAAIFEMGMFCYFANRV

MDEGINVGKAAYESQWYYVSKDYGISVSIIMARCTRPPKITFGKFADLTMENFASVLQISYSYFTLLTRLNE

>SgreOR51

RFSPEAKRLPFQQLPLTEDSPFSVYALSYALQGVSMLWIALISVQMDCFFTAAMIHATSQLRILSSIIADLQLGNDDQKL

QEKISLDSMYKELRLCIQAHQEITRFVDHLESVMNPIAMMQLAVGVFNGCMLIFPATYSSENDALLKCVIAAPTISAQLL

LYCL

>SgreOR35

GGGGRQRVVHSATTAASSFTLQKFEITKMKDGHIPWSETALWMNARVLALGGSWRPPEVRGFTLYRIWVLFTQFSFLIGQ

LQGLYYFWGDANRIIQDVCLLVTTILGLFKFFVFVIKQEDVFKIVQTIDDRRREQGKLENPRIAAILDASYSSAKTITVC

MAGVGGTAPAVWAIMPLVMRSLGVGPPDRELPAMAWYTSRDTVSPVYELLYILQYFSMQYSYFAAMCLDLFFACLIIHVA

AQLEVLNVRLSQIREDLYRKDRTSEPLGDTKDEDVEEDSAWRELSECVEHHKDAIKLVDDLETLVNPIILSQFMGATIII

CVTLFLITTNKQHFVALVRLQAYLAVVVYEIFMYCWFG

>SgreOR91

MGKPSNDPARLLGSEASVLRLLGLWCPQDHGGHIVPTMLAAVTLASVCFLPAGVVLKLCGDFPEEIEEMAHCCYIFIVCF

GSIVKAVLFIVEGGTLREMVQLMYSMRTQYGADEGSENIRSCYQDNVDRMYRYFQVMALLPTLYWICSPLLFGAVTSAAL

DARDNQRQLPLPFWLPSGVDSSPTYQLLYVIQAFSLTVTVESAICLDVFFIRLMMMVAAELQVLNENISAIECDQANRRE

DQECDSLIPRGDCAPQFTEKYPENFSDDEIFSRLLKNILHHQTILRCIWLLQTAMNVSIFILLFVNMANLCFNMFVTAGL

LQDGRNVTKAVTALSTVPGLLLQTAMYCLFGQITTDQ

>SgreOR24

HFADTLVHDINRGLQEFGEAYRQEKEAVYAACARQSVLLTCVHVGMGAVVNACWLTMPATKVAACQTIEcRIKEGLPAPV

WFPFPFTEPPVYEAVYVGVSLALFYGYILTTMLDGFFFTLIIYTAGQLRVLNLMATRLCDPEPGSKVCLDEQLQWRIAEV

VQCHTHIDRKTDSEWLFKYGSYLSAISEQLLLYCWLGHDILTESERLQMSAYSSDWTAAPRRLRAQLRVFLCRAHRPLRL

TASKFYTISRDTFLLLMNASFSYYAVLRQLNGD

>SgreOR22

MKSEQEDNRNVAMDFLVTLMHAWALVSRSTSGPWLLVHHFYKAVVFLCMGSLLVSELIAIGIGGDDMLTLSLNTCVMLSN

AVAIYKLVLSTLWPGRYRSLVEHVVRDLTDHLSHDPSGRRRLGADSRTAYTFTMGFVVCGHVTVASWSLLPLLLKPVEKL

RLPLVAWTPFDSSCGIGFLVTYVYQFTCTLFMAWTSGATDLICVNVVMQLCSHLEILCCHLQRVGGQCCSDVGSSCDHGY

LGKSAEKPAPDKDDLRDQLRRCIRYHQEIISVAREMDSLLWSIVLSQCLCGMTVLCLLLFQMALYTLTIETIAKYLSYMA

SILLQIFCYCWFGDNLSSKSTDVARVAYSCAWTRGSAGFGRSLCILMARAQKPLIVSGGSFYVLSREAFIRILNASYSYF

AVLY

>SgreOR34

GCVLSTCWSLYACWALCTQLSFLVAQARALFHFWGDVGKVTHDVCLMVTVVLGLIKSYVFCRRKGDFFRIVNKIDSARSE

QSKSADPEVTSILSASYKSARNVTLYMTLLGGSSPAVWAVTPTVMRRLHVGPPERELPATAWYSSRDTESPLYELLCVLQ

LFSMQYSFFAAVCLDLFFVSIIIHIAAQLQVLGVRLRRIGELYDKRSNSSRNSPTIRRHAEVGSSEKESQWMDLCTCVRD

HHAIIELVKELEGLLNIIILFQFLGATVVICVTLFQSSTNTGNVMTLLKLQAYLMVIIYEIFIYCWYADDILYQSSQLAV

GAYSCCWLDSTPRLRRALVLVLCRAQRPLGLTAGKFYHISRATFVRLISASYSYYALLNQMNDK

>SgreOR85

MTARPPAGTSMEHPPGDSDWLVRPGATLRRLMGLWRPRGRAASLLNRLLAGMTLSCIAFLVLCVALKLIADPPQELEQIA

LCSLVATIGVGFFVKAALFMAQGGTLRHTVGLLADARLHFSRGDNELTRRRYLKLSNNVHFYGQMVAVPAMIGWVVCPLL

SRSVAKADQGLQEAQRQLPAPVWLPVDAYASPTYEILYVAQSFCVLVTAESCVSINLFFVHMMLMVAAELEVLNDNLSAL

ENINLKTRRTEREGLISRYKINGRGLALLNGGQCLDEHALTENAAHEWLHRQLVKNVIHHQAIIRSVALLQSAMNVSIFI

LLFINMANLCSSLFVAAVLLQRDGNIGKAINALFSIPGLLYETTIYCIFAHIMSDQSERLMY

>SgreOR17

MEPAADLAEELQLLQWLMHWAGTMRHPRAGPWAGRAYYLLNAIALAAIVLFLCGQGTAILREGTRDLDRFMLMVSTFNSV

TIWFLRMCHIAIHEHQFHFLALQMDRDFREFLNLRDIPPLRKRCRGHRRFLLFYLTLGVIVSVVWSVLPMATFGVGPEAI

PNAMALPYDVSHLHTFIPTWIFSAFIVVHVTIMTITTDTFNVSLIAQLRFQLFVLNRNLITLNADAETIKSPLTKGNYIT

NKSSNESLEHRNIHHRLKQNVLHHQLIIRNTELMEKCIGGILLAQCLSIGAAVSLQLFQVAVNSRSLTQAGKCSWYLTLM

LAEVFVYCWFGDELITESENVTMSAYTAATSLQGFPADVRKSLLLVMTRAQRPLRITAGGLFPFCRESFVSIVNMSYSYF

AILRNFKDD

>SgreOR50

MGWDSEGSQPLTWQYTGTSVLKCDLRMLHLIGVWPLSGSRLYRCLVTVVIALCFGHFVEAVIHLCTLDGYLEDFTLALSN

ISVVIVGVLKVTFFLRHERAYCRLVRWLDDLVTGQREYTRGRPQLEEVSAGAQRLARRITKAFTVYDAIVVLAWTLVPLT

APPEAKRLPFEQLPLTEESPHSLYALSYAIQGASTFWIAVISVQMDCFFTAVMIHAASQLRILSLRISDLQLGNGHLQLE

DNGSLDGMYEELRFCIHTHQDIARFVKHLESVMNPIAMMQLAVGVFNGCMLIFPATYSSENEALLKCVAAAPTISAQLLL

YCLGAHSV

>SgreOR64

SGGGIFFAVVFVSNLGNMTEAALGLYLGHGGLQEITLVLPNTLTVASGVFKMVFFYRDRGRYYGLVRRTDRLTMLQLAAP

GEDAATIVRDAGRHSLKLSSSVYAFVSLQIIVWFPMPLIAYPGQRKLPFVQLPWNNNTEIPVYELSYALQCFSSFTIIFI

TLGMDCLFAVIMIHVAAQFEILITRIRNLRLDLSTSVMQQKPILSQHSRNSRTSMNTPTESKEILEFQHQITEAHDKMYS

DLCNCVEVHQEIIRFVRHLETMMSPIAMTQFVFSVLVACVALYQATYSEDFSAVFRCAGFLPVPGGQVYLYCWAAHLIME

QSEAVSAAAYACSWIEASPRFKRTLRILMCRAQKPLVLTAGRLYQVHRSTFLSLVNASYSYYALLGQISKR

>SgreOR73

IWGDLDEMTLVFANTFTIASGLVKLAFCTRDRGLYSALARRLDALLSVQRAVCSEDPELASILRGSRKQAASLTVGMLLM

MFSQGFVWFPIPVVAHPGERRLPFAQHNWDNNTNYYGLSYAVQCVAGVYLSQISFGMDCLFAAIMIIVAAELKILSCRIV

KLEADDISKQVGESDDRLGDTTLRARQKPYSNLCQCIETHQNILRFVVRLQNTMSPIAMT

>SgreOR90

VLLASVTLASIAVVPAGAALRLCADFPEELEELVLCSYMFIICSGSVIKVALLIGEGGTVRELVRLLSDTRRQYDSGGNS

DCIRNRYTRIVDRLHRYFQVMTVPALMCWVASPLLSNGTLTADQQDQRQLPLPLWLPADTHTSPTYELLFIIQATCLTIT

AEATLCLDVFFVRLMMFVAAEIEVLDQSISTMHNFYLKDTGPRDYVYGVGSERSVMAPGVGHSTPPVDESSDTASDEMFS

KLVNNVLHHQAILRSASLLQTAMNVSTFILLFFNMANLCSIMFVTSGLLQRDGNLTKAMKALLTIPPVLFQTGMYCIFGQ

ITTDQSEKLPDSAFGCGWINCDAHFKRSLIILMMMVRRPVEITVGKTCQLSKQMLLQVLNGTYVLLNMLFQVRSTE

>SgreOR48

VLSRPHHLSCRWPYGTVELKPSRVPVFGQSLPMERDSQAVEPLTGQYAAGSVLRYNLRWLHAFGVWPLSGSPLFRIFTAT

ISALSLAHIAEGVVHLCTLRGDLEDYTLALSCLSIMVVGSTKATFFLCNEAGYCRLVRWLDALVNSQREYVRSRSTREAI

FEEVQRRGIRISKAFNAYNISLLSLWFVAPLMSPEKRLPFQQLPVTNTTILPTYAYELSYALQVISMFFVGLIHVQMDSF

FIVVMFHISAQLKILSERIADLQLRRGDKNELFESNENKRLHKSMSAIETHKQLCLCIRTHQDITRFIGYLESVMNPIAM

MQ

>SgreOR89

QDYSGRLRAALTLTAVALMPAFVALKLTMDPPDELEELAMCGFVALVAGGMLVKAALFVHGGRALRQLVQLLYWTRGRYG

SGGSGDATRRRYRKLSDRVYFYLQAASVPAISGWVCSPLLSRIVLQGAPESGDARRQLPTPAWLPLDIQASPTYELLYAV

LAFCLLIASETSVGIDSFFIHLMLMISAEIEVLSQNVSAMQRTLTETTQHKDEDWCSNVEINNETQKFSRSQHSLNTTLI

TEDVSDEEIYSRLVNNVRHHQTIFRLVALLQSSMTVSIFVLLLVNMANLCSSVFVTAVLLQRDKSVTKALKSLVAIPPVL

YQTGLYCIFGNIITDQSEK

>SgreOR41

DICIQSPGGESAGMITIGSSAKPIVEQPSKSWDDVLGRNVKILIFGGVWRPVSRSGWRPYVFPLYFASVCGSLLTIIGLD

IVRIWLLWGDMTEITYAVVTAVCCFNGVYKMVHCFKHGGTYSRLVRGLNDLVALQRPYCERDDTLMEVFQEVCRKTKRLT

IGCLTYMSVLGQMWCVLPLVSPVPPDSREDPMPLVSLPGLHKGNTGWYSFIYLLECHTVFYWNFSSFGMDMFFASIMSHV

TGQLNILNMRLTQLRQEESSEDQVRPSTCNGFNLNTSNQDQEQNVNDSASMYIELCECVKHHQAIIRYLEFLDSVMSPVS

LTQFLCSVVAVCVTLYQITFNPQDSAVVKCALYLPIPALQIFIYCWCGHDTMEALINASYTYYTLLRQMRNL

>SgreOR39

MSVEPDQRLTSLPGPHSVLDVNIGVLKVAGLWPTRPYGLFAIYTAWIYLTQWAVFALDSMSLYYYWGNLNMITAVFCNLT

SITAGIIKMTHFFVYKPKYYMLVNKLDALVASQQRITDPNVNSKSILVQTSKLNKYSTYIIVTYGNLVGVPWIVLPFVMD

TGETERTLPVVEWYGIRQEKSPLFEIGYVLQCLTIMYWFFASWGLDLFFGALMIHLAGQLKILNNRVANVGRKENAALNG

SSAEESERKIMDFNRDVVSRKTPVQQQEVTLYSELRNCIIDHQEMISFMNDLEQTVNFVVLVQFSAGTLVICVNLFQAAL

NVQDFSSVLKVCMYMFELILQLFIYCWCAHDVMVESERLSTSAYFSEWTGASRRFNTALHILMARAQMPLTVSAGRIYTI

NRSTFVSLINASYSYYAILRQMSDR

>SgreOR74

MKQDMQQIRPENMERPVAWTESGESVLRFNIRHLWFFGLWPLYESRMFLIYTAYGFSLGVWNVLEGALAAYFTWGDMEQT

TLVLMATSTNCNGLVKMGFFLWDRRLYYSLVRRVDALMSLQSEFCCRESVLWNILRGSERHAFRLTLGMLLFMFSQCFIW

FPMPLVSHSEEQRLPFAQHVWDNNTGNYELSYIAQCAAGLWMTQISFGMDCLFASVMILLAAQLEIVAERIMGIRVEAST

PMKEKAEGYKKSSTAKHADTMYKDIRLCIESHQKILSFVAHLQDTMSPIAMTQFTSAVLVICFGLFQATYSEDFSAVLKC

ASFLPIPCGQVYLYCWAANNVTMQAEAVMMAAYSCSWVEASGRFKRVLRILISRAQKPLVLTAGHLYPIDREAFLSLVNA

SYSYYALLSQMNNR

>ASgreOR20

SCSWMIRLRHIASWEVEFQRLATEVRFDFDEFLSADDLWLLRLRSRSQRRFVSAYLCSGVFTSAVFAAIPVSAEGFPFIM

ALPYDANQPLAFATTWLYTAYIVFFVVLGNRAADSFNISLIVQLRNQLDLLSRKLRNLNGSISHTKSSIKKTKRSEDVSE

DVYYQLRKSILHHQSIIRNVQLLEQCLGRMLLGQCLSNGTSFCLLLFQAAKRAKGVQELGKTCSYLLNTLFDLFVYCWFG

DDLIFESEKVASSAYDAVPSLQECPTGIKRSLLLLMLRAQRPLRISAGGFFALCRESFVSVLNVSYSFFTILRNFKED

>SgreOR7

QRQVKDVRTEAEATAMLRECARHHLQVCRFVQSVEEVAAPALTLQLLVSTFLLCMSAFTATQIPVGSPLLARVVIYMLTG

ASELLIYCKYCDDVISESGRVQQALYGSGWAARGGAFSSGVLIMMARAQRPLCLRAAHVLPVSLQTFTKVLNASYTVFTL

MRQIKD

>SgreOR94

TSAAAAMALALSDLRTLQGSGASILRLLGVWWSQGRLGRLRSAASAIVTLSAYVWLTVFAALKLIIDPPEQLEDISLCCF

VIVICFGYFIKVAFFIYKRDALRELLQILSDVRGSYGNGKSSHRIRSRNQKLSRRLYIFMQVALIQGLVAWLSTPLLARV

FLASDQESPEPARQFPVPLWFPEYMYQSPSYELLYVVQAFCLLVGTQSATCVDAFFIHIMLVVAAELEVLNGNITAMQKD

RLTVQGTEYQQRSHRTVPKRDGWTVESNDYLQSTEHVSFEGIANDMYVQLVKNIHHHHEILRSVSFLQRVMNVSIFILLF

VNMADLCSCIFVSAVVLQRDGHITKALKPLMTIPPILYETGLYCIAGQMLTDQSEKLVNSAASCDWADCDARFKRSLLVF

ITVAARPLEITVGKTCKLS

>SgreOR76

QRAANFVMSKVSEAARLRTEFGVQLRLLRAAGAWTPGCGGVVQAVYGCYCAFIVVSLVVFVVTQVSAILHFWGDIISITN

SVCVTFVYILATFKLVVLLKMRGLTDELLDNLDRCMERYGRTFGREKAFVFGECARRGRWISLMQAWMGLCLYVAWVMQP

MLRVQSCSTAEcRSQSGFPTVVWYPFSFTEPPAYQVVFIVVSTGLFYGNIISITVHSTFCTLMIYVAGHLRVLNHMARNL

CSTQQYDEATGDHKQRNRCDAGEERVREHLRECVRYHIDIERMVQQLSKLVGPILLGELLADMITVSASAFITTALKSNS

GLLLKYGWYVFVVAEQMFLVCWCGDEVLAESERLQVSVYSCDWTGGSPRLLRELRIVLSRTGRPLLLTASKFY

>SgreOR99

ATRGQPKKPSADESKKQPGMEDAGGLIGPSLAVLRLMGLWRPAAASGGACAGAAASLPGALSCVAIGLLSLSCASRLFID

TPTELTELTVCAYLFVIITANFVKACCLLLQRNTLHELVTLLAEAKKKNVIDVQHNEEIRSSYGRTSVRLYRYLQVMIVV

SSIAWLFVTVPFRVFTAGSTNIDWPTPIPIWLPIDMQLSPSYELIYLGQVLCAVATAGAMLGVDTLFFHLTLMIVAELQV

LNDNISVVGRVASPYEKEVFFRVKDAGEHQQNFSDVINNGQNEGGASLSNPQATITEKNYSDLVEIIQHHQIIIKMVGLL

QTIMDYSVSVLLLTNVLDVCFLIFTMSE

>SgreOR12

NHLLQPLHWTAVLRHPRGATRSPLCFRLCTIVMTALILSFFISEVTVLYREGTDDLTAFTMTLGVADTNIIWLFRMVHIT

LCERAFHKLALQVGNDFAEFLTLDDIPMLRSQSRAVRRFTLIYIWFGIGAVVYYLFSPVSDEGLPVILAVPFDAKQPLGF

AVTWIYVSIVTMHVVVMTMVFDSFNISIIAQLRIQLTLLNSKLVNLAKEMSERPVHSSKTPAYRELHYRLEKCVRHHQAI

IKNADLLEKSLGAMLLAQSLAIGACACFQMFQVATNKNGLQETGKFGCHLTVMLAELFEYCWFGDDLITESENVALAAYD

AVTSLLECPVSIKRSLLLVMQRAQRPLCITAGGFFPLSRE

>ZnevOR1

MHSTAKTINNDQIVNGYHQELVKPQLLMLTLSGLLPPKSWSSSRWKSIMYDVYTFLSFIF

HIPFFLFQIMGVYVFWGNLQIVTGIIFQMTVCFDGLVILVYFTYHRKYLVKIIEMLGTKF

VPYLRKVGSSKKQDDIMREAIKFSNTMTKILMIIFVVVMSAWCVFPFFVKYWSHEQEAEI

ANYTNVRVHFEYFVIATWLPDKAFTSPMYEIIYACQFFYIWSIVANFTVGNMVFSSIFYG

ISIQFQLLATSIRDIDDICTDLNAEFKNKEDMRSDRTHLASDISDKEAYDRYPLRGTFQS

STDTFQVKFLNDEAFAGAYEICCKDITDSAHWNDVTTDTNTCSETAYMIECINYHQCLLK

FCDEVDDFLSPLLLIYIVMYEAMICVPLYQLVMGEADNMFRFFTSAVDATFLPLTICLSG

EMLSTQSMAVRKAAYEcRWYHRSQSLKKLLQMILIRTKEPVKMTAGKFFVISLETFANIA

NKVFAYFTVLKNMQP

>ZnevOR2

MISALSASENEHRDILSKLDSFNPLLILLNVSGMLPPLRIYSSRWKTNLYNVFSGVTFLS

YIPLIVMQIMGIFEYSGNLLLVTVIIFQLAAYFDGMVTMAYFIYHRKRLLNTFYSVENKF

NPFMQKVGNAQTQKKIILGNKKFERIISFVAMAAFLADMCTWCVLPSIVRYLAYFNEEEG

TADVEVKQHLEYFILVMWLPQKASEFPCYEILYIFQFFSVWGVVSNYAAGKLIIVSLYYH

LSSHFRILASAIEDIDVLCNNNTTKDECNTFSEEMTLNTSANDTNVSLVKFTVEDLRRHF

SVTQPENISDKDYTFPSECTKIFSREDVLSTAPEHRSEVTYLTHCIKFHQVLLKFCVEVN

DFLSNILLFDLIIFQILMCLPPLQLILGQNVSMVKFVSSIVDTGIWPLLICFWGEDLSQQ

SLSVQKAAYGCSWYNRSQRLKKLLQFIIMRTKKPVRMTAGKFYYISLETFADIENKVYAY

FTILKNST

>ZnevOR3

MMESTEAEQRKQQELIENYSLRFHFRALKLCGVLPPECVYSSLWKSRLYDMYTCLVLIWF

IPAIAAMFIELCNYLNSLEKAIPIIFQISAFVLSAILAIYFVYKRKIMIELFHILETNFR

PYMEILGSSTKKLKTMEESLYTGKLISNTFLGVSCVTVFIWVVIPTIKGYIEFILQINQE

GIEHNHGKYFGLAMWFPSNVNKSPFYEIAQILHGVTVITGVLNITGWFIAMLGLMYHTAS

HFSMLVDLIEDINIIFSTKKESEQHCLHTPQRKQYRDERHAEDESDEESQHLAQLHKYLT

SCIKYHQEIIGLCKQIEDFLSPMLLIIFVFSEALMCLSTFRLAYGFADDESLKFLMSGSC

FYVWLLSFCWAGEYLTEQSLAVEKAIYGCPWYKHLQTMHDIKFVIMRAQKPIQLTAGKFY

NVSLETYLQIANTVYAYYTLLKNVADS

>ZnevOR4

MDSDDLERETSECEQAGNSTKLDLLRLQLKIIRFFGVFPPEEGTWKHSLFVTISTVYISL

YFPQMYAMVAAAYEYWGNIDMVTKILFQITTAVEAGILTSYFIVRRKKLALLFDMMETNF

IPHIEKVVTPENKRRIIKEASKRSVTITYILMFIFSSVILGWICLPLIRKYNDAPAQADI

QVMSENAWNYYCYILWLPKEMVSGPFMYEIIYLCQVLTIYVVVGHYTGCNVILFFFMFHI

STHLKLLASSFEDMRKVYEMESGENEEKTHTQTGFRIFMSPTIYNYFMYDSGPIVPNFGE

NAAHVEGDKPRFLNAKKNPENIRLLQPRSNGDMMTQSETSERQNVDISTQTASIEEKMNL

HFINCIKYHQEILAYCKLVEDFLSPVFLVYFVISESMMCMAVFQVVMGDDEGLIKSVVSV

GNVCTWPLMICLCGDYITTQSLELKKAAYGCGWYNRSKQFKQNLRMFILRAQEPVRFTAG

KFYTVSLETFADIINTVYAYFTILKRMQES

>ZnevOR5

MSEKRENQILNSHGEEHPRRLKMLHLQLIAFNLSGILPPANTSGSRCKTMMYHVFTALSL

VWFLPAITLQLIALCQRLDDIEVATAILFQVSCYLSTGIIFFYFVWNRTELIKLFDTLEA

GFIGHMDKIGSPTRRNAILEEATRKSAVITRALFGLCFTVEIAWGCIPCILGYLEYLTDD

EPRDLTNDRGRYFGLTMWLPENVNESPTYELMHVFHVIAVYTVVSNITGCYMLMFAFAFH

TTTQFKILCAAFEDVDLFVQRLHNSGRGRRRNTMEHSTDVDLSTEDGNYNYVSGSENAEM

PHEGNFQGHISGSLGPQYSVVPGRRDKAFDLRYPNDFNITKSPRWNTEENPETSDIEKCE

YSLLQYLIECIQFHQALIKFSKDMNKFLSPVILIFFVFSEAMMCLSTFQLALGQADERSF

KFFTSVILAFIWPLVVCTYGDELMQQSAAVSRAAFGCQWYDRSPSFNRLLRTVIMRAQKP

VILTAGRFYVVSLATFADIGNKVYAYFTLLKRMNEK

>ZnevOR6

MSGTIGTGINSKLEDSLFEQEGESKRLNLLGMQLKPLHLCGILPPANIARNTCKSFLYNI

FTTLTLFWFIPHIITQIMALYEHRNKFEMVTGLVFQIALFVHTGTVAAYFVFHRKQLIRL

LESLETHFVPYIEKVGSACRHVPIIREASKQASMLIWTLLVVCWIVLFAWGFLPIIVRYF

DVLTSTDKEIDESLDSKDEYLKYFGLIMWLPPNIDKFPVYELVYVFDSVAAYVVDSNLTG

SNTIFFIFMFNISTHFKILTSCIEDIDEMFPQTKEATDKGLGIPKAEIVTSKRNIISLDD

KIFGPPIPEEIVSGRHNVRREYGKATKSGFQGGDYTTETSEAASSQWAENIFMSNESGEQ

TTSDSLRDDKIYQYLIECVKYHQALLNFCAEVNAFLSPVLLVFFLGSEAMVCFSAFQLAL

GLSDGNFKFFTSVVDTIAWPLMICWYGDYLSEQSLAVKYAAYNSSWYNRSTRVKKLLHMI

ILRSQYPVRLTAGKFYIVSLQTFADVGNKVYAFFTVLKHMLES

>ZnevOR7

MVFGVFRASRNNPHEESSGGILDIQLKCFLYGAILPNENIRSSPWKLRMFNLYSVCSLLM

YVPSVSAQCYALYLESENITELTDTFFTIIAALLHCSISSYLLTQRKALEHLVLKTNEAF

AEFTSKLPLETKHTLIMSDAKKKIRQYTLIFIISIVITGFLWLAIPNIFWHIHNAKEKDG

EDKIHWEHLSYRMWLPPGALEHPTYHFIYTYQVVLIINLLTDNIGYNSMYYALTIYTAAH

FKVLATLLEDIDQYITSSDNTKSSEQNGIRSFLDGGTGFATDMLSKLSGQDETDSNRKKN

SQLEEYHSLDPRHITSIPIAEDYLVNCVKYHQALLQYVEDVDAIFGTLLFIFFSLNGVMM

CLAVVLSTLGKGSTGSIKFMSGVITVWIPTFLICWFGEHLTDE

>ZnevOR8

MGSGIFRSSINNWQEENSSKHLFLFLIFLQLAGILPPETFSSRPWKITIYNIYSALSLLW

FVPAMVAQIFALIQHWGNVSDITDITFEVAAAISNSSIGLYLMLNRKQLQLIISKMQSTF

YSYTKHLQFNRKHSSILSEALRRNAIFSWIVIISNCSSILIWVILPFILWHTEMKNEEEN

TSNIPDQGTHWKYFCFKMWLPTKATHSPLYQILWAYQAFPIYSLVQIFTGYNLMFFSVTT

FTAAHFKILATILKDCTKTKLLSHSIKDTEQGLRNSKSSNGSVLKDTGYVLQAKEKFNKV

VVLKYCERNEVAIHKNKEPEWITGTDLNESEAGIAQQRYETADYLVRCIRYHQALLEFCD

DVNTLYSPLLFISFYMDLFMMCTTVFQVTLGSEDQSYFKFIIAAISVWVPILLTCCYGEH

LTEQSLAVQEAAYECSWYNSSPRFKNHLLFLIMRAQKPVRLTAGNFFAVSLEGFSSIAHT

VYAYLTVLRQMHDS

>ZnevOR9

MAGTSTALGRDAECETTPQRTYMRADDKNWDLMGLNLTLLSLAGVLPTPNIASSSWKLRF

YHAYPSLMLVLYLMVFTAQCLALYKFWGDLDAITDNAFTMVGVFMCYIQAAYAVTNSRKI

LRLVDTLQMKLTPQMLTLASGEDQSIMVAKTARKTRALTWTMFVIVHGMLISWIAMPIIQ

KYSHEPDEEHRNADKPSPYFCFIIWLPFDATRSPTYEIVYTIQTICFLMACLYYTSMNTV

FMTFIIHTATQFRGLAMSLRDMDKLFPAQDMGLEDRRLPSGVELNAHFIECIKHHQAIIR

FAKELNEVLSPLLLFYFFCSQLIMCVVTFQVVLSWGQGKSVLKFVLGLAAAACGPLVFCW

FGTDIMQESLAVQQAAYGCNWYHHPPSFKFLLSVVIMRAQKPVHLSAGMFYEVSLTTFTQ

MLNTVYTYFAVLKQLYDE

>ZnevOR10

MTKHNAMSLNLKLLHVAGIVAPPNIKVSFFKTALYQLYIACCYLVYIPVLSGQILALYHF

WGDIEITTNNIFTLLGAFTCFVEATYARFNTRNIAQLFETFQNKLVPKMSTVGLTEKKEE

IFHSASKKARLNTLIVMIILDLMVAAWAPAPFIKQLMEESKNVTNTETEADRKWLNFCYI

IWFPIDITVSPYFEIMYLIECIVFIIGTTYLKAVEMTIAAMMVHISAQFAILCTALEDVD

KITSVSEEKMYSDTRKLFGRETTSLEGTDDPEVIVLGSQEWKVIQTRRATDCNTVDMDLQ

HNERKNEDFPELTCYLANFVQYHQAVIEFVADVDKAVGPIFLVCFFIRQFLACVMSFQVA

LTWGQDNNILKFIFGFLGSMCSPAIFCWYGTAIIQSSLELQRAAYECKWYQHSQILKHYM

LMIIVRSQKPVRLSACQFYVISLQTFGKMINTIYAYFTLLKNQYEN

>ZnevOR11

MEALSLNLRLLTLSGIVEPTSVSGSLWKKSLFSMYMTTSIIVFIPILLGELTAFYHFWGD

LVVITNNMFTLVGNVTFYGEALYIIARKKDFLKLVEKLQDMIDHMPRAGAKCDLIAKQTA

KRSKSLTLSMLFIVYMVPFSWSISPLVSMLLAVDEDIPEHDTQLEDEVEEFWKSLISIMW

LPLDATQSPAREFIYMGQAFVFLITASYYTSVNTVFVALIVQTTGQFEILLATINHMDDA

VRSQDLLPFEKTEDLTNTVLMDVSEGKVMRWMHQHDDPTDLRPYFVAVIRHHQMIIAFAK

QLNDLLSPLLFLYFFSAQVMMCVMAFQMVLTWEEQNNFLKFCFGLLCVLVGPFFFCSQGD

VLIQKSFEVQQAVYGCKWYYRSERFKRTMLQVITRSQRPVSLTAGKLYIVSMESFAKMLN

TVYYYFAVLKQLHEK

>ZnevOR14

MENQTSPSKKIHERLDLMALNMKLLRNVGIIPSETTSPSSWKWKLNRFGHSLFFLVYISV

LMLQLLSFYFYWGNMEIIVENIGTTSGLAACVFTALYVMVNWEEFCTMVHELETNSIFSA

EMVRANNKQMEIIRTSKRSAIIITWITIASVTALCVSFDMTPMIRLFFSKSGHENSENIE

RINVNFEYSVLIMWLPGDLVEDYWYWHLYTIQAAICFVSFAYISAVMPFILTITVYTETQ

FKIVSSSLIEIDERYKVGDTDTEFPKAEISSEFIGSTREVNNDNTRPLRLIGMREQNTIK

ESDGPNTFPESGGFYGLAATELALDQDDATNHLVECIKLHQAVIKFSNYSNSVTSPIFFI

YFIAATLVIVTHTFKLSLSVSLKYCCTLLFCLTQIFVYCHVGERVKTESLAVGDAAYSCH

WYKRTNRFKRHILLILVRAQKPLYFSAGALWKLTMEFFSQVMNTSYTFYTLLSRVNEI

>ZnevOR15

MTITKGCFESKHRKINLLNFNAKLFNICGIIPNENVKASFWKSILFRGFQIVSFLVYIFI

VILQILGLYFYWGNITLFTDCLGLIAAFAISYTSTTYFIFYWKDVSKLIEAFETNSIYST

EFIRLNQRHMKIVSDTKNFTLLLSKLACLSTLLAGIIYISPTFIQHLMASDKQILEEIEG

AEGFTKYFVFVMWIPPILKQPYMIRLTYVLQVMCASTAFIFGAAFVPLEYVLILYTGKQF

KLVSSAIQDMDKLCSLQNLDKIVDNVSEQMDSPNYSRRFLPEHIRVRKRELGTEEGNPNA

TEGQWNKQFNSKTFDNTLQAHDINRSSGKSDDISSAEPTPWEENDFASSYLLQCIKLHQA

CIQLAAEVDDILSPVNGLGSLCATSFIITHVFQLSLVITAKYVSFTLTFVIIIFVYNWCG

QTVILESTAVGETAYDCLWYERSKRFKRLFLMVLLRAQTPVIMNAGILWTVGYEHFGQLL

NAMYTFSTVLRQVHNT

>ZnevOR16

MSFKMGNLDNQTTTKGVEDKLSPNELKVMSLNRMVLHLSFTVPTKTISASTFKSGLYYLF

TPCVFVLYALVLTAQILGVYQYWGDLDVTMENLFLTTGFVMCYWEGAYLKIKRDQIWKLF

DRVDSSPVPDISNSALSETHKKIVKEAQSSCMKLSWFIFLSVNVGTLMWAVFPLVNLLVH

SLSEGENTDEDTIPQQSEYKPWPYLMYIIWVPYDTTTMPMYAVTYIVQVIVYFAASLYNT

SSNIVFLNLVLHATAKFKVVESSFLSMGEMMSANKDEDDKLEQITATLECHSGKETLVDE

DFVKYNQLQSRTSVTDPNNYPVGNDLQLCLDGVFNSECSGIEDFLVECIKQHQDAIDFTR

HLNHIVSPWIAFALLIHQLLLVAEVFQLATTAGTGFAILKHFMSIIGVLSQSLLLCWLGE

RLIQQSLRVKTAAYDCNWYGQSTMFKRLLLMVIKRAQDPAKFSAGSFFDINIEKFDHILR

RAYSYYAVLRKMKEP

>ZnevOR17

MHPTGDQHKQNPETGDIIVHVEEYPEIMRLNFILLNIFGLLPPTRAGPLMKFIYKMFIAA

VMLMDVLILLGQVLAVVVYWGNLELISAIVGVMNGFAVAFISCAYFVINKEKILMLVDIM

RDKFVATTKSKYMNLIKKADSQVVVYLYLSSPVVVCCSFSWGVATVINMNEFSNMETNNV

TKTEQNVDKMVFVMWTPFDVQQSPQFEIFSVFQICFVCIGVVTSYTLSVLVLSLMSHSAA

QFNILVAMLNDMHENISDNELTAIETAFSANSAIDGGKVFDNETDEESRNGHCSELLRHF

NEPDNLDEDKFQRYLINCIKHHESMINFVGRISDVVSIVVFLQIMNLPIILCVTGFQMTQ

TFDRHEQFFKFCSLSVAALYHIFSYSWFGQQMTNKSEEVATALYSTDWYNQKPGFKRLLL

LAIMRASRPVKVKVGVFYDMSFDTFTSIMNVAYKYFAVLIQLNES

>ZnevOR18

MTELTKEEILDSRKTDVFNKEDETSKKNTKIMTFNFLLNAFFLLPPDGATPFVNLVYKVI

RTSILVIDVITLSCQLIAIVAHWGNIPLIASTMSVMNGLTVSFITCIYFILSKTKFVGLV

DLLRTEFVSKVKSKYIRFIQNAERQVKIDVLLSTPIVLTCCFMWTVLPLLNSNLISNVED

KNITTADHYLERMIFVMWAPFETIESPQYEIVFLLQFAIVSLAIMQLYAVDIMFLCLMSH

AAAQFKVLQVMLDDMDQNISENDLQTAKIVDYEHVTTDDSFRNTAHGSISLRSRIQIDDN

SENPGAQMTYPKDDQMFEDQCRDYLVKCIKYHQAIIEFVDYLNEVVSFPTFLKIVNLPIL

ICMTGFQMTQTVGDQKHFFKFASLLAGSLYLIFSYCWFGQQVINESEEVATALYGTDWYN

QKPGFKRLLPLSIMKASRPVKVKGGVFYDMSFVTLASIVNVSYSYFTMLIQLNDS

>ZnevOR19

MKTIRELSGTDQKQIAIPATGKNADETEENSVYELQIFKLNFALLTYSGLWIPESSSKLS

KILYNVFPLLQLTHMAITIGSQVIAMVIYWGNMRVITYTLGTTMGCVITCCDYINFMYRK

RTVLEMVHHMKTEFIAKMRPKYRKLLDSTEREAKFLTILRVVLAVLAITSAGLLPIINKD

ASAVDIDFKNMTTEQIITEKLVLVMYIPFEFRESPQYEMTFLYQILSASMMVFSSQSVDL

LLMILMSLLAARFKILGKLLDEMNENISEDKINYEDYILPDDFSAHSSNFEGIPDETALT

GDKDDPFRLYLVDCIRQHESAIQFTYKLNNVVSIAGMLQMINIPIMMCATGYQVTQGLNI

NEFCKFLTLLVLIMYKLFVYCWFGQDVLDQSEAVQTSLYGTDWYKLKNSYKSLIPTMLMR

ATRAVKLRNGVFNELHFASYSAVLNTAYSYIALLRQFNE

>ZnevOR21

MKGTEKQLTPTKCLDLNLMLLKLGPVFPLDVSSSTWINILYKGYISITVFFYLVLTASAP

LYIFCRDYPLEVAIELISIFITQIRSGMNIVTFIIYKKEIQNLITSLYRNFYIHGTNLSA

EESSVVREAMDHARSMTIGYVTLYSITGLSMILHPLTFKSTDLDQDDAFNHTEGPHRILP

FKTWYPKWDTTKSPQYEIEYFVQATLTALEAWCVSCTDTFCVTLMIYAGCQFDLLGLALK

NMNKNMKSDIGAHKKGTNASYPIQTLSLNNSTLPVIPEENEGKIRNNVSTSGRLKASDES

TLQDKGWEDGYHVPPTVKSCMQVERETATYIKECIKHHQSVLVYVAEMNKCFSTLFFVLF

MTASLLICLLGFQVIVKPPRGLMFVRVLLHWLCTVFELGFFCWYGSEVMHKSERVYQKAY

DCAWQDLFSGAKQFIPVIIMRAQRPAAVRAGLFGNICLPTFGTLMKNAYSYLAILRQMHE

GDGA

>ZnevOR22

MSATGNRQVSRECLDLNLMFLKLGSIFPLDKVSSSTWINILYKGYTTFTVGLFFSVSAAA

PLFIICADYTLQDGIEVMSILLTQIRSGMKLITFIIYKKEIQNLILSLYKNFFIHGRNLS

AEETSVVRETIDYTRKMTLGYIILYCTTALSMILHPLTFTPTDLEQESAFNQTARPQSSR

PIPFKSWYPNWDITKSPQYEIEYLAQATLTALEAWCLACIDTFCVALMIYAGCQFDLLGL

ALKNMNKGLKFDIGAYQKRKKGSYRIKILTLNDNLRLSVITENVGNKPHTNLKEINRWST

SDVTVLQNSVVQFGWEDEDQRPSTVKPCTQDEIEAMMYIKKCIEHHHSLLIYVADMNTAF

STMFFVVFLTASILICLLGFQVIVMPPSGLMFIRVLLHSLCTVFELGFFCWYGSEVMHKS

ERVYQDAYESEWQDLSSGAKQFICMIIMRAQRPAAVRAGLFGDICLPTFTTLLKNAYSYL

ALLQQLHGGGA

>ZnevOR23

MDTKGEMKLDELDPREILSLNLRLLKIGGIWWRDMGLTGNLLYRCYERFVILLYFSFNIT

VYLHAILAEENVAEFSESLLVCVCQVLHGTKLIPVVLRRKKMTSLTVNLENNFYIHGKNL

NHEEKSIIKNAMSLAKTTTILYFGSLCVTTSVMILTPPTLVLTSGLVNSTIKQGHGLLIW

KAWLPVDATVYPYNIFAYIHQVALTFVEGCFIIGGLNAFYLVLIIYTTSQFELLSSSLRN

ATQNIRDQIKKDYTVNKDSSGADTSMLVVTKAAEVIYIQQRFSDRSSEYKASYFKYDENK

VSSYMKECVQYHQNLLEYANDLNEMLSPLLFMELLTASLIICMLGFQLILMPPNIKFFQM

LTHLFTAVFELGLFCWFGSSLITRSEEVHNAAYECGWYDMLPKAKRHVQMIITRGQKPVA

LRTGQFGLLSMPLFAELMKASYTYLAMLKQLQEG

>ZnevOR24

MDPRTSEVYMYTQLKISRVLGIWPYTEQTMLWKKILSKTVFYSMLGIKFCTLLAVSYHIF

LKWGHLPDPYETIVVLTAHFNTIFGMLYIPIRISKFLLLLKTMDDHFIIPTDSTQQHLFE

ETMRSASFVTKMFLGSALSTGVSYAVIPFTAYTNGTMNHPLPFHAALPFDITQTSSYWMA

YFLLEISMFTLCINGSVNCDFFVSMIIKTTCQFRFLQRMIADIKEEAIKRNKQKQQDKSK

KASHTDGTQSEISILSLTDTNKDDKLTTKEDDKTHGEELDCEFLQRLSECVKYHQLLLQF

VHDLKTAASPFMLFQVLIYVIFLCMNVLGMSTVPVLSSEFANTLIFVICVLSLLLLLCWY

GNELTIQSLEVAQAAYECDWCDGSSSAKRSLVMIMSRAQKPVKLTAWKFFDVDLKMFTAV

LRATYSYYQVLHEVYTE

>ZnevOR25

MELKLHTRGKKFVGHEHQIKPNMKKSTSNIKSAEKVKTKEWQSAKPNSLHLNLTILQWLG

LWPCDEDTTHFKALFLRIFCCLVITIQASVNIAECMDMVVNWGHLNNPSENMYIMGCSLI

AIVKEVTIILKTEKIKDLVNILNNKLAIQRKQGSSEYQKIIKSSIKQARMFTLIFISMCS

FVGTSYSFVPILDTLLLQYSGNETSHRPMPYSAWFPFDVSETPAYEAAYFYLALVAFFHG

VYIPCCDTLFVTLIIHLCGQFQILQASLKNIKVDAMKNVQATYRERIRRNSLSTKALHTA

TSEPAECDIKDEKHNARIDISLTDEDLDNEMQYLLKQSIKHHQILLQFVHELEYICSPLM

VVQLLGSLVLICFTGFNLATVPVQSVKFVSALVFMIAVIIQLLVFCWYSEELSIQNCKVY

QAAYDCDWHETPKSFMTSIQLIMKRAQTARTLTAANFCSIDLQTFTTIIRLSYSNYAFLR

RVYDENSTKSTQPE

>ZnevOR26

MSHTDASHTLWLILCFMSVVALWPLRQGRVYSVLRHVPYILAVCAFVFWFGELGTVVSAR

RDTKQLLGYVGLLSGHSVSLYKWCNLVLKRRRLQRLVDSLSACLQMGSAADRSAELHRFT

RATNTRATIMTVCWMGGAMCVIFYWSLAPLLHNGRLVELPGVHGNESAHKRLYFLPFGEW

HFLDTSGSPTYELLYVVWATGSVSNGWMHACCDCFYLHVISFFCGQFEFLTATIRLSEQY

SDLSACVRYHQQLLRAAKRLDALFSPLMFVQLINTLLALCTFAFDLSTMKPGRGDSDLSS

KAGFFAASVFQLYLYCRAGSRLTELSGLVADAGYETRWVDKDRSWRRAVELIIMRGQRSF

RLNGGPFYVISLETLLAHVQLAFSFFTVLKNMNGPEA

>ZnevOR27

MQLPDDLRNKGPLGLILRLERCGGMLLDASVMTRAWRVLIYNISKALTVSCIIIVSMGFAVESYLTRDNL

EEFAESFGLFMTQMKNSIKLISLFVHRKKVLKMIKDVEENFFIHNKDLLAEERSLINGYLSRAKIYAFMF

WIQWGVCIVFQITSKRVSDNAVREMPMKMWVPFDTKHTPYYELGYVYNTLFCVVISWNVALTDTLFFAII

VYTTAQFELLGMSLRTLNQEHEDITRLLPLCFVISALKRLNVFFKSRADPVQVDRQDDDQYLARCVQYHH

RLFDNVELLNSVLSPIECSEILTASVLLAFAGFQITVVADPVHLPRQISFLALVVLELGLHSWFADSLTA

QSEAVCQAAYSSPWFQHSIGVQRTVAFVMMRAQRPVRIKAGPFANLSLELFGAIMRTSYSYLTLLRQVYD

ED

>ZnevOR28

MKKSSEEPIPKWLAKEDPLSLNNRLLSYCGLLPANTDAEERRNLVICTVMIILNLTMITG

AFIQAYFSHTNFMEIIECGTVCITQLKCLLKFLTMLICHKDLRYVINNVTRNFYVHEKLF

KEEIISKIKAGKKVAWWITVPYTSMFILTIGLIAAEQIVAAIKHADRFSAEGNETEVTGV

YHRKLPLKIWLPINVKESPSYEIGFMYQVVCFTFEIYSTCIIDTFIVVHVMFASIQYELL

GEMIQLSADKVAMLLRVRSESSQQDSEHKAGSLHSEYETETVDKQNTKLSNGEVTYISNK

ENLMSTPTTDAFRREMNIYLGRCIKHHQSLLKYIVHLNILSSPIQFLQALTSSLLICTLG

FTAIVSGSTAILPKVLMYTFDALLQIGLPCWFSTQLRVQGERIAEAAYACEWYNEPASFQ

KSVGFVIMRAQQPVVLTMGPFGTVSLELFAAVVQSAYSYLALLRNVYAEQ

>ZnevOR29

MENKPMESMGLCVAVLKIGGLWHTRDMTQRNAILYNMLRTFVVIVLGAVTLQVYLYLFIM

WGSLEDMFQTTAYSISLTVLSLKYHVMLFRNEEVDNIIQTVQKNFIIHGTELSTENRQII

KNTIKLAKRVAVAYATMQTFALITYTIIGPLTSFGVPLHTENATNISYESRISDRKLSME

MWVPIDISQSPQFEIAYIYVSVTSAINSWNLVAIEVFCMTTFIYIAGQFELLCDSIRNTS

QRVMYRLNETQHPSSGNDHIDKPQKFTSDKKMKIQHSSANTESSVIPEKDEGVHPFGSAV

TMAIQEENMANKTQYFASEQNDAIIRLGRNYDSLGGETYNLDIPGSFSNYQKEMERYLME

CIKHHQKLIECSEKIDLLWKRVFFYQFLTASLLICFIGLKAISMPLNGEFAKTMEYLAAV

IFQLGLYCTFGSNLMTQSEAVFNAAYESDWNNQSKHYKNCTRMLIMRAQRPVKLTAGRFG

TLSLPLFTSMLRSSYSYLALLRQMQEH

>ZnevOR30

MSRSYLQEKLQIPILRFNVRVLQTFGLLLDNSLITNRHGHVLRKGIMYFTIFIHIQFIFG

SVIELFISNDESHQFESLGLILSYVKGAMQLYALIFSGKNFLDLIKSAEVNFSMNGKPLG

KTEIPIFDSYVQTANRLARFMWVSFVLTLSSVFFELVPTFNVSVENHANEFDSQVMGRRN

TAYKVWTPFQKLNNPYLKLDIIYEMISVTIFFIMFTTINLLTLMLIVFFAGHFNLLAECI

ENLTNETGDVTNTEPIGPKKSKSSLNEEKWESLDQQRIGVDDLKTPDSVDSKNKDSKRKK

RDKSAESLKFRNEAKLDLRQYIEHHRKLLKSSEKLNAVLSPIMFMRILSAVVIFGFDGFY

ITRTTDKVMLLRYVAGLLYSIMETGLTCWFASKLSLQSEMVGEGVYNCRWCSKPISFQRS

LRFIIMRAQRPVALSVGPFGTLSMELFARILNSAYSYFTVLTQFHEK

>ZnevOR31

MFFQKLSEKLRKQNILEINLKIFKYYGILLDDSVKRTPWEKAMDIIKQVFTTSLVVQYFL

GTAVELYLSMDNVQRAGNCFIFLISHLKNAVKLGTLIIYRKRILLLLAKIENNNYIQGIT

PTNSERSLVEKYVNLSKRIAKYVWISFFITQVSLAANMPPRPNLELIADPEEIKNIRRDS

SIKMWFPFKAIESPYFEVTAVYECITMSIYFAFTTTVNITLVGLIIHMTGQFAVLVDAIQ

NGATRVAELLSQKRRDTGYGVGAEVVRLLQDQRTGRIYPTRLESTDTEGQSEICGKSERV

IGGTEEPIEEKCFFELSAREMFHREMDRYLGCYVAHHQELLQMVELLDSVVSAIMFTQIL

TSAILFAFIGILMASATDSSIFIMAMTCLTYVMLETGLVCWIATELSTQSEEVGAAAGRC

EWFNESIRFRNSIIPIIMRAQRPARLSVGPFGTLSLELFGKIMNSAYSYFTLLKEVSGK

>ZnevOR32

MMSKSVNLGGMNRLELPESLRALNVLSFNLKLLRFFGILIDYSRSSSRWEVPLYLMLQSG

IVFLHAQHVICTMVEMYILRDRPQDVGDGFIFLIAHVKNVMKLGTLIVYRTKFISLINSL

ERNYFVHERPHTDTELPLLESYMRLTKRLAMYVWISYALTTSSIYFNTPPRPKFDIESNS

TMSGEQMTIRRESGMKEWLPFQAIETPYFELVTAYELLSMTAYFGCVTLMNITFLALIIY

TTAQFAVLANALQQAATNVSKMLNNHVNYSLSSTTVDASGSNTELSEGKQDFRPENRTAT

NYDSEDTDSSHSPRTQLIRCASRSTFFADSVKRSSGKVGFHEEMQKYLSQCVKHHQILLD

YSKLLDRSVSPVMFMQMLCTAVLLAFIGVLLSTATDSARYIRAVSFLGYVLMEFGMICWI

ATELQVQ

>ZnevOR33

MELPEDLQKQNIINLNVRLIQGCGYLREDSINTRRIIIVFMTIMKYSSIFLYISFLVGAV

VEMYKLHNTIHDLGEGIVFIVTITKTMTKLFSLIQNKEDYLHLIHSFGDIFIHDEPLTAK

QSLVIKSYLVLANKLIKCIWYSSIIMAIIFLGNVTPPDDRDRDFVPAWEASSRVTIPFQT

ANSPFYLLRVLYATAAIAIGYFITTVLNTFGFLLLIYETAQFALLADTLKHITENVTKMA

GEAEGSQPLGDAVNNLTGTRPRGFQDFLLDKRTKDYGTKKYKEKEHEAKLESDFNEMLAD

PETENMFEDCEDRFHTKMHKYLNRCIQYHQKRLTNTKLLNRLLGPLIFWQILS

>ZnevOR34

MKLMSPRMKKILGVEEHREAKELLEFNLRTLRILGLWKWDVPHWRYHKAFAFLAIIALIL

FNITQICDVYIHINSIEHITKLLLSIVFSSLVAFKNCYLLLRSDDASELIDQLQNKFFTS

GRRPTPQQTIILNRYAARAKLYTIIRTSLALCTATLWILSPIKEMLLEYAEELHTRQYGD

DVKSNSSKMSILKLPFVAYYPFDVENNFLYFILTYIYQAFCGLAVFISLPTWDMLFVSVF

IHTSGHFKALQHVLIHLREDAGEALEVDGNQLDQDVRVSEHTVSDGNKFWTAAFHLKENY

DXXKGIRNDGREMQSASMEQKLDRPMQHILKNCVQHHQVILSFVKKLEAFLKPVMLMQLL

GSVIAYCVLGFQMSVIPIHGEGTKFVTLCGSFVSALLEQGMFYWFGGELLEASSETLNAA

YHCEWYTTNIKFRKHLHILMERAKRQVKLTAGGFSTLTLENFTKVIQSSYQYFAMLKAVH

NKDTD

>ZnevOR35

MSRGELSTSLPAYQPVNHDFLAINVKVMYLVGQWNGYWQDKRNWRYSAYLGYAVFMILVM

TVHFISEVLELYVSWGDFTNFASTAWFANNYGATIIKQVFILAQADRIQSVTQRLRGGVL

SSGLRWSKEQDEIARNTNRKTRFLSIAYYAIAVSSCTGLIVISLRTSYRQLSGAANLTEV

GADTMELPLRAWFPVDMQNPQNFLLLFGFQLFIVAAGPMINIGTDTFITGLIIHACGEFR

VLKKSLRNLKQRARQLLQEDTGCRKTTSNCKISLSSVAVPESTNHTDNHFKRAKFCPEAT

EPAHENGFLNAKADVDSKVHKALVECVRHHQQLLLFVSDLEDMFSSMMFFQFLSISLRIC

LIIFHITMSDDEGLVQLTYLQILCVSFLQGLPFCWYGSDLTYQSECVARAMYETPWLEGP

LKFRRSLTIIVTRAQAPLRLTGGKIYVMSLETFQAMIYASFSFFKLLQQLSVS

>ZnevOR36

MNSSVNEEHVVNNILAVNLKLLKLLGLWQGHTASFKFSWAIKLFIFYRWALLIIMYLHTA

TEYLDIVFTWGDLENLAATGSVTLIYTTCIMKQTVFLLFEKRLQDLVHSLRSGNLATSLR

WTKQHFEILHIADRRAQAASWRYYYLCIGTIISFFITSIISSYGTAFGLEGFSQGNQTVK

TLVFKAWFPFDIQQSGYFEIAFCFQIITCTMGPAINVGMDTLLVSVIINCCGEFRVLKYS

LRTIRERAEELLASEKSFREENLSVSEMIQSKENVRTEQAGVLTVDIGCNEIFSNSQNNK

NLGQEVHFSQEDLDLKSNICLVECIQHHQLILE

>ZnevOR37

MTEESMNDGFMKDYLRVNIKTLKMVGLWNSFDLQNKSYRAYNIFSGFVSTVIFIHVATQIADLSYTWGDL

ESFSATGSTTLTYGAALVKQLCYLANRKRIYRMVHRLRDGRLSPPSKWSEEQKNIANTYDRHARTMSWCY

YSLGIGCLFCFAMTAIVSNSIQTFDDVNSTETSSRKLPYRAVFPFDIEQTGYYGIAFSFQMLVIVFGPTV

NIGLDTMFVGLVIHASGQFKILKNALRNIKQRTIKIIEIEGIVEEEPRDKTHLTVPNYNWEEDTFFLETL

QQQMLRELNRCLQHHQKILMFISELEDVFSPMMFAQFVSSSGTLCLIIFQITV

>ZnevOR38

MVDTYFRVPLFLVRLSGISVNVRKVSRLSSAYNVISTVCFYVTYLSVIMDFVVKRDDIEE

TMKNVRMSFGMAVLSWMHFYLRSNKHEIEYLIHLTESFKPEDFPNRDPDTGNLTMTGYIP

RVNKLTKYGWLFVAVFHAIQSALRMTLNRSMIFTTWYPFDASVSPVYEIVNLSQVFASIL

AISICVGFPGLYATLVCVACSQLEKLKGALLDIRQTHLTSQQDCGAETNGEEHPHTTEEV

FRHEQKKLNDCIRHHQRIILYMTALEETINFALCGLFLLLLAALCFIAFSAVTSWGDLTD

VSQALVMYCVMMSIVFVFCWLGTQLSVQAENVRDAAWGCDWVGTPVPFQRCLIFIIAAAN

KEFTLTAGKFVPVSNKTMMNMINQTVSFFMFLLHMKKKNEEPKQGE

>ZnevOR39

MVNIKTVGMTDKLFVVPVFLVRLGGIPVNMHKVPKLNCIYNGILAVCYYGTYLSVIMDFV

NKREDIQETMKNVRMIFGMAVVAWMHLYLRYIRRDIDYLIHLAESFNWEILPTRDPDSEN

VTMNEFMFRVGRLTKFAWALTIVFHGTQSALRMLTSHSMVFTTWYPFDASISPIYEIANL

KQGLASLLIISLSTGFPGLYATFICVACSQLEKLRATILDMRQTRVTSEQDRGAETDQEE

TQGQVRTSEEVFHHMQKQLNYCIRHHQQIKRYMTALEDTMNFMFCGLFLILLAALCIIAF

SAVTSWGDFTDVSQALVMYVILLCNVFVFCWLGSQLSEQAESVRDAAWGCDWVGTPVPFQ

RCLIFIIAAANKEFTLTAGKFVPVSNKTMMNMINQTMSFFMFLLHMKSKHEGKEEN

>ZnevOR41

MEHTNKDTHASHNSLSESRFKILLLLLRTTGIRLNVKSKSAVHIIYSVILAVFIHVSILS

LYVDTFVQRHQLVELMKKLRLLIATQIVTWMHFSLSYRKREVEHLIRLTDYFTWEELPTR

DPDTGYLTKAGYLPFIQKLTKYATLFAIIYHCTQTTVRIILNHDMVFASWYPLEVSESPA

YEIANITQAIQTILMIFLFIGFQSLYATFVCVACSQLEKLRAAILDIRQTYITPEQDCGA

ETNKKDGEGHPRTHEELFGHMQKQLNDCIRHHQKIKRYMEALENAMNLPMCGLFLICLST

MCFAAFSATLSWGDHVDVSQALIIYIMVSACVCQFCWLGNELSEEAENVRDAAWGCDWVG

TPVPFQRCLIFIIAAANKEFTLTAGKFVPVSNKTMMNMMNQTLSFFMFLLQMKDKSTDTS

QGA

>ZnevOR42

MKPVISSDRNLDERRFKTILVLLRLGGIPLNMKSISKVRTVYSVFIIFCYYCTMFCLAMD

TFTHRHQLDQAMKKLRVLMGMVLSLWIHFSLSYRKGELEHLIGLTDSFTWEELPTRDPNT

GYLTKAGYIPIIQRFAVYTPAVVCIFHGTQSTVRIVLSHDMVFANWYPFDFSSSPAYEIA

NFTQGIASLFMACTYFGFPSLYATLVCVACSQVEKLRAALLDIGQTRVTSEQDRGAETDR

LEGQALTTEEVSGHMQKQLNDCIRHHQKILRYIQVLEDTMNFPLCGLFLIFLSAMCFDAF

SAITSWGDHTDVSQALAVYIVQTGCMCVYCWLGNELSEGVESVRDAAWGCDWVGTPVPFQ

RCLMFIIAKANKEFILTAGKFVPVSNKTMMNMMNQSISFFMFLLHMKDKNADNNQGV

>ZnevOR43

MKTYTSCDTNLNERRFKTLLSLLRLGGIPLNIKSVSNANTAYNVFIIVCFYTTSVCLWID

SYVHRNQLVQAMKKIRILVGMQLVTWIHFSLSYRRREIEYLIRLTDTFTWEELPSRDPDT

GYLTKAGYIPLIQRLAQYVPAILIGYHSIQSTFRIILNHDMVFFTTWYPFDVSESPAYEI

ANFTQGIASLFVICLFVGFQCLYATFVCVACSQLEKLRAAILDIRQTHIILQQDCGAETN

QQDGEEHPHTPEEVFHRMQKQLNDCIRHHQQIKRYLETLESTLTHPLCGVFLILLSAMCF

VAFSAITSWGDYTDVSQALLLYSLLTSCLYVFCWLGSELSEQAESVRDAAWGCDWVGTPV

PFQRCLIFIIAAANKEFTLTADNFVPVSNKTMMNMMNQSISFFMFLLHMKDKNRDSNHGV

>ZnevOR44

MNARKINAKAVSENRFRKLLLTLRSAGIPLNTHSITTLRSAYNVIVPVSFYITCLSGFMD

FLVSSDLKELMKSSRVVFGMGVVMWQHFFLSYRKRDIEHLISLTDSFTWEEVPTRDPETG

RLTMVGYIQIAQSFARYYPACILLIHFLQSGVRMVRNREMMITKWYPFDSSASPAYEIVN

LTQVFAAILIACVFGGFLSLYATLVCVACSQLEKLKGALLDIRQTHIILQQDCGAETNGE

EHPHTPEEVFHRMQKQLNDCIRHHQQIKQFMQAFEDSWSTVQCGVFLLILTALCSVVFSF

ITSWGDHVDLVQATLIFLHWSCILILTCWLGNELSVLAESVRDAAWGCDWVGTPVPFQRC

LIFIIAVANKEFALTAGKFVPVSNSTMLNMMNQSISFFMFLLYMKDNNREANHGI

>ZnevOR45

MNARKINAKAVSENRFRKLLLTLRSAGIPINTHSTTTLRSAYNVIVPVSFYITCLSGFMD

FLVSSDLKELMKSSRVVFGMGVVMWQHFFLSYRKRDIEHLISLTDSFTWEEVPTRDPETG

RLTMVGYIQIAQSFARYYPACILLIHFPQSGVRMVRNREMMITKWYPFDSSASPAYEILN

LTQVFAAILIACVFGGFLSLYATLVCVACSQLEKLKGALLDIRQTHIILQQDCGAETNGE

EHPHTPEEVFHRMQKQLNDCIRHHQQIKQFMQAFEDSWSTVQCGVFLLILTALCSVVFSF

ITSWGDHVDLVQSTLVFLEWFCILTLTCWLGNELSVQAESVRDAAWGCDWVGAPVPFQRC

LMFIIASANKEFTLTAGKFVPVSNATMLSMMSASLSFFMFLLEMKDRK

>ZnevOR46

MTDQHLRVNGLTEQRFRKSLFTLRLVGIPLNVRSISRAHTVYNCIVVVCFYITYSACLMD

LLFNNDSLMGIMKDIRALLGMQLVVWIHLFFRFRKREIAHLLALTDSFTWEELPTKDPET

GRLTKAGYLPVTQKIASHASALIYTFHFVQSSVRIATSHDKVFPAWFPFDTTVNPAYAAV

NLVQAIGSLFITCAFAGFLSLYATFICVACSQLEKLRAAILDIRQTYITLQQECGAETNK

QDGEGHPLTPEELFGHMQKQLNDCIRHHQQIKRFMQANEDCWNFVLCALFLLMLSAMCFV

AFSAITSWGDHVDVSQAVVIYLAMLSCLCVFCWLGTELSKEAESVRDAAWGCDWVGTPVP

FQRCLMFIIAAANKEFTLTAGKFVPVSNKTMMNILSQTVSFFMFLLQMKDTTNGKKHGI

>ZnevOR48

MDVPTSHKSHTTERRFGSLLFLLRMGGVAVNMKPQSTTHVLYKAYLELCYYATFLSVFMD

CLQKTDDLKESMKKVRLLFAFGYVSWIHLSLSFNKVEIEYLLKFTENFTWEDIPARDPES

GRVTMAGRVQLVQTGAKYVAICMVVFHGIQSFVHMWQNHDMVFTTWYPFDVSNSPVYELI

NIAQFSAFVLVTGVFAGFQGLYAILVCVACSQLEKLRANLSSSGAEIATPDQDSGDNPES

DPGPDKIQEQLNDCIRHHQITQLYMQELEKSLSSALGGLFFCLLAAMCFVSFSVVTSWGD

HNDVSQALVMYVCMMSNVFVFCWLGSELSDQAQRVRDAAWNSNWVGSPVRFQRCLVFIIA

SANKEFKLTAGKYVPMANKTTLNMMNQTISFFMFLLHMKRKTEETI

>ZnevOR51

MENSTLNAGDITERRFSLLFLFLRLIGIPVNRKTSSCYRSLYNTVVSICMYNLYLEFTMD

VVTSRDLEYGFVKARFFLGACVIMWMHLYLRIRKSAFEVLFALTESFRWEDQPETDPDTK

VLTMAGLVTRLQRYMLRGIIFINGYHIIHTIITRARYHFVMFNTWHPFDNSTMLTLQIMN

ISEIFGSLAIITTLMTFTCLYGVMVCVACSQLEKLRAALLDIKQETSEHEGTFSRMQDQL

NACIRHHQEIKRFMQEIEDVFNPCFCGLFLIILITLCCLAFSGVMSWGHSADVLQGFAGY

LSMLLCVFIYCRLGTELTSQAESVGDSAWGCDWVGTPVHFQRCLAFVIATAQKEFTLTAG

KFVPVSNTTMMN

>ZnevOR56

MREEKFLSLSSTTLSEPQASTLISAMAEYRENVGGRFGVIEKLFKIAGIPVTSDEKSFLY

SVYQVLVVTSGYLTLLTTFVGILKNLDDMEYVMEVARPGFVMINLVWMHFFTSMNMNGVR

SLLFMTKHFTWSELPTRDSQTGSLTMAGWIQRIQPLVWRLNFCDWGTHFLYFVLRGVTSN

GKPFFFDAWFPLDTEDPSSYALALIIQALGSVMLGTTLFAVMGLYVALVSIGCTQLEKIQ

AALMDINQQRLPEMQTSLVQCVKHHQHVLRYMKELETTFSPVLFGPFLSVVAALCFTAYT

AIRIGGKFVEIIQIFIITAAMMFQLQAVCWFGTELNKQSGRVRDAAFGSDWVGAPVAFQR

SIRFIISVTKDFTLTAGKVIPVYQSTVMVVLNETYRFLMVLLNFVDKSSEVQ

>ZnevOR60

MLTTKMAEHDKTTRRFSYFVAAFRVTGIPLLMDRVSPLYWVYAAVATVCCYASYSAQVVD

LLKNTRDLERTMETARVVAGAGMTVWIYGFLRFRINSLERLLHLAESFVWEDLPTTDPET

GSMAAAGWIPRIESVTRRLINITLIFHFSYVIIKRAVSSKPQLSYNTWYPFDISSTIVFE

LVNISQLLAALIYTLMLLGFNGLYATLVCIACSQLQKLRANLLDIRQKQDTAEHPDPGAE

TGHEERQKVHAFYHMQDQLNDCVRHHQQILSYIRAMEETFSPMMVVIFLLDLASLCLGSF

SIVTSWGNYVQMSQGIVTYGLLLLVLAIYCWFGYELSYQAENVRDAAWGCDWVGTPVSFQ

KSIRLIISSANKEFTLTAGKIVPVTISTLVTMVNQSVSYFMFLLEVKKNIEHSKIN

>ZnevOR63

MADNKVLHKNQVTENRFRFHQIMFRIAGCPIFRTNESKVVFILSFLCFYINFVAILMDIY

VNIQDMEHAMENVRLAFPIASGLCIHQFMRLRRGAIEQLLRLTDRFTWNGMLTRDPDTGY

LTLAVWIPRIDTSAQKIFALLVFIHFIYIFFRSISSDERQLQFNSWYPFETLVSPQYELI

NATQFIGSIMCLCSFVAFISLYATLVCIACSQLEKLRASLLDIRQNRDISKDSGAETDQE

EIQEVHISQKMFNHMQKQLNDCIRHHQDILEFMRALEATMNPMMLAHFLLIAGGMCFATF

SAVTSSGRLLQLAQILIVYIAFATQLYTYCWFGTELTRQAESLSDAAWRSDWIGTPVSFQ

RCLIFIIATARRDFVLTAGKFVSVSRETMLTIINQTISYFMFLLNFKDMDTDADQ

>ZnevOR64

MRFPHRMKGDWNENERRNFEKLFRFYILWFRLICVPIKSHKPSHIYTLYSVLLTLNTYTA

IAAIMADVFQHADDLERVMENFRVIFPSLSIMWVQLSMRFQKKSMERLMYLAAAFKWEDA

QAKKPADDSFKMTAILPVIKPATLRISIFFVTFHTCHVILRLALGEGRPLSINAYYGVNV

SSSPLYEMVNVSQFFMAANCFPLFFGYTGLYAFLLTIACSQLEKLRASILNIRQRNDTSE

EDSDAENGKVKEGGKVQTSQQVFSHMQQQLNDCVRHHQDILRFMNALEEAISPVLMGQFI

IILTGMCFAAFSFAMSLGNVLNMAQSILEYTAFLVQLNAYCWFGSELTQLAESVRDAAYG

CDWVGTPVSFPKCLVFIIAAASKEFTLTAGKFVPVTRSTMLNVINQTFSYLMFLMQVKEK

NKHV

>ZnevOR65

MNRNEIQHEYNTNMETLFGFYFKWFRLAGVPLKLNKKSKLYNLYSTIMLLNAYSVVITIS

VDMFLHFDDLERIMENSRVIVPASSSLWVHMCIRFQKKALERMMHLASAFKYEDPQIRTP

ADNSFRMTALLTAIKPASLKVLAVSLGYHTFYIIIRLVAREDRPLAINAYYGIDTSNSPF

YEIINLSQFLMTTVCFPLFFGSTSLYVYVVTIACSQLEKLKAAILNINQKRDMDANFYQE

KEERQIQVPQEMFRHTQEHLNDCIRHHQQILELMNALEESASTILVCHFLLILGGTCFAA

FSFVMSFGNPLNMAQSMLVYGSFMLQLYAYCWFGSELTRLAESVRDAAWSCDWVGLPLFI

QRPLVFIIAVANKEFTLTAGKFVPVSRTTMLNVINQTVSYVMFLMQVRNREETV

>ZnevOR66

MNQNEDKRAYELKNRLGFYIFIFRLAGVDTQPQSSWKLYNVYASVAFLCAYGSSVAIAAD

MFLTDDLQHIMENMRALLGSISAFWMQNYVRFRKDAILKLMGLTDSFTWDESPAKDCHTG

KTSKGSLIPVIKNSVWKLCLFVIAFHGSYFTLQVSLGRRILSINSYYPFDWTVSPLYELV

NITQFVAASVYVCTIFGFLGLYATFTCIACTQLEKLRANIYNIRQKHDTTEQDSGAETDQ

EEEGKVYTTQQMFCHMQKQLNDCIRHHQEILKFMWELEKTMNPMLVVQFVIILAGLCLSA

FSLVTNMGDVLQMAQALLIYFGFMTEVCCICWFGDELTEEAESIRDAAWGCDWVGTPVTF

QRCILFIIVAANKKFELTAGKIVGVSNNTMANIFNQTLSYLTFLIQAKDNID

# Supplementary Data S2. GRs

>GjilGR57

MGMLFMLIFMCARITSIGFTFSSSAGLVFFGSSVMSTLLFLQLARRWPAL

AREWERVEIQLRHYNTRNLSTKFRVITAIILTVALSKWPSLSPTHFSTDK

PNKHLSAVSFPCYPWVITPKF

>GjilGR13

MIFFGIICNSAHLASQQVGSIFKTDLQSIAKRLMYTNNISAKREVQAFLH

VIDLNPPNINLGGFGDVNRELELSLLSSMVTYLVVLLQVQLSLTTPTQDC

SSNSTS

>DmGR2a

MEFGMDTLRALEPLHRACQVCNLWPWRLAPPPDSEGILLRRSRWLELYGWTVLIAATSFTVYGLFQESSVEEKQDSEST

ISSIGHTVDFIQLVGMRVAHLAALLEALWQRQAQRGFFAELGEIDRLLSKALRVDVEAMRINMRRQTSRRAVWILWGYA

VSQLLILGAKLLSRGDRFPIYWISYLLPLLVCGLRYFQIFNATQLVRQRLDVLLVALQQLQLHQKGPAVDTVLEEQEDL

EEAAMDRLIAVRLVYQRVWALVALLNRCYGLSMLMQVGNDFLAITSNCYWMFLNFRQSAASPFDILQIVASGVWSAPHL

GNVLVLSLLCDRTAQCASRLALCLHQVSVDLRNESHNALITQFSLQLLHQRLHFSAAGFFNVDCTLLYTIVGATTTYLI

ILIQFHMSESTIGSDSNGQ

>DmGR5a

MRQLKGRNRCNRAVRHLKVQGKMWLKNLKSGLEQIRESQVRGTRKNFLHDGSFHEAVAPVLAVAQCFCLMPVCGISAPT

YRGLSFNRRSWRFWYSSLYLCSTSVDLAFSIRRVAHSVLDVRSVEPIVFHVSILIASWQFLNLAQLWPGLMRHWAAVER

RLPGYTCCLQRARPARRLKLVAFVLLVVSLMEHLLSIISVVYYDFCPRRSDPVESYLLGASAQLFEVFPYSNWLAWLGK

IQNVLLTFGWSYMDIFLMMLGMGLSEMLARLNRSLEQQVRQPMPEAYWTWSRTLYRSIVELIREVDDAVSGIMLISFGS

NLYFICLQLLKSINTMPSSAHAVYFYFSLLFLLSRSTAVLLFVSAINDQAREPLRLLRLVPLKGYHPEVFRFAAELASD

QVALTGLKFFNVTRKLFLAMAGTVATYELVLIQFHEDKKTWDCSPFNLD

>DmGR8a

MSGHLGRVLQFHLRLYQVLGFHGLPLPGDGNPARTRRRLMAWSLFLLISLSALVLACLFSGEEFLYRGDMFGCANDALK

YVFAELGVLAIYLETLSSQRHLANFWWLHFKLGGQKTGLVSLRSEFQQFCRYLIFLYAMMAAEVAIHLGLWQFQALTQH

MLLFWSTYEPLVWLTYLRNLQFVLHLELLREQLTGLEREMGLLAEYSRFASETGRSFPGFESFLRRRLVQKQRIYSHVY

DMLKCFQGAFNFSILAVLLTINIRIAVDCYFMYYSIYNNVINNDYYLIVPALLEIPAFIYASQSCMVVVPRIAHQLHNI

VTDSGCCSCPDLSLQIQNFSLQLLHQPIRIDCLGLTILDCSLLTRMACSVGTYMIYSIQFIPKFSNTYM

>DmGR9a

MSLWLEHFLTGYFQLCGLVCGWSGSRLGRLLSSTFLVLILIELVGEIETYFTEENPDNESVPAYFAKVIMGVNMAYKMI

HAWIALSALFEcRRFRYLLEELPPVKATSFIYRHLILEIILFACNAFLVLSEYTIRGIYLENLRYAYSLQAVRARYLQM

MVLVDRLDGKLEQLHHRVISGSSDYKTLRLDYAHLAKVTRSLSHLFGLSLLLLNVLCLGDWIIVCNVYFMVAYLQVLPA

TLFLFGQVMFVVCPTLIKIWSICAASHRCVSKSKHLQQQLKDLPGQTPVERSQIEGFALQIMQDPIQIDVCGIYHLNLQ

TLAGMFFFILEALVIFLQFVSLVRT

>DmGR10a

MTSPDERKSFWERHEFKFYRYGHVYALIYGQVVIDYVPQRALKRGVKVLLIAYGHLFSMLLIVVLPGYFCYHFRTLTDT

LDRRLQLLFYVSFTNTAIKYATVIVTYVANTVHFEAINQRCTMQRTHLEFEFKNAPQEPKRPFEFFMYFKFCLINLMMM

IQVCGIFAQYGEVGKGSVSQVRVHFAIYAFVLWNYTENMADYCYFINGSVLKYYRQFNLQLGSLRDEMDGLRPGGMLLH

HCCELSDRLEELRRRCREIHDLQRESFRMHQFQLIGLMLSTLINNLTNFYTLFHMLAKQSLEEVSYPVVVGSVYATGFY

IDTYIVALINEHIKLELEAVALTMRRFAEPREMDERLTREIEHLSLELLNYQPPMLCGLLHLDRRLVYLIAVTAFSYFI

TLVQFDLYLRKKS

>DmGR10b

MRVGKLCRLALRFWMGLILVLGFSSHYYNPTRRRLVYSRILQTYDWLLMVINLGAFYLYYRYAMTYFLEGMFRRQGFVN

QVSTCNVFQQLLMAVTGTWLHFLFERHVCQTYNELSRILKHDLKLKEHSRFYCLAFLAKVYNFFHNFNFALSAIMHWGL

RPFNVWDLLANLYFVYNSLARDAILVAYVLLLLNLSEALRLNGQQEHDTYSDLMKQLRRRERLLRIGRRVHRMFAWLVA

IALIYLVFFNTATIYLGYTMFIQKHDALGLRGRGLKMLLTVVSFLVILWDVVLLQVICEKLLAEENKICDCPEDVASSR

TTYRQWEMSALRRAITRSSPENNVLGMFRMDMRCAFALISCSLSYGIIIIQIGYIPG

>DmGR21aNEW

MSFWAVSRGLTPPSKVVPMLNPNQRQFLEDEVRYREKLKLMARGDAMEEVYVRKQETVDDPLELDKHDSFYQTTKSLLV

LFQIMGVMPIHRNPPEKNLPRTGYSWGSKQVMWAIFIYSCQTTIVVLVLRERVKKFVTSPDKRFDEAIYNVIFISLLFT

NFLLPVASWRHGPQVAIFKNMWTNYQYKFFKTTGSPIVFPNLYPLTWSLCVFSWLLSIAINLSQYFLQPDFRLWYTFAY

YPIIAMLNCFCSLWYINCNAFGTASRALSDALQTTIRGEKPAQKLTEYRHLWVDLSHMMQQLGRAYSNMYGMYCLVIFF

TTIIATYGSISEIIDHGATYKEVGLFVIVFYCMGLLYIICNEAHYASRKVGLDFQTKLLNINLTAVDAATQKEVEMLLV

AINKNPPIMNLDGYANINRELITTNISFMATYLVVLLQFKITEQRRIGQQQA

>DmGR22a

MSQPKRIHRICKGLARFTIRATLYGSWVLGLFPFTFDSRKRRLNRSKWLLAYGLVLNLTLLVLSMLPSTDDHNSVKVEV

FQRNPLVKQVEELVEVISLITTLVTHLRTFSRSSELVEILNELLVLDKNHFSKLMLSECHTFNRYVIEKGLVIILEIGS

SLVLYFGIPNSKIVVYEAVCIYIVQLEVLMVVMHFHLAVIYIYRYLWIINGQLLDMASRLRRGDSVDPDRIQLLLWLYS

RLLDLNHRLTAIYDIQVTLFMATLFSVNIIVGHVLVICWINITRFSLLVIFLLFPQALIINFWDLWQGIAFCDLAESTG

KKTSMILKLFNDMENMDQETERRVAEFTLFCSHRRLKVCHLGLLDINYEMGFRMIITNILYVVFLVQFDYMNLKFKTD

>DmGR22b

MFGSSREIRPYLARQMLKTTLYGSWLLGIFPFTLDSGKRIRQLRRSRCLTLYGLVLNYFLIFTLIRLAFEYRKHKLEAF

KRNPVLEMINVVIGIINVLSALIVHFMNFWGSRKVGEICNELLILEYQDFEGLNGRNCPNFNCFVIQKCLTILGQLLSF

FTLNFALPGLEFHICLVLLSCLMEFSLNLNIMHYHVGVLLIYRYVWLINEQLKDLVSQLKLNPETDFSRIHQFLSLYKR

LLELNRKLVIAYEYQMTLFIIAQLSGNIVVIYFLIVYGLSMRTYSIFLVAFPNSLLINIWDFWLCIAACDLTEKAGDET

AIILKIFSDLEHRDDKLEMSVNEFAWLCSHRKFRFQLCGLFSMNCRMGFKMIITTFLYLVYLVQFDYMNL

>DmGR22cNEW

MFASRSDLQSRLCWIILKATLYSSWFLGVFPYRFDSRNGQLKRSRFLLFYGLILNFFLLLKMVCSGGQKLGIPEAFARN

SVLENTHYTTGMLAVFSCVVIHFLNFWGSTRVQDLANELLVLEYQQFASLNETKCPKFNSFVIQKWLSVIGLLLSYLSI

AYGLPGNNFSVEMVLINSLVQFSFNCNIMHYYIGVLLIYRYLWLINGQLLEMVTNLKLDCSVDSSRIRKYLSLYRRLLE

LKGYMVATYEYHMTLVLTTGLASNFLAIYSWIVLDISMNINFIYLLIFPLFLLVNVWNLWLSIAASDLAENAGKSTQTV

LKLFADLEVKDIELERSVNEFALLCGHCQFNFHVCGLFTINYKMGFQMIITSFLYLIYMIQFDFMNL

>DmGR22d

MFRPRCGLRQKFVYVILKSILYSSWLLGIFPFKYEPKKRRLRRSMWLIPFGVVISSSLLILMVKQSAEDREHGIMLDVF

QRNALLYQISSLMGVVGVVSICTVHLRTLWRSKHLEEIYNGLMLLEAKYFCSNAVECPAFDGYVIQKGVVIVVGLLAPW

MVHFGMPDSKLPVLNVLVVSMVKLGTLLLALHYHLGVVIIYRFVWLINRELLSLVCSLRGNHKGSSSRVRFLLKLYNKL

VNLYSKLADCYDCQTVLMMAIFLAANIIVCFYMIVYRISLSKMSFFVMLIMFPLAIANNFMDFWLSMKVCDLLQKTGRQ

TSMILKLFNDIENMDKDLEISISDFALYCSHRRFKFLHCGLFHVNREMGFKMFVASVLYLLYLVQFDYMNL

>DmGR22e

MFRPSGSGYRQKWTGLTLKGALYGSWILGVFPFAYDSWTRTLRRSKWLIAYGFVLNAAFILLVVTNDTESETPLRMEVF

HRNALAEQINGIHDIQSLSMVSIMLLRSFWKSGDIERTLNELEDLQHRYFRNYSLEECISFDRFVLYKGFSVVLELVSM

LVLELGMSPNYSAQFFIGLGSLCLMLLAVLLGASHFHLAVVFVYRYVWIVNRELLKLVNKMAIGETVESERMDLLLYLY

HRLLDLGQRLASIYDYQMVMVMVSFLIANVLGIYFFIIYSISLNKSLDFKILVFVQALVINMLDFWLNVEICELAERTG

RQTSTILKLFNDIENIDEKLERSITDFALFCSHRRLRFHHCGLFYVNYEMGFRMAITSFLYLLFLIQFDYWNL

>DmGR22f

MKMFQPRRGFSCHLAWFMLQTTLYASWLLGLFPFTFDSRRKQLKRSRWLLLYGFVLHSLAMCLAMSSHLASKQRRKYNA

FERNPLLEKIYMQFQVTTFFTISVLLLMNVWKSNTVRKIANELLTLEGQVKDLLTLKNCPNFNCFVIKKHVAAIGQFVI

SIYFCLCQENSYPKILKILCCLPSVGLQLIIMHFHTEIILVYRYVWLVNETLEDSHHLSSSRIHALASLYDRLLKLSEL

VVACNDLQLILMLIIYLIGNTVQIFFLIVLGVSMNKRYIYLVASPQLIINFWDFWLNIVVCDLAGKCGDQTSKVLKLFT

DLEHDDEELERSLNEFAWLCTHRKFRFQLCGLFSINHNMGFQMIITSFLYLVYLLQFDFMNL

>DmGR23aA

MKTLECLTRRFLEVIFSVLALVPLPPISQLGWLFLSLAIRCCWIVYFIYLLDVAISFSWVAIENVGNAVGTMLFVGNSV

LGFALLLESVLKQKTHSQLEDLRVQTELQLQRLGMFGRSRHAAYLLPLIGVQFTCDLVRLATNFGETVSPVFCISLPLM

WLLRYRYVQLVQHVMDLNQRSIHLRRSLLSMASGNDLWQPYGVQECLQLQTLRTTYERIFECYETFSDCYGWGMLGLHL

LTSFQFVTNAYWMIMGIYDGGNVRSLIFNGATGIDFGTPIATLFWHGDSGAENGRQIGCLISKLVKPQGSKLYNDLVSE

FSLQTLHQRFVVTAKDFFSLNLHLLSSMFAAVVTYLVILIQFMFAERSSTRGSG

>DmGR23aB

MFPPTRVQASSRVVLKIFHFILVAFSLRSRRLSRLVLWLQFLGWLTWFISMWTQSVIYAQTIDCTLDCSLRHILTFFQT

VSHAFIVVTSFLDGFRIKQDQLDEPIAFEDSDPWLAFTVLAMLVPTLGVEYLVCSNAPEYAFRIRIYHLKTLPSFLALQ

VQIISFILEVMKVNIRVRQTKLQLLILARELSCRWPQRKQKPQFSDQQAHRVKDLKRRYNDLHYLFVRINGYFGGSLLT

IIIVHFAIFVSNSYWLFVDIRTRPWRIYAILLNLGFIFNVALQMAAACWHCQQSYNLGRQIGCLISKLVKPQGSKLYND

LVSEFSLQTLHQRFVVTAKDFFSLNLHLLSSMFAAVVTYLVILIQFMFAERSSTRGSG

>DmGR28a

MAFKLWERFSQADNVFQALRPLTFISLLGLAPFRLNLNPRKEVQTSKFSFFAGIVHFLFFVLCFGISVKEGDSIIGYFF

QTNITRFSDGTLRLTGILAMSTIFGFAMFKRQRLVSIIQNNIVVDEIFVRLGMKLDYRRILLSSFLISLGMLLFNVIYL

CVSYSLLVSATISPSFVTFTTFALPHINISLMVFKFLCTTDLARSRFSMLNEILQDILDAHIEQLSALELSPMHSVVNH

RRYSHRLRNLISTPMKRYSVTSVIRLNPEYAIKQVSNIHNLLCDICQTIEEYFTYPLLGIIAISFLFILFDDFYILEAI

LNPKRLDVFEADEFFAFFLMQLIWYIVIIVLIVEGSSRTILHSSYTAAIVHKILNITDDPELRDRLFRLSLQLSHRKVL

FTAAGLFRLDRTLIFTITGAATCYLIILIQFRFTHHMDDTSSNSTNNLHSIHLGD

>DmGR28bA

MIRCGLDIFRGCRGRFRYWLSARDCYDSISLMVAIAFALGITPFLVRRNALGENSLEQSWYGFLNAIFRWLLLAYCYSY

INLRNESLIGYFMRNHVSQISTRVHDVGGIIAAVFTFILPLLLRKYFLKSVKNMVQVDTQLERLRSPVNFNTVVGQVVL

VILAVVLLDTVLLTTGLVCLAKMEVYASWQLTFIFVYELLAISITICMFCLMTRTVQRRITCLHKVLKNLAHQWDTRSL

KAVNQKQRSLQCLDSFSMYTIVTKDPAEIIQESMEIHHLICEAAATANKYFTYQLLTIISIAFLIIVFDAYYVLETLLG

KSKRESKFKTVEFVTFFSCQMILYLIAIISIVEGSNRAIKKSEKTGGIVHSLLNKTKSAEVKEKLQQFSMQLMHLKINF

TAAGLFNIDRTLYFTISGALTTYLIILLQFTSNSPNNGYGNGSSCCETFNNMTNHTL

>DmGR28bB

MSALRRVRKYFISSQVYEALRPLFFLTFLYGLTPFHVVRRKMGESYLKMSCFGVFNIFIYICLCGFCYISSLRQGESIV

GYFFRTEISTIGDRLQIFNGLIAGAVIYTSAILKRCKLLGTLTILHSLDTNFSNIGVRVKYSRIFRYSLLVLIFKLLIL

GVYFVGVFRLLVSLDVTPSFCVCMTFFLQHSVVSIAICLFCVIAFSFERRLSIINQVLKNLAHQWDTRSLKAVNQKQRS

LQCLDSFSMYTIVTKDPAEIIQESMEIHHLICEAAATANKYFTYQLLTIISIAFLIIVFDAYYVLETLLGKSKRESKFK

TVEFVTFFSCQMILYLIAIISIVEGSNRAIKKSEKTGGIVHSLLNKTKSAEVKEKLQQFSMQLMHLKINFTAAGLFNID

RTLYFTISGALTTYLIILLQFTSNSPNNGYGNGSSCCETFNNMTNHTL

>DmGR28bC

MDIEMAKEPVNPTDTPDIEVTPGLCQPLRRRFRRFVTAKQLYECLRPVFHVTYIHGLTSFYISCDTKTGKKAIKKTIFG

YINGIMHIAMFVFAYSLTIYNNCESVASYFFRSRITYFGDLMQIVSGFIGVTVIYLTAFVPNHRLERCLQKFHTMDVQL

QTVGVKIMYSKVLRFSYMVLISMFLVNVLFTGGTFSVLYSSEVAPTMALHFTFLIQHTVIAIAIALFSCFTYLVEMRLV

MVNKVLKNLAHQWDTRSLKAVNQKQRSLQCLDSFSMYTIVTKDPAEIIQESMEIHHLICEAAATANKYFTYQLLTIISI

AFLIIVFDAYYVLETLLGKSKRESKFKTVEFVTFFSCQMILYLIAIISIVEGSNRAIKKSEKTGGIVHSLLNKTKSAEV

KEKLQQFSMQLMHLKINFTAAGLFNIDRTLYFTISGALTTYLIILLQFTSNSPNNGYGNGSSCCETFNNMTNHTL

>DmGR28bD

MSFYFCEIFKPRDAFGAEQTLLLYTYLLGLTPFRLRGQAGERQFHLSKIGYLNAFLQLSFFSYCFLAALIEQQSIVGYF

FKSEISQMGDSLQKFIGMTGMSILFLCSSIRVRLLIHIWDRISYIDDRFLNLGVCFNYPAIMRLRLLQIFLINGVQLGY

LISSNWMLLGNDVRPIYTAIVAFYVPQIFLLSIVMLFNATLHRLWQHFTVLNQVLKNLAHQWDTRSLKAVNQKQRSLQC

LDSFSMYTIVTKDPAEIIQESMEIHHLICEAAATANKYFTYQLLTIISIAFLIIVFDAYYVLETLLGKSKRESKFKTVE

FVTFFSCQMILYLIAIISIVEGSNRAIKKSEKTGGIVHSLLNKTKSAEVKEKLQQFSMQLMHLKINFTAAGLFNIDRTL

YFTISGALTTYLIILLQFTSNSPNNGYGNGSSCCETFNNMTNHTL

>DmGR28bE

MWLLRRSVGKSGNRPHDVYTCYRLTIFMALCLGIVPYYVSISSEGRGKLTSSYIGYINIIIRMAIYMVNSFYGAVNRDT

LMSNFFLTDISNVIDALQKINGMLGIFAILLISLLNRKELLKLLATFDRLETEAFPRVGVAMHQVAANKKMNRLVIILV

GSMVAYITCSFLMISLRDTTTFSISAVISFFSPHFIVCAVSFLAGNVMIKLRIYLSALNEVLKNLAHQWDTRSLKAVNQ

KQRSLQCLDSFSMYTIVTKDPAEIIQESMEIHHLICEAAATANKYFTYQLLTIISIAFLIIVFDAYYVLETLLGKSKRE

SKFKTVEFVTFFSCQMILYLIAIISIVEGSNRAIKKSEKTGGIVHSLLNKTKSAEVKEKLQQFSMQLMHLKINFTAAGL

FNIDRTLYFTISGALTTYLIILLQFTSNSPNNGYGNGSSCCETFNNMTNHTL

>DmGR32aNEW

MSPNTWVIEMPTQKTRSHPYPRRISPYRPPVLNRDAFSRDAPPMPARNHDHPVFEDIRTILSVLKASGLMPIYEQVSDY

EVGPPTKTNEFYSFFVRGVVHALTIFNVYSLFTPISAQLFFSYRETDNVNQWIELLLCILTYTLTVFVCAHNTTSMLRI

MNEILQLDEEVRRQFGANLSQNFGFLVKFLVGITACQAYIIVLKIYAVQGEITPTSYILLAFYGIQNGLTATYIVFASA

LLRIVYIRFHFINQLLNGYTYGQQHRRKEGGARARRQRGDVNPNVNPALMEHFPEDSLFIYRMHNKLLRIYKGINDCCN

LILVSFLGYSFYTVTTNCYNLFVQITGKGMVSPNILQWCFAWLCLHVSLLALLSRSCGLTTTEANATSQILARVYAKSK

EYQNIIDKFLTKSIKQEVQFTAYGFFAIDNSTLFKIFSAVTTYLVILIQFKQLEDSKVEDPVPEQT

>DmGR33aNEW

MIQIMNWFSMVIGLIPLNRQQSETNFILDYAMMCIVPIFYVACYLLINLSHIIGLCLLDSCNSVCKLSSHLFMHLGAFL

YLTITLLSLYRRKEFFQQFDARLNDIDAVIQKCQRVAEMDKVKVTAVKHSVAYHFTWLFLFCVFTFALYYDVRSLYLTF

GNLAFIPFMVSSFPYLAGSIIQGEFIYHVSVISQRFEQINMLLEKINQEARHRHAPLTVFDIESEGKKERKTVTPITVM

DGRTTTGFGNENKFAGEMKRQEGQQKNDDDDLDTSNDEDEDDFDYDNATIAENTGNTSEANLPDLFKLHDKILALSVIT

NGEFGPQCVPYMAACFVVSIFGIFLETKVNFIVGGKSRLLDYMTYLYVIWSFTTMMVAYIVLRLCCNANNHSKQSAMIV

HEIMQKKPAFMLSNDLFYNKMKSFTLQFLHWEGFFQFNGVGLFALDYTFIFSTVSAATSYLIVLLQFDMTAILRNEGLM

S

>DmGR36a

MFDWVGLLLKVLYYYGQIIGLINFEIDWQRGRVVAAQRGILFAIAINVLICMVLLLQISKKFNLDVYFGRANQLHQYVI

IVMVSLRMASGISAILNRWRQRAQLMRLVECVLRLFLKKPHVKQMSRWAILVKFSVGVVSNFLQMAISMESLDRLGFNE

FVGMASDFWMSAIINMAISQHYLVILFVRAYYHLLKTEVRQAIHESQMLSEIYPRRAAFMTKCCYLADRIDNIAKLQNQ

LQSIVTQLNQVFGIQGIMVYGGYYIFSVATTYITYSLAINGIEELHLSVRAAALVFSWFLFYYTSAILNLFVMLKLFDD

HKEMERILEERTLFTSALDVRLEQSFESIQLQLIRNPLKIEVLDIFTITRSSSAAMIGSIITNSIFLIQYDMEYF

>DmGR36b

MVDWVVLLLKAVHIYCYLIGLSNFEFDCRTGRVFKSRRCTIYAFMANIFILITIIYNFTAHGDTNLLFQSANKLHEYVI

IIMSGLKIVAGLITVLNRWLQRGQMMQLVKDVIRLYMINPQLKSMIRWGILLKAFISFAIELLQVTLSVDALDRQGTAE

MMGLLVKLCVSFIMNLAISQHFLVILLIRAQYRIMNAKLRMVIEESRRLSFLQLRNGAFMTRCCYLSDQLEDIGEVQSQ

LQSMVGQLDEVFGMQGLMAYSEYYLSIVGTSYMSYSIYKYGPHNLKLSAKTSIIVCILITLFYLDALVNCNNMLRVLDH

HKDFLGLLEERTVFASSLDIRLEESFESLQLQLARNPLKINVMGMFPITRGSTAAMCASVIVNSIFLIQFDMEFF

>DmGR36c

MDLESFLLGAVYYYGLFIGLSNFEFDWNTGRVFTKKWSTLYAIALDSCIFALYIYHWTGNTNIVNAIFGRANMLHEYVV

AILTGLRIVTGLFTLILRWYQRCKMMDLASKVVRMYVARPQVRRMSRWGILTKFIFGSITDGLQMAMVLSAMGSVDSQF

YLGLGLQYWMFVILNMAMMQQHMIMLFVRTQFQLINTELRQVIDEAKDLLLSPRHQGVFMTKCCSLADQIENIARIQSQ

LQTIMNQMEEVFGIQGAMTYGGYYLSSVGTCYLAYSILKHGYENLSMTLSTVILAYSWCFFYYLDGMLNLSVMLHVQDD

YWEMLQILGKRTIFVGLDVRLEEAFENLNLQLIRNPLKITVVKLYDVTRSNTMAMFGNLITHSIFLIQYDIEHF

>DmGR39aA

MGTRNRKLLFFLHYQRYLGLTNLDFSKSLHIYWLHGTWSSTAIQIVVVGVFMAALLGALAESLYYMETKSQTGNTFDNA

VILTTSVTQLLANLWLRSQQKSQVNLLQRLSQVVELLQFEPYAVPQFRWLYRIWLLVCLIYGAMVTHFGINWLTTMQIS

RVLTLIGFVYRCVLANFQFTCYTGMVVILKKLLQVQVKQLEHLVSTTTISMAGVAGCLRTHDEILLLGQRELIAVYGGV

ILFLFIYQVMQCILIFYISNLEGFHSSNDLVLIFCWLAPMLFYLILPLVVNDIHNQANKTAKMLTKVPRTGTGLDRMIE

KFLLKNLRQKPILTAYGFFALDKSTLFKLFTAIFTYMVILVQFKEMENSTKSINKF

>DmGR39aB

MDFQPGELCAYYRLCRYLGIFCIDYNPTKKKFRLRRSVLCYIVHFALQAYLVGCISVMVTYWRRCFKSELTTTGNHFDR

LVMVIALGILVVQNAWLIWLQAPHLRIVRQIEFYRRNHLANVRLLLPKRLLWLIIATNVVYMANFIKTCIFEWLTDASR

LFVITSLGFPLRYLVTSFTMGTYFCMVHIVRLVLDWNQSQINAIIDESADLKMTSPNRLRLRVCLEMHDRLMLLCNDEI

SLVYGFIAWLSWMFASLDVTGVIYLTMVIQTKKSIVLKLITNVVWLSPTFMTCAASFMSNRVTIQANKTAKMLTKVPRT

GTGLDRMIEKFLLKNLRQKPILTAYGFFALDKSTLFKLFTAIFTYMVILVQFKEMENSTKSINKF

>DmGR39aC

MKRNAFEELRVQLRTLKWLGVLRFTIDFNKCLVRENASEERSAWLYLIGVVGITCSLIVYSTYFPSHFIMGKHNTTGNC

YALINIRSCSIVTMLIYTQLYIQRFRFVALLQSILRFNQISGSHREEGRFAFYYYTHLSLLIICMLNYAYGYWTAGVRL

TTIPIYLLQYGFSYLFLGQVVVLFACIQQILLSILKYYNQVVLKNIKSSKESREFYYNFCKYNQVIWLSYTEINHCFGL

LLLLVTGLILLITPSGPFYLVSTIFEGRFRQNWQFSLMSFTAILWSLPWIVLLVLAMGRNDVQKEANKTAKMLTKVPRT

GTGLDRMIEKFLLKNLRQKPILTAYGFFALDKSTLFKLFTAIFTYMVILVQFKEMENSTKSINKF

>DmGR39aD

MSKVCRDLRIYLRLLHIMGMMCWHFDSDHCQLVATSGSERYAVVYAGCILVSTTAGFIFALLHPSRFHIAIYNQTGNFY

EAVIFRSTCVVLFLVYVILYAWRHRYRDLVQHILRLNRRCASSCTNQQFLHNIILYGMLTILCFGNYLHGYTRAGLATL

PLALCMLVYIFAFLVLCLLLMFFVSLKQVMTAGLIHYNQQLCQGDLISGLRGRQQILKLCGGELNECFGLLMLPIVALV

LLMAPSGPFFLISTVLEGKFRPDECLIMLLTSSTWDTPWMIMLVLMLRTNGISEEANKTAKMLTKVPRTGTGLDRMIEK

FLLKNLRQKPILTAYGFFALDKSTLFKLFTAIFTYMVILVQFKEMENSTKSINKF

>DmGR39b

MLYSFHPYLKYFALLGLVPWSESCAQSKFVQKVYSAILIILNAVHFGISIYFPQSAELFLSLMVNVIVFVARIVCVTVI

ILQVMVHYDDYFRFCREMKYLGLRLQCELKIHVGRLKWQSYAKILALGIGFLVTVLPSIYVALSGSLLYFWSSLLSILI

IRMQFVLVLLNVELLGHHVSLLGIRLQNVLECHLMGANCTLDGNANRLCSLEFLLALKQSHMQLHYLFTHFNDLFGWSI

LGTYVVLFSDSTVNIYWTQQVLVEVYEYKYLYATFSVFVPSFFNILVFCRCGEFCQRQSVLIGSYLRNLSCHPSIGRET

SYKDLLMEFILQVEQNVLAINAEGFMSTDNSLLMSILAAKVTYLIVLMQFSSV

>DmGR43aNEW

MEISQPSIGIFYISKVLALAPYATVRNSKGRVEIGRSWLFTVYSATLTVVMVFLTYRGLLFDANSEIPVRMKSATSKVV

TALDVSVVVMAIVSGVYCGLFSLNDTLELNDRLNKIDNTLNAYNNFRRDRWRALGMAAVSLLAISILVGLDVGTWMRIA

QDMNIAQSDTELNVHWYIPFYSLYFILTGLQVNIANTAYGLGRRFGRLNRMLSSSFLAENNATSAIKPQKVSTVKNVSV

NRPAMPSALHASLTKLNGETLPSEAAAKNKGLLLKSLADSHESLGKCVHLLSNSFGIAVLFILVSCLLHLVATAYFLFL

ELLSKRDNGYLWVQMLWICFHFLRLLMVVEPCHLAARESRKTIQIVCEIERKVHEPILAEAVKKFWQQLLVVDADFSAC

GLCRVNRTILTSFASAIATYLVILIQFQRTNG

>DmGR47a

MAFTSSQLCSLLTKFTALNGLNTYYFDTKTNAFRVSSKLKIYCAIHHALCVLALAHMSYSTASNLRVSVTVLTIGGTMA

CCVKSCWEKAQGIRNLARGLVTMEQKYFAGRPSGLLLKCRYYIKITFGSITLLRIHLIQPIYMRRLLPSQFYLNVGAYW

LLYNMLLAAVLGFYFLLWEMCRIQKLINDQMTLILARSGQRNRLKKMQHCLRLYSKLLLLCDQFNSQLGHVAIWVLACK

SWCQITFGYEIFQMVAAPKSIDLTMSMRVFVIFTYIFDAMNLFLGTDISELFSTFRADSQRILRETSRLDRLLSMFALK

LALHPKRVVLLNVFTFDRKLTLTLLAKSTLYTICCLQNDYNKLKA

>DmGR47bNEW

MQRDDGFVYCYGNLYSLLLYWGLVTIRVRSPDRGGAFSNRWTVCYALFTRSFMVICFMATVMTKLRDPEMSAAMFGHLS

PLVKAIFTWECLSCSVTYIEYCLSLDLQKDRHLKLVARMQEFDRSVLMVFPHVQWNYRRARLKYWYGTVIVGFCFFSFS

ISLIFDTTRCTCGIPSTLLMAFTYTLLTSSVGLLGFVHIGIMDFIRVRLRLVQQLLHQLYQADDSSEVHERIAYLFEMS

KRCSFLLAELNGVFGFAAAAGIFYDFTIMTCFVYVICQKLLEREPWDPEYVYMLLHVAIHTYKVVITSTYGYLLLREKR

NCMHLLSQYSRYFSGQDVARRKTEDFQHWRMHNRQAAMVGSTTLLSVSTIYLVYNGMANYVIILVQLLFQQQQIKDHQL

TSGKDVDIVGPMGPITHMD

>DmGR57a

MAVLYFFREPETVFDCAAFICILQFLMGCNGFGIRRSTFRISWASRIYSMSVAIAAFCCLFGSLSVLLAEEDIRERLAK

ADNLVLSISALELLMSTLVFGVTVISLQVFARRHLGIYQRLAALDARLMSDFGANLNYRKMLRKNIAVLGIVTTIYLMA

INSAAVQVASGHRALFLLFALCYTIVTGGPHFTGYVHMTLAEMLGIRFRLLQQLLQPEFLNWRFPQLHVQELRIRQVVS

MIQELHYLIQEINRVYALSLWAAMAHDLAMSTSELYILFGQSVGIGQQNEEENGSCYRMLGYLALVMIPPLYKLLIAPF

YCDRTIYEARRCLRLVEKLDDWFPQKSSLRPLVESLMSWRIQAKIQFTSGLDVVLSRKVIGLFTSILVNYLLILIQFAM

TQKMGEQIEQQKIALQEWIGF

>DmGR58a

MLLKFMYIYGIGCGLMPAPLKKGQFLLGYKQRWYLIYTACLHGGLLTVLPFTFPHYMYDDSYMSSNPVLKWTFNLTNIT

RIMAMFSGVLLMWFRRKRILNLGENLILHCLKCKTLDNRSKKYSKLRKRVRNVLFQMLLVANLSILLGALILFRIHSVQ

RISKTAMIVAHITQFIYVVFMMTGICVILLVLHWQSERLQIALKDLCSFLNHEERNSLTLSENKANRSLGKLAKLFKLF

AENQRLVREVFRTFDLPIALLLLKMFVTNVNLVYHGVQFGNDTIETSSYTRIVGQWVVISHYWSAVLLMNVVDDVTRRS

DLKMGDLLREFSHLELVKRDFHLQLELFSDHLRCHPSTYKVCGLFIFNKQTSLAYFFYVLVQVLVLVQFDLKNKVEKRN

>DmGR58b

MLHPKLGRVMNVVYYHSVVFALMSTTLRIRSCRKCLRLEKVSRTYTIYSFFVGIFLFLNLYFMVPRIMEDGYMKYNIVL

QWNFFVMLFLRAIAVVSCYGTLWLKRHKIIQLYKYSLIYWKRFGHITRAIVDKKELLDLQESLARIMIRKIILLYSAFL

CSTVLQYQLLSVINPQIFLAFCARLTHFLHFLCVKMGFFGVLVLLNHQFLVIHLAINALHGRKARKKWKALRSVAAMHL

KTLRLARRIFDMFDIANATVFINMFMTAINILYHAVQYSNSSIKSNGWGILFGNGLIVFNFWGTMALMEMLDSVVTSCN

NTGQQLRQLSDLPKVGPKMQRELDVFTMQLRQNRLVYKICGIVELDKPACLSYIGSILSNVIILMQFDLRRQRQPINDR

QYLIHLMKNKTKV

>DmGR58c

MNQYFLLHTYFQVSRLIGLCNLHYDSSNHRFILNHVPTVVYCVILNVVYLLVLPFALFVLTGNIYHCPDAGMFGVVYNV

VALTKLLTMLFLMSSVWIQRRRLYKLGNDLMKMLHKFRFNLGNDCRNRCLCKGLLTSSRFVLLTQQLLTRDSVVNCESN

SSLRQAMVPYQSAAIVYALIMILLMSYVDMTVYMVEVAGNWLLVNMTQGVREMVQDLEVLPERNGIPREMGLMQILAAW

RKLWRRCRRLDALLKQFVDIFQWQVLFNLLTTYIFSIAVLFRLWIYLEFDKNFHLWKGILYAIIFLTHHVEIVMQFSIF

EINRCKWLGLLEDVGNLWDINYSGRQCIKSSGTILSRKLEFSLLYMNRKLQLNPKRVRRLHIVGLFDLSNLTVHNMTRS

IITNVLVLCQIAYKKYG

>DmGR59a

MKRIGQAYNVYAVFIGMTSYETMGGKFRQSRITRIYCLLINAIFLTLLPSAFWKSAKLLSTADWMPSYMRVTPYIMCTI

NYAAIAYTLISRCYRDAMLMDLQRIVLEVNREMLRTGKKMNSLLRRMFFLKTFTLTYSCLSYILAVFIYQWKAQNWSNL

CNGLLVNISLTILFVNTFFYFTSLWHIARGYDFVNQQLNEIVACQSMDLERKSKELRGLWALHRNLSYTARRINKHYGP

QMLAMRFDYFIFSIINACIGTIYSTTDQEPSLEKIFGSLIYWVRSFDFFLNDYICDLVSEYQMQPKFFAPESSMSNELS

SYLIYESSTRLDLLVCGLYRVNKRKWLQMVGSIVVHSSMLFQFHLVMRGGL

>DmGR59b

MVYWMIKLYFRYSLAIGITSQQFSNRKFFSTLFSRTYALIANIVTLIMLPIVMWQVQLVFQQKKTFPKLILITNNVREA

VSFLVILYTVLSRGFRDTAFKEMQPLLLTLFREEKRCGFKGIGGVRRSLRILLFVKFFTLSWLCVTDVLFLLYSTDALI

WVNVLRFFFKCNTNNILEMVPMGYFLALWHIARGFDCVNRRLDQIVKSKSTRKHRELQHLWLLHACLTKTALNINKIYA

PQMLASRFDNFVNGVIQAYWGAVFTFDLSTPFFWVVYGSVQYHVRCLDYYLIDNMCDVAVEYHDSAKHSWSEVRWTKEI

SSYVIYANSTKLQLWSCGLFQANRSMWFAMISSVLYYILVLLQFHLVMRK

>DmGR59c

MVDLVKTILLIAYWYGLAVGVSNFEVDWLTGEAIATRRTTIYAAVHNASLITLLILFNLGNNSLKSEFISARYLHEYFF

MLMTAVRISAVLLSLITRWYQRSRFIRIWNQILALVRDRPQVVRGRWYRRSIILKFVFCVLSDSLHTISDVSAQRKRIT

ADLIVKLSLLATLTTIFNMIVCQYYLAMVQVIGLYKILLQDLRCLVRQAECICSIRNRRGGVYSIQCCSLADQLDLIAE

RHYFLKDRLDEMSDLFQIQSLSMSLVYFFSTMGSIYFSVCSILYSSTGFGSTYWGLLLIVLSTASFYMDNWLSVNIGFH

IRDQQDELFRVLADRTLFYRELDNRLEAAFENFQLQLASNRHEFYVMGLFKMERGRLIAMLSSVITHTMVLVQWEIQND

ES

>DmGR59d

MADLLKLCLRIAYAYGRLTGVINFKIDLKTGQALVTRGATLISVSTHLLIFALLLYQTMRKSVVNVMWKYANSLHEYVF

LVIAGFRVVCVFLELVSRWSQRRTFVRLFNSFRRLYQRNPDIIQYCRRSIVSKFFCVTMTETLHIIVTLAMMRNRLSIA

LALRIWAVLSLTAIINVIITQYYVATACVRGRYALLNKDLQAIVTESQSLVPNGGGVFVTKCCYLADRLERIAKSQSDL

QELVENLSTAYEGEVVCLVITYYLNMLGTSYLLFSISKYGNFGNNLLVIITLCGIVYFVFYVVDCWINAFNVFYLLDAH

DKMVKLLNKRTLFQPGLDHRLEMVFENFALNLVRNPLKLHMYGLFEFGRGTSFAVFNSLLTHSLLLIQYDVQNF

>DmGR59e

MDSSYWENLLLTINRFLGVYPSGRVGVLRWLHTLWSLFLLMYIWTGSIVKCLEFTVEIPTIEKLLYLMEFPGNMATIAI

LVYYAVLNRPLAHGAELQIERIITGLKGKAKRLVYKRHGQRTLHLMATTLVFHGLCVLVDVVNYDFEFWTTWSSNSVYN

LPGLMMSLGVLQYAQPVHFLWLVMDQMRMCLKELKLLQRPPQGSTKLDACYESAFAVLVDAGGGSALMIEEMRYTCNLI

EQVHSQFLLRFGLYLVLNLLNSLVSICVELYLIFNFFETPLWEESVLLVYRLLWLAMHGGRIWFILSVNEQILEQKCNL

CQLLNELEVCSSRLQRTINRFLLQLQRSIDQPLEACGIVTLDTRSLGGFIGVLMAIVIFLIQIGLGNKSLMGVALNRSN

WVYV

>DmGR59f

MRSSATKGAKLKNSPRERLSSFNPQYAERYKELYRTLFWLLLISVLANTAPITILPGCPNRFYRLVHLSWMILWYGLFV

LGSYWEFVLVTTQRVSLDRYLNAIESAIYVVHIFSIMLLTWQCRNWAPKLMTNIVTSDLNRAYTIDCNRTKRFIRLQLF

LVGIFACLAIFFNIWTHKFVVYRSILSINSYVMPNIISSISFAQYYLLLQGIAWRQRRLTEGLERELTHLHSPRISEVQ

KIRMHHANLIDFTKAVNRTFQYSILLLFVGCFLNFNLVLFLVYQGIENPSMADFTKWVCMLLWLAMHVGKVCSILHFNQ

SIQNEHSTCLTLLSRVSYARKDIQDTITHFIIQMRTNVRQHVVCGVINLDLKFLTTLLVASADFFIFLLQYDVTYEALS

KSVQGNVTRYK

>DmGR61a

MSRTSDDIRKHLKVRRQKQRAILAMRWRCAQGGLEFEQLDTFYGAIRPYLCVAQFFGIMPLSNIRSRDPQDVKFKVRSI

GLAVTGLFLLLGGMKTLVGANILFTEGLNAKNIVGLVFLIVGMVNWLNFVGFARSWSHIMLPWSSVDILMLFPPYKRGK

RSLRSKVNVLALSVVVLAVGDHMLYYASGYCSYSMHILQCHTNHSRITFGLYLEKEFSDIMFIMPFNIFSMCYGFWLNG

AFTFLWNFMDIFIVMTSIGLAQRFQQFAARVGALEGRHVPEALWYDIRRDHIRLCELASLVEASMSNIVFVSCANNVYV

ICNQALAIFTKLRHPINYVYFWYSLIFLLARTSLVFMTASKIHDASLLPLRSLYLVPSDGWTQEVQRFADQLTSEFVGL

SGYRLFCLTRKSLFGMLATLVTYELMLLQIDAKSHKGLRCA

>DmGR63a

MRPSGEKVVKGHGQGNSGHSLSGMANYYRRKKGDAVFLNAKPLNSANAQAYLYGVRKYSIGLAERLDADYEAPPLDRKK

SSDSTASNNPEFKPSVFYRNIDPINWFLRIIGVLPIVRHGPARAKFEMNSASFIYSVVFFVLLACYVGYVANNRIHIVR

SLSGPFEEAVIAYLFLVNILPIMIIPILWYEARKIAKLFNDWDDFEVLYYQISGHSLPLKLRQKAVYIAIVLPILSVLS

VVITHVTMSDLNINQVVPYCILDNLTAMLGAWWFLICEAMSITAHLLAERFQKALKHIGPAAMVADYRVLWLRLSKLTR

DTGNALCYTFVFMSLYLFFIITLSIYGLMSQLSEGFGIKDIGLTITALWNIGLLFYICDEAHYASVNVRTNFQKKLLMV

ELNWMNSDAQTEINMFLRATEMNPSTINCGGFFDVNRTLFKGLLTTMVTYLVVLLQFQISIPTDKGDSEGANNITVVDF

VMDSLDNDMSLMGASTLSTTTVGTTLPPPIMKLKGRKG

>DmGR64a

MKGPNLNFRKTPSKDNGVKQVESLARPETPPPKFVEDSNLEFNVLASEKLPNYTNLDLFHRAVFPFMFLAQCVAIMPLV

GIRESNPRRVRFAYKSIPMFVTLIFMIATSILFLSMFTHLLKIGITAKNFVGLVFFGCVLSAYVVFIRLAKKWPAVVRI

WTRTEIPFTKPPYEIPKRNLSRRVQLAALAIIGLSLGEHALYQVSAILSYTRRIQMCANITTVPSFNNYMQTNYDYVFQ

LLPYSPIIAVLILLINGACTFVWNYMDLFIMMISKGLSYRFEQITTRIRKLEHEEVCESVFIQIREHYVKMCELLEFVD

SAMSSLILLSCVNNLYFVCYQLLNVFNKLRWPINYIYFWYSLLYLIGRTAFVFLTAADINEESKRGLGVLRRVSSRSWC

VEVERLIFQMTTQTVALSGKKFYFLTRRLLFGMAGTIVTYELVLLQFDEPNRRKGLQPLCA

>DmGR64b

MPQGETFHRAVSNVLFISQIYGLLPVSNVRALDVADIRFRWCSPRILYSLLIGILNLSEFGAVINYVIKVTINFHTSST

LSLYIVCLLEHLFFWRLAIQWPRIMRTWHGVEQLFLRVPYRFYGEYRIKRRIYIVFTIVMSSALVEHCLLLGNSFHLSN

MERTQCKINVTYFESIYKWERPHLYMILPYHFWMLPILEWVNQTIAYPRSFTDCFIMCIGIGLAARFHQLYRRIAAVHR

KVMPAVFWTEVREHYLALKRLVHLLDAAIAPLVLLAFGNNMSFICFQLFNSFKNIGVDFLVMLAFWYSLGFAVVRTLLT

IFVASSINDYERKIVTALRDVPSRAWSIEVQRFSEQLGNDTTALSGSGFFYLTRSLVLAMGTTIITYELMISDVINQGS

IRQKTQYCREY

>DmGR64c

MQQSGQKGTRNTLQHAIGPVLVIAQFFGVLPVAGVWPSCRPERVRFRWISLSLLAALILFVFSIVDCALSSKVVFDHGL

KIYTIGSLSFSVICIFCFGVFLLLSRRWPYIIRRTAECEQIFLEPEYDCSYGRGYSSRLRLWGVCMLVAALCEHSTYVG

SALYNNHLAIVECKLDANFWQNYFQRERQQLFLIMHFTAWWIPFIEWTTLSMTFVWNFVDIFLILICRGMQMRFQQMHW

RIRQHVRQQMPNEFWQRIRCDLLDLSDLLGIYDKELSGLIVLSCAHNMYFVCVQIYHSFQSKGNYADELYFWFCLSYVI

IRVLNMMFAASSIPQEAKEISYTLYEIPTEFWCVELRRLNEIFLSDHFALSGKGYFLLTRRLIFAMAATLMVYELVLIN

QMAGSEVQKSFCEGGVGSSKSIFS

>DmGR64d

MLRSHLSVHGLQMERSVQENTLHYTIGHVLIIARIFGVLPLAGINPNGKPENVRFRWFSPYILFFVVAFTFVIADFMLS

TKIVLNDGLQLYTMGSLSFSVICIFCFGSFIKLSRRWPHIIRETALCERIFLKPCYANQEGLNFTRFLRRWALILLVAA

LCEHLTYVGSAAWSNYVQIRDCNLKVGFVENYFLRERQELFSVFEYRAWMVFFIEWNTMAMTFVWNFGDIFLFLMCRGL

KIRFQQLHWRIRQNLGKPMAKEFWQEIRSDFLDLDSLLKLYDKELSGLILVCCAHNMYFICVQVYHSFQVKGAFMDELY

FWFCLLYVISRLMNMMLAASSIPQEIKDISNTLYEVRSSPWCDELGRLSEMLRNETFALSGMGYFYVTRRLIFAMAGAL

MGYELVLFRQMQGAVVQKSICSRGPGSSMSIFFS

>DmGR64eNEW

MARTTGDPAKRRRCMSRIKFWRRSRVGSEATLGIIKYRVVEKDTKRFKLSLIKAWLLRIRQEDYKYSGSFQEAIKPVLI

IAQIFALMPVRKVSSKFAEDLTFTWFSVRSYYALVTILFFGVSSGYMVAFVTSVSFNFDSVETLVFYLSIFLISLSFFQ

LARKWPEIAQSWQLVEAKLPPLKLPKERRSLAQHINMITIVATTCSLVEHIMSMLSMGYYVNSCPRWPDRPIDSFLYLS

FSSVFYFVDYTRFLGIVGKVVNVLSTFAWNFNDIFVMAVSVALAARFRQLNDYMMREARLPTTVDYWMQCRINFRNLCK

LCEEVDDAISTITLLCFSNNLYFICGKILKSMQAKPSIWHALYFWFSLVYLLGRTLILSLYSSSINDESKRPLVIFRLV

PREYWCDELKRFSEEVQMDNVALTGMKFFRLTRGVVISVAGTIVTYELILLQFNGEEKVPGCFEN

>DmGR64fNEW

MKILPKLERKLRRLKKRVTRTSLFRKLDLVHERARKKAFQESCETYKNQIENEYEIRNSLPKLSRSDKEAFLSDGSFHQ

AVGRVLLVAEFFAMMPVKGVTGKHPSDLSFSWRNIRTCFSLLFIASSLANFGLSLFKVLNNPISFNSIKPIIFRGSVLL

VLIVALNLARQWPQLMMYWHTVEKDLPQYKTQLTKWKMGHTISMVMLLGMMLSFAEHILSMVSAINYASFCNRTADPIQ

NYFLRTNDEIFFVTSYSTTLALWGKFQNVFSTFIWNYMDLFVMIVSIGLASKFRQLNDDLRNFKGMNMAPSYWSERRIQ

YRNICILCDKMDDAISLITMVSFSNNLYFICVQLLRSLNTMPSVAHAVYFYFSLIFLIGRTLAVSLYSSSVHDESRLTL

RYLRCVPKESWCPEVKRFTEEVISDEVALTGMKFFHLTRKLVLSVAGTIVTYELVLIQFHEDNDLWDCDQSYYS

>DmGR66aNEW

MDNMAQAEDAVQPLLQQFQQLFFISKIAGILPQDLEKFRSRNLLEKSRNGMIYMLSTLILYVVLYNILIYSFGEEDRSL

KASQSTLTFVIGLFLTYIGLIMMVSDQLTALRNQGRIGELYERIRLVDERLYKEGCVMDNSTIGRRIRIMLIMTVIFEL

SILVSTYVKLVDYSQWMSLLWIVSAIPTFINTLDKIWFAVSLYALKERFEAINATLEELVDTHEKHKLWLRGNQEVPPP

LDSSQPPQYDSNLEYLYKELGGMDIGSIGKSSVSGSGKNKVAPVAHSMNSFGEAIDAASRKPPPPPLATNMVHESELGN

AAKVEEKLNNLCQVHDEICEIGKALNELWSYPILSLMAYGFLIFTAQLYFLYCATQYQSIPSLFRSAKNPFITVIVLSY

TSGKCVYLIYLSWKTSQASKRTGISLHKCGVVADDNLLYEIVNHLSLKLLNHSVDFSACGFFTLDMETLYGVSGGITSY

LIILIQFNLAAQQAKEAIQTFNSLNDTAGLVGAATDMDNISSTLRDFVTTTMTPAV

>DmGR68a

MKIYQDIYPISKPSQIFAILPFYSGDVDDGFRFGGLGRWYGRLVALIILIGSLTLGEDVLFASKEYRLVASAQGDTEEI

NRTIETLLCIISYTMVVLSSVQNASRHFRTLHDIAKIDEYLLANGFRETYSCRNLTILVTSAAGGVLAVAFYYIHYRSG

IGAKRQIILLLIYFLQLLYSTLLALYLRTLMMNLAQRIGFLNQKLDTFNLQDCGHMENWRELSNLIEVLCKFRYITENI

NCVAGVSLLFYFGFSFYTVTNQSYLAFATLTAGSLSSKTEVADTIGLSCIWVLAETITMIVICSACDGLASEVNGTAQI

LARIYGKSKQFQNLIDKFLTKSIKQDLQFTAYGFFSIDNSTLFKIFSAVTTYLVILIQFKQLEDSKVEDISQA

>DmGR77a

MPLPLGDPLALAVSPQLGYIRITAMPRWLQLPGMSALGILYSLTRVFGLMATANWSPRGIKRVRQSLYLRIHGCVMLIF

VGCFSPFAFWCIFQRMAFLRQNRILLMIGFNRYVLLLVCAFMTLWIHCFKQAEIIGCLNRLLKCRRRLRRLMHTRKLKD

SMDCLATKGHLLEVVVLLSSYLLSMAQPIQILKDDPEVRRNFMYACSLVFVSVCQAILQLSLGMYTMAILFLGHLVRHS

NLLLAKILADAEHIFESSQKAGFWPNRQELYKGQQKWLALELWRLLHVHHQLLKLHRSICSLCAVQAVCFLGFVPLECT

IHLFFTYFMKYSKFILRKYGRSFPLNYFAIAFLVGLFTNLLLVILPTYYSERRFNCTREIIKGGGLAFPSRITVKQLRH

TMHFYGLYLKNVEHVFAVSACGLFKLNNAILFCIVGAILEYLMILIQFDKVLNK

>DmGR85a

MYSLIEAQLLGGKLVNRVMASLRRIIQRSLGYFCALNGILDFNTDIGTGNLRRYRVLFMYRLLHNFAVISLTLKFLFDF

TDHFKYIESSTLITVNFFTYFTLVFFALLSSMGSCYQWQNRILAVLKELKHQRDLSRHMGYRVPRSKQNSIDYLLFALT

VLLILRLSIHLATFTLSARMGFNHPCNCFLPECMIFSMNYLLFAILAEITRCWWSLQSGLKMVLLNRQLSTVAFNLWEI

ERLHTRFQCLIDLTSEVCSIFRYVTLAYMARNLWSGIVAGYLLVRFVIGNGLQDVELVYLVFSFITCIQPLMLSLLVNS

MTSTTGSLVEVTRDILKISHKKSVNLERSIEWLSLQLTWQHTHVTIFGVFRINRSLAFRSASLILVHVLYMVQSDYISI

TN

>DmGR89a

MLRFPHVCGLCLLLKYWQILALAPFRTSEPMVARCQRWMTLIAVFRWLLLTSMAPFVLWKSAAMYEATNVRHSMVFKTI

ALATMTGDVCISLALLGNHLWNRRELANLVNDLARLHRRRRLSWWSTLFLWLKLLLSLYDLLCSVPFLKGAGGRLPWSQ

LVAYGVQLYFQHVASVYGNGIFGGILLMLECYNQLEREEPTNLARLLQKEYSWLRLIQRFVKLFQLGIFLLVLGSFVNI

MVNIYAFMSYYVSLHGVPLTISNNCLVLAIQLYAVILAAHLCQVRSAKLRKKCLQLEYVPEGLTQEQAMASTPFPVLTP

TGNVKFRILGVFILDNSFWLFLVSYAMNFIVVILQTSFEHINHGEI

>DmGR92a

MFEFLHQMSAPKLSTSILRYIFRYAQFIGVIFFCLHTRKDDKTVFIRNWLKWLNVTHRIITFTRFFWVYIASISIKTNR

VLQVLHGMRLVLSIPNVAVILCYHIFRGPEIIDLINQFLRLFRQVSDLFKTKTPGFGGRRELILILLNLISFAHEQTYL

WFTIRKGFSWRFLIDWWCDFYLVSATNIFIHINSIGYLSLGVLYSELNKYVYTNLRIQLQKLNTSGSKQKIRRVQNRLE

KCISLYREIYHTSIMFHKLFVPLLFLALIYKVLLIALIGFNVAVEFYLNSFIFWILLGKHVLDLFLVTVSVEGAVNQFL

NIGMQFGNVGDLSKFQTTLDTLFLHLRLGHFRVSILGLFDVTQMQYLQFLSALLSGLAFIAQYRMQVGNG

>DmGR93a

MFSSSSAMTGKRAESWSRLLLLWLYRCARGLLVLSSSLDRDKLQLKATKQGSRNRFLHILWRCIVVMIYAGLWPMLTSA

VIGKRLESYADVLALAQSMSVSILAVISFVIQARGENQFREVLNRYLALYQRICLTTRLRHLFPTKFVVFFLLKLFFTL

CGCFHEIIPLFENSHFDDISQMVGTGFGIYMWLGTLCVLDACFLGFLVSGILYEHMANNIIAMLKRMEPIESQDERYRM

TKYRRMQLLCDFADELDECAAIYSELYHVTNSFRRILQWQILFYIYLNFINICLMLYQYILHFLNDDEVVFVSIVMAFV

KLANLVLLMMCADYTVRQSEVPKKLPLDIVCSDMDERWDKSVETFLGQLQTQRLEIKVLGFFHLNNEFILLILSAIISY

LFILIQFGITGGFEASEDIKNRFD

>DmGR93b

MVYGFTMSGLLVMPRILRCLNVSRISAILLRSCFLYGTFFGVITFRIERKDSQLVAINRRGYLWICLVIRLLASCFYGY

SYDAWSGQYEDMYLRAFFGFRLIGCLICSVIILVMQFWFGEELINLVNRFLQLFRRMQSLTNSPKNRFGDRAEFLLMFS

KVFSLLFVFMAFRLMLSPWFLLTLVCDLYTSVGTGMITHLCFVGYLSIGVLYRDLNNYVDCQLRAQLRSLNGENNSFRN

NPQPTRQAISNLDKCLYLYDEIHQVSRSFQQLFDLPLFLSLAQSLLAMSMVSYHAILRRQYSFNLWGLVIKLLIDVVLL

TMSVHSAVNGSRLIRRLSFENFYVTDSQSYHQKLELFLGRLQHQELRVFPLGLFEVSNELTLFFLSAMVTYLVFLVQYG

MQSQQI

>DmGR93c

MIERLKKVSLPALSAFILFCSCHYGRILGVICFDIGQRTSDDSLVVRNRHQFKWFCLSCRLISVTAVCCFCAPYVADIE

DPYERLLQCFRLSASLICGICIIVVQVCYEKELLRMIISFLRLFRRVRRLSSLKRIGFGGKREFFLLLFKFICLVYELY

SEICQLWHLPDSLSLFATLCEIFLEIGSLMIIHIGFVGYLSVAALYSEVNSFARIELRRQLRSLERPVGGPVGRKQLRI

VEYRVDECISVYDEIERVGRTFHRLLELPVLIILLGKIFATTILSYEVIIRPELYARKIGMWGLVVKSFADVILLTLAV

HEAVSSSRMMRRLSLENFPITDHKAWHMKWEMFLSRLNFFEFRVRPLGLFEVSNEVILLFLSSMITYFTYVVQYGIQTN

RL

>DmGR93d

MKATKYSVGILRFMSFYARFLSLVCFRLRKQKDNNVWLEEIWSNRSRWKWISVTLRIVPLCIYAFTYAEWISNRMLITE

KFLHSCSLVVSIPCYLSIIHLKICHGPEVTKLVNQYLHIFRLGTLDIRRRSQFGGGRELFLLILSVCCQIHEYVFILVI

ASRLCGFQHIIWWVSYTYVFIICNSIMCFGFIWHLSLGVLYAELNDNLRFESGFQTAFLRKQQRIRVQKSMALFKEISS

VVTSLQDIFNVHLFLSALLTLLQVLVVWYKMIIDLGFSDFRIWSFSLKNLIQTLLPVLAIQEAANQFKQTRERALDIFL

VGKSKHWMKSVEIFVTHLNLSEFRVNLLGLFNVSNELFLIIVSAMFCYLVFVTQCVIVYRRRYVI

>DmGR94a

MDFTSDYAHRRMVKFLTIILIGFMTVFGLLANRYRAGRRERFRFSKANLAFASLWAIAFSLVYGRQIYKEYQEGQINLK

DATTLYSYMNITVAVINYVSQMIISDHVAKVLSKVPFFDTLKEFRLDSRSLYISIVLALVKTVAFPLTIEVAFILQQRR

QHPEMSLIWTLYRLFPLIISNFLNNCYFGAMVVVKEILYALNRRLEAQLQEVNLLQRKDQLKLYTKYYRMQRFCALADE

LDQLAYRYRLIYVHSGKYLTPMSLSMILSLICHLLGITVGFYSLYYAIADTLIMGKPYDGLGSLINLVFLSISLAEITL

LTHLCNHLLVATRRSAVILQEMNLQHADSRYRQAVHGFTLLVTVTKYQIKPLGLYELDMRLISNVFSAVASFLLILVQA

DLSQRFKMQ

>DmGR97a

MRFLRRQTRRLRSIWQRSLPVRFRRGKLHTQLVTICLYATVFLNILYGVYLGRFSFRRKKFVFSKGLTIYSLFVATFFA

LFYIWNIYNEISTGQINLRDTIGIYCYMNVCVCLFNYVTQWEKTLQIIRFQNSVPLFKVLDSLDISAMIVWRAFIYGLL

KIVFCPLITYITLILYHRRSISESQWTSVTTTKTMLPLIVSNQINNCFFGGLVLANLIFAAVNRKLHGIVKEANMLQSP

VQMNLHKPYYRMRRFCELADLLDELARKYGFTASRSKNYLRFTDWSMVLSMLMNLLGITMGCYNQYLAIADHYINEEPF

DLFLAIVLVVFLAVPFLELVMVARISNQTLVETRRTGELLQRFDLQHADARFKQVVNAFWLQVVTINYKLMPLGLLELN

TSLVNKVFSSAIGSLLILIQSDLTLRFSLK

>DmGR98a

MEQMSGELHAASLLYMRRLMKCLGMLPFGQNLFSKGFCYVLLFVSLGFSSYWRFSFDYEFDYDFLNDRFSSTIDLSNFV

ALVLGHAIIVLELLWGNCSKDVDRQLQAIHSQIKLQLGTSNSTDRVRRYCNWIYGSLIIRWLIFIVVTIYSNRALTINA

TYSELVFLARFSEFTLYCAVILFIYQELIVGGSNVLDELYRTRYEMWSIRRLSLQKLAKLQAIHNSLWQAIRCLECYFQ

LSLITLLMKFFIDTSALPYWLYLSRVEHTRVAVQHYVATVECIKLLEIVVPCYLCTRCDAMQRKFLSMFYTVTTDRRSS

QLNAALRSLNLQLSQEKYKFSAGGMVDINTEMLGKFFFGMISYIVICIQFSINFRAKKMSNEQMSQNITSTSAPI

>DmGR98b

MVAQKSRLLARAFPYLDIFSVFALTPPPQSFGHTPHRRLRWYLMTGYVFYATAILATVFIVSYFNIIAIDEEVLEYNVS

DFTRVMGNIQKSLYSIMAIANHLNMLINYRRLGGIYKDIADLEMDMDEASQCFGGQRQRFSFRFRMALCVGVWMILMVG

SMPRLTMTAMGPFVSTLLKILTEFVMIMQQLKSLEYCVFVLIIYELVLRLRRTLSQLQEEFQDCEQQDMLQALCVALKR

NQLLLGRIWRLEGDVGSYFTPTMLLLFLYNGLTILHMVNWAYINKFLYDSCCQYERFLVCSTLLVNLLLPCLLSQRCIN

AYNCFPRILHKIRCTSADPNFAMLTRGLREYSLQMEHLKLRFTCGGLFDINLKYFGGLLVTIFGYIIILIQFKVQAIAA

NRYKKVVN

>DmGR98c

MEMEAKRSRLLTTARPYLQVLSLFGLTPPAEFFTRTLRKRRRFCWMAGYSLYLIAILLMVFYEFHANIVSLHLEIYKFH

VEDFSKVMGRTQKFLIVAIATCNQLNILLNYGRLGLIYDEIANLDLGIDKSSKNFCGKSHWWSFRLRLTLSIGLWMVII

IGVIPRLTLGRAGPFFHWVNQVLTQIILIMLQLKGPEYCLFVLLVYELILRTRHVLEQLKDDLEDFDCGARIQELCVTL

KQNQLLIGRIWRLVDEIGAYFRWSMTLLFLYNGLTILHVVNWAIIRSIDPNDCCQLNRLGSITFLSFNLLLTCFFSECC

VKTYNSISYILHQIGCLPTAEEFQMLKMGLKEYILQMQHLKLLFTCGGLFDINIKLFGGMLVTLCGYVIIIVQFKIQDF

ALIGYRQNTSDTS

>DmGR98d

MEANRSRLLAAARPYIQIYSIFGLTPPIQFFTRTLHKRRRGIVILGYACYLISISLMVIYECYANIVALQKDIHKFHAE

DSSKVMGNTQKVLVVAMFVWNQLNILLNFRRLARIYDDIADLEIDLNNASSGFVGQRHWWRFRFRLALSVGLWIVLLVG

LTPRFTLVALGPYLHWTNKVLTEIILIMLQLKCTEYCVFVLLIYELILRGRHILQQISVELEGNQSRDSVQELCVALKR

NQLLAGRIWGLVNEVSLYFTLSLTLLFLYNELTILQIVNWALIKSVNPNECCQYRRVGTCLLLSINIFLSCLYSEFCIQ

TYNSISRVLHQMYCLSAAEDYLILKMGLREYSLQMEHLKLIFTCGGLFDINLKFFGGMVVTLFGYIIILVQFKIQFFAQ

SNFMQNINSTELKAYTA

>BmGR1N

DIYGPEITDKDDGALLDKHDSFYLNTKSLLVLFQIMGVMPIMRVPKSAQTTRRTTYNWISKATLWAYLVWGLECIIVVKVGQERLANFQIGSNKRFDEVIYNIIFLSILIPHFLLPIASWRHGPQVAIFKNMWTHYQLKYLKITGKPIVFPNLYILTWGLCIFSWVLSFAVVLSQHYLQDDFELWHSFAYYHIIAMLDGFCSLWYINCNAFGTASRGLAINLHKALEAEHPALKLAQYRHLWVDLSHMMQQLGRAYSNMYGIYCMVIFFTTTISLYGALSEILEHGLSYKEMGLFVIVAYCMTLLFIICNEAYHASRKVGHEFQDRLLNVNLGAIDRSTQREVEMFLVAIAKNPPIMNLDGFTNINRELFTANISFMSTYLIVLMQFKLTLLRQGARKTVTAIVRAIFNTTITDNGAGGSDEDQE

>BmGR2NJ

KEQEQRDLLSSQDGDTCEIHDQFYRDHKLLLVLFRALAVMPITRSRPGTITFSWKSTATIYAVCFYIAATAVVLIVGYERIQILQSIKRFDDYIYAILFIVFLVPHFWIPFVGWGVAHQVAIYKTNWGKFQVRYYRVTGENLKFPNLKTLIVIISVGCLLLAVCFLLSLCALLDGFLLKHTSAYYHIITMINMNCALWYINCKAIKIASQSLSECFQRDVDIECSAQLIARYRYLWLNLSELLQSLGNAYARTYSTYCLFMFANITIAVYGALSEIVDHGFGFTFKEVGLFVDAAYCSTLLFVFADCSHKSTLKVAAGVQDTLLSIDVLAVDRPTQKEIDHFIQAIEMNPAFVSLKGYAHVNRELLTSAISMITIYLIVLLQFKISLPKEPHGTGQ

>BmGR3F

MSFEIKNNFFRTSVPIPNGFPVQTEAKSKNKPIFLDVSPAPTPKVNSPNAIIPMKNNLIDPFINKDIIYENIKPVFMVLRIMGVLPLTRTTSGVNEFHFISPAMVYSLTVFIILVSYISYLSLHKVQIVRNSEGKFEEAVIEYLFTVYLFPLTVVPILWYETRKIANVLNGWVQFEVTYKQLSNRILPVKLYKKSLLIAIIIPILSTTSVIVTHVTMVHFKTSQIIPYVFLEILTYMLGGYWYLLCEILSLCANVLADDFQQALRHVGPAGKVAKYRALWLRLSKLARNTGVANCYTFTFVNLYLFLIITLSIYGLLSKISEGFGTKDIGLALTALCSVFLLFFICDEAHYASHNVRTNFQKKLLMVELSWMNTDAQTEVNMFLRATEMNPSQISLGGFFDVNRTLFKSLLATMVTYLVVLLQFQISIPDATQPEIPTNIDDHVQNITDTTTEASSPISTLMSAFAKRKND

>BmGR4S

MDKDKFQEFLPTMSRIFSMTRYFGVSTCKPSIAFGWTVILLLMLLAIEVGAIWKIVRLLGGWAVHSTDSRGFTARLSGCIFYGNALLSLILSIKFVSSWEQLSERWSRTETDPGLRLPSDSRIKRRTVLVSAFVMTCACVEHMLSMMSATGFDCPPEEYTERYILSSHGFLVQNDEYNLWLAIPIFIMSKLATALWNFQDLIIILISMGFTSRYNRLNTYVHRVVMLERNLKEGAQVSSENYMRFQIWRRIRQAYVRQAALVRLVDDQLGALVLLSNVNNLYFICLQLFLGINSKDRGSFINRLYYFISLGWLMFRACGVVLAAADVYIHSKKALISLYLCPELAYNLEIKRLKYQLKNDEVALTGMGLFSLNRELLLEVAAAVLKYELVLVQYDK

>BmGR5NJF

YFAFYLSTGCNTFIFLRVASKWPTLIKHVYETQLDSYIDVKVKNKCFAAYIIFFSMSMTEHMLSLLSKFVITMDCLPKGSDLFESYIIRNFPWLFEFDVPYYLPIGVILQFLTLVSTINWSYSDLFIVCMSIYLTSILKQINKKIEMAGNSNHLPIPFWRTLREDYTRATRLVRSFDDTISSVIFLSFASNLFFICLQLYNILSNGVTSKYNLLKEMCPNYPSGPLGGYEQIMYLLFSLSFLLGRSLVVSLVAAKVHSASMVPASALYNIPRNMYCSEIQRFLDQVHGDKVALSGLRFFYVTRSLVLSVAGTIVTYELVLLQFSNED

>BmGR6P

MLLRNYKQNLSFWTSAKKSKIHKIQSQETVTFQGSLKLVLFIGQLFSLFPVCGLLSNDANKVXFVPISWKCGYSMLSMIGQLFIIVMCILYVAHFETTLNGTTPIIFYGVTFISMIAFIRASRRWPELIQHISKSEELDPSFDFRLKKKCNITLLLVLVLAILEHIFSIRSAYSASQICYPHTGFYEGFVRYLYPWVFDFLPYSEELGMVTQFLNIQSHFIWNFTDLFVICMSYYLTSRLDLVNKKLLPAQGKYLPEIFWRTTRETYCRATKLVRKVDEIINGILFISFANNLFFVCVQLFNTFDDSVDMVGLCYNYSERRTKPVGREPVIYLLFSLGFLISRSITVSLIASQVNLASTVPAPILYDVPSAVYCVEVQRFLEQVNGDNVALTGLQFFSVTRGLLLSVAGTIVTYELVMVQFNQAPASDSFTEKLVENNISTIETFYNYS

>BmGR7

MVLEAHTQIQYCTAKANYCEFHAGLRHLMRLARWAGFFPVQGLSQTNPDDVRFEFRSLYALYHAITVIGQTVMTFLAFYSFVDSNVSLSVVSNFLFYFTNYVTLVLLWRLSKNWSALISKTLEFEQSVTEIRTTRNLVSRTNTLTYVVLIFAMVEHALSKVFNIRSVMCCLGETSLNHTVINNYFKFKWKFVFDYFSTSTTYSYFVGFIAEFLCMQATFLWSFTDVLIMCFSIYLSSFFEDFNSTVSSFMKKASKTVPWSTLRVQYSQIVLIVKQMDEQLDYFVLISYFTNLFFICFQLYNSLNRIYDANDVCNENMDIIATASVTYLTYYVFSFLFLVTRALLLSIMAANVHSCAQVPQLALYEVPTADYSLDVQRFQLQLRYTTVGLSGVCFNVTRGMILRVIGTIVTYELVLIQLTKKNLDNDTSIRDYYLPKHLI

>BmGR8R

MAPRSVRSMVGTSKKDMLKGGFYETVRIPLYIYRLIGILPISGLWHRSSKYNRFSLKSFYTIIYAPTIVMQTFLLLVHIYDLFAFFFGHQRLGRLIYHMNFYTITILIFMGSRKWKNVIKEIETIELTLPRLRNSKKALALTKSFVFAFFVFSLAEVVLILQFTLRLTKQRHVLPGDSGLYLRSYFVYIFPYLYDHFPFSYVMGFIVQIIKVQGIITLNMVNCSVVILSIYLTNRLKHYNRIVFAKGSKTNNTRLKWVELNLLYTRISNLVKIIDKNLNPFVFISFTANLSYICAQLFYILNKLTSSRTVKITSFLEDKRCDWETVLYISISFALVVLKVLLVSIIAAEVHTTSREPLRLLYTLPTAEYTIETQRLMTQVYYSNLSLSGLNFFHITRGMLLGMVATLLTYEIVLLQI

>BmGR9R

MPPSPDLRADEPKTPCLVGGAHAFILKISSFCGLAPLRFEPRSQEYAVTISKGKCFYSYILVTFLVICTIYGLVAEIGVGVEKSVRMSSRMSQVVSACDILVVAVTAGVGVYGAPARMRTMLSYMENIVAVDRELGRHHSAATERKLCALLLLILLSFTILLVDDFCFYAMQAGKTGRQWEIVTNYAGFYFLWYIVMVLELQFAFTALSLRARLKLFNEALNVTASQVCKPVKKPKNSQLSVYATSVRPVSCKRENVIVETIRVRDKDDAFVMMKTADGVPCLQVPPCEAVGRLSRMRCTLCEVTRHIADGYGLPLVIILMSTLLHLIVTPYFLIMEIIVSTHRLHFLVLQFLWCTTHLIRMLVVVEPCHYTIREGKRTEDILCRLMTLAPHGGVLSSRLEVLSRLLMLQNISYSPLGMCTLDRPLMVTVLGAVTTYLVILIQFQRYDS

>BmGR10

MTMSIKPRLQCMVPPSLALALRVSRLAGIAPLKFVAKQSNIMIRLSTSLCVYSYLLVTALNVCTLIAVMIDFSVPVKLSIRMQTETKRFVWIADVVIVGMLSGVGVYTAPIQMRRLIAYLHRIHKINSDLQTYSSSLTDKMLHRLTIGMLLITSVIIVTDFTFVMYLADLNHRQLLIAIMYWCYYCSYFIAHLLEMQFVLIAALALSSLKLVNNGLRTMLHQSGIESLTEIPNSNEQHAANAVLPQPPKKSVNNSIDTLAFVVTKRSVRFPTAGWTDQRTIRRLALSYGSICEVVRQIDNNNGIIVLLLLASFLLHLVVTPYYLIISFVTESPHTGFEKVLNPILQTVWCLYHTFGLVMIIEPCHRTHEEMETTRELVSRVMCSADPRDPISVELEMFFRQLVLNKASYAPLKVCTLTRSLVATILGSITTYLIVIVQLEIKNMQ

>BmGR11F

MKPFRFFLFVENVICVYRNYSFHKRYARAIILSRVMFEVSLIILTLHSCRNFGAVKYKTEIIFTYLATASSTILILLALYKTNRFTELFLNFKAFYRNRNLDVDHLEKWNRKQKMATVIIVLFCVIKFSTLIYTDLIGEYSTPCRGYFTEYLFYTNLFMCNARYLFEFSTACVVLHLVSEQLDYIAISMDCTMFLYIDISKKNIMSSAKKRKLKYFDIFKQFEKWTDAYMNVKRSANLCDTVFRAQLAIMITTITLYYIILLYGITSFNIERGKFSVVKSLSYLISLFGFLIALLLLSKAGQRIQKSAENLRRKLSKFLLHSLEDPEFHRAATNLLRLVCTHHIKMRCFGFIDIDMTLLPSCLMFVTSYTVIALQFNNVV

>BmGR12P

MKNLKLCRTTFYFKIIMCSRFISGLYFTATSKKWISYLYKVICVLYIICITRLFYAKEDTFKPLVFXQFIGNSIESLRTGEGHVLKCYSTIFSLKLIRNYLPDSNNHIPISSITNFLVIIWKVFDQVYIVLMHYFYTTDIIIHLRILSILTTIGVNLSLMPIIVIFELMWRAVKALRKSLGEHLKGPVLIEGRERLKAQQILRCLNVYKDLNATLKFNSTPMKTMILISTLATFIRLTLFLYQAILGHNEGLHLPRKILAIIYYALPVCLLGVLMELVARECDKLKTLMTKELLVCKDDSYCTVIVDAVSYIELNPLKFSILRAFNVNSTLILGLTNLCTTYLIAVIQFTYSCEDINGLSHSHSH

>BmGR13

MEDSFNRLLSIRNMIIFQNVCGFYHMCTEKLYISRIIKMYCVALAIVLSVFCFQNPDITYLSWDVVWVTFGYTLNVIICLRYNGNYFFQYWNGLHEIDIKMNLTSIDKEKVPISRAVFTVFLILRSTAFAMTIFVFGYLETGILSNTIISIYSINLTEFYRNMSNIPMILMFETFYVRIKILKEQLCSELSTVLGCNNDARQLKLILKYLRNYRSLVRHLMDTTLPFKILILVILVGSFLRSLLIGYAFVYNSDQIILLSLPVMFSTKILSEVVEIKLICTKELLKNKNEGLVLLDLDSKKPTFLTSKACGEQLQDALSFLNNRSYSYTLLQVIEFDCSLAFVFTSFCITHLIVVVQFTHVLD

>BmGR14

MNLHKNIIPIRNNLFANKVTAIALPKTLSVLFKLIHIFFLLDLGVYEYKTFKIKCIVKFLTISGSLTISVVCFSFMVSNLSEHTFVGWYGFFISTYIFVVLFFNLSNRMTFVEFYKTLLRFDANYGIDSNEYKFNFKIIFVNILFIANRMVLSFVYCSYYPQNCIRPRYAQILFMLPWLTLDVLLTTNMFLFYATYCRIAKFPMLIKNSMNIVALRNSYKLIVDSLEKTQTSFDIVFIIALVFSVPEIMMSIYSTLLEVISKHFLEVASILSLNYVAIAQSLLLTLAPSLCAGVLPWKTNNIKIILHEKLFTEKDKASAREIELFIKYIESRPLKLRACNLVPLDFSLTIIVLNICVTYLIVIIQFTHLY

>BmGR15

MISSSDINHKRNKVFAYNVPGIALSKTLTVLFKLLHYVLLLDVGIYEYKTFKNKCIVKFLTIATGVSVSIVYFCLIATVLRKNAFFYWFYVLFISQYMIIVFIFTLSNGMSFTDYYKMLLRFDAKYQINSNNYYFNIKIILVIIISILNRIGMAIIYCSYYTKNCYEMSFSQIIFVLPWLTRDVILIMNVFLFYVTYCRITKFPALLENTKNVGSLRNSYKLIVDSLEKTQKPFDFVFTISLVFNIPEIMLSIYFTLLQVIHSHFLEVAPTLSISYFSITHSVVLILAPSLCAGVLPWKTNTIKIVLHDKLFLEKDKNSARNIKLFIKYIEARPLKLRACNLVPLDFSLPVIVLNLCVTYLIVIVQFSHLS

>BmGR16F

MIMNLTTDRISKRNKVFAYNVPEVTLPTTLKVLFKLIQFTLSLDFGVYKYKTFKMKCVAKVLTLAGCLAASAACVSLIISNIFENQLFFGWYTLFVCQYTIVIFMFTFSNGMTFIDYKMMLLRFDAKYQIDSNVYHFNIKIVLVVVISVTSRLFLCAVYCIYSTENCIKPWYNQLLFFPWLSLDIVLIMNMFLFYATYCRLAKFPSLFENPKNVVPLRNSYKLIVDSLEKTKKSFDAVLIAALIFNIPEIMMSIYYTLFQVMNKHFQEVAPVLSLSYFTIILSVLLILAPSLCAGVLPWKTRHMRLILLEKLFAEKDKNSAREIELFIKYIEARPLQLRACNLVPLDFNLPVIVLNLCITYLIVIIQFTHLF

>BmGR17F

MGFSLGTTALSMFFFEKPVVFTIIQITMIIVKPAKYKLSDPFRPKDTSKLSESIIMYFKLFHIFLGIDLGGFRYQNRQVKYAVRLISLIQPLAIYGLCIYALLKIIANTEFLWYTISFTEYVAMSVAITLFSNEMTYCNFMINLKFIDTKLKIGDESFRIGVKLISSTILIGVTRCFTTTTYCLLGFCAKPTAAQILFQIPWLTIDLMLLQYMFIFYACYCRLVKILRILKKRNTDIEEMRRIYKTLVDVLDRARAPFDLAYLLGLLFSIPDVLYSIYESIIKVGEINTAKALSMSIIYITNIQSLALMFAPALTAGFLPSLTMKMRIILHDKLLEEQDKKTYRHIVLFIKYIETCPLKLKACQIIPLDFSFPIIILNIVVTYLIVAIQLTHFL

>BmGR18

MRRSTKVISMVNQSDKGEIKTCSRFMKIYFFVIYILTGFNFGFYTGRGLNFLRVIQASVLLLRFIIASNCIYIAFHFRLLEAIWYSLTFSESLAIVVCFMLSRSALSCKNLFEYLYSVDQELKKSVGPSIEVKLALYTVVVSVLRLTVYVFCAIAYYETLHEGFCVELVYNTPCYCSDLYLVIHFTIFHSVYCRLKALRISMNEKFDVYKGTLIYKSLIDNLEEIKKSLDVPFFVILLNAVAIAMINILVTLEISYGQTMKFIRTAPRYLETVLLFSSAFAPVLAADMMASEAQKIKVTLNNILQRDDSLLEDDRRKVKQFAGYVSARPFRLRACRVLSLDCTLPVTVLSICVTYLIVVVQFTHLY

>BmGR19J

MRRSTKVISLVNQSDKGEIKTCSRFMKIYFFVIYILTGFNFGFYTGCGLNFLRVIQASVLLLRLSVASYSMYIARYSPLLEVIWCCLTASENLAVVVCFMLSRSALSCKNLFEYLYSVDQELKKSVGPSIEVKLALYTVVVSVLRLIIYVFCATAYYRKLFDGLRLELLYHTPCYSLDLYLVVHFTIFHSVYCRLKALRISLNEKFDVYKGTLIYKSLIDNLEEIKKSLDVPLFVILLNAVAIAMINILVTLHISYGKTMKLITAAPRYLETVLLFSAAFAPVLAADMMASEAQKIKVTLNNILQRDDSLLEDDRRKVKQFAGYVSARPFRLRACRVLSLDCTLPVTVLSICVTYLIVVVQFMHLY

>BmGR20J

MRRSTKVISLVKQSDKGEIKTCSRFMKIYFFVIYILTGFNFGFYTGCGLNFLRVIQASVLLLRSIIASYSIYVAIHFRVLEAIWYCLTFSESLMVVVCFMLSRSALSCKSLFEYLYSVDQELKKSVGPSIEVKLVLYTVVVSVLRLTVYVFCAIAYYESLHEGFSVELIYNTPCYCSDLYLVVHFTIFHSVYCRLKALRISMNEKFDVYKGTLIYKSLIDNLEEIKKSLDVPFFVILLNAVAIAMINILVTLEISYGQTMKFIRTASRYLETVLLFSSAFAPVLAADMMASEAQKIKVTLNNILQTDDSLLEDDRRKVKQFAGYVSARPFRLRACRVLSLDCTLPVTVLSICVTYLIVVVQFMHLY

>BmGR21

MAHRTNSINLFRSRPPDIRAGVGEPRIFSKFICGTMFTQKSLVNFDLGKTPRGGDQEHSKFFKIYFLAVHSVTALDFGFDRNAKKLTKILISMFSISVRMGLAAVSFMSLWGRPNALALGWAPGTLLCENILVAVTYSASRSTFKCGDLFADLSTIDELFGSACDYRIESKMLLFTATMTVLRVVIYSTSRLVRADGFDFVDVLEVLNNLETMCMYLFLTVYFFVLFSIYCRFKKLRELMKNDFEIRRANLIYIALKDCTDKIKQSLDVPFLVVLVFTVLVVMVDVFITLEMIISNKYNMAVYVVRYLEITLDFLMLFAPVLLADMMAVQVDGLKITLHDRLCLNNGVDMKRYSSLAEFIGYVEARGCRLRACRVVPLDLTLPVTVFNVCVTYLIVMIQFADLY

>BmGR22J

MTRFEFSQRTKTKSVSKTLNILFNCIYVLTCMDFGFSLGVYKRMKVLENISLVLRVMVAIMCAAMVMKQDILDSAWADITLTESLLVIVSFKLSKPKLSYRELLENLSIVDETQGAPPAGYKVERKLITYIAGVTALRLTVLCLYCVAHTEQYSIDNFIEFLYNVPCYCLDLYLIVHFIIFHSIYCRLRTLRKALSNNFDVYRAHLIYKTLIDCTEEIKKCLDIPLVVILIATILVVMVNVLVTLRMLFKGESIISAFLLRYIEVILSLALLFVPVLVADMMALEAHKIHIFLHGRMQTANDAAEERREVRQFAHYVGTRPFRLRACHVLALDSSLPITVVSVCVTYLIVIVQFTHLY

>BmGR23J

MAQFQIPSMAGSGLNVAPFSRGRRHEGPKHSNFTKKYFLLVHLVTCLDFGFHRDNDTKTYKWFHAANIGVRLVLSAYVCSVSLSQDLSFASAAWTILNNSKHLLVVAIFTIFKPKSSCAEILKDLLMIDEALKIHRGCDVKGQITVCIVLVTAARLLIAAASSLSLHEAFSASVGAAEVLYSFQSYCLDFYILANFFIFYSVYCRLKNLRRVLQNNFNIYRGNMIYKVLVEHMDDIKKFLDIPFVTSLLVTVIMAMINVLKTLQLIHDGENDVLTIVLRYLEMFLSFSLIFAPVILSDLMSIEADNINVVLHNYIYETDAAEERERRHLSQFAWYVSARPFRLRACRVLSLDCTLPVTVLSICVTYLIVVVQFTHLY

>BmGR24P

MCINKKIQSIIKSLVSIRTIMLVQSILGFYHKMSNNFFVSFLFLTYTTILISVLSFYSVNDVMAHKFAYTLSMILEYDINTILSLITAGRQYFNFFEEMKKIDFSIGFGELNIEDLPLSRTLFVTIFVTNILLSIMTAALILFFSTPFLIISSGSTYAMAIVFFGLSLNVLPRIIIFELIYKRIKYINLSLKRKLKALALECDHTIARFEIINENLIIYNKLLQSLGNVNVSLKSSILLTTFTCFFRCSLICYYVITMNEDKVYIMQIIELTKQTLFLGVLIILAEYIKNEIENLKMTVSLQLFTCTDTQLYHQVSDXFEIHRITPIQLCGFKNMSVDTNLFLGLINVCSTYLIIITQFLNAYVN

>BmGR25J

MFVKCLKYVKKFKPMFSVMFIMNFRLICGLYYRIHSDAFVCFVFKVYCILCSMFLFFTSSDLAAPFSRSIPILATLFEYVANVLDCILTGQSYFFHLRMELMRIDPRLRGLDRPPASSIVFTAILSYKIFILAVYIHGKARTTYLQYEWFSSIGIHLLVLFSNLVHMNRMLIFEMVTFSLEAQKKTLGELLKSSLRRERVERKCEILNRFLKTYKRIIELFNNTMAATKLMTLISVVSCFIRILTYLYQVLTTQLSSSSAGSIFTTRHLTFIVTFVREHVLSVYFLKKVGICQRLQINFKIKCNVKTETLELISQDDEYTEKLEDALDFINSCSSKITILRAMTVDATLPLTFISLCTTYIIVVIQFSHIYD

>BmGR26

MNKTKIYRKKLDKNERLVCSVQPAMFARLIVGLYYDIKVSNRVKWMIKSYCISLSSFICYLIIFRDDNFSLHPKLTSVMEYITYVTFSFLTCDKYLFRYLRFNPRTDGYPIFLYLCKKFEKFFKIIICLFVSFKILGVVLMMQSWPILSTPKYIWGTLALHFLWLASHMGRLVFILVYGILFCRMRTIRIIFENRGFQNTPQNRLTPKRYILMYEAVLNSIESVDFPVKFLIFTFICCFAPKLVVSLFEIMEEMKKGELSLTTFIWFLVELSPSYLFLLLSAIALDLVSEDVQELLSITIDRRLNCKNEKERSEIQEFFQYLRNNPFNYTLWQVVSLNLRTLLVATSFSIANVIAIMQIKNSKI

>BmGR27

MVFKYKIMTKAPKSLPVLKILMLFRLVFGNYFRLSSNRYINFLVKSYCSTFTILLSVMCGKRLKNDSPYMLSLTEYILNKILNYATSEGYIFKYCNSIKTCDKIMGFKKLPIITIDVFIAIIITVITRTAITIYFGFLFPFDKYQVVLYVGCIVFSNDLNSLTIMNVFGLLNNRMNLLRKSLEAMTVPINIIGKNEVAPKVRLVRNAFRYYSNLLDNLDSVNHCVQYSLSVTLLLKFPKAVLLCYDSIKTYFVKIDNNFAMDIVDPTEIILSIVVMSFPAMLCEMITNEVEKIKAILTKHLIQCSDNSLRFELNITLLYICHRPFKYILWRAIPLDTSVPIGIVSLIITYVIVLIQLLHFST

>BmGR28P

MAHKIATVGPTNATATVKNNKRKLKISKRVTIFKVVRCLRFILGHYTELTSSKLKAFLIKCCSLLLAVIIIYAPLNYIKMAYVMGLIEYLLFVLLSLFTGDEYFYKFHNSIKSIDVLMGYKRGKIIDSNAIIFLLSVITIMRIVIIYCRSTVLAFRFTIIGVYLAIFSLRISYMLITVIFFAMYHRMKFLRKKFEIITIPVTIIGKQKVASKIRLIRKYLINYHHLLDCLRDINGGLQYFLAIMIACNLPKYIFFAYSAIKIQVLEHITIHSAVQNVKCZRILFVVVPAIFAELTTAEVERIIDVINRQLLRCTDEHMELELKVALEFIRRRPFDYVIWRTVPLNASLPIAIISLCITYVVIVIQLTQFHDNF

>BmGR29

MYLRSKKSRFKLFSFERMIKILLMICGHYVQTDSSNVVSSIHRIFSIVITICLCPYFQFNPFFFHVIESVWYSILSQFTQYGFFFRYCSTIKTFDLLSGFKQIPLYTKRVCFFLLITLLVRLIIVLIHFSAHQTKLKTFCAFLIILSANTGHILMTIMFSILNTRMTLIQKLFANNPIPVNIVGKNQNASHIKRVRKGLICYNNLLDTLKVAEKEIQFTLTVTYLCHVPTIICYVYFVITVIYKSKFSGYNLIPMLDMILACMAVTAPALFAELTKNTVDKIKKILGSQLLRCSDESLRYELEITLEYVIQRPFSFSIWRAVSLDASLPVAMTSLCITYVIVILQLTQLRP

>BmGR30S

MYLRSKKSRFKLFSFERMIKILLMICGHYVQTDSSNVVSSIHRIFSIVITICLCPYFQFNPFFFHVIESVLYSILSQFTQYGFFFRYCSTIKTFDLLSGFKQIPLYTKRVCFFLLITLLVRLIIVLIHFSAHQTKLKTFCAFLIILSANTGHILMTIMFSILNTRMTLIQKLFANNPIPVNIVGKNQNASHIKRVRKGLICYNNLLDTLKVAEKEIQFTLTVTYLCHVPKIICYVYFVITVIYKSKFSGYNLVPLFDMILACMAVTAPAVFAELTKNTVDKIKKILGSQLLRCSDESLRYELEITLEYVIQRPFSFSIWRAVSLDASLPVAMTSLCITYVIVILQLTQLRP

>BmGR31JI

MYLRSKKSRFKLFSFERMIKILLMICGHYVQTDSSNVVSSIHRLFSIAITICLCPNFEFNPFYFHVIESVLYSILSQFTQYGFFFRFCSTIKTFDLLSGFKQIPLYTKRVCFFLLITLFMRLFTVLIHFLAYQSKFVTFCAFIIMLSANTGHILMTIMFSTLHTRMKSIQKLFANNPIPVNIVGKNENASHIKRVRKGLICYNNLLDTLKDAEKEIQFTLTVTCLCHVPKIICYVYFVITVIYKSESLRYELEITLEYVIQRPFSFSIWRAVSLDASLPVAMTSLCITYVIVILQLTQFRP

>BmGR32S

MCYTNFVSRQVSKCINFFSTIRYVIYLRMFCGLYYNCSSSFKIRCIARLYCFIIYCLNLHYNLYIFTSSVSLTNFLHTFITLAEVSIHILFSLYTGESNFMSFCIEMNKLTSGPIDFVATKCVATHFIAFFVIGLHILSSTLICGAEVSCFTFSVVLASMTFLTTLLSRFTTIIMFDLVWIRMRSLRKILVNALESDLSEDEKVKSIESFLKAYKQIIASIRITKLATRNLVTFNFVSLFGKIMTLIYFCINCPGYLNTYLISSWIFGILLAGFVTCAPPVLVEMNVNELDEIKYALADQLVDYTDDNYRTAIYNALDYVEVHSIRYTLWKNFPMDLTMFFGFAGFCATYIIGLLQFTY

>BmGR33F

MCYTNFVSRQVSKCIHFFSTIRYIIYLRMFCGLYYNCSSSFKIRCIARLYCFIIYCLNLHYNSYIFTTNVSLTNFFHTFIILAEVSVHILFSLYTGESNFISFCIEMNKLTSDPNEFIATKCVTTHFIAYLVIVSHILSSTLICGARASCFTFSVILTSMTFLTTLLSRFTTIIMFDVVWIRMRSLRKILVNALESDLAENEKAKSIESFLNAYKQIIASTRITKLATRNLVIFNFVSMFGRIMTLIYFCINNPGYLDTYHMSLWIFGILLAGFVTCAPPVLVEMNVNELDEIKYALADQLVDYTDDNYRTAIYNALDYVEVHSIRYTLWKNFPMDLTMFFGFAGFCATYIIGLLQFTY

>BmGR34J

MCYTNFVSRQVSKCIHFFSTIRYVIYLRMFCGLYYDCSSSFKIRCIARLYCFIIYCLNLHYNLYIFTSGVSLTNFFYSFITFAEVSIHILLPLYTGESSFMSFCIEMNKLTSGPNEFIATKCVATHFIALLVIVSHILSSTLMCGARASCFTFSVILASMMFLTTLLSRFTTIIMFDVVWIRMRSLRKILVNALESDLAEDEKAKSIENFLNAYKKVIASIRITKLATRNLVTFNFVFMFGKIMTLIYFCINNPGYLNTYLISSWIFGILLAGFVTCAPPVLVEMNVNELDEIKYALADQLVDYTDDNYRTAIYNALDYVEVHSIRYTLWKNFPMDLTMFFGFAGFCATYIIGLLQFTY

>BmGR35S

MYSSLKLKDYIVNMESVMCSDQSITISFKNVFKVFFDYISSLDFMMVCRLCFGYYYEFNCSNLCKIMFKCFSISVCIFCVSMHLVQLISPPYLNHCVIMLESTVSIITSLVTEDKYFFEFCLDMKDINSMMNQPRNIKSFKIIYVIISGAICHVIRHISICREKALSFCFSTEYLTASFTIISGYWNYLNITMMFDLLYQRLVAVKQMLTNGLNICDTDEYKIKSVQKFIDVYKALTVSLSKTSNLIKHTVSLGIFCALWRIIFFVYYCISMDFQVESAQFITWVSSMCLSVFLVYIPALIVELCSNEVDAIKWILASELLEYRDKRLRTSLRDALDYIDVCPIDFEIWHCFPMNLSLCLGFIDISSSYIISILQFKY

>BmGR36JP

MTVPYDKIKSALSKFVQLLFSINVVLFVRFLFGFYMKIGSRKYFHIATKIWIVTLTIFRVYFQCRNFMNYPSYALILHDFTCTVELVILCIISSLGGEQHFYTYCSEMAELIDNRKNKRASYFTTSTLLIGFIILIVPTSISCKKVSNALSMLFLNIFNYVACFMHHLTIIYVFELLWREIRKCRISLERLEIMSVDDKIMKIENFLDSYKRSLDSLNKANGVMIPTMALAYFAIIAKIVFFTYNILSLRGFNILVHGDSWLLSTSIAIIFICAPALLVELAANEVNKIQNLLAVELLKMKDDKFZYRATLYDALDYIEVRSVKSMIWRNFPMDLNLCVGFINLIITYVIGLLQLLY

>BmGR37JI

MSAKINYQEKLCSIKSIMYLGLFCGLYFRSSTSRMMLLMTKVYCIVLLFLGISFHLNMLSSDLPTETTLHSSIIMLEFFIHIITSLSTGQAKFLHFCTEMMKINGHNSKGSLDFQLVITNIALIIIIITQTTSSLLYCIIRSKCLSHSYVFTVITSLCVLFSSFTMIIKYELIWNTVRSLKNTLVSNLDSFDLSEQEKVNSVYNFLSTYRDIKANVDLTIKGTRTTDCLHASARVSAIVLSVAVTCIAPILVEINVYEFGRIKFALADQLLEYTDSKLRRSVYDVVEYITVAPPTVTIWPEFTVDLGLYMDFIGLTVCYCIMILQFEY

>BmGR38P

MKPKYVTNIKLVLFLRFLCGYYYEMEIPRRLKTVAKAYCIFFLFFYLVLHHLYCSFSNHTAKWSLYLEYSIYVFMSLYSKKMYLMDYYTSSRIIDFEPHSRIYKKLNIYLAILIPFLVVLKIVNMVTFCLSKSFNCWSWVSLLHNLLWNFTVLGRIPPVFVFALLFCRTRIIRRTLVSITVGPGSTVLKSLFKCTRYWLTVLKRPNTHLNSXLTVFLLCSTPKLILETFLMLNKIKESGPIVEKLAVYAIETFYTHLFFVVSSILFDLINVDLQRIKILIVEKRMKTKNTKHRIEVEKLFQFVKSQTIECTLWRVLSLNVRNILSFVSFAVTTIIAVLQIKNNNIY

>BmGR39SI

MNVSPQYSVLKIFKPLFKVQTLFGSVRVKVGDNGITKTTKLQKFYSIFNILFATTGHFYTSFVYSVCVPCVGNSVAETSMALIQIYAGHLMNSFIVFSNTFLHNEKNVQMFKSLCSIDELMKIVRLEHRDLKLFIAIILLLSSTVIMNVYFLIYMVIILPISEKWVPFANIGIMNEDLEAITFVSVLYMLYDRVKYINQTALDPVNIAKLIKESDGQNDEETVSRILKAFKEISKAYKIVEKTFRVFNLSWGTFMSKNLIGVKFIARIMLISVCVSYLERELQKTKGLCNLAVRNCENDIVRCHLKNIYRIIDTEIEPMTVFGLFYINNVPLDLISLTATYTVVLLQFAFL

>BmGR40SP

MLPICYIKLVYNISSIMFLRLIFGLYFELNVSNRMRWLQRMYCVFISIVIMYFCYFKTYRILMTVKVPFILEYGMHVILSLYLRENFEYTRYDPIIDSAADTKNFYKKFDIFIKIFLFVAILLKMZSMLVFCVLWSGLCYTGFIGICGMNFIWIATFMGRMVLPLIFGILLCRIRMFRLTLQKQGFDNLPYNRFSPRRYIMMYDSIVRGLEKTDFPAKNIMFVFTICIYSKILTGLFDLISVLKREGPKLMNVMLFTLEFLPSYVLLMIYSVTLDMVSTEMKEILKIVTEKRVFYEKQHANIQELCQYIKNNQLKYTIWRLVSLNMQSLLRATSFCIVSTIAILQIKDWNG

>BmGR41SF

MSENSLEEYIHMSFSPIYKYQKFLGSNRISLKAKNKITVANNWEKLYAFLWMLAASYSIHHFISFFYSYYYERSNIIFLACSLGISMHYLTYILTITYDKFLTREADIDLFIDIQKIDRLLKLDRCTVLFKKFRLINIFLLILVTVPFISGFLIHVFDYIDKPYKTFFLGLGVTITYVDVLVTAFFITKLTLRLAYINDRIAMYNKINIPHKKYSGIRRSILWIFGWRIFKIMPKIKKNGTREKKSTFIKYPSIIFNILKCYRSITEIYSLPVFLITATVSIWTFLVIGSLVAGSRSEIKIFPVVAMITVGLWNFYYIIQLTSLAFVNDLFLMEVKNTKQLCISVLLYTCDDSINKAANTILKNIECVPPIFSVYGIFVFDKSIILFLFGIITSNVMTVIQFSY

>BmGR42P

MNKTKKIERLSRDILDEDFIDVFKSIFIFQRIFGLLSVNITYKYITETSKLYKLFVMSLWTVNVLCLVDYILNYRTSFDVATDSMLKLVMSVNVTTNALIVWRNNFKLNTLKSQIYVKLQNLDRDLKTKDAVTMNKKLSALSIALMICGFIWCTIWLFVYNAIAMNTFCVPLTIILSANVGNWLEMVLLFIIFYFVNVRAEYVNKLLRRRLNQTECPDRVFLIQNAKPSDTVSREFICGMQSLLEIIGNIKDIYQFPIFLSTCQVMLCILVIVQNLIISVKEQTSTMVDSMLCMLPALLLMLTIFFSLCVIAEALTSKLDITKKLCAMGMHSFTDDISRRNSXQIVLLLKAKRPLSVFKIYTLGTRLPIHLLGVTASYTIVLLQFAVL

>BmGR43JI

MLKKSAMITMKSPEYLSKDILDEDFVRVFRFPFLVQMVLGSCRVHLKARFITIPTLGQKLYTVMSIIICSLLYFNITKLYISLYYEQSIVYFLFLTVAGLDQLSFFANLIHVRFLNGETNTGFCIMMQRIDRKMKIDHNNIFNKTVIRANILTITLIILLYMSLVISTIILKKYSLVTLFGLVHGQLILLVEMAYCSNLIIFFFIRVRFVNAIIKNHVHPENQNQPPKLVRYFVTNRIMRYLAAQTHDFIVNDTDVYLKQIFEGFSMFIDIYRFQVCLFCIKLIVMSLLTFEFCLVGIQKNGPLRKKATRMLKIIEESTPQFSIYDMWQMDGYTFVKICSLVTNLIVTSLQFAYL

>BmGR44P

IFLSTCQMMLCILVILQNLIISVKQZTSTIVDSMLCMLTALLIMLTIFFSLCVIAEALTSKLDMTKKLCAMSMHSFADDISRRSSKQIVLLLEAKRSMSVFNIYTLGTRLPIHLLGVTASYTIVLLQFAVL

>BmGR45JR

MITMKSPEYLSKDILDEDFVRVFRFPFLVQMALGSCRVHLKARFITIPTLGQKLYTVMSIIICSLLYFNMTKLYLPLYYEHSIVYYIFVTVTGLDQLSFFANLIHLRFLNGETNTAFYIMMQRIDRNMKIDHNNIFNKTVTLANILTITLIILHYVGLVISTIILKEYSLLSLFGLLYGQLMLMVEMALCSNLIIFFFMRVRFVNAILKNHVHPENQNQPPKLVRYFVTNRITRYLAAQTHDFIVNDTDVYLKQIFEGFSMFIDIYRFQVCLFCIKLVVLSLLNFEFCLVGIQRNLLETKNLTNYYIMTYSVIGFFTALYVSGRCELFFREIRETKRLAVAVLLQYQEGPLREKATRMLKIIEESTPQFSIYDMWNMDGYIFIRICSLVTYLIVTLLQFAYL

>BmGR46JR

MITMKSPEYLSKDILDEDFVRVFRFPFLVQMALGSCRVHLKARFITIPTLGQKLYTVMSIIICSLLYFNITKLYLPLYYQHSIVYYLFLAVTGLDQLSFFANLIHVRFLNGETNTAFCIMMQRIDRNMKIDHNNILNKTVIRANIFTITFIILIYVVLVISTIMLNEYSLVTLFGLLYGQLIFMVEMAHCSNLILFFFTRVRFVNAIIKNHVHPENQNQPPKLVRYFVTNRITRYLAAQTHDFIVNDTDVYLKQIFEGFSMFTDIYRFQVCLFCIKIVVLSLLTFELCFVAVQRNLLETKNLTNYYIMTYSVIGFFTALYVSGRCELFFREIRETKRLAVAVLLQYQEGPLREKATRMLKIIEESTPQFSVYDMWNMDGYIFIKICSLVTNLIVTLLQFAYL

>BmGR47JR

MKIRFLFGFYCDFPFNKRFQNILKFYCISVLVVLILGSWACSTGFRSDKKIVIYCEYIAYFLISLSTKDRYIFDYYKQQPLIDGSTTSKVLYKKLERLLKYFVTITIVLKMLNIFVFCGWNLTKCINELDGVLFINLLWIGLLLARLSLPVIYGLLYFRLRVLRMTLESKGFSNSPQNRFTPKKYITIYEKIMKDLLKMDYPLKYVFIIFLIGSVPKLLQNSWQFLNSLKNYGPEISKILEFTLECLHSYIVIILPIVVALDLSEDEIKKMKIITLNKRLACLNERQKMEIQQLFLLLKNNSLRYNLWRVVPVNLKSVLIFLSFGVTNAIAIMQAKNLN

>BmGR48F

MSGNYLEEYINMSFSPIYKYQKFLGSNRISLKAKNKITVANNWEKLYAFLWMLAASYSIHHFISFFYSYYYERSKILFLGCSLGTSSQYLTYILTITYDKFLTREADIDLFINLQKIDRLLKLDRCTVLFKKIRLIYIFLLILVTVPFISGFLIHVFDYIDQPYKTFFLGLGLTIIYVDVLVTAFFIANLTLRLAYINDRIAMYYKRSLPLRKDSGIRRSRSWICGSWIFQIMPKIKNNGTRMKNYTFIKYQSLIFNILKCYRLITEIYSLPVFLITATVSIWTFLVIGSIVAGSRSEIKLFSIVAMITVGLWNLFFIIQLTSLAFVNDLFLMEVKNTKQLCIRVLSYTRDDSINKAVNTILKDIEYAPPIFSVYGIFVFDKSIILFLIGIITGNIMTVIQFSY

>BmGR49SJI

MAGIRTISFKVKPLELPDVSENNFADDGLKIVQRFKFFIYIQVITGINRLYLLKCNKFVMLFSYLYAIFLISFVASVYWTKEPMKNSHLVIRLFSFIEYILLICISVFLKKKKMMKFFENLSMFDQILKIDKNVNSTFCMKRVFFWVTGSIVYNLIEFYALEFYDNTSKGLMTIICTYTIAPAAIGVCVYLIVHCIKYTLLVVIPCYYSSITATQVSLIRITLHDAINTIPLGKLQRRKVKAFYLMTKEYSFVYTLAGVIKLNMSLPLSYISLCTTYLVIIIQFSKFLD

>BmGR50S

MAGIRTISSKVKPLELPDVSENNFADDGLKIVQPFKFFIYIQAITGINRLYLLKCNKFVLMFSYLYAIFLISFVALVYWTTEPKKNSHLVIRLFTFFEYTLLACISVFLKKKKMIKFFENLSLLDKMLKINKNVNSTCCMKQVFFWVTGSIVYNLIEFYAMEFYDNTNKGLKTIICTYAIALAHDCEQIFFFTLQRVVYLRLLVVKRHIQEYFKVDEDSSRKKPNKYEMLSNNVQLNLTALHEVYALLHNCAEKLNTVMSIPVLLMLFTSGLSTTILLKFFVRVIQLTDPSNPGSAIGVCMYLIVRCIKYTLLVVISCYYSSITATQVSLIRITIHDAINTVPLGKLQRRKVKAFYLMTKEYSFVYALAGVIKLNMSLPLSYISLCTTYLVIIIQFSKFLD

>BmGR51F

MAMGIRTILSKVKPLELPDVSENNFADDGLKIVQRFKFFIYIQVLTGINRLYLLKCNKFVMLFSYLYAIFLISFVASVYWTKEPMKNSHLVIRLFSFIEYILLICISVFLKKKKMMKFFENLSMFDQILKIDKNVNSTFCMKRVFFWVTGSIVYNLIEFYALEFYDNTSKGLMTIICTYTIALTHDCEQIFFFTLQRVVYLRLLVVKRHIQEHFKVDEDSNRKKPNKYEMLSKNVQLNLTALHEVYGLLHNCAEKLNKIMSIPVLLMLFTSGLTTTILLRILVRVIQLADPSNPGSAIGLCVYLIVRCIKYTLLVVISCYYSSITATQVSLIRITINDAINTIAFGKLQRRKVKAFYLMTKEYSFVYTLAGVIKLNMSLPLSYISLCTTYLVIIIQFSKFFD

>BmGR52J

MAGIRTISSKVKPLELPDVSENNFADDGLKIVQRFKFFIYIQVITGINRLYLLKCNKFVMLFSYLYAMFLISFVVLVYWTTEAMKNSNLVIRNFTCLEYILLICIAMFLKKKKMIKFFENLSCLDKMLKIDKNVNSTCCMKRVSFWVAGSIVYNLIEFYAIEFYDNTNKGLVTIICTYTFALAHDCEQIFFFTLQRVVYLRLLVVKRHIQEYFKVDEDSSRKKPNKYEMLSNNVQLNLTALHEVYALLHNCAEKLNTVMSIPVLLILFTSGLSTTILLKILVRVIQFTDPSNPGSTIGVCVYLIVRCIKYTLLVVIPCYYSSITATQVSLIRITLHDAINTIPLGKLQRRKVKAFYLMTKEYSFVYDLGGVIKLNMSLPLSYISLCISYLVIIIQFSKFLD

>BmGR53

MAHIKDENQSKQQQKEHETLNKNKLKKVVYTLKPALMLENWFGLSDFLLVNEDELVLLMQTEKFGVILSIFFIVMFAVFVDFPDTETESIMELMDEVPSMVVLSQYFIASITTSSCLSAIAIRIFETFADLDSMLLITTTQDFYNKSRYQTNKYLIILGVSHIISSTLDLLTDDEIVWCKFFVLPIYFLQKLEVLTFCKLIVMIQCRLQIINKYLTNFIEEQEKNKALVFTLAESNPKKTDKFNWIGCPSPNNMKIRDLATMYDVIGTICSLINDLFNIQIFMTLVSTFTYIVIAIWSTLYFYRAPNFTFGTLTTIIIWCITIILSVVVMSFVCERLVSVRNNTKILVNKVIMNYDLPKTMRVQAKAFMELIESWPLKIMVYDMFSVDISLMLKFISVATTYLIVIIQLSHFV

>BmGR54S

MITIKNNFGFRQNNLSFYRPILIILQLCGYDFDYYNINLVLNVLTKAYCASLTCVVVYATIACCSSIQLSHIWSLIEYGTSVVIIACFRSQTKLFLKQLTTLDVYLRISNRRFVLEKCKIFTITSVIFLLRIVYTSIYCSTHHCFNVLIYFLLSQFALVCLDVNRIWRCIVFDAIRYRLKTLRLRMEENPDCNYYLYVKNNKSIRKNKISFCLFLYRTIADLVDLVSPELNVSLFLSVACSLPKIVSNAYHLLLIIEDREPLETGGYVLMHTLQVSLLLFTPFIIVECYTMEVEKIKLYLVHRLIDENGEDTTMRDNIRLFLEYMSVRTFRYRIFRIVPVNATLPLELVNLCVNYVIVLINFTHLYG

>BmGR55

MERINLLKSFAFLENVMCIYRNFMFYNQRARFIIIGRIVAELVFYIFSAYNGFLLVYTDWFSQNFSVFFIEIISKSSFYVITFFTMVNGILKSREYKTFIFSINKIHDYILNDTDYLKRLKCTNIFCTATIIILFVVTLIRTAIDGSNYGQLSGINARSVIWMLTTILLECQYQTECVVYFGFILFIHAIMKYLNIRVTNTIIKIARSDMAVKRIPKYIIGRTELKDETDTGVDVNNVVDLEEVRYWVFIYRQLGLTTELLQKCFGMQTAFIFVTAVLNQIITVFRVIAVFIYGSLANRGAEHSIIANFLFTLLYRLPGLLMIIVGGQMVQNQTDMLRRSMARLNNIISNPHRETFSALSDFHRMIVKNPVKIYVLSVLPVGAYMLPLFMTLLINHIIILLQFNHVA

>BmGR56

MKKIRLLRSIVFLENLLCIYRNFLFFNKKARAIILIHITIELVLYVLSIVNNSFIIYSYFHSDNRSMLIVFTTICCFYVVTFVSIVMGILRSEEFKDLVTSLELINKFFTNNKTYLKSLGRSNTMIIAITTILYCVTCIGIAVDKITLNDFYEFTSSDVIWTVSSTLLELRYQTECVVYFGIEYLFLIFTKHLNLLVKEAIKKVSLDNNGTVKDVPISSDAVTKNEVKRWATIYRQLMMSSKLLQACFSLQIICVFVSAVINFITTAFRMVKVSVLGSIATDMNEIIIVNLIFTLLYQNIGLVLIIVTGQRVWNQILLLNVLLARLYNGILIQPCRDTLRTLKNLQRMVVKNPVQIKMLSVLPVGSYMLPMFMTLSVSYIIVMLQFGHVV

>BmGR57

MEEIKAIKLVTFIENCICVYRNYAMCTKRNKKIISLRIIVEIIIVFFVNINNILLLHKYYNGSGLLYIIYLFLVVYYINYMFCIFYGALQGKAYRQLIFCFNKINAIAKRDKSYKKSLARLKNMCIVISIALLIISALSVFVDRSNSWNIYEVSLRDSLLILSKIHMDFFYHFEYVVYFTHIKIFHLTLRYLNSRVKMAQFEMKMTRRDVHDEGERNIRILLTKELTTEWAVLYKCLVFGTKTMKSLFGLQMLIAMVMSFVNFTLSLYGIILICSIEQSQTASQHNLLLILTYYTATMLLIFIVAQSVYNEVEMLKRNLARMYNILAVDSDETQQKLVKDFLRMVYKNKVEIKMLSIFPVGMPMLTFFLSLSASYVVVMVQFSNVF

>BmGR58

MSSRRVLYRAEVLLSNNVDAHVQDMLKPLNFFQFILFFPKYTIRDGYITPNSLIRNIWSATGAFVFISICVFRILTMNKIAVYDTFTTMLLISKYFDVALYCIGFIVNTYVNIAYSNVNVLLYLKLQTIKTFIPRNNEIMKNVKWYSVILIIVLFCGTLAMFSFFHLSFSYFNIFDLTTDLAVFSFDLNLVYACSVLNFLAQSLDELNKEIWRLGNAKVTVCKDGSKPDWNGINLTYINVLDAYNYFKEAFRLLIFFHTFKTLTHMFIYIQSIIELCKKFYPGDDYDAITVGAVVGVWFFRNITLQCLVGVSCQNFYSATSNTESICAVQVGSIVSDEHKLFLKAVRRLNNVVFYKWSMYGMFIVDATLPRRLIELIATYTVVFLQFAFK

>BmGR59F

MPYKKDSNRCEVLLYNNVDTDLQDMLRPLNFIQTIYLSPKYTIKDGYITPNSLFCNILSAAGAIVFFSICVYRILTASKIGTFEGFSTTLLITKYFDAILFSLGFVANAYVSIRLSHLNVLLYLKLQAIKTFVPCKKIMQKVKYYSIVLIIGLIIVRLIMYIHFHWSLGYLSYLDLITDLGVISFDLNLVYASSIVKFLGYNLEELNKEILRLDEIKATMDEEGSKPDWNGIRRTYLKFSEAYNYFKDAFRILILFHTLNTFAHVFIYVQSVIELCKAPADNYSAAFSVLIAVIVWLLRNIILQSLIGISCQSFYSATSNTQSICSILVRSVLSDDQKLFLKTVQRLNKANISKLDVYGLFIVDATLPLRLIQVIATYTIVLLQFAFQ

>BmGR60JR

MLTPRSDLCNEKLSPSFPSGKTTAADKDDTEARCQVDSSLERLLLPFNLVQHVSFIPMYSIRRGLVSPDGPLAYLYSLLGFCLFTSVSVYRNAIMHGTRLSSLHLFTLYSDLVSFVINYSLSLICNVVNSKSNVEFVCRLQRLQTVLRRNQREQEQFARSNWAHLAVVTALYLAVVGLLNVVVLKQSLPDTLYLLLLFCIDVNVLYATRMLALLRCYLQLWTRKINEKAFNPVHHNMFTAYLDILQEYEVYTTLFKKIITYYVLETFLHGLLYVQVAIQICKSIRRSGRFSEQLMMIVSIFTWTIKNMIIMTLHNVECEKFYLAVEQAVAACQTQRASTTRCREEKRLYKNVCRVSRAAFSRERGWGLLAAGAALTLRFMDLATTYVTVLLQFAFVSRT

>BmGR61

MSIRFEKDLLHNYVEIELQYFLRPFNVMQSLFFQSKYRIVDNFILPNTLFKNIMSFVVSVLCALSFIYTIISVWQNTHATSFHALVTSVYLSYNIYGILIGSVLIIWLSDRNIEFVLKIQDLIKILEFNKCFLIEYAFINSIIMAAIFILNFLLYGYFVVHLQKFALGLTFSAIVCILNQDLDIIYVIIFANILKKCASRWTVEARQKNNFNDQGKWVKLFNAFLNLTESYQLYQKIFEFYELLRRVGIVFLGLQLTVCRVCSNDIKSIQCTVMLHAFQLICVWIVKKFITLSILSFEMEIFYEKLREIETVCIILVSSDNPSERELKIWKNIIRVSSCSVRKTTACGLCEVGAALPQWLLQATTAYTIVLLQFHITTFSRARNDIYDLD

>BmGR62

MNDLFLSKIVKWTKTTKYKLDDDFQSLFRVFNIAQAMNLCPKFLIYDKYITNNAWFIHILAISSFIVLVCLDSFFANFRLVLSEAMGPPFYGFSFYFISILYENIGVIIQITMNGYLTKNNVLIITKLQDTFKDFRTTDYITKSNRWTNWFIFFIYMNFIANYSYFNFYVNTFSFHKFCFAFIKMCFDLNIVYTIFIFKMIGDSLTMFKDTAFCSKNMKLYEVSNRVYWNKMLRLYSNILDVFELSKRTLNFFIFYFVSNILLRILSHVQLAILMNSINWLQHVAYSNIVMVLLTLAKEGIILIVLIAKCEKIYCVIGDVQTACQLALGNAACPEKRRFCKNVRRSSSAAFSKIYICNILAVDAKLAVSLMSVTTTYTIVMLQAILIK

>BmGR63F

MQIGNAVIHLKSTKLTTMNTISPTTKLLKIFALNSNIEEIDLKCSTKLRITMTAFVLCSLIFYSLYYKFIYVFDYVNISIKITDCVQMVYDFCQYIVDLYFVTNYGRNISSEYFQQYKIIDKILEVVCYEIIKHRIVKLLWVFMCIWFSSSCFDFIAWFLNYGWITPLVYSVAYIFLLIKILTTLDLSAHIMNVEIRLKMIADLIHHYYMSCEDNFQAEETLCHKNWLNSKERAKYYELQFRIHALKQLSCNNNEIKLLSRCYLMLTEQVEIINRMYGFRILLNSLSLLIDMVRFTNISVRIMIGSQNLAYNCGYFPAVSSIFRLLTCGAVIINLVSHCERVYYQRTRICNVIDHMIVNKNLSRESTEALQEFRNLVQNHPIEFNMANFFQLNYSLLVSIASVVVTYTIILLQSVN

>BmGR64F

MKISLRKIVSIRNMTLIQNMFGFYHKFTDNRAIGVLLKIFCGFYSLFLSFLCINCTPRFTNDFLTYDIFFFVIEYLTSVLVCLLYDGQYFLNYLYDLKLIDREAGIEESLEKLPISQPLFSLIFITRVIYLLSCLLMFDGIKDSLFLPAQSSVFGANFTEFARTIGYFPRVIMFEMFYKRVNYLKSQLRNDLAHANLYPIGFVCSKVIMKYINFYKLLLRNLQQNSLQFKILMSMSSLYIIIKALASAYAFIYREDGVHVFIFIEFATGVFLFFVMSSIIISIFNEIEDIRQIVLAQLRYCKQGANTKRVQDALTILNIRCFKYALCRIYTVDFTFILRILDVSVTYVIVLVQFTHILD

>BmGR65JI

MKISLRKIVSIRNMTLIQNMFGFYHKFTDNRAIGVLLKIFCGFYSLFLSFLCINCTPRFTNDFLTYDIVVFVIEYLTSVLVCLLYDGQYFFNYLYDLKLMDREAGFEESCSQKLPISQPLFCFVFITRVIYLFLCLLMYDGINDTLFLHGQISVFGVHVFIFIEFAIGVFLFFVMSSIIISIFNEIEDIRQIVLAQLRYCKQGANTKRVQDALTILNIRSFKYALCRIYTVDFTFILRILDVSVTYVIVLVQFTHVLD

>ZnGR1

MTGGFFKECDLAMMNTVTLNEENVNSFHHAISSVIVTAQCFGLLPVLGVTGPNAASLRFQ

WNSFRAVFSIFILLGAGLNMCFAVVLMQSGISFEQSDGVVFFGTVTTTYMLFLQLAKQWP

NLMVEWENLELSQKHYGYPRRLHLKIKIIATAVLTGALVEHALSKSSRITVAAAHTDNIT

DMFHHYFTKTGTYNQMFAVMDYSFTTATLTLITNFIATFAWNFTDLFIILVSLALTERFH

LFNQYLVSLRGKFLPESFWAQRREEYNNLSHLTRTLDSCISKIVLVSFGSNLYFICRQLL

NSLSPLDGVINTLYFCWSFGFVLFRTVTLSLYAASIYDESLLPKRVLYDVPPESYRGEVS

RFLSQIVTDTVALSGMNFFHITRTLLLTLAGTIVTYEIVLVQLNSITIIGSNTTNNMTRK

FYE

>ZnGR2

MAVATNECGTIPELMDPLAIIVPTRNTVDNVARSELVEYKKHSVSESDSFHRAFSSVIVI

AQYFGLMPVYGIRGSSASFIRFSWFNPHVIYTALSLIGTFFMTSLCVIRFFKYGRTFEET

QCSVFYGCSFSRMLLFLLLAQHWPALASRWEKVERVLAHHGYPYHQRHKFNLITAIFLSV

SFIEHALSHVRVIRLAVLCVVEDKMDGICTFFYNSFPHVYDYFPCSIWNGIIMFIINVIC

AFAWTYMDLFIVLMSIALADRFEQLNRCLQSIHGKHVPPRFWHHMHKDYNTLSSLVKTVD

SCITKMVFLSFANNLYTVCIQLLNSLHPPQNAVQMVYFCLSLGYVLLRMIVVSLSAASVN

EQSCQVRNILYSIPATSFSVEVQRFLHQATTKDIALTGLNLFSVRRTLLLTVAGTIVTYE

IVLVQYSGIHADERDSTAHGVARRYCL

>ZnGR3

MSGANNSFQRALCPLLVAGQCFALMPVSGLTGYDASTLQFRWLSPRVAYTFLSLVGIFCH

LCVCVRELVVADHITYSACAPPIFFAVTFLATCFYLRLATKWPDLVSKWQTVEQTMLKDY

GYPRNFERRCKFCAAFMLTASTVEHLMALYSCLLRALPRSTGGLDVVRAFYTSWFEQAFK

MTEYALWKSLLLQLSNFLATFTWSYMDMYVTLVSMALTEKFLQFGEGLRAVRGKAAPLKF

WREARQLYDNLCCLTRTVDEHISHLVLLCLASDLYFICLQLFNSLKNVKTLYGYIYFFYS

FGYLLFRASSMCFCAAAVNEVSQKPRDVLYAVPAGTYNAEVERFIDRVTTDEVALTGFRL

ITLNRRLILTIVGSIVTYELLLVQQGNGQGSFSEELEYAANITIM

>ZnGR4

MTISPQEEIHGHAAVADSGLSSFHRTIAPLTVMAQCFALLPVHGVTAPSPKALRFRWMSA

RVAYTIAVLFSLLFMVFFCILYILQIGGTFQDAQCMIFYGIATATYGAFLHLAGRWPRLV

VQWSAVEHAQSCYGSSKSLRLKIRCITALLLAGAAIEHFLNEYNNLSFAARHANTTSGVV

QQYMLRSHHHLFTYLGYSHACAVFASVAAALATFSWNFMDLFIAVTSIALTDRFRLLNRN

LQAFRGKRMSAEFWKQTRERYTSLMYLTDTLNLGVSHVVLLSFGSNLYFICLQLFNSLNP

LKNWYERVYFYWSFGFLLLRTATVSLSVAGINDESRLPRVLMYALPSDSYNDEVSRFQQL

VTTCHVALTGLSFFSVTRTMLLTMAGTIVTYEIVLVQFSHVNT

>ZnGR5NTE

HSHIFQNNQLLTLSRSESVAPVEVTRRLTLRHVQSDFKTNALSTRNLVYCDDDVNSMLSS

LCCVFMVAQAFALCPVRGITGRRAEDLSFTWRSIRVFYTIVVILALVVSLGFSFTWFLIV

GFTFTTSGAIMFYVTGLTCCVLFLKLATKWPEVAMLWQQTEHNQIRYGYPTNLRLKIRAM

TAVILTLALVEYVLSNANRMYIASQCSTGTFDLVRRYIMVTHRHVFGVIGYTTFMALFTS

IVSVISSFYWSYADMFIMLVSIALASRFRLLNYHLKEVRGKVMPECFWRSAREDYNALSH

LTSSVDSCVSSIVLLCFATNLFFICQQLLNSINRSLNTIQIVYFYVSFGYLLLRTIAMAL

YAASIYDESRVAKEVLYAVPAHSYQVEVHRFLIQVATDNVALTGLNFFPVTRTVLLTLAG

TIVTYEVVLVQFGTTSDSSSSNMTALCLDLTTVHYIQ

>ZnGR6

MEGAENIHNRQIFTVSGKDITRAPDEWRRITGGKHLVPPSAVEPSVIKLYPSNRIRDIRP

INYSENDQNSLRRSMSSFMLIAQCFALCPVQGITSPQVQDLRFRWRCLRVVYSIVMTLGM

ATTFIFTIAFYMKGFFNFETSTTFVFYALGFACCVCFLNLARRWPRLEDLWQRTEQEQIK

YGYPKNLHLKINVMSTVTLAVALGEHLLEKYTVISAVARCSDTKAELIELYYLMDYSYVF

QFVKFSPPLAVLCEIVNFFASFYWNFIDLFVMVLSMALSSRFRLLNNHLRLALGKVKPEE

FWREVREDYNSLSVLTKYLDSCISSIVLLSFATNMFYICQQLYNTLRKSVGTVKLVYFFV

SFSYLLFRTVAVSLCASSIHEESRAAKDFLYAVPTQSYKIEVHRFLVQISTDAVALTGCR

FFTVNRTLLLTLGGTVVTYVVVMVQFSSPSSGYATNDTELCLKMTARLH

>ZnGR7

MHLKRTGPQISFDLKYDHPVPIKWRQVDEASIRKDEVVIVISEAEGPSSEFFEELKPIIY

TMKIFGLFPLQKRIPGEPTFRRCKFSLMYSAVFYLAMNIYAYYVSRERIQFIRYSEASFD

DLMYSVIHFMYVIPHFYLIPCHWKEVSKVGRYFVHWSDFQKQYVRVTGKQLYLGQNRHVM

WSVVLTPVVALCFVATEYYMSSAEEKYWELGFYWYIFTVILLHIVWWWFTCSSLRSGIYD

ISENLFKDPTIFADEGSATLVAQYRALWISLSRLSCETGLFMCYTYGHLCVLSFSVMTLS

LYGSLSNLHDGLYLRHLGLALAVCVIGGVTYVMANGAHHAAREVGPEFCEKLSHVNSDDK

QMRRELKIFTRVMTLNSSEINLGGYVRIDRGFLLRFICTMVTYLVVLLQFRLGLLSITTS

NVTMVTTDVL

>ZnGR9

MAHVTTITAVLNGSVLQGLYVKSESNMNIENEKIRKNFAGEPWVVPLHAQNHPETTRRRS

SQRSPGLQADLMPILQLIRMMGILPINFQPNGEVDFKLLSWITLYSVLIQGIIVVTTVIT

LKERLGKLADDSADFKENVLEISCSLCFFPAFLGLINHLPESGKKARFFKDWGNLEREFR

RVADRDLLIWLGRKMRMKIVKSCVVTVAFTAILLLLQRGYTWWQAVIFTYCSLLNNLMID

FWVLTSDAFILTVEEIESALTESLSKKHRATTTCRVADYGVLWRHLSKQVQDLGNAMGYT

YGGQVMLYFTLQLIGTYGFMSRIQQAEEVTTVGFLISAFIATWLTYVFCNAADRATKFVG

REFQEKLRNLLNVSTSMDAETKAELRTFLETVIAYPPEINLCGFVQVNRGLQTSLISSIV

TYLVVLAQFQSNLVSDDETNNRTN

>ZnGR10

MAGRPGVAMLSPEEDFNVNKLRTAQQKNKKKNIVFPTTGTPRTEIQPGNFVYDLRPMFQL

QKLTGHFPLHTNDAGDVEFRLFSFLTLYTALIQGIMLTGGALIWRHRIDYLMREGPDFAQ

AIIEATFLGAFTPLFLSPFVQLPESGKTAKYVSSWADYQRQYEAVVGRPLDLNLRRSVTL

RIAFIILVCSGITSFPYVTLKEIRAWFALPFFYCLCSCVLNTGMWHINGRALVTAARTLM

EQIERLLTTCEFNCEDKIAEYRMLWLKLSKLTQDMGSSMGLTFGTQVIAYFVEQVLAVYG

LLSNLQEGFVMNNVVFVSCGLTFSWLLYVTCNTAQNATEEVGINMQERLQKLFHKIPRHF

ADSRQEVRFFIQTIVENPPKINFCGFVLVNRGLQTSLTSAMVTYLVVLTQFQISADSTIN

KNATCNISEM

>ZnGR11

MARVLELRSFQRTAKYSGRGYGTSEHRPSSQATKVFSYLLQNLQPAAMTTSAWEDKQEAS

TFYEEIKPVMILLRILGVLPYSTTSTGAVHFKWLSAAMLYSIGFVGSISTCVGFTLMGGF

AFNTDSKDSFSEKTIVFAARLFISSVFIVPINHWPETGKKMRFINSWTELQTEYRKITGR

RLHLELRRKVIVCLSVSAVLIPTFSLMVYFTGIMLYWWQIFGFIFAFAITMILPFIWVFT

CTAITKVARDLADEIEQHLPRGFVTVGKISSYSKFWLRLRKLMQDLGSSSGFTYGGQILI

YFAMSVLLSYGFMVDLGDNYNYAAVSSALLFQIVIFVQCNSAQEATNEASTVGTKFSERL

QSITNDLPVTCTEQIKEMRYFLSVIGRNPPTISFSGFVDVNRGLVTSFISAMATYLVVLV

QFQMSDKSSEQNDINA

>ZnGR13

MTMLHCNKSFSKMTTTITEYGGYIPPKRTASKSAARVFPQPLFVSPISHTGSEDRQPATN

LYFSELWPLFYLLKLLGVFPYYVSSSGKLAFKLMSMTTLYATASTTVTTVGSILTIIQKY

SKLSAEKMKFEDAIVQINLFGGFFFIAILPFVYYTETRKRIQFINRWGIFQEEFRRVTGK

TVDLGLGRRARFFVVMSLHFSVFGVISLGVLVGGFSWWQYCISGLFETMLLLMPAVWYLT

CRGLIRAAKALGNEVEQNLLHHGFRHTDALVQFKFLWLHLSKLTQDAGRSMGFTYGVFTL

FCFGGLVVSSYGYFVSGIILITQGLAFTAFFFSTILYVQCTFAQLATEEVGSKFQDRVQI

ILEKYPLISGTQEEIHRFLAMICSNPPVINFCGFVDINRGLITSLMSQTLTYLIVMTQIN

KN

>ZnGR14

MSVFPASNTTNGFVREIAPISLFGPRGQFVKTIPSNEIPRSKLTGVKESPNSFNRDLNPI

IKVLQLAGIIPITKTARGMWVYSPFSPLMIYSVCLYFTMLVILWFTTGVKLQELMATHRH

FNAMFFVFMAVSYFLIILAIPFVNWPEANSIAHYLSNWTRFQEKYCKMTGRPLNLGLRRK

VCILMCVLPVLIFSFEVIYQLTNGDEVWVDWWVAVKEWYLDVVRFYVVTFWHLTCGALSS

AARNLWRNFRKELAESSRHSLQVIDTHRVLWVELSRLVRQTGFAMTYTYGFYILYMFLSL

TFFVYHTMSNLARQLRADQVLMAIECCVMEYTLYLICNAANNASRDXXXXXXXXXXXXXX

XXXXXXXXXXXXXXXXXXXXXXXXXXXXXXXXXXXXXXXXXXXXXXXXXXXXXXXXXXXX

XXXXXXXXXXXXXXVGDEIRDGLLEARWSSNEKSVQKEFDKFLKTLDIFPPTVSLGDFTI

INRALFMSLATMMTTYLIVLLQFNMSSSDSQGYNITLQI

>ZnGR16

MTMLHINSTPLFRRVEPEPPIRLVHGHLYDLLKPLWLELRLLGALPVEKIATGCGQPVFK

QTFIATLYSVTIYVIVGVCIVYTGLVKLKEVRALKTSEFDEIVNGYLFLVYLAPHFAVPI

CNWIQAGQIACFLNDWTTFQTHFEHVTGNTLHMKVWHKAIVILAVLIPLSTTVSIVVDWL

VMPEFKLWEIYCYSYTCTLVHLHIALWFMMCYSVTSTAHRLVNILRQGLGNGRKVSDYHS

LWQDLSRLSRKMGDCVCYIYGVIIVIWFAALTLSLYGGLTGILDHGVDLRGFALLANALL

CTSVLFVISDAGQRLSEEMGPNFYCTLQTLHLGVNKEVRQELNMFQYDVAMNPPVISLGG

YVTVNRNLLLSLLATMVSHMVILLQFRVGAEPNTLNSAKRTNVFNQ

>ZnGR18

MYRREESVIIGRNRPSMAAMNKIYFENLKVLRFEVVDRSVKRGVTLDTAENKDELYPHQM

KPILVVMRIFSIFPVELSSEVPRFRLISPIMAYSVCFYAVTMISTYFNVCNIASALLFKK

NNLLFSTVLLSIIISVFMCCYMPLLCWVDIKGTVQYMKKWMKFQEIFLSVTKQHLTLALR

RRCFIYAAISVLLGTVLSFFGCYMPGAKFCGFPLYYIFLTLTTCLESYWIIGFNALAIAG

CSLAKRFKNDLRQTGPSAKMIEEYRILRLRLSRLVSDAGGVMGKVMIVNVISFGCLASMS

LYTSLSMDSEERKIEKIGMGTLAATGILHLYSLCNAASKATYSVKTMIQEELQFIRISSE

SDAVGKAVTAFLQTILLNPPIISMLGFADINRGLFTAYASATVTYLVVLLQFDLGERVDK

YDMNIVQNCTGV

>ZnGR25

MVSKPNHFLPAQIAHVGLQDPLAINSENRKKFYIGKPELCAPTGISTDADVSNEFFHNQL

KPLFIVMRFFGFLPIKLLFRGVSKFQLFSSIMAYSMCIYAVTTVIICFIVGDLSSVLFSG

KIDFLNTIFYSMLILFMFTSISSPVLLWLDASNYVHYINKWTEFQILFLKVTHNRLSLNV

KKRCIFHAIRCSTIGFAAIAFHFSTPYIRIWQIPGFFYAITLVSCYLTLWITTCGNLSTA

GKSLSRNFKEELRRKCYTAQSIGQYRILWLRLSQLVSRSGGAMEKTSCIFFAVYLFIMTM

SLYGFLYGITSEAEIPIQSIGMGIGTVTGGCTFYVTCNAAHKVHLNVKAEIEEELQLIMS

SRENHDVLIEVNAFLNTISSHPPVISVAGFFDVNREMLRSYASTVVTYMIVLLQFGMGQP

NNRQMNATQNTTK

>ZnGR32

MDVTGEYLYIHRKIHPVKRTYPNTVVNVMSAKSPIAVDSLATSLMANEEYHKQISPAVNA

LRLLGMLPLEMSSDGVPSFRILSPIMAYSVCIYIFKMFTAYNMICTVVLPSFLRNDDFVN

IIINWVSLIGLFILAFSSILMWLDSSKFILHMKKWSYFQGLFREVTRQQLVLGIRTRCLI

FSIINVLMGICSFGINICILGTNIWPLPYYFFLSTILGSHGVYRVVSYSTLITAGQSLVL

SFKNDEKQGNLSAYLIGEYRVLWLRLSQLASETGVVMGKATVFFLSLHLFALTMSLYGCL

AGILTKGHSVMESAGLGIGFMFGAASLYGICSMAHETTATIGTEIQEQLQLMSSHSLRPE

TNIQVREFLQTISMNPPLISVLGFVVVNKGMAKSYASALVTYMIVLLQFNMGQPDGKCDC

NVTHNGTEPRIIY

>ZnGR34

MDIYSAVYPLNCILRVLGLTPFNVVGVSGSRRLQDSKILTSYDLSIIMCLAILALLKLTL

FMEYTDHSFFINVRAIEYAAIIVTNIILRLACLLKKSKYESFYKTLNKIDKNFQNINRTY

SKVFWRLIYVLLITFVLLSFYYIGRCQLHLANNRSGLFPSIVGCLVTVTSGTTYLVLIID

FTSSVCIINQRFKALRVIINNLVLLEMPIVTSEMHLLNARIVSLSSIKRKLQIILKVHGL

LCDSSSFLTAAFSVQLLFIFGVSFLTVTYNSYFCVLSLFNQSKGVFSGTEWNLIAFYWLM

LTFLSNMALLSACNTTTHEAKQIAAVVHKLFNKVMDPDVRDELEVFSLQLLHRKVHFTAC

GIFPLDFTLLYSIFGAVTTYLVILIQFQLSIEHDNKVSDATGPSLRVSMTPDPI

>ZnGR35b

MDTFPLVKALFSLSKFLGLAPFVMVNTSDGCALRVSRGAVIYSTGMLILISAFLIDRISF

RKDFHMSSWPVSVANTVFQLSTTLFTYWASYISCITNCSNMAKVLKQIFTSVFSQNVLLL

KYKLFNFILAFQIFGGFITFGVLYYIEWCHDLYPDYSEMIPYIVIDCCDYVYVLQLIDLV

LLLGHYFDVLGSRIHKLCKDDEDLISVKRNTIVSLSHYGISYEKTSKISSRVKIQELSCL

YNSLCDTAELVNSIYSFMILVNSAGALVGITYGLYIASITIFDRINAHDRELNPLLPTLS

WSAFYVTMLVCVVTSCSSTCRKANRTAVLVHKLLSKTRDPGVKEELQLFSLQLLHRKVQF

TACGFFPLDFTLLYSIIGAVTTYLVILIQFQLSFANPKQNGTVSAVLPVSTSSDLFTPTL

PP

>ZnGR37

MHMNNSLYFIYRVSKFCGLLPVTFLRSGGYEKPVLCKKSLFYSCVLFIAISVVQLYFVVH

LSMERIHTNSDVSHVNIQIILSLNACIMTTYVLSAITRLIRMHNFFKISYKLRSVGSLIN

YHESTNSYNTVIAVHSILIIAYLGRSVVGWIRINCVLSLLPFFVSCLMCQIITNFADIQF

TYFVFIVHTYFMLLNSRVNEVTASLQTVKSKECISFNRNNTSEFSAENSLVNCDFHELIT

IHDALCDVLDVINSTYSLQVLAMVGWKFLNVTIGFYLMLSVILDSKIYQVLSFSSLMFSI

TCEAMQLVVVVLCCNSTSFQANRTPVLVHRLLSKISDHGTRAELELFSLQLLHRKVQFTA

CGFFPLDFTLLYSIVGAVTTKGITKYCFFYIVDLCYFLTCIFYTKFLDRTFSLYTIRIIF

LIIRILH

>ZnGR38

MCKVYSAFHLLFYVSKFLGFAPYALMKNGKLIPSSIAKKYSIILCIVVIAAEIKVFAVEV

MKDTPLLLVGLVLLSLSSVVTHILSVFMSINGSKKFIRISEKINIFDSMFHQTLDVRKKQ

FYSLVAQIMIGSISLGSYLFWANIYIGNKEEKWEGVVTKLICVLGDYIIVLQYTNLVVIF

GQYFSHINIQLIELDRIFSQKPTARHVETGIFFISHVSITSAKPVRHELRDRVSCFMDLY

EKLFDATRFIDSAYSLQILLIVAKIFVHITFCLYLVFVVIFSTYYDKCYSINALFFCFWA

IFQLVLIIVCCDYTKGKANHTAVLVHKLLNKTKDPEVRDELQLFSLQLFHCKTQFTACGF

FPLDFTLLYAIVGAVTTYLVILIQFQTAYPGRRNVTKGIYLPSYVRISEIDSNRHPF

>ZnGR39NTE

FLLNSKKIARQLSRLNDLIGDIYYSDWKKDIQTMLAMLILTVTMILISAVVEVMSYTSYD

PSLFMPYYIAEFVCIMSECQFAAVMIILKRIIQNWNVQIGAISEVDDVINLPNSLKQINK

RASKTFTVSNNLKISDRSNIQSNVMRFQYLRELHVSAREVAESVNSAYSLLLLLSTAIMF

LSLTHILYFIFMDFVVQKNSLLCNETPNISYFIWLFYYVLRLIWLVYFPCFAAKEANRTA

IVVHKLLGQTNDLVLREEIQQFSLQLLHEKVKFTACGFFPLDFTLLYSIVGAVTTYLVIL

IQFQLALPDSKKNTTHHHYPPVTTPSSSENHYAHQT

>ZnGR41

MVQTSWRTTNISSINLMYYVSKYFGLVVFRWNRDFPVLERFTISIKLIVAHISLIVSLIF

IDLHRFESEVSSALDIMIAVSKPVSHTIVVTICFCHQTNICDITKNLVHWNYLLVGLKES

VCTKIRVVIPQLLLGIALIIFSDMSEWFYVERCSIYFPRNAIYTIIDLSMYVVELQFINF

VLHLQQYFAGVNTHLANGCFTAKRIMEFNYIYKPPKLKTVEYLHSFYDSLCDVAESLNCI

YSPIILLDIGLSFFRSTQKLYRIVFSNFVKDSGTLYPHMLVNRCFHWIKFIFLIYACSSC

IKSANRTAVVVHRLLSKAKDHDVRQELELFSLQLLHRKVQFTACGFFPLDFTLLYAIVGA

VTTYLVILIQFQLTFSEQRK

>ZnGR42

MCTTTNIYSVAYSLVWFPRILGLAPISMKEGSKIYKFQSSTKILVYSVLVAGGIATLSLY

IIYTMDYNFKRGPTIIFKIKEYAETLITATFIIIICVNHRKLIFTITNTSYVDRKLLFIG

VPITFKSHVRILRIRTLCAVILVLMPFFLFSYHFVIDTFGILHILSSFLSVFQTTTSFYM

QSLYQHFMFLIRQRFQIINTQISKISTDASEHGVLNSELFGPLIREPLPSSSNQIIRTST

FRIPTNNVITPAEKRNSADGNLSCIMNTCQQSCKVENTGTFHAISSSDSKCLAIQLPEES

QKSRVRLLAHLHDCMCDTAEVLNSAFSVQNLLLSGKYLVDITVNSYCCFFVFKVPHMNTL

LTHYMYYSLYMTLCSVMQFTGILAASTFTAEEANRTSVAVHNAMQKLRQPSQKRELKEFS

LQLLHRKLRFTACDFFPLDFTLLHSMIGAATTYLVIVIQFHLANTSGLF

>ZnGR43

MEQHLKAVKALLCVSRILGLVPYSLSEGVTPTLSVPALVYSILFVVVNVSFPWYHLVRVH

RLGTSHAESVPELGVSILSVTAILTTMLSTSISLFRCRHVLQILTDALYLCSFIGDKSST

PQKSLTIIIKLLLLECIPFFLLYLVFCASLDDITMGLFSMPFSFFILFGPFMVIVQFASF

VLLCMHLLSCINTCLGEILTELSFPDSDRIIKLSPLRSDFLSAKRHLRRPSFKIIPSKLT

PRRNHVSGMSTSSPQIFSVRPKNLPGLISRGKFEIPSLIDFHTDVCDIVRRINSAYSARI

LVNVADAFVSITVSLFCSYFVPMQHNRGKSTSLFIMLIYWNFMKLLVLLRVCTICAEEGS

RTAFLVHKILTKTRDPELREELQIFSLQLLHRKVQFTACGFFPLDFSLLYSIVGAVTTYL

VILIQFQLAVPNTNRNETTSHGRGTA

>ZnGR44

MPEDIYGAIRPLYYLSKIFGLAPFSFVAEVHPDGLRSNRLKTTYIDVFYSVLIIVISLTA

SATVLSWRIQNSYATDKHMTIILTDANVFCLNSSLALVSVLLGVAFNTRRMTKLVNEISR

IDKILLTRPRDVYKKTYIFGVLQVAYLFCTYVLFYYYHSWVWVGELSSKNFKHLPISYFI

RSVIYVMETQYINLIILFRHRFREINTYLKQIYDNDKGDLVHKIELIYFHNSCALNEVSG

PSILSQSAYVPITTRRSNHSRSFETKISIGRYSTSQHQLLLMTGIHYALCDMADCVNSMY

GLQILVDFTVSFITLTTCIYFCIRFIIQIHLQSEISESEGITRAIGISVVWLTLVIIRLV

AITASCSATTAEANYTAQVLQRILLEPGLHPHAMRNAQLFLQQVTNRPLHFTAWGFFIIN

YNLLGSIIATVATYLVILLQF

>ZnGR45

MPEDIYGAIRPLYYLSKIFGLAPFSFVAEVHPDGLRSNRLKTTYIDVFYSVLIIVISLTA

SATVLSWRIQNSYATDKHMTIILTDANVFCLNSSLALVSVLLGVAFNTRRMTKLVNEISR

IDKILLTRPRDVYKKTYIFGVLQVAYLFCTYVLFYYYHSWVWVGELSSKNFKHLPISYFI

RSVIYVMETQYINLIILFRHRFREINTYLKQIYDNDKGDLVHKIELIYFHNSCALNEVSG

PSILSQSAYVPITTRRSNHSRSFETKISIGRYSTSQHQLLLMTGIHYALCDMADCVNSMY

GLQILVDFTVSFITLTTCIYFCIRFIIQIHLQSEISESEGITRAIGISVVWLTLVIIRLV

AITASCSATTAEANYTAQVLQRILLEPGLHPHAMRNAQLFLQQVTNRPLHFTAWGFFIIN

YNLLGSIIATVATYLVILLQF

>ZnGR46

MDFRSSVLPLYYVSKLLGLAPFLFNKNVSVNREQKTSKLLSPSVIWSLFIFLSHFTAFLS

IMIWSILYDYREYKLNVTVPDALTIFLMYGTCFASLLIGAVLSRNRVETVMTNFTAIDQI

LLQENRDGIYRKTRLILLIQLSVLVAFLVGFYCYHVYVWAYGTSYIYLISKDITNFSNTI

MIIQYINIMQILRHRFRILNEHLSSSCGSKSLESHYSLIKHQNVINRPNFRTIQIENVAN

NSTETPISHSYYLSELPMTTNNQRPEEVLQIHTLRQAYSDLYDVTELINKIYGYQILLEV

GYDFVSLVSYLYYALETLNVKMTVDHNQRGEESFILEVVSSLCWVTQNLVRVLCITGSCF

AATEEARRTSTVVHKLLLRQSLKGDTSAELQLFSMQLLSNKVQFTAGGFFSVNLSLVYSM

VGVATTYIIILLQFK

>ZnGR47

MNLFKPNDVHSATLPLYYVSRLLGLASYRYHIRTSTHPPGPSRSRNGGDTFDLEREFQTS

KCAVLYTGLMLVALLVWLVYSLVWKILYDFSDVKLTYAVTQIMTLCLSAATTVISLALEL

TCNRRKLKKVMLKLTHVDKILMKDSNYVHKKMSCFVVLELVIFVCLLLARHGYELWSRGG

KQYVNIIIRFVIHFMSTLMIVQFVSFIHLLKQRFRCVNRQLSFLGGIKDQERGLEIVKMG

FTHHRFGVPVSQNAVVVPHADDIQSSKSQEQGSVATSIFISDTETGNLPSTSSSSKFRSP

CRPHDVDTIHTLRCIHTILFDIGGLVNSVYGIQLLLAMAYIFMSVVKYFHVVMISQQDQL

SSFRVNGIVPLMCVVSIHIANVLWVTASCNSACFEACRTTTLVNKFLLVQPLTSDISAEL

EHFSQQLLHSKLQFTAFGFFNLDFTFLYGFVGGATTYIVILLQFQ

>ZnGR48

MMKIISKTRNIFSAVKPLYILSKVFGIAHLRFKSSVVAPDNFKGSVGLKGSTFGSCLYSL

TILIILIIFHCVSLHLKIRNIYKRLKYTFILTDSFNSVLTFATVFISIFQVITFNRNKLL

VIFSKLNKSDKFTLRYSESDVRYRKTYILFLLNIMFTALIFGFLCTYDVFSNFKENIIIS

MTRYTSHVIRILMNMQFVSFNLLIKHRFDLLNKQLLSVFGVRSERELENGILDDISMACT

RKYKNPPDYRNARFQKKNYTNWDEYYDFDKKLFCKESLMTDREYSLNASSESQYMLIQLR

INHNEICNISRLVFSTYGVFTIFELLNIIADIITVLYFSLDFVISESYISLEKICWCSSW

LLLHAAKLIGITRSCQLLTTCGNHTSVLVGKLMLFSRPKSRNTMIQLQQFSHQLLHTNIN

ASACDFLDLNNTLLGSVAKVAITYVIVLLLNQKI

>ZnGR49

MQNERFIRKTDFLSAIKPLIYASKLIGIAPFRFTDQYPGKNNRNNKYIETSTWGIAHSLI

LILGLSIAFIPHLNFKITHIHFSYSYTFVIFDVVGSVFLFLASVMSLLQQAIIYREELKD

SLLTICHIDTYLLRKSYSTVYKKTNIILVSQLFITFSYFVTLFSFDFITRRTDFGFFHCF

IRYVINVLDVVMSLQFVNLIFLIGHRFTVLNMELSCTITANQMNIEENASSSEQNDTVSV

IEVDLNNTIRREKRFRNILRYKSDTDTHIYTNELLRKRLRNQIVLTVRTLRRIYLLLHSV

IMTVNSCFGIQIMLTIISSATATTLNIHTLIVVLTKDLDTPVKDTMSTFLMLNLAWTVPA

VLRLIVITASCEMSKKEAERTAVIVQKLLLNRYLDHDILEELQLFSQQLLHVNTNFTACG

FFTLDFGFLYSTLGSVVTFLIFLTQVWESYGQHSRVIV

>ZnGR50

MWKSDNLFSAMLPILLISKFVGLAPFSFPSSMKRDTGNLIEINTFKTLICDITYSVAVLI

LYIVTFPFFIMWKISKMYSTFGDRSITFIIIDCLSVSLQFMTSFLSICEIVLRNRQIIRQ

TVLEMCQIDVLLEINSTTRVYRKTGVILLVQVLFWCAALGILYHFDYELWYKGKDILSGL

SKYETNSIRLVMVVQFVNFVHFIKNSFKTINLEIVKAFGFCDEMELDDYLLQSSQWYLMK

SKNAKLTKYRHANLSMPHLKYSDFRKESLRRQSMCSRIHVLRDVHFRLNHIATMINSVYQ

VPILFEVMAVLTIIALKFHLGLVTIFFENVTSSPMTLFLSLNFWWIVLHLSKLILIVVSC

QYATRHSARTALVVQKVALMKPLDPNTLFELQLFSQQLLHHNLAFSPCGFFTLNHCFLSS

CLESLVTYIIILLQFTHAKK

>ZnGR51

MKRMQHVNIVSAVRPLYYVSRVLGLGLFSFTDSFVVKEIKTLWILYTVILLIAVLTCGVY

CTVQRVGQSGLLATIVVNEFLMMFIGALKAVSSIFISVAMNGAKSRKFISTVVKVDRSLL

SDPITTYRNTFIFTLVQAVAVWSYIAILFTYDTWVWKQVAEKMSIWYLISTYPHRIVNTD

TVVQSCNLVLLLKNRLKTLNKRLGSILQESDEPYIETALLINVPYNSVFSHTENERLFSG

MKRSSLMSHVESPQTVNRLRQHTKISKKWNLYNIRKLYDDLCDISVLVNSMYGFQLLLEL

GVTTMELASALYFILAAILKIQTFERDIIGHFISLMAAWIFLISFKLISITAPCHSATTE

AENTAVLVQKLLLVPNFDHDTVAELQLFSQQLLQCKIKFTAFGFLILDYPLLFTIIGGVT

TYLVIAMQYEK

>ZnGR52

MERVLPKANFVFSVRPLYYVSRVLGLGLFSFTDSFVVKEINTLWILYTVILLIAVLTCGV

YCTVQRVGQSGLLATIVVNEFLMMFIGALKAVSSIFISVAMNGAKSRKFISTVVKVDRSL

LSDPITTYRKTFIFTLVQVFAVYSYEATLFTYDTWVWTRAMGSLSIWYFISGYPHRIVNI

GGVLQFCDIVLLLRSRFQALNSGLGFILKDSVDPSIVFTNVAHESASSSVVMRNENSVSE

DKSVTIIPFRDSSQFMVNRIRQPRFKILKQKNVHDFREIYDDLCDISVLINSMYGFQLLL

ELGVTTFELILSSYLMLATILGIHNVETDTISQFISLMTAWLLQYAFKLISITAPCHSAT

TEAENTAVLVQKLLLVPNFDHDTVAELQLFSQQLLQRKIKFTAFGFLILDYSLLFTIIGG

VTTFLVVAMQYSN

>ZnGR53

MVCKVLRLLSSVIPDSNTLLLLLLLLAVNSLGTRNCQTMSGSNIYSALKPLYLATKLVGL

APYPAGKTGTAIPDVTTKSRFGFCTLHNYIAFLYLLAWFIFNVTWEGIYRYPKLSNRSII

PIVIRTCSFASMCLSSLILCHRGTHEEFCTKIALVDQILLGKKASLSYMKTKLIVVIEIL

LMVVMSCFMILFDMNGRNVSFISTVKLSGWIVGGVIGTITIIQFVIYVLILKNRFRKLNR

QLSAMVVRGFEKETLETLLPILDQPNKIGFAGEVNVVHGSKNLHKNDPLFLSVSNIRNQV

LQYNNNHIRALRLSHGILCDTVKIVNSDYGFQILSELSYAFVSFVMFSFVAMDAKNDPTV

ADCEIGSSCVRVITDLCISCLCILKVVTIAASCHAVTSEISDTSIIVQKLVSPRYISGDT

LSELQLFSQQLWNTDSRFTAFGFFELNLNLLCSMAGTATTYIVVLLQLK

>ZnGR54CTE

MSCIYSALSPLYLASKMLGLAPYPRGRTGTAIPDFTTKRPLGFSVLLNYIVILYIISCFV

FDVVWDATYRYPNLSSRNVIPSVIRTCTFKGSSLLALILCHRGSLTVFCSKMELVDKILM

GSTETLHYKNTKLKIIAGLFAVFVSGSLIALCDINGRRIDFISIIRVFGFVFGGAIGALV

IVQYSVCIMILKNRFGKLNAQLTSMVVRDYDEESLEAFASFLDHPNKMHPEDAVYLSNGS

QDLKEPDPLFLSLSKIRNHLFHHDRYHIRALRQTHGILCDTIQMINSDYGLPILFIISYA

FISFVMYTFVAMDAKHDPSVAECDEEPSCVRVMMNFSISCTCIIKVMCIAVSCHAASSDA

SRTSRIVQKLLSQRPIRAD

>ZnGR55

MEKNISDDFFSTIRPLFYISRALGLAPFVYVKKTLPGGRISQRFECNSAAFTYSVFVAVL

TTFSFSCSIILKATLVYSEMTSIDRIPDIVLSALSIGCTVSLILSLTKNRNKILRIFFLI

QELDSILLKNPGKYYRKGRVFVIFQLTIALSFLGFVITYDSIIWTLAVGVKNFAYIHLYL

DIVIGWIVVIQFMNFVIILRDRFSLLNIHLSRLSNIFGIRNPNEGVYQPVLKFIKIKQNK

SKLTEEVVLNLNNIHDVLCDTSLLVTSAYEIQLLLTLISTFVSMVTWLYFGLIYFYRYVS

GYNTLVGVWNLVVSDMLWCLKHLAKLLCITVPCHLANNEMRQTSVVIRKLHFVARHDSGT

LAELDRFSQHVSLRQFKFTAFGFLNLDLSLLMSMLGAVATYLVILMQFKISDKSSSSCCK

NVTE

>ZnGR57

MDVLYTLKPLYYLSKFVGLASFSVYFNSRTGENVLRSSGVFNFVQNIYTVVIFCGITVGF

VMCVQDVQTKLSTNPGQVVSHMFSSPANFITSLMDIILMSVVNRKQMMKLVMKLFTIDKI

LVQGKELKIYQNSRRRMLLELIIIFGILVPFLCYDSYFFGRYSSHIYEGMSRFSIVVNLT

TVIQFLWAMRFFKHRLSLLNRKVLSTFSCQSNSVMRHKYPSHRETEKPFCRRGLSEVKQG

CGLTDIFGVFKEGNIRTLTVLPVSDGKLSGFLHQNTQFDGVSYILNLRITYNHIYEATIL

ANSVYGISIILTLMHCFVSTVSNTYFTFLDNFTNFDQLEKKLSNVGYYIATHILWIFISV

GKTIAISGSCHMVKEESKILVNNLQKLQLLHPIRSDVLLQLQQFSTQVSENSIHFTAFGF

FSVNLSLLYAFIASSVTYVIILIQFRLN

>ZnGR59

MWGMQLNTIYNDARPLHNVCKFLGLAPYSIRINPLTGEGFIDTDFKSNTLTVIWTLILLC

AMVGGLIDTIIMNVMTFNFSTSDIINKAFCLPMFYMVSILMIVMNGTVNKYKIEQFLSKI

SEIDKVMSYMKGQEYSKIKSKKYSSYFEIAVLIAVFLPCLIYDVCSFMGDVIILCKIQMR

LCEILQFVLMTQHCQLTRFIRVRLKMLGRVVSAVFNDSCSEGESELPGSTREAFNSKLST

GVTRISPELVSNKKLLAYKIFHLSEANTVDLFSCNAKNFARLRRVYSLLCDAVGFVNSIY

GFLVMFLIMQIFIELITGVNGIMHLIKENNSPHFANATLLIVHLISRIVLSLGILVTTIL

SCHVTVLESKELGIYVQKILLKYPLRSDTVQQLKLFYQQISNNDIQFTAFWLFNLDISFL

CTIFASSITYIILLAQIK

>ZnGR60

MCSNKMRKRYSERSHVLQEMKNILCVSKLTGLFPISFNKDCLTADESVDITISANITGIV

CSIAMCCLIVGGFLTALIQPYDAISDPGDFVIKILYIPAMFAMALSSIILNATINRQKLS

KLITKLMWINEEILNHSGCGNENIVKRFSCLMSMALFVIFLFLCYTVSFWGKSAPLLYCV

SANVAKFLGVVVVMQFCKFVNCIKFSLEEIVKIISEYVAEDYIGNHRMCDENNIKLTRIE

VSRNLIAPQMNMNNSIEPLPGKISLPYFINLFPCKHTELRNIIFCRKIFSEVYDSVLLVN

SIYGIPILLEYISDCSALISTIYLVIMNVHKYTENLNDANDEAIIISCVGGNIIMFSTIL

YVSLTCHLTTLEcRKICNIIEKRLLVHPINKDLLQELQLFSNQMSNNRIKFTAFLFFVVD

MKFFGTFMASALTYLIVLIQFN

>ZnGR61

MSGTLVHDSHILHEMRPLYYVAKALGIAPFSFKMNKVTNKEVVDIRFISNIGGFTVSAVI

FITLLTGFISCAVEPEFSFRSDPGDALCNTISVPINLISSLLLVIMTSTIHRYKAEELVD

KLNVIDEGLCQIRGECTYLRDKNKVQLYIPILALTTSFMCYDAFVWSKHLNITFCIIKRF

SHLITLVAIMHFCKLVQTIRSRLFGIHEILSLIISGKLSQTSSSYIFSHESRKAINKACT

FGSNIMQMTSVDVLSNPMSLKNVTADLKTLPVTDMQTILNLRIIYNHIYECAKIINFLFG

ITVLIDMSRTFTGLTAGVYSVVIFFNEPIEDVTSLNFSEFVISRTIWIIILLGTIVSLTA

ICEMAASSAKGVGHKVQTLLLENPLRNDVLEQLKLFSQQISNDKIQFTASGLFTINLSLF

CTFMASVTTYIIVLYQFKPY

>ZnGR62

MWKKRDCRNGTRDAFKTVHFVSRLFGLAPYSFVTNPQKKEETIDISWKTNLKSVIWSLIM

LTVQLIGLIYAISSNFIHNADSLSELVARTLQFPLINSTGLFALALSVSIRPKKMLKIVE

TLWKMEKYHSKINNVYYKKHNVRFTILATISIIYHITVHSVNSYFYPRGHINFYYGVSII

LCDLIWFVNNLQYVNTVEVLTQNLILMNKNLHNVFVSPSRSSHLSRRSRLYSRKKVFCLS

MVTEELYNRDHSAILNSQKPVGFRKNISSITSTAIAQIMELRVCFNNLYQVCRVINSMYG

LSLLMDFMAYTVCLIGDVYVIWCILITPYKGHKLISVTRATTALLWVIASSSNVFSIAFA

SFRANNEFKKTLREVQKLILRTRLKSDVQEQLNLFSIQLVNNKIEFTACGFFDVNFKLLR

TLVYVVTTYIILLVQVTKFN

>ZnGR63

MPRMGNVITNIKFAIRPVHIVAQLFGLAPYSFARHPVDNEEDIDISWNSNYGRVIWSALL

LIIQTVGIIYRLAFNFVRPPTSISELLSNVIHLPFIQSTGVLAVLFALKNRTKMLQIIKR

LSSVDTFLFKSNDKVYRKHNLFLVVVLTCCAVCILPLYFEHHWRSGGRPAEYILVLSHVT

WLINDLQYLNLLVILKERLVSLNDKLRSVFKVDSYNRGEFKIPMQPVSQGISESISQSEF

RLHSVFVRRSFQISLTPELPFSHNISEIASEILTFRLNYNALYEICGLINSMHGCIILFN

WSVYTVNFIINLYYVTISSFYTSASDKVLTSTIVNVTLILSIILSLIRMSIIAVFCQWTS

DEHQRCLSNVQDLELMYCTEEDILIQLESFSDQLANNKIEFTACGIFPMNLSVLCTVIGL

VLQYLIFLFQMIGLSEH

>ZnGR64

MFVKEPVNTNVYYSVKPLHCVAQLVGLAPYCYIRNPQTGEELIDISRSSNVKKIIWALVL

LFTQFIGIIWYTADNFINPPNSLVDFVEDTIQFPFFAATSMTAMMLALTTNRSKMLQIVK

HLNVTDNLLFHNPNNIYKQQHKQLLIILLCVTVSSIILFYFDVYYYLTYNPLYVIVIYVP

YFIWSINELQFLNVVEMLKLRLTTLNGNISMFSAKEYSIGNVSSNFVYMRRRHTRNRYAS

NIDSSVNTEECMGKTLQISNHESIRTSHILKLSEIYNRLYEMCSLLNSMYGYMLLQQFSA

YIMCLVVDGYILVRLLIDLYKADDPLIPREGYTALLLWNVSNVVRPFVVCLSCQRLKNEY

KRTVYYVQTIKLHPDMSREISNQLKLFANQIKHCKLEFSACDFFDINLSLFCSVILTATT

YITVLVLLES

>ZnGR65

MQEALRPLRFVAKIVGIAPYTFIMNTENGEVTVNISWKYNRRSVVWSLLLLTVGTASALY

KITQNSIKHLESVTRLVSTSLVIACVYVSGLVPIFLGLTTYRKGMLKLVKILSTLDKCLI

RHSDNMNKEHEAKILTFLMCFIVLIIAMHGLIIFSWKNYGILSGLILCFADLTWLINDIS

IVITVMLLKERLSIMNKNIETILIAELYRYNVANKSNSRVARSRMHRLLTASRLGHVSEF

VEQRSILHHDVQNSVLTVKAVSSCHQQKIIAIIATYRKIYQRLYDVCCLINSMHGLSLLL

STISHTLRFISEVYHAIYFLIIPYSSISGRDSKTEIMLLIICSLITSIRMISIPFACHKT

CEEYQKCIDIIQELLLRSDQKDITSNLKLFSSQLENNRIEFTAYGFFVVNLSLLTTLMGV

TVTYVILFIQS

>ZnGR66

MKTFDNIFTAIKPLFYLSKAFGLAPLSWIKEKDSGTVKPSSRSFDVYWGAVLFLCFLVNV

PLEFYVMQFNNWNYPIKLNVVCCIYKISCHLSGMTSLLFISAFKRHSLPQILILISEVDQ

LLYKKPERLILYKETRSFISRELCILSIIFIPLLVTYFYFSPKEGLLRYFVLMIESIQNL

SFMLMIVQFTNIILLLRQRYKCLNRWLDIHSNIWRDRIKSVGKDVPLLSDVETYVLSRYS

LKYGRLKILEQRHIYSKLYDIVCLVNSYFGLPVFMLTFWMFVSVVFISYSCMLSIMLVIS

TGKELGEHVWAMTGLIWSFACVVLLLLIIAACHTTTEECGKSQILVEKLILRSGLSYETI

NELKFLSAQLKNMKVSFTAYGFFSLDLPFMYNFVGVICTYLLILAQFN

>ZnGR67

MKGFKQKYVSISSSFHPLYYVCKIVGLAPFCFTVSSDKGVEIDINIHSNIVGVSCSLLII

CMLLGGTLCYFPVLASSRNPTPHFITSFMISFPLTVLMALLAITMNLTVNRHKFGELSRK

LNDIDRALLKYGVSKRNVWSNIEMVLLLLILIPWLCVDGWLWTDRMGFVGEATLRLSHLV

QILVIIQFCKLTQFVWQSSKFLNKALRVSVRHDYERYRVIEKEFLNNPCSNMSSVNIISM

TRRLPRTHNFPIVENVFQRRATDKPKAFLNLLEIRCIYGNIYEAVGFINSIYGLSVLLEL

VRNVLTVIVNILQTMEVLKKPSLYGRPSLLYASLGVLWIVFLISREVAIAVSCHMATSEA

KKIQDNVQCTLLRQHLRTETLEQLKLFSTQLTVNRIEFSAFGFFTLNLSTLSTFLVSVIT

YIVVLEQIK

>ZnGR68

MDNISDIRHELKHLYYLSKVLCLAPFSFSWNPVAREIFINTSLSSNVTGFIWTLFMSIIM

VTGLISGIIRYKHVGHSNPGVALNNSFCFPMNFINALLSFVVLFMNRFKFGELVQKLSEI

DDKLCQIKLRRCTKYQNYFLYFAYTVCALLLMFMCYDILEVEERRYFVYSIVYRIAYFIS

LVLIIQFCHIVRCIEERLIVLERGVSSVLENRTPIGVTSTLTLPSVETVDKSELLSITSL

ISENENQVEPIENSLLFSRKALPHRKSYQLHIIITSRKIYSDIYDATQLINSVYGFILLL

LFIRAAAGLVTNIYHLASITISGYVFVEYKNWGTAGHISSLVTWIVIFLSTITMMTVTCQ

MTILKSKKIGDIIQKLILQQPLRSDMLQQLKLFSDQVAKSEIKFSAFWFFNVDMSLLYTM

LTSVTTYIIVLMQAK

>ZnGR69

MMFKEANILSAMQPLYFISKLCGLTPYSYVELLNNTYVIKGPSAPITIWTLTVTIFVVAG

YIHNLIYLFQQTKYHTASSSVVIIFEKTWLFAFSVSILLLGLTRNRLKVQNILQYILEIE

QNILKNTAGNIYKKTFCSLSFQVLFVSGFHVCMLYSYIKIHNGKDNFACQFSYTIASFVN

TIAVIQFLFLLEILRYSCKNIGEELEHSEINYLKINNVFIVRNIHDIPQPRQQSNKLHHL

RSLHFKLYELAELINSCYGFLMLIETTHNFVMVVGLMRDILTALEEKSDTNLVIRNICLL

IFYSAKETMILRTSQVTKNKSKEILVIVHKLLLHPNVDQECETQLRLFAGQIVDNKFQFT

ACGIFPLDLSTLHSIVAAAATYIIILYQMKNA

>ZnGR71

MRCLKIWKRERHEMDFYCVIKPLYYVSKVTGVAQFSYIQTNLKSLNGKVLKINDLKPSGM

LWTLFVFLVLASGLVSVMMWNFLYDYTNYSFNVIITDTFSILLLYGSSFSCLIIVGIVHR

KDIIQILKKFSLLDTVLLRNHRQRRYKKENNFIIVELSVCVVVLFLSYGYHVSSWATGIA

IIPFASKDLAHFAGTIILTQYVDVVLLLRSRFKKLNEELLLQCADSENNLCRLEVRDTYQ

RCNRIAFLEDFSALSPWKQDCKVECYKSSSKCKLLPPGSHRRDREVIRQFRVLHSELMDI

CDLVNSCYGFSILLLFAYYFTSLLSSVFYVMQKVVSLDITSETDPDVIRGLVSSSFWALV

YFLKVVVTTVVCTEASAAARNTGRIVHSILLQDVSQNVITELKLFSLQLLGNSSELFTAF

KFFEINLTFLYNFVGSTAMYLIILIQLK

>ZnGR72

MEHISPVEDVHIQLKPLLTISRIFGLTHFSLTRTRKGEGLAMNFNFLDVVLVCMWISAYF

TLACFSVYELMGKTPLPGKITILLIINIISIHSTTIVTLITSASLNRRKLPRALSKLIEV

DRMVGHRDVTNSTRTKLKTMIHIFILLLSQVFIYAVNYYIKCEGTLASVIYVTFQNLGIT

FNILITIVYADLIRTLKLRYKYMIDDLEEYFRVKDNAVIVNFRDQHVTFSSGIIRCKQHP

LFSITRRNVSSDLREIRKIHTLRFIYIELYDSIMLLNSYFGIPILFEMLSVMLTCVTAMY

VGSYCLSVGHNFFKTRILGYYLMSFGILFLSTFAWLNICCHALIEEANRGIISIQRITAF

SNVNCETVRELDRLSNQMNNMKVQFTACGFISLGLPIICTVIGGILTYILIMVQIA

>ZnGR73

MENTEDVTDMYSSIKPLLCVSKLFGLTSFSFPKQNNYKYKVMQICLLFMWVTVLLSTTCC

NVYTFLYSVSDVPEKIKVTFIFNNLSMTLTNIITLITNNCCNKSHTINIFKKIKNFDKIL

DRRSRNKIYKTTKLSVLRRIIVVFVLILLSYSGSYYIYYDGKLVSILQALVDNISYTLNI

VVVLQYITLVRMLLHRYKFMIEKITKYSETEDSARIIYGKTHSRNSSVNIFCGNNFMAST

DVRPYVNIENYGIHILRLSYIRLYETVALINSYFGIQILLQIISLVIVCVTAFYYGLYIF

CSINTDIADFTLYFKPCLLIFWTFLYATLFAWLIVCCHQTELEGHRGIICIQIITSHPNI

KHGKLMNLNNLSNQLKDMKVEFTACGIFALNFSLLCTVIGGILTYILIMVQLQ

>ZnGR74

MAKDAYISSRIYRYLRPLYFVCKICGLLPWYLQEDCIKRDRVSVHRIFNIAYCMLWVIVF

ILGLSVRILNQSYQNRSKLALKIQIFYKMHFISQYITSIVCSFHLVIISNMKFIRLLEKL

SQVDYKIFSPDEEQRVNRKTSIIVISELVIILLLILSYETYNSSVHPNWDVIILISVITN

IAAFITNTLTILLFTSFVVMVTRRHGHLNKLLQNVFRGHNHVNRLGYCGHVQRVSKCNAT

VPHVHSTGFKRINYVSTSQDIRSIRILYNELYDMVSVINFQYGFPILVMACWVLIELVLT

VYSFLCCFTINTLKVAFYYIIFFFLFVKMATVCQFAENERQTSINLVHKLLLEDNIDEID

CKELGLLASQLRDMKIKYTACGFFTLNLPYLCSVTSLTVSYIIIMIQLQLV

>ZnGR75

MTENTAGSGQIYVALKPLTFICALFGLFPCSFGRNRADRTEPTVCVFLNIVCSLVWICLF

AVRLCLKLLHLSSDNVTKVRILTEMSVISTHFTSIICLVHLSIVCKNKFIRLLEKVSQAD

YGLFTLSEEKYVNRRTNFIIITELAVMLLLFISYEISYEIYTAQTRSKREVLDYITQVIT

LSSYICNTLTVLLINNCVLIVTQRQRHLNKQLTTFISCPNRVNKISYCGHIKPINKRNAT

TPYVSLIRVETSNHVSGNQEIHSIRILYNQLYDVACIVNYLYGLPLLAMSCWILLDLVLI

TYVAVTSLDVDFIITNSKDIIFFLIFLKMIIMCQISENEIQSSIILVQKVMLEDDLGYKD

CKELLMLSAQLRDMKIKYTACKFFTLNLPYLCSVVGAVVSYVIIMIQIE

>ZnGR78

MKNHELKYNIYFEIELLCFISRLLALAPFSIQKANSAARGKSFQYSDIILTTFQTILILV

GFCISELFVAVIDEPVLLSTIRTMWFISSVSYHTTCIVTLILNVTIHRQYLPRILNSINV

IDSKLCLRDNREKLFRTRRSSRTKQLSLITLILGINSALSFCFSFNRGIVYNAYTILRTL

CNVIFFLIIFQYITLVLVLKTRYEQIYSTLSKLLFTDGTSHDVSVKFNSRRRISILAFNL

DVKSRTEDVFMIRDLRQIYSQLHDVLLLINKYYGTTILLVIISILLNLTPSIYLGIILFK

DTIVYRHELKQYIESVLLLCWCVSIIFMYVWLNSCCHLVTDEVYKLMVCIHRIQLLPNVT

HGTVVELNAFTNQLRDIRVEFSVCGLFALNLQFLCASFGVITSYILVLIQL

>ZnGR80

MMKEPFEQDVHFVIQPLHFVSKIFGLCPFHIDPKFRYDNERLCNVLHNLLATFMIVMFFF

GLYSTMIYATEFNDPTFNIFVRVVWVINIVAAYSTSIVGLIFSVTRNKNHMTAVLASICR

VDKKLVHRHSKQNIYTSQRSHVMLQLKVTLVTLVTVYSFSAYLFYNGTWTSLMYMISQML

SAVINNVKTLQYVNVILIVKQRYQLMKHLLLKEVLADSCSRNPGPFKVHNANKIFLTTDN

LKNYKDSSNVCRIRDLRMVHSELYDVIHENNNSYGILILLEFITVLTNSVGTTYFGVMTI

NDTVFNNGNFEIYFKGITLLSMCTFELLAFFWLTTCCHSTAEEFNATLIHIQKLLLYPYI

STWTIVDLKSFSSQLKNVKIEFNICGFFNLNLHFFCASVGGMFTYIIVLSQFNQKV

>ZnGR81NTE

DVHFVIQPLHFVSKIFGLCPFHIDPKFRYDNERLCNVLHNLLATFMIVMFFFGLYSTMIY

ATEFNDPTFNIFVRVVWVINIVAAYSTSIVGLIFSVTRNKNHMTAVLASICRVDKKLVHR

HSKQNIYTSQRSHVMLQLKVTLVTLVTVYSFSAYLFYNGTWTSLMYMISQMLSAVINNVK

TLQYVNVILIVKQRYQLMKHLLLKEVLAHSCSRNPGPFKVHNANKIFLTTDNLKNYKDSS

NVCRIRDLRMVHSELYDVIHENNNSYGILILLEFITVLTNSVGTTYFGVMTINDTVFNNG

NFEIYFKGITLLSMCTFELLAFFWLTTCCHSTAEEFNATLIHIQKLLLYPYISTWTIVDL

KSFSSQLKNVKIEFNICGFFNLNLHFFSASVGVMFTYIIVLSQFNQKV

>ZnGR82

MKEEPFEHDVHFVIQPLHFVSKIFGMCPFHIDPKYRYDNERLCNVLHNMLATFMIVMLLF

GLYSNMVYATELIDPTFNIIVRVVWVISVVTAYSSSILGLIFSITRNKNHMTAVLASICR

VDKKLLHRHNKQNIYTSQRSHVMMQLKVMFILLGIVFSSCAYSFYNGTWTCLMFMISQIL

SVAINIVNTLQYVNVILIVKQRYQLMKHLLLKEVLSDSCSRNPGPFKSHNANKLFLTTDN

LKNYKDSSNVCRIRDLRMVHSELYDVIHENNNSYGIVILLEFITVLTYSVFATYFGVITM

NDAVFNNGNFEIYFKGITLLSMCAFELLAFFWLTTCCNSTAEEFNATLIHIQKLLLYPYI

STWTIVDLKSFSSQLKNVKIEFNICGFFNLNLHFFCASVGGMFTYIIVLSQFNQKV

>ZnGR83

MPYTNIYSATKPLYYVSSLFGLAPVSWCSGKKHGFIRDRVRILKLFWTVLLTLTLFPSMY

YNISLSVPKSNNVTRIIGFAIFHVPLYLSCIITLFVGLTVNRSKLPQLLYKIMQVDRVLI

ITSTRLCMYRKTKVHIFLQLLVVFVIVVALWLFDMSSYCGLHCYHAYLGIVPFLVNTVEI

IQFLNFVMILRTKYKLLNAYLTSLLPALKINENTVPSSNFHTTFINFVPNEVLEVKSSCS

SSLLNDMTQSEVKIHHLREIYSRLYDISLIISSIYGISLLGVIVWLFTYSIACIVFALQH

FSNGRIPIANILLLLLSQCLLAAIAVPCHVTTDEASRSSVLIQKLLLRRDINKRFISHLD

RFYTQINSMTIKFTACGFFSLDMSLFCGIIGAICTYVFVITQLK

>ZnGR85

MEAISLTSIIKPLYYLCKVFGLASFCLCNNCGKKRYTFLHSGAWLSLSWALIYAVNSCLV

INFIVQDSDIPDNIAIASVLYYLSLHFTSIIYLCLCNIFRRRNLPLIIDKIEELCNIFTM

KVDTNLVYKRMKWFVIFETTVLLLINGTVNAIYLYSEYDSSILETFWLIVEVIGSLCNSL

IIIQFVSIVMLLKYILKCINHELGYCYDVLKDSCRYSIIRIQPGGLKTFRSPGTKLISSL

CNKVHGLRITYSRLSAVISLVNSYYGFTILMLVSWLFITIVTVLYDALLLFFDSEHTDNL

IEYYYDVFDALSLCIYSVILMAVITTSCHLISDETEDTIFHIQNLLLCRDIGKETEKELT

IFCSQVKYSKLELTACDLFKLNLPFMKSFISACFAYFVIVFQLK

>ZnGR86

MKILAENTSLISAIKPLHYVSSVFGLAYFSVLEGKQHRCKSTIPWSLIWTCMWIILYSAS

SYLQLSSNSNSGNTTKITVLNTVQATSLNVTCIISLCMCSVFRRHQIFQIINKLELMTNT

FTKTVNVTTTYRKTKILVLLEIIFVLLVNGILAAIYIGLYNSRDLTIWTGLSLVIELLGC

VCVSLIIIQVATVVLVLRDKCKCINRFLMRASEDDLKHPMTMFQTCKAVEFLYVDTYILP

SQLHRNRLSRNQVFEcRLILAELKSVSRLICSFYGFPILLLSFWMFINIVTVLHSIFYYQ

STFYDNDDYSGLINSFESMLWCVYCAMLMTLMTLSCHLASEEPNITMSHVQNLLLYQNLG

KETIEELIKFYSQLSETNIEITASGFFVLNLQLLYEFFGTTFAYFVIMVQVN

>ZnGR87

MDQDYLCFMMKPVNYLCRLLGIASYSISDIKNYDHKTGNKFWKLLWPYLLAIVLMCCYVY

RILFTFQVESQVVHHNLLVTDFLNTSIEYIGCVILIIIRSINHQRNISLILKKFTLLNER

FFKYRNEFIAQNVISPVIQVTLSLLLIFFFILSCVNVFVWSTHWLPWLIICEEWCAFTIF

VINVKFVLLVQCYRYRHKELNCQIAMLNDLTGPTVRTVVLQDVRETLILEYSSNCRVSYQ

PSEAVVRSTDTSRKTLFLNRVKALREDHLDLFELSQLLNSKYGFQILICFSLIFFQHILN

YNFVIDLMLKLLSGKDGIATDVQEYASLCMAILSSVALIFLTVSCHMASVEANSSQLLVH

KLLLSKDLSSDVTVQLQLFSSQVSNLKVKFKICGLFTINASLLSTIAGVICTYLIILYQF

K

>ZnevGR88

MKIVRVKPIENPKQIHADEHTFYRDVRPMALVCKVLGIFSLQNILQGDGRRLKHKFLSIDAI

WGPIFIGALPIPFIEYGNDDFGRYWRIMQCLRGLITVSLSSYYDASLPEMISKMETLGVVLR

SMRSKTTERNINRHGRISGYVGVAGSVLFVSMNTFVEYVIKRVSFPRLMIHLYGTLNLIPRQ

VYVVMYIFFCYNIILLFRDVQSCWKEHVNRTDEREKQLENLRLVHVDLLRIIRLLNQAYGS

RLAFYLATIFIEVLLDLYIFFFYNNYGYIQMEYYVFNAVTFYMLTSVTDELSHVGDYIALDL

MKIRMTKLNSGCRNQVLMFLRQLIANRVQVSAAGFCAINKGIIVSILIAVVTYFVVIYQQAS

GRN

>ZnevGR89

MAFVIEITAHRTSLTNRNESLKEEIARENKTFYRGLKPFALICKLIGVFSLQNVLQDNGCLL

KHTLFSFHTLWGPFFVGMATISNTYFETEHFASYIAMFYIIRGISISLLTSYYDKFLPEIIFRI

EEFNAIIKSNCNKTHTRRNLNTHGRLVLYIACVGYIILMCANVAVLSLINGYNFPRVVNKIC

DSFTFLTRQMFVMMYIYFCYNIKLILCSISSIWRKTVKTIVINKNVDPVPPEERLESVRLLH

AEAVQTVELINSAYGVRLLFYVTIYCSEVLLSLYEFSNRHVHLKLYFIIYSGFTLYMVTKFTE

DITTQGNVMADDVTDIPIALLNNECQQQAEMFLSQIMVKKLQVSASGLCIINKRLIISIVMG

ITTFFIAIYQLSQET

>ZnevGR90

MFIDVSSNNSVLTCTGPFKCSKFGVGLACGHLAFLLWTTYTTLVDPNANPWNLLDSLLAG

IFVIRATVTQSRSVICLNQLYTASSRFQFSSLAFFCFAVCQSFVLTFTGIKSFRSCLENVFLLLI

QFSVESIIGAMERAFELQFHSINEELETIASQKTQLKSGNFRGIRNQEESKLQRANSGHILR

KLCSKLIRMFQNAFRRQGFNRRKVTFRKTPRKLLNSDRAFRPLPWVVQASRITQHPQTKY

ERNIFARFEIPGKNILGHSYTCDLLEEIRLAHGHLSDIAHRVNCHHGPEVLMTTTVNFCKV

VLILSDFVWEIILQGSATASLITSTTISFMLQLSRILYICYRGEKVCCETDRTKDLLLKLSDST

LEPNIKQEVSIFIKQLHAKKIQFSACGFFCWNKGLILPMVGAVTTYSAMLIQFQSTGFSFK

>ZnevGR91

MFKNLVFTSNQMKVDLKREYPIMKINEDSEYKNIIHKSFRPAYLFSKALGVMPLSYKIRSS

AKVTEARNRKMNSMEFEWSWKGAIYSGLWIALLITIRYFIFITRRSPPPRVERDSDHSNST

FENFSNWSNSHPPPFPGGREHLIGSMNELLDFTCTLLALIIGVVGARKIPEIFRQLQDLDEN

ADEDGHMLLDSAERRCLSRYGRMFSVFSVIACCSYTLTGTFAASYSAISVGVPMEIILPSMF

GDLMKLGNAAGEAQFINFCFFLRQRFSVLNDNLTKLQMDTRKTKLDHWFNDSSQKAPYG

PPNHLPESLASRAHPLWGRVTVANPRVISMTKDEVADTLGRLCRWHRRLCDLVDMVASC

YELPLFVVIAYCFANSVFGVYLVITFRGPDSLLRITGVIWSVAYGCRLILIAVIPSVTVAQAKK

SRMLVERLNNRYLDDASKQEILIFISHLSSRNISFNVCGFFTLNIPFLRSIAVAIVTYVILLMQ

FKFPEN

>ZnevGR92P

MYKSKNSVGDPQCPWYHEYTSAKPLLRVSQVVGLAPVAWPNLRPVTYGIVHTCVMFIAL

FGWFIYATALTILQEYPLKEATYIVPDFCNSASLYLSSIVSLALCATANRYRPQTIMRLVAQ

ADNAVNAVCPSSVHDKTRILVTAELVVSVSGLTLLAVYDNVVWTGKLWNFHNYTGRYYA

HLINLLVVLQFSSLTLLLKQRFARINKLLKSTVITSNDSDAWSQRSILEDAAPSQLVFKSRA

RGFAPPVGVWRDQQVLALRKAHSALCEVASTVNDMYGVQILLVITSDFIGSVWPLYLLLV

TNFNSEETHTTHRTVAWSLLFLLZRCLKILLVVVSCHMAKIEGRRTGCVVHQVLLHGEDP

RVTGGCSRLLQLQMFSQQLVHTREQIDFTACGLFPLDMTLLHSVVGAVTTYIIILMQFQTK

# Supplementary Data S3. IRs

>GjilIR569

MHLPEADSGRLVVGTWWLVVLVIVTSYCGNLVAFLTFPKIEEAVTSVEDL

LKNRRYYTWGLLEGSALQQHLLVSLGHKTFPVNSLRFMPERGPSQLGCRS

GLREPSIALAACELQIQPLVLKFHQISFYPFTTK

>GjilIR76b

MAGVSEFIGMLLLTVCTNYQDENGTLAVGGAGDPGCILKEPRALEGVTIR

IATLEDFPLSHTELDDGSGDRKGMGIVFEFVDILAEIFGFNYEVIVPEKN

VLGDENEGIFGLLKQQKADMAAAFLPVLESERHLVNYSVSLAQADWVILM

RRPSESATGSGLLAPFDNTVWILILVSLILVGPTIHIIILIRVKLCKGSE

KLTKIFPLHQCIWFVYGALMKQGSTLSPISDSSRLLFATWWIFITILTSF

YTANLTAFLTLSRFTLPIDDARGLAKLKYKWMAQAGGAIEKLLQTNPDFE

YLNNSLRDGKGRFVDLKNEEMLKEISDTGHMYINEYQAAEYTMFRDYLAK

TKNNVDEGKRCTFVATPQPFMVTSLAFAFRNNSELKNLIDPILTSLVETG

IVKHLLKRDLPQTEICPLNLGSKERQLRNSDLLMTYMIVVGGFVVAAAVF

LSEMIFRLLLGEGRPKLTGWSNPPSDEATHSKAFNIFGKKPVFAVAGHPP

PYNEAWYTGGGAMRQDLRKRQVINGRDYFVVNAKDGDTTLIPVRTPSAFL

FQYTS

>GjilIR68a

MNFKPQVYEAINADVELWGRKQLNGSFSGLLGEVVTGHADVALGNLHYTP

YHLSLMDLTIPYNTECLTFLTPESLTDNSWKTLILPFKPYMWAAVLVALF

LAGFVFYALAHFHQSIHEPTLIIKQQDITDMSKNITRNVIEVETNEGLYL

FSDLTNGILYTYSMLLLVSLPKLPSGWPLRLLTGWWWIYCVLVVVAYRAS

MTAILANPAPRVTIDTLDQLADNHISCGGWGEQVKEFFLTSLDSASQKIG

QKFEVVYDVSAAIDRVAKGEFAYYENIYFLQAASIKREILSPDEKEKHKN

NPSEERSLHIMHDCAINMPISIGLQRNSPLKPRVDQFLRRVIEAGLVQKW

LNDVMLATRSIEILDEDDTSKALMDLRKLYGGLVALGVGYFSSFCMLIIE

KIYWKYVVMKNPLFDKYAKDVYYKTPMCYKK

>GjilIR411

MFLDSPTDVIVNCYSTWFHCFFVAFGLLTFQPPATLPVRRNNVPMRHLVS

WLLVFYLLVATCYSAGLALILTVPRFERPIDTAEDFAASNILWATNWQES

TIALEDFPEDHFKTIVKNFRVFEQATLAAEMLLGHYATVVEKLSTGNMAL

EPQVTEEIMPLYRMMKGELYSGNIYIGMRKSSPYAEQLNRLIHAMTECGL

FSRWEGQAVRDYMSQRLQVAIKQSVHMVADNEPTTLKLEHVQGAFILLAL

GLVAALVVFISEVNGILPKCCRPKNSSSLKNYPFLK

>GjilIR20

MCVALMLGTGQDVKSRSGPCKFSLVVAYLVGVVLLNAYSGAYIALLATHE

YKMPFTTLQGLLDNGQYELGTNGDGAVLSMFQNAERGVMKELYAKHMSPR

MEDYPHNTLDGLRRICSSDKYAFITKYDTAVILTSSMNCPITVLPEIFSV

SAFAFAFQKQSPYIKLFDHKYEEHLVHCELP

>GjilIR29

MKLMLQELSETRGLSGPLWVVALPFNSLETFFGDILIPFDCMMFVVGTAD

GGEVPIIEVFQPHRGAPLQTVDFGRWSARGGLVVIDGNFYSRRSDCYGAV

IRAITADDGVFVSFKPEENGTSKLGGILGEPWAEMAYLLNFTTVFIPIST

MPRGQLLENGTWTGLIGMLLAGEADVALGFFPVTSDIGAVLDFTLPLATL

TLQVFIKEPRNDKVQWNYLLHPFSPTLWLTVAVAILVLAVHLTALHRLGR

HYGNPEADGPPQYLLYDSLLYVLGAFCQQNIEERVDATTLRQDVCGS

>GjilIR252

MERAVAYGSQTLHKPERNYAQLDKEALDIMFGITKFRHISMGGNLLYIQT

ISRYWGC

>GjilIR246

MNILKTISRRMSAFYKLKPIKLGCEVRGIDLKTENRPKVIKQIKEDVTIH

RLLIFKDQGIIDGKRHVEISKWFGDLESTFYKHEKSPHPDVFIVSNDPTE

GCTGVGRTGWHIDGSFQSAPFAYSLYHMVSVPKRGNTSFAPLAEIIEALP

PKKRAKWDRLWMLSDRRTGPIHPLIYRHPITKINVMCFHLGMTAGYIWDR

NTAKERHATEEEFLEINADIGHEFVKNYGKIQYSHKWEKGDFIITDNLAV

GHEASPETQQPREIVGLRVLHRTTVKGTHPPQK

>GjilIR8a

MTSEREENGADIWDGYCIDLLKMLATNMNFDYEIVPPANGAFGSRHADGT

WDGMVGDLAMGETDMVIAPLTMTSEREEVIDFVAPYFDQSGISIVIRKPV

RKTSLFKFMTVLRLEVWLSIVGALTVTGIMIWILDKYSPYSAQNNKTMYP

YPCREFTLKESFWFALTSFTPQGGGEAPKALSGRTLVAAYWLFVVLMLAT

FTANLAAFLTVERMKSPVASLEQLARQSRINYTVVANSDTHEYFINMKNA

EDVLYGVWKDITLNSSSDQSKYRVWDYPIKEQYGHILQAINQTGPVVNDT

IGFKQVVDEEEGKFAFIHDAARIRYEVSRNCNLTEVGEMFAEQPYAIAVQ

QGSHLQEEISRKILDLQKDRYFETLSGKYWNSSAKGDCPNTDDSEGITLE

SLGGVFIATLFGLALAMITLAGEIFYYKRKKFNVVSGTPGIDAAPKKQIT

IGNEFRPVIDKTMPRVSYISVFPRNQLY

>LmigIR8a

MWPLWMSVVAAHLQLASSQATSEPLLIRFLLVTEVNASWVGGELRANLSGLEARYVGLRLQLDLSAVEVD

REHEVEEFQQKVCGALASGVSALLDATWTGWRRLRDEAQHRGLPYLRLDATLANFVDAVDKYLHAREASD

AALIFHTEEELDQALYHLIGNSVLRVIALNGLEKDTVSRLKDMRPVASYFVIFADTAHMAELYSKAAAGG

LVRKAERWALAFTDWDWRSFRTDQLNLSTALLQMKPASCCALQGEPDTCKCALRKVAPAFLRAALSAAVD

TLAELHSKGMDVRATPRQCSSTGDGGADDEDGDGAGEEEESAEPPANATGNYDAFLRAIATRGQSNSTLF

FRAATAQLTFNTPLQLRMVNRSEDVSLGDWSPEKGLQLERQLKPAKRFFRVGTAEGVPWSFPVRDEKTGE

PLVGPDGEPIWDGYCIDLLKKLAEPTHMNFDYELVPAKDNDFGSRSPSGSWTGLVGDLAMGETDMIIAPL

TMTSEREEMIDFVAPYFDQSGISIVIRKPVRETSLFKFMTVLRLEVWLSIVGALTVTGIMIWLLDKYSPY

SAQNNKEMYPYPCREFTLKESFWFALTSFTPQGGGEAPKALSGRTLVAAYWLFVVLMLATFTANLAAFLT

VERMKSPVQSLEQLARQSRINYTVVLNSDTHEYFRNMKNAEDVLYNVWKDITLNSSSDQSRYRVWDYPIK

EQYGHILQAIDQAGPVPNASVGFQKVIDQEEGKFAFIHDAAQIRYEVSKNCNLTEVGEMFAEQPYAIAVQ

QGSHLQEEISRQILDLQKDRYFESLTAKFWNNSAKGTCPNSDDSEGITLESLGGVFIATLFGLALAMITL

AGEIFYYKRKKLTAVNVTSSSAKVPKKQVTIGKEFRPVMDKTAPRVSYISVFPRNQLY

>LmigIR25a

MEAGVTWVPDLQAETPEGVPAAAQGAPSAEKVHVNDDKNSIPNKALKQVVADLSKQGMIFDGVFKATANG

SDVEALIDSMCLEYNTSIDENKKIHVVLDTTLQDVSSEAVKYFTRALELPTVSASCGQEGDLRRRLSWWE

NMYKKNLSTICLPRCVCVCICVGVGVFAYLPFSPQVIKTLLFSLYAVMDHKYKSLLQNVPTRHIINEIKF

QNIANQLSTFKQREVFNYFILGRMDTVNKVLEAAADMEFYGRQFGWYAITQDEGNPSCQKCGKGASVLHV

KPNDAEGTVVGAENPKLAYQFYYELFRNTFLAIGQMIKEESWPDMQYIPCEEYEENKNVPPTRKLNLLEA

LQQISMLNPGAYGQLMLSSNGHSHMQFNMTAFNVSLSDNSATEVGTWAADLDSPFITKVKPSVPVTQYTV

VVALQQPFVIKYQDENGNTKFKGYCIDLINAIRNITNFEIEIYEVADGKFGNMDEEGRWNGMIKDLIDKK

AHIALGALSVMAERENVVDFTVPYYDLVGITILMKKPKTPTSLFKFLTVLENDVWLCILAAYFFTSFLMW

VFDRWSPYSYQNNREKYKDDEEKREFDLKECLWFCMTSLTPQGGGEAPKNLSGRLVAATWWLFGFIIIAS

YTANLAAFLTVSRLDTPVESLDDLSKQYKIQYAPIANSSAHGGGEAPKNLSGRLVAATWWLFGFIIIASY

TANLAAFLTVSRLDTPVESLDDLSKQYKIQYAPIANSSAHVYFQRMAAIENRFYEIWKDMSLNDSLSEVE

RAKLAVWDYPVSDKYTKILQAMTEAGFPANIEEALERVRASKSSSEGFAFIGDATDIRYQVLTNCDLQMV

GEEFSRKPYAIAVQQGSPLKDQFNNAILQLLNKRKLEKLKEQWWNQNPEKRNDCEKQDDQSDGISIQNIG

GVFIVIFVGIGLACITLAFEYWWYKLRPQHNAVVEAAPPRTKSDSLQALNMMRSSFDKRYGRRQGVALAG

VTNPW

>LmigIR20

MPSLCKASVIMTVLTLCLTSTIFCESQKSYGNSINFILDFIRHTTTASSIKGVHAFVCWDAGDLQLLKAL

SRNGMLASVYSGWQHWEQLPTFHMDGNSLLFILDLKCEKSFAFLKKTGRNVEFFKPPHMWLILHDAYHPD

TQLFTDHSAETMLPAAKNNGKNVLKQQHNVSSYNNSIILDAMELGDEDFSSADVYDLSYGTSPKRRAGYT

NSNSTHVTHVPSGVGMKNSEFTSNHINDNSAVVEAAETGFYCEVFRGLDILVDSQVTVGRRDADSKYTLL

EAYRRRRQGELVVSELGYWEATAGIVWRNVREVSVRRLDLKRTKLVASIVVTNPETLDHLDDIHNRHIDT

VTKLNYVLLLHVADVLNASLELLVTDEWGYESNGSWSGLVGSLQRAEADVGGTALFVTADRMRLIDYIAL

TTPSVAAFVFRQPPLSLVSNLFTLPYTVRQS

>LmigIR28

LEALYRVHPSRPLTVRQAGSWTPHQGIVLHRSAVHNGWRLLDGLILGAAGVVVDTPTDRLGERLRVVQDR

HLDTNIRFGWDLCDKLRFMFNFSVVLYKTESYGYLTPNGTIDGQAGMLCDGTIDLALSQLMLSHHRLDFI

DFTTAPSRMWTLKTVFRHPASRAVYGTIFRPFSAALWLSSGLVFLLVLVAARLGCWAAAHNPADDSWSAA

FLLVSSAISQQGTTLDTGRPSWRLVVFLSFSCALLLDTYYTAAIVTSLLLPPPRTINSKADLVHSQLAVG

MENISYTHEYFEKSSDPVDHALMKYKVWPAGTARPNYHSLEAGVRKVAAEAFAFTGEDVSLYPLLDRYVT

EADKCALVALDFMKSRSTYMPVRKNSPYRELLTIGLRRLLERGHLAR

>LmigIR25

LHNGTYGGMIELMQRNLSQVSGSSLIMKKNRWDVVDYTGPAWRFKSYLAFRHPTSQGLRSSLFAPFCSSV

WVASGAVWLLIMSSLRLITWIGARQAGGIVQAESSWGAVVVFAACAIGLQGTTERSQWLSWRVVLLFTFL

LSLMLNTYYGAAVVGSLLVPPPKTIRTLRHLIDSPIQVGIEDIGYNRDYLEKSTDPLVRELFVRKVFPPS

AKRPHYYPIDVGVERMRTSLFAFQAEAV

>LmigIR27

GTDLLCADHCRNHGFRRSLGGLILRAGTVVHDLPLVNLGHRLTNVEEKHVDTMARFGWELTTILSKKLNF

TPVLYATDSFGYKQDTGNDTLDGLVGMLETGLVDVGSAGLAMFKDRLNRIDFVGPSRLWVSKIMFRHPTV

AAEQGALFRPFTPGLWLGVMLVFAALALVSHAICRAHGWSAQRAWGDTFLLVASAVGQQGSSEGVQWPSW

RLLLMVSLVCATLLDVHYSAAIVSSLLLPPPRTINTREDLLRSPLHFGIENISYAHDLIEMSEDPVIRSL

YQKKVAPPGAVRPNYFTLEEGMTKVATEMFAFHSQEFQMYQLVEKLFTEQDKCVLVTIPLFPPQMTYVTV

AKNSPLRETFTVGMRAMWEQGHLRHLRMRWHSKKPACMAERDFVNVDLATISPAFMLLLSATLISLLLLL

RESAKARQTQDNTPVPLSH

>LmigIR11

MSACEVTIGADRIGVLTFAQPVRNAGCRFYFQTQFQEVMFKPYVQPFEDRVWIALLGYVVATALLKWLFH

YVLRERADPITALFAVIAALLNQASGDPRRCAVRVLYFSLCALFLILRIGYGARLTATSTLRQAAPPYRR

MEDILASDWGVNVISNSFALESMQMSPPNSTAWKLWQTKIDNNPYSTVETTEDGLWNVLDKKKLAFFGFE

DACRDVLHRRFSPDQSCRISELDGVFFKAPLSFALPRDSEFLLTINYWILRMFETGIIDRLSRKWLPKPP

VCDETKYEAETFADVLPMVILFASGFCLAIIVLIGEICVYSFKNRNMKVVSKNRKVRKTQKNYLYPML

>LmigIR26

MLGGKAGSVDAGATNLRQNVSRVPTCKRSCCPAADDDVAEALMFEIITITITQHAEYLITVTSTQTDNLH

ERLISENQKHLDPLARFNYALFLHLKEFYNFTFSVQATRSWGYKTKAGRFDGMMGVIQRNEADIGASSAL

IKKERLEIVDYAGHTWKFWPRFLFLHPSGQRLHTALLTPLSTKVWFCAILAGLMITLILNTSSYVHADNF

CNLDGSWSSTLITTIGTFTLQGAGSSWSQISWRITLLTALLLASLLNIHYGAAVVGSLLIPAPHTIRTLQ

DLMESPLRVAFENVSYNREYVARTTDKLGRELIHRKKPEFVQLSEGVIKIRKGFFAFHTEGGSVFRLAAM

TFTESQKCALSDVSLFTPAVMSMPVKKKSPLRELFARGWSTRRPNCDFLRQSLPMDGPMVEVKKIDSTNP

WLKL

>LmigIR2

VSSIFALRSIEIIPDILVARQLDDVDSFELVTMKFTGEETWRDELVLARWRRGHGFDPPTVDLFPDRMAD

MEGRQLVFATIDYPPYVILKSDSNILDGVESRILLEKLFVVGWQKNATWRVVDHAVDRWGTIWENGSGNG

LLGAVATGAADAGFAAVYRWFPEFLWVDYSRPFLRAGIACLAPRPPEVPLLPFAPPLWAAVAASVLLATI

ALYTARITSNWVLGDVQATGRYSTVEDCFFRSVGLLVLQTPDLERRHTRVVGPTRHVLSWLLIAYLLVTA

SYGSGLSSVLTVPRYDRPIDTVSDLHESGLEWAERHLVYLYSIRELTDQIYVDLIDLFRVLTSDVLHSRT

TTRDLAFVIERLPGGYFTIGDYIDEEAASRWLRPMREDIYWEYVVFAVPKGWAYLQRLDDLIDRLLQAGI

MYAWEGQVAHKWLVPRVQLAAQIGMRSYAQSPDGPIQLQLTHVQGEFALLGLGLCLALVVLLVEVAV

>LmigIR1

NASWRFTVDAEHEWGEIWDNNGSGNGILGAVVRDAADAGFGALYQWLHEYLFLDFSRPYIRTGITCLAPR

PRPGWQVPLLRPSPPLWAAVTASVLLATVALFAARRSSDRLLGEDAVRAAAGSRYSTVEDCFFRSVGLLV

LQTPDVERRHTRVVGPTRHVLSWLLIAYLLVTASYGSGLSSVLTVPKYEPPIDSVRDLYESGIEWAATHE

AWVFSLREATQPVISDLYRRFRVHKEDVLHARTVARDLAYSIERLPAGYFAIGSYIDEASRWLRPMREDI

YWERSNVVVRKGWPHLPRLDALIDRLLDSGLLLAWEGQV

>LmigIR24

MKPPAALLVLLLVCQESSGQDSYFTDVISLTRDYFVAKRVSLVTVYTCWNSWLEKDLLRSLWDRGLRASR

LPLAEAAETSCGGSHGAALASLEAAGPYRSGVLVDVACPGGQQLLTKASARRMFGIQRHWLLVDSSGNST

AGDEPEFPALPARWRDVLSSLWMMPDSEVVWMGAADDGAIQLLDVYRLTSFTPVLNISLAGWAVRRNSGV

SLYLLPRPDTSKRRNSLHGAKLKAGAAILFPKYFTGMYDLRLPHLDTWTKITYPLIEYLGQNFNFTMEVF

YTDSYGWQTNGTFDGVIGMMQREEIQIAASSLFMRRDRMPYVDFAAEAFYLKTAVVFRQPTLASVANIFT

LPFSAAVWACCLLLCVLTLLLFGVQLRLAAKRGIEGELTHVTYAELFTFVLGSICQQGLPQTPTSLSGRV

TVFVLALTSLFLFTSYSANIVALLQSPSHSIRTVSDLASSPLTLGVQDIAYNKVYLGETTDRELRQFVRR

KMQPLGNRVFYNGAEGMERVRKGMFGFQVDTSTAYKIISETYTEREKCGLMEVNLFPLPPLCVATTKHAG

YREMFSQRVGWQREVGILTRQRRLWLPQRPVCENMVSGFVSVGIMDFYPALLVLQYGVAGAVVVLALELL

YFHRSRLWQRMICISQLSGAKPTSMYAVATDQISQKRSQLQRTGSRLK

>LmigIR3

FELVTMKFTGEETWRDELVLARWRRGHGFDPPTVDLFPDRMADMEGRQLVFATIDYPPYVILKSDSNILD

GVESRILLEFVSKKNATWRVVEHAVDRWGTIWENGSGNGLLGAVAAGAADAGFAAVYRWFPEFLWVDYSR

PFLRAGIACLAPRPPDIKGGFEPPTAYSLTLRIIAPQCTRPLPRAPCPPWSDMSVQYTISSSYSGRYSTV

EDCFFRSVGLLVLQTPDLERRHTRVVGPTRHVLSWLLIAYLLVTASYGSGLSSVLTVPRYDRPIDTVSDL

HESGLEWAERHLVYLYSIRELTDQIYVDLIDRFRVLTSDVLHSRTTTRDLAFVIERLPGGYFTIGDYIDE

EAASRWLRPMREDIYWEYVVFAVPKGWAYLQRLDDLIDRLLQAGIMYAWEGQVARKWLVPRVQLAAQIGM

RSYAQSPDGPIQLQLTHVQGEFALLGLGLCLALVALLVEVAV

>LmigIR21

LSLVSNLFTLPFSRAVWACAAALVVTCTVLLLAATRWERTRGDPQLADYLSQFYAVAGQLRDKWGEVAML

AVGAVCQQGSPAESRGVPGRIVTLSLLVTVMFLYTSYSASIVVLLQSTTSSIRTLADLLYSPLGLGVHDI

VYNRHFFPAADDPVRRALYRQKVAPPGAEPRFMTLEEGVRRMRTEPFAFHSELSPAWQLVQETFREDEKC

GLQAIPFLQLMHPYIAVQRGSAYKEMFKIAYRRLWESGLQHRQLSRLYTMRKPRCAAGRGSSFVSVGIAD

CYPALLVPVYGVAVAIIVVLAEILFHRRVEVLRW

>LmigIR76b

MQVSPVLRTVLTTVCSNYFLNGTKMEVPEGEPDPGCVLRIPKLMEGKTIRIGTLENPPLTMINKTDGTLI

GHGVIFEIVDILKHKLGFNYEVVTPRANILGDENHGIIGLVHSRQVDLAVGYLPQFSQQARLVRHSESLA

EAPWVFLMKRPLVSASGTGLLAPFDATVWYLVLASVVLMGPAIYFIILVRVRLCAGSERLTRIFPLSSCV

WFVYGALMKQGSTLMPVTDSSRMLFATWWIFITLLTSFYTANLTAFLTLSRFTLQITSLKDIATKKAHWA

AQKGSAMEYLVYNNDEYSFLNQSLQAGFGQFVDISDTDMLLRIKKDDLVYLREKQHVEHTMFRDYLEKTR

NPKVEEKDRCTFVMTKQPFLHLPISFYYPLNSNLAHIFDPLLKALVETGIVRHLLRKDLPQIEICPLDLG

SKERQLRNSDLYMTYMIVVTGFCAATVAFFGEILTRQVKRCIAEAELQTGPSTSYPDDWKTVKAQANRMP

YTMYLNGNIINVKQPAYSITKDFQSSNRKSLSRQRNTNYVFQYTS

>LmigIR4

EEDKWGAIWENGSGNGLLGAVAMDDADAGYAAVYHWYPEYHFVEYTRPYVRAGLTCMAPRPRPQPGWQRS

VDMLDNRDFVLVCYIITRCLLHVVPMNRVIGWIESSLDACMVLDTRLLTQRRCLLSSPDSVVLWGHRLQY

LHLHVGLYHIKYYSHLVTIWSSLGVTLSALFGGRLRNLLCLRRYEPPIDSVVDLRDSGLEWAETHIAFLN

SLRGRTEQVYLDLVKQYRVLSVDVLKSRTTTRDLAFIIERLPGGYFTIGDYIDEEAASRYLRPMREDIYW

EYVVLAVQKGWPYAQQLDELIDKLFEAGIMHAWEGQV

>LmigIR19

MCINKPPDGELSYKIHIYDIGKESDYKYYARDTVVRTDGNFQMDMNGNRELFDKSILQLLQDILEFSLLC

VEYHEIKIYLIKLGHIQYGRVRRLKVSNHNGKKYSRDLSILRQLATDKIDIGSTTILLTYDRIGMADYTI

GIDTFNSSYVFVKNPAAENSPVTGINASRKQFSLNMLEQPSWDLSEVMLVTIGALSQQGADRHPNTMAAR

ILFLVLFLLAVLMYTAYSASVISIMSSTKPVSNSLQGILESSSKMSVALHDIHYYHTQFQIERNPLSQNL

HKLELVPEFLSLEEGLEGALEGNIAFSCGRVDAHTYLQNSQHTDESLCGLGEIPILRGMGYQRSFALKHN

SPLRRAFDRGLLRLMEHGLIKREWHRYFGKRPASELHCEETSSSTGFVRITLDDVWPAVKMFGVGVIIAI

LLLPTELLVRQWLVLLEGNNLCENVLRSKMLTPIIK

>LmigIR17

MFFYLLIRDVATGGIREVYRFYVGHAVLYFNPYPLSKNSTQMDGSQLTVNEDGVINDVFPAGELWKFIAK

RLNISTQLIIKKDPSCGSYDHGQWSGMVGDVHEGKAHVGLSLFTMSASRASVVSFTTPIFLGRYFIFIRS

DEITHVGPNPLELLVPLKHDLWLAVIFTLFAYTTVMAGIFYIQRKTLCDAVFTIIGAFVQKSGKNMAADR

IIRFGPLDGITSYHQRITSEQCLRFAAYVTGSVLLSAYSAGIISILTITEPKLPFQNFAEIEEDGSYMLG

VTDNSFEYDFFKLSQQKLLQKLFNHQMDIHNLPRSKPEGLSRVCTGTKYAFLSTDLSVQSSWSQQNCNVI

AVPIDMFRCTLALILRKNSPYIGGMNFHIQILRGAGLLNHMLILYNIRDTSFHQLYAERNIMELQNIFPI

ICVLVLGIIFSFMCLIME

>LmigIR22

MDEGHQADCVFLDFKKAFDTVPHCRLLTKIRAYGIGSQLCDWLEDVLSNRTQPAFMFREPSLAAVGNVYT

RPFSRGVWLSYSLAAMLLALLVVGSQRLLASARGLLDDMTDPAVAPPWADVPLVGFSIICEEGASLVWGY

ELLSAFSAVHSFFNKHTVYYNPLSIFVFEIFDFEIRESFSGHPVFSTRELDQFLYQRTSKTLQEINNFFS

SMILSVHSRRSPIEFPEHFRDNRALVEPTSNEPSCSPLNCFYISSKRDRKYHKNNWMYFKVRVNPYCEGY

RDTFNINARPTPWRWSGIRPPPQNVSSRILLMFLLVLAVFHVTAYSACIVSLLQLPSGSINDLHSLFGSK

LRVVMQNLPYNFNYGNTHLVTPLCLAVKETKDPLTRSFYQERVYPQPYGKVFKPLEDCVALMRGGRFACH

ADEAAYKVIGDTFLEAEKCSLKSVPMFPLRAIILGVRKHSQYKETLSVIQAWLRETGLLKREWTRWVAQK

PRCLNRDSGYAEVGLTEVSPALLMLCYGVAGSLVALLLELLLHRAIAARPGRHQRQPKTPARGNALVNRA

FLR

>LmigIR12

LQPEVSSHSTDPSGKKFDGHRFKKDTNVNRCRGGVAKRIKALCSDERGHSPLGDYMAFETAWQIKIVCRN

RDSNLGPLPFAGKRTTDCAIPTRLTTPPHNFTSASTSPPSSRSASLVTSSALPKNWYEISGKCLSCVHAT

VSQTYQKLDKVLRHVIATARYEVSTYSGLVPGNQKEDKSWTGVIGLLQQGSAEIITDLLTVTASRVNAVD

FSSPATTDKFGLFIKDEVYTDVNRWSFVSPFETTLWMAVIGTILLYMLCVTVMNLASYDKNPQCSTKEIL

MGIFAAFCLRGYSLLLPRWSLRLAYLSAFITAVVIHAAYCARVVSHLANSNRSLPFTDLEEAYAAGYEIQ

VVPGTSAAETFKYASDGIIRTINEEMIEPRYFYLPVTINEGLLHMCNWKKTCFVSERNSVRCSQKQPCGI

IEVSVTMPSVYLAFALRKKSPFRRIISYQMEKLRTGGILKRLKSTSCNRAQADQGSDFKRVQLKNAAPLL

AIIYFAVIISFLVVILERALFRYTQKTNSKSVPKFRKYRKRIRQTPMYLP

>LmigIR29

LLKDLQGLALGAALPIVDTPLDNLGERLKNVNDRQLDTMARFGWGLSTTLAELLNFSIILYRVRNFGSLI

NDHEMDGAVALIHNGTVEFGAAGFIMTTRRMDFMDYTGPGRLWAPEIMFRHPKSASVLTTIFKPYTAELW

VSSGALFVLILVVSRLFCWVEHKVTATVDEIDNSWNSTFLLVSSAIGQQGVSRSSEWLSWRMLLFVSFLC

TNLLDTHYAAGIVSSLLMPPPRTINNKKDLADSTLGFGLENVSYTYQFFVKSDDPVDRALCSRKLYQAGG

RANFFPAEVGVLKMAREPFAFHAEDVRVGPLIDRFFSDDDKCALVFIPLLTPVATYTAVRRNSPVKELFN

FGLRMMWERGHVNYLRKAWYFTRVRCLSETEYASVDLVPMSPAFMLLGCAFLLSGFLL

>LmigIR18

FADSVNSVWLEKNLAKQRKALTFAAHNWTSGVRRCPRNKVLTIADLSYVATPGVFTKGVRRNVTWLVAAN

GGEWRRQLERCYVPLDALLLVAEALSAGQLVEITEVFSPRRGRPPTFAFFGRWTPATGLYTKRDDVTALY

RRRTSLDGAQLAVITYHDPPSVTVTQNETHTTVGGYFGEVWNTLAKHLRFRTQWLVQEWPNPGYNRADGT

WDGAAGALLKGEADVALVATTMRPYQGTPIAFSYPLYYSSRSDDIEGRPGVNSSYSASGAGRDRLFVRRM

DSPRSSWGDFVAPFERRLWVAVALSVPALAAALAALYRLGRHFGTADATGPYDYSFYDSLLYVFGAFCQQ

AIITFAAYSAALISSLTVVSDDLPFSDIEGLLRDGTYTVGVLNRTETFHGLMSPPGNTNGGLFYLRNIFT

REDALIIYYTVELHVLGILPYPIKGSNIPRSNLLLPLFRNHTFVYPLHKHPSVLVVPTRFIALEVKSSKV

FKVLKTRENQVGRHAKTATWSNKEMVSLLMPKHVLIFLALLVNIEVSLYEGC

>LmigIR13

MDRMCDCCVRAQEELAAVREQLSVLLAAVSRLKALGGSGASHGTPQVVLASPTIPAVGTSSRVPDAVESP

SPQGVWRVQRRSRRTGRKVNVEAGRAATPALPNKEFCTKSIWLVFLRGDEILEKAFEDMYIPLNCQLLVV

HQYGQQHFITEIFHLKEKLHLRKVLYGIWSAEHDLQKTADGFYSRRCDLLGETVKVSTFKSYNSEYGLSQ

LRLLSETLNFTYKFVKVKEMFPGRLTASGYSGMLGAITRREVDMTIDLLTHTTARSHVVDFLFPTQKDTH

SMFIKLSLDEGIPWTSYLSPFCGRLWGTVVLIITLDAVALAVLLRSSGHKVWTSDFLHLLLDVLGMYSLQ

GLKGSTGTDRPPCHPPPEVVIGYEIAKDLEEQLNGMENVLKGTHEMNINKSKTRVMECSLTKSGDAQEIR

LGVSVGSRTSAAQLVSVSAYVTAIVLQAAYCANVVSVVASRRYSPPFSDLAGLLADRTYRVAVMRESITN

DVFEFAGDKEMKLVYQHFIEPHNNDMPVLEEDIDVILCHRYRYCFTGESKLVDSHTVSCEIFEVRLKTPP

SHLGFALWKGHPYKNIFNYRYTIRIARLADVTRPAQLIVQTVAPVTTEKAAAPLQEPHICLSSPQIPLCC

GFTYGTPICILEARKPPHLGNVFDYDPALVPFLTDRLTRDENSTNGIEDDTSNGEGD

>LmigIR6

MRSLLIVTMLVNSMCDGRLVLQNNDVSSIDSLIRELGSRILSKEFGSYKCVAALSDVPEAVAYFTQWFPT

TVLISLVSGDVSAEDLLVSALDADCQGFLVRCRDAAAGVDAILKASKFAMRRVNRRLLVLPTAQSPHINI

TAIFDLRNIDLVPEIVVARPSGDNSFDLVTLKLTGEDTWRDELLVAQWYRGRGLQPPSADLYAHRLADLG

GRRIVISTVDYPPYVIFNSGDNVFDGIEARIIIEFIKKINVTWSVVEDTTYKWGTVWCNGSGNGMLGAVA

ADEVDVAFSALYRWYPEFMEYSRQYVRSSVVCLAPRPLMLAGWQVPVLPFSPAMWAAVGFSVVVATAALY

AVKKLSDWVLGSDDDPSGGRYSTVEDCFFRSVGLLVLQTPDVERRHTRVVGPTRHVLSWLLITYLLVTSS

YGGGLSSVLTIPRYEPPIDTVTDLHDSGLEWAQTDIAYLNSLRGLTDPVYLDLIDRYRVLTPDMLRSRVT

TRDLAFAVEKLPGGYFAIGDYIDEEAVSNWLRPMREDIYWGTVVLATRKCWPHLERLNDIIDRLFEAGII

RAWEGTVAREWLVARVQLAAQVGMRSAQAPDGPIKLRLTHVQGGFALLILGLCLSLLLFFLEIFVYWKSN

GSTRLEFSSTIKVTLEHYRS

>LmigIR15

MFIGFEQYASCKIPIKTVAAVVSSLQDEIHFGCVYLTYSSREAELLPQSRFHVEFRRGLRNSIQLGFLPD

THETQLRHNCSSSPLLVVPHTDLLLNHSSVIKDLLRKRQSDFMWLLLLNDGNMDAALADIYVEFDSNFFV

IFESTDGTVVIVEAYRVAKDFPLVTKKFCTWQINTNLTCTKEGIRSRRNDLMGYELKTGVVEVWPDMYDL

GNGSCSGYLCDIWNILSRKLNFTKLAKHNGHTIIRKPLNVSNTVAIEEEEKEARQRKINVWIHSTWITKP

VQKEFRTLFPHLMEPYGPAAVAELSAARQSQSARPLQSWVRMRPRAWLFVPSLLLVSSKQTRWVKDGVSQ

QRVGFESGELMLLFLRMQNSQCGDHLLRLLQENLFGVRYRLQRSSLFGSRLSDGSWSGAVGLLAGGEAEV

ALSVMMITPSRLSAVDFSLPFFSTREYLYIREPEEEDLAWDGFLRPFDGPLCVCTVAVVLVIWGVLKVHH

RCAPSAGTQLDEGRLYSDFLHVIGIFCMQGCASASRDSARLSLLVASMCALVVYTAYCGALTASLATHQP

RLPFTDLKGLLQDGSYQLLLLDGSGEMTILAIWPSCAFVWSPSHIGVLVNEHVNRLAKQATTTPALEIGF

TESNLKLALHLKESEDPVTKEIYNNMVVKDDLPEDSNEAFDRLCSSEKQVLLCQLMDFRLNENSLKCNVT

TTQEYITSQLAIAFRKRSPYREIINYQLTNYEWGAQPSVVRTSVLAVCVSASEYEAPVWDASAHTKQVDV

SVKETLRIVTGCLRPPPTGRRRHQGLKEACGESALPYRTVARWVKAFNEDRQTVAATHRAGLPSAPEEDC

ILLPRWRTVIDARRFVNSPMKPD

>LmigIR14

MRRGAININGDYTGKKVELAETSSKMGLDVLAFSDVRVRGEKEDEVGEYNVYLSGVKAGRAQWGVGFYIR

KAIEPSIIAIRPFFTWQELLKTRQSEFMWLLLLRRDQSPSAVLQDIDIRFDTLFFVAVPTTESDVHIVEA

YRVAKGYPLVMQTFCTWNTTYHPSCRTESTIARRGDLMGYKMKSGIVEHSPMVQEGLSNLMRPPRHELAS

RYQFKEMSGWGIRSRDGSWSGLIGRLVSGNLDVGVAYKLYIQTPSEQEFIWGHFLWPFDKWLWLCSFALV

VTTWLVLRVQERFTRDDESEISARGTYNDFLQVLGIFLMQGVYGGRTAPRACVRASLCAASLLALLLYTA

YCGALTASLASRRPRLPFRDMAGLLRDASYSLVTIADSSILNVLSAERQAEDEALRGGSDECGPQMEHRQ

PPVEREILNPVHSESSLRSHLMKDSNNPVDRKIYEKHIEGKHMPHTSEELFGWLCSSQKHAMLVSKTRYM

HGTDFVQCPVTSVHETSGSQEGFVLTKKSPYREAINYHHDCHRDGEASGSDPPLSSKPMDPINPGNYATN

QQLCLYPVCEGSSLHQFRQLPIGTEDVLRTQATSIIDADVSHDLQSTAPHNLDSRQQDRIHISDEAPRQT

H

>LmigIR9

MQLHPSPCENWTEPHVWIHYPYRKENPMLRVPASQFVVVETLATTHRYWNLEGAHISAVMYPAYPTAMPA

GQREINTKTCDGDTTGYIELHANSVRIFKYVRGSDHFLRLTLEEKLNARVVARYPKDENFVEGEELPNGQ

LTGALAEMASGQAQLGLNLRFVRPLPTPEILYLQPVCSFARVGIAVARAPLAPLHVAIMRSFDLKVWFSV

AAFYILSAMIWSLLTSSINYKSVLQEIQKFLLIGCGSFAQSPSQKFFASACAIFSLIIVTIFQEKEKERK

GEEEIRKELGKTNGSLVNVLTTPVYDRDINTANELLQSGLPIATNRTSLIHMFQLAAEGSPVMTELCKHM

KPTDKHNLTELLFDRKQALIIADAGLAQLKNHHPSQEKMLHMIKDTFIVLQRAYAARTDCPVKEQVEKII

WCLTEAGIPRYWNTQASRSEFSNCPLPEDTPKPFSLKQFKIAFYTLCIGLLFSSFVFLLEIISYKRCQKH

ILK

>LmigIR7

MLAWVAVTSFFLWPDVVARHQPAVVTNSSLEDCLAYVFSSQPFTWAPLLLVTTTSPDDREPDVSAITNAV

VHTVQLPVVLVKLPTTSVYADLVAGYIIVLPDSDLDQDTFQSILVNISYVANWSPRRPVLVIFRAGYGRH

EDSVRMILDCLDKRNAIEATVFVVNVERTSWEVFGYYTVRNISAYTWFPYRQGHCRDDGVETELIGNIAT

TGRHNFTVFPEKLPRSLHGCSVVAATYHLPPFVSKVESEGRQTSFSGYDVILFRLLARAANFRPVVSAPH

FSGPQWGERLENGSWSGVVGRMLRRQADAAFGGLHDNAXXXXXXXXXXXXXXXXXLPDALTWFAPRARRA

CALCEFGLIVPARDAGLLLLGALLMSVPFKLFSGQDEISALRRLDAAFLFALRMLVTTPARAPRTLRPRI

VFIFWLQLTYILITGYQSTVTSKMVQPGTEEQILTLEQLLRSDLYCGTLLPFQRWMDTVMKSKAPKISYL

CPNIADCMFRMAYSKDLAVMTTKVHTEFMGKVKYVDQAGRSLFLPFTENLFTYYVAVHVQQGSPLLPLFD

DVILRVQAGALMTPAMREYERLFRYGTVDHSDLPEEVDSQGFIALSHLSGAFTAWAVALATAFCVFVAEL

AYWRSGFRLFARQLRCSRLPEKEKDKNAFGSDNFITEKKGRKLRL

>LmigIR10

LDVRLLASTHGLCRSAVADVATSILRSHHPRPAVVSTGPTYLPPATTFLTPLQNEQRLLVTDSASPVVEF

FQFGRAHSRLWSATNDFIILLLKAYDHDGGDCNADCANLLRHLWSAFGAARVLVRHVEVSDGCHVHRLGP

TVALFDPFDTSAGTIVSTTWTNVELIKTLLDNRTRDLRGRPFRVCMFARNYTALPVDANGVLVYRSSLET

VKYAGVDGWALQTLAQYMNFTTVFRQPYDRERYGELLPNGSFTGALGELMRGRVEIIFNAIFMKSYGSHE

IYFTKGVSKDDIRLVVPKGGLLSKRHLLTMLMRPEVQLSILVTYLLMTLLWYASSRLHMFFKPEDAERPD

ILMIAFQNFHPPTSSALEKLPRHAAERFLVVSYLLFHMLVFTIVQGKMLEVINNPRYEDDINTLQELVAS

HLPIKTGSATLIDIFGPTGSAPQPESLLQSLSRKTNENHQKGGRKPDGNLRFNVMIFRAYVSASFVALLL

STGHRHSSVAPMAFTFTVLGNECIKELDDVIDNMVLLEKGSAVEEVARSRDAACVVRDSDLRHGKYSAYR

RADGSWLLHAVQEAALSFELGYATRRDSPFLAAIDRFLWRAVEAGLSRCWERRGYLLVTLRARADPASID

TAAAAAKTYKQFTVSDLQMPFYAWIVGVASSVICFAAELLYSWWLSGHKN

>LmigIR8

LCGKATLADVTRPAQSIIQTVAPVTTEKAAAPLQEPHVCLASPQIPIRCGCTYGTAICIVEARKPPHLGN

VCGSWEWDTAVKFHKVNKRLNIFEQTNLHWTVDKRRKTRNERCSLSLLSRKNSGLTSLETFANTRRISAA

EVWEMTSRIFFHETINSDRIVDWPPRYADLNPCDLYLWGMLKELACREMNFSSGTVLTYDPFLVKDSIWS

DSYTPKLQEVKETADIYYKKYMNLNGNNVRASMFTVNPSAIEDNTTVSKFRGCDAQMIETLAKYMNATLV

MLPNDGSGFGKWNGTVNTGTDGDVMFDRADIAPNTRYVIVERLKVHGYTYPHDKEDLCILVNKSPRIPQY

LNIILPFALIAWLTILLSLPFSALFWSLIRRFGARPSNSEPFARVYIGSFLKIFSAFLSVAVSALPTVGR

ERILFVMWTFFSLIITNTYQGSLTSYLTIPKYMPDIDTMEELSKSGLKILIHPELLPVFKLDTGNPVMDA

LNRNLVSDIDMEGYPEKIQSNTDTCALVNAYVGQFLIRSRHYVINGFPLLHLTNECPMPAVVAFATPKFS

PLQPRFDVLIRRIVEAGLYKKWQKNMLDESIASGDLLLISNREGKADPERITLSHLQMPFYLLFLGYFLT

SVLFLTEYLRLKMNTRSTNEEEAKLERREGCQTLENDSASYLLDV

>DmelIR8a

MELPLLVLLLALRFAGSEVLKITFWIEPVQRAEFDTDIAMVLKELDALRLDVKVDDTTLT

LTRSEDGLDMQRFCEILSTVGASAVIDLTYSHWEEGYNLVRSLGIGYVRLERIMRPFLDM

FGDFMRQKRANNVAMVFMNARDAVEAMQQMLVGYPFRTLIMDASQTDPGQHFLERIRSLR

PAPTYIALFARAAAMNGIFEKVQKADLFQRPLEWHFVFLDTRDRVFKYRRQAELCTRFTL

NPRAICRSMPMPDLYCGSGFTMQRAMLLNVLRSLINAAQVSPGYPLAIYQDCNATASSSE

VSDPLEKDDYNWLDMVHWSNFLAYAPPLPHIQDQFQSPVPGLTFAVNISAGYYSSEHEAK

TDLAAWSSVGEMRLLNETISPARRFFRIGTAESIPWSYLRREEGTGELIRDRSGLPIWEG

YCIDFIIRLSQKLNFEFEIVAPEVGHMGELNELGEWDGVVGDLVRGETDFAIAALKMYSE

REEVIDFLPPYYEQTGISIAIRKPVRRTSLFKFMTVLRLEVWLSIVAALVGTAIMIWFMD

KYSPYSSRNNRQAYPYACREFTLRESFWFALTSFTPQGGGEAPKAISGRMLVAAYWLFVV

LMLATFTANLAAFLTVERMQTPVQSLEQLARQSRINYTVVKDSDTHQYFVNMKFAEDTLY

RMWKELALNASKDFKKFRIWDYPIKEQYGHILLAINSSQPVADAKEGFANVDAHENADYA

FIHDSAEIKYEITRNCNLTEVGEVFAEQPYAVAVQQGSHLGDELSYAILELQKDRFFEEL

KAKYWNQSNLPNCPLSEDQEGITLESLGGVFIATLFGLVLAMMTLGMEVLYYKKKQNALE

ITQVRPVNDSSGSGGNSSTAPPTATSTTKQAWHIPVLEAEEKPAKVSPPPSFETATFRGK

KLPARITLGDGKFKPRHGLYARRNLGASDSHSGYME

>DmelIR25a

MILMNPKTSKILWLLGFLSLLSSFSLEIAAQTTQNINVLFINEVDNEPAAKAVEVVLTYL

KKNIRYGLSVQLDSIEANKSDAKVLLEAICNKYATSIEKKQTPHLILDTTKSGIASETVK

SFTQALGLPTISASYGQQGDLRQWRDLDEAKQKYLLQVMPPADIIPEAIRSIVIHMNITN

AAILYDDSFVMDHKYKSLLQNIQTRHVITAIAKDGKREREEQIEKLRNLDINNFFILGTL

QSIRMVLESVKPAYFERNFAWHAITQNEGEISSQRDNATIMFMKPMAYTQYRDRLGLLRT

TYNLNEEPQLSSAFYFDLALRSFLTIKEMLQSGAWPKDMEYLNCDDFQGGNTPQRNLDLR

DYFTKITEPTSYGTFDLVTQSTQPFNGHSFMKFEMDINVLQIRGGSSVNSKSIGKWISGL

NSELIVKDEEQMKNLTADTVYRIFTVVQAPFIMRDETAPKGYKGYCIDLINEIAAIVHFD

YTIQEVEDGKFGNMDENGQWNGIVKKLMDKQADIGLGSMSVMAEREIVIDFTVPYYDLVG

ITIMMQRPSSPSSLFKFLTVLETNVWLCILAAYFFTSFLMWIFDRWSPYSYQNNREKYKD

DEEKREFNLKECLWFCMTSLTPQGGGEAPKNLSGRLVAATWWLFGFIIIASYTANLAAFL

TVSRLDTPVESLDDLAKQYKILYAPLNGSSAMTYFERMSNIEQMFYEIWKDLSLNDSLTA

VERSKLAVWDYPVSDKYTKMWQAMQEAKLPATLDEAVARVRNSTAATGFAFLGDATDIRY

LQLTNCDLQVVGEEFSRKPYAIAVQQGSHLKDQFNNAILTLLNKRQLEKLKEKWWKNDEA

LAKCDKPEDQSDGISIQNIGGVFIVIFVGIGMACITLVFEYWWYRYRKNPRIIDVAEANA

ERSNAADHPGKLVDGVILGHSGEKFEKSKAALRPRFNQYPATFKPRF

>DmelIR21a

MSYYWVALVLFTAQAFSIEGDRSASYQEKCISRRLINHYQLNKEIFGVGMCDGNNENEFR

QKRRIVPTFQGNPRPRGELLASKFHVNSYNFEQTNSLVGLVNKIAQEYLNKCPPVIYYDS

FVEKSDGLILENLFKTIPITFYHGEINADYEAKNKRFTSHIDCNCKSYILFLSDPLMTRK

ILGPQTESRVVLVSRSTQWRLRDFLSSELSSNIVNLLVIGESLMADPMRERPYVLYTHKL

YADGLGSNTPVVLTSWIKGALSRPHINLFPSKFQFGFAGHRFQISAANQPPFIFRIRTLD

SSGMGQLRWDGVEFRLLTMISKRLNFSIDITETPTRSNTRGVVDTIQEQIIERTVDIGMS

GIYITQERLMDSAMSVGHSPDCAAFITLASKALPKYRAIMGPFQWPVWVALICVYLGGIF

PIVFTDRLTLSHLMGNWGEVENMFWYVFGMFTNAFSFTGKYSWSNTRKNSTRLLIGAYWL

FTIIITSCYTGSIIAFVTLPAFPDTVDSVLDLLGLFFRVGTLNNGGWETWFQNSTHIPTS

RLYKKMEFVGSVDEGIGNVTQSFFWNYAFLGSKAQLEYLVQSNFSDENISRRSALHLSEE

CFALFQIGFLFPRESVYKIKIDSMILLAQQSGLIAKINNEVSWVMQRSSSGRLLQASSSN

SLREIIQEERQLTTADTEGMFLLMALGYFLGATALVSEIVGGITNKCRQIIKRSRKSAAS

SWSSASSGSMLRTNAEQLSHDKRKANRREAAEVAQKMSFGMRELNLTRATLREIYGSYGA

PETDHGQLDIVHTEFPNSSAKLNNIEDEESREALESLQRLDEFMDQMDNDGNPSSHTFRI

DN

>DmelIR31a

MNLLISMFILILAAGEGEIIPSMEESVVTNFVKSLVKTKQAIVFSCLFKDFKEISLALMR

INQFVSVVNLNQSYSLTSILTRENYARTSVMVNARCSGSSELLFEASENRYFNKTYQWFL

WGVDLEVQSLFPLNLNYVGPNAQITYVNETADGYAYWDIHSKGRHLKSNLEINLIATLIN

DTLNIARDIFHLQSIDFRGQFNGLTLRGASVIDKEDIISNEQIESILSRPTKDAGVAAFI

KYHYELLGLLRERFNFTVNFRNSRGWAGRLGNTTFRLGLLGIVMRNEADIAASGAFNRIN

RFAEFDTIHQSWKFETAFLYRYTSDLDTHGKSGNFLSPFSDRVWLFCLLTLGAFSIIWVL

FEIIDYKILRIRVNSQKLEHLNQKSSVICIKTTCIERILQTFGACCQQGLDPNPVDRSVR

FLVMTLFLFSLVMYNYYTSSVVGGLLSSSDQGPSTVDEITASPLKISFEDIGYYKVLFRE

SQNRSITRLIEKKLSSSRSLNELPIFSHIEDAVPYLKAGGFAFHCEVVDAYPVISEYFDA

NEICDLREVSGLMEVEILNWILHKNSQYTEIFKTAMCNAQEKGFVERILRRRQIKKPACQ

SLYTVYPVSLSGVLPGFVILICKSINKFS

>DmelIR40a

MHKFLALGLLPYLLGLLNSTRLTFIGNDESDTAIALTQIVRGLQQSSLAILALPSLALSD

GVCQKERNVYLDDFLQRLHRSNYKSVVFSQTELFFQHIEENLQGANECISLILDEPNQLL

NSLHDRHLGHRLSLFIFYWGARWPPSSRVIRFREPLRVVVVTRPRKKAFRIYYNQARPCS

DSQLQLVNWYDGDNLGLQRIPLLPTALSVYANFKGRTFRVPVFHSPPWFWVTYCNNSFEE

DEEFNSLDSIEKRKVRVTGGRDHRLLMLLSKHMNFRFKYIEAPGRTQGSMRSEDGKDSND

SFTGGIGLLQSGQQADFFLGDVGLSWERRKAIEFSFFTLADSGAFATHAPRRLNEALAIM

RPFKQDIWPHLILTIIFSGPIFYGIIALPYIWRRRWANSDVEHLGELYIHMTYLKEITPR

LLKLKPRTVLSAHQMPHQLFQKCIWFTLRLFLKQSCNELHNGYRAKFLTIVYWIAATYVL

ADVYSAQLTSQFARPAREPPINTLQRLQAAMIHDGYRLYVEKESSSLEMLENGTELFRQL

YALMRQQVINDPQGFFIDSVEAGIKLIAEGGEDKAVLGGRETLFFNVQQYGSNNFQLSQK

LYTRYSAVAVQIGCPFLGSLNNVLMQLFESGILDKMTAAEYAKQYQEVEATRIYKGSVQA

KNSEAYSRTESYDSTVISPLNLRMLQGAFIALGVGSLAAAALNNTINVRSLNSRDKFICG

GPVKIWYYLVLLLWYYFNRGLVGIYQLWHKTSIRNTGKGMPFLGE

>DmelIR64a

MHWWLLVFLPLSCQGLPEHELLELELDYGLAEPQRTSLLQSSLILQFSQDYKHIPRITYF

TCQKPHLQTPNQIPNAAEHRDAFAAKNFQLIKSLYESELFVRIVLLDVLAQSPTSGRPNR

PGNGPTGGFSQTPSQAQSNSEWLEGVLRMEALRQIAVVDLACGAVSRRFLELASAKMLYS

EKFHWLLIEDFAWHGRTQTAEGSGKRDDGEMEEEEPPGQQIQATDDEDLPSIESFLGGMN

LYMNTELTLAKRMSEAAHYTLFDVWNPGLNYGGHVNLTEIGSFTPTEGIQLHTWFRTTST

VRRRMDMQHARVRCMVVVTNKNMTGTLMYYLTHTMSGHIDTMNRFNFNLLMAVRDMFNWT

FVLSRTTSWGYVKNGRFDGMIGALIRNETDIGGAPIFYWLERHKWIDVAGRSWSSRPCFI

FRHPRSTQKDRIVFLQPFTNDVWILIVGCGVLTVFILWFLTTIEWKLVPHDGSALIKPKG

GAPPRHHYQQQQQQEQVEAPVRPITAVSVVVSKEKVEEKQEEYEDSTPIDAGTLWQRCYQ

KLNKYIKDRKAKQKKAPERVGLFLESVLFFVGIICQQGLGFSTSFVSGRCIVITSLLFSF

CIYQFYSASIVGTLLMEKPKTIKTLSDLVHSSLKVGMEDILYNRDYFLHTKDPVSMELYA

KKITSVPTTKENEADEDEPVDPNPVSTDPAKSYRDIVHSHETGAHAKDNAASNWLDPETG

LLRVKHERFAFHVDVAAAYKIIAETFSEQDICDLTEVSMFPPQKTVSIMQKNSPMRKVIS

YGLRRVTETGILTYHFNVWHSRKPPCVKKIETSDLHVDMDTVSSALLILLFSYAITLMIL

GTEILYSKWHNRIQLKWVGAT

>DmelIR75a

MQLVQLANFVLDNLVQSRIGFIVLFHCWQSDESLKFAQQFMKPIHPILVYHQFVQMRGVL

NWSHLELSYMGHTQPTLAIYVDIKCDQTQDLLEEASREQIYNQHYHWLLVGNQSKLEFYD

LFGLFNISIDADVSYVKEQIQDNNDSVAYAVHDVYNNGKIIGGQLNVTGSHEMSCDPFVC

RRTRHLSSLQKRSKYGNREQLTDVVLRVATVVTQRPLTLSDDELIRFLSQENDTHIDSLA

RFGFHLTLILRDLLHCKMKFIFSDSWSKSDVVGGSVGAVVDQTADLTATPSLATEGRLKY

LSAIIETGFFRSVCIFRTPHNAGLRGDVFLQPFSPLVWYLFGGVLSLIGVLLWITFYMEC

KRMQKRWRLDYLPSLLSTFLISFGAACIQSSSLIPRSAGGRLIYFALFLISFIMYNYYTS

VVVSSLLSSPVKSKIKTMRQLAESSLTVGLEPLPFTKSYLNYSRLPEIHLFIKRKIESQT

QNPELWLPAEQGVLRVRDNPGYVYVFETSSGYAYVERYFTAQEICDLNEVLFRPEQLFYT

HLHRNSTYKELFRLRFLRILETGVYRKQRSYWVHMKLHCVAQNFVITVGMEYVAPLLLML

ICADILVVVILLVELAWKRFFTRHLTFHP

>DmelIR75b

MNFSVLESHFKEAQIFVDADVTYVTHDPFSKNFLLYDVYNKGRQLGGELNITADREIFCN

KTNCRVERYLSELYTRSALQHRKSFTGLTMRATAVVTALPLNVSIKEIFDFMNSKYRIQL

DTYARLGYQARQPLRDMLDCKFKYIFRDRWSDGNATGGMIGDLILDKADLAIAPFIYSFD

RALFLQPITKFSVFREICMFRNPRSVSAGLSATEFLQPFSGGVWLTFALLLLLAGCLLWV

TFILERRKQWKPSLLTSCLLSFGAGCIQGAWLTPRSMGGRMAFFALMVTSYLMYNYYTSI

VVSKLLGQPIKSNIRTLQQLADSNLDVGIEPTVYTRIYVETSEEPDVRDLYRKKVLGSKR

SPDKIWIPTEAGVLSVRDQEGFVYITGVATGYEFVRKHFLAHQICELNEIPLRDASHTHT

VLAKRSPYAELIKLSELRMLETGVHFKHERSWMETKLHCYQHNHTVAVGLEYAAPLFIIL

LGAIILCMGILGLEVIWHRHCTLH

>DmelIR75c

MTSWPLYRLIVFNLLEINLSNLMVFHCWSIKEAFPLVEMLNQNGIFSQYIDVQNPDNLAN

VHKEYLDSDLVSLNADVTYVSREDEERFILHDVYNKGSHLGGKLNITVDQTLQCNRSHCQ

VKEYLSELHLRPRLQHRMDLSSVTFRLAALVSVLPINSSEEELLEFLNSDRDSHMDSISR

IGNRLIMHTQEILGFNVQDAFGGAIGMLTNESAELCTTPFVPSWNRLHYLHPMTEQAQFR

AVCMFRTPHNAGIKAAVFLEPFMPSVWFAFAGLLIFAGVLLWMIFHLERHWMQRCLDFIP

SLLSSCLISFGAACIQGSYLMPKSAGGRLAFIAVMLTSFLMYNYYTSIVVSTLLGSPVRS

NIRTIQQLADSSLDVGFDTVPFTKTYLVSSPRPDIRSLYKQKVESKRDPNSVWLSPEEGV

IRVRDQPGFVYTSEASFMYHFVEKHYLPREISDLNEIILRPESAVYGMVHLNSTYRQLLT

QLQVRMLETGITSKQSRFFSKTKLHTFSNSFVIQVGMEYAAPLFISLLVAYFLALLILIL

EICWARYAKKKFSTIIPQNQ

>DmelIR75d

MKVQVAHWLPLIFFLLVSGTPRVAGSWRSEYSRQDPDPKTRWGNQLPDMLVAYYRHHGVH

SLMLVVCHTDIADFRLWKLWQHFNLNNFYVQVSTESSLRDLQHVDALDEHKDAPPPKSFH

ANNSTHWETSFLLPALPYKMGILLLEFSSECALNLLRWSAASEHNYFTTNRFWLLLTEDP

GDIDLLEDPEIFIPPDSELRVLHYENVGNFSCSLIDLYKVAAWKPLKRTLVGHNIRNSRH

VIHALQHFGSAITYRQDLEGIVFNSAIVIAFPDLFTNIEDLSLRHIDTISKVNHRLMLEL

ANRLNMSYNTYQTVNYGWRQPNGSFDGLMGRFQRYELDLAQLAIFMRLDRIALVDFVAET

YRVRAGIMFRQPPLSAVANIFAMPFENDVWVSILMLLIITTVVLVLELFFSPHNHDMSYM

DTLNFVWGAMCQQGFYVEVRNRSARIIVFTTFVAALFLFTSFSANIVALLQSPSDAIQSL

SDLGQSPLEIGVQDTQYNKIYFTESTDPVTKNLYHKKIASKGENIYMRPLLGMEKMRTGL

FAYQVELQAGYQIVSDTFSEPEKCGLMELEPFQLPMLAIPTRKNFPYKELIRRQLRWQRE

VSLVNREERKWIPQKPKCEGGVGGFVSIGITEcRYALGIFGCGAAVSFVLFLFEFIFRHF

KQVYRIIKGYREVQR

>DmelIR76a

MENLLVESYYFSTVLSFFAQQFFADSHATCIFWHPAFDFRLETVHPMPLIIMDWHRWANR

SDQDVYDYKIKEDEFEGKGIPYNDWTLRLTVAIERSHCETFIAFQEQIPEFARYFYHASI

YSIWRSLRNRFMFVYTKEFEDKKDSYLSGYIFQDQPNILVITSQYLNSSTFEIKTNRFVG

PRNFNKNPEPVEFYILQRFDAKGTKATWETQSAMSSKMRNLKGREVVIGIFDYKPFMLLD

YEKPPLYYDRFMNTTDVTIDGTDIQLMLIFCELYNCTIQVDTSEPYDWGDIYLNASGYGL

VGMILDRRNDYGVGGMYLWYEAYEYMDMTHFLGRSGVTCLVPAPNRLISWTLLLRPFQFV

LWMCVMLCLLLESLALGITRRWEHSSVAAGNSWISSLRFGCISTLKLFVNQSTNYVTSSY

ALRTVLVASYMIDIILTTVYSGGLAAILTLPTLEEAADSRQRLFDHKLIWTGTSQAWITT

IDERSADPVLLGLMEHYRVYDANLISAFSHTEQMGFVVERLQFGHLGNTELIENDALKRL

KLMVDDIYFAFTVAFVPRLWPHLNAYNDFILAWHSSGFDKFWEWKIAAEYMNAHRQNRIV

ASEKTNLDIGPVKLGIDNFIGLILLWCFGMICSLLTFLGELWRGQG

>DmelIR76b

MATGIELLVAAALCVACPPLNDSPPTNLIQMGENGTLSPVTELPMDVDASEAGFDADAPV

ETLETINRKKPKLREMLDWIGGKHLRIATLEDFPLSYTEVLENGTRVGHGVSFQIIDFLK

KKFNFTYEVVVPQDNIIGSPSDFDRSLIEMVNSSTVDLAAAFIPSLSDQRSFVYYSTTTL

DEGEWIMVMQRPRESASGSGLLAPFEFWVWILILVSLLAVGPIIYALIILRNRLTGDGQQ

TPYSLGHCAWFVYGALMKQGSTLSPIADSTRLLFATWWIFITILTSFYTANLTAFLTLSK

FTLPYNTVNDILTKNKHFVSMRGGGVEYAIRTTNESLSMLNRMIQNNYAVFSDETNDTYN

LQNYVEKNGYVFVRDRPAINIMLYRDYLYRKTVSFSDEKVHCPFAMAKEPFLKKKRTFAY

PIGSNLSQLFDPELLHLVESGIVKHLSKRNLPSAEICPQDLGGTERQLRNGDLMMTYYIM

LAGFATALAVFSTELMFRYVNSRQEANKWARHGIGRTPNGQSVAPSRWLRGWRRLNSGHG

QLLGASTHGQNVTPPPPYQSIFNGGSHGDPLNRWRRPLANGNALGNGVLLGGDSEGGVRR

LINGRDYMVFRNPNGQSQLVPVRSPSAALFQYSYTE

>DmelIR84a

MIKLQVKVISWPLIILTAFLRVLQIESINTNFLELAAFEDFLRSEHLSHVLVVRGDDADG

DWKIECHQKLLANYRVQFYRPEMSANFEDLMFYGSPRTAVLVLNSEHVLVRRQVFGVASE

AGYFNNSLAWFILGSGRESLPVEQLIDQLLSGYRMGIDADITVALRGPDNASMLFYDVYR

ISRQANTPLIIEKKGLWTHSGGYQKFGNFKNTWVIRRRNFLNVTLIGSTVLTEKPPGFGD

MEYLADDKQLQQLDPMQRKTYQLFQLVERMFNLSLAISLTDKWGELLDNGSWSGVMGQVT

SREADFAVCPIRFVLDRQPYVQYSAVLHTQNIHFLFRHPRRSHIKNIFFEPLSNQVWWCV

LALVTGSTILLLFHVRLERMLSNMENRFSFVWFTMLETYLQQGPANEIFRLFSTRLLISL

SCIFSFMLMQFYGAFIVGSLLSESARSIVNLQALYDSNLAIGMENISYNFPIFTNTSNQL

VRDVYVKKICKSGEHNIMSLQQGAERIIQGRFAFHTAIDRMYRLLLELQMDEAEFCDLQE

VMFNLPYDSGSVMPKGSPWREHLAHALLHFRATGLLQYNDKKWMVRRPDCSLFKTSQAEV

DLEHFAPALFALALAMVASALVFLLELFLHWLPDFRRRLGTMST

>DmelIR92a

MLLQPLVMHLSQLLRIIVGQYFAEFPSILIVYNNSASTTPLQLEYLSALELVLRELSKPI

RLQWINVAFLKDLNDLEDQVMGALNSSVTEGFITILSQTHHFIHARYYATRNANVRLKDK

RYLFLCEDESPAELLCMDILQFYPHHLMVRPGTETAPTGPTGPHPDPRRGGGASVSTKNK

DDGEGGAGNKTTSPYRDINFELWTQKFVGAVGNLDALLLDAFLPNETFANRVELYPNKLL

NLQRRSLLVGSITYVPYTITNYVPAGQGDVDPIHPQWPNRSLTFDGAEANVMKTFCQVHN

CHLRVEAYGADNWGGIYDNESSDGMLGDIYEQRVEMAIGCIYNWYDGITETSHTIARSSV

TILGPAPAPLPSWRTNIMPFNNRAWLVLISTLVICGTFLYFMKYVSYRLRYSGTQVKFHH

SRKLEKSMLDIFALFIQQPSAPLSFDRFAPRFFLATILCATITLENIYSGQLKSMLTFPF

YSAPVDTIEKWAQSGWKWSAPSIIWVHTVQSSDLETEQILARNFEVHDYSYLSNVSFMPN

YGFGIERLSSGSLSVGDYVSTEALENRIVLHDDLYFDYTRAVSIRGWILMPELNKHIRTC

QETGLYFHWELEFIDKYMDKKKQEVLMDLANGHKVKGAPQALDVRNIAGALFVLAFGVAF

AGCALVAELLIHRMDLSK

>DmelIR93a

MNPGEMRPSACLLLLAGLQLSILVPTEANDFSSFLSANASLAVVVDHEYMTVHGENILAH

FEKILSDVIRENLRNGGINVKYFSWNAVRLKKDFLAAITVTDCENTWNFYKNTQETSILL

IAITDSDCPRLPLNRALMTVEcRINAVVFVDQTILEENALLVKSIVHESITNHITPISLI

LYEINDSLRGQQKRVALRQALSQFAPKKHEEMRQQFLVISAFHEDIIEIAETLNMFHVGN

QWMIFVLDMVARDFDAGTVTINLDEGANIAFALNETDPNCQDSLNCTISEISLALVNAIS

KITVEEESIYGEISDEEWEAIRFTKQEKQAEILEYMKEFLKTNAKCSSCARWRVETAITW

GKSQENRKFRSTPQRDAKNRNFEFINIGYWTPVLGFVCQELAFPHIEHHFRNITMDILTV

HNPPWQILTKNSNGVIVEHKGIVMEIVKELSRALNFSYYLHEASAWKEEDSLSTSAGGNE

SDELVGSMTFRIPYRVVEMVQGNQFFIAAVAATVEDPDQKPFNYTQPISVQKYSFITRKP

DEVSRIYLFTAPFTVETWFCLMGIILLTAPTLYAINRLAPLKEMRIVGLSTVKSCFWYIF

GALLQQGGMYLPTADSGRLVVGFWWIVVIVLVTTYCGNLVAFLTFPKFQPGVDYLNQLED

HKDIVQYGLRNGTFFERYVQSTTREDFKHYLERAKIYGSAQEEDIEAVKRGERINIDWRI

NLQLIVQRHFEREKECHFALGRESFVDEQIAMIVPAQSAYLHLVNRHIKSMFRMGFIERW

HQMNLPSAGKCNGKSAQRQVTNHKVNMDDMQGCFLVLLLGFTLALLIVCGEFWYRRFRAS

RKRRQFTN

>DmelIR7a

MFHHLWLLMGLRSLAMGALHPPQPEAMTPLVAAALEILAEQVSPSQSTLAVMDLTQDAEH

RDERQEQLMTIILRSVGSEMALRTFQKPPAEVPASFVVFLVNSAQAFNTLGFHFTDIHST

REFNFLILLTHRMSSRAERLQVLRDISRTCVRFHTSNVILLTEKRDGVVLVYAYRLLNMD

CDLSVNLELIDIYKNGLFRHGHEARSFNRVLSLSGCPLQVSWYPLPPFVSFIGNSSDPEE

RAQIWRLTGIDGELIKLLASIFDFRILLEEPCNKCLSPDIKDDCSGCFDQVIISNSSILI

GAMSGSHQHRSHFSFTSSYHQSSLVFIMHMSSQFGAVAQLAVPFTVIVWLALVVSSLLLV

LVLWMRNRLVCGRSDLASHALQVLTTLMGNPLEARSLPRSSRLRILYAGWLLLVLVLRVV

YQGKLFDSFRLPYHKPLPTEISELIRSNYTLINQEYLDYYPRELTVLTRNGSKDRFDYIQ

GLGKEGKFTTTSLIATMEYYNMMHWSTSRLTHIKEHIFLYQMVIYLRRHSLLKFAFDRKI

KQLLSAGIIGYFVREFDACQYRKPFEEDYEVTPIPLDSFCGLYYISLIWLSAAVVAFILE

LLSQRIVWLRRIFE

>DmelIR7b

MKYWLYILSCCSLVASTMESSSDWDLAEALAQVVANSEMGRFKTLYIYTHTNSQSTGGHL

EELLDQVLMIVPNNLQARRLLLQQSMEYKPYVHAVLALVDGLPSLSAIYARIRATQDLSH

TLIYMSMPTDAYGEEMQATLRFLWRLSVLNVGVVLRPPGDHILMVSYFPFSALHGCQVIS

ANVVNRYQVGTKRWASQDYFPSKLGNFYGCLLTCATWEDMPYLVWRPDGSGSFVGIEGAL

LQFMAENLNFTVGLYWMNKEEVLATFDESGRIFDEIFGHHADFSLGGFHFKPSAGSEIPY

SQSTYYFMSHIMLVTNLQSAYSAYEKLSFPFTPLLWRAIGLVLILACLLLMLLVRWRHHH

ELPRNPYYELLVLTMGGNLEDRWVPQRFPSRLVLLTWLFATLVLRSGYQSGMYQLLRQDT

QRNPPQTISEVLAQHFTIQLAEVNEARILASLPELRPEQLVYLEGSELQSFPALAQQSGS

SARVAILTPYEYFGYFRKVHPMSRRLHLVRERIYTQQLAFYVRRHSHLVGVLNKQIQHAH

THGFLEHWTRQYVSAVDEKDESVARIASTSYSTLDGIDGDPSLSESEEDQQVAPVRQNVL

SMRELAALFWLILWANLGAVVVFVLELLLPRIKLRKILRKMKKSTRASATTTSTLSSPST

TKDIPFSCKDGFQDSWPKCSLLVS

>DmelIR7c

MLHSAVHNVSLVYALVWAIDNYYGMATSTPLAVVQFPTSRESRRLHNDLIDAALGRSSGT

GRIQFLLEDDRVEMTETDTDPPPPSGLTGRPIAIWFLDSLRSYFRLEMYLNQLGSPYKRN

GFFLVIYTGLEDQPMESLKIMFRRLLNMYVLNVNVFLQRDGTVHLYTYYPYGPHHCQSSL

PVYYTAFQDLAAPANGFGLTKPLFPRKLTNMHGCEMVVATFEHRPYVIIEDDPKTPGGRS

IHGIEGLIFRSLAERMNFTIKLVEQKDKNRGEILPDGNFTGILKMMVDGEVNLTFVCFMY

SKARSDLMLPSTSYTSFPIVLVVPSGGSISPMGRLTRPFRYIIWSCILVSLIFGFVLICL

LKITALPGLRNLVLGRRNRLPFMGMWASLLGGLALYNPQRNFARYILVMWLLQTLILRAA

YTGQLYLLLQDVEMRSPIKSLSEVLAKDYEFRILPALRTIFKDSMPTTNFHAVLSLEESL

YRLRDEDDPGITVALLQPTVNQFDFRSGPNKRHLTVLPDPLMTAPLTFYMRPHSYFKRRI

DRLIMAMMSSGIVARYRKMYMDRIKRVSKRRNLEPKPLSIWRLSGIFVCCAGLYLVALIV

FILEILTTNHRRLRRAFNVINRYAA

>DmelIR7d

MDIRCVVALLLGLCKVQAVVWPHQHLLEEQLASQISATLQKIFINGLAVYNFGVFISTSY

EEMDRDRVILVHQVLNRNLYPPNFPVAVVLASKMNRKITAQVFTQLLFVQNAEQAIAIAE

GVNRNGLCVIVLLTSQPERPIMTKIFTYFMQERYNINVVILVPRLHGVQAFNVRPYTPTS

CSSLEPVEIDIKDGDLWDVFPRRLKNLHGCPLSVIVWDIPPYMRINWKSSDPMDGLDGLD

GLLLRIVARKMNFTLKLIPNEPNGLIGGSSFMNGTFTGAYKMLRERRANITIGCAACTPE

RSTFLEATSPYSQMSYIIVLQARGGYSIYEVMLFPFEKYTWLLLSTILGLHWIVGSRWRM

PSPILAGWMLWIFVIRASYEASVFNFIQNSPVKPSPRTLDQALSGGFRFITDHASYRMTL

KIPSFQGKTLISAGQPVDVFDALLKAPWKTGAFTSRAFLADHLVRHRKHRNQLVILAEKI

VDNMLCMYFPHGSYFAWEINKLLFNMRSFGIFQHHSQILAWDNLPTTTDTDTPGKRIHSS

TESVATGFAESMSFVVAALNCLMGALCISIVVFGLELLSRRRHWTGLEWLFERV

>DmelIR7e

MNHINEFVARAVLHVVHHYILSVTPSLVLTLCCRSNHTCNFYNKMMSTLFREWGLAPLQI

VNVLRGVPWHPVPGRRHFNVIFTDSFAAFEEIRMEYYSREYNYNEHYFIFLQARDRLLQG

EMRLIFDYCWRYRLIHCSIQVQKSNGDILFYSYYPFGEHGCSDMEPQLINRYNGSMLVEP

DLFPRKLRNFFGCPLRCALWDVPPFLTLDEDQEEVLRVNGGYEGRLLLALAEKMNFTIAV

RKVHVNMRDEALEMLRRDEVDLTLGGIRQTVARGMVATSSHNYHQTREVFGVLASSYELS

SFDILFYPYRLQIWMGILGVVALSALIQLIVGRMLRERMGSRFWLNLELVFVGMPLLECP

RSHTARLYCVMLMMYTLIIRTIYQGLLYHLIRTHQLNRWPQTIESLVQKNFTVVLTPIVQ

EVLDEIPSVQHMRFRLLEANSELDPLYFLEANHQLRQHVTASALDIFIHFNRLSADKVHQ

RGEQGSGAHFEIVPEDIISMQLTMYLAKHSFLIDQLNEEIMWMRSVGLLSVWSRWELSES

YLRNEQSFQVLGTMELYAIFLMVLVGLIVGLLVFILELVSMRSIYLRKLFT

>DmelIR7f

MQGEDANLYVARALRLVIENVLAQLSTTLVVTISTRHLGTAHWFEYMMNILMDSWRMVAV

QLLRIRPDLVVNPVPGRKRVSLLMVDSYQGLLDTNITASNANFDDPDYYFIFLQARDHLI

PKELQLILDHCLAHFWLHCNVMIQTAQVEVLVYTYYPYTADACQKAYPIPVNTFDGRKWK

ASQMFPDKLSQMHGCPLTVLTWHQPPFVELVWDPKHNRSRGSGFEIQLVEHLARRMNFSL

ELVNIALLRPNAYRLAEGSSEGPIEKLLQRNVNISMGYFRKTARRNQLLTTPMSYYSANL

VAVLQLERYRIGSLALLVFPFELSVWMLLLLALLIHLGIHLPSARRGNEEDGGGGLQVVA

LLLGAALARLPRSWRHRFIAAHWLWASIPLRISYQSLLFHLIRLQLYNTPSFSLDQLLAE

GFQGICTANTQRLLLEMPQLARDPDSIQSVDTPFDWDVLNVLTRNRNRKIFAVANQDVTL

SFLHSSAHPNAFHVVKQPVNVEYAGMYMPKHSFLYEKMDDDIRRLDASGFIHAWRRASFA

SVHRKEQVHMTSRRYINHAKLSGIYMVMAGLYLLAGLLFAGEVLLRQRN

>DmelIR7g

MNVTSLLNFESMKYIGAQTQAASINHHVAQALRVFIEDFYQRIAPAFIVVLSCRRPSPMN

FYRNIMQLLYESVDTMIVQLVLVELGRPRRIAGPRTHNLLLVDSLDALLDIEIHTYTAQS

DTSEYYFIFLQQRDALIPHDMQGVFAYCWRHQLINCNVMTQSSGGQVLLHTYFPYAPGQC

NDSQPTRINMFLGESWKHRDYFPSKLHNLNGCPLIVLARKVSPFLDLDEGQRELRGLEGR

LLQELSRRMNFSIQFSGLQDQLKNRTTWTEKQLLQKLVQERIAHLAIGYVRKRIQYATNL

TPVFPHYSNRVVGCLLLNAHNLTSLEIWSFPFQALTWICLVAGDRLALVLAVYAASLGLP

IDPPERPSLQLLFASWLIFGLIVRSMYSALLFFILRYHLHQRLPGNLQDLTHGDYAAVMG

RTTLQDLREVPSLQDLLGLKSVIVTSEREEEVLRTLDRCTLREGAGSHPLFFGLISQDAL

LHLTQRGHRAGAYHIIPQDVLEQQLAIYLQKHSHLASHLDHLVMSIRSVGLVHHWAGQMA

SERYFRSRFLYREKRIRQPDLWAVYILTAGLYLLSLVVFICELLASRRAGL

>DmelIR10a

MAVLGTVFLLFMLDLKTLNLTRLNGLLVEPTRDLPQLELWLRAGSDHQDAENPYVQWFLL

RTEIPLSIVTYQENRYWMDDPFGRRNLVLVMSLDQLLTNRGAAAPIQKASTFFYILADQD

KDLSADEQLRLEGSCRQLWTQHKVYNRFFLTRDGVWIYDPFKRRDSAFGRLVRYYGSETL

DKLLFRDMAGYPLRIQMFRSVYTRPEFDKETGLLTRVTGVDFLVAQMLRERLNFTMLLQQ

PEKKYFGERSANGSYNGAIGSIIKDGLDICLTGFFVKDYLVQQYMDFTVAVYDDELCIYV

PKASRIPQSILPIFAVGYDIWLGFVLTAFACALIWLTLRVINLKLRIVSLGNQHIVGQAL

GIMVDTWVVWVRLNLSHLPASYAERMFIGTLCLVSVIFGAIFESSLATVYIHPLYYKDIN

TMQELDESGLKVVYKYSSMADDLFFSETSPXWNRDLRADVIDEVARFRNKAGVSRYTSLI

LESSHFTLLRKIWVVPECPKYYTISYVMPRDSPWEDAVNALLLRFLNAGLIVKWIQDEKS

WVDIKMRSNILEADAESELVRVLTIGDLQLAFYVVIGGNLLAFLGFLAEHFRWKLQKKGV

>DmelIR11a

MRFAILWLFSGCLLPGIQVGIWVVVRAQPTGRDVLLSRLGNQQNELNTRRLANASSYLTR

NYIANRINTLVVREICVECPYELSERQRQLVDQILASLAPELSVLLHKGTAEETTWEYTL

FVVNDHTAFTGQVFIFPDELLEREFFCIVVVSEIQSRQFVRQTVGSIVKSNLQMHFVNVV

VVAQLEDGTVGTYSYKLFKANCTPGITVRQINHFDRITGKPQQSMPDLYPVRNGHLGDCP

FNVGAAHMPPHLIYKRHKDPPPASNVSIPAEDLAGIDWDLLQLLAKALKFRIQLYMPQEP

SQIFGEGNVSGCFRQLADGTVSIAIGGLSGSDKRRSLFSKSTVYHQSNFVMVVRRDRYLG

RLGPLILPFRGKLWGVIIVILLLAVLSTCWLRSRLGLSHPIEDLLTVIVGNPIPDHRLPG

KGFLRYLLASWMLLTLVLRCAYQARLFDVLRLSRHRPLPKDLSGLIKDNYTMVANGYHDF

YPLELTCRQPLDFSARFERVQRAAPDERLTTIALISNLAYWNHKHPNISRLTFVRQPIYM

YHLVIYFPRRFFLRPAIDRKIKQLLSAGVMAHIERRYMQYENKRKVASNDPVLLRRITKS

IMNGAYRIHGLVIVLATGMFILELLAGRSNGRLRRWMEWVHQ

>DmelIR20a

MLASLNRSTGLSAELLDLYGLVVHFLLSGEHTTLVYFNPAGLDCSWGVLWQRNLTAHPQI

VWQRNYSYPDLYYQFNAKLLVLACLPMDSRAAIQLEILANSLSHLRTVVRLLIEVAGPDQ

VTLARQYLSFCLRRSMLHVELYFRDYHHSLILYSFRAFPSFELVMRWISVGQGVKLFLHK

LDDLRGHRLRVIPDLSPPNTFFYRDARGDNQVTGYLWDFLATFAGRLNAGLEVVRPSWRA

GSASDSSYMLEYSAKGLIDVGLTTTLITKWNLWAIHQYTYPLLVSSWCTMLPVEKPLATP

DLFGRIVCPTLAMTLLLIILVTWLVFRQLRCLTRLKNSRPARIVPHLLTLLLLTTCSAQL

LSLLIFPPYHVRIASFEDLLRGDQKILGMRNEFYNFDGAFRARYAGVFYLIDDPNELYDL

RNHFNTTWAYTMPYIKWLVIKTQQRHFSKPLFRWSKDLCFFDFMPTSVIVAPDSIYWESI

KDFTFRIHQAGLMKHWIRKSFYDMIKAGKMSIKDYSDLETLKPLNIGDLEIVWRVCGAAI

AVASAIFIMELLYFYINVFFNSL

>DmelIR41a

MFIDLSWSLVLSAIVGKYLNESTICIFWNDKFEFQLLHKSDYISFVGINIKSFDDNGGHY

IIDTGLKKKELQNKHLFLDELVIKIIISIEVTHCETFVVFDKDIDRFVNAFNKASVYSIW

RSLHNKFVFAHIANESPESRNHFFEDQPNILFVVRDHSSASSFDIKTNKFVGRKAENPSQ

MILVDRYLASEQRFQFGKSLFADKLNNLQGREVIIAGFDYPPYTVIKHNMSTNAQDMGVS

GESDFKNVYIDGTETRIVLNFCEQFNCTIQIDSSAANDWGKVYPNMSGDGALGMLINRKA

DICIGAMYSWYEDYTYLDLSMYLVRSGITCLVPAPLRLTSWYLPLEPFKETLWAAILLCL

CAEATGLVLAYKSEQALYVLPGYREGWWTCTSFGVCTTFKLFISQSGNSKAYSLTVRVLL

FACFLNDLIITSIYGGGLASILTIPSMDEAADTVTRLRFHRLQWAANSEAWVSAIRASDE

ALVKDILYNFHIYSDDELLRLAQDQHMRIGFTVERLPFGHFAIGNYLGPQAIDQLVIMKD

DIYFQYTVAFVPRLWPLLDKLNTLIYSWHSSGFDKYWEYRVVADNLNLKIQQQVQETMTG

TKDIGPVPLGMSNFAGFIIVWILGSAIATLTFLLELSLTYILKQSNLK

>DmelIR47a

MRQIKLLVWLLVVGVVSSTEQLQFLKNFLEAVHKERSISTILLIQRKVHKNDFLHGLYPI

FWPIICLDETKRVELVNNFNKDFLALVYMESEADTLLLSALAADLNHIRDARIMIWLQMS

PSENFLDRIVFQASKQKFLNLVVIENTLKTRRFYPFPQPKVQVIDKPFEEKEIYPALWRN

FMGKNAIAVPDLVPPRSFNSFDPKTGHRRESGSIYNVFKAFTQRYNITMLLKWPLIRNTT

QEEIIGKSVRGEIDLPITGQLISFRHPNGSRSQPLLGMTALSIAVPCGPELPMFDRFFLF

YGLATPITITGYYVLLNTIEIILGTLSDRIKRHPRRKKILNLVLNLRVFSCILSLPTPQG

NRLRSVKGQLTMVMSITGLILSCIVAAQTSTILTMKPQYRHIKNFQELSDSNITVVCNHL

NYLTIKQQMDPKFMAKFMQNIWIVNSIEQMKMIFDLNTSYAYQTFSYKKDPFTLLQMHTT

RKAFCRTPGLDLVSGLAYTAVLEKNSIYALALQDYTLKAFSAGLVYYWAEESIRDLISTV

GRTQFEKLPIVIGYQSLKLQDYNVCWKILLIGGALAFCVFIVEVVVGLINRRI

>DmelIR47b

MREAQIIIFLLTSAAAVTLKQYEFLXSFLKAGEQEQTITTLLMMQKHVHTKNLLQGLYPX

PWPIIHFVETQRIKFIALLYMSSEKDIFLSSLAANLKFERLDKPFGKSNIFPVLWRNYMG

XIALTLDHLVEPRSFYWTDPRTNIKRRTGYIYMLITNFAEQHNITLQLXSPPNEDMSQMV

IIERTHKGPRSTHNWADDQLETFERXQDSLLPWHGSMAIVVPCGQEMSAYERFHAAHAFR

APIIFFGFHIFLSLIDFLLRTISDRIRCNPRRIQLLQTVLSLCVLRCILSTSLPNSNXLR

SRLRDNSPXXXVLQAXSYSALWXLTGTAXQXHNRDFQSHKLHDYXTTDGSXHSIEVPGLL

KARNXXIXLFHIFSSLGTKFDLRIGSAGSHTSGVEFRYYELLDRXSSLENNIVSQVFTIL

KLPYSRFRVLKLEDCRGCWQTLFVGFSIATFVFIVNVLMGFFRNINQKK

>DmelIR48a

MHLLITETYMIIGKTLHDILNELNERLIISTNIIFCKQFDNLIHFEAQTSRFVYSSLEAF

NITSLWNHVGNDNKLFVIVGNVPPYELFAKLELSSPENCTQFILNNTVDMCADALVKNSK

AFSVSRELRIAPANVIVPHGKPLLSYRYLAAPFNTKVWIALGTYVFLISGFLCLIHWLRS

GKWDFSQNLLEVYSSLLFTVFHLKATNGIERYILFGVLFISGFVYSTSYLRLLKSMLIAE

TFEKQIQTFEELAESNIPLLINPYDRMIFQHHHIPKSLWTAVRTVSSETLLNHRSHGYVR

LCPAILTASKIPSHTHRHLFSVCRFSHEQEVVPKGSSXXSLVPCIRKRNREXNHLGCLSG

VSWPGISXFFHYGALGGEAFGSILLHDANYFPSPRLFRRLAELHYGSY

>DmelIR48b

MILQQSSNLLKLLLLLAISSVRTQGLNDIIIELNQRLLISNNFLYCNQSDKLNEYEIKYL

QHMPPISLMIFTSIESMNFTQVEYNLGADNKLFLIMGNEEPPYDFLHALNLHFQFAEYII

VIDEPVDLKKSTKWLDFVNHLWQQGYVQLLIYTSYDEKLYHKIIFPETVIEETLVEQYIS

IRGSFNNLYGYPVRVAAYNNAPRSMLYVNRWGKHIFAGFYMRFLRAFIDARNGSFVPVLT

PSNSPGNCTLNLVNETVDVCADALAANPAAFSLTHGFRIASANVLVTHAKPLHSYRYLTA

PFQWSVWACLVIYVLLVVNFLSFIGWLRSGKWEFSKYLLEVFSSLLFSGFYLKEIRGRER

YILFGVLFIAGFVYSTEYLGLLKSMLISEVFEKQIDTFEALVESNITLMVDPYDKILFAK

YNMPEILSPIMELVSFETLLKHRNRFDQDYAYILFSDRMALYDYAQQFLKHPKLLRIPID

FSFLYTGIPMRKRWFLKHHLGRAWYWAFESGLTRKLALDADFEAVRVGYLSFLITEHVEA

QPLNVDYFVMPAIALAIGYILALLSFVIEMTAWRIREFLGCRKATMTSTGCSEGGHVDVD

>DmelIR48c

MSLLRIILIIIFLRIVSSIPDTIISHLSAELQIKIQIYFGLGNDLYDFSRLDGNYQKIII

SHNISEEFKTYHDEPVLIIIRLERDLNLNLATLDVLRSYLTDRQYNDILLIDNDEENLNS

YVDIRKAYWNAGFSQVLIYNSQQRTWSIKPYPYLQIRPTSLKEYIENRNTRNLMGYPLRV

LVTNDPPHCFVDKDELPGSPNRYKGSIVTMLKIFADQLNATFQANPFREFRRYSTADCVQ

MVSDDEIDACGSIFIRTYTYATSQPVRLNRVVIMAPFGNPIEKFYYFFRPFDLYVWIGTG

IIVVYIAVMGSLLHRWHFKEWNVGQYLLLAVQTLLNRELSLPQSSSGSKFMLLLLLFAIG

FILSNLYVALLSMMLTTKLYQRPIENLADLKAANVNILLQTHNIRPNSVYGSSEELRERF

LLVEESQHLEKRNGLDPSYAYVDSEDRMDFYLYQQKFLRRRRMKKLSNPVGYTWAVQVIK

QNWVLEKHYNDHVQRFFETGLQNKLVDDVHELAVKAGFLHFFPTQTQTIEPLRLEDIVMA

AMVLGGGHALAVICFLVELFA

>DmelIR51a

MYNVLVLFLLLFTRAQMEPHRRGHNMTLLRSVLTVIRGRENWKNTPIFLGGHCNSDDLNN

LMSWLQNTMEVTCHTVDTSTSAKNENALGHFNINADNSLGLLFCQSSHELIWFNMDKRLR

RLRGIRLIVILSDKRSSSSKAIMSTFKRLWHFQFQXNFQGYVVSTPVENDIPRVFFVKDK

KTGRKQIRGFGYRTFVEYLHRYNASLHVSNSQQEHAINSSVNMGRIINQIVDGQLEISLH

PYVDVPENMGDNSYPLLIASNCLIVPVRNEISRYMYLLLPLNQSSWILLLGSVIYISGVL

YYIQPGLLHRTWDQRIGLNILDSISRIINICSPSRIYNPSLRYFIVSVHLSILGFVVTNL

YSIMLGSFFTTLVVGEQVDSMQQLIQXQQKVLVKYYEVSTFLRHVEPDLVDGVAQLLVGV

NASEQVSALLGFNRSYAYPFTLERWEFFSLQQQYAFKPIFRFSSACLGSPIIGYPMKSDC

HLQSSLNMFIMRIQAAGLLRHWVVSDFNDAMRAGYVRLLENFLGFHSLDVDSLRLRWAVL

LCGWLLSTLIFLCER

>DmelIR51b

MCKVLTLLVVILLLALTNAAYNVTLLKSVLSLISTREPWINTPIFVGHNTQGGDLNDLII

WLHQTMGVTSLTMNLFLQPEHIRPLGHFKITRYNGIALFFCHDKHDIMWLTLDRNLRKLR

RIRLIIILRNQRSGSQGAIKSIFNALWQYQFLNVLVLQRDQLYSYTPYPAMRFFKLDIHT

EPLFPHAARNFHGYVVSTPAENDIPRVFHVHDPLTKSRKVLGYAYRTFVEYLDHYNASLR

LTNPDENLDPTTSVNMNHIVQLIIDGQLEISLHPYVFTPPTATKSYPLLIYPNCLIVPMR

NEIPRHMYLLRPFQLYSWYILLFAVFYITGILYCISPKLNKSSWPQRLGLNFLDAISKIL

FISPPITIYRPTWRHLIIFLQLSVLGFMSTSWYNIELDSFFTTIVVGEQVNSMDQLVHQQ

QRVLVKEYEINTFLRHVEPRLVEKVSRLLVPVNASEQVSALLSFNRSFAYPFTEERWQFF

AMQQQYAFKPIFRFSSACLGSPHIGYPMRVDSHLETSLNHFILKIQDTGLLNHWVVSDFN

DAMRAGYVRFVDNVLGYQSIDVDTLRLGWCVLGIGWILSALVFSCEYWHLYPWRFIA

>DmelIR52a

MALGWSVIILGFIGQLSAQILNYTQSRDLELLEGSLFRVLSRLNLEEEYNTLLIYGKECV

FHSLLRKLEISAVTVPSGSTDYDWSFSTAILILSCGYDAENEENSYTLMKLQRTRRLIYL

EDNSEPESVCMRYSLKEQHNIAMVKSDFDQSDTFYSCRLFQTPNYVEGHFFKDQPIYIEN

FQNMRGATIRTVADSLVPRTILYRDEKSGETKMMGYLGHMINTYAQKLNAKLHFIDTSKL

GAKKPSVLDIMNWVNEDIVDIGTALASSLQFKNMDSVWYPYLLTGYCLMVPVPAKMPYNL

VYSMIVDPLVLSIIFVMLCLFSVLIIYTQHLSWKNLTLANILLNDKSLRGLLGQSFPFPP

NPSKHLKLIIFVLCFASVMITTMYEAYLQSYFTQPPSEPYIRSFRDIGNSSLKMAISRLE

VNVLTSLNNSHFREISEDHLLIFDDLSEYLVLRDSFNTSFIFPVSVDRWNGYEEQQKLFA

EPAFYLATNLCFNQFMLFSPPLRRYLPHRHLFEDHMMRQHEFGLVTFWKSQSFIEMVRLG

LASMEDLSRKRNEEVSLLLDDISWILKLYLGAMFISSFCFILEILRCGERCKRLWRCRW

>DmelIR52b

MTWLVILLCFLGYMAAHIADISVQNQSLMDNELINLLLKLRNEEFYDTLLVYGKDCEFHS

VIKNVDVAVVLVSDSMNFEWNFSSLTLILSCGPDIDNGGPNSTSIKLQRNRRLVLLKEDF

QPSNICNIYTQKEQYNIALVRENFTKSKSIYTCRYFQDPNVDEVNLSGTKPIFIEQFQNM

KGKAIRIVPDLLPPRVMLYQDANDGELKMIGYVANLITNFAQKVNATLQLDFLKPSTSIT

EISRMAKDDELDMGITLEASLNTSNLETSSYPYLLTSYCLMVQVPAKFPYNLVYALIVDP

LVLGIIFVLFLLLSVLLIYSQKMSWQDLSVANILLNDKSLRGLLGQSFPFPLNASKKLRL

IFTILCFASIMLTTMYEAYLQSFFTNPPSEPEICSFQDVGSYNRRIAMSALEVNGLIKTN

NSHFREIRMDDLEIFDNMPECYELRDAFNLSYNYVVTGDRWRSYAEQQTLFKEPVFYFAR

DLCFSRLIFLSVPLRRHLPYRHLFDEHMMQQHEFGFVNYWMSHSFFDMVRLGLTSLKDLS

RPLAYTPSLLMDDISWIMKIYLAAIVLCVFCFLLEIGVDKWKRWMKFRNLQILNTC

>DmelIR52c

MVWLIIILFCLGNSSSQILDVTNNSHLDFDYRLFGLLQRLQVEKSYDTLLVYGEDCAIPS

LFERLQVPAVLVSSGSTNFDWNFSSLTLILSCNFQDEREENYRTLMKLQTSRRLILLKGH

IKPESVCDFYSKKEQHNVAMVKENFYQLEVVYSCRLFQDQNYEKLNLFDGKSIYKDQFRN

MHGAPIRTLSDKEPPRTIPYIDSKTGEEKFKGYVGMLISQFVKKVNATMQIREDLIKDDE

EVSFVDITNFTSNDILDIGICEARTLEMSNYDAISYPYLMSSYCFMAPLPDSLPFSDVYM

AIVAPSILIMFLIIFCICSVLIIYIQERSYRSLTIRSVLMNDICLRGFLAQPFPFPRQYN

RKLKLIFMLVCFSSLISTTMYTAYLQAFLWGPPIEPRLTSFDDVKKSRYTMAINIYEREF

LEALNVSLEDVEIYDYGKFSKLRSTFNTNYLFPVTALQWFTINEEQKLFKYKIFYYCDAF

CLNQFDILSIPLRRHLPYRDIFEEHMLLQKEFGLTKYWIDQSYRDmelIRANLTTFKDFSPL

LENDYIEVHNLYWVFTMYFVGMGMGLCFFILEILRPLRYWRNCKIKCEYCYAFLKNFAK

>DmelIR52d

MVRIIIILLCLGYTKARILDATNTNHTDLEERLLSLLLRLQQEQFFNTLLIYGEDCAFSS

LSRRLQVPTILVSSGSTSFEWNYSSLALILTCEFKAEREENYQTLKKLQMNRRLILLNGN

IKPDSVCDFYSKKDQYNIAMVNNNFHQVGIIYACRLFQERNYEKVYLSEGNPIYVDQFRN

MQGALLKSITFNLIPGSMAYRDPKTGQEKHIGYVANLLNNFVEKVNATLDMQVKLHKAGK

KTSFYNITKWASEDLVDIGMSYAAYFEMTNFDTISYPYLMTSTCFMVPLPDMMPNSEIYM

GIVDPPVLVVLIAIFCIFSVMLNYIKQRSWRSLSLVNVLLNDICLRGFLAQPFPFPRQSN

RKLKLISMLVCFFSVITTTMYTSYLQSFMWGPPIDPKMCSFADLENSRYKLAIRRYDIEM

LRPFNVSMDHVVVFDESSQLEYLRDSFDDNYMYPMSALSWSAFKEQQKLFAFPLFYYSEK

LCLKPISFFSFPIRRHLPYRDLFEEHMLQQNEFGLSTYWIDRSFSDMVRLKLATMNDFSP

PRLEDYIEVSDLSWVFGMYFTGLGISCCCFGLELLGLPSWTRRLRLTNWLRVRN

>DmelIR54a

MWTVITGIVLWAPVLVAGSAVDFIFRAAAEHSLSVIMIRIDYCPYNWAKDIFENQTIPVV

VLSDSETFINIRMFSRPLHVACLPGHELQKDLALLENFTSSLMDFPSQKKIVYISNNFSD

PTRMDYIFETCYHRRIWNIVGLLASDEHRYFYRYHLYPSFRTEYRSLESSTIFDKDFPNM

HGHPLTVMPDQWLPRSVLYVDRRTGKQILAGSVGRFFHVLSWKLNATLQLSKKVTTGRFL

NATALKELSESFSVDVPASLTIMERVEQLASTSYPMEVTHVCLMVPVARRIPIKDIYFIL

SSASNMFLAIVIVSSYGLALNLLRNMTHRDVRLVDFVLNDKALRGILGQSFNLPLSRSFS

TRLIFLMLGIVGLNVSSIFGAGLDTLMAHPPRQFQARSFAGLRRTKIPLVTTEEDFPTWM

KLRVPMLVVNVSEYNHLRNGRNTSNAYFASRLYWNLFSEQQKRFTRELFIYSTDDCLWSL

ALLSFQWPQNSLFTEPVSQLILEVNANGLYDFWVGMHYYDMTAAGLSGLEDPSLQLKERE

HPTSLRIVDFQWMWQAYGTFMVIAILVFLLEVSWHRITSLFVSLVY

>DmelIR56a

MGSRFFIRNLILFGLLASSNMQIPFGELEKKFELDVDFLLGVTELVGHIQGLYSITVYAD

CIDIHPSIQQRIMDKFMVPVNTIGSNLSRPNYHKLDNSRIRIVLFTGLNDTILVNLNKTD

VPYSDNFYMLAYASAIKNKCIELDFIEEVFTLLWKMSIQNAILLIRGEFMMEMWSYLYMG

KIHKIKLTKPNSYLESLRKYNYRFSLEVINDPPAIFWYNSSEQADVTGGGNLSVSGPLGL

IIINFLRHLNVTIDIVPIPGKQTSQYELFQQPDNLRAENGVNMVGSALLKYSPMVTQSRM

CLLVSNRRMIPFSRFLDRLVSPGVHKLTFVSSIGIFVIKYFSHRPRSFVDAIFCTIRFFF

AIPLPSIILNRLPVVDRFIEVFIIIFVQILLSSNISITTSALTTGFWEPPIINVETMRAS

GLHILTEDPTILQAFKENILPSSLADLVILVDEDTYFHHVTTLNNSYVYVVQAHNWQIFR

LYQQQMTNEPFEIASEELCSKWRILGIPLNPKSPLRFMFKDYFYRILESGLREQWVHSGF

KKFCEFNNLKKLPVDSVDSWQPLSIEFYSNVIRAYIIGLVIATLAFVAELLHNGYRRKNV

KKT

>DmelIR56b

MLLDTDLASGVIRSPYSFDIPHAFIFNETQFVVPKFCGPYMEIVKHFAEVYHYQLFLDSL

ESLPKKSVVEQDIISGKYNLSLHGVIIRPEETSDFFNATQHSYPLELMTNCVMVPLAPEL

PKWMYMVWPLGKYIWTCLFLGTFYVALLLRYVHWREPGNATRSYTRNVLHAMALLMFSAN

MNMSVKLKHASIRVIIFYTLLYIFGFILTNYHLSHMTAFDMKPVFLRPIDTWSDLIHSRL

RIVIHDSLLEELRWLPVEYQALLASPSRSYAYVVTQDAWLFFNRQQKVLIQPYFHLSKVC

FGGLFNALPMASNASFADSLNKFILNVWQAGLWNYWEELAFRYAEQAGYAKVFLDTYPVE

PLNLEFFTTAWIVLSAGIPISSLAFCLELFIHRRKQRRPQYERFECYDY

>DmelIR56c

MRSSFRICLFLLTTYHPSHGWNMQHLLNLLAPFGRMNVFQEIVWFVSPHQRLDQLDEFIM

RIDEAFGKSATQTVVNNNTEMRMIYSSARRNHMSFVFTTGAEDPIMKVFSKVLLGRHFYV

SMVIYVDKVGDMHPIYDLLTFAYNQQFFNSMVHFESMEGVNQLFGVSKFPVMSFENRTDF

LKYMGKIWKQVQNARSDVGGFGFTTPLRQDLPHLFQSQGHYDGSTYRIIETFVRFINGSF

KELIMPPDSLGGQVINMKDALQLIRERKMEFCAHAYALFMSDEELEKSYPLLVVQWCLMV

PLYNSVSTYFYPLQPFDWNVWFFALGALLALVLLELMWLRMFGGWSGYRGAVLNSFCYII

NVPIEGQLQQPCLLRFLLLATVFFHGFFLSAYYTSNLGSILTVNLFHAQINTMNDIVSAQ

LPVMIIDYEMEFLLNLNKELPQEFLELLRPVDSAVFSEHQTSFNSSFAYFVTEDHWEFLD

EQQKHLKQRLFKLSSICFGSYHLAFPLQMDSSLWRDIEYFTFRIHSSGLLNFYARSSFGS

ALHAGLVQRMPDTQEYTSAGLQHLAIAFILLLVMSFLAGIVFVLETLSR

>DmelIR56d

MDNRAAELILRERNIFPTNGSDNITLLNNMFVLEMFYRITQLYHFKNFIFYISERLDLNN

KDSQEFFHNFWTYFPMAPNLIITREHHLGIPMMQFISTPSLVMVFTTGKDDPIMELASHN

QQGIHWLKTIFVLFPSLQSRDFETNPESLAQFTAEIKDVYDWVWRKQFINTFLITIKDNV

FILDPYPTPSIVNKTGVWQAEEFFHKYAKNMKGYLVRTPILYDMPRVFKSDRPTNRYEKN

FIHGTSGNLFLGFLEFVNATLMDTSANVTADYLNMTNLLDLVSQGVYETLIHSFTEITTK

FVVSYSYPIGINDCCIMVPYRNQSPADQYMHEALQENVWVLISLFTLYITVAIYLCSPLR

PRDLSAAFLQSICTLTYSVPTFIIRTPTLRMRYLYILLAIWGIVTSNLYISRMTSYFTTA

PPVRQINTVQDVVEANLRIKMLAIEYERMAKSPLQYPESYLNQVDLVDKHMLDLHRDPFN

TSFGYTVSSDRWRFLNLQQLHLRKPIFRLTEICEGPFYHVFPLHKDSHMRSVMTEYIMIA

QQAGLMNHWERETFWEAVHLHRIHVHLFDDEPMALSLDFFSSLLRTWTLGLILAGLAFAA

EMKWHEHVTFKRRPVIRITRKPRSFLRRFMKL

>DmelIR56e

ERXAFRNQWAFCFPRTXAIEVVLSAWSPXCPGQRSKPQPISXPHHXGSCWRKRKWKXKPR

LLVVDKRTLVEHLNSLNDGYAYCIIAGHWQVGMM

>DmelIR60a

MWCNNPGLIIIIFLGQILNLCQGIVNLSNETANTVIFMLPEKDLGPDVWKAGVGCLDSFA

QIFFFRNPKERFTRAYNLMLVHAFHLSSPADQIQEGFSKLINEAVTNPGPPDREELFQMR

VASDYNITNGTEDKGELILADNYVIVVDSVDRLKELMKKKIVEMRSWNPGARFLVLFHNA

TCRNRPLGVASNIFKDLMEMFYVHRVALLYANSTMNYNLLVNDYYSNVNCRILNVQSVGQ

CHDGKLYPNNAVVKASMQDYVSGFSPRNCTFFACSSISAPFVEADCILGLEMRILGFMKN

RLKFDVNQTCSLESRGEMDGPANWTGLLGKVQNNECDFVFGGYYPDNEVADHFWGSDTYL

QDAHTWYIKMADRRPAWQALVGIFEAYTWIGFILILIISWLFWFTLVMILPEPKYYQQLS

LTAINALAVTISIAVQERPICETTRLFFMALTLYGLNVVATYTSKMIATFQDPGYLHQLD

ELTEVVAAGIPFGGHEESRDWFENDDDMWIFNGYNISPEFIPQSKNLEAVKWGQRCILSN

RMYTMQSPLADVIYAFPNNVFSSPVQMIMKAGFPFLFEMNSIIRLMRDVGIFQKIDADFR

YNNTYLNRINKMRPQFPETAIVLTTEHLKGPFFILVVGSCWAALTFIGELIIHRWRTQLV

STSEQQDRRSDKRRRRRRRRKPEKDNRWQRQVQVAPVVRFTPVKRRKVFQGQTSQK

>DmelIR60b

MRRSLYLIIAIGLVDVHCVSLRYILNALENELQYRAILLVESASEIESCWEQKYIQGAVP

ILNFNANQSLYLKDALNTNILALVCLNENVESTMQALYENLEDMRDTPTILFVLSDSKVQ

DVFLECLRRKMLNVLAFKGLDRGFVYSFRAFPTFRVIERNVMDILQYFEQQLEDLGGHTL

TTLPDNIIPRTVVYKSPDGSRQLAGYLYPFLRNYVSTINATLKVCWHLVPEDGMIQLGEV

VRLSEIHDVDFPLGMHGIEHGSTSQNVPLEVSSWFLMLPMEPSLSRAQFFIMLGFEKVTP

VLLLLTILLSTAHRIEMGLRPSWRCYVLGDRVLQGTLGQAFFLPRRLSVKLMLVYSLILL

NGFTFSNYSITSLETWLVHPPSGHPIHSWEQMRTLNLKVLIVPSELDSMTKALGKQFTES

NSDLFELSKSGNFQDKRLAMDQSYAYPVTCTLWPLLEHAQIRLPKPEFRRSREMVLIPLL

IMAMPLPKNSMFHKSLNRYRALTHQSGLYEFWFKRSFNELVALRKIHYKVNGDHQIYRDF

EWQDFSYVWLGFVGGTIASILVLLAEIGYHRWQLNQN

>DmelIR60c

MEMRLALFFTFACLAGAHDGSLRNMLKSLEDELGYRTILLLEGFVYSFKAFPTLRVVKRR

VKDVRRYFEPQLEDLGGCVLKVVPDGIMPRTMVYQGEDGELQMGGYLSHFIRNYVSTINA

SLQIRWDLFPEDGDFDMDSLTGSNHVDFPLGLGSLSFQTLHKDVAMEISSWFLMLPMEPS

LPRARFFIRFGISLYLIPLIILLAIVLSNAHRFEAGLTPSWRCCSMGNTVLRGVLAQAFV

LPKGLSPKLMFVYWLLLVSGFFVSNYVIVYLTAWLIQPPTSDPVTDFDQMRRAKLKILMV

PTDMDYLKSIRGAEYVDAHSDVFQTADSTDFQTQRMSMELHFAFSVTGTLWPLLRQAQVK

LHRPIFRRSKEMVFLPFVIMGMTMPNNSIFLSSLKQYRLRTSEAGLYLLWFKKSFSELVA

IHKISYKEDWVHDSYSDLKWEDFLFAWLGFLGGTTVSCLALLAEIGYHRWLWKRTHQ

>DmelIR60d

MRLAIYVAFLSSIGNRSGFLSSLLMSLGKELHYKTILLVGGSSTCWSLEPFETGVPILNL

RGENNAYPQDTFNSQMLALACLQTESEDAVKLLYRSLKDMRDTPTLLFASSEEHIHDTLF

LGCFRENMLNVLALTASSKEFIYSYQAFPTFRVIKRKLVEIHRYFEPQLKDLGGHIVSAL

PGNIMPRTMCYRNAEGERQLAGYLNTFIRNYVESINGTLRISWGLVPEDDMRHLTISRLS

KIQHVDFPLGIIPLYNKTDKQHVYMEISSWFLMLPMETSVPRAHLFVKLGLERLLPIIVV

VGAVLGNAHRIEVGLGPSWRCYYLADKVLRGALAQPIVLPRRLSPKLMLIYSLLLLSGFF

LSNYYMASLTTWLVHPPASDRILEWDQLRYLHLKVLTIPEEFKYMSLILGTDFMTAYGSI

FQLTNSTDFQRRRISMDPSYAYPVTTSLWPFLELSQVRLRRPLFRRSYDMVLQPFQVMSL

PLPRNSIFHKSLLRYAALTRETGLYYYWFRRSYYELVALGKISYKEEEGNPYCDLKWNDF

RIVWLAFLGGTIISCLALLLEVAHYRWHLGNSSL

>DmelIR60e

MVIKMISFLLVSVLLCLVGASDSESMQVQVLQDLNLALQTELNVFIDFECCATSEILHKL

DSPRILLSSNSREARDLRIRGNFTESTLIIVSVMDSDLNPLVASLLPRLLDELHELHIVF

LSNEEPGFPKQDLYTYCFKEGFVNVILMSGKGLYSYLPYPSIQPISLSNVSEYFDRARII

RNFQGFPVRILRSTLAPRDFEYSNEQGGLVRAGYLFTAVKELTYRYNATIESVPIPDLPE

YDVYLAVAEMLHTKKIDIVCYFKDFSLEVAYTAPLSIIREYFMAPHARPISSYLYYSKPF

GWTLWAVVISTVLYGTVMLHLAARGARVEIGKCLLYSLSHILYNCHQKIRVAGWRDVAIH

GILTIGGFILTNVYLATLSSILTSGLYDEEYNTLEDLARAPYPSLHDEYYRSQMKAKTFL

PERLRRNSLSLNATLLKAYRDGLNQSYIYILYEDRLELILMQQYLLKTPRFNMIRQAVGF

TLESYCVSNSLPYLAMTSEFMRRLQEHGISIKMKADTFRELIHQGIYTLMRDDEPPAKAF

DLDYYFFAFVLXTVGLISSLLVFFAELVSGHL

>DmelIR60f

MRFHLNIANSGLLGLHLCPTRSALPEQNPCFSKAGAVIXNLTLPWRRWRERCLLGALRPX

TLPTPELQCXSKYLPXRKSQQENASSGLPGFCXGDXQTELHRGSRAIALPRSPYHDLYYV

WIAYLGGTMIGIGMLAVEIACFKWDLLRRPPIXMY

>DmelIR62a

MYLQFLFALFLSRYQIVATENFDRAFELALFLDRIGRVHRLHAITIVNSLGSVDPSYLDD

LHRGLMCNSSNHFYMLPQMTATDKDSSHVHFSSLQDEETIYLVFARDSKDAVIYLQAERA

RGRRYTRTMFLLRKQESQKDIKYFFELLWKLQFRSALVVVAARNFYQMDPYPTVRVIRMR

RLSSYDPHHVFPPANRKNFRGYRMRLPVQQDVPNTFWYKNRRTKAWELAGLGGILINQLM

MHLNVTMDLFRFEVNGSSLLNMAALTDLIVKGKVELSPHLYDTLQSNTSVDYSYPTQVAP

RCFMIPLDNEISRSLYVFLPFSLTMWLCLLFVLLVVHFVYVRRLIPDGHFWAILGVPGAG

QVRYGNRKPVRRFSTFLILFGIFILGQTYSTKLTSSLTVTLIRRPDNSLEELFLLPYRIL

VLPTDVYAIVDSLGHAEQFSTKFSCTDAENFSQKRISMHPEYIYPISTIRWRFFDMQQRF

LRKKRFYFSKICHGSFPYQYQLRVDSHLKDALHRFLLHVQQAGLHDLWLDTCYRKAHRMG

YLKDFSTLAELEEKLRLRPLALNLLVPAFSLFLCGMLGSGIAFLVEIRHSFGCRQKPPSI

NRNPGD

>DmelIR67a

MLPILVPVLLLFNETSWINPILTSIYKDRHHETVLLLQHSQHGNASGLERFPWPVFSFNE

QMDFYVRGKYNSEMLVLIWQTGNSDWDLDLWQALDRSLLNMRKVRVLLLRKWEKIPTADV

AATAEHLLFLHVAVIGQGNRIYRLQPYAPQSWLQVDPIESPIFIKIRNYFGRYIVTLPDQ

FPPRSIVYRNPKTDEIQMTGYVYKFLLEFIRIYNFTFRWQRPIVQGERMNLILLRNMTLN

GTINLAISLCGFETPSXLGVFSDVYDMEEWYIMVPRAQEISIADVYVVMVSGNFLIVLII

FYFIFTILDTCFGPLLLKERVDWSNLMLNERMISGIMGQSFNMSARNTISSKVTNATLFL

LGLVLSTLYAAHLKTLLTKRPTSQQISNFKQLRDSPVTVFFEEAERFYLKHAWDRPIRYI

KDQLNFRETIEYNALRMGLNRSNAFSALTSEWMIVAKRQELFKQPIFTVQPELRVIQTSV

LLSLVMQSNSIYEDHINDLIHRVQSAGIVEYWKHQTLREMITMGMISQKDPFPYVAFREF

KVGDLFWIWLLWVSFLFMSFVIFLCELLVDCFISKTLIRNKRPH

>DmelIR67b

MELLYLNTLQSLSLLEGNRLVQTVQELNNIYQTELNVFLEFGNGADILESAQGTFVPTLW

IKNPQNQKVMKGNFTSCTLTILYLEDEHLDRGLYYLANWLWEYHHLEVLIFFNGGSYDKL

IQIFSRCFNEGFVNVLVMLPGSDELYTFMPYQDLKILNLKSIKEFYSLSRKKMDLNGYNI

TSGLVIAGAPRWFSFRDRQNRLILTGYMLRMIVDFTNHFNGSVRLMNVLTVNDGLELLAN

RTIDFFPFLIRPLKSFSMSNILYLENCGLIVPTSRPLPNWVYLLRPYAFDTWIAWLIMLI

YCSLALRILSKGQISISAAFLKVLRLVMYLSGSRDMGTRPTTRRLFLFVILTTSGFILTN

LYVAQLSSNSAAGLYEKQINTWEDLDKSDSIWPLIDVDIKTMEKLIPDRTKLLKKIVPTL

EADVDTYRRNLNTSCIHSGFFDRIDFALYQQKFLRFPIFRKFPHLLYQQPLQISAAFGRP

YLQLFNWFVRKIFESGIYLKMKDDAYRHGIQSGLLNLAFRDRHLEVKSNDVEYYYLIAGL

WFGGLTLATVCFLLELLIGYAKIKVTISCKMNIM

>DmelIR67c

MFCWLIFLNIILLSDRSESWSAREVIHQFNHDQQLQLNIYLDCNDVELQIGQEVSNLFVN

STADKMKILGRFSSHSLIIACFKDSTRNRTLNGVKELLWGLQYLPILFVVDSNMDFYFQQ

ALRHGFIHVLALNFMNGSLYTYKPYPKVEVHQIKDMQKFYKLTKLRNLQGQAVRTTVETM

TPRCFRYRNRHGQLVYAGYMYRMVKEFISTYNGTEEHVFGNVDTVPYKEGLAALKNGEID

MMPRIIHALEWYYFYRSHILYNIKTYIMVPWAEPLPKSLYFIQPFRGTVWITIMVSFVYA

SIVIWWIRYRQQGNSSLTQSFMDVLQLLFQLPLSKIWHFNMGTHQVVSFIVLFVFGFMLT

NLYTAQLSSYLTTGLFKSQINTFDDLFREKRTLLVESFDAEVLHNMTKEKIIQKEFESII

LITSIEEVFKHRKSLNTSYAYEAYEDRIAFELSQQRYLRVPIFKILKEVYDQRPVFVALR

HGLPYVELFNNYLRRIFESGIWIKLQEDSFLEGIASGEISFRKSKSREIKIFDKDFYFFA

YILLGMGWCVSTIALFLELWSFKYSVTNVLHEG

>DmelIR68a

MRCLWILIVAFISLAMATSIPIPIANPAPLSGYEMQLKILLQKILWVANVKRCFAVITDD

LHYPIYDRIFFESVGRRVIPFFVMRTNESDDLQRPSRQVELFVKAIKSSDCELNVITILN

GWQVQRFLGYIYDNRSLNMQKKFVLLHDLRLFESDMIHLWSVFIDAIFLKRQLDNKYTIS

TIAFPGILSGVLVMKNIANWELGKGLNGRILFADKTSNLFGTSLPVAISEHVPMVLWANA

TKSFQGVEVEIMNALGKALNFKPVYYKPNQTENMDWTELDGGASVAYGSGNPDGYAQNGT

HIDSMLVDEVAAHSARFAIGDLHLFQVYLKLVELSAPHNFECLTFLTPESSTDNSWQTFI

LPFSAGMWVGVLLSLFVVGTVFYAISFLNAIINGNVSSEFFRCLRPNRNVPMDPKIYRRI

SFRIAISRYRSSKGDRMPRDLFDGYTNCILLTYSMLLYVALPRMPRNWPLRVLTGWYWIY

CILLVATYRASFTAILANPAARVTIDTLEDLLRSHIPPSTGATENRQFFLEANDEVARKV

GEKMEVFGYSDDLTSRIAKGQCAYYDNEFYLRYLRVADESGSALHIMKECVLYMPVVLAM

EKNSALKPRVDASIQHLAEGGLIAKWLKDAIEHLPAEALAQQEALMNIQKFWSSFVALLI

GYVISMLTLLAERWHFKHIVMKHPMYDVYNPSLYYNFKRIYPQH

>DmelIR68b

MKFLVGLLLQWYLPGIYALAEIACRIAVEQNVQVTYLYRCASCPASFDADYSALELDLYR

CVGSRLPVITRNMEAHELEPFRRTDSLSIFQIPAAEKGDSLVRRILDMLNPHQRRKHMHK

YLFVWPNAGRHQLLRLFRGSWAKKLLYGLAITGRENGTFDFDPFAWGGLQVIQRLDGEVP

YARKVKDLRGYPLRFSMFTDPLMAMPRSPVETAGYQAVDGVAARVVGEMLNASVTYVFPE

DNESYGRCLPNGNYTGVVSDIVGGHTHFAPNSRFVLDCIWPAVEVLYPYTRRNLHLVVPA

SAIQPEYLIFVRVFRRTVWYLLLVTLLVVVLVFWVMQRLQRRIPRRGVIQFQATWYEILE

MFGKTHVGEPAGRLSSFSSMRTFLMGWILFSYVLSTIYFAKLESGFVRPSYEEQVDRVDD

LVHLDVHIYAVTTMYDAVRSALTEHQYGLLENRSRQLPLGIATSYYQPVVRRRDRRAAFI

MRDFHARDFLAITYDSQAERPAYHIAREYLRSMICTYILPRGSPFLHRLESLYSGFLEHG

FFEHWRQMDLITRVGASPDAEEFLEDLGDQTDTDSGSNELAIRNKKVVLTLDILQGAFYL

WSVGIGISCLGFAVEHAHWFWRRQTLRNAVEARTS

>DmelIR85a

MSIQWLKHILLLAILVNLAGTRENHIPLDLKKSSIVMVKMSQILCKARIKVLFVYFENQT

SHEHTGQILKEVTKCDISNQNTPLEAVKDDGILMYMVMITTNISQPLELSLIRKKSAAKH

RSHVFLLVRDADTVSDAWMRASFRQFWKIWLLNIVILYWRDGRLNAYRYNPFMDNYLIPV

DNKPNEVPTLEQLFPKTIPNMQRKPLRMCIYKDDVRAIFWRQGTILGTDGLLAAYVAERL

NATMMITRPHSYNNHNLSSDICFLEVAKEYVDVAMNIRFLVPDTFRKQAESTVSHTRDDL

CVIVPKAKTAPTFWNIFRSFGSLVWALILVSVLVANVFCYILKSEVGRVPMQLFAGALTM

PMTQIPPNHSIRLFLIFWLYFGLLICSAFKGNLTSMMVFQPYLPDINQLGALARSHYHII

IRPRHVKHIQHFLTLGHKHESRIREQMLEVSDTQMYEMMRNNDIRFAYLEKYHIARFQVN

SRVHMHLGRPLFHLMNSCLVPFHAVYIVPYGSPYLGFLDSLIRSSHEFGFERYWDRIMNS

AFIKSGVKVVNRRRGSGNDEPVVLKLQHFHAVFALWLVGIGMACIVLAWEHLTHNYNLAV

TKRRD

>DmelIR87a

MSTPEQRFWLAALLFLLSQHSEVRGFGINLMKVQTEDKGQEACILALLRKYFDSGDGLSG

SVLCINRNYQLPNIEEQLLRGVNNYENYPWSLLITNSREGPSPAKFLMNEKPQCYFLIVD

NLEDEDLDEVFEHWKGMVNWNPLAQFVVYLASLEETDEEMNDLMVELLLTFINKKIFNVN

VIGQSEENQFYYGKTVFPYHPDNNCGNRVISVELLDACDYPSEETDSEDENDEDEGDGAQ

EEDDGPQEEGDGEQEEEDGPQEQEDGDQAKGDEGQENDDGGLENKVENEFRIGASDDDEL

ENDLSSNSSEPEAIIEEFFRAKFEDKFPRDLSGCPLTASFRPWEPYIFRNSEEQPVDDYY

YGLQGDEDDYNDTSPNYGESDDESYADPGEDGDGAIPDTETQSGGKLKLSGIEYEMVQTI

AERLHVSIEMQGENSNLYHLFQQLIDGEIEMIVGGIDEDPSISQFVSSSIPYHQDELTWC

VARAKRRHGFFNFVATFNADAGFLIGIFVVTCSLVVWLAQRVSGFQLRNLNGYFPTCLRV

LGILLNQAIPAQDFPITLRQLFALSFLMGFFFSNTYQSFLISTLTTPRSSYQIHTLQEIY

SNKMTVMGTSEHVRHLNKDGEIFKYIREKFQMCYNLVDCLNDAAQNEHIAVAVSRQHSFY

NPRIQRDRLYCFDRRESLYVYLVTMLLPKKYHLLHQINPVIQHIIESGHMQKWARDLDMR

RMIHEEITRVREDPFKALTFDQFRGAIAFSGGLLLVASCVFAFELCYVKYVYRTEKRERK

TKKITKKVHNIKIQHD

>DmelIR94a

MALPKQLKFINIFLVLLIIYGSSDGTENQHEIFLNRLLQAVHNERSVETLFLLHHSNLAN

CSLQDWNPPRIPTIRSNELTVFNVEKTFNHNALALVCLMKNSYREILNTLAKSFDCMRQE

RIILMIHRKSDSKFIEDITHEVKNLQFLHLIVLIVQEKYNGQVFASTLRLQSFPEPHFKR

IRNVFAIQRIFYRPINFHGKVLNAIPNDIPILFVALNEMFTEYARRYNSTLRIQNRTIKE

DIEITEDNYDIDMKIQLHNSQNFLHHMNIAMDIGSNSLIILVPCATELRGLDIFKELGVR

TLTWLALLFYIIFVLVEMLFVFISNRFNGRNFTMRYTNPLINLRAVRAILGQTSPISNRY

SLSIQHFFVFMSLFGTLFGGFFDCKLRSFLTKRPYYSQIENFSELRKSGVTVVVDHTTRQ

FIEQEINANFFRDEVPNVRTTTIQELINHVYSYDRKFAFVANSIPWRTFREEMKSINQKI

LCDSKNLTILENVPLTFSIRRNAIFSHHLRNFIINAADSGMITCWFKMAGKVIRKHIKTT

LRESEQQPSHLPLSFDHFKWLWAVLCIAYVMSFMVFVMEILWSKYQRRTRSVSIV

>DmelIR94b

MSLIFNLLFILILSQAVSQETEFLQLKYLNNIVRSMIKLHKMETLVIVKHHLDNNCSLQN

WNAHGMGIIRTNDQGKLIMKDTFNSRTLAIICIGQNSHITLLRNVFETFGKVQQKKIILW

TQMELKEKFFQEISKKSRDLKLLNLLVLKAVTKDKLLIYRLNPFPSPHFKRIENIWTPND

TLFMDTKFNFHGMTAVVKHDYNWTIQMGNIRKFPISRIEDKEVIEFALKYNLTLQFFNDV

ERFDIELRKRIILKSNSTQPIDSGIPMVFSSLLIVVPCGNYLSIQDVIKVSGIEKWIFYI

ILVYVIFVLIEITFLGVTILISRQSRHQMIPNTLVNLCAFRAILGLPFPETRRTSLSLRQ

LFLAIALFGMIFSIFINCKLSSMLTNPCPRPQVNNFEELKTSGLTVVMDHDAENFIEKEI

GVDFFNQYMPRKVTLTFTERAKLLFSLKGNHAFTLFSESFAIIESYQRSKGLRAHCTSED

LIVAERVPRIYILENNSILDRPLRRFIRQMQESGITNHWLKNIPSSLEKNLMQITIPYDR

ERVHPLSIEHLTWLWCILILGYSISMIVFFVEMSLKRRKKNLENRAPNICIC

>DmelIR94c

MSKVFKLLVLPLIYLSLTKGSKNPQLKFLRELINVIEEGREIRTIMVIKHSRDEYCHLDQ

WNPRGSPILRTNEMGSIRISGYFNDQAVILACMGENSDYGLLKSLANAMDNMRQERIILW

SEREPTKMLMDYISQQADRYNFAQIIIVTMNEDVDAVPSLHQLNPYPTPRFRQITNISNI

RRTSFFGCGLSFQGKTAILKESVVSNIRFKVWSPSGPIPLSELKDYEIVQFAVKYNLSLK

LYDQNESKSDHFDIQLGPLFITKDFPTQMAFVSPNTACSLIVIVPCSPKWRFMDVLHKLG

VLKLIGCLLIAYAVFVLIETLILWLTHRISGREVRLTSLNQLLNPRAFRGILGLPFPEFR

RSSISLRQLFLVISVFGLVYSNFVSCTLSALLTKPAQNPQVRNFKELRDSGLITIMDKYT

HSFIEKHIDPEFFDHVLPHYLILQKKEALRMIWNFNDSYSYVMYTTTWKSLNTVQKSFDE

RVFCESESLTIAWNLPRMYVLGNNSVLKWMLSRYITYMPQTGIPDSWTEQLPKVLKLLYN

VTSPRRIKEGAVPLSIQHLSWIWHLLFIGESIATLVFIVEILLQKSNQHTSNMRERSSED

DDFV

>DmelIR94d

MGQLHLLLVALVLLSPGGDSFYHSLIHHLNRELKIEYVLLLGNFDTTWLDILWQLPVSVL

QIKEHSRETYSLLENPSHNVLTIAFVNDSPEDILEILYRNLRMLNTQPVLLVIRKSTIRV

NSLLEWCWHHQLLKVVAIAQDFMESLIVYSYNPFPVLQFIERRLDNSTVIFEKRLENLHG

YEVPIALGGSSPRLIVYRDLEGKLIFSGPVGNFMKSFEQRYNCRLVQPYPFDESAISPAR

DLIASVQNGSVQIALGAIYPQVPYTGYSYPIELMSWCLMMPVPEEVPHSQLYSMVFSPMA

FGITIVAMVLISLTLSMALRLHGYRVSFSEYFLHDSCLRGVLSQSFYEVLRAPALIKAMY

LVICLLGLLITSWYNSYFSTFVTSAPRFPQLTSYESIRHSNIKIVIWKPEYEMLLFFSEN

MEKYSSIFQLQEDYKEFLHLRDSFDTRYGYMMPMEKWSLMKEQQRVFSSPLFSLQDDLCV

FHTVPIVFPMVKNSIFKEPFDRLILDVTATGLLSRWRDMSFTEMIKAGQLGLEDRGHPKE

FRAMKVGDLIQIWRFVGWMLGLATIVFLLELICFWRHKMWQNMKYMFCRNKNI

>DmelIR94e

MDCPKWILSGLCLISLVSGATVIELLGTLKLELDFEYVLLMKNRNFSLSDQVWNGTSLTK

DVMDEVQVPVLQFNENVSYFLHNSISRRLVTLGFMSDANLDEHRGLLTALVANLRHMTTS

RVIFLVQSKASTDFLYELFRNCWRKKLLNVIVIFQDFETTSTFYSYSNFPILQIEERIYE

TSLQTLPIFPDRLRNLHGYEMPVILGGTAPRMIAYRNKKGNVVYDGTVGHFMTAFQQKYN

VKFVQPLQAKNPLDFAPSMQTVGAVRNETVEISISLTFPTIPPFGFSYPYEQMNWCVMLP

VEADVPPFEYYTRVFELAAFLLTLGTLVLISCLLASALSLHGYATNISEFLLHDSCLRGV

LGQSFVEVFRAPTLVRGIYLEICVLGILITAWYNSYFSSYVTSAPKQPPFRTYDDILASK

LKVVAWKPEYAELVGRLLEFRKYETMFLVEPDFNRYLALRDTLDTRYGYMITTNRWVLIN

EQQKVFSRPLFQKRDDFCFFNNIPFGFPLHENSVFMEPVQKLIMELAETGLYYHWITTGF

SELIDAGEMHFVDLSPHREFRAMQIQDLQYVWYGYAFMVVLSSLVWLLENLAYTVKSKTI

FPTHFMQRNKK

>DmelIR94f

MSGMWQQVLLAETSNWFRSDVLQRFWTHLRVEIRFRTMLNYRLESCDCWFDNVLGSDNST

ALLWNDQTYPHYLRRRQDTDILVVSCLRFHQYQEVLLALSLMLDQMRSMPVVLQLCGDED

SMQELNSARLLLKHSQDLKMPNVVLLSSTFFTSATLYSYEMFPEFNVQKLVYQAYLTLFP

YKLGNLKGHPIRTVPDNSEPLTIVRKTLNGSIAIDGLVWQFMIEFAKHINATLQLPIEPH

PEKSIKLVQILDLVRNQTVDIAASLRPYSLNVQRSSTHIYGSPMMVGNWCMMLPTERVIG

SHEALTRLMKSPWTWLILLLFYSVHRFLAQKTRLRSSLIHLIKLLINLSLICFLQAQLSA

YFIGPQKVNHISNMQQVEESGLKIRGMRGEFMEYPIDMRSRYASSFLLHDLFFDLAQYRN

SLNTSYGYTVTSVKWELYKEAQRHFRRPLFRYSEEICVQKLSLFSLIQQSNCIYCYRSRI

FILRMHEAGLIRLWYRRSYYVMVTAGRFPIGDLSTVHRAQPIRWTEWQNVVLLHGVGLLF

SVVVFVIELTVHYANVCLNNL

>DmelIR94g

MSTAVNSVHSKLVSLISRGQELTSIFFYAPAKEKCHLEDTISSATWGLPLVIWRTDRTVI

LNGFIGEGLLVLACLPGFHWRALLGSLARSLKYLRQARILIELMQDRDEFLVSEVLQFCL

SQDMINVNAIFDDFPETENLSSFEAYPSFEVVNQTFTPDTQVSDLYPNKMLNLRGGVIRT

MPDYSEPNTILYQDKEGNKEILGYLWDLLEAYAHKHNAQLQVVNKYADDRPLNFIELLDA

AQSGIIDVGASIQPMSMGSLSRMHEMSYPVNQASWCTMLPVERQLHVSELLTRVIPYPTL

ALLLLLWIFYEVLRGRWRRHSRLQSIGWLVLATLVSSNYVGKLLNLFTDPPSLPPVNSLA

ALMESPVRIISIRSEYSAIEFTQRTKYSAAFHLALHASILIGLRNAFNTSYGYTITSEKW

KIYEEQQKRSSKPVFRYSKDLCFYEMIPFGLVIPENSPHRAPLHSYTLLLRQAGLHDFWV

NRGFSYMVKAGKINFTAVGERYEAKTLTITDLRNVFIIYVSVLLISLILFTCELFVSWVN

YWLGF

>DmelIR94h

MLSNISFSSAPELVDLYGLVLKFLVSSETTLFYFNPTGQKCSWETLPRTILSNHPQIIWF

REETYPGLYKRHSSNLFVMACLSSTSYDGQLQLLAESLTRYRSVRVLIEVQDKEGSFLAS

QILLLCQQHSMLNVVLYFSRWTRTLNVFSYLAFPYFKLLKQRLSGSLRPKIFINQLKDLQ

GYKIRVQPDLSPPNSFSYRDRHGECQVGGFLWRIVENFSKSLKGDTQVLYPTWAKAKVSA

AEYMIQFTRNGSSDIGVTTTMITFKHEERYRDYSYPMYDISWCTMLPVEKPLSVEILFSH

VLSPGSALLLILAFILFFLIVPQLIKCLGITFRGRLIGMASRIFALVMLCSSSAQLLSLL

MSPPLHTRIKSFDDLLTSGLKIFGIRSELYFLDGGFRAKYASAFHLTENPNELYDNRNYF

NTSWAYTITSVKWNVIEAQQRHFAHPVFRYSTDLCFSSETPWGLLIAPESFYREPLQHFT

LKINQAGLITQWMTQSFHEMVRAGRMTIKDYSRTNLMKPLRIQDLRKCWVIFAVGLGTST

VVFTIELLLIYTNVFLNSL

>DmelIR100a

MATTLQLIMLALVGGTLGQANNTDHKQVLTSIVKQLEGGLELHLRTSEDGGNDLVQFLMQ

EKSSIIISAKQEEVPSRAKIMRHHFFIFDGVHQMQEIRTSLFNTDGFYILALENNTIEDD

VLLMEFAADVWLQHGHSRIYYVQLSKKSVLLFNPFLQRLVVVQDSKTYSRIYKDLEGYHL

RIYIFDSVYSSVIGDGENKVLSVTGADAKLAKTVARQLNFTADFVWPDDEFFGGRLANGE

YSGGVGRAHRGEVDIIFAGFFIKDYLTTHIQFSAAVYMDELCLYVKKAQRIPQSILPLFA

VHMDVWLCFLLVGLLGALVWLILRAVNLILGIEGVPDGSRATRISYFGAARRIFVDTWVI

WVRVNVGRFPPFHSERIFVASLCLVSVIFGALLESSLATVYIRPLYYRDVNTLRELDESG

QPIYIKHPAFKDDLFYGHNSEVYRRLDAKMMLVAEGEERLIEMVSKRGGFAGVTRSASLQ

LSDIRYVMTKKVHKIPECPKNYHIAYVLPRPSPYLEEVNRIVLRLVAGGIVGLWTGEAKE

RAKWSIQRFPEYLAELDVGRWKVLTLSDVQLAFYALTIGCLLSAIVCMAEILLGRQRRLH

SPK

>BmorIR87a

MTTGNSDQIAKTAECVLKLSAKYFVERKALSGSIVIINVNSYSSTTQGLLLKTIHSSIKYSVMAKDSFYPHANASHFPEKAKNYMLILEERTELKRNIFQLNKLPSWNPLAKAVVFYQIKGNESAQRIAIEFINELREHKFFRSIIFINNGTESGVTSYTWRPYSENNCGGKCDSVYVLDRCKNNIVEQIEPQPEWFPSNMNGCPLTTYAIVSEPYVMPPIRKIPNAKFDDVYEFQKGGETNLVKTIAEFSNMTLIVRLSAIEENWGIIYANGTATGAYGVLRNDSVDIVFGNIEVTKQIRKWFHPTISYTQDEITWCLPKAGQASAWDNLVIIFQWTIWVATFTSLILMGLLFHYMYYREKNKKITKWPTNSLLMTFSMLLGWGSHFEPKTATFRILIFGWLCFSINMGISYESFLRSFLMHPRFEKQIATESDLIQSGIRFGGREIYRTYFESNDASSSYLHTEYSSTTFSEGIRRAALNRDFAVVSSRRQAEYQDQKLGKGASLIYCFPESDNLYKYSVVLLARKWFPMLERFNGIIRSVSENGLINKWNDEMFIHRVSLEGASTIVPLSIQHLLGAFMFIGFMYGTSAFIFLVEVFVGFVQRRAFLSAFFCGKKKRFSAVFKVKV

>BmorIR7d.3

MRTEPEDITLFLQHFHGSAVIVPLDYQNMKAVSELNKATGFKQTVLFAVSVEEFILFITTLNLDLIVPIRMVLVLTTQLTDLAMITKEAWKHDLAEIIIISKDENEEIRLTTYFPYKNGICGDYTPHSISNEKELFPEKFKNLHGCPIKVTLLNFLPYVGLQKVNGTITFIFGIDGSVFILLIKELNAIMDIVSSTDHGGMGVFVNGSWKGSFGDIVRREADIFAPAGIITQKRFSVAQMSHTYETLNIHWCAPPRREIYAWAKVLLPFLTNITPFLVLAFTVFVITIVLVKRSKLHGIKSNKNVFLQSFMIFLGQGVKFETKSSVINSFFVAWLWFCLIVRIAYQGDLVNGLQKKIYEPPFESVEQALQELDGYGGTELFREYYAGSPIADNYQVIKIGDLPRYIRDVIAGKRFLIATDILMHQYAKKFQILQEPLTHSPTCLFMRPGWPVSRRVDVIIIRAIEAGLVQKIIYDFHYTVRLRRHEKEEETGTRPLGMSTMFACYYGLILLWIFSFVIFLFEVLYYNWKHKIAYIKRKRNKLFKFHH

>BmorIR7d.1

FNLPLKLTILSLFLGIILLNMLRKTIFFNNIRRVCNITPPKRNSLFYAWLLFLGLPLEKFSSRKHFKIIILAWIWFSFVIRCAYQVTLVTSLKSITYNYNLRYDSDILKYPFGGMSSIRDYFIEDKDFYENWTSVDMQKAYKLLDEIMEEKTDFVLALNKDTILHHAAEHIGSKRIQVIDNCIVNSPIVLYFRKHSPMTDPIAKIMNAALECGFIQYSYQTNWKRQKHLLNSHYAYNLQPLTLDNFSGCFFLLIIGYGISILYFVLEVVCHKIDKTNQRIDLRVDQE

>BmorIR7d.2

MSPRNLSDASHFCEENSNEITTAALNIALHNFKWRILTYVFFNATFLCNLNIFLKTYNKGVVVGNGLVEPRIDGKIQQLVLFCDDIVGITLALNSLPNQFDETGKVIVICQSPISWKCSAEEAMRSFWSVKITNVVFLKKDVFVMAYTYMPVYNEQCEISDPIPLFGLKPCIINATKCGVFDKKLDNLNKCKIVVSTLIRRPFMIINNGIPEGADGDLLLLIMERLNATLEVIIPGDHNYWGKLDSNGTWSGSLGDVYYGAADISMTSAALTASIISYFKISIPYRSTNVVWISHPPKALSPALKLLHPFKPSTQIALGIIFFIVIACVLFVSSKKMWLLCCRRVRPTKKKPSLLFNTWMICIGVPIAHLPSTSTFLSLIVLWIWYCFLIRTFYQVWLINSLQGKFYLDGFEKIDEAIEAGYDIGGGIFLKEYFVDYPYIYNNWKETVSLNVTLHEISEGSNFIAATIYDLAKSLTNFEKINVHFLAEKVVVSPSVLFFNKNSPLVAPINELLQQLTESGFVEKISRNYFTHNVTNWKRQKHLLNSHYAY

>BmorIR143

MFSVKYPRFYLFIIQIASNFGDAVTMPQLNSSINSKSATDCLIKVCYADLSFRRTVILKHVSYESDEENAFYNEIIHAVNNNNIQLVVLEEIDNLNDTINIDDADWLVVVYFKNCKALTEFNVKIVFEKIKYFIIVSDDLNEDCTSKMKTIGNVINKYDVTFVFNENKEDNFKFMTFIPQIDEETCKEIVTLPKIVNICANGQIERKSIFPSKNPKDIKKCPINVGMGSLYPFGIINHKEKYKTFDPLNETEVRGLDVDLVKVLVNQFNGTLNLYFIYKKEENPFGQLDFIPLVLNGSLDVIAGGFYRIYGNVVAYSGIYTSQAVTWMYVANRTTKSWQSLIVKIDGLYIFVIFHLIYSYVWYFVRKFDEQAVDFRNTILYSWGALVGTTSLQDALSLKQRILNLTYLIMCVHLSAYVSLHLYYFLTVLEPPELLKSNDDVMRSGRPAFLIPISKYFVLDEKYLSFANASEECTKFQDCSDLSLLRNGVTIILQGFFLNYQARTAINYEAKVLSAAENVLTVYYEMLLRKNSPYVERLQKLMTHLFEAGIPDRFYRHAIGLTVIGKAHSACQNTVSNSYSCQSGCKITFDQFAGVFYLWLFGCVLSCGAFIFELFSKFGRA

>BmorIR68a

TIMPLTLFSRSPRSGKTRAVSKASPILEDIYEQKDLEFVLVDLLNHAGRYHDFTCVAVICDAIYYNVFDGAFFKRIDTVPFVMIVVEEYDDLLSPNFDILEALREARRDGCNMYIILLANGLQAARLLKFGDRHRVLDTRAKYIILHDYRLFHSDLHYLWKRIVNVIFLKHHRKIGSVAKSQAWFDLSTVPFPNPIKGVFVPRRVDLWKSGKFHYNTVPFDDKTSNLNDEVLHVVYLDHVPSVVVVNSNETGQIGGVEIEIINTLSEKMNFRPKLYQPMNVELHKWGQKQPNGSFSGLLGEMVNGRADLALGNLQYTPYHLELIDLSIPYTSQCWTFLTPEALTDNSWKTLLLPFKLYMWIAVLLVLXITGTIFYGLARYQTYLHGLKRQEEMKKPVYSKPVGLYLFGEIINSILYTYGMLLVVSLPKLPTGWSIRFLTGWYWLYCILLVVSYRASMTAILANPAPRVTIDTLVELAASKLTCGGWGIETKNFFQDSLDEIGQKISDRFEISNDPNIAADKVAQGTFAYYDNKNFLKYITVRRQNGFIMETIDNTTNFTSISTKSNNERNLHIMSDCVVNIPISIGFHKNSPLKPLTDIYITRIVEVGLVEKWLNDAMYTIKTLETNEEEIKALMNLKKLYGAFIALAIGYFLSVMCLIGELAHWNCVVKKDPNYDKYALHKYYEKINKK

>BmorIR41a

IEILLQIIINKYLSESYCLVVISETPLSVKLPMSFTYLDPKKEHFSVETLLKLSEEGCSDYIIRMEDPRQFMNALEEIRPMSMVRRSDKKLVILPVTDDENSMEPILNLLTMKESSYYAHILLILPTQTERFECLAFNLITHRFVGSDSESKLPIILDRWYSCTNHFENNVYLFPNDLKNLNGKTMKISTFIYKPYVLLDVDTAVAPLGRDGIEIRMIDEFCRWINCTVQIIREDVDLWGEIYENETGIGVIGSVVEGRSDFGIAALYSWYEEWKAMDFSVSVVRSAVICLVPAPRVLESWELPFLPFGKSIWIAVVITFVYASIGLTIAQGCSSNKALLIVFGTIISQSQYIVSDSWRIRSVIGWLLVSSLILVSAYGAGLASTFTVPQYEPSIDTVQDLLNSRMEWGANHEAWTFSLALSSEPVAKKLIKQFKIYSFEELQRRSFLRKMAFSLEELPAGTFAIGEYLSKEAVQDMQLMLEFFYFDHCVAMLHKNSPYTEKLSELIGRLHQSGLLLAWESQVSLKYLDYKIQLEIRLSRARSDVGDLKPLNFNHVEGIFLIFITGTILSTLFFALEIFIGKQARKK

>BmorIR21a

MDRRSLYGLLFIFYIISSQEIISYHSESLLKNASRNLLWNKKITSIIKEHNDFAYHYESDLHFGNRIKNVKSKRAVDPVFHGHPKTREELWYERFLNRSSVFDQTPSLIKLIQNITLTYLNECTPVILYDSQIKLKESYLFQNLLRNFPVSFVHGYINEHSQLQEPKLLQPVRECLHFIIFLSDVKVSAKVLGKQSESKVVVVARSSQWAVHEFLSSSFSRGFINLVVIGQSFKEDDDSTIESPYILYTHKLYTDGLGASKPVVLNSWSHGKFSRNVNLFPPKMTGGYAGHRVVVAAANQPPFVFRRIFYKKIYRIKSDLDGGNPRVVWDGIEIRLLHLLAEKNNFSIEIVEPQELHLGSGDAVAKEIAKGRADIGVAGMYLTIDRTREMDVTFAHSQDCAVFITLMSTALPRYQAILGPFHWHVWVALTLTYLFGMFPLAFSDKHTLRHLINNSGEIENMFWYVFGTFTNCFTFLGRNSWSKTDKITTRLLIEIGKIFPCYYFRMVLDFTIIITSCYTGSIIAFVTLPMFPETVDTIHQLLAGFYRVGTLDRGGWERWFLNSSDPNTNKLLKKLELVPNVEAGIMNTTKAFFWPYAFLGSKAELEYIVQSNFTKTTSKRAVLHISNECFVPFGVTIGFPNNSLYTAKLNNDLRRMVQSGIVDKIVDEVRWEMQRSSNGKLLSAVGGSLKVSAAEEKGLTLEDTQGMFLLLAAGFLIAATALISEWIGGFSKLCRFRKKKNTLVNSSTKEDSINMPPTDSKDFKTETESVLHFCSRSTSPGSNESLDGQIINVTEESIEIHKQFTSEWDSRRSSSVDLEKEVKEIFERDLRRRGAALXXXXSTASNNAFGDAVK

>BmorIR64a

MNILGLNIISFLCSLDISSVIEVFKCKHVRDVIVFHCFKENQLILPQRMFHFNNFRTVFVFISNNISWELPNSYPKIGVLINTSCDGWEKFQEFQNSHTWVYYTDNLTSTITALSTFPIEINSDVTVVYKENSAYQVFDTYNTGRKNNGVFNVNYIGHINPGLQTNLKFTTRNLNGVTLKSTVVILKKVQYESFEEYLRKTEQTGLDSVHKHKFFQLLQYISEMYNITYDLIRTNTWGYAHDGRIDGMVGSLQRHEADVGGSPIFFKTDRAYVVDYVAETWPSKQSFIFRHPKHPTGVHTVYSRPLSNSVWYCVIAFLFVTASTVFFMLKFNIDEIERAETSQSLAFLFAWSAICQQGMSLRRNSLALKVVVFVTFVCSITLYQYYNATVVSTLLKESPITIRTLKDLLQSDLKVGVEDVAYVKDYFAHTKDPIAITMYEKKIVTGNNRNFFDPEYGMSLVKKGGYAFHVDTVYSYGIMKKTFTEREICEIHDVTMYPPQKMGAVLKKNSPYRNYFAIGIRRLWETGLMQRMKHIWDEPKPPCVRTQDSSIFSVSILEFSTPLFIVVFGVIASVVVLLCETLFDTLFNMR

>BmorIR75d

VNIQNNFANAPELPAYDFRREGVVLDLNCPNSKLILEKASKNRAFIHRYTWLLIHNSTYKLETIQKILSDAAVLPDADVTWCAADDILDIHRLNEHQPYVVMDLGLSVNSTIEDLDAVWSTIPTAATRRRHLNNLTINAVVIVSQPQYFKGWSDLSNRQIDTFPKLTYPMLMLCAEDLRFRFNLKQVDEYGVELNGSFTGTVGLLQRGRAELGVASMFMRSDRWRVLHFSSATVALLNAFMLRAPAQSSVSNIFLLPLSRGVWCCAAALLCGSAVLLAVLSCRLVAADPTLQLLTLPEIFVFSIGTVCQQGFYIMPKLSSIRMIMFLTLLTSLFTFTAYSAKIVAILQTPSAAVRTVADLADSHMDVGIQETTYKKVYYAESTDPSILRLFHRKVAPLGERVYMSVVEGVERMRTGLFAFQVERSSGYEIISKTFTESEKCGLMEIEAFKLPMVAVPLRKHSGYRELFGTRLRWQREVGLMSRVRAIWLAARPRCEGRGVGFRAVRLLDMLPALQMLAAGGLVAVVLLILENVYHHYSRTGNVLRRTYRNVFKLVCYKCYTF

>BmorIR75p

MVDVRPHRELFKRRRDVMGRPLTMANVIQDSNNTRYHLPREDALELQYDVIPKICWMTAKLAFQMLNATPRYTFSYRWGYKVNGQWSGMINDLHTSKADLGTNCVVSDVERLSVVTYTDMLAPFRVRFVFRQPPLPSVANIFYLPFTGRVWAAVAVCAMVYTAAIYWASKWEFNLEKRSASQFDGTVGDAMLLTMSALSQQGCFIEPKRAPGRIMLFVLFTALMALYAAYSANIVVLLQAPSNSITSLAQLAASKVTLAANDVDYNHFVFSLYKDPVRVMIHKRIDPETGNGQFYSLEDGVDMIRQGFFAFHSIVEPVYRRIEETFLETEKCDLTEVDFLSSFDPFVPVKKDSPYLELLRVVKKKKIRCSFKQIRESGIQSALNRRYQVPKPRCSNKVAAFSSVGIVDLRPVLIMMIYGIISSCLILIMEMLVFKM

>BmorIR75q.1

IFKIKNPVAITFGKIGNVEEVTPSNHILFLVDTTCNNSHIVLQEADAHQQFRRSYRWLVLETQGSGYNSKLLEIEPLNILIDSDVLLATKIENVTYVLKKIYKISTQSEWITEDYGNWTAEHGLIMSNIISSDASRRRNIRGHPVTTSIIVTENRTKSELDDLKNLLSDSLAKICFRHTKNLCQFMNASHKIGFASMWGYKTNGTWNGMMGDLAKGTVDFGGTIAFLTSQRLQVVDYLSSPVPINAKFVFREPPLSYQNNLFLLPYKANVWYCTAAFVVLLVIILYINAKWEIKKAEYEQAVTLQPSVSDVTILVISAISQQGSSNELKGTLGRAVLFLLFLTFLFLYISYSANIVALLQSNSKQIRTLQDLLNSNLNIXDGKPLFQTATEPIRKAIYETKVAPKGSKANFMSIEEGVKKLQKSPFAFNMNIGTGYKIIERYFEEHEKCGLQEINYIESSIPWMSCRKNSPFREIYKLGLFKLQEHGITDRENRLLFARKPVCIVRGGNVGSVNMVDVYPVILMFLYGLFLAFLILLVEIVVHRKL

>BmorIR75q.2

MSGGFQKNTNINEVIAVRRRDLEGYEIKICYVLTDNDSIHHLSDEVNDHIDTITKVNFPSTNHLLDFLNAERKYVFVNTWGYRINGTWNGLTGFLVNGDVEIGGSPMFFTAERTAVVDFISSPTPTRSKFVFQQPKLSYENNLFLLSFRTAVWYSTLALISLIFTMLLSVTAWEWKKMSQIKTRDIDAGVLRPSVTDVTMLVFGATCQQGSTVELKGSLGRVVMLILFLTLMFLYTSYSANIVALLQSSSSQIKTLEDLLHSRLKFGVHDTVFNRYYFSTADEPVRKAIYEKKIAPPGVAPQFMSMEEGVKKMRKGLFAFHMETGVGYKFVGKYFKESEKCGLKEIQYLQVIDPWLAVRKNTPYKEMFKIGMKRIQEHGLQNRENRLLYEKRPKCSGRESNFVSVSMVDCYPALLVLSYGIIIAIALVIMENLWQYRHLIKGKLEFFSSVNTIENFNQFEKHPSHNWKKFYVIDSAKIKKINPSTN

>BmorIR40a

MTKLPKDFNVAIKDIAESLPSKEMTVVRGNSTNIRSQDVFELLRLLCQHNIQVVNLDIAAMENKEMYYGYLKKALDVSDERTNLILCEPYECENLLLELRENNLIHRTILYIFFWPYGSVSDRFLNTMVEAMRVAVITNPRESVFRIYYNQATPNRLNHLSLVNWWAFRLYKSPLLPSADKVYKNFRGRVFDVPVLHAPPWHFVKYNNDSSINVTGGRDDKLLKLIANKLNFRYRYYDPPDRSQGSGIIGNGTFKGTLGLIWKRQADFFLGDVTMTWERLQAVEFSFLTLADSGAFLTHAPAKLSETLAIIRPFRWEVWPLVCATLFITGPALWIVIAAPSLWQRKKRDQMGLLNNCCWFTVTLFLRQSSTKEPSSTHKARLVTVLISLGATYVIGDMYSANLTSLLARPAKEPPIGTLPALEEAMREHGYELVVESHSSSLSILENGTGVYGRLAKLMKRQRVQRVHNVEAGVRLVLNRRRVAVLGGRETLYYDTERFGSHNFHLSEKLYTRYSAIAFQIGSPYLETINNVVMTLFEAGILGKMTTDEYKNLPEQSRRSEPVTESENLSTEKTGETAAVTQIQNETSKGLEPVSLTMLRGAFCLLGIGHLLAGVTLLIEIQLYRRARKRALPPQTRNPTNTFKAKAKKCILRGWRRIKAAAILAIDRALAPDRGID

>BmorIR76b

MLFSNAQRSGQNFPLSWIERDENGTVQAYGVAFKIIDILQQKFNFTYEIVIPHRNFEIGGSKPEDSLIGLTNTSKVDMIAAFIPRLVRFRKLVTFSRDLDEGVWMMMLRRPKESAAGSGLLAPFNNFVWYVTLASVLCYGPCICFLTHVRSKLIKNEERPLRLSPSFWFVYSAFIKQSTNLAPEANTTRVLFATWWLFIILLSAFYTANLTAFLTLSKFTLDIETPEDLYKKNYRWVSVEGGSVQYTVKTQDEDLYYLNKMVTSGRAEFRTLSPDQEYLPIVKAGAVLVKEMISLEHLMYGDYLTKTREGVEEAKRCTYVVAPKPFMKKPRAFVYPVGSKLKSLFDPTLAYILQSGIIDYLEHKDLPSTTICPLDLQSKDRQLTNSHLMMTYYIMCVGLASGLAVFVVEILVKRYINIKIKPIDKVKLKKFKRSKRSPRYDDSGPPPYESLFVKPKFKDSEKRWKMINGREYYVYEDARGGTRLVPVRTPSAFLYR

>BmorIR93a

MKIWVLGVLCLAISVQGEDFPSLITANASIAVILDRQYLGDKYQTVLDELKDYIKELARVELKHGGVLVHYYSWTNISLNKGFLAVFSIASCEDTWELFSRTEEEDLLLFALTEVDCPRLPQRSAITVTYSEPGEELPQLLLDLRSSNAISWKSAVILHDDTLGRDMVSRVVQSLTSQIDEESARPVSVTVFKMKHEMNEYLRRKEMHRVLSKLPVKYIGENFIAIVTSDVMTTMAEIARELLMSHTMAQWLYVISDTNAHASNLSGFINTLNEGENVAFIYNITENGPDCKNGLMCYSQEMMSAFISALDAAIQAEFDVAAQVSDEEWEAIRPSKVQRRDILLKHMQQYILAKSVCGNCTLWRALAADTWGVTYRQNDVPEQINEHANGSTGVIEHLELMNVGIWRPIDAMTFADLLFPHVHHGFRGKELPIITYHNPPWTFLQANESGAIVKYSGLMFDIVNQLAKNKNFQRLPHPSNRNALLLHGRNRQGGGTYPCGLTKGPITYNNIPLYFRAVFIAHQAGVNLKNNYYRCINYTIPVSTQPHTFIVARPRELSRALLFLLPFTTDTWLCLGFAVILMGPMLYIVHRLSPYYEAMEITREGGLATIHNCLWYIYGALLQQGGMYLPRADSGRLVIGTWWLVVLVIVTTYSGNLVAFLTFPKLEAPVTTISELLKNSDAYTWSVTKGSYLEMELKNSEEPKYKRLIKEAELLKETGGIEGTIHAARGTLDRVRGQRHLIFDWRLRLTYLMSADHIATETCDFALAVEDFMEEQVAMIVPAGSPYLPVINKEINRMHKAGLISKWLSAYLPKPNRCLKISTVTQEVSNHTVNLSDMQGSFFVLFLGNDKIYVYMYIAELI

>BmorIR8a

AVPWTLPKLDPETGDPLYNEDGQPIYEGYCVDLIQKLSEAMNFDYEIVSPRSGGFGRRLPNGSWDGVVGDLTTGETDIAVAALTMTAEREEVIDFVAPYFEQTGILIAIRKPIRKTSLFKFMTVLRTEVWLSIVAALVLTGFMIWLLEKYSPYSAKNNPGAYPYPCRDFTLKESFWFALTSFTPQGGGEAPKALSGRTLVAAYWLFVVLMLATFTANLAAFLTVERMQTPVSSLEQLARQSRINYTVVEGSSTHQYFINMKFAEDTLYRVWKEITLNATSDQAQYRVWDYPIREQYGHILLAINASGPVADAETGFKQVNDHTDADFAFIHDSAEIKYEVTRNCNLTEVGELFAEQPYAIAVQQGSRLQEDISRALLELQKERFLEQLTSKYWNETLRQSCSDADESEGITLESLGGVFIATLFGLGLAMITLAWEVFYYKRKEKNKVQSTKENVERPPIKSAKLGGKMAVGVARLRKRATKIGKKKNVTIGDSFKPSVSYISVYPKGDYR

>BmorIR25a

MCRSSHIFQNSMPLFVVFLQFFIFRLIVSQTTQNINVLLINEENNALAEKSFEIAKEYVRRNPSLGLAIEPVIVVGNRSDAKTFLENVCRKYNDMLSSKKTPHVVLDFTMTGVGSETIKSFTAALALPTISGSFGQTGDLRQWRSLNANQTKFLLQVMPPADILPESIRAIVTKQDITNAAIIFDELFVMDHKYKSLLQNIPTRHVITPVKSFNKEDIKTQLRSLRELDIVNFFIVGSLRTIKNVLDAADENQYFGRKTAWFAFSLDKGDITCGCKDATIVYMRPTPDAKSRDRLGKIKTTYSMNGEPEITSAFYFDLSLRTFLAVKSLLDSGKWPNNMKYITCDDYDGKNTPNRTLDLKLAFQEVKETPTYAPFYIPGDDPMNGRSYMEFSTDLSAVTVKDGASIGSKALGTWKAGLNSPLSLTDSDNMSDYSAQLVYRVVTVEQQPFIIRDDNAPKGFKGYCIDLIEEIRQIVKFDYEVTLSPDGNFGTMDENGNWNGIIKELIEKRADIALTSLSVMAERENVVDFTVPYYDLVGITIMMKLPRTPTSLFKFLTVLENDVWLSILAAYFFTSFLMWVFDKWSPYSYQNNREKYKDDEEKREFTLKECLWFCMTSLTPQGGGEAPKNLSGRLLAATWWLFGFIIIASYTANLAAFLTVSRLDTPIESLDDLSKQYKIQYAPLNGSAAMTYFERMAAIEVRFYEIWKEMSLNDSLSDVERAKLAVWDYPVSDKYSKMWQAMKEAGLPNSIEEAVQRVRDSKSSSEGFAWLGDATDVRYYVLTSCDLQMVGDEFSRKPYAIAVQQGSPLKDQFNNAILQLLNRRRLEKLKENWWNNNPKAMKCEKQDDQSDGISIQNIGGVFIVIFMGIGLACITLGVEYWWYKWRRRPIVGDVTQVEPAKSTRNNIGNFVKGEGFTFRSRNFGLSDLKQKF

>SgreIR8a

MWPLWVSVIAVQLQLASSQATSDPLLIRFLLVTEVNATWVGGELRANLSGLEARYAGLRLQLDLSAIEVD

REHEVEEFQQKVCGELAVGVSALLDATWTGWRRLRDEAQHRALPYLRLDATLANLVDAVDKYLQAREASD

AALIFHTEEELDQALYHLIGNSVLRVIALNGLETETVNRLKDMRPVASYFVIFADTAHMSELYSKATAGG

LVRKAERWTLVFTDWEWRSFGREHLNLSTALLQMKPASCCALQGEPDACRCTLRKVAPAYLRAALSAVVE

ALTELHSKGLEVRPVPKQCSAADAGSPSDDEGGGGGGGGVDAEDATEPPPNATGNYDAFLRAIAPRGQTN

STLFFRMQAAQLTFNTPLQLRMVNRSEEIDLGNWSAEKGLQLNTQLKPAKRFFRVGTAEGVPWSFPVRDE

QTGAPLVGPEGEPIWDGYCIDLLKKLAEPTHMNFDYELVPAKDNDFGSRSPSGSWTGLVGDLAMGETDMI

IAPLTMTSEREEVIDFVAPYFDQSGISIVIRKPVRETSLFKFMTVLRLEVWLSIVGALTVTGVMIWLLDK

YSPYSAQNNKEMYPYPCREFTLKESFWFALTSFTPQGGGEAPKALSGRTLVAAYWLFVVLMLATFTANLA

AFLTVERMKSPVQSLEQLARQSRINYTVVMNSDTHEYFRNMKNAEDVLYNVWKDITLNSSSDQSKYRVWD

YPIKEQYGHILQAIDQAGPVPNASVGFQKVIDQEEGKFAFIHDAAQIRYEVSKNCNLTEVGEMFAEQPYA

IAVQQGSHLQEEISRQILDLQKDRYFESLTAKFWNNSAKGTCPNSDDSEGITLESLGGVFIATLFGLALA

MITLAGEIFYYKRKKLTTVNVTSASAKIPKKQVTIGKEFRPVMEKTAPRVSYISVFPRNQLY

>SgreIR25a

IQKVCLGYNSSIDQNKKIHIILDTTLQDVSSEAVKYFTRALELPTVSASCGQEGDLRYWRNIDKNQEKYL

IQVMPPIDTIPEFVRSFCSEQNLTNAGILFDDTFIMDHKYKSLLQNVPTRHIINEIKFKNIGEQLSTFKQ

REVFNYFILGRMDTVNKVLEAAADKEFYGRQFGWYAVTQDEGNPSCQKCGKGASVLHVKPNDPEGTVIGS

ENPKLTYQFYYELFRNTFLAIAQMMGEGSWPDLEYIPCEEYEENKNIPPVRKLNLLDTLQQISMQIPGAY

GQIILSSNGHSHMQFNMTALNVSLSDNTATEVGTWAADLDSPFITKVKPSVPVTQYTVVVALQQPFVIKY

QDENGSTKFKGYCIDLINAIRNITNFEIEIYEVADGKFGNMDEEGRWNGMIKDLIDKKAHIALGALSVMA

ERENVVDFTVPYYDLVGITILMKKPKTPTSLFKFLTVLENDVWLCILAAYFFTSFLMWVFDRWSPYSYQN

NREKYKDDEEKREFDLKECLWFCMTSLTPQGGGEAPKNLSGRLVAATWWLFGFIIIASYTANLAAFLTVS

RLDTPVESLDDLSKQYKIQYAPIANSSAHVYFQRMAAIENRFYEIWKDMSLNDSLSEVERAKLAVWDYPV

SDKYTKILQAMTEAGFPANIEEALERVRASKSSSEGFAFIGDATDIRYQVLTNCDLQMVGEEFSRKPYAI

AVQQGSPLKDQFNNAILQLLNKRKLEKLKEQWWNQNPEKRNDCEKQDDQSDGISIQNIGGVFIVIFVGIG

LACITLAFEYWWYKLRPQHNAIVEAAPHRTKSDSLQALNMMRSSFDKRYGRRQGVALAGVTNPW

>ZnevNMDAR1

MATSLHLLVLSILMQPLYLTADSRQHVTADVRIKNNPIYFNIGGVLSNMESQMFFKETIS

HLNFNSKYVPKGVTYDSTAILMDPNPIRTALNVCNYLINQSVYAVVVSHPLTGDLSPAAV

SYTSGFYHIPVIGISSRDSAFSDKNIHVSFLRTVPPYSHQADVWVELLKHLGYRKVIFIH

SSDTDGRALLGRFQSTSQSLEEDVEMKVQVESVIEFEPGLDNFIDQLLEVKNAQSRVYVM

YAGETDSKVIFHDASLLNMTGKDYAWIVTEQALEASNVPIGILGLKLVNATSEENHIKDS

IYVLASALRQMNQTEVITKAPKDCGNSGQIWETGKALFEFIRKQTLENGSTGNVAFDDNG

DRINAEYDIINVQNQTQISVGQHHYSNEHKRMKLKLDKSAIIWPGNTKNQPPGFMIPTHL

KVLTIEEKPFVYVRELQAEGDGCTPDEIPCPHFNNTNDGFKQYCCKGFCMDLLKELAKKI

NITYELLLSPDGQFGSYIIRNSTVPGKKEWTGLIGELVNQQAEMIVAPLTINPERAEFIE

FSKPFKYQGITILEKKPSRSSTLVSFLQPFSNTLWILVMVSVHVVALVLYLLDRFSPFGR

FKLANTDATEEDALNLSSAIWFAWGVLLNSGIGEGTPRSFSARVLGMVWAGFAMIIVASY

TANLAAFLVLERPKTKLTGINDARLRNTMENLTCATVKGSAVDMYFRRQVELSNMYRTME

ANNYDTAEEAIQDVKQGKLMAFIWDSSRLEFEAAQDCELVTAGELFGRSGYGIGLQKGSP

WADAVTLAILDFHESGIMESLDNRWILQGNFQQCEQFEKTPNTLGLKNMAGVFILVAAGI

VGGIGLIIIEMAYKKHQIRKQKRMELARHAADKWRGAIEKRKTLRATMATQRRLKANGVN

DPATVSLSVDALPRHCLETRSPARAWPGGCDIRQRIPHTDEIRVATTAQSNLAYGTGLPD

MIV

>ZnevKAINATE1

MELNLSQADSFSTGKRVCGAVREGVAAVIGPRAPASTGIVQSICETMEVPNLQIYPEVQQ

HPGCCVISLYPAQESVAQAIADVVRNFLHWSAFTVLYESDEGLLRLQEVLKAHGLEDRKI

IVRQFVPGKDQRKLLKEINASNETRILLDCATDRVLDVLQQARDVKLISVYHSYFITSLD

THTLDLSEFEPGQTNFTLLQIVNPEDMNVELTVNYWTDKYYYYHRRSLGVTAETVRTETA

LMHDAVFLFARALHRCYFDVNQSFSVESLECDDRHKWIYGLPLANYMKISEMDGMTGKIR

FDDSGRRTRFKLYVVQYFTGEFRKVGWWETGHGVNRTQSETEKEAEIQKSLQSKKLIVAS

RIGEPFLTEVEPRGELQGNERYAGYSVDLMGKIAEIVGFQLEFRLVESNKHADLVNNLIA

RTADLAICDLTITHEREKLIDFTMPFMNLGISILYGKPVKEDASLFAFLDPFSVDVWIYV

ATAYLGVSILLFVLARTSPGEWDNPHPCRSDPEELENTFNLMNCLWFSIGSLMGQGSDIL

PKAVSTRMVAGMWWFFTLIMIASYTANLAAFLTLNRLEGEISSAEDLAKQHKVKYGTMAG

GSTAGFFKNSNDSTYKRMWTTMSQSVPSVFVNSNEDGVQRVQKANGKYAFFMESTSIEYQ

VERKCDLQQVGGTLDSKGYGIGLPVNSPYRTKVNGALLKLQENGVLQELKEKWWKAPANE

SCSAKDASELDEDSNKLGLANVGGVFVVLIAGTLTAFLFAVLELLWNCRKIAVQEKISPC

EALTSEMKFALNCSNENKPVRRRKEEEEEEEEEEDAMPAGVTAGSFARIGFEKFRDKE

>ZnevKAINATE2

MAACSEMSSHVLLVVXXXXXXXXXXXXXXXVLCCGNMAAGLPDVIRIGGLFHPADDRQEV

AFRYAVERINDDRSILPRSRLSAQIERISPQDSFHASKRVCHLLRSGVAAIFGPQSGQTA

SHVQSICDTMEIPHLETRWDYRLRRESCLVNLYPHPTALSKAYVDLVRAWNWRSFTIIYE

NNEGLVRLQELLKAHGPSEFPIAVRQLGEGTDYRPLLKQIKNSAESHIVLDCSTERIYDV

LKQAQQIGMMSDYHSYLITSLDLHSVDLEEFKYGGTNITALRLVDPEKPELQKVVRNWVY

GELRYGRKLDLPPPRTETALMYDAVHLFAKALHDLDSSQRIDIKPLSCDAVDTWPHGYSL

INYMKIVEMRGLTGVIKFDNQGFRSNFVLDIVELNKDGLKKIGTWNSTEGVNFTRTYGEA

YTQIVESLQNKTFVVTTILSSPYCMRKDSSEKLSGNAQFEGYGIDLVHEISKILGFNYTF

KLVPDGRYGSLNRETNEWDGMMKELLDQKADIAIADLTITYDREQAVDFTMPFMNLGISI

LYRKPIKQPPNLFSFLSPLSLDVWMYMATAYLGVSVLLFILARFTPYEWQNPHPCNPNPD

HLENQFTLLNCMWFAIGSLMQQGCDFLPKAVSTRMVAGMWWFFTLIMISSYTANLAAFLT

VERMDSPIESAEDLAKQTKIKYGALRGGSTAAFFRDSNFSTYQRMWSFMESARPSVFTSS

NVEGVDRVVKGKGSYAFLMESTSIEYVIERNCELTQVGGLLDSKGYGIAMPPNSPYRTAI

SGAVLKLQEEGKLHILKTRWWKEKRGGGSCRDDTSKSSSAANELGLANVGGVFVVLMGGM

GVACVIAVCEFVWKSRKVAVEERLDSFPEQNRVVVQQHQCNGSVLKTSRQ

>ZnevKAINATE3

MSVSIMAPLLIFLLLAAVAGLPGTIKIGGLFHSTETLEELVFQYATQTINNDSMILHKRL

MAIPEHVKSHDSLSVSDTVCRLLGQGVAGIFGPQAGSTSALVQSMCDTMDIPHVETSWDM

KQRRQDFLVNLHPHPSTLARLYMELVSAWGWEKFTVLYEDGSSLVRLGNLVKTLDRQVTL

RQLDAHGNHRQVLRDMRHSGERNIVLDCSMGTLPEVLKQAQQVGLMTSDQSYVITSLDLH

TINLEPYQYGGTNITGVRMVDPNSKIVQSAVKNWGYLEARKGRNIGISSENITVSMALMH

DAVQLFARALSRLDSPAVDIKPLDCESQNNWGHGTSLINFMKDGQSTLHGLTGVIQFDNE

GFRTNIMLDIMELSYSGLERVGTWSTKDGLNIIRAVPPAPPHHSESLQNRTFRVITALSD

PYGMRRQSSVPLKDNDRFEGFGIELIHELSLMLGFNYIFDLQLDNAYGSLNKKTGQWTGM

IKKLRDEEADLAITDLTITSEREEAVDFTTPFMTLGIAILYKSPQKESPSLFSFMAPFTT

GVWFCMFSVYMGVSILLWIMGRICPYEWNNPYPCIEEPEELENQFTLQNSLWFTIGSLMQ

QGSEIAPIAVSTRMVASIWWFFTLIMVSSYTANLAAFLTVETSTTPFKNVWELANQNVIK

YGAKKGGSTVNFFRDSSDSRYQHMYAYMMRNQDEVLTSTNDEGLQKVKTQNYAYFMESTS

IQYITERDCEVAQVGDLLDSKGYGIAIKKNSIYRNQLSTAVLKLQESGKLAKMKNRWWKE

ERGGGKCVXXXXXXXXXXXXXXXXXXXXXXXXXXXXXXXXXXXXXXXXXXXXXXXXXXXX

XXXXXXXXXXXXXXXXXXXXXXXXXXXXXXXXXXXXXXXXXXXXXXXXTQVAAGTPVL

>ZnevIR25

MRPRSLLVSLFLFITLDASQPVTAELMNILFLNEEGNKIGDEAFNVALDYVKKNPSLGVE

IGEVIKAVGNTTDAQTFLKSICSVYDAAIKAETRPHVVLDMTMSGVPSETAKSVTAALAL

PTISTSFGQEGDLRQWRSLEEAEKNYLIQIMPPADIMPEIIRRIVIFQNITNAGILFDDS

IVMNHKYKSLLQNLPTRHMIVEADEGNGEAQLKRLRERDIFNYFILGRLSTIVSVLDSAE

KCGFFDRQFAWHGITLDSGNLGCSCKNATVFFVKPKPNEEYTETYTELTEKYNLQNLPEI

SAAFYFDVALRTLLATKEIMQGNDYRKNYVTCDDYDETKHVTRDVDLLTAFKQVSQPESY

GKLSITSNGESMMEFQMEMTAVKIRSSVPQTAIDMATWNASLTLPLDVKDSTTMVKHSAV

TVYRIVTVVQNPFVIYDGVDGKNRTKFKGYCIDLIDEIRNITKFDYEIYEAPDKKFGNMD

ENGNWNGMIKELMLKNADIALGSLSVMAERENVVDFTVPYYDLVGITILMKKPKAATSLF

KFLTVLENEVWLCILGAYFFTSFLMWVFDRWSPYSYQNNREKYKDDEEKREFNLKECLWF

CMTSLTPQGGGEAPKNLSGRLVAATWWLFGFIIIASYTANLAAFLTVSRLDTPVESLDDL

AKQYKIQYAPLNNSASMIYFQRMSDIENRFYEIWKDMSLNDSLSDVERAKLAVWDYPVSD

KYTKIWQAMNEAKFPNTLEEAVNRVLDSKSSSEGFAYIGDATDVRYLVLTSCNLQMVGEE

FSRKPYAIATQQGSPLKDQFNNAILQLLNKRKLEKLKEQWWNQNPEKRRDCEKQDDQTDG

ISIQNIGGVFIVIFVGIGLACITLAFEYWWYKYKKIPKVVDTGKVVAHSRQIPTTGGGKL

ETGLKMQGFRPRNPTFPTHSFRRNVGPMTGVKSPW

>ZnevIR93

MMLLSWTTIVLFITFHQVSNAEADTDYSGTVAHLAVVIDKEFRGLDYKNLLRQMRHFLRN

ATHQHLTHGELITKFFTKTDIAVEKDITALFSILSCDDTWKIYRRYQDYHLLHLAITEAD

CPRLPRDDGLTVPLVAVNRVASQLMLDIKMSQLASWTTSILIYDESVDTETVQRIITSLS

LPTLGRERSAAPVAVFKVNDTQREWERRASIMKLLKDFPVNRLGSNFIVAVSHEVVGVIM

EVCKAVGLSHPETQWLYVIADSDAIINMSAFTSLLSEGENIAFVHNSRSSGVECEGGLLC

HVHELLQSFVEALGVVIEDEEDFISQVSTEEWNAIQPSKRKRRSTLLDLMKAQLIETGRC

DSCLTWTLEAGDTWGLEYQEHEEEETGQKIVRRLNPVGRWSPRDGLSMSSHLFPHLRKGF

VGRDLTIISFHNPPWQIIKHNDTSQITEYKGLIFKIIDQLAENLNFRYTVIFPANNIPGW

TNDSSLMKDSEDNRTRAFLVTDRIIEILRRKKVFLAAGAFVVTPNRKTLVNFTMPVSIQT

ATLLTARPREVSRALIFMHPFTYGTWACIATLIVMVTPVLNYFHRHSPYYEYYSKDNVKG

GLSSHYNCLWYLYGALMQQGGMHLPEADSGRIIVGAWWLVVLVIVTSYGGNLVAFLTFPK

YEVAVTNLEELLTRRGTVSWGILKDTATEQHLKEMDYPKYKSLFEGATIHEEQDDDLVSR

VRSGSHVFIEWKLNLLKIMKKEFLSKNSCDFALGDEEFLEEQVAMMMQFGSPYLGLVNRE

LRRMHQAGLIYKWYLEYLPRKDRCWTTNRLLQATTHTVNLDDMQGSFFVLGLGCAFAMVL

ICMEQCYHTYKISKEKRVIKPFAS

>ZnevIR76b

GRRSVDDNRASGQLRLVCVRCVNEAGVHANADGXXXXXXXXXXXXXXXXXXXXXXXXXXX

XXXXXXXXXXXXXXXXXXXXXXXSTRLLFATWWIFITILTSFYTANLTAFLTLSRFTLPI

DGPKDLAKNKAGWIAHRGSPLEYVVQNDKDYEYLNKSVRDGKGRFVDLADIDIFTLVKNK

KLPLLREKRSVEYWMFRDYMTKTENSVPENERCTFVVTPNSFMIHGIAFAYPKDSVLGKT

FDPLFQSLVESGIVKHLLKQGLPPTEICPLNLRSTERQLRNGDLFTTYMVVVVGFISSIV

AFIGEVLYTTMKRCSMSSKIRTSDADWTGDYGYKKSEMLPPPYSVLMQQQHGLGKHQIIN

GRDYLVINSKSGDSQIIPVRSPSAFLFQYSA

>ZnevIR68

MLSDLNCLVIMNDNIQQDIFEGHFFKKLGSVPYYKVLVKEKEDLQSPNYKTLSVIRHVKR

AGCQVYILMISNGGKVSRFLKFGDRHRVLDTRAKFILLHDHRLFHSSLHYLWRKIVNVVF

LHQQGRHHGSVITRQKIHPWYDISTVPFPSPIDSTFVPLHLDTWHQGKFRSGADLFRKKT

SDLRGQQLRVVTFQHLPASVKMASPSLRIDSVVEGNGPVGFGGLEIEVLRTLATVMNFHP

DVYEAENADVEQWGRRQLNGSYSGLLGEVMSGQADIALGNLYYTPYYLELIDLTIPYTTE

CLTFLTPESLTNNSWMTLILPFRPLMWAAVFVALILAGFVFYALANYHIHIVSTAMNLQT

NNAIMVQERSKINDSKVIHIQEERNKTGDGLYLFSKLENGILYTYGMLLLISLPKFPSDW

SLRVLTGWWWIYCILLVVAYRASMTAILANPTPRVTIDTMEQLVDNHITCGGWGEEIKQF

FLTSLDISGQKIGLKFEVIYDTDLAVEKVAKGEFAYYENIYFLQYLRVRRQLIVKEVGTK

KDVNTNEESGGNRNLHIMHDCVIHMPVSIGLQKNSPLKPHMDRFLRRIVEAGLIKKWLKD

VMLSIVSVDNTDKEDGNKPLMNLQKLYGAFVALGVGYLISICAFAGEKIHWQCVVKKSPL

FDKYAINIYYDHQKSSKAIKK

>ZnevIR21

MSKLLPSQQNKFYHFHHGKRDKSEELSHKRIQETSAPLPTQDTIAQQSVLVRLLTDKITL

TSGHRLAKNCMNASDSVQVPSVVKLLNYIAEQYLFNCISVILYDDFYENQFHLLRALLST

YPLAFFHGKVGKQGLTDPPDSRCRNFLLLVRDLKTTQAVVGGRSTSRVIVVTQASRWRVR

EFLSSRLSHNLVNLLVIGKQSQSNSIKQQNIDINLYTHDLFVNGLGSSSVWILTAWRCGA

LTRPTVNLFPDKLRAGFMGHQFVVAAGHQPPFVVKREQFFDGQRLVTSWDGTEIRLLWLM

AKVLNFSFTVEEPKDALFLPEASSAVVKELLAGRADIGVAGIYTTPSRVAVLITSSPHTQ

DCVTFMTLASTALPRYRAIMGPFHWTVWLALTLTYILAIFPIAFSYNHSLKHLLKDPWQV

ENMFWYVFGTFTNCFTFKGEWSWSKSDKAATRMLIGSYWVFSIIISACYTGCIIAFITLA

VYPSYVENCYDVLDRDFRVGTLGHGGWQEWFNDSADPATTKLFEKMEYLPDLESALHNIT

KAYYRDYAFLGSWSLLDYIIRINYTKXXXXXXXXXXXXXLKASAHTCTWGQIVLYPSM

>ZnevIR411

MSVETTFIFWIICFCMVPTGYAMYFTEGCYFGTISNLLGPMVYNIAERYFINSQCVCVIT

EENADILKYIPKSILIFHIQIGGIGSTIQEIEDSDILDSTKLTNQTQLFERLLTETMNAG

CEPYIVQVRNMRSVIHSFARTSRRAITRYSKKFLYLPVTLEDEIPSLENVFSMKEMNYMP

DLIVARFVNAKKRSDLIQHRYENMISKNKHYESNGILNSSTDITIKFIGDRHEKKQSEVP

SNNTTMAAKCFIQWGMEGKLANRFINLCHASNLNRIPQNVSVIEIVTLKFVGQTPSREIV

LDAWITDCQNTGFLTRTNLFPNKIRNLEGRKVNVITFNYVPFTVITHDKEKSTVYDGFEM

RTFIECAEKLNFTWKLVLDEENLWGAVWSNGSGNGVVGMVAEDQVDVGFAGLYYWYSTFL

WTDYTTALSSSGITCLVPKPKLLPSWMSLWLPFSAVMWIAVGVSILIITVVFYTLAKASN

KCLGGAILNSYATWINCFLTTMGMFVLQSPPEPQDASHRPIKGFVISLLIFFLLVSSVYS

GGLASILTVPRYEHPIDTVKELADSNLPWVNIHEAWVWSLLDSDDPISKTLVHNFQVLTE

EEMTELSTQGETAFPLEKFASNHYSVPAHINETAIQTLRIMKEMMYGSHITIATRKSSPY

KESFNQIISSLYETGIMLFWEEQMTERYMSRRLQIAIKQSVILSNKEGPTQLMVDHLQGA

FVILSLGLALATVVFTAEMAYSTI

>ZnevIR412

MSVKLIVLWMCFSHATGSPPSFHGDNSDGIADSIGYMTKHIVQKHFSHSYCVGLIVENGE

ILKHIPTFTSTIVVDISTISKTVNSKEFETTQEEAGVFDKTLVKLLDQGCLSTIIQVSNP

KLLVEYLYTSSRKSESRSNKRYLYLPPFHNSGTSNYDVPGLFRMKEMAVMPDLVVAKVIR

KHEIASDTDDVNQGHNSIRQRIPYTNTSDRRLSGVQRREKEGIERLRYSRLNWYEIEVVT

HRFVGSNPGTEVWLDTWIPGKGFAEDTVLYPNKMRNLHGKVVHLAAVPNYPPYTIINMKT

TPPVYDGIELRFIKDFARHLNFTFSVITDEVNWWGKVWPNGTGNGMWGNVAMDVAVMGFG

ATYAWLENYPHLDYSLPYFRSSVRCLTPCPQKLPGWMTPLLPFTSNMWAAVGISMLITSS

SLYLVTRTIMSLPGPNSKPHQCFSTVTDAIISSVGLLLLQVPDEERSPRYIPQRHLLAWL

MVYFLLINTIYSGGLASVLTVPRYEHPINSIADLARSDMLWAATTEAWVFSIQEATEPDF

RRITRNFRVMTDEQLLARAFAGDLAFAVERLLGGNFALPPFLNEKTVTNVRAMREDLYSG

YCVFNVRKASPFLESLNTLILKLHAAGLLLNYEGQVVQAFSSQRMQLILAQNDQPTSDSG

PVTLQIQHVQGSLCFLGFGLLIASLVFITEVLKK

>ZnevIR413

MAHSWVRMFWELVRIISISLQWLAVTSEPGIHQDVLISSVGEMTHNIILNNFGGAACVGV

ITEINSDVIDYIPKSLLRFHIQIGRNDSILKLEESDTLDYASLSDGTLAFERLLIESLNA

GCPAYVIQVSNPKPVIHCFARASRRAMFRANRQYLYLPILQGSTIYDIPDMNADDIFTMK

EMNYMPDLVVARIVLQNKNKRDGINTNHRFKSFKNIGIKKYNLEGRNNSTQRECTESNSS

IIVGTVTSKFKIELRTHRFVGSETSKNVLIDVWLPNSGECTGKFLRSLDLFPDKTRDVKG

KELTLVTWYYPPFVILDLYANPPLYDGIEFRVVREFLRYINSTFRVTSDPENYWGDAFDN

GTGNGVLGFVAMDQADIGFSAFYAWPSSFKVVDFPASHMRSSVTMVTPQPKLLPGWMVPL

APFSSTMWGAVGACLVGSTYILYNLQRFVDKYLGNSQDKVNPFSKMDYTALRTYAMLVNQ

APTFSWNSNVPRHVPARHFVAWFQFLGEVISDTYNAGLASVLSSPRYEPPIETIEDLANR

DVIWAGNHVAWIWSIEEDDNPNLQTITENFRCLTNEQMNEIGQRSGDLAFGIERLQGGHF

TTEPHINGGTVKHRRIMRGTLYWSHLYILLRKGSPYMRHFRTLVSRVRDAGLPLYWEGDV

IRRYMSERLQLQISTSRIPVGNLGPTKLNLDQLQGVFFLLVLGEVLGFIAFVAEFTYGKN

>ZnevIR414

MLTVMVLAMSALGSCNELPNYATELIGKMCHEVARKYFLKSKCLALVTEDNSKILDYMYP

LDFPTFHIQLPLSFMKSSERLDEEFETYPWDKFFMAPIDAGCLAFVVQTSDVKFMVQTFA

RLAHSPRSIHRANKKYLFVPSRDVEDAIFETGVKNVFSMREIDFMPDLVIARFVAERNLV

SGYSKRTLPVADSSKAEGNLTLELITHRFVGQNPSERVSLDMWMPTMDFLYKANLYPDKI

SNLMGKHITMTTMHYPPLTVIDENADPPIYDGLELRFIYEWARAVNFTWSIVYDGGWWGD

VWPNGSGDGLCGLVSSDIADVGFSAIYLWDNAHLFLDFSSIYFGSSLTVVTPRPKLLPGW

MVPIHPFNPDMWYAVGMSVIGCTLALYITSKTSMRLLGRGTRNNVVNLYSTWVECAFRTM

GLLVLQVPPDERDFSTPRHVPMRHLVTWLILFYFVVTTAYCGGLATVLTLPRYEKPIESV

SDLADHNLIWGGMDDAFTFIMRGSSDPKMQTVVRNFRLESEEYLTARAKVGDMGFVIERM

LGGHFYMPSFVNEETMQRLRVMKGNLYFSHTVYVLRKGSPLETSINNMIQWVRDAGLFVF

WEGLTVRNYMSSPRQHSVINSRNEEDMGPTKLLLQHVSGAFYMLGFGLAVSLVILIFELV

RFKFRQIVEK

>ZnevIR415

MLALTVCMYAVAATRGLPYDVTKELAGMSREIASRHFSNSKCLALLTDDNNDIMDYIHPQ

NIPIFHVHLPFAEMKDSKVPSSGFKQYAWDSILLAPMDAGCLAFIVQTKDVRFMMKTMAR

LAHSPQSTYRANRRYLYLPSKRIPDDAFETIIKEMFATREMDFMPDLVIAKLTELNVTSE

SNCSTLLRAGNLGTVRNMKFELITHRFNGQNPSERISLDVWDPKHGFQLRADLYPDKVTN

LMGKTLSLTAIFYPPLSVVFEDSDPPIYDGLEFRVMYSWAQQNNFTWRVVYKPDEWWGAI

WENGSGVGLSGHVSMDKADVGFGAIYLWENEHRFTDYSSVYFRTSLTTVLPKPKLLPGWM

VPIHPFSTYLWSAVGLSVITSTLVLYMTSQASSRILGSAQGKVVINKYSTWVECAFRTMG

LLVLQVPPDERDFSTPRHVPMRHLVTWLILFYFVVTTGYSAGLASVLTLPRYEEPIETPV

DMADRNIKWGGTDVAYVFFLQQSLDPKMRKLANNFIEGNEEYLTREAGTGKMGFVIERMQ

NGHFFLPPFINEDTMQNLRVMKEDYVYGNCVFMLRKGSPYMGIINSIVYKVRDTGLVLYW

EDLTVRRYMSTRRQLSVINSRKEADPGPTQLLLRHVTGSFYLLGYGLAASLFVFILELIR

YK

>ZnevIR75

MFMCLKTHRYIKVLRIAEIGTMNYWWVYFGFSLAFHNTLTGVYADFVSRNTLSLIRSFLY

ENKISFAVVINCRTAEDIRIMKFFSDKSIRIKVQTKCDAVEASSLLSVDNHKLGVVLDWQ

CSDSTRFLTQASEQKLYSKLHHWLILRFYENSGRTVKLNGDTIAQLRNNTHVTQSDDTLI

STKERSRSTPHEDALIAALEPLDLPLDSQVTVTRPGADVNQVLLQDIYRIKSGLPLIFTP

LCDWAPGQPFPFSLRRDNYGGIVLNTATVVIGDTWENFLNWRYKHINSLTKFHYILVEHL

SRMLNFRTNVTPVNSWGFPVNNTANYDGVIGLLQRGEIEVSSVGLLHKTARMDIVDYAGE

TVCYEGAFLFLKPSLSDVSNIYTLPFSRAVWVTYLVAMAVFSYALYVTQRAESGLDESQG

TQPLSLSDSLLTVVGIICQEGTTRDPQRISSRIILLSLLLLSLFLTTSYSAIIVSLLQTT

STAINSLKDLMNSPFTLAMNDVATSINYVNETTDADIKNLYFKHLFTQPYHKAFTTSEVG

VEKMREGLYAFHGDADAFKIISETYEEHEKCRLKNIKMFSTIQLALVVQKGSPFKEHIRQ

RARWLKESGISDREYKYWIVQKPKCHGNVEGFKSVRLQDFYPALLVFAYGLVLSVGTLVS

EVLYQKLQCLKYSQH

>ZnevIR75d

MWLTLGTLLISLVVWMHCYAVCDNRLATVRAFAYHNHISSVISFLCHQRAATSFYKTLSD

EGIPVSVFLPGQSVDAPSLLKTEYYRLAVIVDIACDGSVQFMAEASRRKQFSSLHFWLLL

SQSGNMFTSSNNGDGPTLSNESLITALEPLDLPLDSHVTLALPGPDQVLLQDVYRLVNGQ

SLTVTPPRHWRQIEGFPVGPQRDNYRGIVFPTTAVVTEDDKWENFLDWRYKHINSQSKFH

YTLAEYVSRMLNFRMNVTPVESWGYPIRNTARYGGAMGLLQRGEVQIAAVGLLFKPTRMN

IIDYAGETVRFEGAFLFLKPSLSDVSNIYTLPFSRSVWVTYLVTMAVFSYALYVSQRTEG

GIDGPRGTRPMFLSDFFFTAIGIICQEGTTRDPQRISSRIILLSLLLLSLFLTTSYSAII

VSLLQTTSTAINSLKDLMNSPFTVTMNDISYNINYVNETTDADIKNLYFKHLFTQPYHKA

FTTSEVGVEKMREGLYAFHGDADAFKIISETYEEYEKCRLKNIKMFSSVHLGFPMKKGTP

YKEHVRQRVRWLKESGITDREYKYWIVQKPKCHGNTEGFTSVRLQDIYPALLVFLYGLVL

AVFILVLEVSCQRLHCSQCRSYQTSPQYSYTN

>ZnevIR75f

MLPVSLIPGSVTMLFVCCVSETRVPQITLSLMESFIVQNDVSSAVIVNCWNLPDKASVTK

NLSQKNIMVKFQDPGRDTDTSLLLRTDYHRIAVIVDLKCERSFLFLSYAGKNNCLKLQHH

WLMLVTSDLSLNGLKSKQEPLITALESLPLAVDSQVFLAHQGSDLSHVLLQEVYRIRPDL

PLVFTPLLEWSSGQLLPPKLRRDNYGGISINAATVVIDDVWENFFDMRYKHINSLGKCNY

MLMLLVSQMLNFTVNLTLTNTWGFPKKGTSCYDGVVGLLQCGDTEIGALAILYKESRMDR

IDYAGETFVFGLSFKFLKPSLSEVSIIYTLPFSRSVWVTYTITGAILSVALYATQKVESW

VNPSRTGPPLLFGDTALNSLAIMCAEGSPRSPENLSSRIVFLFLLLLSLFLTTSYSAIIV

SLLQTPSNAINTLTELINSPFKLSMKDIAYNTKYVNDTTNPEIRRMFYQKLYTRPHYQAF

TTEEIGVENLRKGLYAFYAGSEAYKIMSDTYEEYEKCRLKEISINPSNVLAFPVKKGSPY

REHITQKARWLKEAGMVDREYKRWFTQKPKCDANSQGFVSVRIKDFYPTLVVLLIGIICS

TILFILEFVYHKIKYGTYWPRDSTTSQGELVNARHEKHGN

>ZnevIR75h

MKLLSLIVCLLLVFNLNRAHSLAINDGSITLVTSFMTQNHVASAVVYTCLKKADSLQLIT

VCSTRGLRTQIHMPDSESDMPLLLSTSYYPVAVIVDLECEGSGQLLQMACSQHMFSRLHY

WLLVSGEELSSASVLEQLYLPLDSHVTVARVASRYNQTALWDIYHVGVGQSLTVTTTRYW

TPGLELPAAPPRTDYGGITIPTVTVLVQGTWENFLDFQYRHINTESKVQYILMGHIATML

NFRMKECLTNTWGYPINGSECFNGAVGLLQCGGCEIGATGLLFKKERLSVIDYAGETITF

RGAFMFLKPSLSEVSVIYELPFDTSVWITYSVSVAILTLVLMFTRRVYQHVNPSASSDSQ

IGWGDSIMDSLAVVCQQDALHNPRNLSSRIVFFFMLILTVFLITSYSATIVSLLQTSSNA

INTMQDLMKSPLKLSMIDIFVNETTDPEVAKVFKEKLYTQPFKEAFTTEDVGMEKIRRGL

YAYHGLSGAYKIISDTYEDHEKCRFKEVVMFTSLHLCLTARKGSPYIPHVKERTCWLREI

GLIDREDKRWFYHKPKCETAGQNFVSVGIQEFYPVLVVLAYGIVGSVGALIIEILFFKRQ

QINCALDFTNNVTVMHKDGN

>ZnevIR75i

MHLHRMFLLYFYTSCILNGEARSVYKDTMHLVKAFYEFKSVSLVTASVCWGGYEHSLVRS

LSSSGVRVNLVLEPIDLVTVLEQDIYYRVGVLVDLACNNSMDALERASSKKLFNMQKFWL

LLESGRLKEVPAVLQGSISNYAVVYSVDSNIQGQFNFNVTYTLSELGNFVLSKLRILADS

ELTWVQRNRATGLIQILYPDQFIDWYDLRLRHVDKWSKIHWPIYGYLAEQLNFSFEVTYQ

LDNYGWLINGTFDGMMGYMQREEVEFPSTGIFVRNDRFAVTSYAASTFPLRATTMFRQPS

LSTVRNIFILPFDRSVWLCCFALCLTSVFTLGVQITCSVRQHIEENMLHSDWSDMFTLVL

GAICQQGYHLTPTSLAGRTTVFVLTLSSLFLFTSYSANIVALLQTPSTSIQTIHDLAISP

MIIEVENQTYNSVYMKETTDRELRDFYHRKIAPLGEKAYLHATIGMKHIKQSMHAFQVDT

TSAYKIMSETYQEHEKCGLKEIDLFPAPMFTIATTKGSSLREFFSQRVHWYRNVGLLDRL

FKIWMPQKATCGSNAGGFVSVSLTEFYPALMVLQYGTAFSVVILVLEVLSFHRSRIALKL

RCILYPKYLRQTKQSSTADPNGNKAKMTFRSQGNWLK

>ZnevIR75k

MRWTKTHVLLFYAIYWLLCLSAVSQPMDSATMTFIGDISKASPPAVVGAFVCCKSGDILR

LWRHLSDQHVLLTTQTTIQGAETLLQTTERDRQMTFIVDMACQDSRAILHKAHVLNLFDE

RRRWILLGNQTRTQDVLEDLNILLYSDVVVAERVSDGTVLLVEMYKRRSYETLIKTVIGD

WSQGRGVRLTSSPVKFSRRADLQKSVLQAVMVVTNNNSLLHLKDTTDRHIDTISKLNYAI

FCHVADIVNARVEMTVVGTWGYDVNGSWTGLSGYLQRGDADIGATALFVTKNRLPFMTYI

AGTTDSESGFLFRQPPLSFVANIFTLPFSRNVWLTSVGFITVAGFLLHGTINWENRQVTW

SDMVLFAVGAVCQQGSTMEAKGLSGRIITIFLFTTEAKGLPGRIITIFLFVAVIFLYTSY

SASIVVLLQSSTTSIRTLHDLLDSDLSLGAHDIVYNRYYFKAVSDPIRHAIYTRKVNPPA

GPENFLPLEDGIKRIRTSRFAFHMELGPGYKYIWDTFLEEDKCNLQRITFLTEIPKPFVA

VSKKTPFKEILTIGYRKVHERGLQQRENLRFYHAKPECASRGSNFVSVGIVDCYSALLVL

VYGMLLSLVLLLAEIVANKRCRKLAQ

>ZnevIR75l

WKSTNEELARVWSGARRVPGSSMPRALSLPRRIYCQFGGRISTNRSSRPSWXVTNNDTLN

HLSDTINKHIDTITKVNDVLFHHIVDVLNASVETTVVDTWGYPDSAGNWSGLTGYLQRSE

ADIGATGMFVTKERIPFVRYIASTSVTRNAFIFRQPPLSFVTNIFTLPFSRSVWLASAAL

IAVAGASLYFALKCEKRRDEVTGSDITLLSLGAVCQQGTTVEAESMPGRIVLFVLFTTVI

FLYTAYSACIVALLQSSTDAIRTLKDLLNSGLTLGVEDVVYNRHYFPAASDPIRRAIYNK

VGADRFMSLEEGVEKLRKGLFAFHMELGPGYELISDTFLDEEKCGLQTINFLIEIVEPWV

GVSKTTPLTEILSVAYRKVKEFGLQHRENSRFYQTKPKCVTRGSMFVSVGLVDCYAALLM

LACGLLFSGAVLLMEFLTMTLHSYKNSVVTFDDREEHLVRDTDVSLRPDQLGPAPPNRYH

GSSDRSVKLITPI

>ZnevIR75m

MMLKEPFVFVMLIYCAAESLDGETSVQFIADYFAYKGAHMITTFTCSSHDARYLMSLTKT

SPTAANFWTVSVTAENGNFSHEGTKRTRNVLNAAGNVHGVFLDYGCATNRDIFTQMSDKF

LLNASFNWLIWSTDGLSGMENVNLNIDSDVTWATPDTPRGLVLLYDVYKVNYTWPVISTL

AGQWNRDNGLVYTLTQFKYLRRGDLQGLLFNAAVAVSDSPGDDIERWLSSPVDRHHMDSI

AKYNYALHRQLGDRFNFTMQLHATYSQGYPDSNGQFDGMLGMLQRGEVQLGITSLTMLPQ

RFEIVDFTGPTWKFRTSVLFRHPRTTGTSYSLLKPFTKTVWLYTLLSWALMMVTIHLVKW

LEYLRQRDDHQPSRSDCDHSWGATILTIFGAISEQGTEMESKWMTWRIVYLVTLVQVVLL

NAYYGACMVSDLLQPPPRTIHTVRDLINSRLHVGYVDNRHNREYFQDITDPTLKELYSKK

MKPPHAFPVSTAIEKLHKEPFAVHDEAISLYPTIEQTFNNEDKCAITEIVLFSPRMTYPV

IHKRSPYRRLLSYGFHKLWETGTMARELLMWRPEPPKCQHLDNFYSVDMEGVAVAFLIFV

FGVITGSVILFLERLQDFKCFWKKKDE

>ZnevIR75n

MFVAVKVITVLFTLFFVQYSVVVSLDSSVIELATDFFAYKRVRMVRSFACKTHDSILLAK

SLQSSGNIWNVLSKRKGSAFPEEDPYAKWKPSADTADYYKFGIFMDFDCDSSKEIFNESC

EQLYLNSRHHWLLWSNDHYTNIKYAKLNVDSEVTWATPDSSQHGVNLYDLYKINFTWPIL

GTPAGHWDEIGGLVYNITNSKYLRRGNLHCLNFDTAIAVYNYSMAVDDVHEHLITHEESN

FTTIMTKASYAQLCMLGEKFNFTMTLLLTDTSNFAPFENGSFQGQIGMMQRNDAKLSPSP

LLFYRTREAAADFVAPTWSFDTAAMFRHPKVSAVRNALLQPFTATVWLCFLGTWLLVLVT

LKVSAWLESTYDSDESTTVESSWSATMLATVGAVSEQGSEMESKWVTFRIVLLFAMVQVV

LLNNYYGGGIVSSLLTEPAKTIRTIQNLIDCNLEVGYENVSHNRGLFEQTTDPLVQQLYR

KKVFPPTQSYPNTYRVDIGVKKMKNEPFVFQVEQIEAYPHIDSTFTDSEKCALTEIPLFT

SMVGPTFALVQKGSPYKKLLALGFQQVRERGLMKYKWQEWRPAKPRCVLTRDVVSVEIET

ISFAFFLLVFGLFASVSILVIERLHYDRCSIRRQTGRSIPTQGPRRILGT

>ZnevIR75o

CSRCTIFXXXXXXXXXXXKTILHTNTFGYVVEDGKPEGLVRMMKYGEADVSISALKLNHR

RIDMSDFCPVHTWELRVCAIFRHPQITDSYGVLLKPFEPTLWLGCGLIWLLMMIALRFIS

FFESQYYVVDNLHERNKTELEEAWTWSDTLVIIMGAVGQQGSTMDSKWITGRIVFLNTHI

LALMMNVYYSAFVVSSLLSAPRQSIRNTRDLIDSSLTFGAEDINYNRPFFEMNSDPLVQE

LYQTKMAPPHTGYFSREMGLQKVLNERFVFHTEAINIYKVIEETFTEQAKCDLTEIQVLP

IWQCYPFVPRTSPLKELLTYGYRRAAESGLISHLNRHWRPTKPACSVKTEFRSVGTDTIV

FAIWLLGAGTLISVIIFATEIILRSRFRRSCNTPE

>ZnevIR75q

MQGLSISYLVLILSSCTTGWLSEETIQFVEEYFTHKHVRVITGFTSSKHDTFLLMKSLMS

AGKFWMVFADPEQPLSKAIDPIIKRSYKKFGIFVDYDCPQGKEYLLESSAQRSLNDSFFW

LIWSKTKYTSDIKTLNLNFDTEMTWVYPETDLSHFTLYDLYKINYSLPINGTLAGYWSPE

RGLNLILTQSKYERRHDMRGTVFTAGIVVNNIPTDNIKEEIVKPENRIKDTMATYNYRVF

QLLEQSYNFSVIIKHTSLYGYVVENGKLEGLTLMLKNREVDFSITPSFMNKHHYECMDYM

NARTWKYRSLALFRHPPVTRAYGAELLTPFEMNVWISCAGMWLVVILTIRFVSWVETSMF

DFVATSHEEETVRSWSDSLMIIIGAISEQGTTMDSKWISWRTALLAACIMSMMVNTSYSA

SLVSSLLSTPMKTIRTTRDLIESSLQFAAEDISYNVELFALNPDPLVHELYEKKMAPPNR

PYYKREVGLDKMRKDYFAFHTEPLNVYPMIRDTYTEKEKCDLTEVEILHPEYCFMAIPWA

SPYKEAITIAMRKIIQGGIVNYQDQLWQTPKPPCMVKEEFVSVDIVKISPAFLIIGAGIV

LAIILLIGEIIIKKENLMKASQDLLLAKGERLQPGIQATIKHRRRTTNKPWIN

>ZnevIR101

MAVALTQRVFRGPLYCACVMGDDSSVARDVIGTVIATIPVPVSGGNCPGFAGGMFIAQDA

ASVITSVVTASRHFHRLLLLIPGSLQELLDKAPLFRNISFEVVAANCSSVFHLTSSHHLS

FQRLTTDSDTVQRQLPNLQGRVLRVSTFHCPPFSYRNSWKNINHGGSLPKGVPDGVEMAI

FLALSRRLNFTWQMNDLHDADVWGHRDDNGTWSGGIVESMSQKLADIAFCGIYVEETIMQ

EMDLTIPWTHYCLTFLVPRSARGFRFAFLKTFQPLLWLFIIIVTLGTAVVMWLLSRFERR

PRGIIETVLTAVGLLFLSSFPEQQTRLGTLRLLMAFWSAFSLLLTTALSSGLVSHLTQPA

PTTQLNSVRDLVESGLSWGQAYEQDYSAIFNMQDPWHAQFADQFKLESNPRDRVTRVRRG

HYAILGGKLDGTAPYFMEGDTLKDTGLLSQLRVMDECLIRQYAALGLIKXXXXXXXXXXX

XXXXXXXXXXXXXXXXXXXXXXXXXXXVVTDTACESCDALVT

>ZnevIR102

MQQLYELMAGSVLRATFPHNCSCLHMAVTGNAMETLVIASAAQICPLLVLMLGITPTVPT

EGTIMVTEKAHDVLAVSRRYSRLLIFYTGDTPLRDFDDTLAMLLVVQQDTNFIITEPISG

RILGQWDPVEGLILRNKLPWIDYALKTRNGRLSSLRVSLFECPPYVMYKHVNINGTEAIQ

FDGIEVRIVREAMCKMNITFLPEDGSVWGNTSKGPWKRIVEDVAEHHADVAVCSLWLTAN

PPVGLQMSAPWTRQKGTFLVPKSRPLTPAMMLYLPLKLWTWVVLLHVTLIFVLILYGISR

MLPSGEGCQYHSLCICLLDTFRMLMLNSCPRFPQQAAMRYLLTSWAFFSLLTMTTYSTGY

TSLFTFPPYSPPINTVDDLLYQGIYWGERSNDFKLLFIDVGNPKLAEFGKRFKPEISVLD

REKRILQGQYAVFTKTMSSTFVTETETLSHKARQKLRIMKEPVFTFYVAIGLCPNSPFKS

HIDSTITRLQDAGIVHYWQRLMIQNLGYHYMSKFFEVTHKENTGHSALSLDSMQGAFCML

GMGLALSCITWFIEISTGHRNKMCTTSTYTKT

>ZnevIR105

MKHHSTRGIMKGLVLCLLAKLPLIQISSSLSHDKQLMFLQTFCHHQLCSSIILYDITDWC

NISLKEFQFVKFISQLYLKILVTSTQAWTSCQPLPDSAGKTPLLVLPYRASTQHHEFLKQ

CSQEGQFNGRILWLFLTSDSLSSHLFQNLAQLNIRLDSNIFIAVYGKEANIQLLETYALG

TLGQNTSLQHHIISEWNPGNLVPPSTHKYDRHRHGNLQGYQLRATTVLINEFYTQIQKSG

DHFQVTGGYFGEVFKTLANALNFTYTIIYLPGYSYGNTPTDNTSMWTGMIGAIQKQEADL

SICEVSITADRLEVIDFTLPLHTAATQLYVHEGTKEPSMDWYFHPFNVWTWFALIVGLLL

FTSINNLLHYITYNLHGSTQRSGFLSNLFTTVTIMLQQGTDSPSSFSMRLVQHSSALLFL

VVHIAYSAKLTSLATLYKPQPPLGDLSDILKSSSWKFGVMNSSLPFNTFKNAEPGSIFGK

LWHLKLEPFPGHLMNSYTSGLSATLAGSHFAFLGLEDSCQNTLQHSFTPAQACKILALPH

KYFRGGLSFALQKNSPYKEVINHSILKMKSIGLIQRLKLKWVPQPQQCNKMLYVVMEWTD

ILLPVRGFIAIVLTSLFILIAELMLYKYRTVMGHQKKNHVQTQSVDKLYKHNIKPTRNLF

ALYK

>ZnevIR106

MHPKRSVIKQAYNLNVNQIKLIRWLSSGLLHCVVEVCQRFTGPCCLHRQNDEYLVSQKQL

FHAYVSWLIITEKKIPAVDSSYSIEERLRYFNIGIDTDLVIATKINVSDISDSDGRSWTH

GWGDKADATNDDRDGTYRVRKTDNPVNDEKLNSVRSYCINENEKYHERIRIQKEEKRRRN

NRKYNNKNNRNDKTSEIGNCVENSNRREYYGLEFSLLQVYKIRPNSNNSLIMISLGSWNA

INGTLNMVSSNGIEQRNDFRGFPLFVGVKNTTQNTGEVVGGSIKSISLPAQNSDDEIEYD

THLMEVLDLITRSLNASYELIPFSKLGWKNDQGAWLFMLGAVTVGTVDLGLDNVVITPDR

YRDMFFTLPIVQSMRNIYFRRPETGAMRDIFLAPFSPRLLASVVATGVVIATAMFLVNAS

TAKTNRRNESNSNSKSSKTKWILGEAMVWSVSVLCMQGSSWSPKSPSGCLVLIFSLCFAL

VIYNAYAAFITSVLSVRVGSIRGLGDLLESDFHFGYTRKGQDEAFLLGLVNDSALRQLYL

RGFVKNHGVDEASSGLLKASHGGYAFFVSARLGRKALNSFIAQDKRCAIQELTVEATKSA

IALPMGIASPYRRLINLSLLRMREAGVLGPIQEHMLPPMPRCQAYSAFNSASLTDVYSAF

LVLGGGLAAAICMGLSEKVWKHRVSIGHWLAIKWAGRDKVESISDQQRLQVHRQMHQRSR

FQHDIQWFN

>ZnevIR109

MVCVARILATLTLLVAGAEGAVPDWVVARVVSSLQRRYHSGCVFLLHTADKTDLTSQERL

VQFLLRKRLSEEATQVAARRLRPDNSSLQCSRNVPLNVVVSSEPDVKLTLTEFSNRHNLA

ESKWLLFLTEGNIGSFFADMNVPFNCEFLVARVTTSSVTLYEVYRVAEGRPLNNIYFGRW

TPLSGLVSVQESIYRRRGDLQGTVINAITANDPPIVTILQNQTEIVGFFGRVWKILEQNM

NFRTNYTITGDLTKGKPTQKGDCSGMIGVLQCGQVDVALGAFSTSSYPTLPVRFSMPLLY

TTYRVFIMKPAGRDAEWDAFLMPFSPRLWLVLLATILFIAFYLSTFYSIGRWVGNEESGG

PELYTFYDSILYIFGAFCQQGHDLTPRSTSCRLVYITAYLTALVLLAAYSAALISSLTVY

RSNLPFQDLEGILRDKTYKLGVVNMSEIYYTVSDPTDKDIFHEVYSKLMLPDQDNFPRTV

LQGLKRICNIRYAFMSIPESVMPLLNQMNCTIVPLAYNTYPISLAMAFSPRSPYTDFWSY

RLRMLRDGGVIYRLRTDSWPYLGQDMTKSTTVGVDLKAVAPLLALLSASTVMSVVILFLE

RGRFSVSDRRSRGRTRRERNASRQQMVIMHAAPEKPSIVPLRPGGRKD

>ZnevIR110

MPQLTCSAMGPLTLLATYIILTLSVARCQQWNKTATPLVIFHVGAHVRSTCVFLYYNQES

LMLDKIRLLVQVQKALTLRQMASAVISIDQLIAHHHLGCRSNRPLYVLFSSATLLQVSSK

VRLSDAVWILFSNFQEKDVESLQEFFHNVYIPLDCHFLVAHSQGPDNIIIISEVYHVTRD

LPLAKNYFGILSHGAWVTLATISFFERRNDLQGMTMKVATMTEPPVTIVKTVDGRLVLEG

FFGKVWQVLEKRMNFTSEFIMPADRAWGSLAHNGSWNGMMGMIVRGEAEIAVAEFTMTAL

RAEAVDFTVPLINTRNCVLIRQISGEKDPTWYGFLKPLSSQLWALVILTQLVIILCLNVV

RRLKLRCLGRKSSDSRDDGHFAAQIVGIFFLQGVNVKTGFCSERVLYLVAQLSALTIHTA

YAAFLISCLTVHKFSLPFDSLRELIDIGTYRLGVLANSGQLNIFNNATDDLMREVYAKLI

APDKDNLPISIEEGMQRVCDVNCYAFMTSLDVVLGLLDNITCELSSVLGASIPESLAMSA

IKQSPYLGLINHKAMFRSGVLKRLRAQEWPTELPPATVLWVPVDLPSTLPFLAVLGGALL

VSALLLTAERLLGRRRQSVSCKVFCPKNALSSTAANSTANSGGIFLLL

>ZnevIR111

MTQIYYLTDFSANTCWLPNNGAFMLTVLARLTFKKTKKSLLHAWFSDTPYSGTQQSQSGL

TTTSTLAQWQLQLSSELCPSSVQFRLNSTQTFSTCQRSRPLFVLHPGDPTRIKPFLQFAA

NRSFSRVIWLLFLDKNSSLEELFTDINIPFDCEFLVAQPEDDHAVGLTEVYRVSPTLPLQ

TYRFGNWTPDGGLTWPSRGFYHRRNSLQGLVLKTAVIDAFYTNVVEESNNKPIKISGFFG

EMWNTLEDQLKFKSDFYKPLDNFFGAPTENGSWNGMIGMLHRGEADFAVAKFVMSPARLQ

AVDFISTLEQVKTRLFIKEQRALLVKWDEFLRPFTRRLWMLQLFSMILIGLCFGVTYHVG

QRLGLEAGVASVLQNLYDALFYTFASFCQQGRSVTPRMLSCRVVFLTSYFTAVVLLAGYS

AFLISFLATRETKEFPFTSFKQFLDDGRFQLGMLSDSAFTFYFENSTDPVMQNIYQKMIA

PNRHNFPFSDDEGFRRVCNVPNYAYITYTDLEYQETGTLSCGLTEIPQIYIPGSLAITTV

KKSPYKGIFNSVIWKMKRNGILKRLHTMFYPIKVPISEIPRPVVYLVQVTPVLTLLGAAT

LVAIVLLLVENKAAYIKACLQIGRQRMNQLLFGLILVSPTHARKRRQT

>ZnevIR113

MTDESVATSRTHSVLQMFQMQTSICIMLLLSSGAISAGLEKVLPLTELIADIYHQFERGC

IFLMTSRVQQQGFEAKILMQKIRKHLLYEYIPIMDTVFRTGVSRRCSTQRSIYVVLKGDA

ETRNYIHQSFLKTHSSRAIWLLFLDKNSSLEEFFTDINIPFDCEFLVAQPEDDHAVGLTE

VYRVSPTLPLQTYRFGNWTPARSPTWPSTNFLLRRSNLQGCVIRATYKHEYLVVNIVNNS

DGSEVAEGYFGQMWTTMAEEINAITVYHETESYGSKSANGSWSGMIALVKNREADIGIGD

FTATSERSNVVDFIDTVEFSSTRVFIRIPNSADMSWNLYLAPFSYGLWLAVAIACCALSV

CLAVTNISYDGQKRNPTLSVVAIFFYILGCLCQQGQRDAVNNTAGRIVTLTSYVMSLVIF

AGYSASLISSLAVQPRALPFRDLQGLLSDGSYRLGVQENSAVLNIFDGAVEGVENEVYKK

LIAPFTNDIPRDDSEGFKKVCTLHKYAYAGSYFVSQQYKGATLCQMTTLPGTSYPEPLTY

IISKKSPYKYLINWKLAQLREKGIQSRIRQHYITSKASYVNRSATTVNLAAVAPILVVFV

AGNLIAIFLLVIERQIHGNLFKCWPVGNSRRPENNEcRKRYSNRLLSLSHKNVYRPPLYR

NIHRSIRKHKCK

>ZnevIR115

MSLLTNMISPSFVFCCISMFLFIDCQDKFIFPLTRIIDTVTSVNTYFRCGCVFLVKSLEQ

ESQGKFSTDRVVYEQKLLASRRVASAVLPLPFPAGVQCGRNRPLMVITQHHRAVGLQEQF

SGSHAMSNFFWLLFLDKSSSLEEFFTDINIPFDCEFLVAQPENDHAVGLTEVYRVSPTLP

LQTYRFGNWTPDGGLTWPSRGFYHRRNNLQGLTLKTGLKLEGNNSESLNRVETKTFSGVV

SRIWRQLENEMNFTTQYYEPEANTCNSSTSNASCAGINGMLQTGQVDVSNAATRMSAVRL

ATVDFTVPIGESRFYIVIKANDVFIKKWIVFATPFSLRLWIAVACIMVVSAICLTEIQVL

ERRNDEGNTEDFSFVTAMFLVVGVFCQQGHNISTKSTPVLLTFVTSYLTAVVLFACYSAG

FVSYLTLRHSQLPFTDFKGFLKDGSFELLAMKRSVEWDYFKSSSDDVLREIYSKFLLRLE

VSHPDTALEGLQRVCHHTKVAFIICTDDMMTYVDKINCTIVRIPEASFPASVAFALAKGS

HFRGLFNHRLQQLRRNGVLNMWNWYVSSKMRHRVDPPKPSISIRVAKPILYVLAVGAVFA

VGVLTVEICTKHISSVLRLAKGKLYAVQIG

>ZnevIR117

MECQRAVEQLCQLMVVSVSLSAGTVFCDPAADVPVSATADIISATHRYFTTSCVFLIHTE

AHINFNQRPKVVRVLKDSKVAVVTISVVTLFGLQEGYHRRCSRPLYVLPFSGLKLLDGIR

QLSRAVNLSHSIWLLLLDKNSSLEEFFTDINIPFDCEFLVAQPEDDHVVGLTEVYRVSPT

LPLQTYRFGNWTPDAGLSHPSQSFLHRRNNLQRLNLHVAVNNDSLTIVTGADRGDVLKVG

GLFGQMWRVAERYRNFTSHFSTPSLNLHGENLKDGKWNGVVGMLAARQADVSCSDLTMTT

SRVDVVDFIEPIWTDRTYIFLKKVDDVAVGWTTFFKPYSWQLWLAFTAAATLLIVCLAAI

YYVRSQFVGHTDFQMQRCGLSHAMFHITACFCCQGSEIVPRGVSCRTVHILACFMSVVMF

TSYSGALVSTIASRKAILPFKTFQDFLNDGTYRLGVVANSSTISNMKESSETLLQKVYES

LLPDIGNLPKRNNEGLRRACEDGRYAYMSPLYVLLTNAATCTLQTVPHAFISSTKAMAAV

KGSPYRLVLKQTFRTIRRSGIFRRICSETLTGQPQEKETTFYSVDHNAVSPLLSILFIGM

VASILILTVEINYSHSQRCL

>ZnevIR118

MMQPGAGLLVSIILPALLGCVLSEMPSLPEKAEIIHSVAHHFHLNSVFPLEIQGEGIDYG

QSVVKIRRYLSGSRIFTFSYLYPDTMVRLGVLNVMLSTEKSEESLRQVNFSVPLSNTVWL

AFMDSDSSVEELFINIAVPFDCEFLVAQEEDGCIIITEVYHINAALPLQTCRIGNWSSHI

GMSWSSMTFYQRRRSLQNTVFRAGVLQGPATSVFKGADGKIMKIGSYIGHIWNILQGIMN

FSTSYYTAADSAFGVEMVNGRWNGLLGMIDRKEIDVISTDILMDRKRLGIVNFMNPLFEV

RTYMFIRSPKKSDMDWENFLKPFSTGLWMAILGVILVLSVFYFIGIKVCRKYNEESEGIW

TFRLYDSVFYIYYSFCQQAQSDPVVYSTSCRVLSVTTYLTAVVLLVGYSGFLISYLTFRT

TELPFTSFGHFLQAGTHRLGVLSNSTHYVHFKESTNPNMQQLFLRYIFPNKADHPKSFLE

GLQRVCSKERYSFMAPVFIANGFLKELPCDLVAIPEAYIPATATLAIEKRSPFQGLFSHN

IQKMRRAGILQKLRKDFLFSKLINVENAKSPADIDEIEPILAILLLGIALSCIVLLFEIA

ASRASSYTNSVAKK

>ZnevIR121

MLKVLLLLSLATSSVPMNLSNVADIVMRVQRHFYSRCVVLLKTRERLLQPSYNIFGLEKL

LAENGIMTSLSNKAQYVNCCQHKRPLYVIMSSGLEERQILYDFTSNSCMSAIWLLFLNTA

SSLQGFFANISIPFDCEFLVAQEEDDHVVVLTEVYRVSPSLPLQTYRFGEWTQRGGMICV

SVGLYQRRTGLQGAVLKTAVKNEFLTDRQFEYSLRTDIFFNQVWLTLERLLDFRSELKLT

PENVYGKQMENGSWDGVMGMMARGEVDATSVELTMEPMRSEAVDYIAPLINDREYLFIKN

HNLFETSWTSFLQPFDCWLWVAIFATMLLLSASLFLISQFCCLHEYEDKQTAALNSFSDC

LFAMHTALCHQGFSEVPQCISGRLVCAVTHFTMVVILAAYSASLVSYLAFRQPVLPFSTF

SELLNDSTYQLGVHAASYLGKFDMEGMEPALRELYLKQIASNPGDLPDSNLEGFRRVCSR

HKYTFLSSLKYANRYAEEVPCKLSTITNAFIPETNGMALVKGSPYLGLFRQMIHKLRATG

ILQRLKKNVDSKLSKNKDPLDISMDFISVTPIFGILIVGLIGAVLILLTEFVVHRIIRSM

KITYYVGIIMLNNDR

>ZnevIR122

MISNPSRIKEDRGNTAKMSQIFKINLLLLLMVAHCISMDTSNVVEIVSQVKKHFRSIFVY

LLHYEEEYQFINASTLRVGKMLSARNIQVATASFSVIQAILSRDIRPLCVSTSGSKSIKQ

SIMKYFQNAEVSNAVWLLFLDSNTCMHEFFTDINIPFDCEFLVAQEADDHVVVLTEVYRV

SPSLPLQTYRFGEWTSCGALNWSVNGLFQRRNNFQGLVIRSSVNSSLFVTVITDDANKPV

DVGGYVGEVWRVLEPLMNFTXXXXXXYCYHGQWRDVVKMIHNGTVDVSIADILMKPQDLM

FADFIAPLVDVRTYVVIRMPSKTEVLWTNFLTPFSSRLWIAVLSLMVLIAMSLAFTDRAN

LCRRDNGILKTTNYNYLNSVFYVIGTFCQQAQGSTLRRASSRIVFLTSHLTALVFLASYS

ASLISSLTVRRPALPFETFQQVLEDGSYRLDVLSNSGELDYFRLNTGDALMRQLYQKLIV

RHEADLPSSVLEGLQKVCRGNRQAYFASQHAVKAFLRNLSCSPVIVPRAYIPGTISMSIA

KNSPYQGLFKYNIQNMRCSGILQRLMQEAFPARLPQSELKLERMDLATVIPIFSLLAIGL

LLTTLLLVIEMKLCHRRWITRKMCRIKFR

>ZnevIR124

MFLNILMLSLLFASCLAHNFVEVLTAVQSHFRSGCVFLLHEENSFLRTAAIVNDLKSLRR

KHIQATALFKATNTLECQHNRPLYVIRSTSAKYKTSIKSFLNIGRQSYAIWLLFLDKNSH

LGTVLSNINIPFDCEFLVAQEADDHVVVLTEVYRVSPSLPLQTYRFGEWTYRERPNWPTL

SFYQRRNNLQGLLFRTVFSEKLLKDNFNSGGRDYFFYRVWVILSNVMNFTIEYLPSEING

NGVLLDNGSWNGVVGILQRREADASIESLTMTSLRWNVVDLLSPLWVDRKYMFIKAATEF

ELSWLSIFYPFNCPLWFALIASILLLSTCLGIICQICHRNTEFRRNGLLQNICNSLFFIF

SVFCGQGEKLGLTTTSIMVVCVSTDLLVVVLLASYSASLMSYLIVRRPRLPFRTFSEFLA

DGTFTLGVHRFSSLLSFFETTEDPVINQLYLSHIAPHKDTLPMSHLEGYTRVCSSHKYVY

IHFLEFLEDKRNKLKCELTIVPGAFLTVTKSIALIRNSSYRGLFRYSLTKLRRNGILQQL

LYVTKHGNEVQETPLISMDMTSLAPIFTVLAFGLVATILSISLEFAIHKCKKQKQTSYRM

GNDWVL

>ZnevIR125

MTVIFKVNIGQCHFLPLFLLSAVACDSMKQVVTYAEVTTDLHRYFSHSCIILIHTEAGVN

GFSAETELVLIQKHLSSRGVPAVTISNSSVHKLAMVRRQAINVLFRGTEDTKRYIKQATE

PSTDRNFSIDIWVLFLDAESLLEEFFAGIYIPFDCEFLVAQIEDKYVSLTEVYHIGPARP

LQTYRFGTWTARSGLAWRLIGFFRRRYNLQEQLIKVAYVDQKHALTETHNNGSKKITGFF

VHVWNTIANFLNMKAVCQRVDTYGSRMENGSWNGIIQMVISNEVHVGVGDFTVTAGRSAV

VDCLAPLIISSLNVFIRVPDQSGIVVWGRYVAPFSLGLWVAVATAVCALSFCLWLTAALQ

AREKKFAATEAMFYVFGTLCQQGQPDSAVCTAVRAVELTSYMTSLVLFAGYSAYLVSSQT

VQHTVLPFRSLQGLLEDGSYGLGLAQNSSHLNLFDAETEGLKSIVYKTLISPHKNDMPAG

NLEGFTRVCDSRHKYAFMASLYVGQYWAMKLPCHFTTLPDTFYPECLTFVISKNSPYKKL

INWNLLRLRESGIQDRIRQEYLPPKPSYVNKSVISVDIISVAPILVVFFGGVVTAVAVLA

IEHIVCICLRQLDFFTTPRKSKTFLL

>ZnevIR126

MWTASALLFTTILFWSAGLDRQRLKKIAEVVFTVQRHLHSGCVSLLLSSALEGTNANLVL

VKLLSAKGITTTTLCISTLNVTHDDTNCRLNRPLYVMTHDATLELKQLTATMSPTGPTWL

LFSDSALKLDHLFTDVYIPLDCEFLVAREDLAGNVTLHEVYRMNRTMPLMNLQVAVWSPQ

FGVLWDNRSLYDRRNNLYGVTNVLWDNRSLYERRNNLYGVTIKATTFENPPNVEPKYNAD

TVEIGGTFGRVWSILSEELNYTTEVYLPEVNGAGASSGNGTWSGVIQLLVDGTVDVAVGD

VTMTSHRASAIDFSVPLLISRVCIFIRRPESSYQALNNILAPFSSELWFTIQGAMVGLAA

CLSLVYHVGRRYDGLERPDLYTLPQALLCVFEIFCQQGHDETPRSWSCRLIYWVSYMAAV

VLFAAYSGALVSFLAMQRVEPPFRTLRGLMQHGGYRVATVANSAHFNSFDQTTDETMRNI

YEKMFDKNLPNTTLEGLKNLCSIKNYAFMTTYDTVLLFWKNANCSFMALKEASFPETLSI

AFSKGSPYLGVINFKLQQMRDTGVLYRILNNAWSSEANDDRALWTSVSIDALTPMFSILT

IGILLATVLMILERQAFYLVQRPGKRQRSGHPQTRNGADF

>ZnevIR128

MGLTEAKRFVIFVLFFMHMGESRFSVDVFSRITLGVAERYHSKRVYLIQRSTGSQDDLQR

SVNFLKIISSMAKHILVATLPTDKLHEARCLHGSVFVTTLTATALQSLLLHVPCSGTKWL

VFIAADTLLVQEFIADVNIPFGCRLLAAQSEGDSGRLTLTEVFNFPPTLPPQMYRVADWC

PDRGFTWYTSPFRQKMVDLQGAVIKGAYIFDPPFVNSPLEEGHPDYMHNLWREMQRRMNF

TTVYLRTEDRILGYKDQYGAWRGVVNELVTRKADVGINLLAMTTERLEAIDFLPPILNDR

MMVHIRKDKMEISTVRHILSPFALKLWLAVSLVIIVFAVLLAVTAAINLKYSHEKVLALR

HFSLQNSWWCVLGIFCQQGQDITPRSWSCRLVHATLCLTAAVLFAAYSATFVSFLTVRRY

ELPFSDFRGLLKNGKYRLGILHGSSTTNYFQKARDPILLDVYKKLIQPNEDSFVADDIEG

LRNVCHSHNYAYVISIYNSIPALSKYIHCSITGVPEAYYSYTASIIITKDNPYKKLFQRH

VQEMRRSGILNRIRKDAWWEDLEDDFRGASAGGTTLDTVTVFYHILAAGLLLSTFLVVLE

KMWWKMRVQFRNKS

>ZnevIR129

MRTHTLLSLLALSRAASLSVETVVDVTNSVMRRYLSTCVCILQSSLHQDWLRALNSAKLF

KSVSTGYVPVSSGSPDMFAKLPHIHHANLLYVIRADLTTPTILKQLSETGNMSKGKWLLF

FDSMTSLEEFFADIYIPLDSEFVVARSSNGTEDGDSGISVVEVYNPHPNQPLRENTVATW

SSGEGLIWTPVSFFDRRGDLQGISLKCVMVKEPPYIQYPKDTALETSHVSGYSIQFLDLF

KQKMNFTSEFLVPEDGTDGSKKVRGVWSSFIGTVASRRAEIGLNLMTFTSSRLDTIDYVH

PLADSRVTIFIKDPDISFDGVTQIVSPFTARLWEVIGLTMLLISALLCTLFYNGSETTNY

RLYNSIFYVFYIFCQQSAGISPRSWASRLIHFTTHITFLIVFIAYSANFISHLTVQRSRL

PFSDFQGLLDDGTYGLGVVYNNAHEDYLKNSKDPVIRDAYRTLVEPKREELPRHTLVGLS

RLCDETKYSFFCSKITASGSMQNLSCNIIEIKEMSYRITLSFVINKKSPYKRFFNRLSLD

LKSTGIIDRTNKAFWPPIMEKGTNEHHTLVTLDMVSIVYVMWAAGVSISVLILCLERRLR

ART

>ZnevIR130

MMARDKKSDQGQISDLNHWSVSMNPKLSKACTLFVLTLSLCFQVNSFNFNIITSVTEDVI

TKYSSVCVYLMYPKHHQVSYHLAKMKKSLSDKGVFVAARFIDREPEYLHSTRNRPFHVVV

SSDIHTKYALSQISKTSDLSSGTWLLFMDTKTSLEEFFADIHIPFDSDFLVAQHSSGVSE

ENYEVSLTEVYRVRDTLPLQHLRIANWSSVSGLSWTTSNIQQRRDLQGIAIRCVFIPEPP

YLSVGGYHGKDPVYVNGYSVELMKVLQYRMNYTTNYYIPDEISYGTEDENRSWNGMVGEV

VKRRADVGLNLFMLTTKRAGAVDFFPPIFNTKMMVHVRMPGIDHSSFRQLVSQFTPLLWV

TAIITMVILTLFLSATWYAAVRYNYHREVVSYRMHESWIYTVGIICQQAHHVTPRSWPCR

LVYLTVYVTALVLFAAYSAIFISFLAVRRFHLPFTDFQGLLQDGTYVLGVIKNSERESYF

KKADIPVLKQIYKSLIEPYRSDPPLRDEDGLTRVCKEQTYSYICTQVTVQGLARTLPCNL

VEIPQAYFSVTVSMIIQKSSPFKRLFSHRLQELKRSGLVDRINKQIWPEDTDDAVQQARP

SVTLGTTGIFFVILSAGVVLSVLVLLAEIWWQKQCPVRRRTSRED

>ZnevIR131

MVLINTFTAFSLYLTVKYLYVQAYDYNYSLTTVAAVTTDIATRYHIHSIYLLHSSYIKAS

DLRNCISDDAINIYKDVLNKMNARLLMQTSVKSLDDIVHVADKSLQHLHVIFSSDRETQR

SITEISSTNRLMKRRWLLFLDSTTTPKQFFARIFIPFDCEFLVVQTEYGDVEVSVTVVYH

VHPTRPLQMYHVANWSYGSGLSWYTIPFHHRRRNLQGIVLKGAFIEDAPYVMNKRPSGNL

TDQLSGYSLEFWNLLQRLANFTLEYHVPEDNSYGSKETNVSWSGILGMVIRHEVEIGVNL

LQFATYRVGVVGFLPPILVSKVSVYVRQSDMAYDTFIQILTPFSTGLWGTILPTVLIIIL

IESAIFKIRVHHELPHETNNRRRLLDCSLRVFGIFCQQGNTTALKSWSYKLVYLTAHFTA

LVLYIAYTATFISSLAVTRQGSYFKTFEDLLNDGTFKIGIRTKSSYEDYFKNSSNPVLKR

VYEKMMKPNVKTLTNSFEEGLQHVCKESKFGFLIGQITFQGLAQNTSCKIVNVPKAYYTT

AASFIINSGSPYKKLFACLIQDMRRAGILQRIDRNYWPRDLEEIIRDSPISVALETVSVY

FVLLVAGILISAVLLMVEIQFQK

>ZnevIR132

MYIKIINKTLKSLALITIIWPCVSSLQLPIGVITDITTGVLQHYHSTCVFILQSTQEPGF

DNDKRIALFLKILSKRHIIVSTLTFESMQYLTEDIRCLRLFVICSNNTENIQESLAEVSG

ISSGKWLLFYDSVTPLNEFFRDIHIPLDSQFLVAQWSSHEQARFTEVYHIDHRLLPLQIK

WVGNWSFTAGLAWTNTSHVHRRSDLQGLIIKSGFIDFSNILYATKFRRNKPIQIRGFCYE

IWNALRKKINFTNDYYMPEDRVFGSLENGKWSGLIKMLVAEEVDVIILIVMKTPNRMLAA

DFFTPLVKTKFSIYVKKTEAVENTLKQFLKPLSMNLWRASCVAMVLITIVLTALWHTLKK

HETQNYNFHSSAFYVFGIFCQRGLDPTPQTWACRLVILTAYLTAILLLVAYSAKFISILT

VNRYKLPFTDIKGFSEHGNYRLGMLPNSAQLSIFQTSADPVLRDVYRRLVVPSKHLPLTN

EEGLASTCQTNNYAYLTLSLSEKLIRPYLSCSIVEVPNYFIPVSMSMVGKKRSKFKNLFN

YYFTKMHEAGIVKRNEANLIPLKFTEHEQQLPSSVSLEKTSPIFIIFLAGVSMAVLLLLF

EISAHKIKQKDAHMRLE

>ZnevIR133

MIPVMILCLFVARVKSSEFSVDTVVAVSVGVLDTHFSCCVFLMCHTHHQGLREDWQIAAL

SSSFSKSRVMSVLLSGEVFLSSKREFCSRPLNVIRSSDHTIRRYLQQVMVQCPLCLFDKA

VSASDNFRRARWLMFMDSSTSVEEFFAGIHIPFGSEFLVAQWSAQRVQLSLTELYRVHYA

SPLQTFCIGNWTYNGGLSWSSVPFFTRRKDLKGIAIKAAVIPYNPYLTVAKYRDKKPVKY

AGYLTELWNEMERRMNFTTVYVAPRDAVFGFRLDNGSWSGAVGMVVRKEVDVGISFFAYM

VDRMDAVGFLPPIWNVKLMVHIKQPGMEVSSLSHILSPFVSTLWGAVLSTIIVLTVILST

TWYMNPEYKVDAGHTPYNLYESWLNVFGAFCQQGHDETPRTGASRLVYITTYLIALLLFV

AYSASFMSFLAVRRRDMPFADFNGLLQDGTYKLGVLSGSARVEFFKKSNVSTLKEVYRKL

IHPGAGNLPSSDVEGLQRICTREKYSYVVSDTTLRGLGRSAPCSIIGVPQAYYSITASMI

ISKTCPYKRIFSHHVQEIKRTGILTRIEGTAWPPKYTEILDDPPSSVTLETVTIFFIVLL

IGVVTSVFVLVVEFGLHKLRQSFQWVTF

>ZnevIR134

MFSWGRATFLLLAAVDSGVTTLPSETTAFVLDALLRRSRQPCVIFIQVQTGDTFHQGTFL

HTELKKVFGQRALTTGTCPFYRLTNASQQSGCREIPPTYVVLTSDPQTRLELSQFSMKAD

LSSGRWILFLDTETSVEEFFADIHIPLDCECLVAEYSPVMAQVSITEVYRVQPNRPLQVH

RIGYWSRSDGLTWTALSSFYQRRDFRGVTIKGAQVSELHEVVTGNKQKKAEEEEEEGDSF

SSQVWTILEQSCNFTTKYVKPSHVSSAEDTIHILGSMLSKSEADVSVNVFDLDAFRLLQS

DAEYLPVVWTYKLATYIKQPSLLESTPYLIVAPFSASLWFRLVVILVFLTATLSASWYLG

ERHVRQRNDQVFTVTASCFGVLRGFCQQANHTTPRSWSCRLTFFTVYYSGAMILTAYSAV

AIQHLAFLKPVLPFNTFQGLLDDGTYDLGMRAQPSIFVIFEREADPAVQAAYDKHIKPHR

ENQPDDIEGLRRLCGPKKYAFMTDPLTFERYKRYSTCEVVQLPKVSLTGRGSFLTARGSP

YRRLLRRKLHDLTRFGILSALKKRVALAEKSADSVLRTQPAITVAAVKFILAILAAGVAL

SFAVLIAERQCCCWRRRRSANMQGITLVR

>ZnevIR135

MSSALSFVLMTSCALSSAPFAVDVSSDIIAVIRKHYHFSCVFLVHDDVEETWNLLAKLMS

LHGVTIGHVHAGILGNYSEETKCDDKPPLHFLPSSDRKSQQLLHELSKGSSLRSGKWLMI

LDPATSVEKFFADIHIPFDCEFLVAEIVDVPGKGGTSVALTEVYRVQPAQPLQKYRAGNW

SPPTGLAWSTLPFHQRRPDLQGITIRAAIVADGIFATVKKYEGQRAVEFDGYMIELWTAL

EHELNFTTQFFVPEDGVFGSLDVNRTWNGMIKMILEDKVEFGIGGFVFTSERMAAVHFLP

PTRTFKMKVFIRQPKLEETSGSFVVSPFTGGLWTAIIITAIALTMALKVAWNVRAKSGLQ

KSEDWASSVFLIFSIFCQQGDIRHLGRSCSLICLSAHVTAVVLLDAYSATLTSLLAVRRH

ELPFTDFESLVNDGTYRVIVGSGSAHLNYFNNPRNPVLREVYRKLIEPGIDSLPKSVDEG

LAKMCDHPKIAYVASDLFLRIARSRRACSVVPVDKASYLLTESLVLSKKSPYRRVFSSKV

QDMRRSGILSRAARHFWPPLMPDMAQSTLSTVTMTPVGIIFVALATGTLTSALFLVLESV

PQRSKQRKGKRRERERERADANFKNGNDVFWGAAPGRN

>ZnevIR137

MNIVFVMCFIQMGASQFPLDSVVSVTIGVMDRYHSSCVYLFHAARIQDNLKTHSLLVLKK

LLKAENISVGSVSMATNPDALDGMSCQGNRPLFVLASSCETTRSYLNQVSVTPLISRGIW

LMFFSASSIEEYFTDVKVPYDCEFLVGQSLSRKGIEDFTISLKKIYQDHPSRTSKTHSVA

NWTSSDGLIWPPKPLLNRRENLHDLVIRVGVPGEVRSDINSKCRRHTTFCRTSYEIWNTL

QDKINFKSDYRSGPRRNFSYENENGSWNGVVGMLALDEADVAIGKFAMTSRRLSVVSYLS

VIFPAKISTFIKKQRSNSSWKNILAPFSPEFWWVLLASLPVFTVSLSAVWYINLSFGMQE

GKEMYDIHNSWIYIFGSFCQQGHDIATSLWSCRLLYLTSYLTYVILFTAYSAIFISMLAV

HQFSMPFSDFQGLVDIGTYRFAVVGLSVYLDLFYQATDPALKQVYQRLIVPNINNLPKTM

MEGFQRICDENNYAFAVDDELVSGRQLSCDVIAVPYAFFRTPASMIVSKSCPYKKLFSLY

IEMMRESGILKKLETYTSVIPAAEQVPGTQASLEDISSVLVIAMIGVLASVICLLAEILL

FRFRLYFRRQMFNPTIPFSD

>ZnevIR141

MKLMILAVLITSSTCLYVNNTVNMATAVIQHFRSVCVYLLHGYDTXXXXXXXXXXXXXXX

XXXXXXXXXXXXXXXXXXXXXXXXXXXXXXXXXXXXXXXXXXXXXXXXXXXXXXXXXXXX

XXXXXXXXXXXXXXXXXXXXXXXXXXXXXXXXXXXXXXXXXXXXXXXXXXXXXXXXXXXX

XXXXXXXXXXXXXXXXXXXXXXXXXXXXXXXXXXXXXXXXXXXXXXXXXXXXXXXXXXXX

XXXXXXXXXXXXXXXXXXXXXXXXXXXXXXXXXXXXXXXXXXXXXXXXXXXXXXXXXXXX

ITFIHHPRSSSPGSEHNDPSAAAKPGSIPGNLSLSACQAPSAILPESLLSSHISFPLAEF

SSLGRGRSHTELRKEVGLARTVRLSGAKWLLFLDRDSVLEHFFADADIPFDCELLVAQPG

DSRHVMLSEVYRVSSSLPLQTHRLGNWTPVGAFSWPTQTFYGRRNTLQGLVLKTGTIEHL

LTVITKEENKKPMEVGGYYGDIWSALKNQMDFKADYYTPKDDSFGVKLDNGSWNGVVGMV

VGGGVHVSNAAFMYNAPRMRAVDYLDPMSVDRMVIYVRKADSTDLVWMSFLAPFSLDVWV

TLSASLFLLSAASLLLDRASPHRRRQEEEGLLLEFPRALFSVLKPFCWQGSTDDRVEVSW

RVIQLTANLTALVIVVSYSASVLSRIMTSKGKLPFNSFEEFLELGTYTLAVGRHGGHFTH

FQISGDPVLRAVYDRHMAPYVSSFPEGDLEALNMACARHNFAAVDLKNILPYYSDKLICS

LEEVPKAYVEFKESMIIKKRSPYLGIFRHTVHNMHRSGILDAISKRNTKKLTPITGLEFK

SVSLTQVTPVLVLLTTAAVAAMLLLTLENIIFRINLRFNKYNRKD

>ZnevIR143

MAILSYSMMLMIAVPCCGKREVQIPATTAANVISSLKNYFLSGCVFLLSTKHQTEMXXXX

XXXXXXXXPSSVKLGRLLSDKRILATEVSLQSFNHTLPEYRCCSNHPLNVILSGDTQAKS

SVRRLSDTTSLTGPTWLLLLDNTSSPKEFLGDSNIPFDCEFLVAQQNAGGLVMLTEVYKI

ATERPLLYHDFGYWLSETTFRFPSTDFYFRRSSLQGLSISTATIENPPITVLERTDGQIK

FSGYFGKVWRVLEKRMEFRTNFSFPADGGRGSVMKNGTATGMIRMIQDNQVHVAVDAFGM

SGIRATAVDFTVPLLSTRYCVIVKSPDSLKLQWDNFLAPFSVMLWVAMMATVIVIAIHYT

LLYYLGQRYGNQEADEIKFYTFSDSLLLVLGIFCQQGHDTTPRSYSCRFVCISMYIIAVM

IFGTYSATFISFLTVHENRLPYTDLESLFSKHAQNYISEPSRQFLDKTYGESYQDNVQPS

VLDGMHKVCNSKYSFLMSMDTALAFKNKVNCTVVPLPRETSLYIPQGFAIAKRSPYLGLF

NYNLNKLRSNGVLYKHRVYDSMRRTFSKENAAWTRVRLEEVAPIIGFLLAGIVASSVVLL

LEKVITTNMFLQQQNVFCCIAAVRYVDGGHSFHPKNSQINFLRPVGHNIRVRRAPQHQRL

REVV

>ZnevIR144

MPKGKVETKEIERVQEQTIWAMKHLPWNKFLIVLAMVELCPGQIEKALTLVETTAAIIGL

QQYFRSGCVFFLYSNGRTHYELRDKLTLVRLSKTMSQNTVTSATLNTGKGDVSLKYLRCQ

KNRPVNVLTSDDQNMMLSLKEMATQPHFARSVWLMLLSVGTEIDTFFSDIDVPVDSEFVV

AHRPEDYVQLTEVYRVRNGYPLQKQVFGEINAGNKVLNLTDIGFYERRNNLHGLIMEAVT

IQDNPYTSIAKNPDGSAIVTGGFFGLVWKEFENKLHFRTNFTYLEEKSYTYEVNDKTDPF

AKMVYDVHLKKYDIGVAAFRCVNERAKIVEYTYIMHRSKFVIVLKPADNEDMAWNHFFGP

FQPSLWFGLISCILVIAGCLSLFYYVGQRLAYPEYETPKQYTFAISIFYVFSMFCQQGHD

VTPKSCACRLVYWLGYVTAVVVLAAYSATLISFLTIQNKEPHVKTLEALAADGTYKLGMI

RHYVRIVKNDVDHGITNEIYDKIIEPDPNNIVDTNLEGFRRVCTTKYAFWTSLEAVKEVG

DLVNCNIALLSYSYVFMLGMVVTDNNPYRRLLNYQISRMQSGGVLRKHKLDSWAAHVPEA

QNMWTSVSVGQITPLLTILSGGVAISLALLLTEMVASWWQHRQTERKNAWSGQLSKQLRT

KYMFFRV

>ZnevIR145

YCNFCARVCQIRVCFLFVSYHIXXXXXXXXXXXXXXXXXXXXXXXXXXXXXXXXXXXXXX

XXXXXXXXXXXXXXXXXXXXXXXXXXXIRDSLLLMRLERSVNRQGVLAAAVSTRTIDQAF

NCRHNRPLFVSLFTDPTIQQSLIQLSSTIDISEAKWLQFINLEQVEEYFGKMYVPLDCQF

IVAQPGENGAVTFTEVYHIGKNQRLMTFKLGEWVPKRGSVWTNTTFYTRRGDLQGVSINA

AVISDGAICNITEMRNNKPVRVEGFFGTLWNALQYRLNFTSEFMKPTDGNYGSMNRRKWS

GMIGMLVRQEADVAVGAYIMTERLLQYVNFASPMGRTKIAVFVRSPRSLFETSWDSYLAP

FSIKLWVAVLCAILVLTVGLAVTFNIGRNIGNEESGSIALYSLYDSFIYVFGCFCQQGHE

RTPRSWPCRLVYLTAYLTAVVLFAAYSATFISFLAVRHTPLPFKTFEEMLQDGTYKLGIL

NHSVELNFFDTATEAVMQQVYDQLILPTMYDAPPDTLAGLQRVCDKSKYGFVVDSTRAAS

VMNNVTCEIEELPHAFIPATGTFVIGKHSPYRRIINSNLESMRRHGELGWLFHHAMPSMA

TDTSSSILPKVQIQEVLPVLVIIAASLVVANAFLVMERCIHRLVYRYRHRQRLCQCDARF

VNSACKTVK

>ZnevIR146

MAASTATVCSCASLILTPTLAFQQFTPIFVDLLISVQKYFSAGYISFVYPDNVTDKLSVK

QQMADVIKNLGEHDVMSTAASYRKLKDPPLRGKTPLHFVLSDSANGDLREILAEMSLKTA

VWLWQVEAVPSLHEVLRGVRVTLDARFLVAQIVDHCVLLSEVYQVSGKTQLRRFATWSRK

SGLRGPVRDDFLDRRSDLQGTVINVSSVEGPPMTTVNSESDRITGFVGDVWAELERRMNF

TXXXXXXXXXXXXXXXXXXXXXXXXXXXXXXXXXXXXXXXXXXXXXXXXXXXXXXTKLKV

APDNAYGSLTKNGTWTGMIALLVNNEVELAVGDFTVNNLRTKVVDFTVPFSESAVSVFIH

GTGSRDFGWSDFLAPFSKELWLAAITVMFIMAICLVAIHSFECHKDTRHGIGFLKSFCNV

YAIFCQQGVAVKPDALSRRLVYLGTCLTALTLSAAYSATLVSSLAVRINVLPFNSFETLL

KRKDYHLGVVNNSAVLSEFEETTDYVLRQVYDTFIASKRSNLPADSKSGLSKVCQNSKYA

FVTSCLVATYMQNSLSCSIKPLPEAFTKEYLSLATKKNSPYLGVINHNLLKLRQDGTLKR

LHAHTWRPTESDPGPPWRSVDILEVASVLLVLAAGVVSSVLLLVVEVVYQRVRLRVSGHD

RLRVNLQRRPQHFC

>ZnevIR148

LSSERGVMAKAVWLLFLDTLLEQFLEDVYIPVDCEFIVAQRSGENAVRLTEVYRMAKEFP

LHRRHFGIWSQDRLHVITESLLRRRSDFHGFTITATSMNSPPLVILDEEKMKMRGGLFGS

VWFSLEERLNFTTRYVVPSLRSWGTVYANNTTTGVVGVLADGQAEVGVFSMLITSRRMDV

MDFTTPILSPKFHVFVRQPDGLQLEWDTYLAPFSVRLWLTVVLLVLLIALLLPNLYSLGR

RHGIAEATQVPFKFKDSLFYVFGAFCQQGMEPSCPQLSACRVLYLTTYLTAVILLAAYSA

TLISFLSVKNAELPFSTLEELLKAGTHKLGCLEQSTVLSHFMDAKLPLYQQIYQRFLHRI

HGTLVPDIGVGLYRVCSSNYAFIALYTDVIQHPPSERGCRLATLPASYFQSSLSLGLTLN

CPYKAFLNYHLLTLYDDGVLHRLKDGLFASLNLQEAEHRAMVGGWLSVEMQHLLPILMAL

GTGMVLGATCLLLELGSGRRSVGKWTV

>ZnevIR149

MMRGVLTAIVILLHSTCVPQESSSVQAQISAHTWLSTSAHVVAAIQKRLHLQCVYVVHNR

AVDFDEQRMTSQFWKLLSLHGRQVAFSGPSGLGRTSVCGRSPNLYAIIAADESRSHCIQE

SVVTDKRCTLLSQFAQKRYLSQGIWLLFLNTTTKEAFEDSNVPFNSLVVVATQTRQRMVI

LEEVYRVSPHLPLVTRRIGTWNSTTGELSWTRQVDSRSHLHGLTIKAAANHNPPFTVVEL

NNNRAQLTNGFFGKVWKILEKRIGFSTDWFTPHNTTWNETTANGTELLQLISRKQVDMVV

SDVTITPSSADVVTFTVPLLTYRNHNTSIPHSKCILSRFSVFIRLPDSEDSDWSMYLEPF

RKSLWLTVLGAIPIFAIFLSISQYCGRVLRKEESNGRDAQFSFCNSVFYMFGALCQQGTD

VTPKETSGRMVYSLFFLSTSVVLAAYSGLLVSFITNKYGALPFSDLDGLLRSGIYSFGVL

NNSIPFTYFSGAKPNTTLHRIYSEIMTKDPKNFPKTDEDGFQRICNEKYAYMAPTEYVVS

VIEAANCDVVPLPRQYFEKILAIALPSDSEYKNLINHNLRAMTNTGVLKKLKEDTWTLSS

PKPKPHWNSVGIKHIAPLLAVLLVGIVTASVLLVFELSGVPVIKKSLLPCFERRLRRQTS

REFWYLKSG

>ZnevIR151

MLQNVLLCLLVPITRVALKKPEIAENRGEVISELKRHIQFKSVILLQSSDHHNAWSSQMT

RVQLARYLLIRFIPTTAMATNSSQQPLQSTHPGQLVVLLTSDPTFLRNVFEKYQLANVVW

LAFLELGQSLQDLAGDSNIPFDVQFIVAQPSDVETRLFDVYRVAPGTPLRVQLLGALPRS

IAASEQTHEYVYRRRTSLGGHTMRTSSLNNSFLVWVNEDKTIGGFYGKVWANLEQHLNFK

SDVIVPASGLWGRKHINNTWDGVIGMVNRGEAEVGVCDIAMNAERVGVVSYTMQIATFHR

QTPSSCSYKVFIRKGDEDVSWVSFLQAFSAGLWLAIVASVFVMALALKICFHVGLLQGKE

HSRHNKFHDWLFATLGALICLQGQTSAPLSVQCRLVYILPYVLGSVLMCAYSAAVISFLT

VHKTNLPISGVEQLHKDKTYEVGAVQNSSTFVSFQESGEKLFQAVYKERLQPNQARMPKR

VLSGLRRVCQDKRYAFMAPVEETVEHLPYLSCAVIYLPEGLFEMKYGIVFNERSPYVGIF

RY

>ZnevIR156

MASYHFQIHLAATVVLCLSFPVITSENLDNTKHAVDCIQSIASQYFIGSDSLTVVIPLFL

EDATRNDFSLESNEGAYKLLPKLHALTTLPLSVIRPSIMAKVSLKSDVYRKETNYVIILR

DTCKLLMKEIILILRGNMQSITFLPSWNSRARFIVVVTTEDCGPDTEEIALNIVEELWTW

KVLNVVVVTPTSEPFRSQQISHIQEDHKEVPVLDLFTFAPFKTSINLWKRVQSVFLIDQW

SSFSRFLHNRHLFPSKVPHNLKGCPITVSTIVYEPFVLDPDVIPNDKNSTVLRYKDGFEI

RLLRLIGSAINASLVFRPPPDNGELWGRNLLNGTWTGIRGDVVHGKSDVALCAIVVTSEN

SVVMDPTVTYMRGGFVWVVPHPETFPRWLSVFRVFKLFAWLCIIVTILLASVIMWRLSRT

PINREDAKRTMSANIFDAWAVILGISVPEMPRCLHIRIFFIFWVIYCLAVNTVFQAFLTS

FLIDPGLLPPIMNLDELLDSGIEYGYHPLMDRYLDSDNEKHREILHNHKICLNTTACLER

VAVKGDFAHFLSRQVMDYTSYYKFQDANGDSLIQPFNEDFVQYNIAMYLTKGSPFLDRFN

DIIAHAMQAGLLDLWYNNEIYWAKLSAHKISEIAADDGYSALSKVHIQGAIFLYGIGNVF

GVLVFVFEIFYRSFIANVRFKRTGVI

>ZnevIR158

MRAESCYHLPHLIDCIETISNRFFPPRSSVVLSVPSRLPNSELLHKLSSRHTDTTGLLLK

RLHHSVQRPIVVFHTGCVKVPCYNHDKHGSYILWTSVGIGTQLRQLKSYGDAWNPNTRFL

VVWDDPIVDNRRQMQAIVEEMIRWKILNVIILVPSKDGEEEFDVYTWFPYQPPSGNCGNF

VNVILLDKWINKNGGYFSKNASLYQEKIPRDLMGCPITAATIALEPHVISDQHKNGTTYN

RGLDVRIFLHITQTMNASAVFTPPTPELWGRKIQNGSWTGIFGEVYNNRADIAFSSTMLV

ITKFGDLDFTTPYGTANFVWVVPCAKPYPPWSSITRVFSTGMWILVFVVVILAAAVMLCL

SVFSANTSHEVELYKNMCGCLTSAWAVLLGTSVVKMPVSIPLRLFFILWVSCSLAVNTVF

QTFVTTYLVNPGQQKQIDSVKSLLDSGIYYGFHPDMDAFLMDDTNLILSEILKQREACSN

IVSCLQRVAEKCDFATLLSSDSVHYMNTYKFLDRSGKGLLCTIKAPAFTAYKTFYLPKHS

KYRKILNRMIAVANEAGLINYWWKEILITSRIKAGSIKRHTLQDDYSVFLLTHLQGAFYL

LMLGHFISSSCFVGELIFHWLRSM

>ZnevIR159

MFTLVVSVLWQTPTQVQQQMVQCLNDISLKHFTPVHALVVAYYNSQLQESLSRILENETQ

ERNKLYWERDFGNKLLQDLHKSEKWSLLLYLNNDKWNEEMPQSKHGSYIIISLHHHHKNV

VQDVSVQIKKLRNDWDWNPRAKFIITLPHAEGNSTKQLAADVFNELWRWRVINMIAVIPA

LGPKNTTDAVPVLDVYTWFPYRPPGRCADVRDPVVLDHWIVDKYGNGSFLNNATLFPEKV

PKDLQGCPIVASLFELEPAVMSKKFTTEDSAKTVFNEGIEIRLLQEFGSATNMTITYTQP

PNEELWGLPLKNGSWTGASGQLIQGSVDMAMDLYFYRCDVIKEVECLTPHLIDHVRWYVP

CASPFPGWMSLIRVFTLSLWLGFLVSYFIVAFIMWQVVKKSNMIFTQSIENQGYTSLVKC

LMNFWAIILGESVPNNPPEKPVIRMVFLIWVLYCLAINTVYQTYLTSFLVDPGLQHQISS

DEEVINSGMEYGVPSAISTVIPHVTNCRYQHLLQCDDHKGCQNRIAFKRDFSYIYSTFSM

EFIIAARYTDANGKQLICSFEEIILSQLITMPVPKGYMMLDRFNSIILHLLQAGILEQWF

KDIKYTTSLSSRVETDMLSGEYTKLSLLHMQSAVYILLLGLFLSFAVFLLEMLLCMNLRF

LYP

>ZnevIR160

MFTLVVSVLWQTPTQVQQQMVQCLNDISLKHFTPVHALVVAYYNSQLQESLSRILENETQ

ERNKLYWERDFGNKLLQDLHKSEKWSLLLYLNNDKWNEEMPQSKHGSYIIISLHHHHKNV

VQDVSVQIKKLRNDWDWNPRAKFIITLPHAEGNSTKQLAADVFNELWRWRVINMIAVIPA

LGPKNTTDAVPVLDVYTWFPYRPPGRCADVRDPVVLDHWIVDKYGNGSFLNNATLFPEKV

PKDLQGCPIVASLFELEPAVMSKKFTTEDSAKTVFNEGIEIRLLQEFGSATNMTVKYIEP

PSGELWGVPLENGSWTGTSGQLVLGLVDVAMDLYFYRCDIIKGTECLTPHMIDHVRWYVP

CAAPFPGWMSLIRVFTLSLWLGFLVSYFIVAFIMWQVVKKSNTMFTQSMENQGYTSLVKC

LMNFWAIILGESVPNNPPEKPVIRMVFLIWVLYCLAINTVYQTYLTSFLVDPGLQHQISS

DEEVINSGMEYGVPAPVLSVVPDLTGYRYQRLLHCNDYKVCQDRIAFKRDFSYIYSTFSM

DFTIAARYTNGDGKQLICSFEEIFSSQLITVPVPKGYIMLDRFNQIILHLLQAGIIDQWF

KDIKYTTSLRSRVENDVLSGEYIKLSLLHMQSAIYILLLGLLLSFIVFLYEILFCRNAQG

YSKRLSGF

>ZnevIR161

MLFTASDLHTGKSSISQRKIKNSLKPVICIPYLSTKTFEFKSFIFQQIALLILSLPSTNT

GSGYQYPDGQLVVDSALAVGVHYFTPGGTVVVSLPNTTLLAPDFKIHASRKLGKLALSTD

GNTEQLIDNLTQRLHSSSLWPVVVTCPADGIIKHDPTNWDTRNDDVHSSYVLVTMQHKDW

QDQAVYSLREQIQNLETIPAWNSRARFVIMLLAEVTNAREVAKDVLQELWQWQIMNVVVL

LPASNINSKMHIVLVYTWFPYQSPSGLCGELRDSVHINSWILDEEGGRFLKTSPLYPQKV

PYNLNSCPIRASTFEFYPFVICDKRLGIGTNTSITDGLEIQLMQCIAEGMNMTLILRIPP

GDERGGIQLENGTWTGLRADIVNGKADITFASLLSKLEDHLIFDDTISYFTDRFTWFVAR

AKPYPRWLGMARVFDPMTWLVGFLVIILASIFTRILLSFKTIRHNEVPGNYWRFSVCLSS

MWAVFLGEGVPIMPCSLPLRSFFLCMIIYSLAVNTVFQTFFTSYVVNPGLLHQISNVDEL

VQSDLVYAFYYVLDKFFTADFLHKLEPRVQCEPFKCLDYVATQDNYATFCGRALLAYIVD

ELVKQEGKHEIYAFQEDSFQLHSVMLMPKGSHLLDRINVVMTHIVEAGLPNQFLKSILDA

RRIEAGILALQDLTDEYSALSLNHLQGSFFFLLMGLALSLGMFLFELLKICK

>ZnevIR162

MLRFLFVIISSSSQVTWLLLILAAFRKEVLGSLYTHDTKHLDQHAMDCALKISEKYFETG

RTLVVSLPANNMSQKDEDTDKITNSAQLDTQPDTSGVLLERLHLEVSWPIIVYRALSKIT

KRDPRDWDTRNSDKHSSYVLIARHRDEVASQIQQLESLLAWNSRAKFVLVVEGHQAKNND

QMLKSILKEFWKWNIFNLVVLLPILLTISFTSVGVYTWYPYRLPSGRCGELRQVVHLDTW

LSTGNSTGRFLNDSPIFEQKIPYRLNGCPFRVSTLKFHPFIIYDNSTLDGSEIRLIRNLA

EKINVTLNLSVSMAPERKGQQLSNGTWTGLRGEIMYGITDMIFGHVLANLDDHLLFDDTI

VYSSDGFTWFVARANPYPRWLSMVRVFTPTVWFILLTVIPAAAFVMNLLSYRGGDGLWSY

VKSLVSFWAVLLGVGTDMPNRVTLRVFFFSWVIYSLAVNTVFQTYVTSYLVDPGLQHQID

NADELYESNSVYAFPDTIDKFFTSSFLDKLKPRFTSDPVICLDYVSSKDNFVTVAGRKLV

EFFSEDLVKRGSKHEIFPFREDLFQLSTVMLLSKGSPLLESVNMALTSTFEAGLVNKWFK

EIVIEKRIKAGVREIPVFIDEYVPLSLSHLQASFLLLFLGLGLSLITLLLEKSLQRK

>ZnevIR163

MADLHEATQVNQQIHLLADCVLRISVQYFSPGRTVVVSSAFNHENRTFQVTETLVDIEDI

VMEGLNLIGLWPIAVYRPAEDIIEHNPDIWDNTNDEKHFSYILILRCHEGGWKNVLDYMT

KMIMQLKTSPAWNSRGKFVVVATHCVSSNVQNELKKILEHLWDFNIFNVITLYPSAQETV

EIFTWFPYQFPSARCGRLLNVVHLDTWVREQDTGIFQRNLTLFTNGMLRDLQGCVFRAST

INFQPYVICNENATVEGGIDVEVLRAVTKKLNASLKFRVILEKERKGQILSNGTWTGLKG

DLKYDKADIIIGSFLSNFDDHVEFDDTNTYHTGRFTWIVARARPYPQWLSMGRVFTLTAW

LLVLAFVFIVGLLMRYLSKSTYYKTNGEWGISKCILSAWATLLQAGIPKMPRNSTLRVLF

IFWVAYSLAMNTVFQAFFTSYEVDPGLQHQIDNIDELFNEPTVYAFSSPLDRFFTNDMLK

RLTPRIRCEPIRCLAYVATVYNATTFVSRVLVGYHREEQIKQEKRHDVHPFREDSFQIHS

IMLMQKGSPFLLYVNEIIRRIVEAGLSDYWKEAILEDRRIKAGVLALKSLKDAYVEMNLS

HMQGAFIFLLIGTGFSFTAFLAEILFEMFFTKFRINNCKLH

>ZnevIR164

MADCLTTVSGRYFPAGHTLVFSWNSHAEEKEDLHGAGTWEVVETSLLARLHLSGRWPILA

SRTTDGRDRRHPLDWGDKHGSYVMAVLFQNNDEERALRHARNQIEELACLPSWNPRARFV

IVMTGQDIRNSQRGLIKVILKELSDKQVLNAIVLLRPSKPFSDLHVLTWFPFTPPSGRCG

SLKRVITVDAWMSDSREFSLNKDLFSRSVPKNLGSCPVKVSTSHFPPFVIYPDKSDATIS

SVGGVEIDMLRHISEAMNFSVVLPPRADATDSKLENGTWTGPIGDLLYRRSDLALGAWCF

TLEDSLKVDGTNSYFAEEFTWFIPRAEMYPRFLSISRVFAPDVWLLVFVAMLFAGVLFHV

AALTQTARESDGYKHFTKCLFNAWSVVLGVGVHEMPRSDVLRGIFCVWLTYALAINTVFQ

TYVTSYLVDPGHRHQIDSVEEVIESGSEVFVADFLQDFLSDDLLNQLRSWRNCGSAKQCL

AAASRSPGVVVLSGKVFAEYDAGERSDAFDYHESSRDFLHSHIVMVLKKGSPFLDQINDV

IRRLVEGGFPAKFYKDIVSKTKLKLLPEYSDEYVPMSVPHLQSAFVAMFVGTSLSLVLLA

CELLLKRTQAREQT

>ZnevIR165

MKVEIHIIPVLVCVFLNQLVQISGETQHHLLQCLHDIATRHFTLVSTTIVSDNEFPYKPC

NVSGTFKTTMFSDPIFETDMILRQLFKSEECDLLYFHENDGDRINPLPNKNHGNYIITSS

YCEHQHLLKDIVQQISRLHETRHLNSRAKFVIVSARAHGNLYNFASDLFAELWKWKIMNA

ILVAPTSETSNAEEPDVVPVFDIYTWFPYDPPGHCSDVRDTVLLDRWVSGDKGPGRFLYN

ASLFPSKIPRNFHGCEIRVSTFEYMPFVGRKKTTKSDPNKVNFDEGLEIRLLKLVSEKKN

ISLKLLDLPADGGRWGLKLGNGTWTGVTGEVIRSFSDIGSGNWWNRCHLIEEVECPIQYT

VDQVRWYVPCAKPYPRWMSIARVFKLSLWLGFLSSYVVVGISMWFIVKISNSISTVPIEN

QAYTSLVKCLLNFWAIILEESASNNPPHVAVIRLVFFMWVLYCWAVNTVYQTYMTSFLID

PGLQHQISSENELLESGIGLGIPETVLSVIPGVTDGRYRRHDSCYDVTVCQDRMAFVGDM

AFFFSKYNMEYFIAAKYVDGDSNDLVCKFDEIYCIQLAILPVPKGSQFLEIFNEIIIYLQ

QSGIREYWWKDIQYIATLDLASEISLPPGEYIKLTLEHLQSAFYFLFLGIIFSIVAFLSE

IFCRPK

>ZnevIR166

MVFWKFLFGLGVMVQVSGERQSHLVQCLHDIVLRQFTSELTCVVSYQIHDSPEIESKILK

DLNELNQPLLVVCNYEYDDHIIKPESGDKYGSYVLIHQSEDVLQDIRSQFIRLKSNSAWN

SRALFVVVIMESVGLPPQNQEKVETENAQVFAETWKHNVFEVIMLRSTRLSDENVRVDRG

TPTVVVVLRSTRLSEENFRVDRGTPTVDVYTYFPYSSLGRCGQATDCVLLDTWVTTKDGV

GHFLRNVSLFPPKIPEYLPGCPIKISTFEFEPVVMYPWHSDDGTEVVYKGGLEIKMLQMI

QKATNMSVVFRQPPSDGKWGILLDNGSWTGVLRELIEGVSDMAFGAVYYRCHISGDVIEC

TTPYILDETVWYVPCAQPNPRWTSLSRVFKLSLWLGFILGYIIISVLIWIIVKISNVVAT

VGNKSLTYTKLAKCFLNLWAVILGVSATEDIPKNTVVRIVFLLWVAYSLAVNTVYQTFLT

SYMVDPGLQHQISSVDELLDSRLDYGMVDTLESLLPDLKAKRYERRQICDDIEVCARRTA

MKGDYAILHSKICTDYIAAVRYVDSNGDPLFCQLDEVFSRQYITTSVQKGSPMLARYNDV

IQRVVEGGILDQWWRELRFQATLSAARNFTVPVGDYVPLSMEHLQSAFYILILGYIIPTV

TFLVEILSHKISRQ

>ZnevIR167

MAVNFVVLLGVFMLAACGGMQHQLVKCLHDIATKHFSPGRALVVSLSSDIINYREETNSL

SCEETMNTVDVLLEEIHQSVSWHVLILSPFGELERRTEDKHESYIMFGQADGVVQEVKIQ

LSKLKTSSGWNPKAKFVIVVMVRDVNKSVCNATADDIFFELWKENIVNVIVILPGKHTHE

ADNRTRNSREFVPVLQVYTWFPYQPPDRCAKSTDPVLLDIWVANSEGDGRFVHNSYLFPQ

KVPNDLHGCPIRVSTFPFVTMVRNLIRNADGDISYEGGVEIRHFNMLMNAVNMIPVYLPP

PSDGERWGTLVNGSWTGVIGHIIERKSDVAFGSMLNDNYQYSEGVDVADGYRWHVPCASP

VPGWLSLTRVFYPSMWLVLILALVFVSLLVCCLVKTSGSPGALAYSNFTVSPLNLWAVIL

GVSAPNAVPHWFEVRAMFLVWVMFSLAINTVYQTFLTTFLINPGLQKQISSEDEILNSGI

KFGYHPVTELSNTELSGPRYRNRQMCFDMNKCISRMAHERDFAVLIVKLFADYMTSVEFV

DTDGKPLFCHLDETFTTLFMAMYLQKGSPLLERFDQIILRGLSAGLIDMWWRDIVYVERL

AVAKNVTAYGGDYSVMLLMHFQSAFIMLILGFVCSVLVFLRELSCSSSTGSRNLRCGDSL

RHAT

>ZnevIR168

MNLLRVVFQCLLVSLSEGIDRHLCRCLSDIAKRHFSSRINLVISDNTESYTCHHVSTNFS

RTLNHFNENSSGIDTQTWDLFLSDLHLSERWSLLLTRPNKEINTKYIADRKHGSYILVPG

YGQIEDVTEYIRIQITNLKTSRDWNPRARFLIILTQSNNFTEARLKILEDIFTELWKFRV

INVVILTRALKTKLGKIDVDEKNLPVFDAFTWFPYEPSAKCGEIRDAVLLDRWILQGKGN

FLFNTSLYPPKIPKDFRGCPLRVSAFEYPPMMMRMKTEYGIIEYEEGIELRLLHQMARIT

NMSLLFRPPPADNGYWGNDLGNGSWTGLTGEIIRGYSDIALDNFWYRCHILNDMECLEPH

FMDSAMWFVPCAKRYPRWTSITRVFEANLWLGLLSSYITVSLFMWLVVKIIHNISHPDNE

AYTGIVQCFLNFWAVILEESAPNNPPNVFSIRFVFLMWVMYCWAVNTVYQTFLTSYLIDT

GLQSQISSEKELLNSGMTYGIHKVLLVQIPHLASKHYPRRIDCVTFEYCQDRTAFKGDLA

FVFSSLNMAYVIAERYMDGNGKPLICKFDEIISTQIISIPVIKGLPMIDELNNVIQNTKE

AGLLEYWLKNVEYIAILASANDFSVPGGEYTKLTLEHLQSPFYFLFLGYALSLITFITEF

ISRWKQAVH

>ZnevIR169

MCSFKKHVAFVIFILCLIEKTSGGLYLQLVNSLCDIATRHFTPSNTIVLSHNIPVDNLSA

SRNIFPDYYTFERVQENSFSELRSVVDLWHMILEEFNKIEEWTLLSFSANDGLEDQVPSN

NNDGYVLLSGYHLHKDVMKDIGHHLQKLKHNWGWNPRAKFVILATEISDTDAKMLATDIF

AELWMSRVVDSVVLIPTTNKYTTDTVNVLDAYIWFPYHPAGQCPDIKEAVLWDQWVLNNA

SIGRFLHNASLFPQKIPRDLHGCPLTISTFELSPMVMRKNTIKPDTESITYDKGLEVQIL

SEFAKSTNSSIKYRDAPPDGGQWGWDIGNGTWNGVTGEIARSYSDIGMDCLWYRCHLVKE

IECLRPHLIDKVRWYVPCAVPYPRWMSLTRVFKLSLWLSFLGAYVAISVIMWQVVKINSY

FSTEAALNQAYTSLPKCLLNFWAIILEESASNNPPDVVLIRAVFFAWVLYCWAINTVYQT

YFTSFLIDPGLQRQLGSEDEILASGIDYNTETSIISLYGELAGTRYKHMNGTDYIDTAEE

RVSKGTLAFLFSKYLVDYKIAVKYMDANGKPIICEIEEDFAFNFITIFVPKGSPFKTRYD

EVLLSMMQAGLVDLWWNSIKYTANLEQASDFNLPPGEYISLTMEHLQSAFYFLFMGYIIS

VVCFLLELSQFCNHRNHKKK

>ZnevIR171

MEELHRLEGWPFLVCNTCYEVKQGTRRKHSSYILLLRTPDFIRETRNQLTALKDLPEWNP

RANFVVILKQSIFPPTKPSDFVDEILTEFSNWNAMYVIVLLPTKISKLLSTENETNKKSI

KRSEIEEFLPILKVYSSAPGESFDLCGKVTKASLINVWVSGANDEGYFLYKMPLFRQKIL

GTFHGCPIRVSAFDYRPFFIQDINSGKNKDTISFDDGLEFRLLNTISKSTNMSVVFDSIP

VGGDLWGRPLENGSWTGILGRVLSGSSDVAMCSLYYVCHLSNDFECSKPYVFDKTSWCLP

CPRPFPHWLSLTRVFKLDLWLVFILSYVIYASLMWISATLNNMQGNKELSYTSVSKCFLN

LWAVILGAAAHGKIPRKSVIRTSFVLWVLYSLVLNVVYQTFLTTFLIQPGFEHEISTVNE

LLESGMEMGLPTTVDTVLPELTSKLYIRRTFCTDMSTCFHRLAFEGNFAVLCSKYNTEYD

VARKYTDSNGKSLICHLKEDFSLQFITLTVRKGSHLLDKFNRIISHVLEAGLMNTWWKNL

KYKATLRAASAFTGHNSKHTAMTTQHFQSALFVLAIGLILAMLCFVWERLHGVE

>ZnevIR172

MLWIVQLLIHGAVGHSDSLLDGHITTCVLQIIDSYIDEGFPVAVSLYRKSTSADSTRTRN

LSQDNGGNNMANYDLLQNLHFRMKWQIVTSSPDADEEKYNTGLHEIYDKHKGYIIRAPNE

TGFLSRQLKSLRSYRNAWNSRARFVIISDEVLPDSGDTAKEILKELRHLNVYNVILLTPS

RGGLRALDLYTWFPYQLPSGQCGNVKDVVVLDQWIMEGSGRFLRNVSLFPPKVPRNLGGC

NMTASAVAQPPFVMSSDRNTNERNILKYDEGSDLRLFLFIAEAMNVSVMITASEDTIYVW

PTKLQNGTWTSALGDIDNKKADIAFSDLLLNLDKLTSFDTTSIYYFSGLVWIIPCAKPFE

RWSSITRVFSMSMWLLLLISICVSAGFMYFLTKCHSNVVDEIGLYRTLLDCFSNVWAIHL

GLSVAKMPTTGHLKIYFIMFVWYCIAINTVFQAFFTSYLVDPGYQKQISSVEDIIESGLE

YGFYPGIGVLLPDNSDWRFKEILSHRIPCYDNSCVERAIEKNDFATISDSKFAEYLNTCG

NNSKSTLCTFTQDSTTKPMAMYLEQGHLLTEYVSRLIDIAVEAGLYNFWFKNIMDTSRIK

NAFIRSPILTDEYTLLLQTHLQSIYYIQGLSYCLSVTTFLGEMLHHKIWVKISPRNSREQ

LK

>ZnevIR173

MLFFVFLSISCWMLYTAVANMTDVQHLVACLIAISYKHFTPGSNIALSLPKRVQDNNNPH

ATLALNSTINNPEDSLPLELQRMAVWPITVFRHCDESPADSFDGKRQESHKSYIIVVYSE

EEDDVTEDVAEQLQFLSYSASWNPRARFVVTAVIKAQLYDSQETAKNLLQEISLWKIFDV

VVIVKASATHNTEKIKNDIQNFQINTWFPYQAPDRCSNVHEVTLLESWLMEGKGRLSKNT

NLFPNKIKYDLNGCTITAATFPVDIAVGPYRYVDKNDSTATPSVTYESGTEVRLIKSVAE

KLNLTLRFLPPPSKNEKWGDLAEDGTFTGLLGDVVYGRADVGFAVWPLHPKLLVIMDATK

SYLGDDWVWWVPCAKKVPRWKSISMVFLPSTWSALLVSVIFAVVIIPCMVTREPSELKIY

RNCGNSVSTVWATFLGVSIPRMPKTVPVRTFLISWIWYCLAINTIFQTFLISFLIEPGLQ

HQMNSLQEILASEKKYGYNEWFDMVVRDTEDEFSETILRNRVACNEGNEPPCLDWVAHHD

NFSLLCSRALLQYILTREYLDKNGKPLICQAGGLFFPVNYVTYMAKWNPLLNRFNDIITR

LLESGISDKWIESGLYLQRVHARTSSRRVGVGEYYDISLEHSQGIFAILLPGTILSVMVF

FLEVFYHMFSLK

>ZnevIR174

MKEQKELISLRRKLHYRMVMIFVLLMIQTVMCELNKTRLIEQQHLVTCLIQISKQYFDQQ

IMISLPRYDEQSFRKKVSGGTENKFLAQEGNNFESILLHKLLISGQSSVFVYRDADESGV

EEMHSKHSSYLIYLQRNAIGSLNYRLQCLKKNYFRNLKSRFVVILDKKFEDPEKEVTNVL

SKLWEFKVINVIVLLKLRAQDVLQIGIVPKDLKQTLEVRKFDNVIGIYTWFPYRDYKYCY

NTRKFYLLDVWIMEGNGHFILNSSLFPDKVGNSLNKCTVVVSTTEYYPFVEFPIYIKSTG

SVKAVYDKGWDISLLNIITEVMNVSVQFLPPSREKFGRFLMNGTFTGIIGDLAYSRCDIA

VGGLPLTPPFIDTGDHSAIYSRSEFVWLIPCARRLHGWRNIFRIFSSTLWLCVFLSISLA

ACIIHCLAKCTLSFKTPGTERYLDMSESSYKIFAVFLGTSVSSIPRTTPLRVFFLAWIGY

SLAISTVIQSFLTSFLVRSDMEEQVSSFNDILNSGMEYGFTPQYDIFFSMAGTSHEKDVL

GDRKNCGYEKRCLHRVAHKRDFAMLFSKVNYDYEVKHDYVDPDRWTILICTIPESFLGFY

FTVYTPKGHQLLGRLNVVISRILQAGLYDKIQDAHVHWMRLKLKVHGEETDDDGPYQLSL

QHLKVAFFILMAGHLIGLIVFLKEIVYWKFIRSA

>ZnevIR175

MSLVRLFLIASGLVQVARLISSDELHWVRCLVTIARRHLASGRTLAISLPTAERESRMAD

TGERLLGYLQHSHHWSLIVFRDSSDATVSTASMGNHCINYVVLVSSSCNSRELRLKLDRR

FSSLDFRHKLMPSWSARTRFVIAVENNCNGTNPRKLSEQIISILWIYKVTNVLIVMQEEY

YRNASTSAGRLRHQKLGLYTWFPYESPNRCTQVENAVLLGNWVMDGKGYLVQNSNLFPRK

IGKRFNGCPLTVVARNFHNMVEYKKPATNYSGSYTPVIERGWEISLLTIVANSLNMTARY

LPAPNSFESQNKLLDVVQALVFGEADIVLGGVESRARWGWEEYVDTTISYLSRRMLWYVP

CAFKHPRWSSIFRIFSPQLWLCLVTTLAVISTTVTLVSRCGGARSKPRAYWTVTDSLTCA

WAVILGVTAPALPRRDAVRVVFVAWLLFSLAVNAIFQSVLTTFLTESGYKSPVEDMDQML

ASEIKYGYHPVLDDVYRQSGDAHASTILRNRMSCPVHAICLKWAFTFKNISLILDELSTD

EQYASSRLMDENSKPLLCPIDDGVVMHGNHVMMMHVGHPLLDRINDIIQRVVEAGIFMQW

KNAKLRAGALGIYSPLNNYYSFTMNHMQPAFYLLLMGISTSAFIFILELIASRLGSGRRC

IFNRN

>ZnevIR176

MFVPLFFAVIFRTSLAFLQVNFPEHPLMECTKSISEEHFSLGRPLVIVLPLAEEYSNSDE

VRYLIETLQNSNRWPILVLDTSYEIERNTRNEIHSHGNYIILASGACRDINNFTDHIYKQ

LADLTVMDSWNPRAKFVVPVINTCSNYNTTKLSHAILTKLWLHKVMNGVVLFSESNDQGT

NVIQEHQTNSAQNTQCTLHTWLPYENSERCDPIRGTVPVKIFSAQNFNDVRIARIFERNV

GKNLHGCPFNVIHRTAEHFVVPLFKSDFSNYLLDIILVYEIAEKLNFYMLVTFPNYPNSQ

KITKMEYIKTLLEDGKADAVIGGVTRLDTRSKNLEATRGYNNVKISWCTPCAVKRPRWSR

FFGIFSPELWIYFILSVVLAVVVMVCISKFGRKFRLPEFAAYRNVQIISYNILAVVLGVS

ATAKPRTSPLRVFFFSWVCFSIAFSTVFQAYLTSFLIDTGYEEPITTVDEMLKSGKKFGV

SKENQKLFEDSTDSVNNEILKGMVFCPDYATCVHWARTYQNISTILFDLNTKQAKSIGIS

TDENNRSLLCDLEDGDIVNLEIVMGVLKGNPMLDRINDVIDRIVESGIFMHWKKGYLEQV

KISTKPTLSYTLADEYLIISITHMQSAIYLLMFGYALAFLCFFIEIAWHKLISKRRLCHV

KTTHKNTSHEQAQLFVNYLLHNIKRGTQNTELVPTVCTILSLK

>ZnevIR180

MRNVDEVLDLVFPELSRWILVVCKNTCERNRINYDDIHRNYVIITPIDTDNPERTPIILR

NQIIHLSRSNAWNPRGRFVVALSNSFKNSSLKVQRIFEELWDHKIINVVVLVPSFEERSE

MESVLKAYTWYPYENSDRCIEVDEVALLDTWVRGHFIKDADLFPPKTGLDLHGCPLRILT

QPTIFTVLDPCFTYVNRPGVYKISYRDGWEIRLLNIITRKMNMTEEYLLPVKDFWNLTDD

KGDFAGFTRELLQNRADLAVGLLVVRDDVPIEATRPYHWGQLSWYVPCGSIYPRWMSISR

MFSGSLWASVMMSVLISVPVIMLLERLSEDSVYTTGSNVLSDTWAVILSVSVPEMPRKWS

LRCFFMSWVFYSLAIDTVFQTYLTSFLTDPGVIPHEKNVEELARSDIKLGLSFRDGVFYE

DKNDVQSRRIMSKKVECEDQTTCFIWATKYKNLSILFSDILYKFLISINRPEETNSKSLC

EIEDGVVERGPVVMVLPKGSCLFDRINDILLHVVESGIFAEWVKITDYIQMVKTKAFFPY

GLSDEYFKLSLEHLQSGFYLLLIGYFASFIIFVAEFLRKFVLST

>ZnevIR181

MRTPEACSSKGPVLLFVISLIFHVAIFETDHNLQLHLVRCAVTVSTRHFSPGGTLLISVP

NCSASSTHDRTIGHTHEESKIYDQIVKGLQLQTRWPIVISRPGPIKHETSHLKDHSYVLI

TCFQVTLNDVLRNLEEQLNALKVRKSWNPRGQFLVLTTEKHSSGRLLVRKVLELLWSFKV

LNAVVVVPELPQALVLYTWYPYQSRDVCTHVKEVALDSWLFENGGQLVSNTTLFPLKIPY

DLKGCNITISALQVEPYTFLSKADNSEVACKDGVEcRLLQFIMDKLNMSFELKSPGEEMW

GDKLENNSWSGMKGHLYKNISDIAFGALLLEEELCNVFECTNSYIENYSFWNLPRSEEVA

KWKGVFRVFKPTTWLAFGAVCFAVVLLLWRIARNNNAVSSHTSSYANFCKCFLNVWAVVL

GVSASEMPRTSRLRSLFLIWLFYCLHMDTVYLSYLTTFMIHSGYEHQIQNVEELVDSDME

YGYNKGFDKYFNDLTDPTMVKMLRHRKHCDGYGVECLRRMTKNKDFALLATSYFMEYQIS

RDNVLQPGKSLFYTFQDGFLRNSFVFYLTKRSPLLERINTIIRHAAEGGFLDQWWREMKT

SWILSSAMDIQEESSTLTLSHLQSAFVILFLGLGLCLVVFVVELACVYGLKKNA

>ZnevIR182

MIMLQILTLASLGMLQVAGLLRDDGVLSVKQRHMVVCVVTIAKGHFSPGQSTLVSFPNAG

QGISRRTLAQYRTNTEMHMLCHTLQNMHEQMLWLIGTSSLYVSLNTGYETYWKIDHYIIF

TWSGEEQDSVTDAVIDQVQKLKDSSWWNNKAEFLVVVTEYHTKNDSQLALEILRILWTSY

RILNVIVLIPQTINLQPMMDTHDGNIKTSTFDLHTWFPYQSEIECADVRNTIVIDRWILE

GEGRFLKEVPLFPAKIHKNFHGCPLKISAIDVQPLVVKDNYIDEDNKMRPVYSGLEVECC

RSITTALNMTPVFVPVTTAGLQARVDLISDLEDGSTDVAFGAFPLHELIYSLVDSTISYI

DDYMSWYVPCGKPVPRMEKITKMFTVSVWLMIGLVFVLSVVMTWLIAKRAQNCAVNESSG

YATVSDSVQNLWAVTLGLAVRELPRTGKLRSYFCLFVWYCFSVSIVFQTYFTSVLVNPGV

KERITTLEELYSSDLVYHYIEENDDYLKYSLQSYYSEIKLRRTECGETEYCLYDLLRSQT

FAVIEHSFHIEYVTSIIKNPELCSLDVAIYRLSFAMHLAEGSHLVDTFNDIIYKIIQAGL

IVKWWNDLKTKYQLSAYDHYSMSPNYADFNDDDKDYFVFSLSHLQVAFYVIGVGGIIGFL

ALMGEILHDKFFKKP

>ZnevIR185

MKLFPILTVIANIHIVLASLLTSEERQLISCQMAVLNQHFIPGRRLLFSLPQTGHNATQQ

RAIVHTLKSSYDSHVLDFMLKTIHHESRWHLCVSTPDDESSSEPTSEVEGKIDYYLVVTT

PEHTDDEISENALQQFGSFQRLRSWNQRADFFVMVTGKTQQPKLFALKLFDKLWKSENIM

NIVLLVPTYDNATNKEEPMAFELYTWFPYRSGHCANVQEVTILDRWIITDNGYSFKDVLL

FPQKIPLNLQACPLKVSVAGSEPYVFRHDIQDGNIQKYRGLEIEFLHIVSKFMNMTLVYL

PTPAGSSVSQRTSILMSLSFGELDVVLGAFPMHLSILEFADATTPYLQTVFQWFVPCPTP

VPRIETILGIFTVPVWLTLILVLILTTATVCFLGTRSYESALYNSMTRSFHDVWAIFMGV

AVARLPRTSRLRIFLLIFLSYCFVINIIFQAFFVTFLVKPGFRRQISTFDDLMNSGLIYA

FQEGTEIALNSTTYYDHTKLNLRRMKCLDHEKCLERLMISQDITMISIPLQAEYLAFKLI

PIYKRWINVCFLNEDIHKMFFVMYLQLGSPLLDRFNTIILRTIDAGLVDRYWTMLKWEYH

LKSSGNSTYDGNAGDNDLYFAFSLSHLKVTFFILFVGYILSFIIFVGELFYATLSKHRK

>ZnevIR186

MKLIIAFTIIKAVFCVTYEILPSESRHVAWCLKNIIYQHFQQKSPILVVVPPSDNNSIPE

SVQNKNSYLVDSILQYLNSAELRPLHVFRPDLPVDFSNELPNLHHGYIIFIWSSEENDVI

QTVENHLEELRYSQSWNPRARFMVVVTAYDSPHSHLVALSVSEALWSLNGILNVIIVIEN

RDTTVTASRDVDTEETLVVNIYTWFPYRGGRCAEPAEVVLIAQCLSGSSGQLTTNEPLFP

NKIPKNLLGCPINVAVIDVSPFVIPTEDYTDTSGNATYKFRGLEIEYLLLVSKALNLTLE

FRLITDSVLTFPIGSAVNMVLDGLADISIGRMFHLAQYVGIAEPTIAFMFDTIKWYVPCP

KPTLRTEKIMGIFSPSVWFAMTVVIVLTTLVFWRTSNCSISAVTESHTYRTVAYCAYNAW

SAFMGVSVPEMPRTPKLRIIFFIFVWYCFAMNVIFQAFFTSFLVAPGYEKAIESIDELSQ

SNLMYGRDEHYEEALFYISVDDHKKIKLKTLTCSNYSSCLKRLFTKGDISTMSTRYDAEF

GYPYRIEAVGGKQSLCTTREVMVTLHSVMFARFGFPLRDRFNAIIRRCIESGLVEKYWSE

LKFEHQFQGDLKFEENNCEFCSNMYFVFSLSHLKAAFILLGFGYVLSSVVFLVELFLR

>ZnevIR187

MHQRLLTFIVLQMATLIRNELLTSEQRYIALCLRSVIRRNFTPGQSLIVSFPKSGCQGSQ

TSPKCDSSQLVNFVLENTHQDVLWPLQVFQTEFLNLEMSDEQLRIHHNYVIFTWPEEFDV

IGTLVTQLEKLQSVVSWNPRAKFFIVVTENYISPANILALNICETMWNINRIVNVVVMVP

DPDYPRLGARIHILNLYTWFPYEAKSCAKLTNVVLMDQCLPDGDGQLSNNALLFPNKIPN

NLQGCPIRVSTSELIPYVISTNTYVDSDGSTVYNYRGLEIEYLLLLTEAANLTVVYLPLA

EGDVKDTHLQQLVEVSMGISDVAIGHFPLNSILIPFADPTITIVFDDLRWYVPCPKPVPR

VEKVMGLYTLPVWFCIALVFILTALTFWLSANAPNITLVTESSTYRDMLQCICVVWCVSL

GTPVPKMPRSPKLRALFTLLLCYCFVMGIVAQAFFVSFLINPGYHNDINNFDELVESGLV

YGKEDSLEFFLRLADYHEQEMFRSHVDCSDRHKCLERLFVEGDITMLSPIIDVVYVLSHM

GMAKIKKVLCTLNDNVFPLDISIYLTKGHPLLDLFNVAIRRCTESGLVVKYWSDLIFYTN

LQNVDRFKERGCEVCSDMYFVFTLSHLKVSFFVLVFGLVVGFIVFVAELVYRCRSESHRL

STSKYGH

>ZnevIR188

MLRFLCWFLMLKFVVSSRIKLMTTEEIHISSCLSAIVQQYFTHDQPLLVCLPESESVHPV

WMDVMLGQLNQVTLWPVYVSQGESEDGIVATLTTKPHSYIIFTGPQGEFGNVTETLVGQL

DALQSSLSWNPRGRFVVLVTARDSRPSHLLALKISETLWNKAMIVNDVIMIPNSDALLIK

DSANENVIKLDVYTWFPYQTGRCAEPLEVVLVGQCIEGNVNLSHSVSVFANRIPNNLQSC

PLRVTARDYEPYVVLTSNDSGTDGSTTYKYRGINTEHVLLFSEVTNMTVQFLPPGEGTLL

DAHVLQLADVVERSADVAAGRYFQSTGLSAFADPTIPFITDNVRWYVPCPRPAAKMEKVM

GVFSFSSWLCMIVVFILISLVFWRSANGPDIPVAMGSRNLKTVLNCVYFVWCVCMSVSVS

EMPRTWKLRTWFLLFVWYCFAMNTVFQAFFITFLVEPGYNKQIKTMEELNESHLTYCLDA

NSKEHHKQISYQVYENFDSLPINLGSLKECLEYYFINGNITLLSPALYARFVASLMGLDQ

NGNELFCTLDQDEYYIHAVLYLSPGHPLLDRFNTLIRRFAESGLVNKYWSQLNFEVYIRN

VAVSRETECEACSDVYLVFSLYHLRVAFVVLGFGCVLSFTVFLGELIYKWYGRQRTVTT

>ZnevIR191

MNIYISCIIIGFLSSVLTVRSNILTLEQLHTVLCVETIAHRYCTPGQPLIVSLPATAEES

TSRQLIQTTPHKDDLQLVNFMLQKLNEGTRWPIELFRPNEDEIADAAVLHHSYILFVWLE

EEASLNKSLENQVEYLKYSASWNPRGRFVVVMTGHSGQQLPQIVATHVCAILWQMSKIVN

VVVLIPGQEADPPLTTTGNKHRRGTNLLNLYTWFPYHMGSCGEVGDAVLQVEWVCEHNGR

FSRDVNLYPERVPKDLMGCPIRVASVGIDPFVILKDNFTQNDGSVEYKLTGLCVEMLRVV

CKKMNLTTIFLSPSISLDSELYLKQAANLEDGTADVLTGIVPLLPLAMSSFFDPTIPYTH

TEFKMLVPCPKPIPGMEKIMTTFSLSVWLTMGLVFVLTTSVFWFVANGPHVSTFKELEIY

KSISLCFYNAWAVFMGVSVTQLPTTSKLRAFFFIYVSYCFAMSTVFQAFFVSYLVEPEAG

KEIKTFDELLKSDIAYGYHSAWNYILHGIVYPEFSKFHERKETRKDCNDFQKCVQKMITQ

HDRATMVGIVYSTYIASELGIADPSTVICSFDENLIYVSIIMLLKKGSPFLDMFNVLIRQ

YLEAGLQERHWSQLKHEASLRGRDRLKKESNMFVIFSVSHLTPAFVVLISGNILSFGVFI

IELIHNWYCK

>ZnevIR192

MVLFKSYSNMPCYEFQMASLPVKKFLHLVLIGMLIVIGPARGRVLLTEEQNHMALCVEAI

AQKYFSDGRSLLFSVPRDVREVTGRPRVQLSYGDDLDLVNSVLQKINENMSCPVQFPSDA

EVTMTAETNYNYIIFIWREQEHENVIDCLRRQLDTLSDSEIQQWNPRGRFVVVVTDHNSG

TPRSLAHEIYETMWEEHSIFDNVVIISNSERNHLQKQMRNLSSEGAIFDLFSGFPYQRGN

CGKVNEVTLMDQCIVQSNVTFPSRANLFPPKIPKDFQNCVIRVATIGIEPFVILNKNHTR

KDGTTEYDVRGFSVEFFLLSARKMNMTVVFLPPVLEITFQGGWTVMENLVGGSAEVIVGL

VPMLPIIITPDSEPSIPYIHGTIKWLVPCPQAMPRVEKIITMFDASVWLAITLCFVLTSV

VFWCLASISDSLAMKESNTLQTITSCMYSTWAIFVGISVTEMPKTWKTRVFFVFYVCYCF

AINTVFQAFFVSYLVEPGYEDRIETFDELVESNVSYGFNSAMETGILTTDYTEQIKLFAS

RRIDCSDMENCIKRILQDHDIATIAVPTYAKYVANKFGTEGKTDAPCTLDENLIDGSIVV

LLRRGSPILNQLNKFIRLSQEGGLVDRYWAELNSDALLKRIPESDKDGSTGYFVFSVSHT

GPAFALLLCGHALSLLVCLAECVYRRIKQRWSKENTGRRRSSNLQ

>ZnevIR193

FPLKIPKNFMGCPITVTSVGFPPFLIMTANYTDKDGNTRYNMSGFVVEFYRISAEKMNLT

INFLRPSFGSSIELYITEVTTMMERLSDITIGVIPLAPFILLPGLIYTTPFVTVDFTICF

PCPARVHRMDKIISTFTIPVWLTLILVLILTGAVFWSTEHFARRSVHTTTSLSLSFYNAW

AILMGVSVTRNPDTWKQRIFFLLYVFYCFAIITVFQAFFVSYLVEPGHQKRLRTIDDLLE

TDLVFGTNTAVELSVLAVGYQEYYRFPESRRVDCDDMEKCIKRLVSRRDLTTANAGLFVK

YIVNTFGIKDESKVVCYIDESIFHVSGTRRKSVSGGDKHRNPTMSRGRPRRQILVRII

>ZnevIR194

MDKAGKILSTFIFLSVLYAAYSDYLTTEQIGTLLCVDEIASHHFTQGQPLVISMPSVSPE

FSYRTLNHRYSAGIEFKLVDQLLKNTHSRTRWPLLTSQPNIEILDTKMTYKHESYIILLW

ADDKEENILRTLQDQLDHMSGYADSWNLRGRFLVVVTQFGTQSPKTIAMNITEILWQYKI

SNSLIMTPSELQFNPATNKSEYIFDLYTWFPYESGQCGTLNDIVLLERCALGKDRNTSLY

PSKIPENMHGCPIRVFTFASKPNVILTVKHDKEDNSSIYEYSGVEIEYLKLLAEAINMTV

KYLPPLDDKFHLSDFFFHGLISVVNDEADVTLGNFPLHHRATAYCEPTTPYIYASFRWYV

PCAKPIRRIDNIINVFTVPVWSTLVLTLVLSSLLFWGMSSGPSRSVTKESTTYKAVSQCF

SNAWAVLMGVSVAKMPRTFRLRAFFFTFVCYCFAMNTVFQVYFTSFLIEPVYEKQVETFD

ELQQSEVIYLAHPSLDEIGVLINYDEHKLLPRRENCEPYEKCMLRVLQGQDVTTMVMDFY

AEYFASTAGQRKIGKKPLCSIDKAVFTMGLTMYMSKGNILLQRFNVLIQRCLEAGLGDKY

WSELKWNATIRKSDERVDDDDDVSSGNLMYFVFTVFHLRVAFCLLAFGNTLSFIVFLAEL

LSKLFFPRVGFCKA

>ZnevIR195

MSWITTLLLTFNMLPAEHATLHEHVALCVLSVAQRHFARGKPVVMSTPSSVRDAREHKLL

PRSNEIQLANEMLQNLHEESEWPLLSCQLDKPISDSDNAHKHQSYIITLWPDGVDGVIDS

LQQQIYNLLSYENSFNHRARFLLVVMDYGVKVPELDALKMFEMLWNPYKIINVLILLPDI

NTAHKTSSNRAYDTLNLYTCFPYDSEQCGEARKVDLLDQCLLDVDGRLHSNANLFPVKVP

RNLHGCPLVVAPIERIPFVRQTTNHTDSEGDVTYVFEGLEVEYVLNLAEAMNFTPVFLQR

RVGDFVQVRLEIFMELSQGFVDITVGTHPLHLLLARAADPVRPYYELTMRWWVPCATPAP

RMEKIMAVFTPSVWVSILAVFILTAVVFWRTAVGPRSVSRTDSKIFKTFHYTFYNVWAIF

LGVSVPKQPKTIRLRGIFLIYAWYSFAMSTVLQVFLISFLVNPGYNKRVDNFEELVNSDL

KLATDTKMMSFANSSGYWEYLRVGLSTDSCSDIDECLIQLVRYKNITTVSSGFQFEYVLA

KIGRTKDKQRFLCTIPEIVVTSRFSMYVSKGNPLLDRFNLWIQRVTESGLVKKYWSEFIW

NVTVEGLRAPGWDHSDDTESDDSMFFVFTLSHLSVAFCQLLICYLFSFAVFVAEcRL

>ZnevIR196

MLTGILLQLMCVTHNNLLTIAQHHTVACLDNIVHQHFSPQRQVVVSMPGTEPNLAQRTLL

PVSLQYGFEMVDQVLNYINEKTRFPLLTSQSKFTDILPDTGGMFDDDLYPKVHSYVIFLW

TEGAEGNLLASLMHQLNTLRANKYSNNRYRQDMVIVTNQNSQSLAKDLLEFLWKTRRIVS

AVILVQRGNELEDPVLDLYTWFPYESGHCTEGKFTLLDQWAEGKFSNNAHLFSRKIPKDL

MGCSIRVVTLHYPPFVILTTNYTDVDGSTAYKFRGLEIEYLLLITAAMNLKVEFLRPMES

NLEKMSELEESRADVVIGSFSMRFYHLIDGDPTRPYVYTSMKCYTPCPRPIPRMESITRV

FMPSVWIAAVLVLALTAAVFWITTIIIFDAKSADFTSYKTYHHCFYMVWAVFLGVSAPAM

PRNYQLRALLFIFIWFCFAINTVFQAFFISFLIEPGYEKQIQTPEELREAGIPDLVLDLF

MKEFATDNEKAYCRDDECLINLIEKHDIALKTSKHHMQYIASTIGITKDYDKYLCSLYDI

ISPVMYGMYLTKGSPLLDRFNILIQRCLEGGLGEKYWSNLRWSAILESEAQFMEHGTTDD

DMYFVFELSHLTVAFYTLLLGCTCSFTVFLVEIIFDCMHL

>ZnevIR197

MRNALKLLLSYWALLGTVTALEEFLTSESQQLLSCVETLLNRQLPPGHSLVVSLPTDERI

SNHRTLAPRDHHAIYFELIETMLKNVNTKTIWPVFISRPTTPVSENLIPHKHHMYIIFLC

PNADENIGVTLESHVRNLTEFLESFNRRGKFVVVASGYEEFSPTYYAKEIIDIMWKGNKL

TNVLVIIPNKHTSAPKSNLLSGNDTEQSPGFYVYTWSAYESGQCGEVEEVFLIDEWVVNE

TKGRFRKNMTHFPSKFPQNVHGCPITVSTVHFPPSVIMTENNTDVNNIKYRGIEIEYLHL

LSKDMNMTLEFRTPPEGKLEDRFYKAFSEAVLEGVSDIAIGCLWIHPVMIYYGEPTIPYM

FSRIKWFVPCPQPTPRVQKIKEMFAPSVFILMALVLILTSVLFWSTARVPNCVSWCDCLQ

SAWAIFLSMSVPKLPGSSLIRAVFLIYVWYSFLINSVFISFFISFLANPGHEAQIKTMKE

LAESRLTVYIPDLMNKIFVFTSYETYFKLKLPIKTAETFHESFRRLIDENDVATAGLQML

AEYFAASEGKFGKYGHLCSIDETISFARVTMYFAKGNPLLNRANVVLRRCFESGLVKKYS

SELMLNTRLKSSAKLLECENMEICKGGHTYFVFTLDHMKYSFIILLVGYSLSFVVFFAEL

ICKYYSKSVVNT

>ZnevIR198

MYISFIGEGTMKFLVQKHQDKAIKEVVMIILTFFNIVCSTRANHVFTSAQRHILNCVSTI

IHQHFTTDHTILLSVPSAEVVVAQPSIEISSFEADNEDLVELSLRSINEKCLWSLQISRP

GVKMFENDLQVSDGRHNYIIYTWTDEEIGDVLTNVISEVEEMKTKALLNPHERFLVVILG

TGNQSPSQAALSVLKELWSNYMILDVLILVCDITCQKFDSYEFPVTSTGGNKLLLYTWFP

YHQSGEEVILLNTWDMKKHGNLFRDLDLFPNKIPNKINSRPLRVTTLDLEPFVELEETYT

NENNEKMYKFKGPEIELLYVIMEHLNISLYYLPPPSNDMKYTDQLIVSIERVALGEADIA

IGSLPLLLEAVSYVDFTTSYFITGDRWYVPCPRPLPRLKRIAGIFHETTWSMMVIVYLLV

VIVTWGSAKLVSRKESYRYRSISRCFLNICSVFLGVPVPVLPRSSLTRSCFFSFVCYSLA

MSTVFQTFLTSILVDPGLSDQIRSFDEILHSGIEFGYVKDSHLYYFPSGSELYGDEEVEK

HGQNCSDYKECLSRVIIKGDYATLRSEIYADYFVAKTMPKRVKPLCSLDSRFKNYLVNIY

VQKDNPLLNSFNKIISQALESGFADKAISDFNVVWRYGTPNNATGFDIEDTEDMYFVFAI

EHLTPAFYILIFGYASSFVIFVGELVYWLGVGKLQNIILYHRKG

>ZnevIR199

MLILLLISNMLNTLISFVFRRRKITFLLGFASLLITTGLTFNELTAHNDVMILSATDFII

RHFTHGRPVIIILPGSTGNIKRNSPDKIFIQEYEFKMSDILLKNVNEQTYWPLYVFCCYD

TTGTMPDNDKLYHSYIIFIRSETGGDASSNIIRQLKYLKENYLLNPRGRFLVVVNSPTSD

TPSDVAFRVLNAAWKYLIVDVILMVVSVPLNINNSNHLKSNELIAPVIDLFTFLPYSKEQ

NCSNVKSITHLYGLFPNYTEKSIPKKINLLPNRNIKNLYRCPVKVSTYHNPPTIVDTSSG

GFANCTGLEVNVLVFILQSLNATVAFKIIPPTNGSFFGTYANVLSDLDSGSADIVIGALP

LHEILYVNYDPTVPYFYTPVQWVVPCPKPSRRWGTIFKVFPLSVWLCIFICFVVVIIVMW

LVASSTEHFNYSSVQYCLLSAWAVVLGTSAPKKPQTPSVRLIFLSWIWVSFALCVIFQAF

FTTFLVNPGFDRKIKTMDDLLRSGIKYGYTEDYGLVADYTVKTSNLKKCLDPNVCLEYAI

KYGNYATVSNTFHTDYYRAKLSWHDSHLRVCTMDDDIIHVNVVMYLTKGHPLLQQINEAT

KVMVESGLMEKWKNDFMYTSRLHSLLANGDNYEIPEGDWNSDYFVFSLTHLRSAFMVLVL

GNILSVSALFIEVIYCKIHFRNPVRKPVRYKRISHFKRRLENSRYRFSRYR

>ZnevIR200

MGATLLIPNIARNMHVTTIIIFLECLGILQASFSTHDDALMTSKQRHILICLNEILQLHL

TPRQTLLISLPSLPNNATVRKVTHKYPFGEDFDFHTVDAFLRKTSEDTGRSVQVSRPGAP

EPETQHEDYFKFDSCVVFTGFEEKQDDEVSDSIIEQLNMLERRKTWNFKARFIVVASVAE

NVSLQRLGNRIFKVMWKKYSIMNVLLIITAINYKTDANLAPKSNNSQVELQLYTWLPYTS

HAYCEDLKVALIDKWISKGEFVLKAQLFPEKVPKTFHGCKSKVTTFIYPPAVTKTPDLKY

TGLELKYLTIVFKKLNLTVEYNEIPYIFKSHYQQFYYAISKLQPASLDMSVGILPFGGIN

VSAESTRSYFDFKLKWYVPCPEHASRWRSFYQFFSLNVWMCFCLFIVLAVITMWLLARYS

TKFSIRESTNYMTIMNCAYNVWAITIGISAHKMPVSSSLRMFFVVWVWYSLALTTVYQAY

FVGFIVNPGFEKSLSTLNDILESGIEYGFTNDMDNIQFSDPTYDIIKKNRTTCLSIFKCL

ERVIRHKNFSTIADDFHVQYVSTRLLFYNIHIPICSLPSDIMRYSISTYMAKGNPLLYKF

NEILKYLIEAGLFDKWKRDFLSNTRLAGQRIDNDDTNFEEIFQNNFFGDNLSYSLVHLQV

VFFILLFGHAFGILVLVGEILHYRMYGSMETASS

>ZnevIR201

MKGVSFITFWLIQTALSSGDNDFLTTEHHHLVMNVMKTVEQNFRPGASVHVSLPRNDRNT

LKTHRNLINSPLRGTSLDVANTLLAKLNEKDRWIFSVSCIDINDENVLKQEAYLILFSLD

QILNDDISNTMYRLTGTSMWNSKAQFLLVIIDHISSSSEKIAVDVLKELWEGFKVFNSVI

MMPTVDKSLPPEDTEYVPVIDIYSWIPRQSLEMCMELKDVILVDRWYWKFNKNLKNITDL

YANKLPLEFGRCPLIVGTALRNYFWVTNDMNESILEYYEPEINLLHFVFKELNLTLIYDT

PEPVNSDYVRSITNVMVRLVGGGIDLAIGEIPLMYNITKHADNTISHQNYHGVWYVPCER

RESRVTTISRIFSVEVWIMIIMFILTASVLMACIGKYLKSNKVHESHIFLDVKTCLVTLW

AVTMGVSASELPRTSNLRIFFLIFVWYSLAISTVFQTYFTSFLVEPGLTDRIRDFDGLLK

SGIEFGYDPIFDVLVENSSDSRYEEVLKRRILCRDRDYCFGRVSKADFAYLDVEAVKMIY

KISHKNALICHIDEGVINIVLTMYMRKGDVLIEKINHILSIIGQSGLNIRILRGFFYDIV

FAGRKNKWFKKTDNDSLVPPDEDESLIQEDYVPLSLTHLYIAFYVAIIGYILSFIVFLGE

ILVYKIKLSFGV

>ZnevIR202

MLFIIHFIAVGNMKTVFTLAVFALVETLSRERTHQRHLALCITTIARRHFDLDRTLLVSS

SGSGDGLLDIVMENLQNIELWPLRVSRTDSVTLAPYEKHHDKIGSYVILTGGPEDIEKQV

DKITDSVLWGDEARFLVVVTKCEAIPHLLALNLLRELWDNTKALNVLVAVYENSVLHLYT

SFPYQSDKSCEIITDAVLLSKWDTQSDTKLGNNGTLFPDKYPRHFHGCRIDISIPHSKIA

EKTYISETAKRLNITVNYISGRPNDMSLYDRIRSSISEVLLGSSEITVGGIPLIKDIADI

LDPSFSYHETKYTWYVPCARPLPRLYSISRIFSASVWAVVVVVIFAVSVIIWCLANRSLE

TSTYTSISNDLYNVWAIAMGVCVTEMPRTCPLRLIIFSWMCYCFAINAVFQTFFTSYLVD

PGYDKQIRTLEELIDSGLKFGFRPEFDVYYQNSNYEIHKELMSRKEHCDPPSFCVQRIID

TRGYATLAESYSIENYLNTVNNSNYVCTMNDLEAYPVKIVAYFSKGSIFLRAFNKALVSS

VEAGLATKAVKKKVSSTVSNAVDNFFVFTVSHLSIAFYLLLLGHSVSIVSFFFEVIYKKR

AES

>ZnevIR203

MLVPAMLALLQCATNECEQNQLVSSVAGIAQRNFNPEQTLLISSTGEDHVDLLLGCIGAM

SKWTVRVVRPDIAPEETNHEDQNTIGSYVILARDLEALEEQRQELADRMAWRNQARFLVV

VTGRVDDPAMLALNIVQEMWNSARVLQVVVLVQNQLYTWFPYQQSSTSKEIVRLDEHSTL

FPDKTLSSRHGCELTVGTTPVKAHVMESGNNSFWGLEIEYLRLLQRALNFTVTYKTPAPG

SSHDRHIKLVTDLHVGLVDVILGDFPLHQFIAQYADPTIPYLDSNLKWFVPCARPASRTQ

SVLRLFSTSVWTALGGVMLLATLCTWLLGRRVRHPPSLWSVLHDVWALALGVSVPQLPRD

SRLRALFAFLVWYFLAISTVFQTLFTSVLVDPGLNKQISTYEELQHSGLKYCTHPDIELL

IKASGLYLEDIKLQKDKTLNHTECIRRVLLGDDMATVTFSYLTEYFALAELGTRSKICSL

DEDIFQLRYTMYVTKGSSLLDKFNMVISHMLEAGVMQRLWLDVKESVRKIGLREASEEET

YFILSLSHLQLVFYMLVIGNAASFVVFLVECLIRS

>ZnevIR204

MLKLLLLLMMLQPARFVPLSSMLTPRQQHVILCARAIMTRHFIPGRVTLVSAPNIPHDVH

IRHRSLSLTPSHWCDHYLMDVMLATLNTEQMWPLTLFRPQDSITRPSFSNNFGKHLWIEQ

HIIIVWVVKDKTDEHFRLIYDTKYTINTVMHTSRFVVIVFHEGAENIKVCRVMFSEIVST

HMYNVQLVIPNSGEEIDGFEAIDLFTWFPYQPEKQCGKFKQVTKLDQWIEEDEGRFMNGV

DLYPNKAPKNFQRCPIKLSNYQFKNRISRLLEPVEGKVVKFIFSSLNLRISVARVPDIIF

GGVTLNFFGKINKNSITFPHLFTTLKWYVPCAKHVPRQGNFTKVFSWSLWLATASVSLLA

VAAIFWIYKSYREPRSVAECFLEVSAVVLGVSVSQAPRACGLRAFFLIWVCYSLAISTVF

QGFFTSYLVDPGLQDQISTLEEMLQSGIKYGIPPTEELFWCNMTEFNRRTCDQVTISQKN

KLLYYNDFIGRDDTSLLAHDVEMDVVLSCLKKPRACSFSDGCIEVMFAIYFPNGIKSILY

EPFNTVVIRMFEAGITEKLKDDSFMELRSSLHNSYFAKTLKEEMRRTNFSFKNSTYSDDI

DGDGYFVFSVSHLQMAFYILAFGKVVSFVIFIAEIIYNKV

>ZnevIR206

MKLVTLLLFVAQVMAQANLPTAKHRQLLLCTVSVIRRYIAEGHPLVLSAPDEVFPWDEMN

TLDETRNLTSELLEDLHKVERWPVYILYERTTVSFEEIYASRKYKSYVIITQNIRSDINS

LIKCISSQVRRLQEIEAGNPEGVYLVVLLNAGEVDREMIIKVFSILWEWKVLDVIVITES

LSRQSGITDLKTKVDLFSWFHFQYPRHCTHVTDIVLMDQWENGAFVKNADLFPKKITRNL

NQCPLVASTISWGDLVIASNESDSGSTEPRVAYKDGLEVILYHTIAEAFNMTTAFIDPIP

SHDNIWGDFSSADNTYTGVIGDAKFGRSNVSFCGLAKNFFFEMHVDSTHSYLESGFNWYV

QCARPRPRWRVFVRTFTLSVWAALIVSFFAVVIVVWRIAKFPDERQETAQYRSFVGTFQA

VLAVLLNIGVSRMPLTLFLRGFFCVWIWSCFALTTVFQTYFTSFLVDPGLEHQVSDVEEL

LASDLVLTFDNGYLDLFKYEEEREKQIISRWISCPGFEACSRRTATIGDAATVLDFQEFD

YYKHKFVDENGDSLLCRLPGKISGYLITMFMQKGNPLFRPVNDVILRIMEAGLVNFWWSL

IVERQKFTRNPETEDTPITFFVFSMSHLYVAFTFLVVGCGLSFLVFILELLCNFVHITKS

RNIYPAEVTAY

>ZnevIR207

MKFAMAVFLRWKWERLLIVLVFIVTEVLKTNQAAVSSWTAGQEFRRPRDVLECVTKICTR

HFAPNRTIVLSSSSTVRQFPGFVSSLQMNDDSKYSADFETRLLEDLHIVGRWSIVLSQPR

KNTNKSALEKYSNYIVVARSQDGKLLGFKEQIFHLESSEGWNTLAKFIVVLETHGRLVSV

RETAVIALQELWQRKVMNAVVLVEQNETVNIYTWFPYPHPRDEFLDVCVTQTDRDWFPRK

RNLFPGKIPRDMQGYMFRVSAPNLPPFVMIEGGNRTSNLYEVDGLEVRLLRCVSQKMNLS

NIVIHYDGDFPYGKEIENGTWDGIIGDLVSNRSDVAIGGWFGYVIREEPLDSSVHYFTDQ

FPIFVPLAEKFPSWLSFSRVFGKFTWLCLLISIPLTGILFRYVATQQADESRRYGRFLNC

VSYAWCVVLGVSVVEMPRSSPLRVLFIAWLIYSLAINTLFQTYVTVFIMNPGVEHQIDSY

EELGESELEFAFEEFYNSFLSEETLKTLKPRYVCEGPHSCFLYAISNHGKALLTSRVFAL

SEADTLGYARNFPLHSFTEDMFQLYIVMVFREGYPLLESFNTIITRLVEAGLSNKFLEDV

MDMLRSKNDSLAFDTSFDYTPMSASHLHSPFVLLFLGFASSFVIFLGELIIHRARLLTIA

SNA

>ZnevIR209

MTKARILHVLIMTQWFFSGISALSYKADDTNDFEKHLLKGIVNIGIAYLKNYSPLAIVTA

DTWCNKTLHSKLGEAVLRELQDTGNLPVVVLGNDRTARPTRKSTTKPGSVILLLNDGDSG

NEDLVLHSMLQTLSTSPSWNPRARFIIVSTAIPSSTLGQLKIVRSALNHSFLINILDAIF

MTPAPGFSETSSGPPAIEILTCFPYGSNRNCSDNVDVTLLDRWEEEEVEEGIRDARFRNN

VNLFPSKSITNLRGCKVAMAYLQWAPMVMSSDGNTSTEFFDEGLEVRIMKTVAEKTNFTL

ELRIAENNIKVHGYFAAQWLKPELLGAQDATRPHFTGAFTWFVPGERKIPRWKSLIRIFN

PTFWLLVLLLYVLGSVTFWALARIPTNGEDAAAFSDVILIFMHTLSMILSESVYKKPKHT

KCQMYFLLWAFYCLQINIAYQSSLIGFLANPGKEPRINNLDDLLESGIELGIQSGLETFF

NDTSDPRNKRILKSYIRCDSKNIDVCLKRMAYKRNLAVAGGRIGIEFSAYTKYLKNGKPL

YVPFKDNIQQGHMVMYLTKNSVLRQRIDSIVLRLQHSGLVEKWVHDLRRKFGKHFNKAPR

KNGFCVLSLEHLEGAFYLLFLGLLTGLMTCFLEIIYNIWVTHLKNRLTTRN

>ZnevIR210

MDLIALLFPVAAMGVQHKILACAVNSFRHLQPGPVVVVTPRDSDSLASELLQELHAIGQW

AVFTSSVDSFAPQPSERPSNFVLPPVPSQAELRRILTELASWNPRARFVILMLDETMLHL

IINSLSDLNIVNAVLLVLTSNTSSVKAYTWFPYLPPGQCGNQGFVPVLLDECLMENTSKF

VRNVPLFPHKVPPDLYGCPVTLSTFPWPPFIIKSHVYDTAGKVFYTKGLEIRLLNTIAQT

MNSTFQYLPPPANDSKWGSRTPSGVWTGLLGEVFYKRSDIAFASMTATDDRILYFDTTVT

YWSNSVVWIVPRPKFISGWRSLLGIFKPTMWGIVIVTYLLGSVSLCSLSXXXXXXXXXAL

YRNPGSCLMTTWALSLEMGGHMQPRGMVMRLMFACWVIYCLQISTAYKSSLISVLTNPQL

EPAILNMEQLAKSGLRLEYTVGLSEYFDDSVDVSMRRIRESLRFCANVTVCLNEVAFAAD

TALVSDKWYVEYLIPHLYLDSSGQPLLQIMPQDVLSYHVVMIVSKGNILLDRFNALISRV

VESGLMVKWANDIKHTRTIGKVSQDGVRGRRLSLFHLQSLFVFLLLGECLAIVTIIIEIL

ISKAF

>ZnevIR211

MANCVLAHNTMRQQGHVVTCITTVAQNFFAQEGTTVVSLPNEEHLTYSHRQDTDLLLEQF

HGSVGKPIVVSGPGQEMCPRLPKIISGPLISAVRTPRQCNDFDKQDSYILWASGQVREQL

ERLKSCYNAWNARGQFLVVLETPGDLLQILEELRQWKILNVVVLTPSDSEQHTFDLYSWF

PYRKPSSDCGKLRQAVLVDKCVANREYLLRNAALFSSEVPTDLGGCPISVSTFPLDPFVM

LGSDKEEEMDETEVRYTEGLDIRLLHFMAKCLNASVKFVPPPIGVRNDANNTWGGVAGDV

IGRRADIGMGATSYIYSIVRDLDFTVPYDVLEVVWAVPRAKARPRWGSITRVFHLPMWLL

LMGVMVVAAGAMLCLSKYKAKYQQGRSEYTTLSGCLSSAWAAMLGVGVPVQPRSGPLRII

FIAWVIHSLAVSTVFQAFMTSFLVDPGLEKQIMNVEDLLTSGFHYGVHKIFFGYLFFLQI

KQRDVMLPNTDICVKVEECAQKVADEGNYATILNSITAEYLNTYKTSDNNGVGKLYMLEE

KLSTNFQVFILPRGSEFLGTFNRLVTVANEAGLVTHFWKEMLTTNKIKARSITEPVGDGY

EVYTIIHLQSAFYLLLLGYCTAFCVFFAEIIHQKWRTRRRRQNKIRFLERRVRRCLRRRR

VAW

>ZnevIR212

METWRAISWLLTATCFHAHTRQQSHLATCINTIAENFFNPQGTTVVSLPTEVQLTDSDSN

STVFEDTLRTQDTNVLNLFLKQLHSSLRKTIVVSGHDHEMCKRLPNIISGPSLDLADWDW

GTEIPPNPCKVYDKQDSYILWATGELNQQLERLKSYYNAWNARGRFLVVLERTGELPMIL

EELRQWKILNVVVLTPSSSEQDAFELYSWFPYQKPSGECGKLREAVLVDKCIAKREYLLR

NASLFSAEFPNDLGGCPITVSTFPLDPHVMIGSNKDEGTDENGVRYTEGLDIRLINFMAK

SLNASVRFLPPPPGDWSDFDERNYSWRGIVGDLIFRRADIGMCGTSYAFSFIRDLDLTVS

YDVLEAVWVVPRAKVRPRWSSITRVFRLPMWLLLMAVMIVASVTMICLSKYQANYEEERS

EYTTLSDCLSSAWAAMMGVAVARQPRTGPMRFFFIAWVIYSLAVSTVFQTFVSSFLVDPG

LEKQIANVEDILSSGIRYGLHKTFQSFLYDLPGNQRDVMMNHTETCVKVEECALRVADQG

NYATILASITAEYLNTYKTLDSTGVGKLYMLDEAKLWTNFQVFILPRGSEFLGTFNRLLS

VAKESGLVTHFWKEMLTTATIKGGSIRTQPAVDDYAVFTLTYLQSAFYLLVLGHCIALCM

FLAELMHRKWRTIRRHKMADRKRGHGLRRPRIARR

>ZnevIR213

MKSYWLLNQVCMYVRLCINSSVHYGRKASVSLLQTHLCLLALTELYADNVDLYEDEDVLL

ECTTETSKRYFPSADSLLFSKSITNLTSNPSFPTVTYNWIKRKFYQKIGRNGCIIDKEVE

YLHFDTQNREQQIRIMSHILLELQNFSKALYNMENVLDFENRILKNLNRICRWSIVISPY

KDSLSDVTSELFGGFILVTRFENEDSFAVLRDQSYQVDVLDRSGLLNRLVPTLVVVTGDA

GTRDLVYDVLHYFSDNQILNVTLLIRDNVSEAVNLFTWSPYQSPSGQCGRLKEVVLFDSW

IPSPNGGRFLQNSKFHSNTQININGCYIEIFGYEQPPFLIFEDNEKGEDISMKGLFVEML

RYVAKQMNMSLISDSDESKEFYVEADVHVRRVLPFFQYKTATFYTLTYAWYVPLAETYPR

WASMSRVFSTTTWLIGFLLILVASFVLRLTAAISSVEEPPKYQSVVQCLTNAWSALLCIG

VGEMPRSLSLRSVFLSWIIYCLCVNTVFQMYITSYLVDPGFQHQVDSFEELEESQYELLF

NTYDTLLLYLEKRRMPIKLLRRSTDSMLFLMQTPTVAMFYDADLLNYDYGRLCGGTATYV

FYKFREEQLQIHVSMVLSNNYILNRINEIIHHLVQAGIPDKIMRDLTDPMGQRKVTRMVV

DLAEEYSSLSLSHFVSPFALLLLGHELSLSVFMCESAYHWIQDRRAK

>ZnevIR215

MRHICYVVLLLGYPLFVHVTIINWFDSNYLHDKEATEDFLQDPTFWSVSKIHFRSSKILT

VTVTSLNNRVWNDTVADLIQEMYKHLNIPIILTYFKAFETYPGRSLHPISLDYVREYPPE

SYHSYLIISHSEMNTYLYTYDSTSYPQYEWTSRDNYLILTILHSSWHAGGTTDMYGSTFL

NLWTKYQVLNVITIVKYMDICNKQSDIMVYDFFETNGVINVTPEQVAELPRSYLERTWNL

KGYSVRVTMLHSFPNAVFNCRSLKKCSYEGRDWEVLRNLATYMNFTPVISQPRDGKGLGY

KTESVFTGSLGDLVYKRVDISANERYIKDYDADIEFTMPAFYTQQLVVIVPKAQRIPWWR

AMSECFTFHFWVCLLAVFLVTTVVWYVLRRLSEKVSFLTNAVDTMAVFLTMSLSFLTKIS

ALSQRLLIASCLFFSLVVMCLFQSSLLDTVAHPRFQPDIDTLQKLDKTGLPIITLDRNLL

DTFNESLALRNLADRLQHQDVSEDALLHQIANHRNACMLTSKGEALWFLGKFPNKLHIVS

ESPREYFVSYMIPKGSPYATRIHNLLGKMSQAGLVRKWDVDASYRLQLKALREGRANLQD

TGNAKVFAFFDFQFSFLVWAVGVAVGTVVFLLERCFA

>ZnevIR216

MKLVIFPLLTLTVLCTTYSKPTGLLARLHPEQFEEQRRTSTTHHRTLLWTACLYHFRNSR

VLSVIILESVSETFHSYVTHVVSEVNRLLDIPIILTVGHAELRRRYTPSREHSHLLIAPN

SSSLTRYLDSGWKEYPQSTWRPKENFLILISYSETDTTSKHHNIFDRLWSDRGILNVLIV

IKSLTKSDVEYILAYDPFLMDEVTRKTKMWMVKADELHELPKTYLQRTTNLHGYTLNVSM

FSDVPTAFLQYDNATGKWISRRRDGRVLNIIAKYMNFTPVIIPPKQNEKIGYKLENGTFT

GAMADLISRRTDIAVNEIYLKYYGTDEIEFTMPAIRNQEVVVLVPKSARLHIWMVIYKAL

RRLHWQYVLASFLSCVVVWYLLRRADAGRHLKRRYETSFFTNILEMLAIFMNMPLSFLTK

IKSSTQRLLLSSCLIFSWFIMCNFQGLLLDVVTNPHFDTDIDTLQQLDEAGLFIFTDNPN

LLDTFNGSETMENLSGKLAYRQDSLFVVSRMKKYKNVSLLCSKKKATWFMRHNGNGMLHI

VEEAPRVYFMSYMVPKGSPYLPRLHVLFGRITQAGLVDKWDDATNYEMKLEAEFGDSEEL

ELPQWSLGMSDVAVDFIIWAIGLVACVTVFLVELCLCKNKKTNKLLTL

>ZnevIR217

MIFPLTVSSATLLVLSLIFLSQCSTSAVQYQSLQRLLRNPQPLLVRESDMEILRNVCLWY

FSKSRFVVIIPIDTQVADDFVVELLPFIHNTIKEKIIVSHVVLPEDFKVFPGRLWKNLSI

LHQAFVLIVEREDALMQYLKFIQQISTYPFLRNPRGNFLIIFLGPSENDRDEVNRNYMEN

KYSHILKALWNELGIINVITMVIKYSNYRTLEAGHIVQLATFNPFIKSLNSSGKLVVYNK

YNVFNLVRSYTDTLSNLRGYPIRISLFTRPPSSFPAHSGDKVIDYIGVDGFFMRNLAKQM

NFTAISGSPKDGEEYGYMFETNGTFTGSLGDILYDRADISLNSRFVKEYGTKDIEYTFPV

GFDNLCIVVPKAERLPKWLAFFRVFPLHVYLALVCVYVGSCIISHILQKLYSFFMPVNKD

TSLISTFIEMLPLFLSLSFVRIPLSRSQKVFVITCLVFGIITTSLFQGKLVTVLSKPDYY

PDVNTLEELDASGLVIATGSINLIENTFSAEESPLVKHLSNKVTYLSTKEGVADYVAKHK

DTAALTRLSNAIYNVLIYRNSDGTLLLHIVREFPRTYSLAYIVPKGSPFLFRINHIITQL

VESGIVDKWSKDTYFNMTAFQRSENSGSDSLKVFSMEDLLVAFLILACGLLSSVIAFFVE

LTVQRVASRKRIKYKTFTSSHEHTLLQNDYVQPSFRNYKI

>ZnevIR218

MNVVQKVLLTVLLCDFYHFCLPCLGHTHSFRELYVNNRTDEREQNTDDKSFCCLVTCFFT

DAQYITLITIVNLKEPEVVTEISTELIALFHHRVQMPVMTFYGTTDATIHAVNVLSDSRR

DVLFITENIASVGQYLDEDRDNKTRWHATDNLFIGLVTTSNRQLHLSDYEMKYRELFQRI

WTRYNVLNILFLDSRISPRFWHGTGTSIVTFNPFLKNGNVRGMVQAYNITDANTVCQRFE

RKLDDLHGYPLRVTMFGSYPYAPRIYSGPTTSQKYGGVDGNVLNVVTQLMNFTPVLHRPK

DKIKFGFRTKDGTFVGSLGDIVYGKSDIAFNSHYIKNYDDAQIQFITPPIMYDGIVILVP

KSQLIPGWMDLFECFHLTELLFILTFYVISVCFGTFVKRYLGTEINIYEISRIAICILKM

FLTVPIVGTRELTSFSERVFASSCLLFGVLMVTAIQVTLVTEISSPNYYPDIDTLEQLAE

SGLPIATSYQNLLDTFSDTENPTMIRLAKNVRFVSDSFSIKERIAYKMDYAAITSLSNVP

YFLSHYIGPSDNPLLHAVEEKPRGYFLSYVVPKYSPYLRRINILTATLTESGVVGKWEAD

EVLENQIIQRHDHTHIEKSTLHAYSMNDLQTAFFVLVFGLSCGSVVFVAEIVVAFKLFSW

K

>ZnevIR219

MRLDTQGNIIMWALLISCDIISTTKSQSRKNFMYQTKHDITDNNHFNCFVDAKCVSLITA

EMQLNSVDIPTYSIWCIQEQLHIPMVIWKLETNSCRTNSMDIPSGDALLMILHYEFITTL

TVQIKACIQHCSPRTQFMFIILYTQTLEKNDILAIFEMMWQQYNILNVVILTIKIYPNDI

IINISDIKADTWTYDPFISHLYEDLSNSKRLNLQNLNGLPLRVSLFGHYPTSYLLQESNL

ETKVSLFPTNDSQHLNWESYQGVDGQMFSTITHYMNFTPKITTPSNGEVYGFPLPNGTFT

GALGDVIYKHVDISFNSRFIKHYNTADIEFSNPILSDKMCIIVPKAKPIPHWRRMLSSFN

SELWLILFCTFAIVASFKFILRKYHNPKELQWLVILETFQVFLLIGIQNSPKVTSERCFY

ASCLIFCLVVMNAFQGLLVTNITYPTYEADINTLVELDHSNLPIWSRSPENKDVFKDIGT

PVMERLLQKFSVFNGTAGELLNHVANLTDAAVIVRETSSTYTESIYVAQDGTQLIHTVEE

CPAYYHLAYIFPRGSPYLPLINIFILRMNEAGLTFKWHQDGTDVKSLLSYRKKKYRSQEP

LKVFSLTDLQLAFYIFVAGLIFSILVFVLEALIGY

>ZnevIR221

MVTIMKKEVLVLVIFIAITGMTRTLEPKLKFFLEDNISDETRRSLRETLLCKSDIQTTRV

AVATAGRLQDSTSVPFLNILRDISDEQPVILYYHTNSSSTNGENQPSRRPIGRTTMFVLF

VNGNNDKEFSETLRILKQSEHWNARAQFILLLFQTYKNLEDKIGLIFRICWHNQMLNVAL

INMETKFKLITVADLNVYSYNPFNQHSNTTNIIGVSFESFKNSMCRSDRQHFYLNNLRLR

DVKGYPLRVSMFDHRPKSILKVGEDEKIYSIGGEDGILLKVLAKHMNITPIIQTPKDKVV

TSFRRSDGIVTGSTGDIIYDRADVSFNSRFLRFDYINDVDFTYPHDKEGFCIIVPKAERI

PEYLCIFLPFRPVIWLACGVSIIVTATFWYLTELRRKGRSSYINVILNAYAVFLATSLRS

NTSHNYGRIIFITWAFSSMILISTYQSSLITYLMSPQFYDDIDTLQEFDKSGLKLVMFPG

IKETSLVDNLDPLRVSLDKKTILTTQNFSSCIRDVIRHKDRGCAFNKLSADWTVRQAEYF

RNGEPQLHVVRECLSWYAEAYEVHRDSPFLPYFNAIIIRISESGLIRKWRKDVKFKVISK

QRRIYSTSKQKTVMQLKHFEAAFFSLIIGLFISMLVFLWEIKLSVFPHRQRKRCKYGTQF

CF

>ZnevIR222

MKNILFSEWSNTNNGAFLIRTNQMFPFLKFRCFLLNIVWLVIILAVLDVHLCCSEQIDTD

LAQKLLVPNDDKFRNLTQLFDRCFDHNHRLLTVVVQGKSLPDSVLCHIHSRNMSYPVIFM

TNDEFEAENGRFSETLHQSSDTFILFVDKMDETEAALNKIKSFPLWNARGRFMLIVSHPL

RSVRRNVIEETFKRLWEENILQVVLICRFSSSDQCNEDSKNCSALTLNQVVLTYNPFRGI

TYNKDISNIWPRSSNLDIFFPSLSDLHGYPLRVSMFPATLSVHPILGPDGQVESFEGYDG

HTVTSLAQYMNAVLVLLPQKDRAMFGQKFQNGTITGTIGDVAYGRADIASNSQYIKTEGF

ELEYTYPHDTNNLCFIVPKSKRVPPFRNMFLSFSCSVWTVLACAMFLTPQCWYCIRKYGR

ISVKEKTNNITMNDAFFDIFRSFISGTLNTVPTSVLERVFIITWVLVGIIITNAFQGSFT

SYLAVPKYLPEIDSLQQLDESGLGIFVSPLVNSYMTLDINDEIMTNLWRKFKYDRNYGLI

ADRVAWKRDMGAIFNELSFSYYLRSSRYVKDGRPLLHRVKENILSIYSVYSVPRHSPFLP

RFNVIISRLVESGFQIKWISDSLHRAALEGEITPVSSFQAAEPAPLSLTHLQTAFYILAI

GLLSSTLVFMLPFK

# Supplementary Data S4. OBPs

>GjilOBP22a

MCFPGGYRKYIKGYRVALGATVTREVGTIRHSTKDSSRRMCVKITWCIVL

GFAVFFTAEAAKLPETFPLCKKSDPRLNECLKDAIHKSILELKEGNKRLH

LLPLDPLDITKIVINQGNGPVSLNLDLENAKVYGLTALEIRSVSSELDKY

IINARGDVPRIHIEADYTMDGKFLILPLRGKGRCNITFTNVDASMNLQGA

ELKKGSKSHMDLTSYEVIVNKISRLQVNFENLFNGDKALGDNTNQLLNQN

WSEFWEELKPSFQDTFGAVFKAYGNQIFHKVAYNDIFQE

>GjilPBPA1

MSARLALVVIASLLIAVECSQEIMKNLSINFAKPLEDCRKEMDLPDSVLT

DFNNFWKEGYEFTNRQTGCAILCLSSKLELLDPEMKLHHGRAQEFAQKHG

ADEAMAKQLVDMLHSCMQTTPDDANDPCLKTLKVVTCFKTKIHELKWAPS

MDLIVGEVLAEV

>GjilOBP12

MYNLQTVLCASVVGVLISTAEGLKCRSDEDRQSHDHFKDVARLCMSQVST

MGGSSDHDSKSYGDEGYGDENNGGRNTGHQEPRQSSARPRYRRQSQYDYN

DDDDMRGNNNNMRRNNKNQGDGISGLNSAPLEHVEPCVVHCIFRQMHMLD

KESMPDRQSVVTVMSHNIRDNKVKDFVEDSVDECFESIDYDSKKGKCEFT

KDLALCLEEKGRSTCDDWDEEADDNKTRNKRPNKNPRRDDIFEGEDSM

>GjilOBP83g

MFGFGFLSLAAVLLCLGSTSALTPEEESSLKEVLHPFVLECADEYGIPAE

KFEEAKAKGSADDIDPCFISCFLKKAEFFDSDGKLDVEKTNAFVKAHLTS

EHVIQFFEAVGGECAKVNDEEVTDGDKGCDRAKLLFDCIQELKSKIGD

>GjilOBP57c

MKYAFVIFFCIIAVASLTKAEVSSGEESGEDSYIQELMGEIMGAMFGCKG

ETNITVCKEMWFEINDTSTHNKPEYNQCKCLGSCVCKELKTMTPEGVLDN

NAIQARIANITNVEMKAEATRINDACFPKADGKIDCEASFAYSLCVIEVS

SEARQNIEGLVSMF

>GjilOBP14a

MRRSVVTLLLLAAAAANLISVLAEQEDFEDEAECFERHPIQEKARTFLTR

FWDTGHVPDEESDNQKCFIVCITKRILRDENGDLKMDLLTDEFGQHLQNI

KDQGIVKPAVVQQLKSDLVECSSCQGEGECTTEYKQTQCMRDVARRALEL

ISSVRDRKNDI

>GjilOBP84a

MQAVLMGIVVTCSCILSSTIIKAEENLPSFIAALDPALVEKGVACYDRFN

ISQDGLIPLMAGSDTPTNIKCFLHCVLSDCGMLSSSGEVVADRLRPIIDL

VPKDEFKTLLTNNLDDCCLVEKGTDECENAYLFASCFQKKISA

>GjilOBP1

MKPILAFVIFAVLAAAVRGQTDMKKDEIKEFIKARSEECKQDNTLTEDDI

ASLRERNVPTTASGKCFVDCMFEKLGMMENGQFSPEKSLQTAADVFTDDS

ERFEIAKKMIDGCKDEVGTSEEKCEAGPKLFLCLKKSAEGLDILPED

>GjilOBP19

MMQSIVVTFAAVLIATVSAQLFTGLKGQECAMKYGITKGDIDYVFGHKGT

LEDENNEQHRCFIECVFEACGVMKGGVFDASMARVEFEKLSQMMPEEGKS

VKVNAVIEAIGTCANEKGTGTCETAYKIFKCVMGKVMPGLHPNYVARVKE

CGEEHSVDGKERDIEVSFGSTGAVLDENDLSQKCFVACLADGTGVYRQAD

GKMDVERMMSEPHSIAFEVMVGNVSGEPLTREQFRADIEACAKIVDEDQC

EMGYKQIQCLRIFSLQLYKIVVDLNKSHNMTTPDIP

>GjilOBP14

MQIMNSFMFVPLMVAVAACSPMTSESSTTDQSAMSMEEMVQQCNTTYRID

AKYLEELNKTGKFPDETDQTPMCFIHCLFDKSGMMDAKGDFNVDHIQMVF

KNFENGTEIPDAKEMIDNCLSERSEYDICEKSYGLSKCLITEQIKKARES

EDSKAPQDKMGGKN

>GjilOBP8f

MKLIIFCCVLGAVTYSEAEMTMTQIKQATKMLRKVCQPKTGASTDMLDKL

QVGEFVQDPKVKCYIKCVLGMMNAMKKDVYSVEAALILADKMLPDEIREA

TKEVIRQCSDAGKDAPDACEMAFQLTKCAYTANPEIFIFP

>GjilOBP8

MKPNFCEMCNTVVVVVVVIACCAFHNVKCRVVSSEASSDLDIIRSCNKTS

PISLHGMNTVLVHRKFHKDTNSQFKCFLHCLYTKYNWMDDEGGFLLANMK

QVLEETDLDELTIDWVIFRCSAMDSMDKCERAYKFTECFWAETKEKPDNP

ESTFYNVEGH

>GjilOBP14b

MFGKFVIYIAVTIALVTANHLSDPMGAIGKCKDKYTVDEDTMKYAISNGM

LQNENDETARCFTECILNEFSVMKDGKLDVQGFINDTKVLSEKFGRQMPD

EAKLQADITACAATQGDGTCMKGYRTWKCLHQAIGSVMRPV

>GjilOBP56

MDENTLDEIKDSCFQQYSNISDGDWDNIRENNMPDSQAAKCYVGCFFEGI

GILIDGRFTTESVAKYHTMHKGYRPPEEILQEIYSVCSSTMTSSSDRCET

GPMLMRCFKQSMG

>BgerOBP1

MRAEFTRDSLKCTLQSDTMRFYGISLAVLSLVTVIQANEEKPFLKMNTIKIITACKQDNKA

QRDDYDAIRKSETPTTENGKCFLACLFDKFQVVQNGAFSVETFSKRLAELYKDDPDKLQK

AMNMANECKTSLNGVQETNKCQYAPKVLECFASYKTKIEVVQDLLKYQKIKSS

>BgerOBP2

MKGPTSAVLFAGLLLFASHEVPVRALEMSDMVSMAPQIISMGVEVIMNCMSENNMSIMK

TGASLMTARTIDQNAKCTVCCITSQIMITDGDKCSVNTAMIDTMVGALPKAVQDIVKKITK

GCTNAGAGKSKCQGLFDYGACIFKEGKNNIGSIFKMG

>BgerOBP3

MLLRGLIVLLGAVYCYAQSGPKEYALQCQKDYNIPDDVFKKIQWNLKATDEQNVNQRCFI

ECMLKAEGTIKNGELDQDFVVEEAKKDLVQVNLTLDEPKFRRSVATCAAQDGQGQCTRS

NNIWKCLADLLSGGLPLVS

>BgerOBP4

MRHLIVVLIAVLSLLVIEVELGTDEMRRIMQECLNQANIRSQDQLDFLKKKSMPVTTEGKC

FLGCLMEGYGYLIDGEFTEDSCEEWATRQGQSHEAAADLFQSCHSTVGDGDDRCETGPLL

WKCLLKLDNNQ

>BgerOBP5

MGGHHVVSLLCIAVMLVTPLSGAGVPDFYQQLPNFMTFLTTSQNVNPILSRVARQTKTSV

PCCGHEKFSWSIITDEFNGCTEEVGDIEKKERVPCFFECAGVKLGICDNEGYLKKDEAVELS

PKFVEGDEALENFVKQLAPKIADTVANIPDDVRKQLKCNIAVYAYLRGLHFHMELMCPIE

MQNPADECEKLRAEYRQMAEQKRG

>BgerOBP6

MTCRTKVFSNFIIILAVLHSIDGFTIEQVKKTANMVKDICIRKTAVDPEKVEQATNGNIPED

DNFKCFTKCLLEMLQAIRGDQYNSDGLIRMIKVLLPTDLGTRAITAIQQCNNAGDGLENIC

EVTYSIVVCFYKTDPEFLSLIL

>BgerOBP7

MKRITATLCVFLLAAAIRNSSAGMSEAQIKQAMKMIRQACQGKNSVSTEMLDGIQQGNFP

DDKNLKCYMKCVMGMMQSLKNGKYKPDAAIAQAKAMLPDEIKDRVIVSMDKCRNSGDG

IDDACELAYVVTKCIYASDPGAFMFP

>BgerOBP8F

MMKLHYILIVSCLFISTPISFAKMPSKGQVLQAIGMLYEHCQEKTNAEPDDISGICDGNFPE

DKVPLKEYTCCVLDMLRVMNGDKFEPDEALDNLKDLPDRVKKHLMEGINQCRNSGKNG

STCAETSYEVVKCFKATLGKYFFFPCGTDELPPEE

>BgerOBP9

MLPVVTGSVVICAILFSHLVVCEVTEDEIEKNCEEKFGVDESIFVDIDGDGNEDAIDEFDITL

KDEENEDHRCFIECLMEGYGYIKNGRLDVDFLVEEVLIELEEEGIEIDVDELRGDIAFCADQ

HAEGKCSTSYTIAKCLFNLQKKRRR

>BgerOBP10

MVSTKTFSLIVVCCLVYTVSAHYSREDDSNTVEDSGSASASRGINTGDKQISVKGSVSWQQ

TITVKQQSGAAPPHHGGHHGHRHHPHYFNMCCMVGTNLTDNPKLMKSLMKCRDEVMA

LFTNNGTFKPSPKNHLKASNCFFQCVGKEMDLVEENGSLKTKETVDFIATDYPLMELGVT

KEQIEACVITSKRSTPAPMPAEGGKVCNLVNFEAARFTKCLMDLKNLNCPTEKRVHTPIC

DHMREQWQRK

>BgerOBP11

MNMRNQIVIVLLSFYGYALCASKQGGLPKKVIEEcRKATGVTEAEAQELQENDLVKDESN

EAHRCFVACILSKRGAMKDGKMVVEEAVHAAKDGFKEAGFPFDEAKYRKGLEECNKQTG

EGKCVKSYNAWKCFLSFTSKTMIKSAEDKGK

>BgerOBP12

MLTTLICFLGSLLLAFAKPTFPQSPSTAEMRNDLALIRLCNQTSPISLATMNSVLINKQLSG

VSNAEGFKCFLHCLYNNYNWMDEDGGFMLSNMKGSLEATRLDELSIEFLIYKCTSVDSSD

RCERAFKFTECFWSETKTFPDESAAVDSSDYRIDWAAK

>BgerOBP13

MNNTGLVLALLLLGVSTPHVFAALTMDQMRNAAKMIRNVCQPKTGVATELIDQISEGVF

DEENRDLKCYIKCAMQMMQSMGSNGKLRPDAAISQAKKMLPIEIRDAMIAGIDTCRNVD

KENPGLDPCDLALVATKCVYNHTKDVFLFP

>BgerOBP14

MHRELLVLVLAVVIQLALAELSLEEIKQANEILRKHCQPESGVSDDVLNGAMSGNFPEDRG

LKCYLACMMRLAHVLKNGQYRSELAVRLADDVLPSSIKDRARVVVEKCKDEGAGLADECE

MAFAIKKCSYAADPEIFYVQ

>BgerOBP15

MHNRVLLPALVVILVALLGHTHALIILTQNEGTGKLIRSKCQPESGVSTDLIEGVPKGNFPD

DKNLKCYMKCAMSMTMTMRDDKLRIDIAKIMTERAVPEPNRSRILAAIDKCQNADQGLT

DPCEIAFAATKCIHDADSEVLLFP

>BgerOBP16

MSWDTALFLVLASVLCASAHHHHQEAASSSYGHPLTDRDALLNETRRVHPYFNPRDRER

PLITTCCDLSLNKSSSHTSPPAWVKEKCQLEVIAQFETESPESSSEEDLGLDVMNRNMTVC

LLECYFREMHLSTEDGIMIYDNVLKASVSNGKSQMGVTDRMVERCVVRANIIGPAMSQHK

GYACNFAPVEFEQCILERVDLLCPEYNQIRADLCDRYREKLRRKYTI

>BgerOBP17

MNRVLLASLCLVILATVLRPTLAGMSMDQIRQAMKMMRNSCQNKAKASPELVDGIQQGQ

FPDDRNLKCYIKCAMGMMQSMKGGKLKPEAAIQQAKMMLPDEVKGRVIAALETCRNAA

DGITDACDVAMAGAKCIYDTDPEAFIFP

>BgerOBP18

MEHHLFFLSLVTLFGTLSTLHVPKLDDLSDHDYLHKEVPSYFSKSCCNYNEPNAEDEMNI

FIDCRGIINDKYKLQRPKPTGQDEEDRITMISWIWNRSTCFSYCFLHKQGMANEDGTINVE

TAMKKQKKSIPELVNPEAWTNIEKLCTKPNDDDKETYVCKKDALHFSGCVHDISEFYCPD

ALQSKGESCVKYREDTKKKYGI

>BgerOBP19

MFSFKLKLLLAVVMWVCSADPTTLQEKQNIHKQCQEEYDISDEVFAEMRNTEMILEDES

DIKKKCTVECLLLGYGMIKDGQLDVTSVLDFAKPMLEHIQQEGGEIDEENLKENISKCSDT

VSGEEENCTSSYETWVCLHDVMTKLMAGSKNQE

>BgerOBP20

MVLKRIKLILFLSFISVTFSLKPEEIGISCQQKYKLSGEDIVEMRENRMILKDETNVNQRCFI

ECLMVESNMFVNGELNQEEVKKGEKELKEFYKSHGKELNLEKFDTSIQECSSKDDEGQCM

KSYQMWKCLVSAMPTDVVGGQPLKQQHL

>BgerOBP21

MKEYFVCVYILFIVEVLYLTDAGGEFLWRMKREANITEIKCCMQEGIPTGKRGLRDSIEMR

DIGKICFEEIQNKSSDINLEELNLERRVVFLECQLQCVFDKLKKVNEEGNLKKEETASSMIG

LFMIADRKFGDIFIEKCWKNLEGHAETKVEGHVCNPAAFNIYLCFEDIANLMQNVRITE

>BgerOBP22F

MHLLLPVAIFVVFVGFASAGNPMDSLDDDQKEMLKMLHDSCVGDSGADEGLIAKAIKGDF

TDDGNLKAYMACIFQNLGAMDENGVPDMDTMISMLPASMQERGGKMIGSCKGVTGSDA

ADTAMKLNQCFYNADPGYYFMF

>BgerOBP23

MQLSIELVCLLFAICSVKADSTKSFKNVIGPIARECMTKMDNVTEDDFQAVFNRNPLETRP

AMCLLKCVYQKLGVYKDDGALKEGDDLVPIIERIYGFNDSKNAMRIQAINTCVQKANADR

GEKCEEVAQFVRCVLQNY

>BgerOBP24

MKLPICIVFIFAAISAVSAESTQSFKDAVGPLLRECMGQVDGVTEDDFQTVYNRNKVETHS

TKCLLWCLYKSLGCYLPDGQLKAGDEMMPIIDKLYGFKEFKTVFRAQVVNNCVHEVNAES

SEDCEKAADFLHCLIVHY

>BgerOBP25F

MELQMKLCLGLALFTTIAAESTQSFKDGVEPHLKKCMEKVVDATEDDFNAVYNRNPLET

HSARCLLKCLYGELDALLPDGRIKSGDDLMPIMEKLYGFKEFKTVMRTQIVNQCVDMVNS

GEARDCELASDLIHCVMQHY

>BgerOBP26

MQIIFTVNAAPYFDGLPPEAEKMLKECNETFPIDMGYLKDLNDTGSFHDEDNKTPKCFIR

CVLMKAGLMDEDFTFDAPKLKEAFKDSKYPDMDNMIDMCIAKDTETQCRCTKAYLFIKC

LMSEEITKYGDASKEITKYGDASKVKTSN

>BgerOBP27

MATVGCVCLFLLSTVVSHVQPFFLARTLEKSEANHLIRMKRGSEEAYIDDDYVLVCCDELQ
[truncated: 1,258,304 more chars]
