# Supplementary material for: Transcriptome analysis and identification of genes related to environmental adaptation of Grylloprimevala jilina Zhou & Ren 2023
Source: Ecol Evol. 2023 Nov 20;13(11):e10717. doi: 10.1002/ece3.10717 (PMC10659822; doi:10.1002/ece3.10717)
Supplement: Supplementary file 2 — Appendix S2 [file ECE3-13-e10717-s001.docx]

**Supplemental Information.**

**Supplementary result.**

**Identification and homology analysis of reproduction-related genes**

Two spires with 339–372 aa and homologous similarity with known insects ranging from 71.43–85.09% (Table S10 and Figure S8). Six Tudor genes, encoding 270–1042 aa, were identified, and have 26.15–44.37% homologous similarity with known insects (Table S13 and Figure S11). One vasa was identified, which is 70.94% similar to other species and encodes 462 aa (Table S17 and Figure S15). Spire, Tudor, and vasa genes have similar functions, they regulate the production of germ cells, and their mutation results in reduced or no germ cells and other phenomena (Manseau, Schüpbach, & development, 1989). We found that Gjilspire-c160217 was expressed in all parts of the body, while Gjilspire-c162025 was barely expressed in the antennae. Among Tudor genes, GjilTudor6b, GjilTudor7, and GjilTudor6a were mainly expressed in the legs, heads, and antennae, while GjilTudor5b, GjilTudor5a, and GjilTudor1 were mainly expressed in the tails, thoraxesthoraces, and abdomens. Gjilvasa was expressed in 5 other tissues but was almost absent in the heads, suggesting its role in regulating the reproductive behavior of *G. jilina*. Among the Vg genes, GjilVg-c125869 and GjilVg-c177851 were expressed only in the abdomens and legs.

**Identification and homology analysis of temperature adaptation-related genes**

Forty-four heat shock proteins (HSPs), encoding 39 to 733 aa, were identified, which formed five intensive clusters in the phylogenetic tree (Table S19 and Figure S17). Eleven DnaJs, encoding 50–397 aa, were identified, which shared the lowest similarity of 67.93% with other species, indicating that these have specific functions in *G. jilina* (Table S20 and Figure S18). HSPs are highly conserved in all organisms. Under heat stimuli, HSPs are quickly produced for self-protection to reduce body damage (Dong et al., 2022). In this study, most HSPs were highly expressed in all tissues of *G. jilina*, indicating their role in the whole body. DnaJ, an HSP, is a ubiquitous molecular chaperone protein (Yang, Meng, Zhou, & Zhang, 2022). In this study, most DnaJs were highly expressed in the antennae.

References

Angeli, S., Ceron, F., Scaloni, A., Monti, M., Monteforti, G., Minnocci, A., . . . Pelosi, P. (1999). Purification, structural characterization, cloning and immunocytochemical localization of chemoreception proteins from Schistocerca gregaria. *European Journal of Biochemistry, 262*(3), 745-754.

Chen, Y., He, M., Li, Z.-Q., Zhang, Y.-N., & He, P. (2016). Identification and tissue expression profile of genes from three chemoreceptor families in an urban pest, Periplaneta americana. *Scientific reports, 6*(1), 27495.

Couto, A., Alenius, M., & Dickson, B. J. (2005). Molecular, anatomical, and functional organization of the Drosophila olfactory system. *Current Biology, 15*(17), 1535-1547.

Croset, V., Rytz, R., Cummins, S. F., Budd, A., Brawand, D., Kaessmann, H., . . . Benton, R. (2010). Ancient protostome origin of chemosensory ionotropic glutamate receptors and the evolution of insect taste and olfaction. *PLoS Genetics, 6*(8), e1001064.

Dong, B., Liu, X. Y., Li, B., Li, M. Y., Li, S. G., & Liu, S. (2022). A heat shock protein protects against oxidative stress induced by lambda-cyhalothrin in the green peach aphid *Myzus persicae*. *Pestic Biochem Physiol,* 181, 104995. <https://doi.org/10.1016/j.pestbp.2021.104995>

Gong, D.-P., Zhang, H.-j., Zhao, P., Lin, Y., Xia, Q.-Y., & Xiang, Z.-H. (2007). Identification and expression pattern of the chemosensory protein gene family in the silkworm, Bombyx mori. *Insect biochemistry and molecular biology, 37*(3), 266-277.

Gong, D.-P., Zhang, H.-J., Zhao, P., Xia, Q.-Y., & Xiang, Z.-H. (2009). The odorant binding protein gene family from the genome of silkworm, Bombyx mori. *BMC genomics, 10*(1), 1-14.

Guo, H., Cheng, T., Chen, Z., Jiang, L., Guo, Y., Liu, J., . . . Kadono-Okuda, K. (2017). Expression map of a complete set of gustatory receptor genes in chemosensory organs of Bombyx mori. *Insect biochemistry and molecular biology, 82*, 74-82.

Guo, M., Krieger, J., Große-Wilde, E., Mißbach, C., Zhang, L., & Breer, H. (2014). Variant ionotropic receptors are expressed in olfactory sensory neurons of coeloconic sensilla on the antenna of the desert locust (Schistocerca gregaria). *International Journal of Biological Sciences, 10*(1), 1.

He, M., Ma, Y.-F., Guo, H., Liu, X.-Z., Long, G.-J., Wang, Q., . . . He, P. (2022). Genome-wide identification and expression pattern analysis of novel chemosensory genes in the German cockroach Blattella germanica. *Genomics, 114*(2), 110310.

Jiang, X., Pregitzer, P., Grosse-Wilde, E., Breer, H., & Krieger, J. (2016). Identification and characterization of two “sensory neuron membrane proteins”(SNMPs) of the desert locust, Schistocerca gregaria (Orthoptera: Acrididae). *Journal of Insect Science, 16*(1), 33.

Li, H., Wang, P., Zhang, L., Xu, X., Cao, Z., & Zhang, L. (2018). Expressions of olfactory proteins in locust olfactory organs and a palp odorant receptor involved in plant aldehydes detection. *Frontiers in Physiology, 9*, 663.

Li, P.-Y., & Qin, Y.-C. (2011). Molecular cloning and characterization of sensory neuron membrane protein and expression pattern analysis in the diamondback moth, Plutella xylostella (Lepidoptera: Plutellidae). *Applied Entomology and Zoology, 46*, 497-504.

Liu, S., Rao, X.-J., Li, M.-Y., Feng, M.-F., He, M.-Z., & Li, S.-G. (2015). Identification of candidate chemosensory genes in the antennal transcriptome of Tenebrio molitor (Coleoptera: Tenebrionidae). *Comparative Biochemistry and Physiology Part D: Genomics and Proteomics, 13*, 44-51.

Manseau, L. J., & Schüpbach, T. (1989). cappuccino and spire: two unique maternal-effect loci required for both the anteroposterior and dorsoventral patterns of the *Drosophila* embryo. *10.1101/gad.3.9.1437,* 3(9), 1437-1452. <https://doi.org/10.1101/gad.3.9.1437>

Niu, D.-J., Liu, Y., Dong, X.-T., & Dong, S.-L. (2016). Transcriptome based identification and tissue expression profiles of chemosensory genes in Blattella germanica (Blattaria: Blattidae). *Comparative Biochemistry and Physiology Part D: Genomics and Proteomics, 18*, 30-43.

Olivier, V., Monsempes, C., François, M. C., Poivet, E., & Jacquin‐Joly, E. (2011). Candidate chemosensory ionotropic receptors in a Lepidoptera. *Insect molecular biology, 20*(2), 189-199.

Pregitzer, P., Jiang, X., Grosse-Wilde, E., Breer, H., Krieger, J., & Fleischer, J. (2017). In search for pheromone receptors: certain members of the odorant receptor family in the desert locust Schistocerca gregaria (Orthoptera: Acrididae) are co-expressed with SNMP1. *International Journal of Biological Sciences, 13*(7), 911.

Purandare, S. R., & Brisson, J. A. (2020). Divergent chemosensory gene expression accompanies ecological specialisation of pea aphid morphs. *Ecological Entomology, 45*(2), 364-368.

Robertson, H. M., Baits, R. L., Walden, K. K., Wada‐Katsumata, A., & Schal, C. (2018). Enormous expansion of the chemosensory gene repertoire in the omnivorous German cockroach Blattella germanica. *Journal of Experimental Zoology Part B: Molecular and Developmental Evolution, 330*(5), 265-278.

Robertson, H. M., Warr, C. G., & Carlson, J. R. (2003). Molecular evolution of the insect chemoreceptor gene superfamily in Drosophila melanogaster. *Proceedings of the National Academy of Sciences, 100*(suppl_2), 14537-14542.

Tassone, E. E., Geib, S. M., Hall, B., Brent, C. S., Fabrick, J. A., Brent, C. S., & Hull, J. J. (2016). De novo construction of an expanded transcriptome assembly for the western tarnished plant bug, Lygus hesperus. *GigaScience, 5*(1), s13742-13016-10109-13746.

Terrapon, N., Li, C., Robertson, H. M., Ji, L., Meng, X., Booth, W., . . . Gokhale, K. (2014). Molecular traces of alternative social organization in a termite genome. *Nature communications, 5*(1), 3636.

Vieira, F. G., & Rozas, J. (2011). Comparative genomics of the odorant-binding and chemosensory protein gene families across the Arthropoda: origin and evolutionary history of the chemosensory system. *Genome biology and evolution, 3*, 476-490.

Vogt, R. G., Miller, N. E., Litvack, R., Fandino, R. A., Sparks, J., Staples, J., . . . Dickens, J. C. (2009). The insect SNMP gene family. *Insect biochemistry and molecular biology, 39*(7), 448-456.

Wang, Z., Yang, P., Chen, D., Jiang, F., Li, Y., Wang, X., & Kang, L. (2015). Identification and functional analysis of olfactory receptor family reveal unusual characteristics of the olfactory system in the migratory locust. *Cellular and Molecular Life Sciences, 72*, 4429-4443.

Xu, Y.-L., He, P., Zhang, L., Fang, S.-Q., Dong, S.-L., Zhang, Y.-J., & Li, F. (2009). Large-scale identification of odorant-binding proteins and chemosensory proteins from expressed sequence tags in insects. *BMC genomics, 10*(1), 1-13.

Yang, C.-L., Meng, J.-Y., Zhou, L., & Zhang, C.-Y. (2022). Induced heat shock protein 70 confers biological tolerance in UV-B stress–adapted *Myzus persicae* (Hemiptera). *International Journal of Biological Macromolecules,* 220, 1146-1154. <https://doi.org/10.1016/j.ijbiomac.2022.08.159>

Yang, Y., Krieger, J., Zhang, L., & Breer, H. (2012). The olfactory co-receptor Orco from the migratory locust (Locusta migratoria) and the desert locust (Schistocerca gregaria): identification and expression pattern. *International Journal of Biological Sciences, 8*(2), 159.

Zhang, J., Liu, Y., Walker, W. B., Dong, S. L., & Wang, G. R. (2015). Identification and localization of two sensory neuron membrane proteins from Spodoptera litura (Lepidoptera: Noctuidae). *Insect science, 22*(3), 399-408.

Zhou, Y.-T., Li, L., Zhou, X.-R., Tan, Y., & Pang, B.-P. (2019). Identification and expression profiling of candidate chemosensory membrane proteins in the band-winged grasshopper, Oedaleus asiaticus. *Comparative Biochemistry and Physiology Part D: Genomics and Proteomics, 30*, 33-44.

**Supplementary materials.**


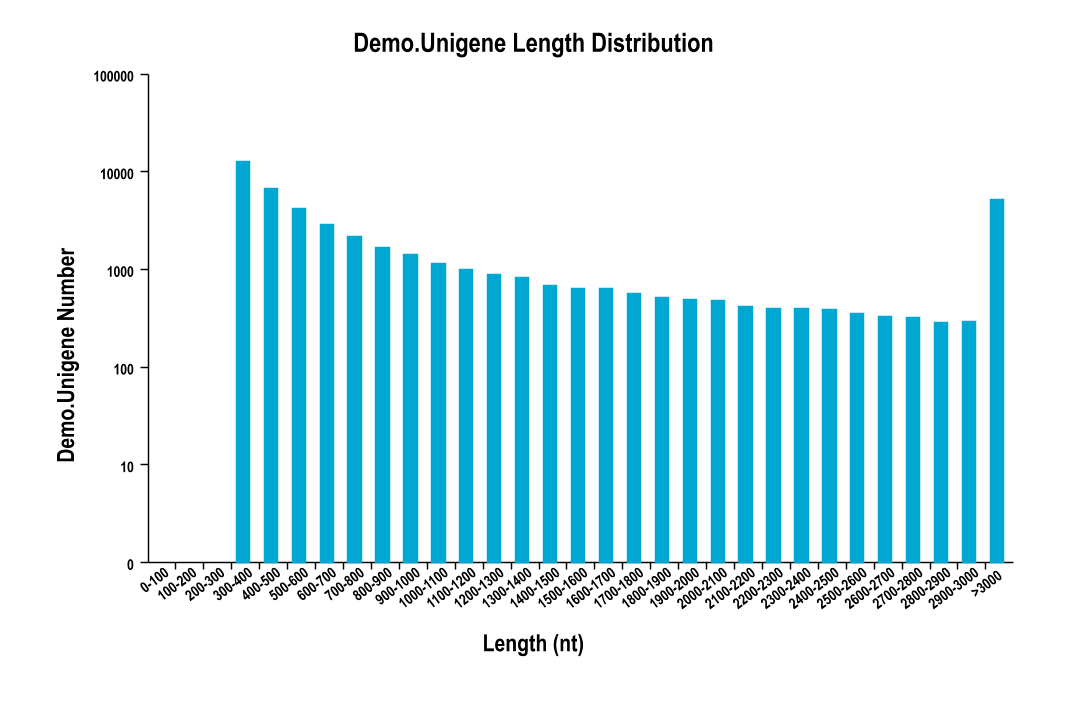


**Figure S1.** Size distribution of assembled Unigenes of *G. jilina*. Length distribution of unigene after splicing.


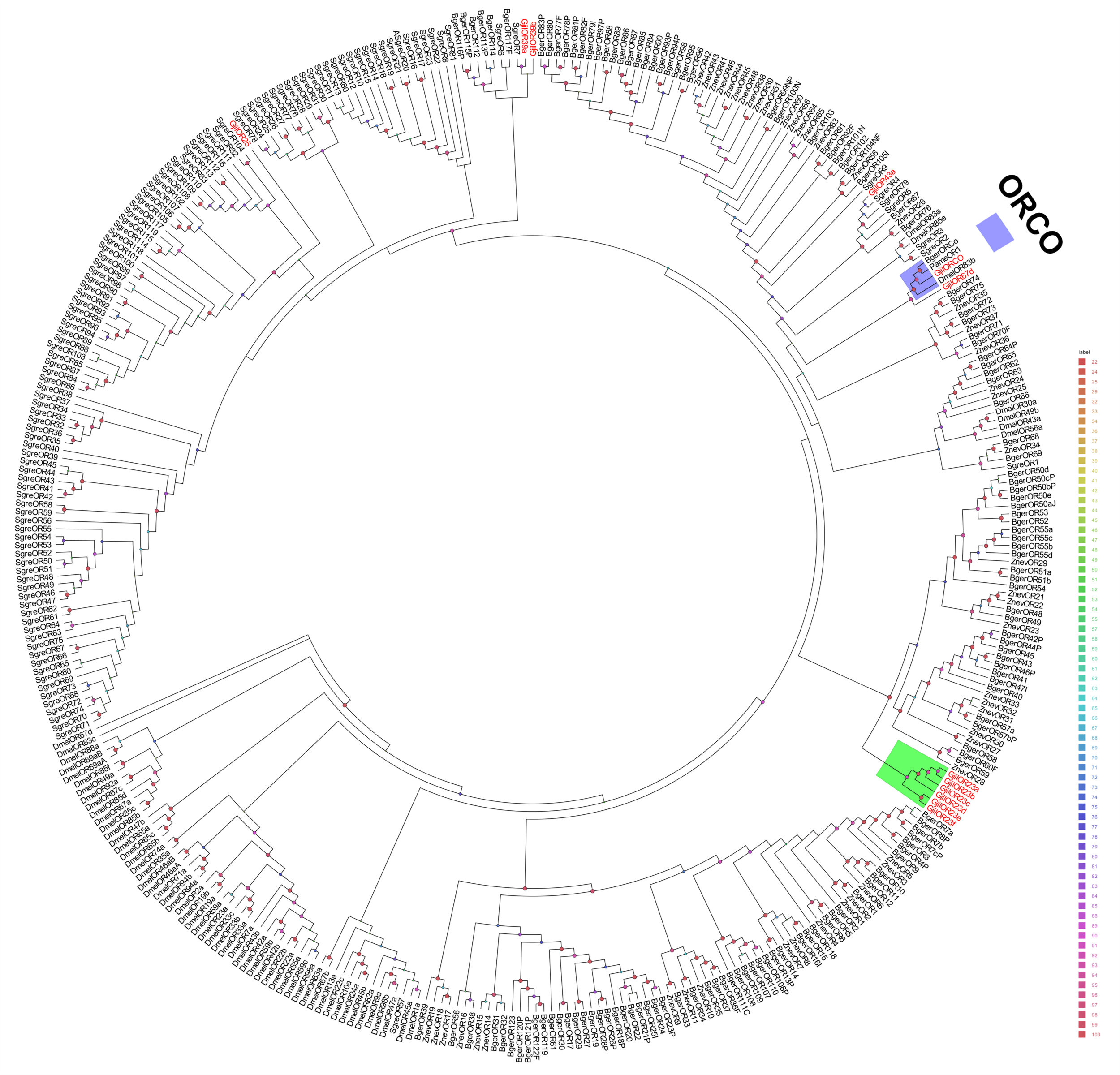


**Figure S2.** Homology analysis of ORs from *G. jilina* and other representative insect species. Bger (Robertson, Baits, Walden, Wada‐Katsumata, & Schal, 2018): *Blattella germanica*, Dmel (Couto, Alenius, & Dickson, 2005): *Drosophila melanogaster*, Sgre (Pregitzer et al., 2017): *Schistocerca gregaria*, Znev (Terrapon et al., 2014): *Zootermopsis nevadensis*, Pame (Chen, He, Li, Zhang, & He, 2016): *Periplaneta americana.*


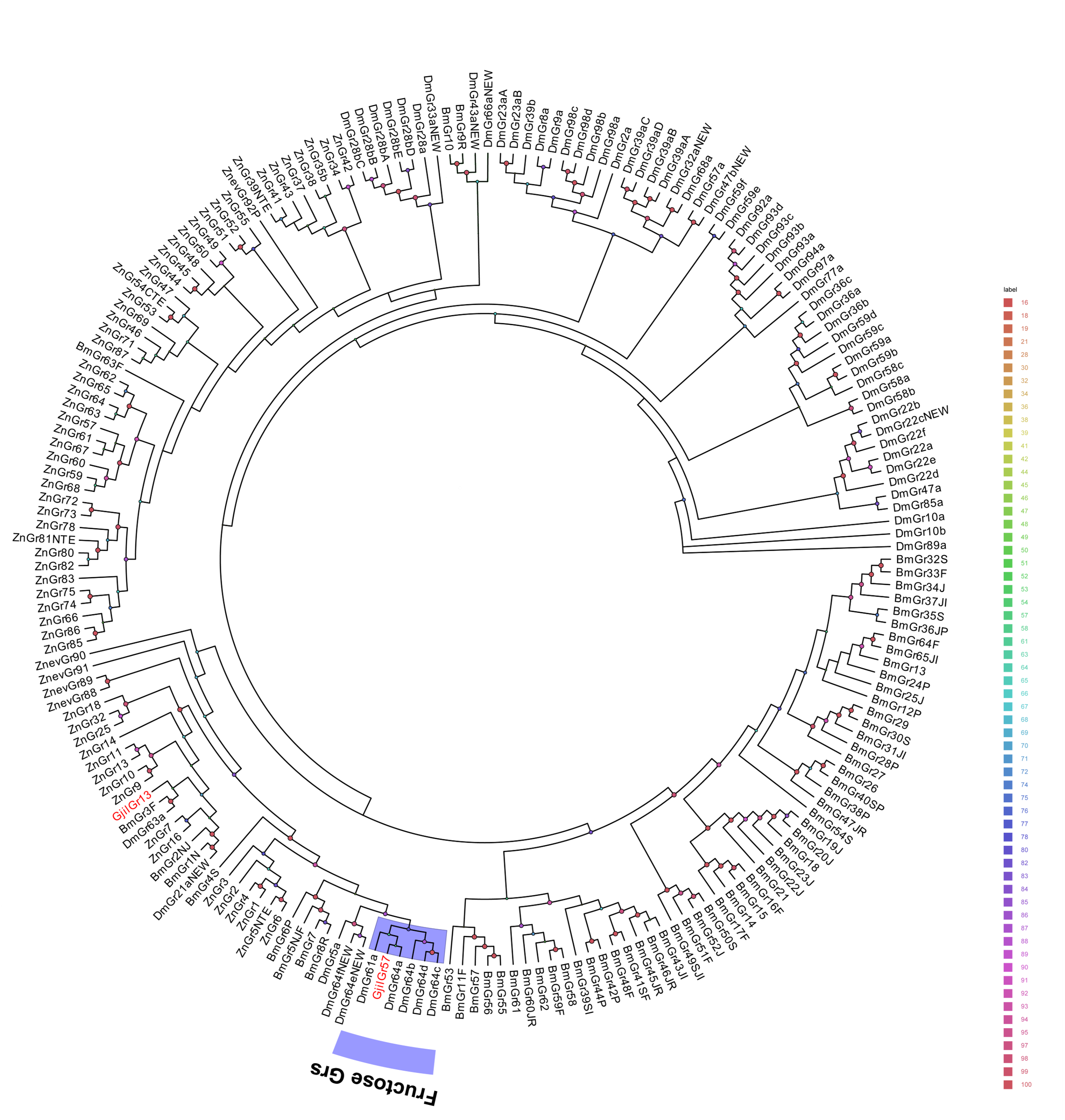


**Figure S3.** Homology analysis of GRs from *G. jilina* and other representative insect species. Bger (Robertson et al., 2018): *Blattella germanica*, Dmel (Robertson, Warr, & Carlson, 2003): *Drosophila melanogaster*, Bmor (H. Guo et al., 2017): *Bombyx mori*, Znev (Terrapon et al., 2014): *Zootermopsis nevadensis.*


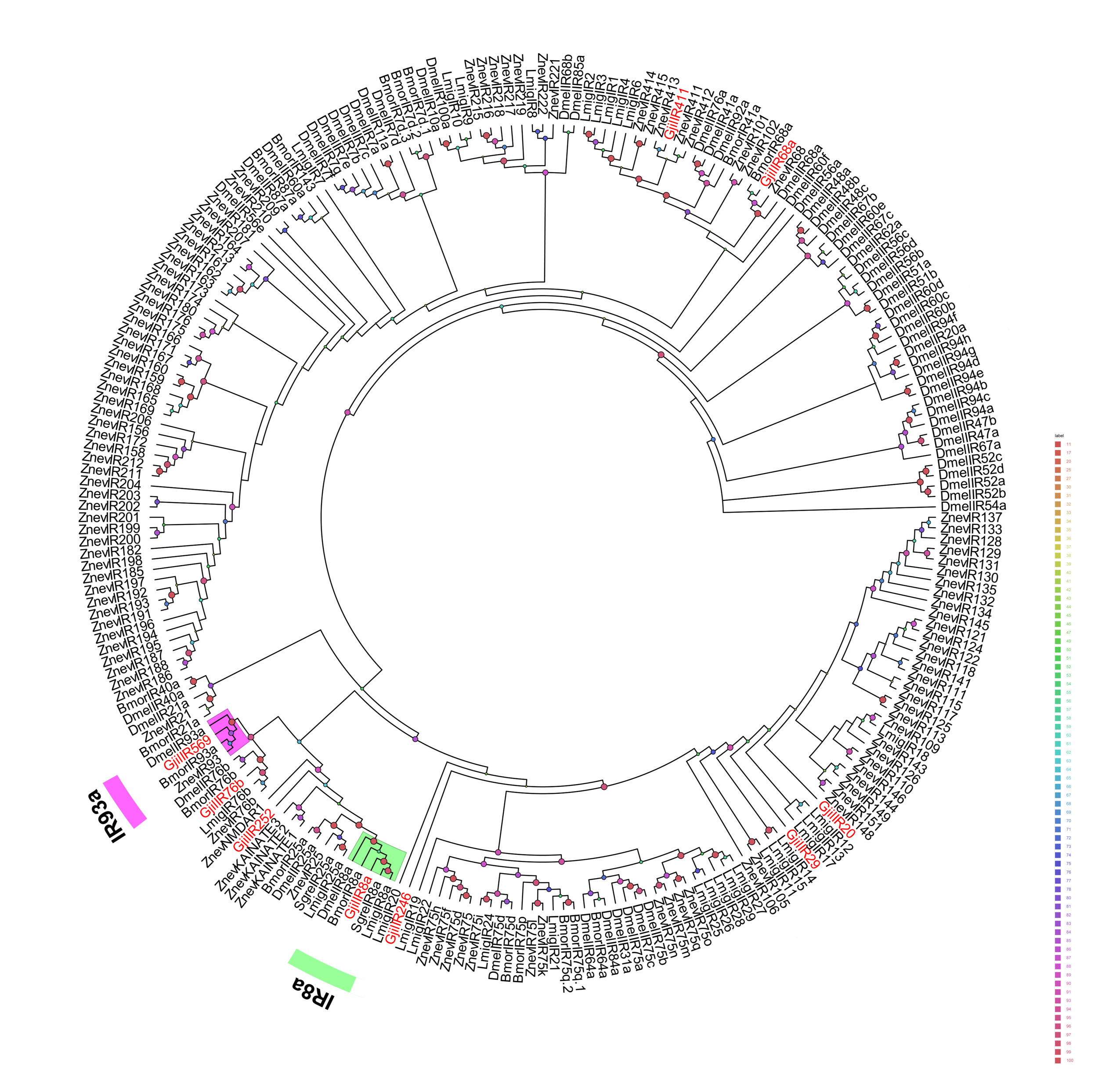


**Figure S4.** Homology analysis of IRs from *G. jilina* and other representative insect species. Lmig (Wang et al., 2015): *Locusta migratoria*, Dmel (Croset et al., 2010): *Drosophila melanogaster*, Bmor (Olivier, Monsempes, François, Poivet, & Jacquin‐Joly, 2011): *Bombyx mori*, Pame (Chen et al., 2016): *Periplaneta americana*, Sgre (M. Guo et al., 2014): *Schistocerca gregaria*, Znev (Terrapon et al., 2014): *Zootermopsis nevadensis.*


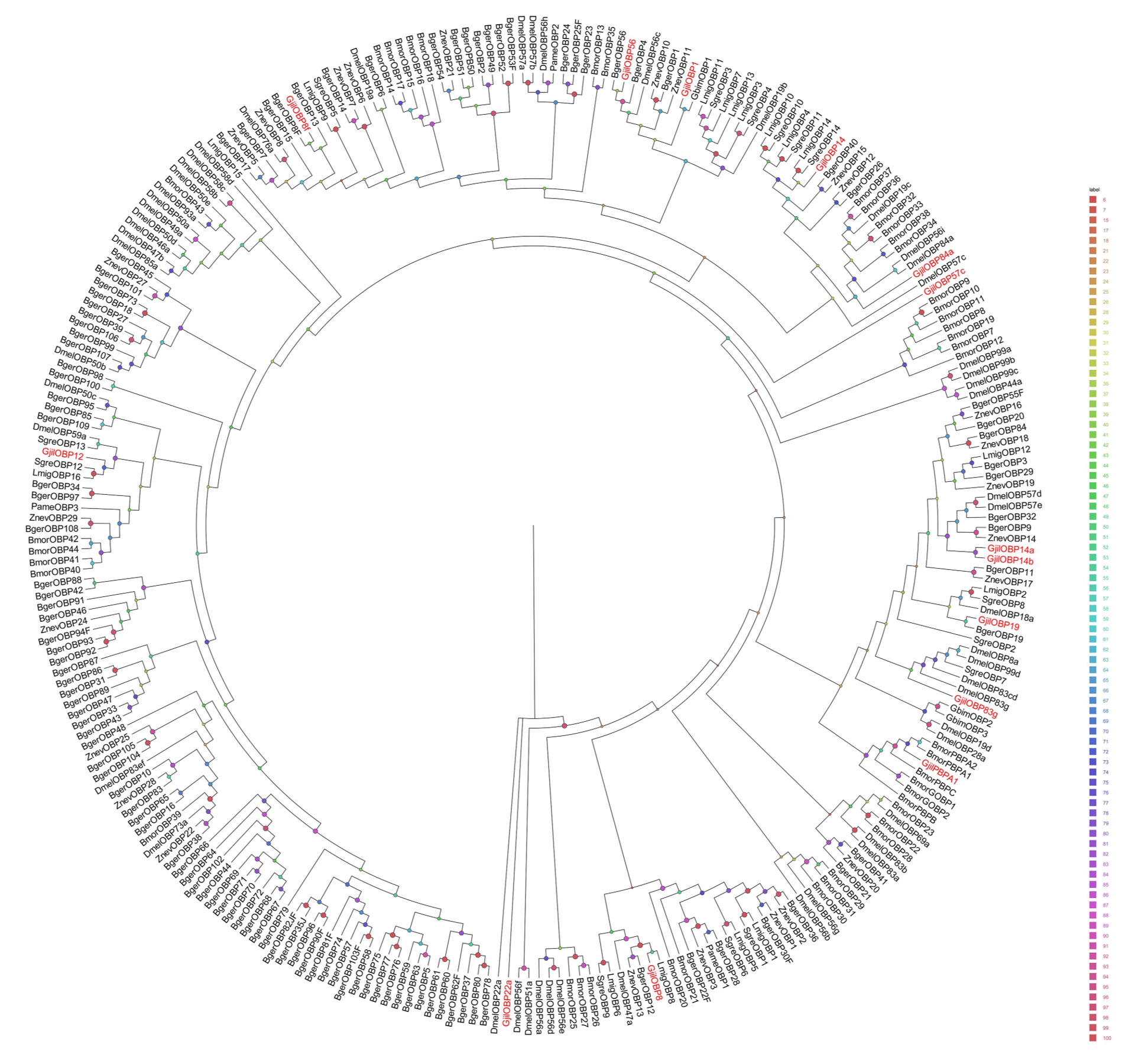


**Figure S5.** Homology analysis of OBPs from *G. jilina* and other representative insect species. Bger (Niu, Liu, Dong, & Dong, 2016): *Blattella germanica*, Lmig (H. Li et al., 2018): *Locusta migratoria*, Dmel (Vieira & Rozas, 2011): *Drosophila melanogaster*, Bmor (Gong, Zhang, Zhao, Xia, & Xiang, 2009):*Bombyx mori*, Pame (Xu et al., 2009): *Periplaneta americana*, Sgre (Yang, Krieger, Zhang, & Breer, 2012): *Schistocerca gregaria*, Znev (Terrapon et al., 2014): *Zootermopsis nevadensis*, Gbim (Xu et al., 2009): *Gryllus bimaculatus.*


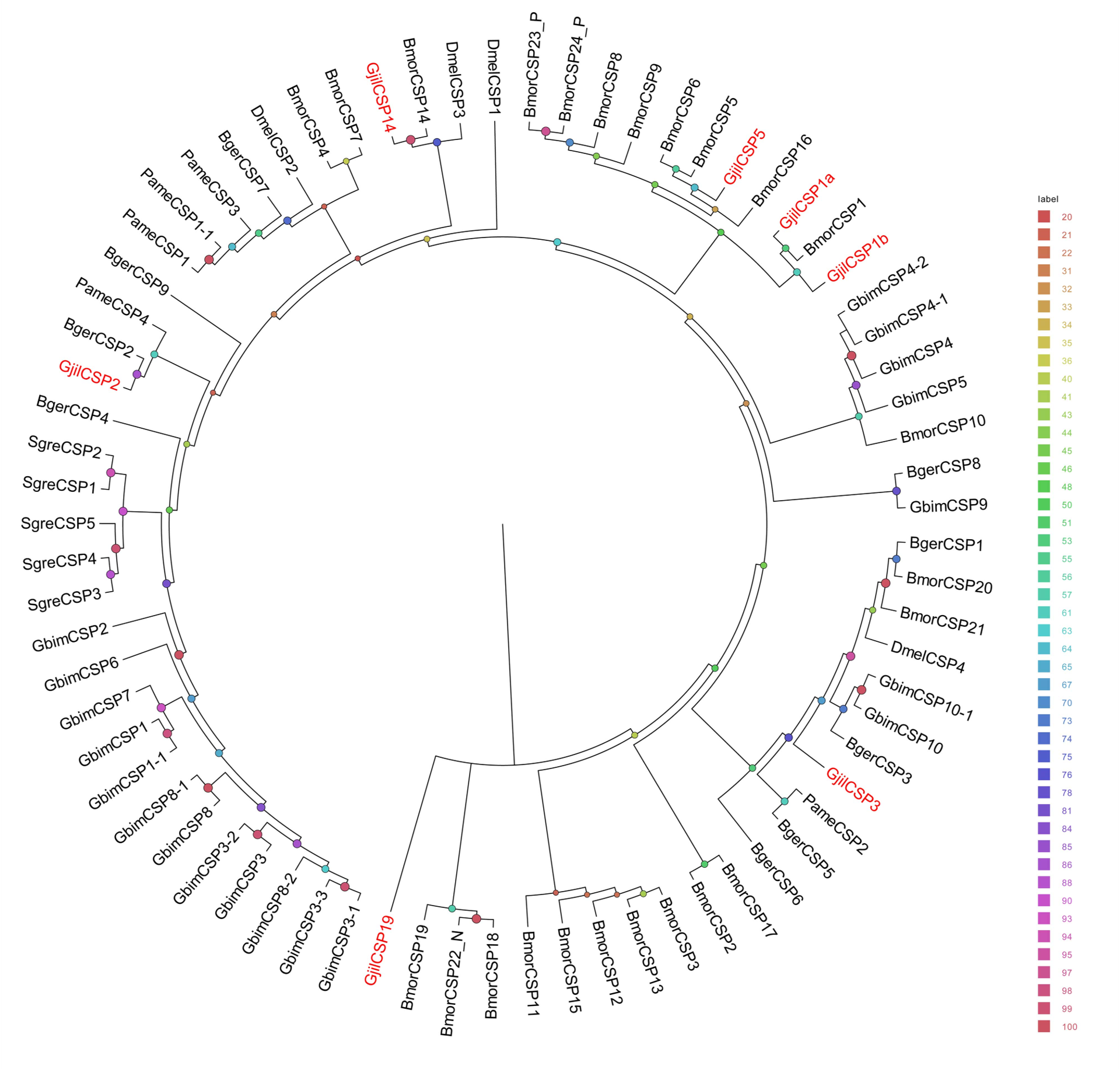


**Figure S6.** Homology analysis of CSPs from *G. jilina* and other representative insect species. Bger (Niu et al., 2016): *Blattella germanica*, Dmel (Vieira & Rozas, 2011): *Drosophila melanogaster*, Bmor (Gong et al., 2007): *Bombyx mori*, Pame (Xu et al., 2009): *Periplaneta americana*, Sgre (Angeli et al., 1999): *Schistocerca gregaria*, Gbim (Xu et al., 2009): *Gryllus bimaculatus.*


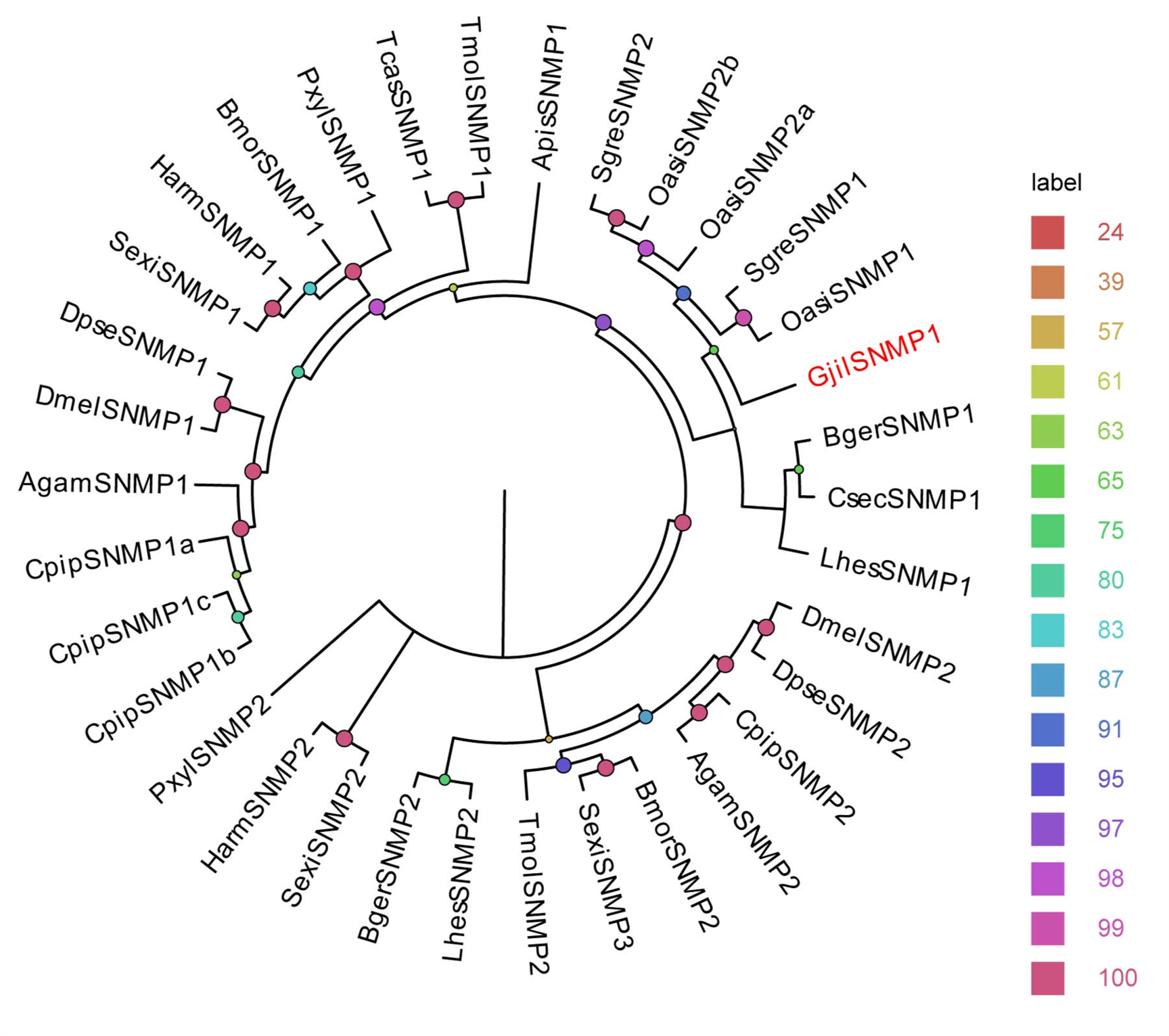


**Figure S7.** Homology analysis of SNMPs from *G. jilina* and other representative insect species. Bger (Niu et al., 2016): *Blattella germanica*, Dmel (Vogt et al., 2009): *Drosophila melanogaster*, Bmor (Vogt et al., 2009): *Bombyx mori*, Sgre (Jiang, Pregitzer, Grosse-Wilde, Breer, & Krieger, 2016): *Schistocerca gregaria*, Dpse (Vogt et al., 2009): *Drosophila pseudoobscura*, Cpip (Vogt et al., 2009): *Culex pipiens*, Oasi (Zhou, Li, Zhou, Tan, & Pang, 2019): *Oedaleus asiaticus*, Sexi (Zhang, Liu, Walker, Dong, & Wang, 2015): *Spodoptera exigua*, Pxyl (P.-Y. Li & Qin, 2011): *Plutella xylostella*, Harm (Vogt et al., 2009): *Helicoverpa armigera*, Tcas (Vogt et al., 2009): *Tribolium castaneum*, Agam (Vogt et al., 2009): *Anopheles gambiae*, Tmol (Liu et al., 2015): *Tenebrio molitor*, Lhes (Tassone et al., 2016): *Lygus hesperus*, Apis (Purandare & Brisson, 2020): *Acyrthosiphon pisum*, Csec (He et al., 2022) : *Cryptotermes secundus.*


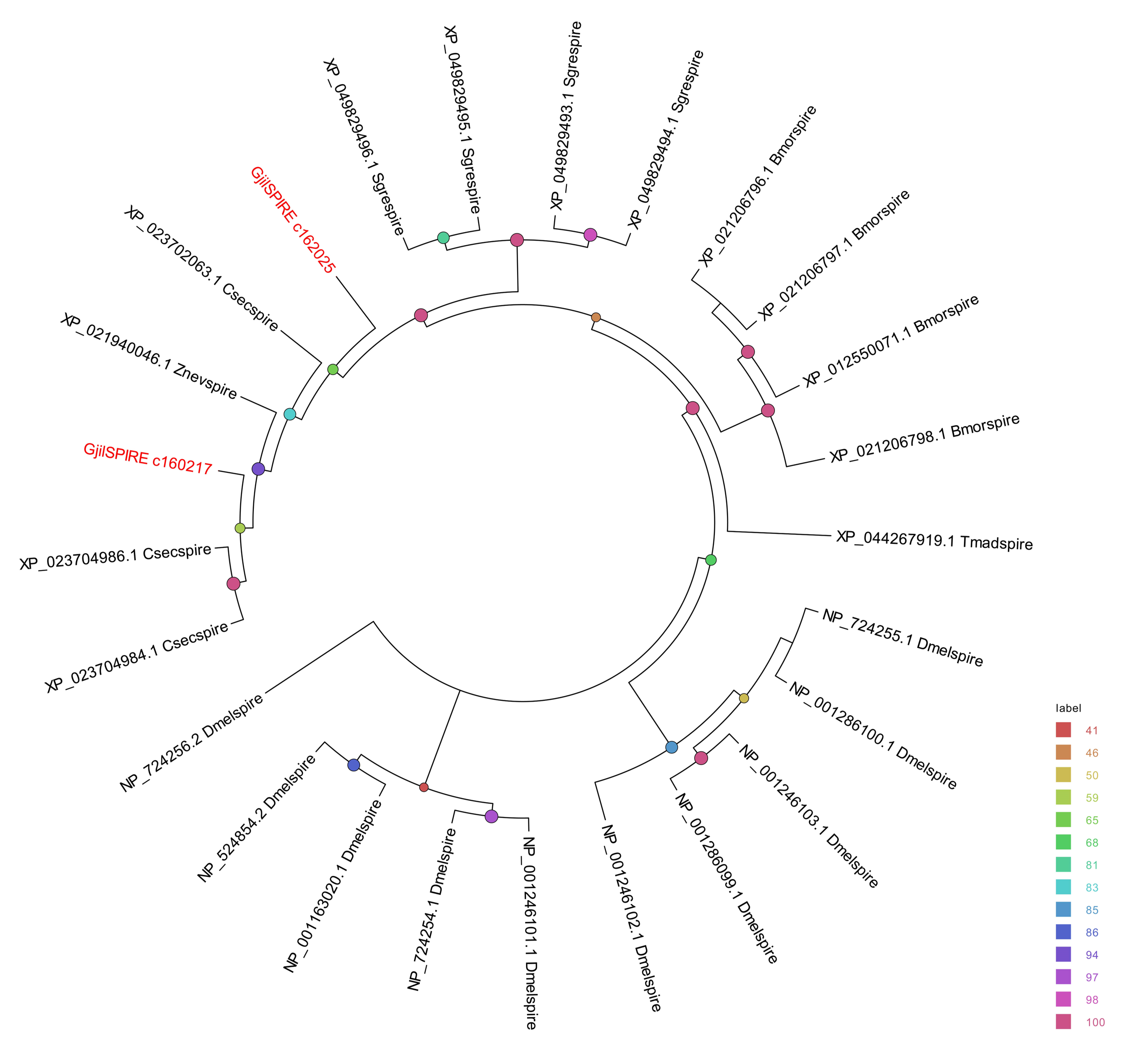


**Figure S8.** Homology analysis of spires from *G. jilina* and other representative insect species. Among them, the homologous genes of other species are screened from the NCBI genome. Bmor: *Bombyx mori* (GCF_014905235.1), Csec: *Cryptotermes secundus* (GCF_002891405.2), Dmel: *Drosophila melanogaster* (GCF_000001215.4), Sgre: *Schistocerca gregaria* (GCF_023897955.1)*,* Tmad: *Tribolium madens* (GCF_015345945.1), Znev: *Zootermopsis nevadensis* (GCF_000696155.1).


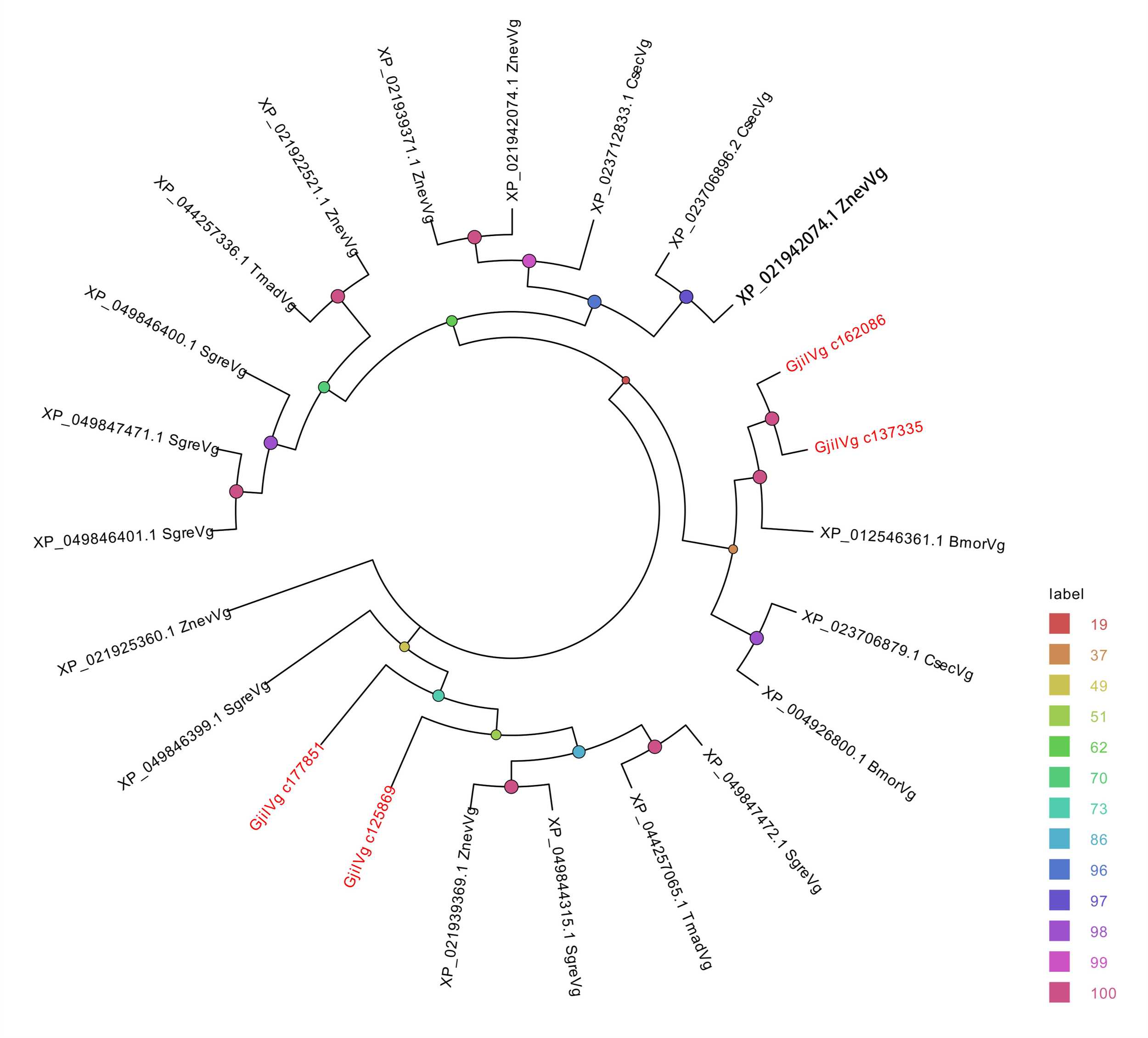


**Figure S9.** Homology analysis of Vgs from *G. jilina* and other representative insect species. Among them, the homologous genes of other species are screened from the NCBI genome. Bmor: *Bombyx mori* (GCF_014905235.1), Csec: *Cryptotermes secundus* (GCF_002891405.2), Sgre: *Schistocerca gregaria* (GCF_023897955.1)*,* Tmad: *Tribolium madens* (GCF_015345945.1), Znev: *Zootermopsis nevadensis* (GCF_000696155.1).


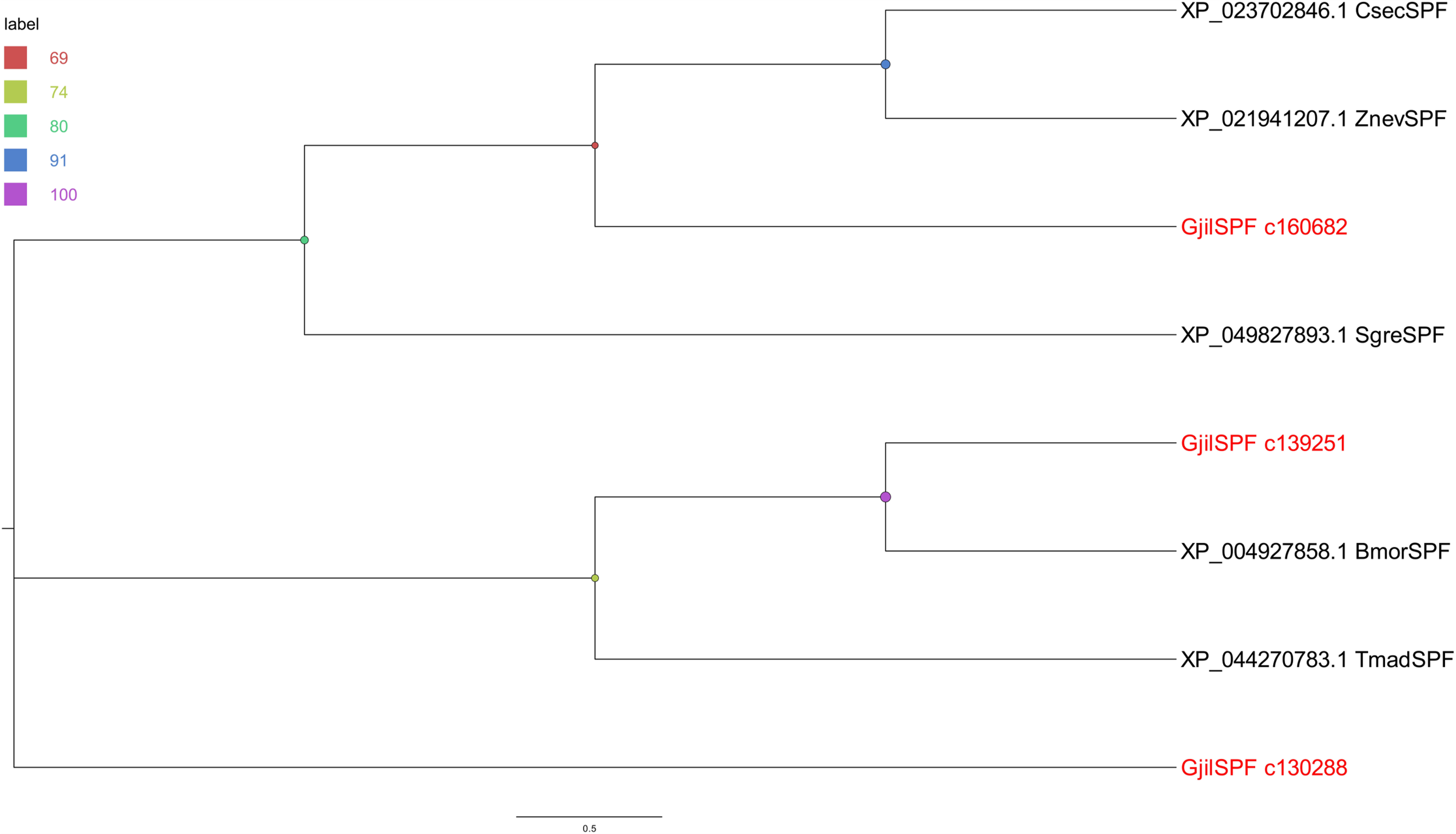


**Figure S10.** Homology analysis of SPFs from *G. jilina* and other representative insect species. Among them, the homologous genes of other species are screened from the NCBI genome. Bmor: *Bombyx mori* (GCF_014905235.1), Csec: *Cryptotermes secundus* (GCF_002891405.2), Sgre: *Schistocerca gregaria* (GCF_023897955.1)*,* Tmad: *Tribolium madens* (GCF_015345945.1), Znev: *Zootermopsis nevadensis* (GCF_000696155.1).


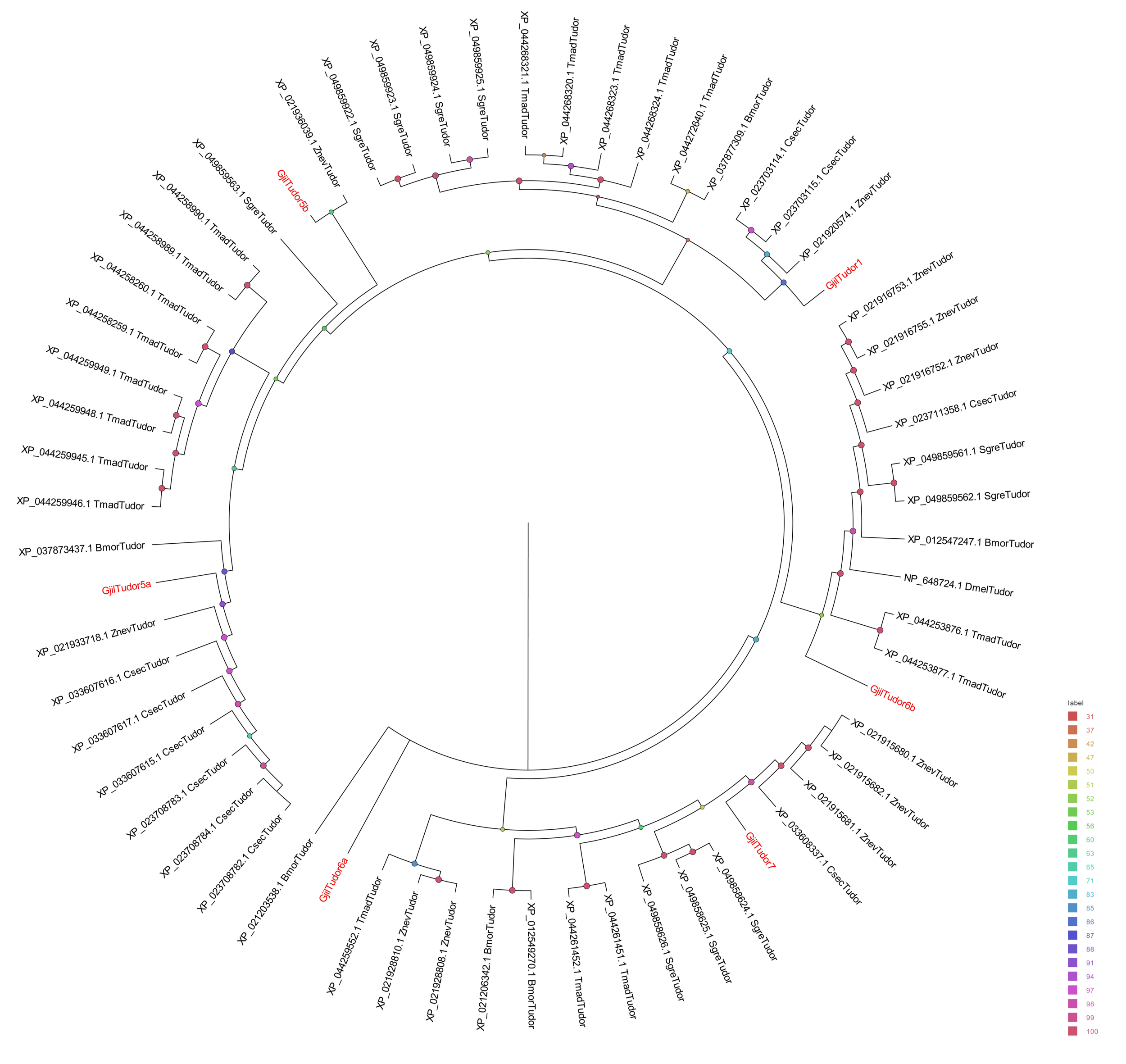


**Figure S11.** Homology analysis of Tudors from *G. jilina* and other representative insect species. Among them, the homologous genes of other species are screened from the NCBI genome. Bmor: *Bombyx mori* (GCF_014905235.1), Csec: *Cryptotermes secundus* (GCF_002891405.2), Dmel: *Drosophila melanogaster* (GCF_000001215.4), Sgre: *Schistocerca gregaria* (GCF_023897955.1)*,* Tmad: *Tribolium madens* (GCF_015345945.1), Znev: *Zootermopsis nevadensis* (GCF_000696155.1).


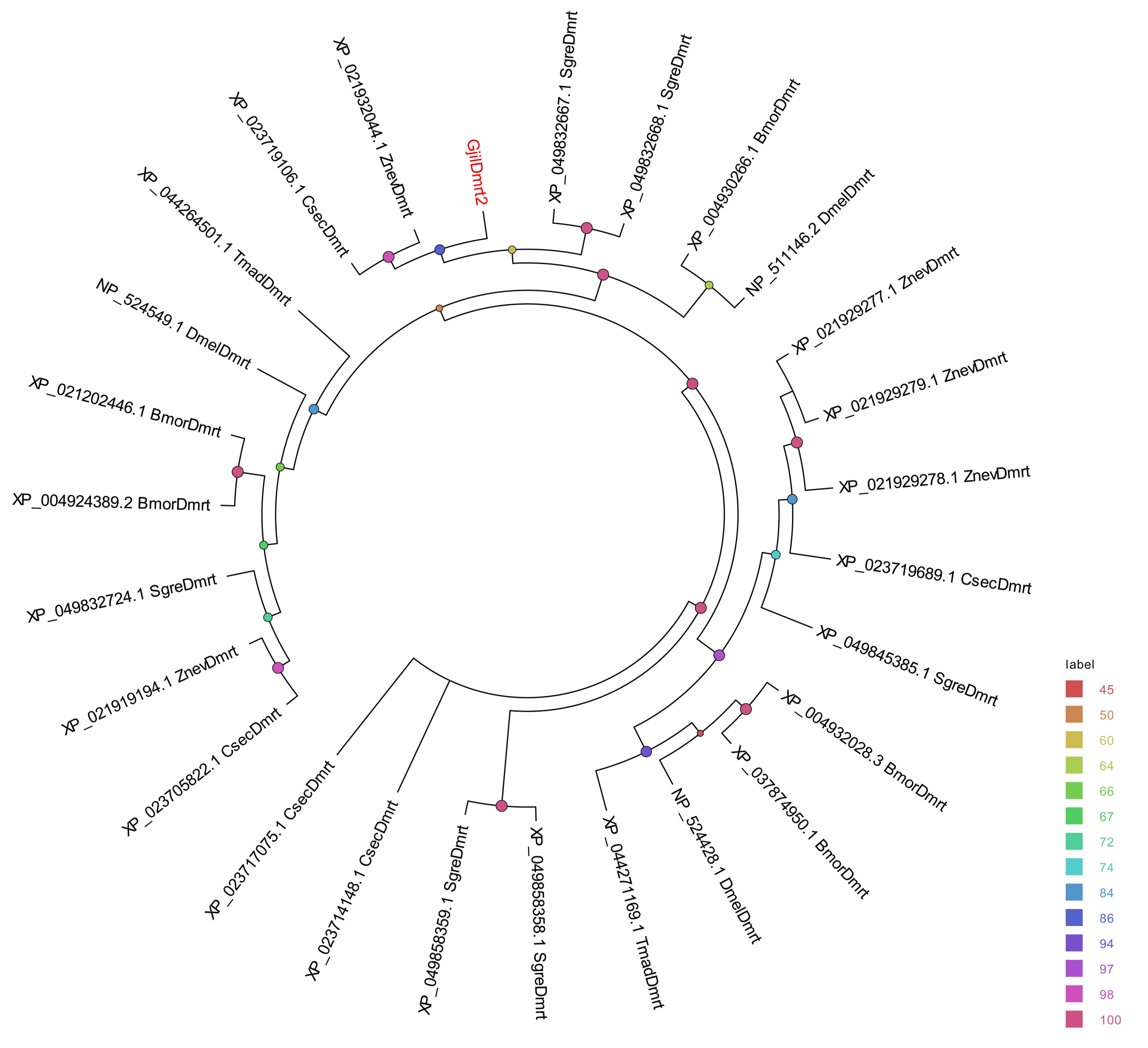


**Figure S12.** Homology analysis of Dmrts from *G. jilina* and other representative insect species. Among them, the homologous genes of other species are screened from the NCBI genome. Bmor: *Bombyx mori* (GCF_014905235.1), Csec: *Cryptotermes secundus* (GCF_002891405.2), Dmel: *Drosophila melanogaster* (GCF_000001215.4), Sgre: *Schistocerca gregaria* (GCF_023897955.1)*,* Tmad: *Tribolium madens* (GCF_015345945.1), Znev: *Zootermopsis nevadensis* (GCF_000696155.1).


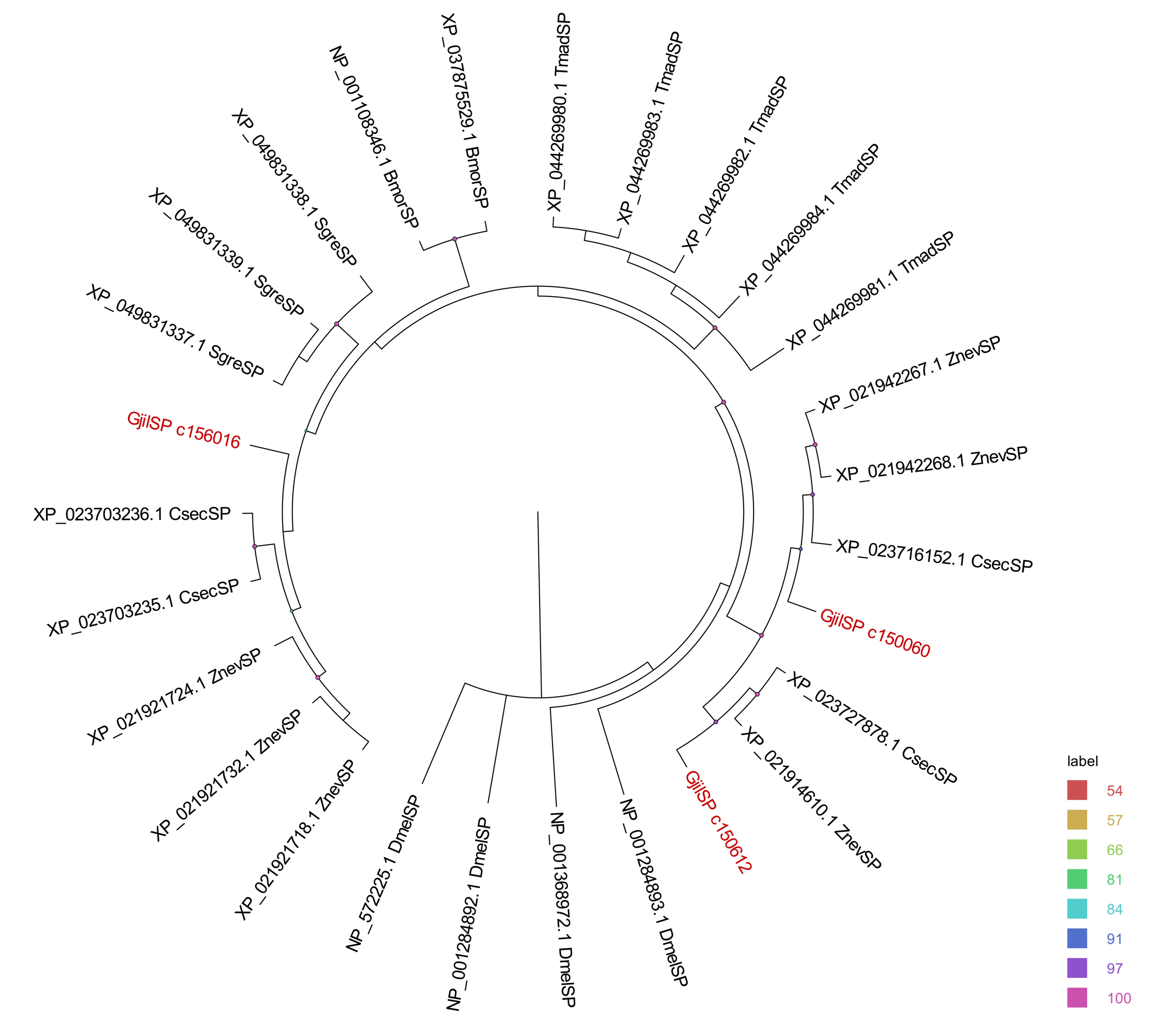


**Figure S13.** Homology analysis of SPs from *G. jilina* and other representative insect species. Among them, the homologous genes of other species are screened from the NCBI genome. Bmor: *Bombyx mori* (GCF_014905235.1), Csec: *Cryptotermes secundus* (GCF_002891405.2), Dmel: *Drosophila melanogaster* (GCF_000001215.4), Sgre: *Schistocerca gregaria* (GCF_023897955.1)*,* Tmad: *Tribolium madens* (GCF_015345945.1), Znev: *Zootermopsis nevadensis* (GCF_000696155.1).


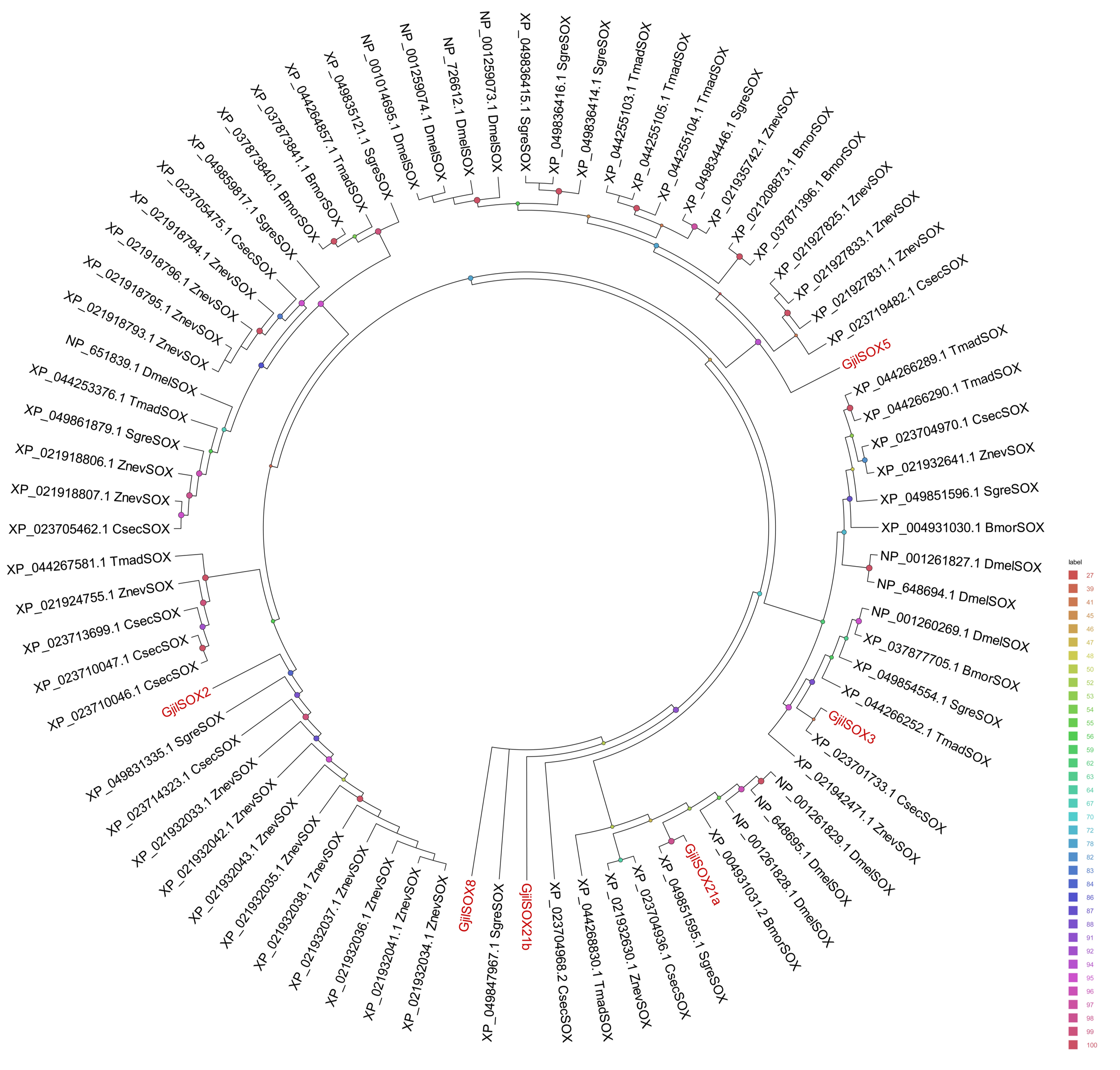


**Figure S14.** Homology analysis of SOXs from *G. jilina* and other representative insect species. Among them, the homologous genes of other species are screened from the NCBI genome. Bmor: *Bombyx mori* (GCF_000696155.1), Csec: *Cryptotermes secundus* (GCF_002891405.2), Dmel: *Drosophila melanogaster* (GCF_000001215.4), Sgre: *Schistocerca gregaria* (GCF_023897955.1)*,* Tmad: *Tribolium madens* (GCF_015345945.1), Znev: *Zootermopsis nevadensis* (GCF_000696155.1).


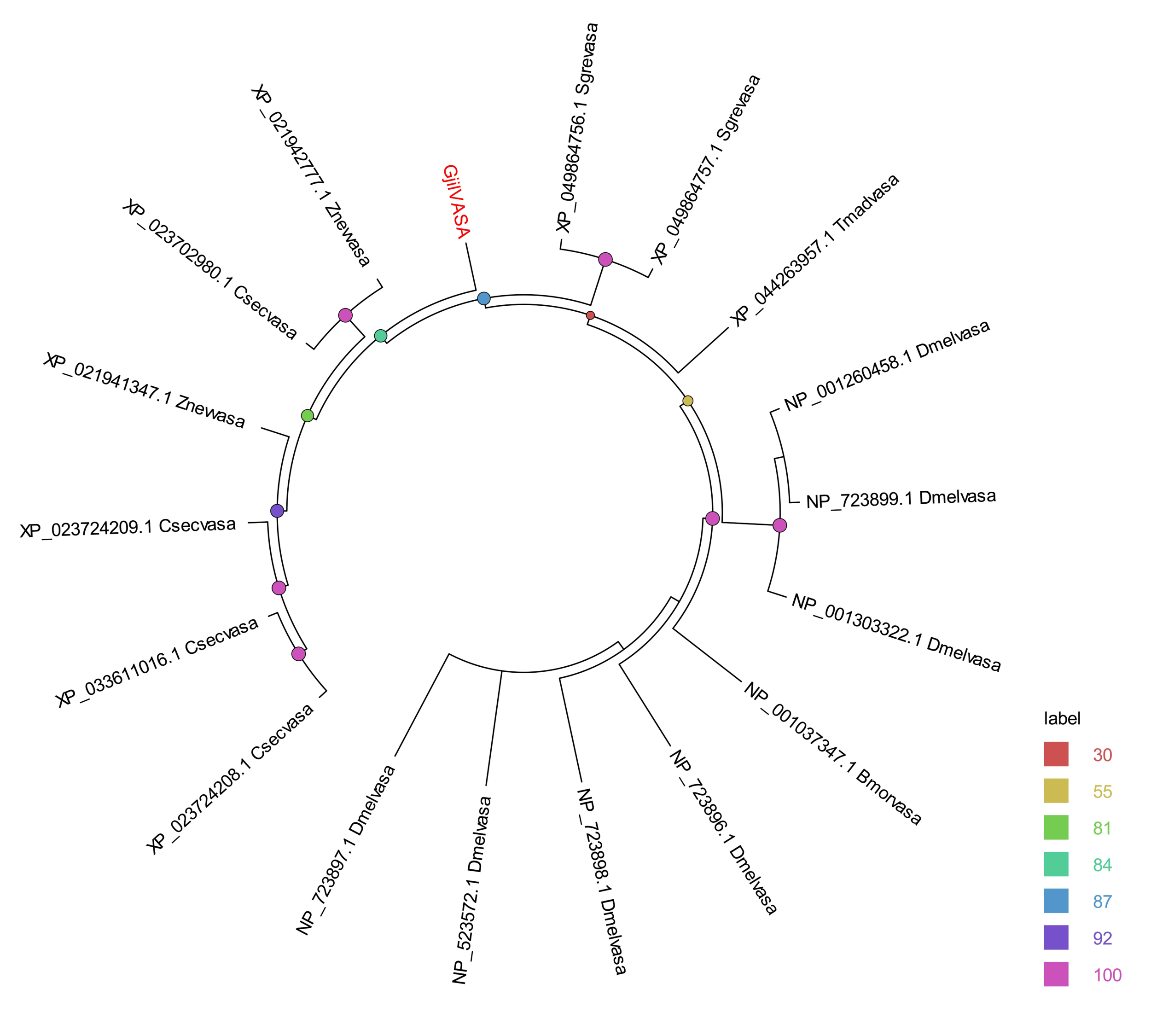


**Figure S15.** Homology analysis of vasas from *G. jilina* and other representative insect species. Among them, the homologous genes of other species are screened from the NCBI genome. Bmor: *Bombyx mori* (GCF_014905235.1), Csec: *Cryptotermes secundus* (GCF_002891405.2), Dmel: *Drosophila melanogaster* (GCF_000001215.4), Sgre: *Schistocerca gregaria* (GCF_023897955.1)*,* Tmad: *Tribolium madens* (GCF_015345945.1), Znev: *Zootermopsis nevadensis* (GCF_000696155.1).


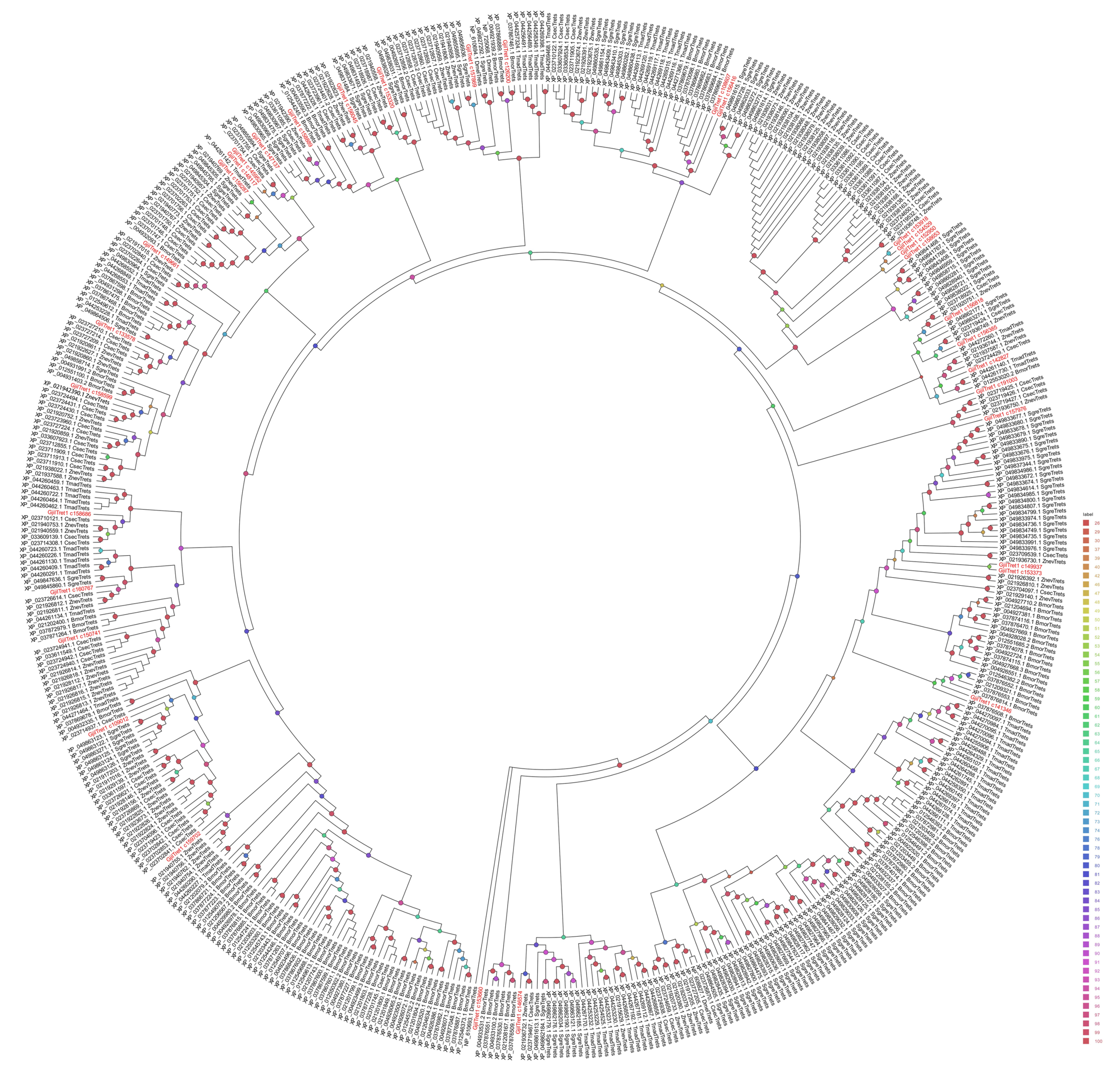


**Figure S16.** Homology analysis of Trets from *G. jilina* and other representative insect species. Among them, the homologous genes of other species are screened from the NCBI genome. Bmor: *Bombyx mori* (GCF_014905235.1), Csec: *Cryptotermes secundus* (GCF_002891405.2), Dmel: *Drosophila melanogaster* (GCF_000001215.4), Sgre: *Schistocerca gregaria* (GCF_023897955.1)*,* Tmad: *Tribolium madens* (GCF_015345945.1), Znev: *Zootermopsis nevadensis* (GCF_000696155.1).


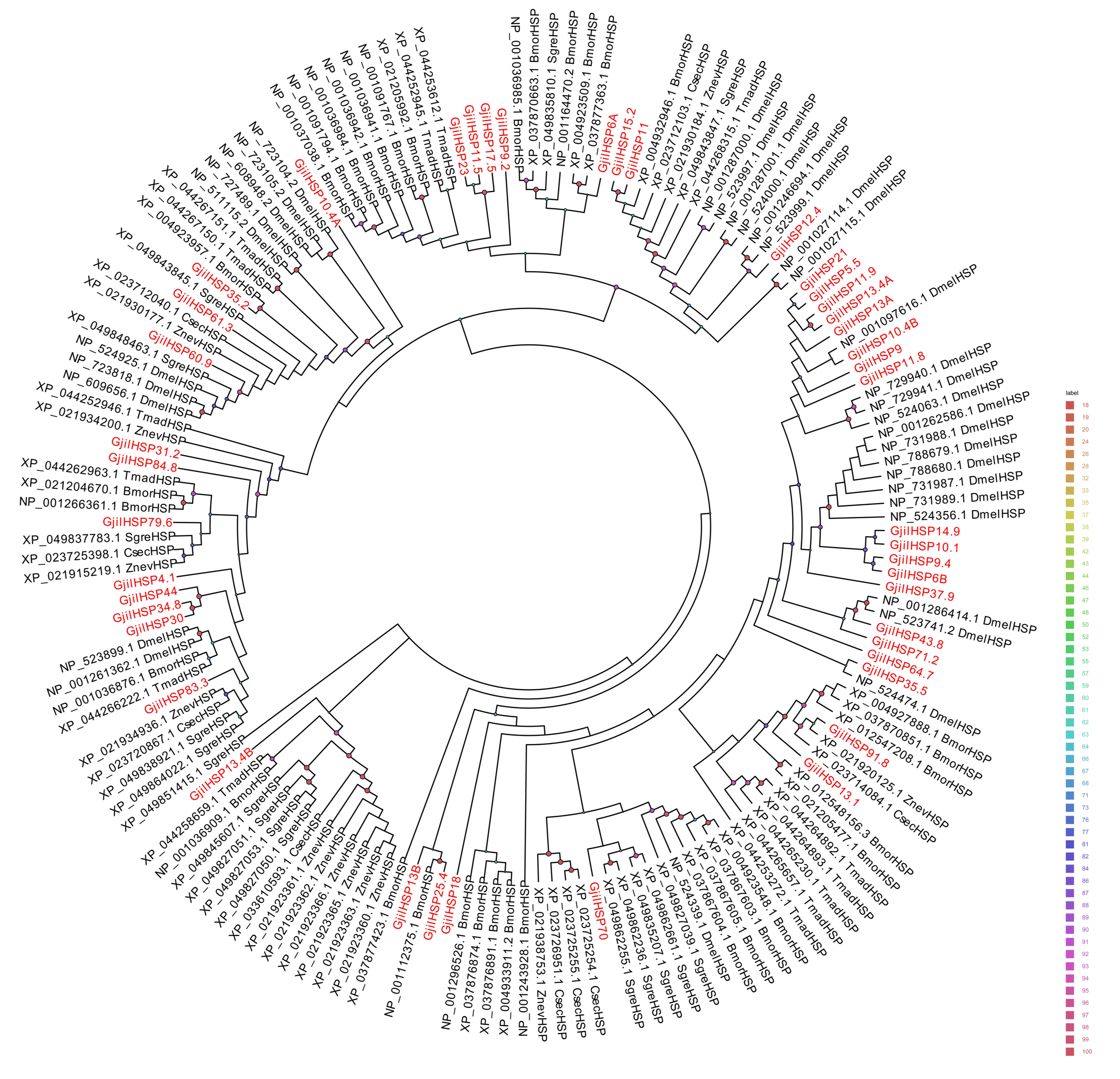


**Figure S17.** Homology analysis of HSPs from *G. jilina* and other representative insect species. Among them, the homologous genes of other species are screened from the NCBI genome. Bmor: *Bombyx mori* (GCF_014905235.1), Csec: *Cryptotermes secundus* (GCF_002891405.2), Dmel: *Drosophila melanogaster* (GCF_000001215.4), Sgre: *Schistocerca gregaria* (GCF_023897955.1)*,* Tmad: *Tribolium madens* (GCF_015345945.1), Znev: *Zootermopsis nevadensis* (GCF_000696155.1).


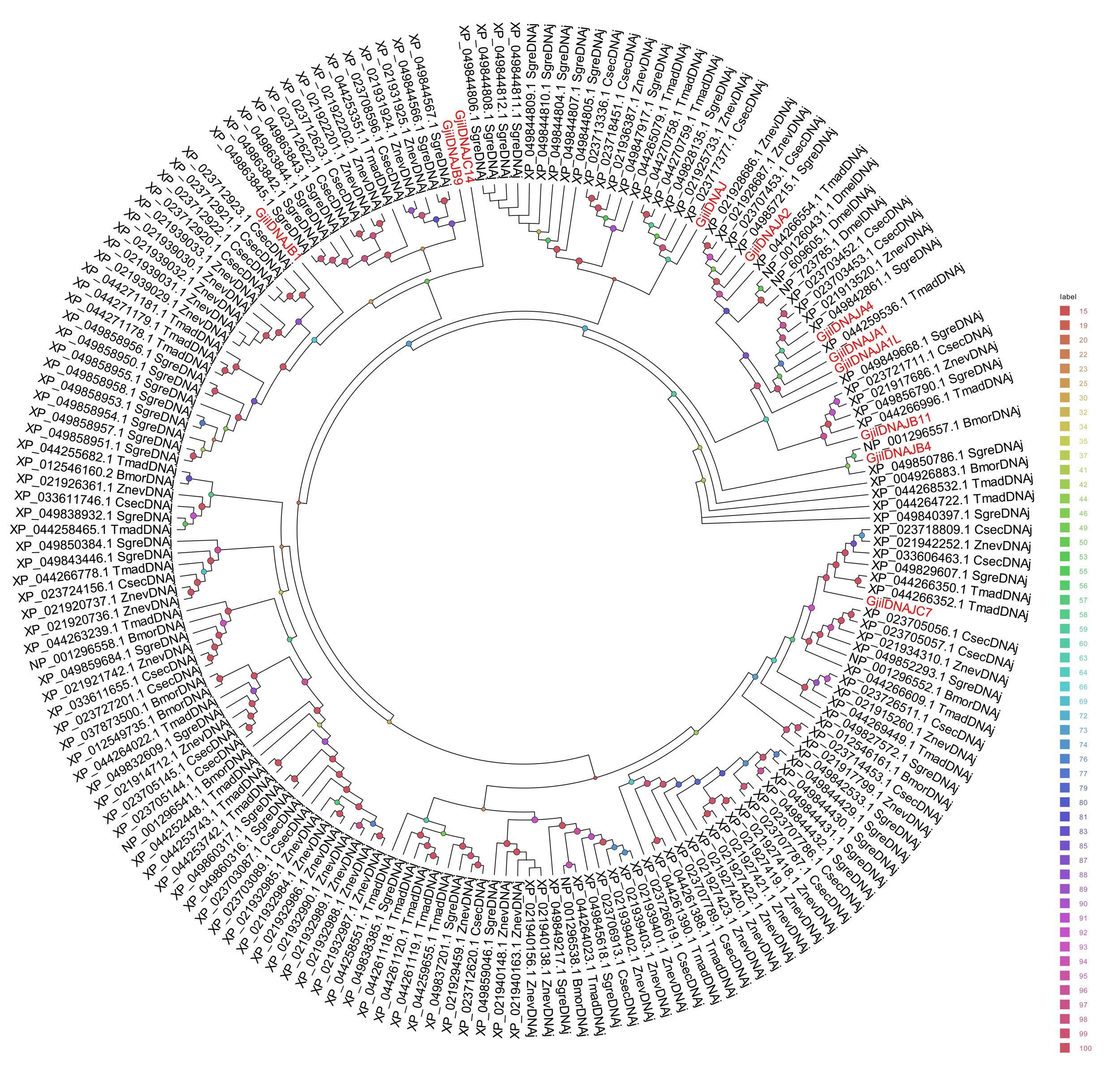


**Figure S18.** Homology analysis of DnaJs from *G. jilina* and other representative insect species. Among them, the homologous genes of other species are screened from the NCBI genome. Bmor: *Bombyx mori* (GCF_014905235.1), Csec: *Cryptotermes secundus* (GCF_002891405.2), Dmel: *Drosophila melanogaster* (GCF_000001215.4), Sgre: *Schistocerca gregaria* (GCF_023897955.1)*,* Tmad: *Tribolium madens* (GCF_015345945.1), Znev: *Zootermopsis nevadensis* (GCF_000696155.1).


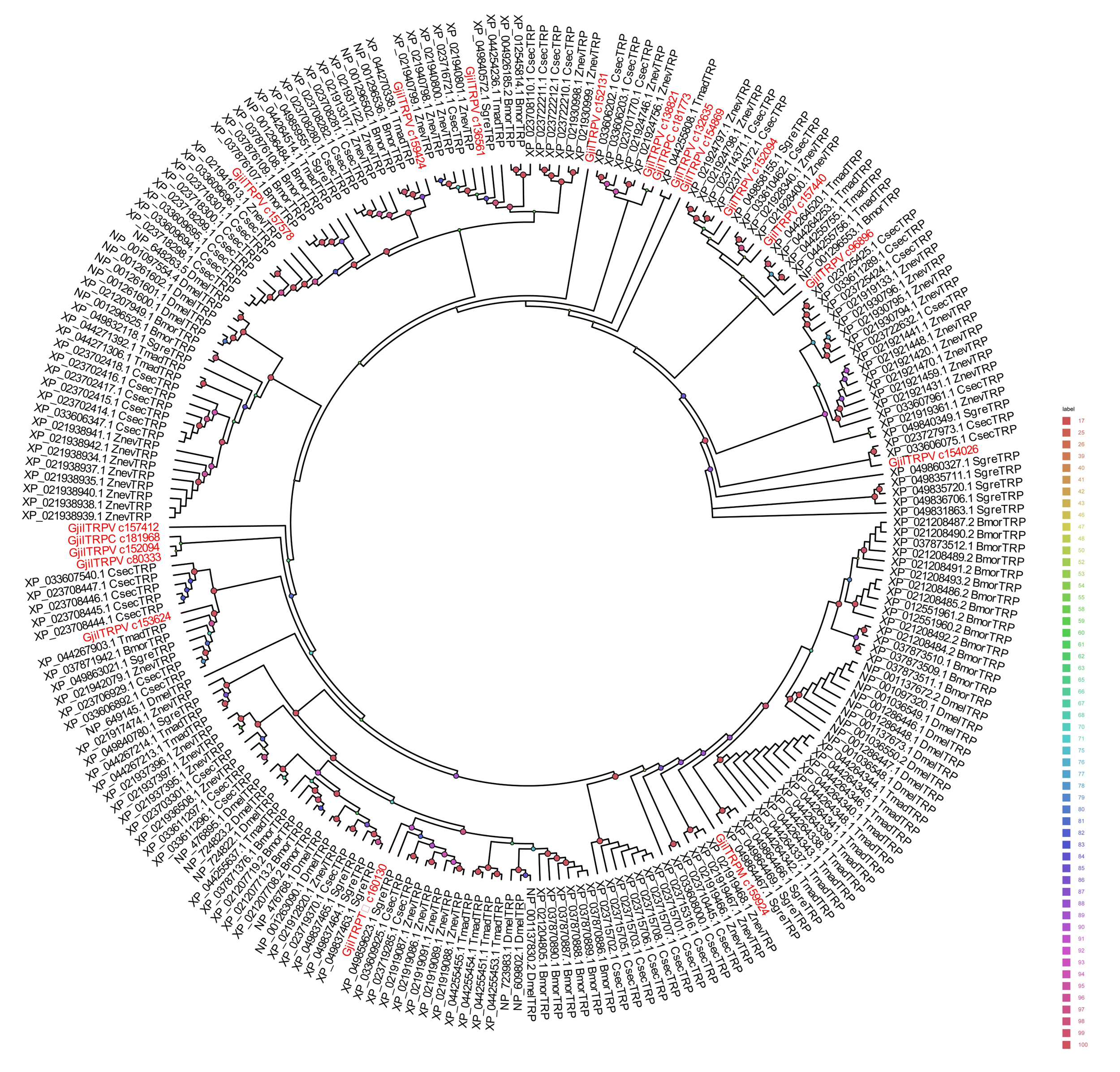


**Figure S19.** Homology analysis of TRPs from *G. jilina* and other representative insect species. Among them, the homologous genes of other species are screened from the NCBI genome. Bmor: *Bombyx mori* (GCF_014905235.1), Csec: *Cryptotermes secundus* (GCF_002891405.2), Dmel: *Drosophila melanogaster* (GCF_000001215.4), Sgre: *Schistocerca gregaria* (GCF_023897955.1)*,* Tmad: *Tribolium madens* (GCF_015345945.1), Znev: *Zootermopsis nevadensis* (GCF_000696155.1).


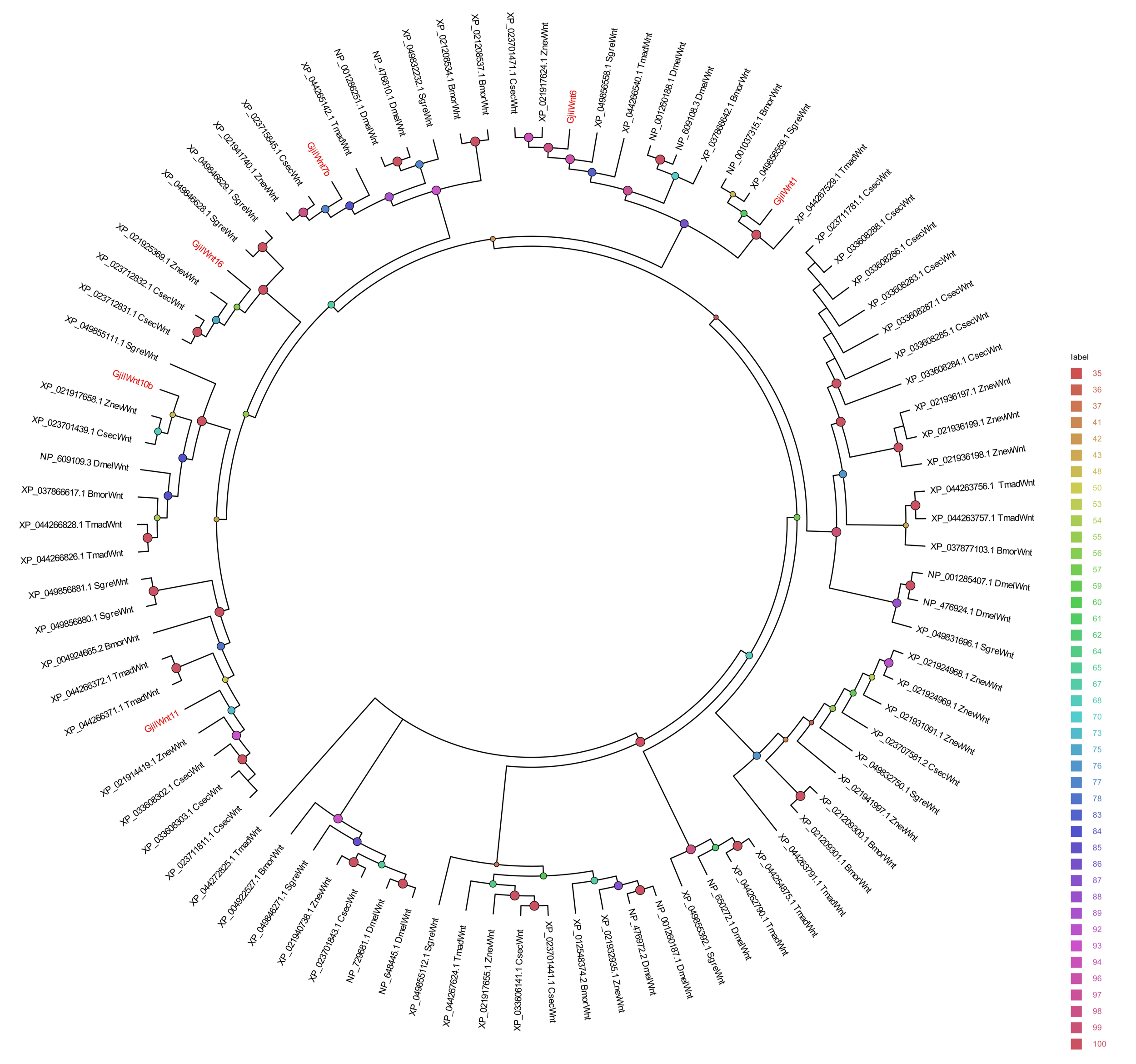


**Figure S20.** Homology analysis of wnts from *G. jilina* and other representative insect species. Among them, the homologous genes of other species are screened from the NCBI genome. Bmor: *Bombyx mori* (GCF_014905235.1), Csec: *Cryptotermes secundus* (GCF_002891405.2), Dmel: *Drosophila melanogaster* (GCF_000001215.4), Sgre: *Schistocerca gregaria* (GCF_023897955.1)*,* Tmad: *Tribolium madens* (GCF_015345945.1), Znev: *Zootermopsis nevadensis* (GCF_000696155.1).


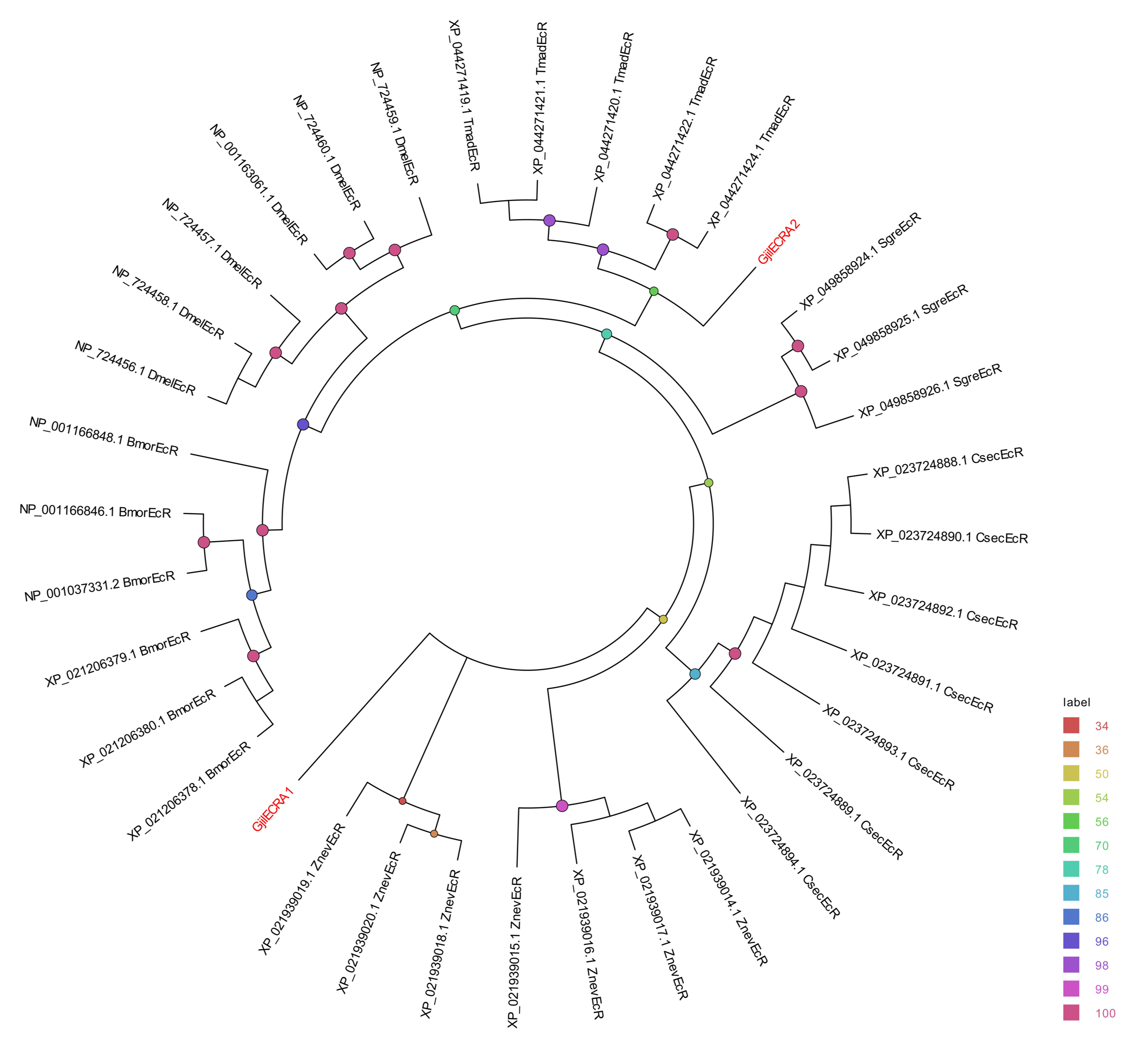


**Figure S21.** Homology analysis of EcRs from *G. jilina* and other representative insect species. Among them, the homologous genes of other species are screened from the NCBI genome. Bmor: *Bombyx mori* (GCF_014905235.1), Csec: *Cryptotermes secundus* (GCF_002891405.2), Dmel: *Drosophila melanogaster* (GCF_000001215.4), Sgre: *Schistocerca gregaria* (GCF_023897955.1)*,* Tmad: *Tribolium madens* (GCF_015345945.1), Znev: *Zootermopsis nevadensis* (GCF_000696155.1).


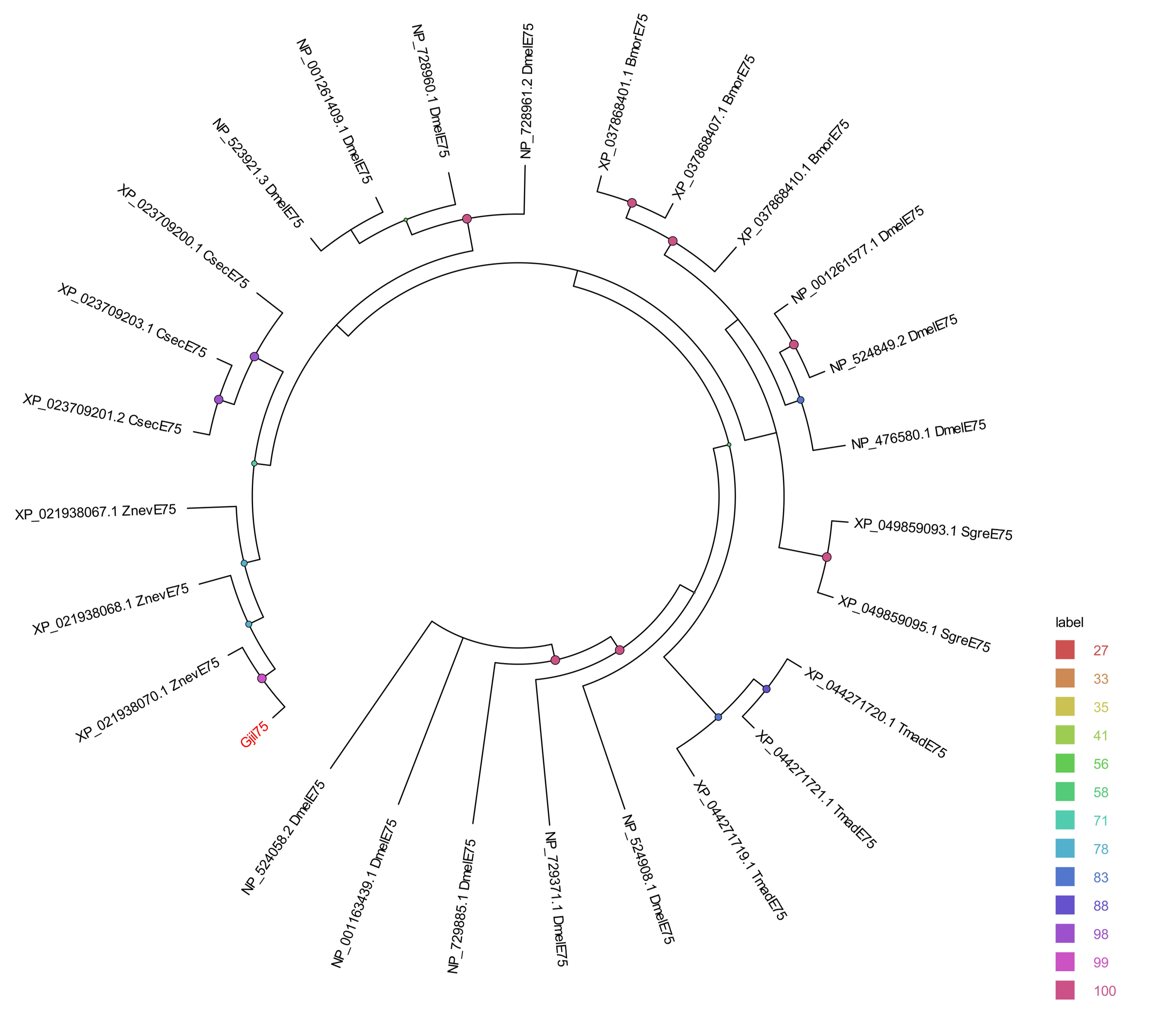


**Figure S22.** Homology analysis of E75s from *G. jilina* and other representative insect species. Among them, the homologous genes of other species are screened from the NCBI genome. Bmor: *Bombyx mori* (GCF_014905235.1), Csec: *Cryptotermes secundus* (GCF_002891405.2), Dmel: *Drosophila melanogaster* (GCF_000001215.4), Sgre: *Schistocerca gregaria* (GCF_023897955.1)*,* Tmad: *Tribolium madens* (GCF_015345945.1), Znev: *Zootermopsis nevadensis* (GCF_000696155.1).


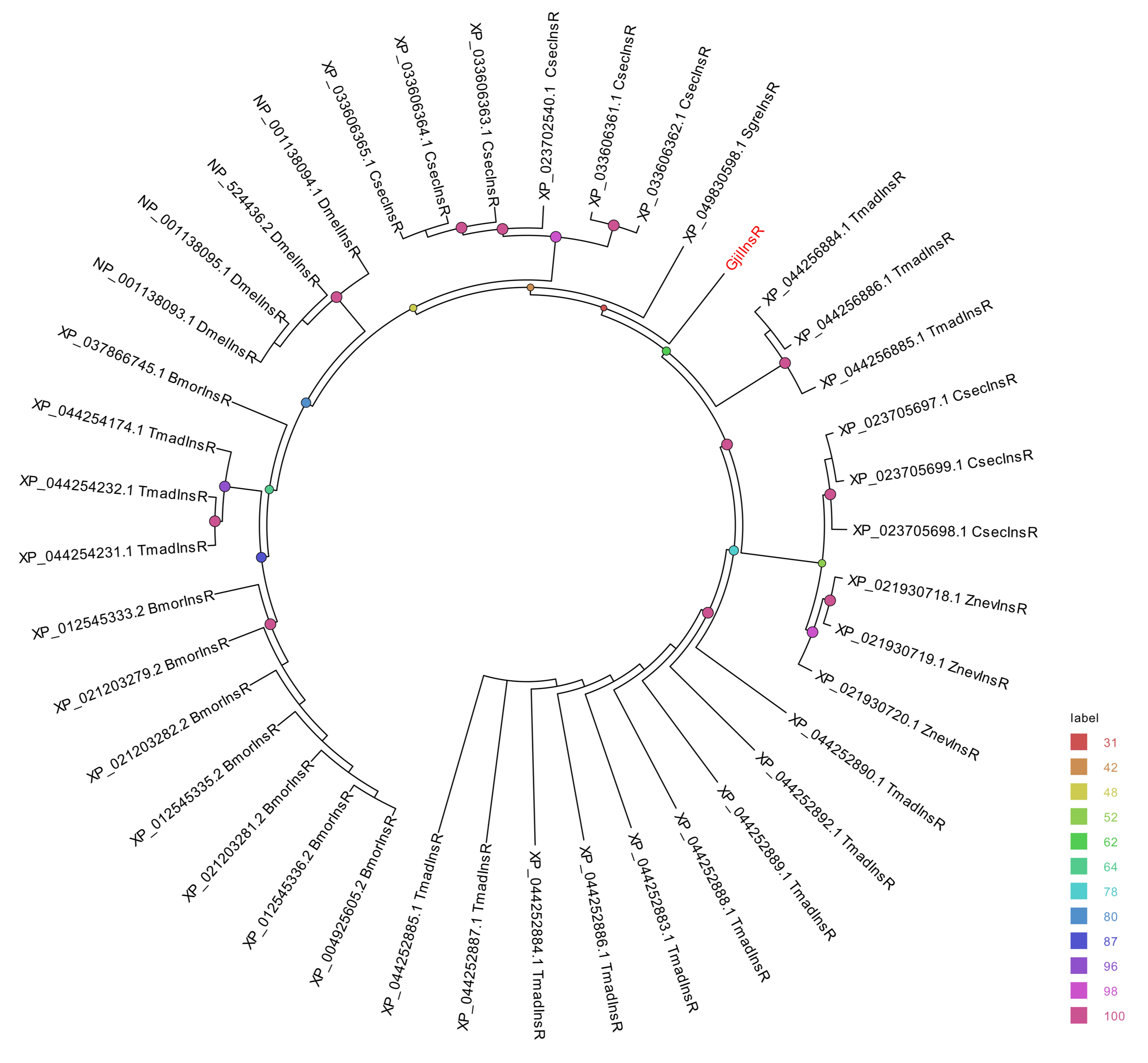


**Figure S23.** Homology analysis of InsRs from *G. jilina* and other representative insect species. Among them, the homologous genes of other species are screened from the NCBI genome. Bmor: *Bombyx mori* (GCF_014905235.1), Csec: *Cryptotermes secundus* (GCF_002891405.2), Dmel: *Drosophila melanogaster* (GCF_000001215.4), Sgre: *Schistocerca gregaria* (GCF_023897955.1)*,* Tmad: *Tribolium madens* (GCF_015345945.1), Znev: *Zootermopsis nevadensis* (GCF_000696155.1).


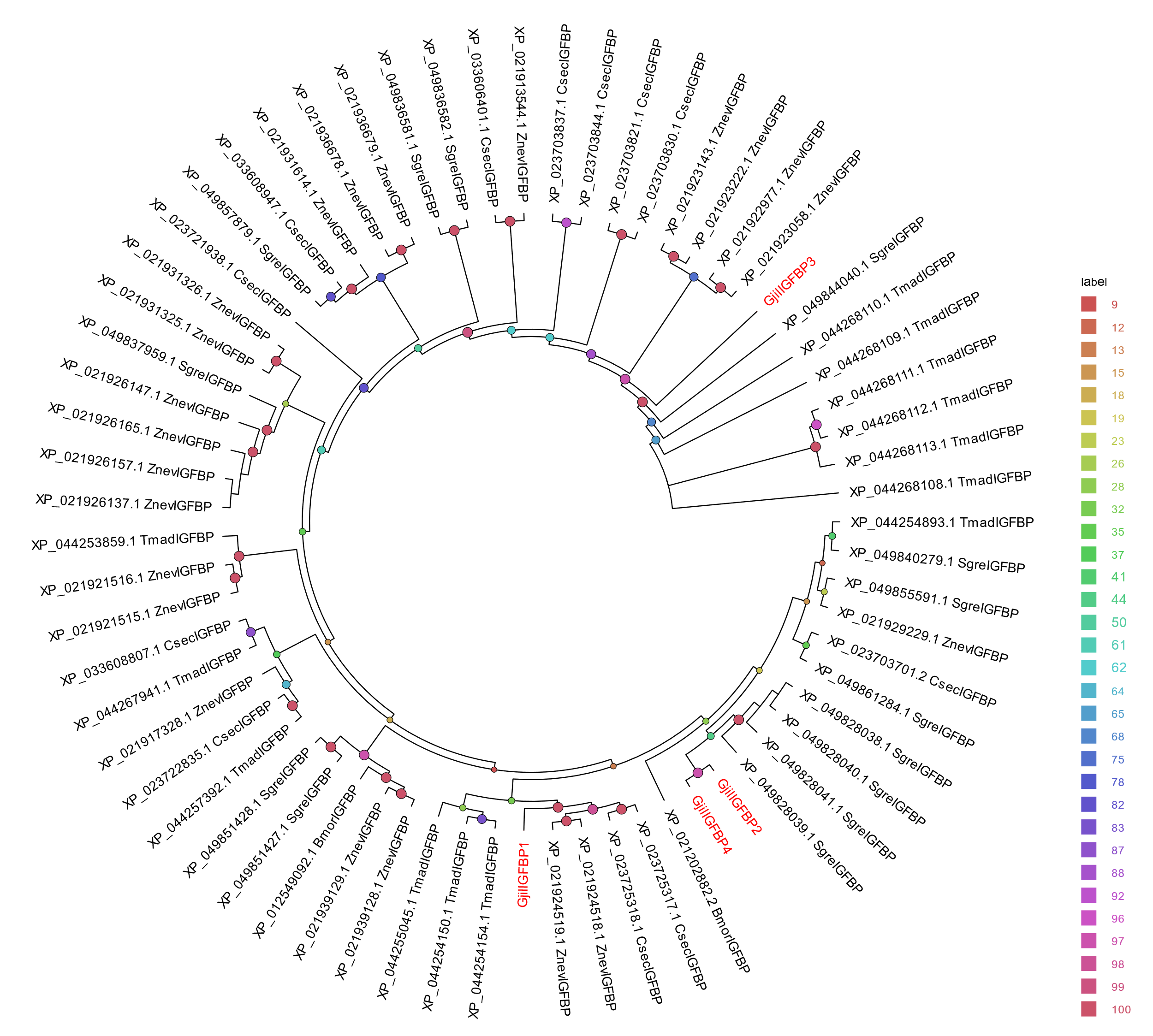


**Figure S24.** Homology analysis of IGFBPs from *G. jilina* and other representative insect species. Among them, the homologous genes of other species are screened from the NCBI genome. Bmor: *Bombyx mori* (GCF_014905235.1), Csec: *Cryptotermes secundus* (GCF_002891405.2), Dmel: *Drosophila melanogaster* (GCF_000001215.4), Sgre: *Schistocerca gregaria* (GCF_023897955.1)*,* Tmad: *Tribolium madens* (GCF_015345945.1), Znev: *Zootermopsis nevadensis* (GCF_000696155.1).


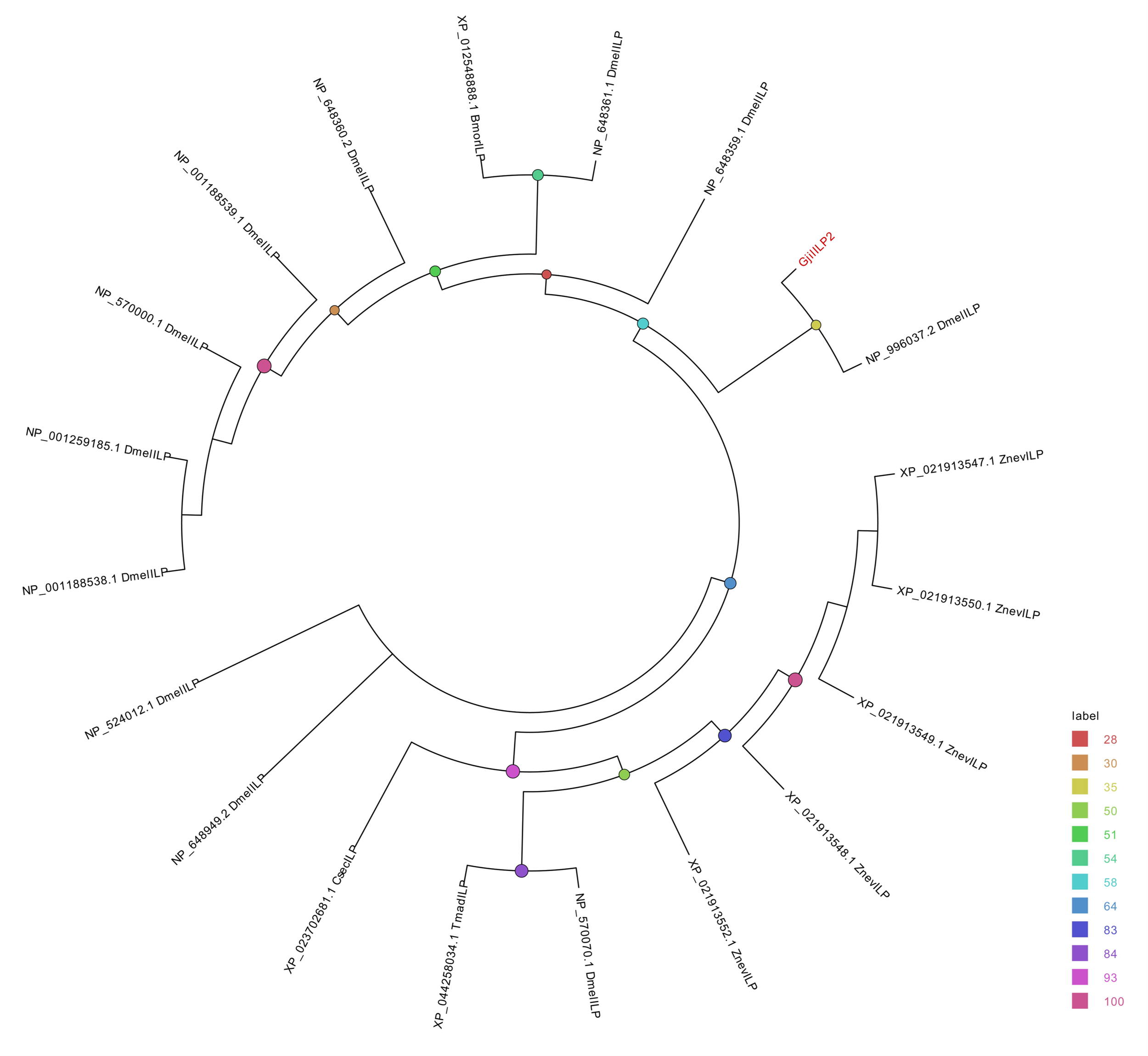


**Figure S25.** Homology analysis of ILPs from *G. jilina* and other representative insect species. Among them, the homologous genes of other species are screened from the NCBI genome. Bmor: *Bombyx mori* (GCF_014905235.1), Csec: *Cryptotermes secundus* (GCF_002891405.2), Dmel: *Drosophila melanogaster* (GCF_000001215.4), Tmad: *Tribolium madens* (GCF_015345945.1), Znev: *Zootermopsis nevadensis* (GCF_000696155.1).


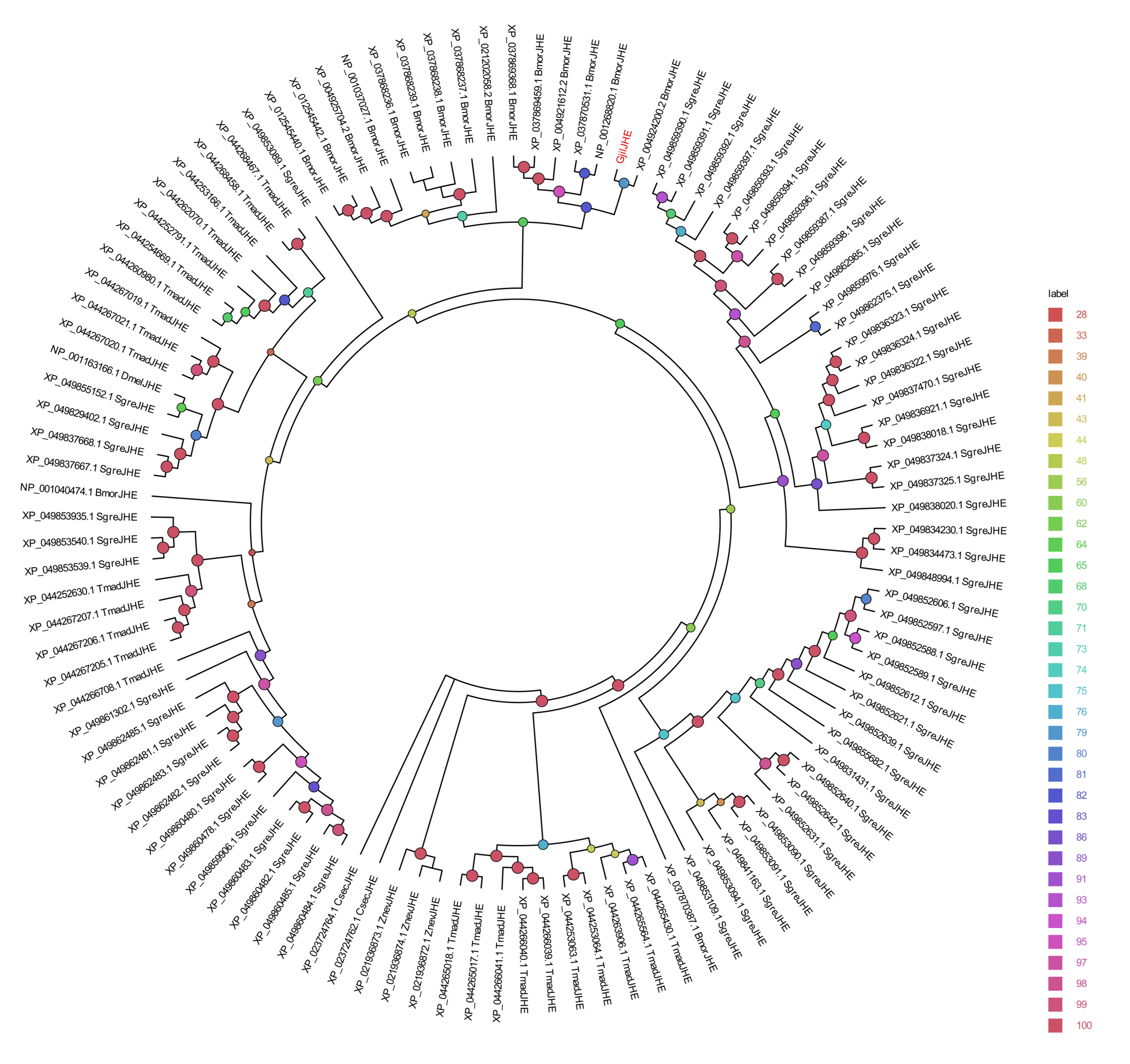


**Figure S26.** Homology analysis of JHEs from *G. jilina* and other representative insect species. Among them, the homologous genes of other species are screened from the NCBI genome. Bmor: *Bombyx mori* (GCF_014905235.1), Csec: *Cryptotermes secundus* (GCF_002891405.2), Dmel: *Drosophila melanogaster* (GCF_000001215.4), Sgre: *Schistocerca gregaria* (GCF_023897955.1)*,* Tmad: *Tribolium madens* (GCF_015345945.1), Znev: *Zootermopsis nevadensis* (GCF_000696155.1).


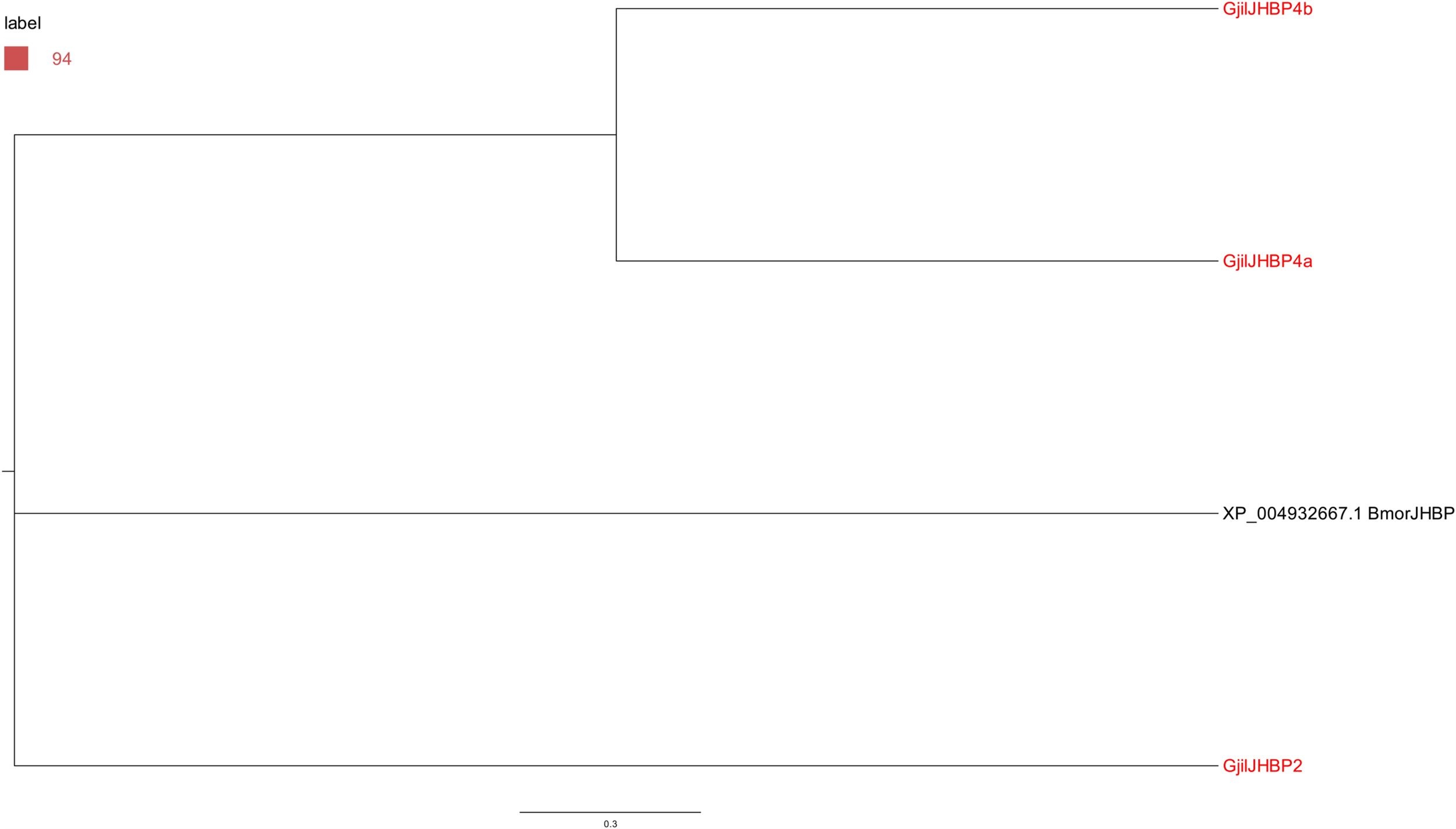


**Figure S27.** Homology analysis of JHBPs from *G. jilina* and other representative insect species. Among them, the homologous genes of other species are screened from the NCBI genome. Bmor: *Bombyx mori* (GCF_014905235.1).


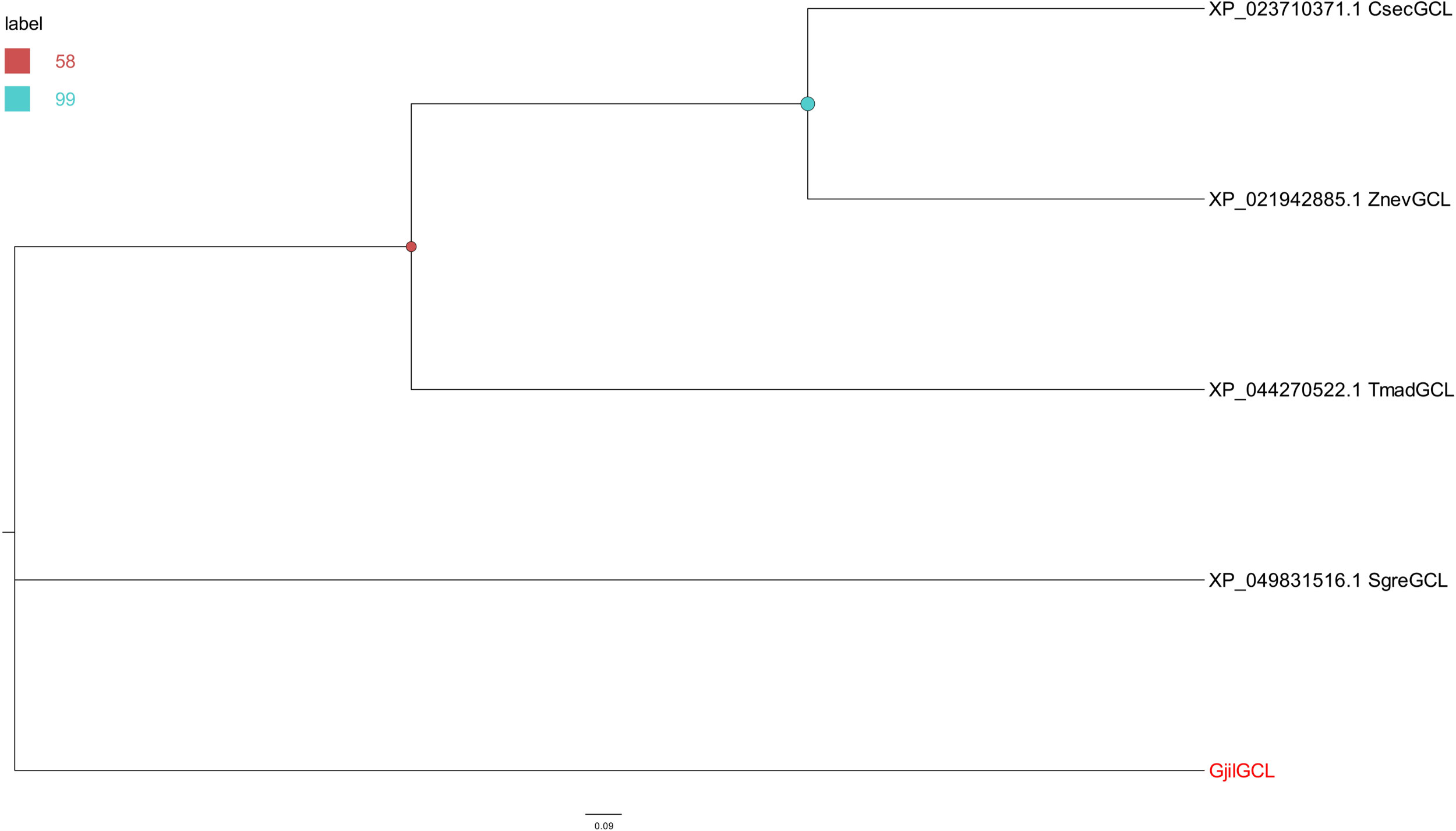


**Figure S28.** Homology analysis of GCLs from *G. jilina* and other representative insect species. Among them, the homologous genes of other species are screened from the NCBI genome. Csec: *Cryptotermes secundus* (GCF_002891405.2), Sgre: *Schistocerca gregaria* (GCF_023897955.1)*,* Tmad: *Tribolium madens* (GCF_015345945.1), Znev: *Zootermopsis nevadensis* (GCF_000696155.1).


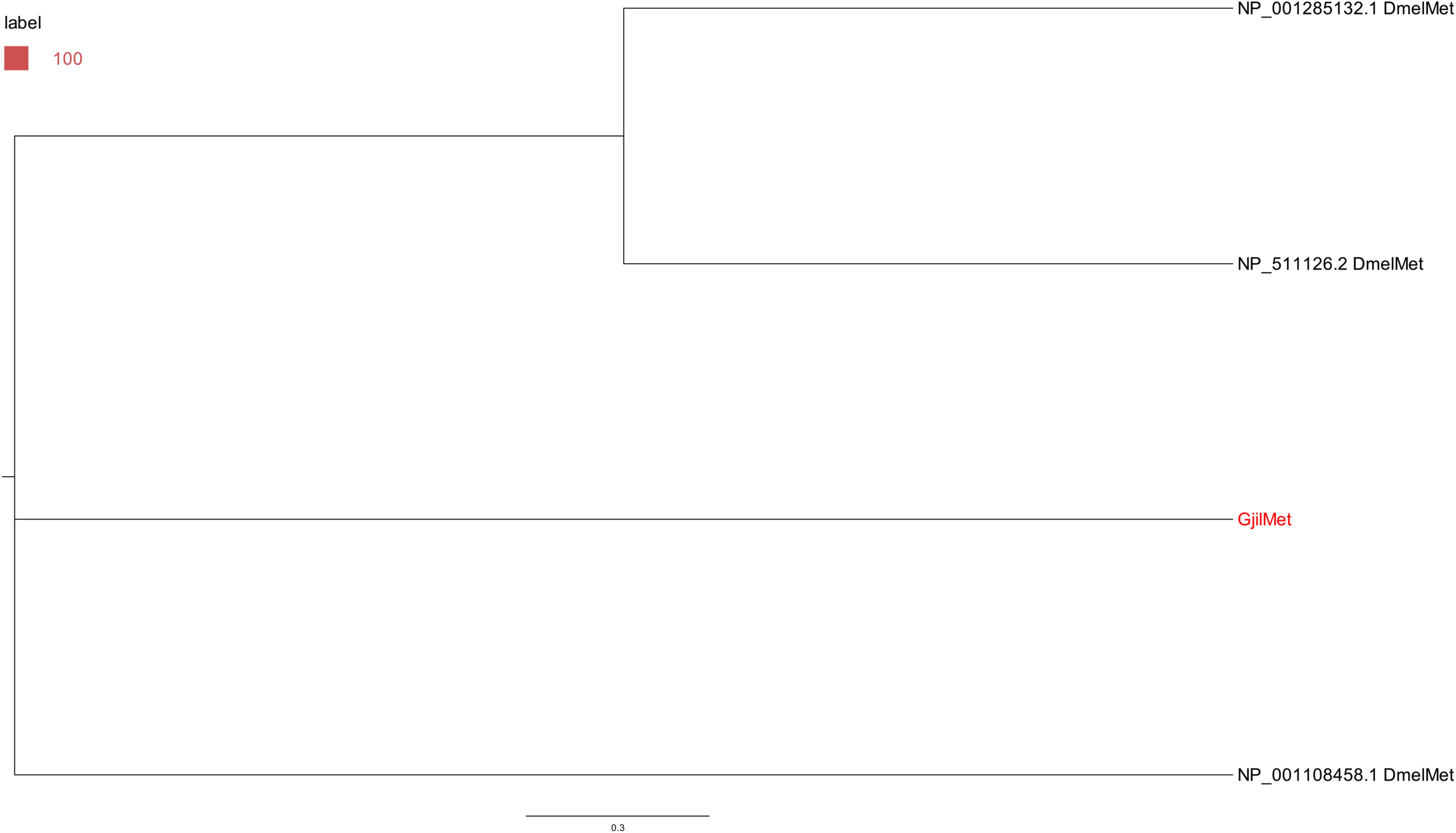


**Figure S29.** Homology analysis of Mets from *G. jilina* and other representative insect species. Among them, the homologous genes of other species are screened from the NCBI genome. Bmor: *Bombyx mori* (GCF_014905235.1), Dmel: *Drosophila melanogaster* (GCF_000001215.4).


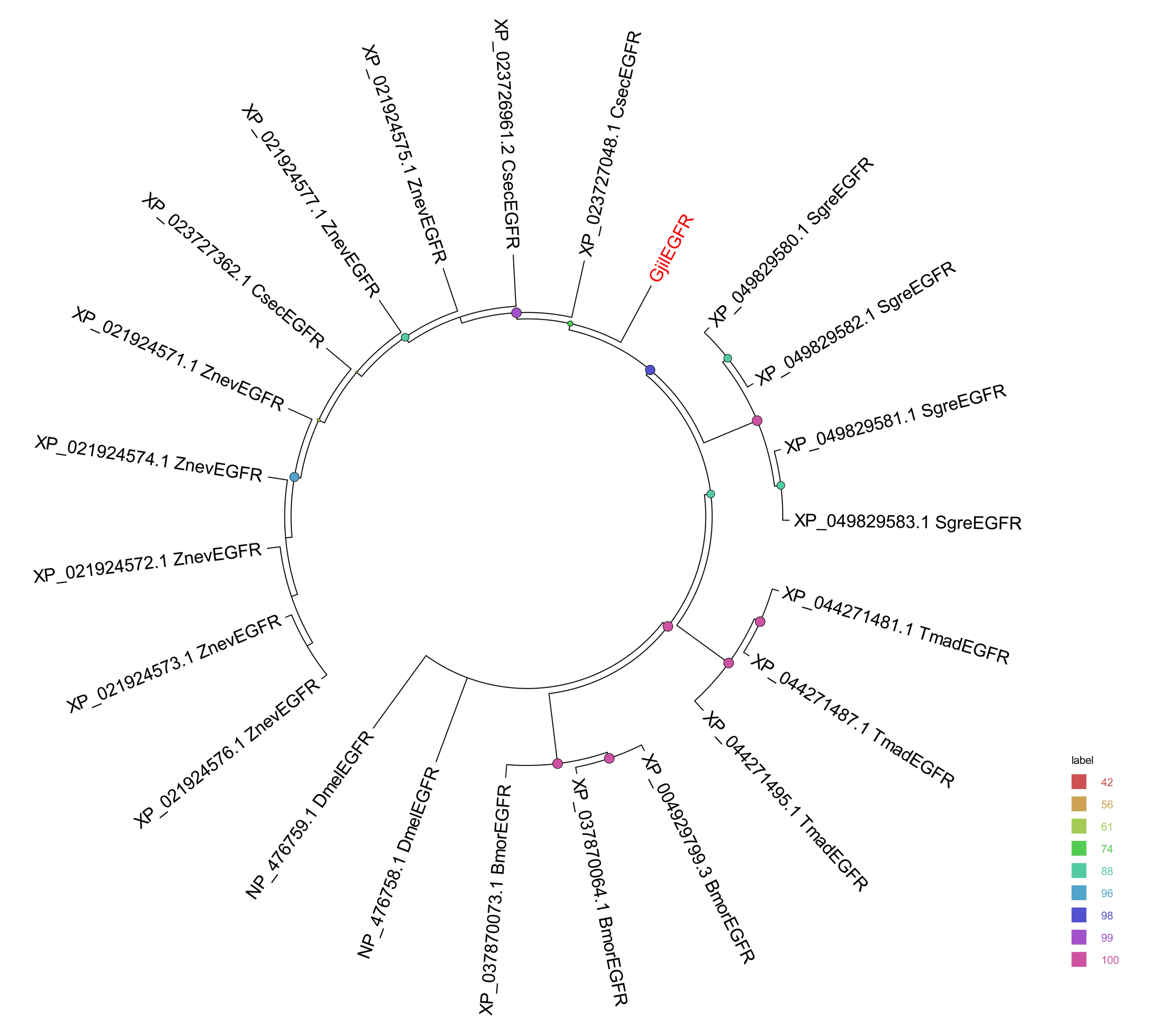


**Figure S30.** Homology analysis of EGFRs from *G. jilina* and other representative insect species. Among them, the homologous genes of other species are screened from the NCBI genome. Bmor: *Bombyx mori* (GCF_014905235.1), Csec: *Cryptotermes secundus* (GCF_002891405.2), Dmel: *Drosophila melanogaster* (GCF_000001215.4), Sgre: *Schistocerca gregaria* (GCF_023897955.1)*,* Tmad: *Tribolium madens* (GCF_015345945.1), Znev: *Zootermopsis nevadensis* (GCF_000696155.1).


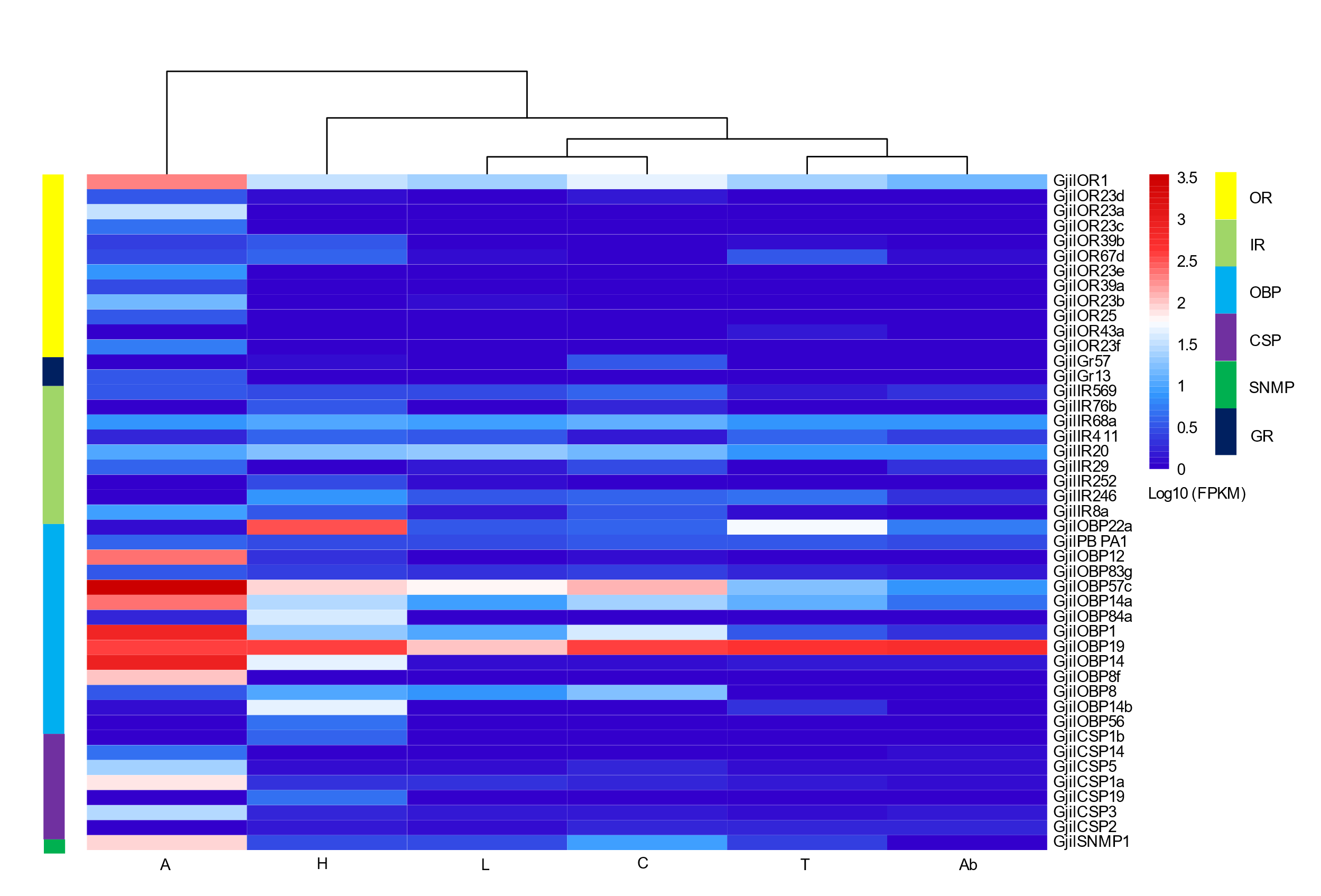


**Figure S31.** Expression patterns of chemosensory genes in *G. jilina*. Each row and column represent a gene and specimen, respectively. The specimens are (A) antenna, (H) heads, (T) thoraxes, (L) legs, (Ab) abdomens, and (C), tails. The color gradient from red to green represents log10 (FPKM) values from large to small.


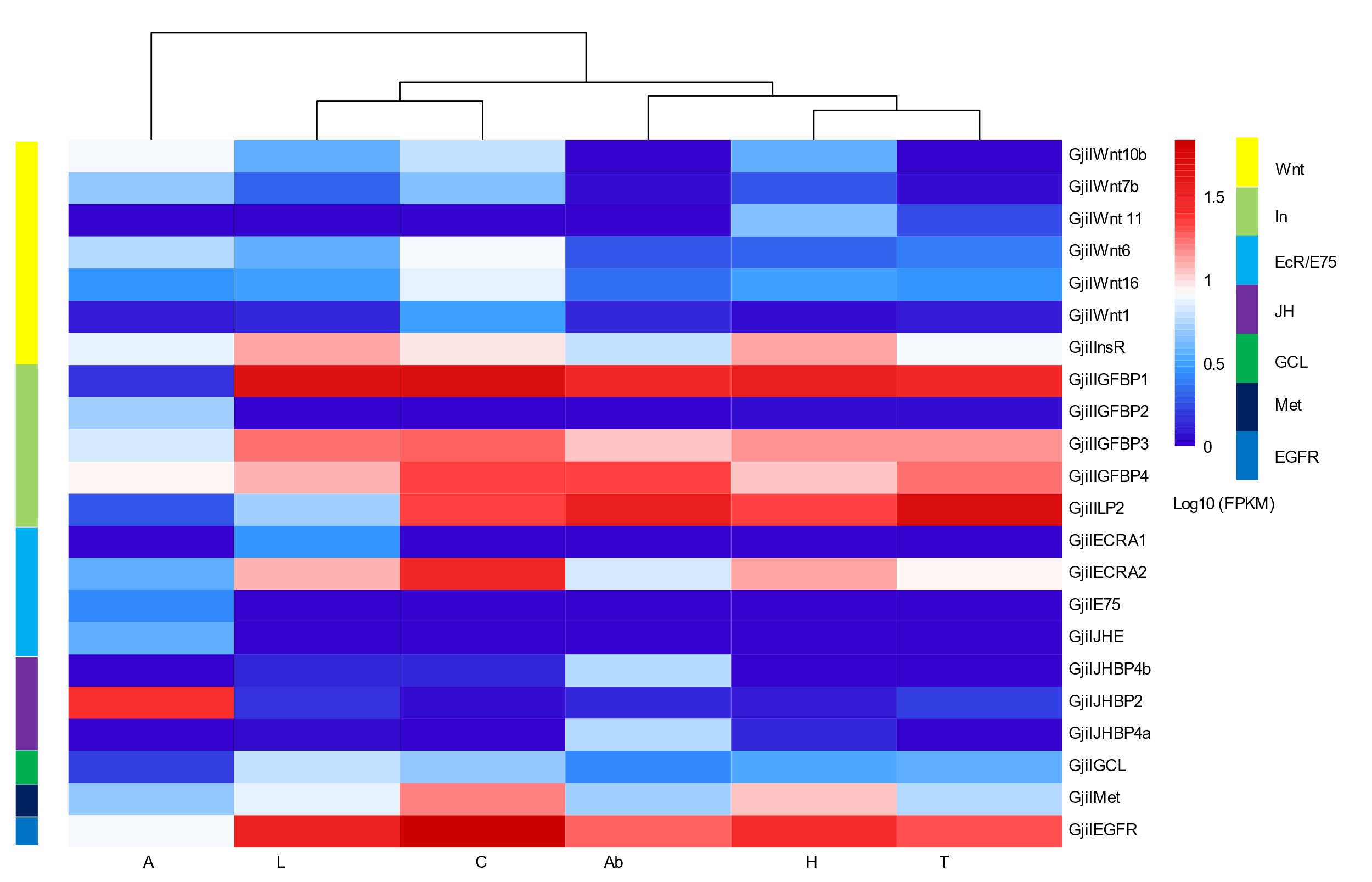


**Figure S32.** Expression patterns of reproduction-related genes in *G. jilina*. Each row and column represent a gene and specimen, respectively. The specimens are (A) antenna, (H) heads, (T) thoraxes, (L) legs, (Ab) abdomens, and (C), tails. The color gradient from red to green represents log10 (FPKM) values from large to small.


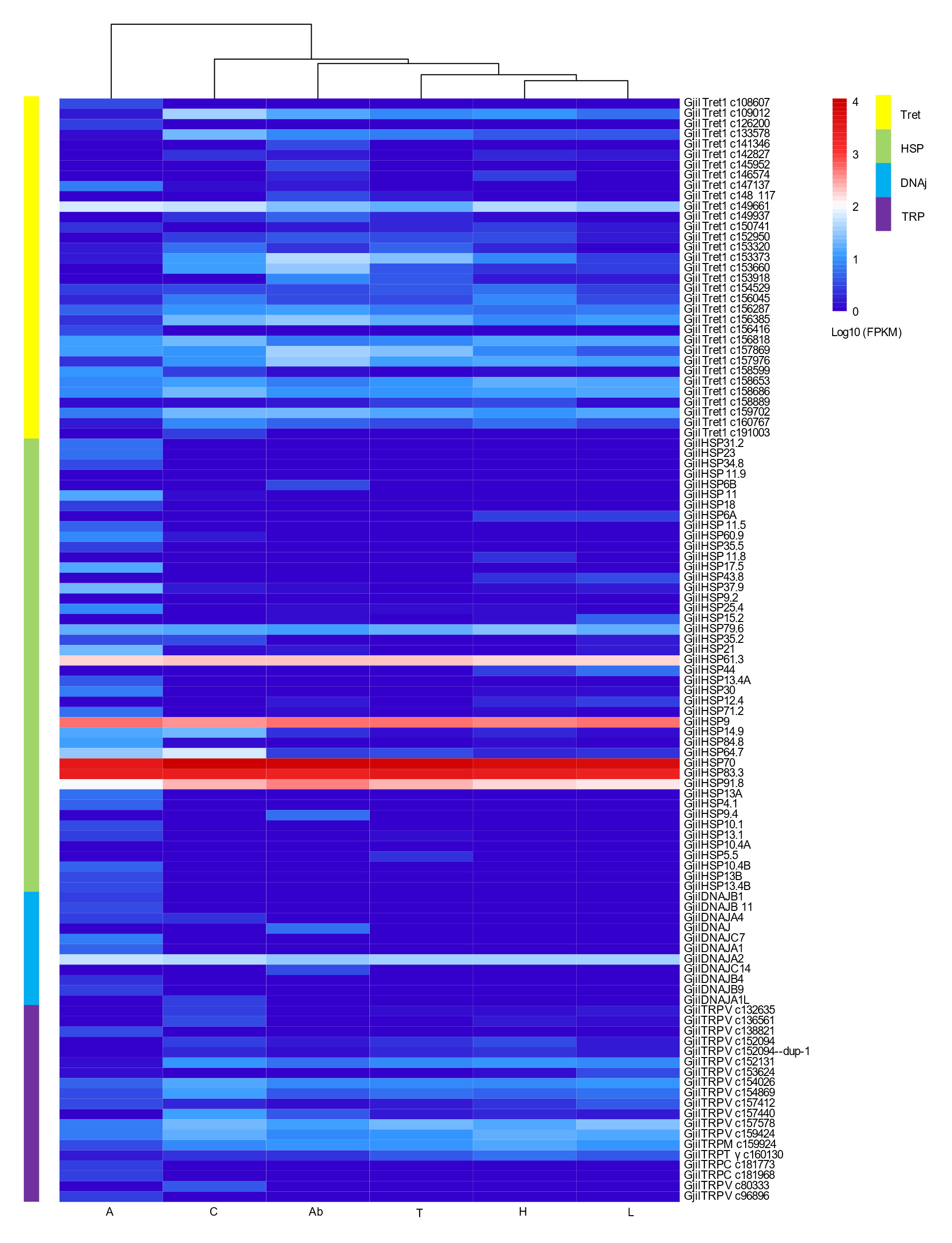


**Figure S33.** Expression patterns of temperature adaptation-related genes in *G. jilina*. Each row represents a gene, and each column represents a specimen. The specimens are, (A), antenna; (H), heads; (T), thoraxes; (L), legs; (Ab), abdomens; (C), tails. The color ranges from red to green, representing log10 (FPKM) from large to small.


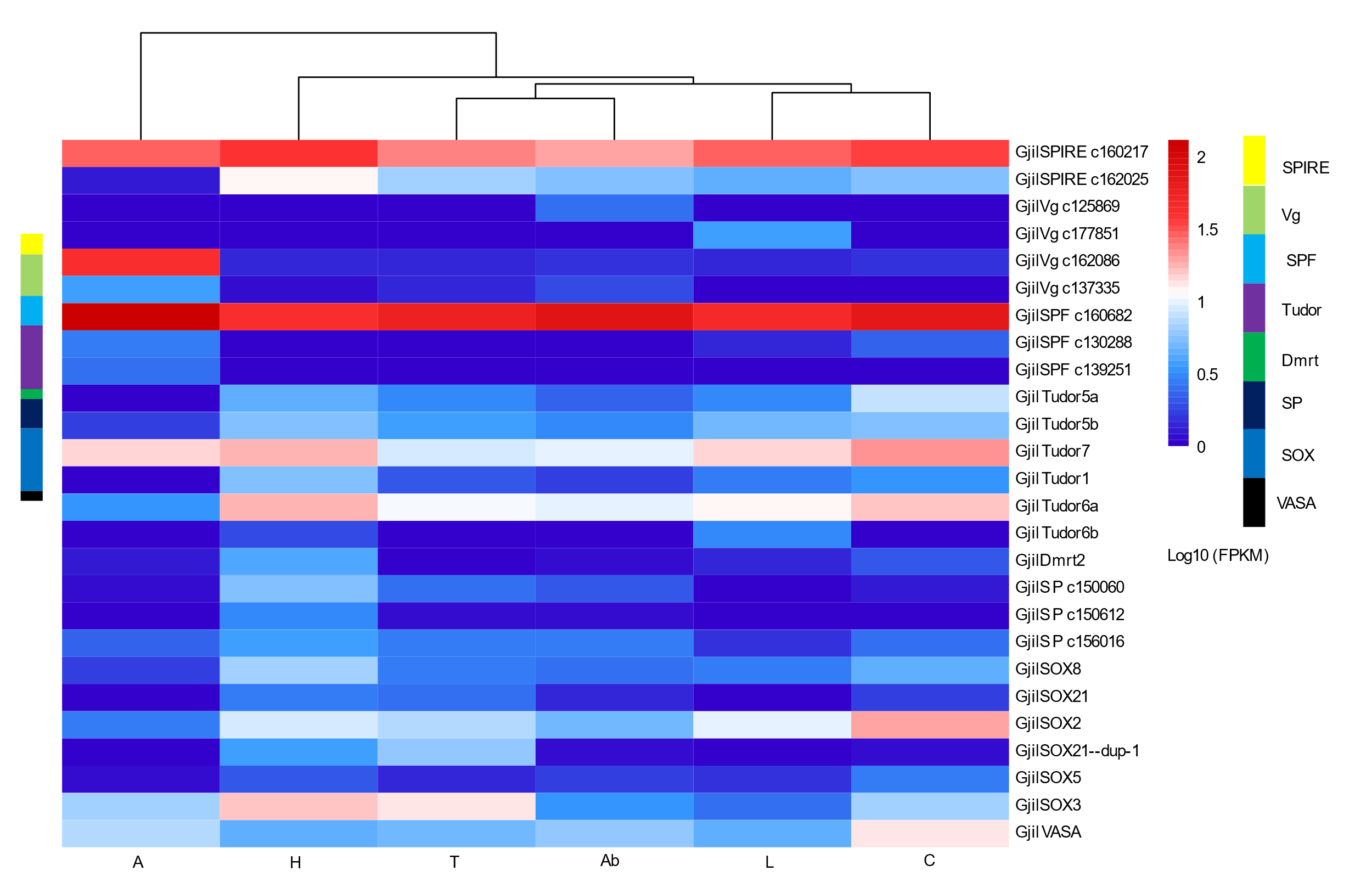


**Figure S34.** Expression patterns of winged morph differentiation-related genes in *G. jilina.* Each row represents a gene, and each column represents a specimen. The specimens are, (A), antenna; (H), heads; (T), thoraxes; (L), legs; (Ab), abdomens; (C), tails. The color ranges from red to green, representing log10 (FPKM) from large to small.

**Table S1**. Summary of *G. jilina* Transcriptome

| **Length range** | **Transcript** | **Unigene** |
| --- | --- | --- |
| 300-500bp | 24365（26.51%） | 19497（40.56%） |
| 500-1000bp | 22405（24.37%） | 12347（25.68%） |
| 1000-2000bp | 20144（21.91） | 7393（15.38%） |
| ＞2000bp | 25007（27.20%） | 8834（18.38%） |
| Total Number | 91921 | 48071 |
| Total Length | 150136619bp | 60892310bp |
| N50 Length | 2760bp | 2431bp |
| Mean Length | 163332bp | 126672bp |

**Table S2.** Annotation of *G. jilina* with BLAST

| Anno_Database | Annotated_Number | 300<=length<1000 | length>=1000 |
| --- | --- | --- | --- |
| COG_Annotation | 5007 | 2330 | 2677 |
| GO_Annotation | 15711 | 7399 | 8310 |
| KEGG_Annotation | 14621 | 6734 | 7887 |
| KOG_Annotation | 13092 | 6493 | 6599 |
| Pfam_Annotation | 15220 | 6886 | 8334 |
| Swissprot_Annotation | 9320 | 3974 | 5346 |
| TrEMBL_Annotation | 17958 | 8102 | 9856 |
| eggNOG_Annotation | 14740 | 6454 | 8286 |
| nr_Annotation | 17928 | 8049 | 9879 |
| All_Annotated | 20517 | 10250 | 1026 |

| **Table S3**. List of OR genes in *G. jilina* | | | |  |  | | |  |  | | |  |
| --- | --- | --- | --- | --- | --- | --- | --- | --- | --- | --- | --- | --- |
| **Gene name** | **Unigene ID** | **Unigene** | **BLASTx best hit** | **Query cover** | **E** | **Ident** | **The Length of** | | | **ID** |  |  |
|  |  | **ORF (aa）** |  |  |  |  | **"Best hit" (aa)** | | |  |  |  |
| *GjilOR1* | c153606.graph_c1 | 472 | odorant receptor coreceptor [Zootermopsis nevadensis] | 100.00% | 0 | 68.50% | 472 | | | [XP_021933609.1](https://www.ncbi.nlm.nih.gov/protein/XP_021933609.1?report=genbank&log$=prottop&blast_rank=6&RID=4MPE9FM2016) |  |  |
| *GjilOR23d* | c149468.graph_c1 | 452 | Odorant receptor 51a [Blattella germanica] | 81.00% | 2.00E-48 | 28.98% | 464 | | | [PSN35482.1](https://www.ncbi.nlm.nih.gov/protein/PSN35482.1?report=genbank&log$=prottop&blast_rank=3&RID=4MPEV1YD013) |  |  |
| *GjilOR23a* | c160303.graph_c0 | 343 | odorant receptor Or2-like [Cryptotermes secundus] | 96.00% | 8.00E-39 | 30.26% | 462 | | | [XP_033611726.1](https://www.ncbi.nlm.nih.gov/protein/XP_033611726.1?report=genbank&log$=prottop&blast_rank=2&RID=4MPJXE86016) |  |  |
| *GjilOR23c* | c158420.graph_c0 | 330 | odorant receptor coreceptor-like [Zootermopsis nevadensis] | 86.00% | 1.00E-40 | 30.91% | 397 | | | [XP_021929942.1](https://www.ncbi.nlm.nih.gov/protein/XP_021929942.1?report=genbank&log$=prottop&blast_rank=1&RID=4MPKA2CE013) |  |  |
| *GjilOR39b* | c150841.graph_c1 | 286 | odorant receptor 28 [Locusta migratoria] | 98.00% | 6.00E-44 | 32.53% | 307 | | | [ALD51442.1](https://www.ncbi.nlm.nih.gov/protein/ALD51442.1?report=genbank&log$=prottop&blast_rank=2&RID=4MPKPR91013) |  |  |
| *GjilOR67d* | c156314.graph_c0 | 222 | odorant receptor 3 [Subpsaltria yangi] | 90.00% | 5.00E-24 | 32.37% | 407 | | | [AXY87890.1](https://www.ncbi.nlm.nih.gov/protein/AXY87890.1?report=genbank&log$=prottop&blast_rank=2&RID=4MRGTW0B013) |  |  |
| *GjilOR23e* | c152606.graph_c2 | 152 | Odorant receptor 53 [Blattella germanica] | 97.00% | 5.00E-32 | 44.00% | 448 | | | [PSN30186.1](https://www.ncbi.nlm.nih.gov/protein/PSN30186.1?report=genbank&log$=prottop&blast_rank=1&RID=4MRH22BC013) |  |  |
| *GjilOR39a* | c82394.graph_c0 | 340 | odorant receptor 13a [Cryptotermes secundus] | 96.00% | 2.00E-19 | 28.82% | 476 | | | [XP_023724790.1](https://www.ncbi.nlm.nih.gov/protein/XP_023724790.1?report=genbank&log$=prottop&blast_rank=10&RID=4Z528ND4013) |  |  |
| *GjilOR23b* | c157623.graph_c1 | 46 | [Odorant receptor Or2 [Blattella germanica]](https://blast.ncbi.nlm.nih.gov/Blast.cgi#alnHdr_PSN55029) | 89.00% | 4.00E-10 | 60.98% | 67 | | | [PSN55029.1](https://www.ncbi.nlm.nih.gov/protein/PSN55029.1?report=genbank&log$=prottop&blast_rank=1&RID=4Z53G5KF013) |  |  |
| *GjilOR25* | c141175.graph_c0 | 106 | PREDICTED: gustatory and odorant receptor 21a-like [Musca domestica] | 74.00% | 1.00E-08 | 44.30% | 453 | | | [XP_005189990.1](https://www.ncbi.nlm.nih.gov/protein/XP_005189990.1?report=genbank&log$=prottop&blast_rank=1&RID=4Z54BS5T013) |  |  |
| *GjilOR43a* | c139994.graph_c0 | 45 | odorant receptor 13a-like [Athalia rosae] | 100.00% | 0.03 | 37.78% | 442 | | | [XP_012260422.1](https://www.ncbi.nlm.nih.gov/protein/XP_012260422.1?report=genbank&log$=prottop&blast_rank=1&RID=4Z55HCWU016) |  |  |
| *GjilOR23f* | c107046.graph_c0 | 277 | Odorant receptor 53 [Blattella germanica] | 53.00% | 1.00E-28 | 42.18% | 448 | | | [PSN30186.1](https://www.ncbi.nlm.nih.gov/protein/PSN30186.1?report=genbank&log$=prottop&blast_rank=2&RID=4Z57YYU0016) |  |  |
| **Table S4.** List of GR genes in *G. jilina* | | | |  |  |  |  | | |  |  |  |
| **Gene name** | **Unigene ID** | **Unigene ORF (aa)** | **BLASTx best hit** | **Query cover** | **E** | **Ident** | **The Length of "Best hit" (aa)** | | | **ID** |  |  |
| *GjilGr57* | c123811.graph_c1 | 121 | Gustatory receptor 35 [Frankliniella occidentalis] | 69.00% | 3.00E-12 | 42.35% | 456 | | | [KAE8747292.1](https://www.ncbi.nlm.nih.gov/protein/KAE8747292.1?report=genbank&log$=prottop&blast_rank=2&RID=4RDD78H7013) |  |  |
| *GjilGr13* | c141175.graph_c0 | 106 | gustatory and odorant receptor 22-like [Harmonia axyridis] | 74.00% | 2E-08 | 40.51% | 437 | | | [XP_045460911.1](https://www.ncbi.nlm.nih.gov/protein/XP_045460911.1?report=genbank&log$=prottop&blast_rank=2&RID=4RDS0A8501R) |  |  |
| **Table S5.** List of IR genes in *G. jilina* | | | |  |  |  |  | | |  |  |  |
| **Gene name** | **Unigene ID** | **Unigene** | **BLASTx best hit** | **Query cover** | **E** | **Ident** | **The Length of "Best hit" (aa)** | | | **ID** |  |  |
|  |  | **ORF (aa)** |  |  |  |  |  |  |  |  |  |  |
| *GjilIR569* | c154680.graph_c1 | 134 | Ionotropic receptor 93a-like  [Ctenocephalides felis] | 52.00% | 1.00E-13 | 66.20% | 887 | | | [XP_026467502.1](https://www.ncbi.nlm.nih.gov/protein/XP_026467502.1?report=genbank&log$=prottop&blast_rank=1&RID=4RB2V5XP013) |  |  |
| *GjilIR76b* | c156448.graph_c0 | 555 | Ionotropic receptor 76b [Blattella germanica] | 99.00% | 2.00E-170 | 51.33% | 538 | | | [PSN56156.1](https://www.ncbi.nlm.nih.gov/protein/PSN56156.1?report=genbank&log$=prottop&blast_rank=2&RID=4RB0E4G5013) |  |  |
| *GjilIR68a* | c160083.graph_c1 | 431 | Ionotropic receptor 68a [Blattella germanica] | 96.00% | 5.00E-166 | 59.82% | 679 | | | [PSN33061.1](https://www.ncbi.nlm.nih.gov/protein/PSN33061.1?report=genbank&log$=prottop&blast_rank=6&RID=4RANGADX016) |  |  |
| *GjilIR411* | c151922.graph_c0 | 286 | Ionotropic receptor 41a3 [Blattella germanica] | 84.00% | 4.00E-48 | 39.51% | 615 | | | [PSN50071.1](https://www.ncbi.nlm.nih.gov/protein/PSN50071.1?report=genbank&log$=prottop&blast_rank=1&RID=4RBEYJZ0016) |  |  |
| *GjilIR20* | c160667.graph_c2 | 181 | Ionotropic receptor 173 [Blattella germanica] | 90.00% | 9.00E-22 | 33.33% | 612 | | | [PSN36467.1](https://www.ncbi.nlm.nih.gov/protein/PSN36467.1?report=genbank&log$=prottop&blast_rank=7&RID=4RA1C026013) |  |  |
| *GjilIR29* | c154685.graph_c0 | 297 | Ionotropic receptor 124 [Blattella germanica] | 97.00% | 3.00E-54 | 38.83% | 663 | | | [PSN40369.1](https://www.ncbi.nlm.nih.gov/protein/PSN40369.1?report=genbank&log$=prottop&blast_rank=1&RID=4RBH7SXJ013) |  |  |
| *GjilIR252* | c139907.graph_c1 | 57 | hypothetical protein JTE90_003218 [Oedothorax gibbosus] | 82.00% | 2E-07 | 51.06% | 828 | | | [KAG8185679.1](https://www.ncbi.nlm.nih.gov/protein/KAG8185679.1?report=genbank&log$=prottop&blast_rank=1&RID=4RB77CA8016) |  |  |
| *GjilIR246* | c148510.graph_c0 | 283 | alpha-ketoglutarate-dependent sulfonate dioxygenase-like isoform X1 [Schistocerca piceifrons] | 100.00% | 2.00E-153 | 73.85% | 282 | | | [XP_047114767.1](https://www.ncbi.nlm.nih.gov/protein/XP_047114767.1?report=genbank&log$=prottop&blast_rank=3&RID=4RATYDMN013) |  |  |
| *GjilIR8a* | c152744.graph_c0 | 478 | Ionotropic receptor 8a [Blattella germanica] | 99.00% | 0 | 90.30% | 874 | | | [PSN54615.1](https://www.ncbi.nlm.nih.gov/protein/PSN54615.1?report=genbank&log$=prottop&blast_rank=1&RID=N2E9SH3G016) |  |  |
| **Table S6.** List of OBP genes in *G. jilina* | | | |  |  |  |  | | |  |  |  |
| **Gene name** | **Unigene ID** | **Unigene** | **BLASTx best hit** | **Query cover** | **E** | **Ident** | **The Length of "Best hit" (aa)** | | | **ID** |  |  |
|  |  | **ORF (aa)** |  |  |  |  |  |  |  |  |  |  |
| *GjilOBP22a* | c159245.graph_c8 | 289 | protein takeout [Cryptotermes secundus] | 83.00% | 8.00E-71 | 45.23% | 255 | | | [XP_023723281.1](https://www.ncbi.nlm.nih.gov/protein/XP_023723281.1?report=genbank&log$=prottop&blast_rank=2&RID=4R7TYMMJ013) |  |  |
| *GjilPBPA1* | c157672.graph_c0 | 162 | [pheromone binding protein [Mythimna separata]](https://blast.ncbi.nlm.nih.gov/Blast.cgi#alnHdr_BAG71416) | 89.00% | 4.00E-104 | 100.00% | 170 | | | [BAG71416.1](https://www.ncbi.nlm.nih.gov/protein/BAG71416.1?report=genbank&log$=prottop&blast_rank=1&RID=4R5PV8XZ013) |  |  |
| *GjilOBP12* | c141716.graph_c0 | 248 | odorant-binding protein 47 [Matsumurasca onukii] | 37.00% | 2.00E-31 | 52.13% | 129 | | | [AWC68016.1](https://www.ncbi.nlm.nih.gov/protein/AWC68016.1?report=genbank&log$=prottop&blast_rank=2&RID=4R76G3GJ016) |  |  |
| *GjilOBP83g* | c150885.graph_c0 | 148 | odorant-binding protein 19 [Mythimna separata] | 72.00% | 3.00E-66 | 97.20% | 107 | | | [AWT22235.1](https://www.ncbi.nlm.nih.gov/protein/AWT22235.1?report=genbank&log$=prottop&blast_rank=1&RID=4R6M9GRH016) |  |  |
| *GjilOBP57c* | c149886.graph_c0 | 164 | odorant binding protein OBP14 [Aphis glycines] | 76.00% | 3.00E-08 | 31.78% | 168 | | | [QYL02822.1](https://www.ncbi.nlm.nih.gov/protein/QYL02822.1?report=genbank&log$=prottop&blast_rank=3&RID=4R5W7DM4013) |  |  |
| *GjilOBP14a* | c143869.graph_c0 | 161 | odorant binding protein 9 [Ceracris nigricornis] | 69.00% | 0.009 | 28.70% | 157 | | | [QHR83103.1](https://www.ncbi.nlm.nih.gov/protein/QHR83103.1?report=genbank&log$=prottop&blast_rank=2&RID=4R50KBXV013) |  |  |
| *GjilOBP84a* | c149575.graph_c0 | 143 | odorant binding protein 36 [Nezara viridula] | 95.00% | 5.00E-13 | 29.71% | 163 | | | [QCZ25093.1](https://www.ncbi.nlm.nih.gov/protein/QCZ25093.1?report=genbank&log$=prottop&blast_rank=1&RID=4R7SE7Y3016) |  |  |
| *GjilOBP1* | c155875.graph_c1 | 147 | odorant binding protein 3 [Laodelphax striatellus] | 68.00% | 2.00E-17 | 36.00% | 120 | | | [AEQ19909.1](https://www.ncbi.nlm.nih.gov/protein/AEQ19909.1?report=genbank&log$=prottop&blast_rank=2&RID=4R6T0NVE016) |  |  |
| *GjilOBP19* | c156005.graph_c3 | 286 | odorant binding protein 17 [Ceracris nigricornis] | 37.00% | 1.00E-10 | 38.32% | 138 | | | [QHR83111.1](https://www.ncbi.nlm.nih.gov/protein/QHR83111.1?report=genbank&log$=prottop&blast_rank=3&RID=4R7ESHS8013) |  |  |
| *GjilOBP14* | c133413.graph_c0 | 164 | general odorant-binding protein 84a-like [Zootermopsis nevadensis] | 68.00% | 2.00E-29 | 43.36% | 143 | | | [XP_021916985.1](https://www.ncbi.nlm.nih.gov/protein/XP_021916985.1?report=genbank&log$=prottop&blast_rank=1&RID=4R5D6RF7013) |  |  |
| *GjilOBP8f* | c108483.graph_c0 | 140 | general odorant-binding protein 72 isoform X1 [Danaus plexippus plexippus] | 93.00% | 3.00E-34 | 46.56% | 139 | | | [XP_032518166.1](https://www.ncbi.nlm.nih.gov/protein/XP_032518166.1?report=genbank&log$=prottop&blast_rank=2&RID=4R64H1ZF013) |  |  |
| *GjilOBP8* | c150575.graph_c0 | 160 | general odorant-binding protein 57c isoform X2 [Zootermopsis nevadensis] | 87.00% | 3.00E-48 | 55.78% | 155 | | | [XP_021924930.1](https://www.ncbi.nlm.nih.gov/protein/XP_021924930.1?report=genbank&log$=prottop&blast_rank=2&RID=4R6FJ418013) |  |  |
| *GjilOBP14b* | c144608.graph_c0 | 141 | general odorant-binding protein 56a-like [Cryptotermes secundus] | 95.00% | 9.00E-17 | 32.61% | 152 | | | [XP_023719059.1](https://www.ncbi.nlm.nih.gov/protein/XP_023719059.1?report=genbank&log$=prottop&blast_rank=1&RID=4R61W87D01R) |  |  |
| *GjilOBP56* | c141764.graph_c0 | 113 | chemosensory protein [Blattella germanica] | 88.00% | 1.00E-09 | 35.00% | 135 | | | [AMA98134.1](https://www.ncbi.nlm.nih.gov/protein/AMA98134.1?report=genbank&log$=prottop&blast_rank=1&RID=4R743DC1013) |  |  |
| **Table S7**. List of CSP genes in *G. jilina* | | | |  |  |  |  | | |  |  |  |
| **Gene name** | **Unigene ID** | **Unigene** | **BLASTx best hit** | **Query cover** | **E** | **Ident** | **The Length of "Best hit" (aa)** | | | **ID** |  |  |
|  |  | **ORF (aa)** |  |  |  |  |  |  |  |  |  |  |
| *GjilCSP1b* | c174662.graph_c0 | 114 | Chemosensory protein 14 [Dendrolimus punctatus] | 100.00% | 4.00E-78 | 100.00% | 114 | | | [ARO70318.1](https://www.ncbi.nlm.nih.gov/protein/ARO70318.1?report=genbank&log$=prottop&blast_rank=1&RID=4RSFKKCD01R) |  |  |
| *GjilCSP14* | c143681.graph_c0 | 125 | chemosensory protein 4 [Mythimna separata] | 100.00% | 2.00E-78 | 91.34% | 127 | | | [AWT22250.1](https://www.ncbi.nlm.nih.gov/protein/AWT22250.1?report=genbank&log$=prottop&blast_rank=1&RID=4RRDVVF2013) |  |  |
| *GjilCSP5* | c155581.graph_c0 | 127 | chemosensory protein 6 [Mythimna separata] | 100.00% | 2.00E-66 | 93.70% | 127 | | | [AWT22260.1](https://www.ncbi.nlm.nih.gov/protein/AWT22260.1?report=genbank&log$=prottop&blast_rank=1&RID=4RRS6NTD013) |  |  |
| *GjilCSP1a* | c154976.graph_c1 | 128 | chemosensory protein 3 [Mythimna separata] | 98.00% | 8.00E-63 | 85.71% | 128 | | | [AWT22254.1](https://www.ncbi.nlm.nih.gov/protein/AWT22254.1?report=genbank&log$=prottop&blast_rank=2&RID=4RRYP2DC013) |  |  |
| *GjilCSP19* | c141764.graph_c0 | 113 | chemosensory protein [Blattella germanica] | 88.00% | 1.00E-09 | 35.00% | 135 | | | [AMA98134.1](https://www.ncbi.nlm.nih.gov/protein/AMA98134.1?report=genbank&log$=prottop&blast_rank=1&RID=4RS57TYS01R) |  |  |
| *GjilCSP3* | c159016.graph_c1 | 111 | putative chemosensory protein [Sesamia inferens] | 82.00% | 6.00E-32 | 56.52% | 111 | | | [AGY49261.1](https://www.ncbi.nlm.nih.gov/protein/AGY49261.1?report=genbank&log$=prottop&blast_rank=2&RID=4RS8YRUN013) |  |  |
| *GjilCSP2* | c154404.graph_c0 | 126 | chemosensory protein [Agrilus zanthoxylumi] | 76.00% | 2.00E-49 | 77.08% | 121 | | | [QTJ02340.1](https://www.ncbi.nlm.nih.gov/protein/QTJ02340.1?report=genbank&log$=prottop&blast_rank=1&RID=4RSCB3KY013) |  |  |
| **Table S8.** List of SNMP gene in *G. jilina* | | | |  |  |  |  | | |  |  |  |
| **Gene name** | **Unigene ID** | **Unigene** | **BLASTx best hit** | **Query cover** | **E** | **Ident** | **The Length of "Best hit" (aa)** | | | **ID** |  |  |
|  |  | **ORF (aa)** |  |  |  |  |  |  |  |  |  |  |
| *GjilSNMP1* | c158226.graph_c0 | 513 | sensory neuron membrane protein 1 [Cryptotermes secundus] | 96.00% | 3.00E-180 | 51.52% | 515 | | | [XP_023702700.1](https://www.ncbi.nlm.nih.gov/protein/XP_023702700.1?report=genbank&log$=prottop&blast_rank=3&RID=4RTWN6TX013) |  |  |
| **Table S9.** List of VSX gene in *G. jilina* | | | |  |  |  |  | | |  |  |  |
| **Gene name** | **Unigene ID** | **Unigene** | **BLASTx best hit** | **Query cover** | **E** | **Ident** | **The Length of "Best hit" (aa)** | | | **ID** |  |  |
|  |  | **ORF (aa)** |  |  |  |  |  |  |  |  |  |  |
| *GjilVSX2* | c74952.graph_c0 | 138 | visual system homeobox 2-like [Agrilus planipennis] | 86% | 9.00E-08 | 50.42% | 482 | | | XP_018321674.1 |  |  |
| **Table S10.** List of spire genes in *G. jilina* | | | |  |  |  |  | | |  |  |  |
| **Gene name** | **Unigene ID** | **Unigene** | **BLASTx best hit** | **Query cover** | **E** | **Ident** | **The Length of "Best hit" (aa)** | | | **ID** |  |  |
|  |  | **ORF (aa)** |  |  |  |  |  |  |  |  |  |  |
| *Gjilspire-c160217* | c160217.graph_c4 | 339 | Protein spire [Zootermopsis nevadensis] | 94% | 5.00E-164 | 85.09% | 403 | | | KDR08002.1 |  |  |
| *Gjilspire-c162025* | c162025.graph_c1 | 372 | protein spire isoform X4 [Schistocerca gregaria] | 99% | 0 | 71.43% | 783 | | | XP_049829496.1 |  |  |
| **Table S11.** List of Vg genes in *G. jilina* | | | |  |  |  |  | | |  |  |  |
| **Gene name** | **Unigene ID** | **Unigene** | **BLASTx best hit** | **Query cover** | **E** | **Ident** | **The Length of "Best hit" (aa)** | | | **ID** |  |  |
|  |  | **ORF (aa)** |  |  |  |  |  |  |  |  |  |  |
| *GjilVg-c125869* | c125869.graph_c0 | 207 | vitellogenin-like [Zootermopsis nevadensis] | 97% | 2.00E-31 | 33.66% | 1655 | | | XP_021925360.1 |  |  |
| *GjilVg-c177851* | c177851.graph_c0 | 139 | LOW QUALITY PROTEIN: vitellogenin-like [Lepeophtheirus salmonis] | 95% | 8.00E-15 | 32.84% | 1965 | | | XP_040568718.1 |  |  |
| **Table S12.** List of SPF genes in *G. jilina* | | | |  |  |  |  | | |  |  |  |
| **Gene name** | **Unigene ID** | **Unigene** | **BLASTx best hit** | **Query cover** | **E** | **Ident** | **The Length of "Best hit" (aa)** | | | **ID** |  |  |
|  |  | **ORF (aa)** |  |  |  |  |  |  |  |  |  |  |
| *GjilSPF-c160682* | c160682.graph_c2 | 91 | selenoprotein F [Cryptotermes secundus] | 100% | 3.00E-85 | 74.55% | 165 | | | XP_023702846.1 |  |  |
| *GjilSPF-c130288* | c130288.graph_c0 | 86 | selenoprotein F [Leptinotarsa decemlineata] | 91% | 2.00E-21 | 51.25% | 161 | | | XP_023018475.1 |  |  |
| *GjilSPF-c139251* | c139251.graph_c0 | 162 | selenoprotein F [Helicoverpa zea] | 100% | 8.00E-61 | 100.00% | 172 | | | XP_047023478.1 |  |  |
| **Table S13.** List of Tudor genes in *G. jilina* | | | |  |  |  |  | | |  |  |  |
| **Gene name** | **Unigene ID** | **Unigene** | **BLASTx best hit** | **Query cover** | **E** | **Ident** | **The Length of "Best hit" (aa)** | | | **ID** |  |  |
|  |  | **ORF (aa)** |  |  |  |  |  |  |  |  |  |  |
| *GjilTudor5a* | c157801.graph_c4 | 270 | tudor domain-containing protein 5 isoform X3 [Cryptotermes secundus] | 84% | 1.00E-103 | 40.62% | 1187 | | | XP_023708782.1 |  |  |
| *GjilTudor5b* | c157907.graph_c0 | 819 | tudor domain-containing protein 5 isoform X3 [Cryptotermes secundus] | 33% | 4.00E-16 | 41.67% | 1187 | | | XP_023708782.1 |  |  |
| *GjilTudor7* | c158490.graph_c1 | 1042 | tudor domain-containing protein 7 [Zootermopsis nevadensis] | 89% | 0 | 41.26% | 1161 | | | XP_021915680.1 |  |  |
| *GjilTudor1* | c132657.graph_c0 | 197 | tudor domain-containing protein 1 isoform X1 [Cryptotermes secundus] | 54% | 3.00E-26 | 44.37% | 1359 | | | XP_023703114.1 |  |  |
| *GjilTudor6a* | c161545.graph_c0 | 488 | tudor domain-containing protein 6 [Parasteatoda tepidariorum] | 32% | 1.00E-14 | 26.15% | 1208 | | | XP_015910632.2 |  |  |
| *GjilTudor6b* | c148839.graph_c0 | 315 | Tudor domain-containing protein 6 [Blomia tropicalis] | 94% | 6.00E-32 | 35.11% | 276 | | | KAI2797385.1 |  |  |
| **Table S14.** List of Dmrt gene in *G. jilina* | | | |  |  |  |  | | |  |  |  |
| **Gene name** | **Unigene ID** | **Unigene** | **BLASTx best hit** | **Query cover** | **E** | **Ident** | **The Length of "Best hit" (aa)** | | | **ID** |  |  |
|  |  | **ORF (aa)** |  |  |  |  |  |  |  |  |  |  |
| *GjilDmrt2* | c145436.graph_c0 | 265 | doublesex- and mab-3-related transcription factor 2 [Cryptotermes secundus] | 90% | 2.00E-98 | 67.59% | 257 | | | XP_023719106.1 |  |  |
| **Table S15.** List of SP genes in *G. jilina* | | | |  |  |  |  | | |  |  |  |
| **Gene name** | **Unigene ID** | **Unigene** | **BLASTx best hit** | **Query cover** | **E** | **Ident** | **The Length of "Best hit" (aa)** | | | **ID** |  |  |
|  |  | **ORF (aa)** |  |  |  |  |  |  |  |  |  |  |
| *GjilSP-c150060* | c150060.graph_c0 | 448 | sex peptide receptor-like [Orussus abietinus] | 90% | 1.00E-174 | 66.84% | 412 | | | XP_012287919.2 |  |  |
| *GjilSP-c150612* | c150612.graph_c0 | 344 | sex peptide receptor [Cryptotermes secundus] | 95% | 1.00E-169 | 65.97% | 456 | | | XP_023727878.1 |  |  |
| *GjilSP-c156016* | c156016.graph_c1 | 432 | sex peptide receptor [Cryptotermes secundus] | 100% | 0 | 88.44% | 377 | | | XP_023703235.1 |  |  |
| **Table S16.** List of Sox genes in *G. jilina* | | | |  |  |  |  | | |  |  |  |
| **Gene name** | **Unigene ID** | **Unigene** | **BLASTx best hit** | **Query cover** | **E** | **Ident** | **The Length of "Best hit" (aa)** | | | **ID** |  |  |
|  |  | **ORF (aa)** |  |  |  |  |  |  |  |  |  |  |
| *GjilSox8* | c148240.graph_c0 | 172 | transcription factor SOX-8-like [Cryptotermes secundus] | 65% | 1.00E-05 | 40.29% | 378 | | | XP_023705475.1 |  |  |
| *GjilSox21b* | c157424.graph_c1 | 63 | transcription factor SOX-21 [Cryptotermes secundus] | 100% | 5.00E-84 | 80.35% | 433 | | | XP_023704936.1 |  |  |
| *GjilSox2* | c156806.graph_c3 | 266 | transcription factor sox-2-like isoform X1 [Zootermopsis nevadensis] | 99% | 3.00E-128 | 61.33% | 430 | | | XP_021932033.1 |  |  |
| *GjilSox21a* | c94690.graph_c0 | 416 | transcription factor SOX-21 [Cryptotermes secundus] | 90% | 3.00E-14 | 59.09% | 433 | | | XP_023704936.1 |  |  |
| *GjilSox5* | c152747.graph_c0 | 562 | transcription factor SOX-5-like isoform X2 [Zootermopsis nevadensis] | 99% | 0 | 71.82% | 824 | | | XP_021927831.1 |  |  |
| *GjilSox3* | c153265.graph_c2 | 480 | transcription factor Sox-3-like [Cimex lectularius] | 61% | 4.00E-71 | 61.79% | 365 | | | XP_014248151.1 |  |  |
| **Table S17.** List of vasa gene in *G. jilina* | | | |  |  |  |  | | |  |  |  |
| **Gene name** | **Unigene ID** | **Unigene** | **BLASTx best hit** | **Query cover** | **E** | **Ident** | **The Length of "Best hit" (aa)** | | | **ID** |  |  |
|  |  | **ORF (aa)** |  |  |  |  |  |  |  |  |  |  |
| *Gjilvasa* | c151313.graph_c2 | 462 | vasa [Gryllus bimaculatus] | 100% | 0 | 70.94% | 650 | | | BAG65665.1 |  |  |
| **Table S18**. List of Trets genes in *G. jilina* | | | |  |  |  |  | | |  |  |  |
| **Gene name** | **Unigene ID** | **Unigene** | **BLASTx best hit** | **Query cover** | **E** | **Ident** | **The Length of "Best hit" (aa)** | | | **ID** |  |  |
|  |  | **ORF (aa)** |  |  |  |  |  |  |  |  |  |  |
| *GjilTret1-c154529* | c154529.graph_c0 | 521 | facilitated trehalose transporter Tret1-2 homolog [Zootermopsis nevadensis] | 90% | 2.00E-146 | 48.65% | 495 | | | XP_021938022.1 |  |  |
| *GjilTret1-c157976* | c157976.graph_c0 | 476 | facilitated trehalose transporter Tret1-like [Zootermopsis nevadensis] | 89% | 3.00E-143 | 50.70% | 489 | | | XP_021936749.1 |  |  |
| *GjilTret1-c152950* | c152950.graph_c0 | 484 | facilitated trehalose transporter Tret1-2 homolog isoform X1 [Zootermopsis nevadensis] | 99% | 2.00E-95 | 37.85% | 491 | | | XP_021938146.1 |  |  |
| *GjilTret1-c158889* | c158889.graph_c0 | 508 | facilitated trehalose transporter Tret1-like [Cryptotermes secundus] | 97% | 0 | 83.57% | 501 | | | XP_033609139.1 |  |  |
| *GjilTret1-c158686* | c158686.graph_c1 | 169 | facilitated trehalose transporter Tret1 isoform X4 [Cephus cinctus] | 94% | 1.00E-44 | 52.10% | 493 | | | XP_024941943.1 |  |  |
| *GjilTret1-c148117* | c148117.graph_c0 | 154 | facilitated trehalose transporter Tret1-2 homolog isoform X2 [Cryptotermes secundus] | 94% | 5.00E-42 | 54.55% | 420 | | | XP_023701754.1 |  |  |
| *GjilTret1-c149937* | c149937.graph_c0 | 486 | facilitated trehalose transporter Tret1-2 homolog [Cryptotermes secundus] | 95% | 8.00E-151 | 46.57% | 492 | | | XP_023704097.1 |  |  |
| *GjilTret1-c156045* | c156045.graph_c1 | 137 | facilitated trehalose transporter Tret1-like isoform X2 [Zootermopsis nevadensis] | 97% | 2.00E-58 | 68.42% | 489 | | | XP_021922628.1 |  |  |
| *GjilTret1-c191003* | c191003.graph_c0 | 86 | RecName: Full=Facilitated trehalose transporter Tret1 [Drosophila mojavensis] | 100% | 3.00E-23 | 47.67% | 863 | | | B4KR05.2 |  |  |
| *GjilTret1-c153320* | c153320.graph_c0 | 529 | facilitated trehalose transporter Tret1-2 homolog isoform X1 [Cryptotermes secundus] | 96% | 0 | 62.67% | 553 | | | XP_023718925.1 |  |  |
| *GjilTret1-c158599* | c158599.graph_c0 | 672 | facilitated trehalose transporter Tret1-like [Zootermopsis nevadensis] | 90% | 0 | 51.55% | 611 | | | XP_021942390.1 |  |  |
| *GjilTret1-c109012* | c109012.graph_c0 | 564 | facilitated trehalose transporter Tret1-like [Zootermopsis nevadensis] | 90% | 0 | 51.55% | 611 | | | XP_021942390.1 |  |  |
| *GjilTret1-c159702* | c159702.graph_c3 | 523 | Facilitated trehalose transporter Tret1-2 [Blattella germanica] | 85% | 2.00E-131 | 52.68% | 443 | | | PSN45090.1 |  |  |
| *GjilTret1-c150741* | c150741.graph_c1 | 201 | facilitated trehalose transporter Tret1-2 homolog [Cryptotermes secundus] | 98% | 5.00E-76 | 64.65% | 554 | | | XP_023724938.1 |  |  |
| *GjilTret1-c142827* | c142827.graph_c0 | 229 | facilitated trehalose transporter Tret1-like [Zootermopsis nevadensis] | 94% | 4.00E-82 | 57.41% | 370 | | | XP_021936732.1 |  |  |
| *GjilTret1-c147137* | c147137.graph_c0 | 362 | facilitated trehalose transporter Tret1 [Nilaparvata lugens] | 97% | 1.00E-50 | 34.83% | 485 | | | XP_022188380.2 |  |  |
| *GjilTret1-c156416* | c156416.graph_c1 | 243 | facilitated trehalose transporter Tret1 [Nilaparvata lugens] | 97% | 1.00E-50 | 34.83% | 485 | | | XP_022188380.2 |  |  |
| *GjilTret1-c146574* | c146574.graph_c0 | 230 | facilitated trehalose transporter Tret1 [Nilaparvata lugens] | 97% | 1.00E-50 | 34.83% | 485 | | | XP_022188380.2 |  |  |
| *GjilTret1-c149661* | c149661.graph_c1 | 520 | facilitated trehalose transporter Tret1-2 homolog isoform X2 [Cryptotermes secundus] | 100% | 0 | 76.62% | 526 | | | XP_023702264.1 |  |  |
| *GjilTret1-c160767* | c160767.graph_c3 | 535 | facilitated trehalose transporter Tret1-2 homolog isoform X2 [Zootermopsis nevadensis] | 100% | 0 | 65.98% | 529 | | | XP_021926811.1 |  |  |
| *GjilTret1-c145952* | c145952.graph_c1 | 171 | facilitated trehalose transporter Tret1-2 homolog isoform X2 [Zootermopsis nevadensis] | 100% | 0 | 65.98% | 529 | | | XP_021926811.1 |  |  |
| *GjilTret1-c157869* | c157869.graph_c0 | 615 | facilitated trehalose transporter Tret1-2 homolog isoform X2 [Zootermopsis nevadensis] | 100% | 0 | 65.98% | 529 | | | XP_021926811.1 |  |  |
| *GjilTret1-c156385* | c156385.graph_c1 | 479 | facilitated trehalose transporter Tret1 [Cryptotermes secundus] | 98% | 0 | 64.27% | 490 | | | XP_023719423.1 |  |  |
| *GjilTret1-c133578* | c133578.graph_c0 | 544 | facilitated trehalose transporter Tret1 [Cryptotermes secundus] | 98% | 0 | 64.27% | 490 | | | XP_023719423.1 |  |  |
| *GjilTret1-c141346* | c141346.graph_c0 | 88 | facilitated trehalose transporter Tret1 [Cryptotermes secundus] | 98% | 0 | 64.27% | 490 | | | XP_023719423.1 |  |  |
| *GjilTret1-c156287* | c156287.graph_c2 | 554 | facilitated trehalose transporter Tret1-2 homolog isoform X2 [Schistocerca gregaria] | 91% | 5.00E-170 | 54.79% | 522 | | | XP_049845304.1 |  |  |
| *GjilTret1-c153918* | c153918.graph_c0 | 508 | facilitated trehalose transporter Tret1-2 homolog isoform X1 [Zootermopsis nevadensis] | 95% | 9.00E-132 | 44.60% | 491 | | | XP_021938146.1 |  |  |
| *GjilTret1-c153373* | c153373.graph_c0 | 489 | facilitated trehalose transporter Tret1-2 homolog [Cryptotermes secundus] | 97% | 3.00E-154 | 48.95% | 492 | | | XP_023704097.1 |  |  |
| *GjilTret1-c158653* | c158653.graph_c0 | 366 | facilitated trehalose transporter Tret1-2 homolog [Cryptotermes secundus] | 98% | 6.00E-91 | 45.63% | 525 | | | XP_023724494.1 |  |  |
| *GjilTret1-c126200* | c126200.graph_c2 | 156 | facilitated trehalose transporter Tret1-2 homolog [Spodoptera frugiperda] | 100% | 4.00E-98 | 95.51% | 490 | | | XP_035454471.2 |  |  |
| *GjilTret1-c153660* | c153660.graph_c0 | 871 | facilitated trehalose transporter Tret1-2 homolog [Spodoptera frugiperda] | 100% | 4.00E-98 | 95.51% | 490 | | | XP_035454471.2 |  |  |
| *GjilTret1-c108607* | c108607.graph_c0 | 160 | facilitated trehalose transporter Tret1 isoform X7 [Spodoptera frugiperda] | 100% | 3.00E-99 | 93.12% | 482 | | | XP_035444815.1 |  |  |
| *GjilTret1-c156818* | c156818.graph_c2 | 510 | facilitated trehalose transporter Tret1 [Cryptotermes secundus] | 88% | 1.00E-96 | 39.13% | 469 | | | XP_023714937.1 |  |  |
| **Table S19.** List of HSP genes in *G. jilina* | | | |  |  |  |  | | |  |  |  |
| **Gene name** | **Unigene ID** | **Unigene** | **BLASTx best hit** | **Query cover** | **E** | **Ident** | **The Length of "Best hit" (aa)** | | | **ID** |  |  |
|  |  | **ORF (aa)** |  |  |  |  |  |  |  |  |  |  |
| *GjilHSP6A* | c132703.graph_c0 | 141 | heat shock protein 75 kDa, mitochondrial [Sus scrofa] | 1 | 7.00E-52 | 1 | 702 | | | NP_001230874.1 |  |  |
| *GjilHSP4.1* | c165434.graph_c0 | 259 | heat shock protein 70 [Elliptio complanata] | 0.96 | 3.00E-58 | 0.8627 | 210 | | | AJF96695.1 |  |  |
| *GjilHSP11.8* | c146755.graph_c1 | 813 | heat-shock protein [Arabidopsis thaliana] | 0.76 | 3.00E-147 | 0.9714 | 246 | | | BAD95030.1 |  |  |
| *GjilHSP31.2* | c105662.graph_c0 | 106 | PREDICTED: heat shock protein 68-like [Papilio polytes] | 0.99 | 0 | 0.806 | 633 | | | XP_013148831.1 |  |  |
| *GjilHSP70* | c160917.graph_c3 | 638 | PREDICTED: heat shock protein 90-1-like [Raphanus sativus] | 1 | 2.00E-69 | 0.9237 | 255 | | | XP_018457177.1 |  |  |
| *GjilHSP13.4A* | c155920.graph_c2 | 344 | 97 kDa heat shock protein [Cryptotermes secundus] | 1 | 0 | 0.7337 | 828 | | | XP_023714084.1 |  |  |
| *GjilHSP91.8* | c162447.graph_c1 | 201 | heat shock protein 60A [Ischnura elegans] | 0.99 | 0 | 0.8638 | 572 | | | XP_046397660.1 |  |  |
| *GjilHSP35.2* | c154696.graph_c1 | 300 | 22.7 kDa class IV heat shock protein-like [Quercus lobata] | 0.93 | 3.00E-42 | 0.7234 | 187 | | | XP_030940488.1 |  |  |
| *GjilHSP11.5* | c140696.graph_c0 | 89 | small heat shock protein, chloroplastic-like [Pistacia vera] | 1 | 5.00E-53 | 0.7778 | 229 | | | XP_031256898.1 |  |  |
| *GjilHSP13.4B* | c79404.graph_c0 | 78 | CI small heat shock protein 2 [Prunus salicina] | 1 | 2.00E-72 | 0.8182 | 154 | | | ACV93249.1 |  |  |
| *GjilHSP5.5* | c211160.graph_c0 | 115 | heat shock protein 75 kDa, mitochondrial [Cryptotermes secundus] | 1 | 0 | 0.761 | 704 | | | XP_023725398.1 |  |  |
| *GjilHSP79.6* | c153645.graph_c0 | 118 | 17.9 kDa class II heat shock protein [Ziziphus jujuba var. spinosa] | 0.85 | 5.00E-66 | 0.8947 | 156 | | | XP_015884537.1 |  |  |
| *GjilHSP17.5* | c147453.graph_c0 | 156 | heat shock protein 60A [Cryptotermes secundus] | 0.92 | 0 | 0.8679 | 575 | | | XP_023712040.1 |  |  |
| *GjilHSP61.3* | c154983.graph_c0 | 733 | 60 kDa heat shock protein, mitochondrial [Phacochoerus africanus] | 0.97 | 0 | 1 | 573 | | | XP_047629105.1 |  |  |
| *GjilHSP60.9* | c143909.graph_c0 | 706 | heat shock protein 27.2 [Mythimna separata] | 0.92 | 7.00E-128 | 0.8419 | 249 | | | ATN45246.1 |  |  |
| *GjilHSP25.4* | c153210.graph_c0 | 648 | heat shock protein 90 [Mythimna separata] | 1 | 4.00E-73 | 0.9913 | 789 | | | ATN45250.1 |  |  |
| *GjilHSP13B* | c78856.graph_c0 | 101 | heat shock cognate 71 kDa protein isoform X6 [Canis lupus dingo] | 1 | 0 | 0.9971 | 586 | | | XP_048965810.1 |  |  |
| *GjilHSP37.9* | c149962.graph_c0 | 399 | RecName: Full=Heat shock protein 82 [Nicotiana tabacum] | 1 | 6.00E-162 | 0.9459 | 499 | | | P36182.1 |  |  |
| *GjilHSP30* | c155920.graph_c3 | 379 | PREDICTED: 10 kDa heat shock protein, mitochondrial [Erinaceus europaeus] | 1 | 4.00E-56 | 0.9804 | 102 | | | XP_007537962.1 |  |  |
| *GjilHSP11* | c130737.graph_c0 | 113 | Heat shock protein HSP 90-alpha [Fragariocoptes setiger] | 1 | 4.00E-30 | 0.9524 | 723 | | | KAG9509817.1 |  |  |
| *GjilHSP6B* | c126079.graph_c0 | 102 | heat shock 70 kDa protein 4 [Sus scrofa] | 1 | 3.00E-172 | 1 | 840 | | | XP_005661711.1 |  |  |
| *GjilHSP35.5* | c144480.graph_c0 | 325 | heat shock protein 90 alpha family class B member 1 [Myotis myotis] | 1 | 0 | 1 | 602 | | | KAF6341341.1 |  |  |
| *GjilHSP12.4* | c155920.graph_c6 | 52 | 60 kDa heat shock protein [uncultured Ehrlichia sp.] | 0.66 | 0.0003 | 0.5455 | 348 | | | BCG61751.1 |  |  |
| *GjilHSP10.1* | c175557.graph_c0 | 81 | Alpha crystallin/Hsp20 domain-containing protein [Cinnamomum micranthum f. kanehirae] | 0.79 | 0.0008 | 0.3804 | 212 | | | RWR97319.1 |  |  |
| *GjilHSP44* | c155920.graph_c0 | 724 | heat shock protein cognate 5 [Histiostomatidae gen. sp. AD692] | 0.9 | 0 | 0.9724 | 571 | | | AFJ22334.1 |  |  |
| *GjilHSP43.8* | c149147.graph_c0 | 125 | Heat shock protein HSP 90-alpha [Camelus dromedarius] | 0.91 | 0 | 1 | 745 | | | KAB1277738.1 |  |  |
| *GjilHSP84.8* | c158936.graph_c0 | 50 | Heat shock protein Hsp90 [Corchorus olitorius] | 1 | 7.00E-174 | 0.9467 | 651 | | | OMP06063.1 |  |  |
| *GjilHSP34.8* | c108774.graph_c0 | 83 | Heat shock protein [Eschrichtius robustus] | 1 | 7.00E-78 | 0.9831 | 379 | | | MBW01164.1 |  |  |
| *GjilHSP13.1* | c180061.graph_c0 | 582 | heat shock 70 kDa protein 15-like [Prunus avium] | 0.81 | 3.00E-37 | 0.9853 | 855 | | | XP_021829102.1 |  |  |
| *GjilHSP9.4* | c168152.graph_c0 | 118 | small heat shock protein, chloroplastic-like [Pistacia vera] | 1 | 1.00E-58 | 0.5467 | 211 | | | XP_031248490.1 |  |  |
| *GjilHSP23* | c107363.graph_c0 | 573 | heat shock protein-like protein [Coptotermes formosanus] | 0.85 | 3.00E-46 | 0.5823 | 181 | | | AGM32453.1 |  |  |
| *GjilHSP9.2* | c152198.graph_c1 | 39 | heat shock protein 90 [Neocloeon triangulifer] | 1 | 6.00E-79 | 0.9044 | 224 | | | AEE01388.1 |  |  |
| *GjilHSP14.9* | c157031.graph_c1 | 98 | putative HSP100 protein [Mucor mucedo] | 1 | 1.00E-53 | 0.9894 | 891 | | | XP_051458699.1 |  |  |
| *GjilHSP18* | c132603.graph_c0 | 156 | 10 kda heat shock protein [Blomia tropicalis] | 0.7 | 6.00E-48 | 0.7374 | 100 | | | KAI2800118.1 |  |  |
| *GjilHSP15.2* | c153553.graph_c0 | 305 | major heat shock 70 kDa protein Ba [Agrilus planipennis] | 0.98 | 0 | 0.7782 | 626 | | | XP_018332229.1 |  |  |
| *GjilHSP64.7* | c158962.graph_c2 | 51 | heat shock protein 90 [Rhizopus stolonifer] | 0.83 | 3.00E-42 | 0.9865 | 504 | | | RCI02057.1 |  |  |
| *GjilHSP10.4A* | c197110.graph_c0 | 193 | heat shock protein 40 [Oxya chinensis] | 0.98 | 1.00E-41 | 0.9 | 347 | | | AFN08645.1 |  |  |
| *GjilHSP9* | c156291.graph_c2 | 227 | heat shock protein 83 [Cryptotermes secundus] | 1 | 0 | 0.8803 | 727 | | | XP_023720867.1 |  |  |
| *GjilHSP83.3* | c161227.graph_c6 | 575 | Small heat shock protein HSP [Parasponia andersonii] | 0.99 | 5.00E-74 | 0.8085 | 143 | | | PON55550.1 |  |  |
| *GjilHSP10.4B* | c73273.graph_c0 | 106 | PREDICTED: heat shock cognate 70 kDa protein 2 [Theobroma cacao] | 1 | 0 | 0.9738 | 646 | | | XP_007027052.2 |  |  |
| *GjilHSP71.2* | c156111.graph_c0 | 116 | 10 kDa heat shock protein, mitochondrial [Cephus cinctus] | 0.97 | 9.00E-46 | 0.72 | 104 | | | XP_015601248.1 |  |  |
| *GjilHSP21* | c154721.graph_c0 | 100 | heat shock protein 90-like protein [Blattamonas nauphoetae] | 0.74 | 3.00E-42 | 0.9367 | 717 | | | AXC08495.1 |  |  |
| *GjilHSP11.9* | c115803.graph_c0 | 275 | 10 kDa heat shock protein, mitochondrial [Athalia rosae] | 0.98 | 1.00E-57 | 0.8544 | 104 | | | XP_012257765.1 |  |  |
| *GjilHSP13A* | c163949.graph_c0 | 136 | Human Hsp90-beta with PU3 (9-Butyl-8(3,4,5-trimethoxy-benzyl)-9H-purin-6-ylamine) [Homo sapiens] | 0.68 | 5.00E-86 | 1 | 220 | | | 1UYM_A |  |  |
| **Table S20.** List of DnaJ genes in *G. jilina* | | | |  |  |  |  | | |  |  |  |
| **Gene name** | **Unigene ID** | **Unigene** | **BLASTx best hit** | **Query cover** | **E** | **Ident** | **The Length of "Best hit" (aa)** | | | **ID** |  |  |
|  |  | **ORF (aa)** |  |  |  |  |  |  |  |  |  |  |
| *GjilDnaJA2* | c157023.graph_c1 | 342 | dnaJ homolog subfamily A member 2-like isoform X2 [Zootermopsis nevadensis] | 1 | 0 | 0.8538 | 444 | | | XP_021928687.1 |  |  |
| *GjilDnaJB9* | c75412.graph_c0 | 173 | dnaJ homolog subfamily B member 9 isoform X1 [Phacochoerus africanus] | 1 | 3.00E-108 | 1 | 257 | | | XP_047618959.1 |  |  |
| *GjilDnaJB4* | c187495.graph_c0 | 66 | PREDICTED: dnaJ homolog subfamily B member 4 isoform X2 [Erinaceus europaeus] | 1 | 9.00E-39 | 0.9848 | 177 | | | XP_016048471.1 |  |  |
| *GjilDnaJB1* | c120154.graph_c1 | 96 | dnaJ homolog subfamily B member 1-like [Mangifera indica] | 1 | 3.00E-47 | 0.8333 | 319 | | | XP_044492045.1 |  |  |
| *GjilDnaJA4* | c133876.graph_c0 | 234 | dnaJ homolog subfamily A member 1 [Schistocerca piceifrons] | 0.99 | 6.00E-109 | 0.6793 | 400 | | | XP_047100745.1 |  |  |
| *GjilDnaJB11* | c129815.graph_c1 | 154 | DnaJ heat shock protein family (Hsp40) member B11 [Phyllostomus discolor] | 0.99 | 9.00E-90 | 0.9935 | 169 | | | KAF6118318.1 |  |  |
| *GjilDnaJC14* | c177758.graph_c0 | 97 | PREDICTED: dnaJ homolog [Prunus dulcis] | 1 | 1.00E-63 | 1 | 590 | | | VVA38102.1 |  |  |
| *GjilDnaJC7* | c145581.graph_c0 | 382 | PREDICTED: dnaJ homolog subfamily C member 7 isoform X2 [Ceratotherium simum simum] | 1 | 0 | 0.9895 | 507 | | | XP_014646154.1 |  |  |
| *GjilDnaJA1* | c149111.graph_c0 | 397 | dnaJ homolog subfamily A member 1-like [Vulpes lagopus] | 1 | 0 | 0.9975 | 397 | | | XP_041609732.1 |  |  |
| *GjilDnaJA1L* | c82960.graph_c0 | 89 | dnaJ homolog subfamily A member 1 [Leptidea sinapis] | 0.84 | 6.00E-21 | 0.7532 | 400 | | | XP_050681376.1 |  |  |
| *GjilDnaJ* | c135797.graph_c0 | 50 | dnaJ protein homolog [Prunus persica] | 0.94 | 3.00E-24 | 1 | 417 | | | XP_007211787.1 |  |  |
| **Table S21.** List of TRP genes in *G. jilina* | | | |  |  |  |  | | |  |  |  |
| **Gene name** | **Unigene ID** | **Unigene** | **BLASTx best hit** | **Query cover** | **E** | **Ident** | **The Length of "Best hit" (aa)** | | | **ID** |  |  |
|  |  | **ORF (aa)** |  |  |  |  |  |  |  |  |  |  |
| *GjilTRPV-c159424* | c159424.graph_c2 | 989 | transient receptor potential channel pyrexia [Cryptotermes secundus] | 0.97 | 0 | 0.5887 | 1010 | | | XP_023708280.1 |  |  |
| *GjilTRPV-c152094_0* | c152094.graph_c0 | 710 | transient receptor potential cation channel protein painless isoform X1 [Cryptotermes secundus] | 0.99 | 0 | 0.4513 | 985 | | | XP_023714371.1 |  |  |
| *GjilTRPC-c181968* | c181968.graph_c0 | 103 | transient receptor potential cation channel subfamily C member 4 associated protein [Rhinolophus ferrumequinum] | 1 | 2.00E-66 | 1 | 370 | | | KAF6285031.1 |  |  |
| *GjilTRPV-c152094_1* | c152094.graph_c1 | 269 | transient receptor potential cation channel protein painless-like [Zootermopsis nevadensis] | 0.98 | 1.00E-42 | 0.4291 | 998 | | | XP_021924797.1 |  |  |
| *GjilTRPV-c154026* | c154026.graph_c0 | 1286 | transient receptor potential cation channel protein painless isoform X1 [Cryptotermes secundus] | 0.83 | 1.00E-156 | 0.3384 | 1182 | | | XP_023727973.1 |  |  |
| *GjilTRPM-c159924* | c159924.graph_c3 | 699 | transient receptor potential cation channel trpm isoform X1 [Zootermopsis nevadensis] | 1 | 0 | 0.6838 | 1703 | | | XP_021919466.1 |  |  |
| *GjilTRPV-c136561* | c136561.graph_c0 | 178 | transient receptor potential channel pyrexia-like isoform X3 [Zootermopsis nevadensis] | 0.91 | 1.00E-48 | 0.5333 | 862 | | | XP_021940800.1 |  |  |
| *GjilTRPV-c157412* | c157412.graph_c0 | 332 | transient receptor potential cation channel subfamily V member 4 isoform X2 [Cryptotermes secundus] | 0.99 | 4.00E-27 | 0.4139 | 1125 | | | XP_023708446.1 |  |  |
| *GjilTRPV-c138821* | c138821.graph_c1 | 93 | transient receptor potential cation channel protein painless isoform X1 [Cryptotermes secundus] | 0.65 | 2.00E-10 | 0.5082 | 985 | | | XP_023714371.1 |  |  |
| *GjilTRPV-c80333* | c80333.graph_c0 | 188 | transient receptor potential cation channel protein painless-like [Zootermopsis nevadensis] | 0.97 | 6E-08 | 0.299 | 998 | | | XP_021924797.1 |  |  |
| *GjilTRPV-c132635* | c132635.graph_c0 | 334 | transient receptor potential cation channel protein painless isoform X2 [Cryptotermes secundus] | 0.91 | 4.00E-42 | 0.3446 | 961 | | | XP_023714372.1 |  |  |
| *GjilTRPV-c152131* | c152131.graph_c0 | 505 | transient receptor potential cation channel protein painless-like [Zootermopsis nevadensis] | 0.92 | 4.00E-105 | 0.411 | 998 | | | XP_021924797.1 |  |  |
| *GjilTRPC-c181773* | c181773.graph_c0 | 105 | short transient receptor potential channel 4-associated protein isoform X5 [Orcinus orca] | 1 | 4.00E-68 | 1 | 736 | | | XP_049554850.1 |  |  |
| *GjilTRPV-c157578* | c157578.graph_c5 | 910 | transient receptor potential channel pyrexia isoform X1 [Cryptotermes secundus] | 0.99 | 0 | 0.6195 | 949 | | | XP_023718298.1 |  |  |
| *GjilTRPV-c157440* | c157440.graph_c0 | 852 | transient receptor potential cation channel protein painless isoform X1 [Cryptotermes secundus] | 0.93 | 7.00E-99 | 0.2918 | 1182 | | | XP_023727973.1 |  |  |
| *GjilTRPV-c154869* | c154869.graph_c0 | 682 | transient receptor potential cation channel protein painless-like [Schistocerca gregaria] | 0.89 | 3.00E-80 | 0.3824 | 1019 | | | XP_049858155.1 |  |  |
| *GjilTRPV-c153624* | c153624.graph_c0 | 277 | transient receptor potential cation channel subfamily V member 5 [Schistocerca americana] | 0.93 | 8.00E-157 | 0.9077 | 793 | | | XP_046993972.1 |  |  |
| *GjilTRPV-c96896* | c96896.graph_c0 | 108 | transient receptor potential channel pyrexia-like [Lepeophtheirus salmonis] | 0.61 | 0.00006 | 0.4697 | 955 | | | XP_040573146.1 |  |  |
| *GjilTRPTγ-c160130* | c160130.graph_c1 | 641 | transient receptor potential-gamma protein isoform X1 [Cryptotermes secundus] | 1 | 0 | 0.9019 | 1210 | | | XP_033609925.1 |  |  |
| **Table S22.** List of Wnt genes in *G. jilina* | | | |  |  |  |  | | |  |  |  |
| **Gene name** | **Unigene ID** | **Unigene** | **BLASTx best hit** | **Query cover** | **E** | **Ident** | **The Length of "Best hit" (aa)** | | | **ID** |  |  |
|  |  | **ORF (aa)** |  |  |  |  |  |  |  |  |  |  |
| *GjilWnt10b* | c143916.graph_c0 | 262 | Protein Wnt-10b [Zootermopsis nevadensis] | 100% | 3.00E-121 | 73.18% | 352 | | | KDR20619.1 |  |  |
| *GjilWnt7b* | c144605.graph_c0 | 371 | protein Wnt-7b [Cryptotermes secundus] | 97% | 0 | 82.60% | 371 | | | XP_023715845.1 |  |  |
| *GjilWnt11* | c145594.graph_c0 | 93 | Protein Wnt-11 [Zootermopsis nevadensis] | 67% | 9.00E-27 | 71.43% | 313 | | | KDR22144.1 |  |  |
| *GjilWnt6* | c151829.graph_c0 | 355 | protein Wnt-6 [Cryptotermes secundus] | 100% | 0 | 83.01% | 359 | | | XP_023701471.1 |  |  |
| *GjilWnt16* | c156537.graph_c3 | 391 | protein Wnt-16-like [Homalodisca vitripennis] | 93% | 0 | 79.35% | 385 | | | XP_046684210.1 |  |  |
| *GjilWnt1* | c81087.graph_c0 | 181 | wingless [Halyomorpha halys] | 100% | 4.00E-98 | 82.22% | 289 | | | KAE8574038.1 |  |  |
| **Table S23**. List of EcR genes in *G. jilina* | | | |  |  |  |  | | |  |  |  |
| **Gene name** | **Unigene ID** | **Unigene** | **BLASTx best hit** | **Query cover** | **E** | **Ident** | **The Length of "Best hit" (aa)** | | | **ID** |  |  |
|  |  | **ORF (aa)** |  |  |  |  |  |  |  |  |  |  |
| *GjilEcRA1* | c206303.graph_c0 | 366 | ecdysone receptor-like isoform X1 [Dermatophagoides pteronyssinus] | 87% | 7.00E-31 | 77.92% | 812 | | | XP_027197658.1 |  |  |
| *GjilEcRA2* | c156696.graph_c1 | 87 | ecdysone receptor isoform A [Diploptera punctata] | 65% | 1.00E-80 | 66.28% | 538 | | | AEZ64363.1 |  |  |
| **Table S24**. List of E75 gene in *G. jilina* | | | |  |  |  |  | | |  |  |  |
| **Gene name** | **Unigene ID** | **Unigene** | **BLASTx best hit** | **Query cover** | **E** | **Ident** | **The Length of "Best hit" (aa)** | | | **ID** |  |  |
|  |  | **ORF (aa)** |  |  |  |  |  |  |  |  |  |  |
| *Gjil75* | c185337.graph_c0 | 90 | ecdysone-inducible protein E75 isoform X4 [Zootermopsis nevadensis] | 94% | 3.00E-38 | 76.47% | 858 | | | XP_021938070.1 |  |  |
| **Table S25**. List of InsR gene in *G. jilina* | | | |  |  |  |  | | |  |  |  |
| **Gene name** | **Unigene ID** | **Unigene** | **BLASTx best hit** | **Query cover** | **E** | **Ident** | **The Length of "Best hit" (aa)** | | | **ID** |  |  |
|  |  | **ORF (aa)** |  |  |  |  |  |  |  |  |  |  |
| *GjilInsR* | c157735.graph_c0 | 1688 | insulin-like receptor isoform X2 [Cryptotermes secundus] | 81% | 0 | 64.15% | 1626 | | | XP_023705699.1 |  |  |
| **Table S26.** List of IGFBP genes in *G. jilina* | | | |  |  |  |  | | |  |  |  |
| **Gene name** | **Unigene ID** | **Unigene** | **BLASTx best hit** | **Query cover** | **E** | **Ident** | **The Length of "Best hit" (aa)** | | | **ID** |  |  |
|  |  | **ORF (aa)** |  |  |  |  |  |  |  |  |  |  |
| *GjilIGFBP1* | c160838.graph_c3 | 761 | insulin-like growth factor-binding protein complex acid labile subunit [Spodoptera frugiperda] | 100% | 0 | 87.02% | 865 | | | XP_035445676.2 |  |  |
| *GjilIGFBP2* | c154533.graph_c0 | 755 | insulin-like growth factor-binding protein complex acid labile subunit [Cryptotermes secundus] | 100% | 5.00E-129 | 35.56% | 774 | | | XP_023706930.1 |  |  |
| *GjilIGFBP3* | c160874.graph_c3 | 524 | insulin-like growth factor-binding protein complex acid labile subunit isoform X2 [Cryptotermes secundus] | 99% | 0 | 81.96% | 530 | | | XP_023725318.1 |  |  |
| *GjilIGFBP4* | c156044.graph_c0 | 551 | insulin-like growth factor 2 mRNA-binding protein 1 isoform X3 [Cryptotermes secundus] | 94% | 0 | 85.93% | 553 | | | XP_023703837.1 |  |  |
| **Table S27.** List of ILP gene in *G. jilina* | | | |  |  |  |  | | |  |  |  |
| **Gene name** | **Unigene ID** | **Unigene** | **BLASTx best hit** | **Query cover** | **E** | **Ident** | **The Length of "Best hit" (aa)** | | | **ID** |  |  |
|  |  | **ORF (aa)** |  |  |  |  |  |  |  |  |  |  |
| *GjilILP2* | c144860.graph_c0 | 135 | Insulin-like Peptide 2 (ILP2) [Blattella germanica] | 100% | 8.00E-32 | 57.78% | 151 | | | SOX29888.1 |  |  |
| **Table S28.** List of JHE gene in *G. jilina* | | | |  |  |  |  | | |  |  |  |
| **Gene name** | **Unigene ID** | **Unigene** | **BLASTx best hit** | **Query cover** | **E** | **Ident** | **The Length of "Best hit" (aa)** | | | **ID** |  |  |
|  |  | **ORF (aa)** |  |  |  |  |  |  |  |  |  |  |
| *GjilJHE* | c75715.graph_c0 | 60 | juvenile hormone esterase [Mythimna separata] | 100% | 6.00E-17 | 61.67% | 582 | | | ACO81854.2 |  |  |
| **Table S29.** List of JHBP genes in *G. jilina* | | | |  |  |  |  | | |  |  |  |
| **Gene name** | **Unigene ID** | **Unigene** | **BLASTx best hit** | **Query cover** | **E** | **Ident** | **The Length of "Best hit" (aa)** | | | **ID** |  |  |
|  |  | **ORF (aa)** |  |  |  |  |  |  |  |  |  |  |
| *GjilJHBP4a* | c149187.graph_c0 | 245 | juvenile hormone binding protein 4 [Scylla paramamosain] | 76% | 3.00E-17 | 29.26% | 435 | | | QDP16282.1 |  |  |
| *GjilJHBP2* | c156379.graph_c0 | 256 | Juvenile hormone-binding protein 2 [Mythimna separata] | 90% | 8.00E-119 | 88.36% | 232 | | | UXX34492.1 |  |  |
| *GjilJHBP4b* | c163893.graph_c0 | 275 | hemolymph juvenile hormone binding protein [Coptotermes formosanus] | 87% | 4.00E-27 | 32.10% | 263 | | | AGM32080.1 |  |  |
| **Table S30.** List of GCL gene in *G. jilina* | | | |  |  |  |  | | |  |  |  |
| **Gene name** | **Unigene ID** | **Unigene** | **BLASTx best hit** | **Query cover** | **E** | **Ident** | **The Length of "Best hit" (aa)** | | | **ID** |  |  |
|  |  | **ORF (aa)** |  |  |  |  |  |  |  |  |  |  |
| *GjilGCL* | c149187.graph_c0 | 424 | Protein germ cell-less [Blattella germanica] | 99% | 3.00E-149 | 51.54% | 500 | | | PSN37223.1 |  |  |
| **Table S31.** List of Met gene in *G. jilina* | | | |  |  |  |  | | |  |  |  |
| **Gene name** | **Unigene ID** | **Unigene** | **BLASTx best hit** | **Query cover** | **E** | **Ident** | **The Length of "Best hit" (aa)** | | | **ID** |  |  |
|  |  | **ORF (aa)** |  |  |  |  |  |  |  |  |  |  |
| *GjilMet* | c156601.graph_c2 | 634 | methoprene-tolerant [Blattella germanica] | 76% | 2.00E-128 | 47.28% | 952 | | | UUR40056.1 |  |  |
| **Table S32.** List of EGFR gene in *G. jilina* | | | |  |  |  |  | | |  |  |  |
| **Gene name** | **Unigene ID** | **Unigene** | **BLASTx best hit** | **Query cover** | **E** | **Ident** | **The Length of "Best hit" (aa)** | | | **ID** |  |  |
|  |  | **ORF (aa)** |  |  |  |  |  |  |  |  |  |  |
| *GjilEGFR* | c156432.graph_c0 | 1418 | Epidermal growth factor receptor [Blattella germanica] | 96% | 0 | 76.50% | 1452 | | | CEG62429.1 |  |  |
